# Supplementary material for: Folding kinetics of an entangled protein
Source: PLoS Comput Biol. 2023 Nov 13;19(11):e1011107. doi: 10.1371/journal.pcbi.1011107 (PMC10681328; doi:10.1371/journal.pcbi.1011107)
Supplement: S1 Appendix — (PDF) [file pcbi.1011107.s001.pdf]

Folding kinetics of an entangled protein

Leonardo Salicari[1,2], Marco Baiesi[1,2], Enzo Orlandini[1,2], Antonio Trovato[1,2*]

1 Department of Physics and Astronomy 'G. Galilei', University of Padova, Padova, Italy

2 National Institute of Nuclear Physics (INFN), Padova Section, Padova, Italy

* antonio.trovato@unipd.it

Supporting Information

TABLE A: This table shows RD1 contact map computed from the experimental structure. Two residues, satisfying $|i - j| > 3$, are said to form a native contact if any two non-Hydrogen atoms in different residues are closer than 4.5 Å. The classification (**Class** column) for each contact, as used in the main text, is the following: en = entangled contacts, fe = first-entangling, and ta = trap-avoiding. Two residues are involved in an entangled contact if the maximum modulus of the Gaussian entanglement, for the loop defined by these, satisfies $|G'| > 0.75$. First-entangling and trap-avoiding contacts definitions are reported in the Material and Methods' section "Ensemble definition and pathway classification".

**Res 1**	**Res 2**	**Class**	**Res 1**	**Res 2**	**Class**	**Res 1**	**Res 2**	**Class**
1	26		8	20		22	41	en
1	27	ta	8	21		23	37	en, fe
1	28		8	22		24	34	en
1	30		8	23		24	37	en, fe
2	26	ta	8	51		27	54	
2	30	ta	9	20		29	54	
2	35	ta	9	21		29	56	
3	24		9	49		30	54	
3	25		9	50		30	56	
3	26		9	51		31	54	
3	30	ta	10	48		31	55	
3	32	ta	10	49		31	56	
3	33		10	50		32	36	
3	34		10	51		32	40	
3	35	ta	11	15		32	55	
4	23		11	17		32	56	

Continued on next page...

TABLE A – continued from previous page.

Res 1	Res 2	Class	Res 1	Res 2	Class	Res 1	Res 2	Class
4	24		11	21		32	57	
4	25	ta	11	45		32	60	
4	27	ta	11	47		32	63	
4	30	ta	11	48		33	37	
4	31		11	49		36	40	
4	32	ta	12	45	en	37	41	
4	33		12	47	en	39	43	
4	34		12	48	en	40	55	
4	54		13	45	en	40	60	
5	22		13	46	en	40	63	
5	23		13	47	en	43	60	
5	32		13	48	en	43	61	
5	33	ta	14	44	en	43	62	
5	34		14	45	en	43	63	
5	37		14	61	en	44	59	
5	40		15	21		44	60	
5	53		15	43	en	44	61	
5	54		15	44	en	45	49	
5	55		15	45	en	45	55	
6	21		16	21		45	59	
6	22		16	42	en	45	61	
6	23		16	43	en	46	58	
6	25	ta	16	44	en	46	59	
6	27	ta	17	21		46	60	
6	52		17	22		46	61	
6	53		17	40	en	47	58	
6	54		17	41	en	47	59	

Continued on next page...

TABLE A – continued from previous page.

**Res 1**	**Res 2**	**Class**	**Res 1**	**Res 2**	**Class**	**Res 1**	**Res 2**	**Class**
6	55		17	42	en	49	53	
7	11		17	43	en	49	55	
7	21		17	45	en	49	59	
7	23		17	55	en	53	59	
7	49		18	22		54	59	
7	50		19	37	en	55	59	
7	51		19	41	en	55	60	
7	52		22	37	en	56	60	
7	53		22	40	en, fe	57	63	

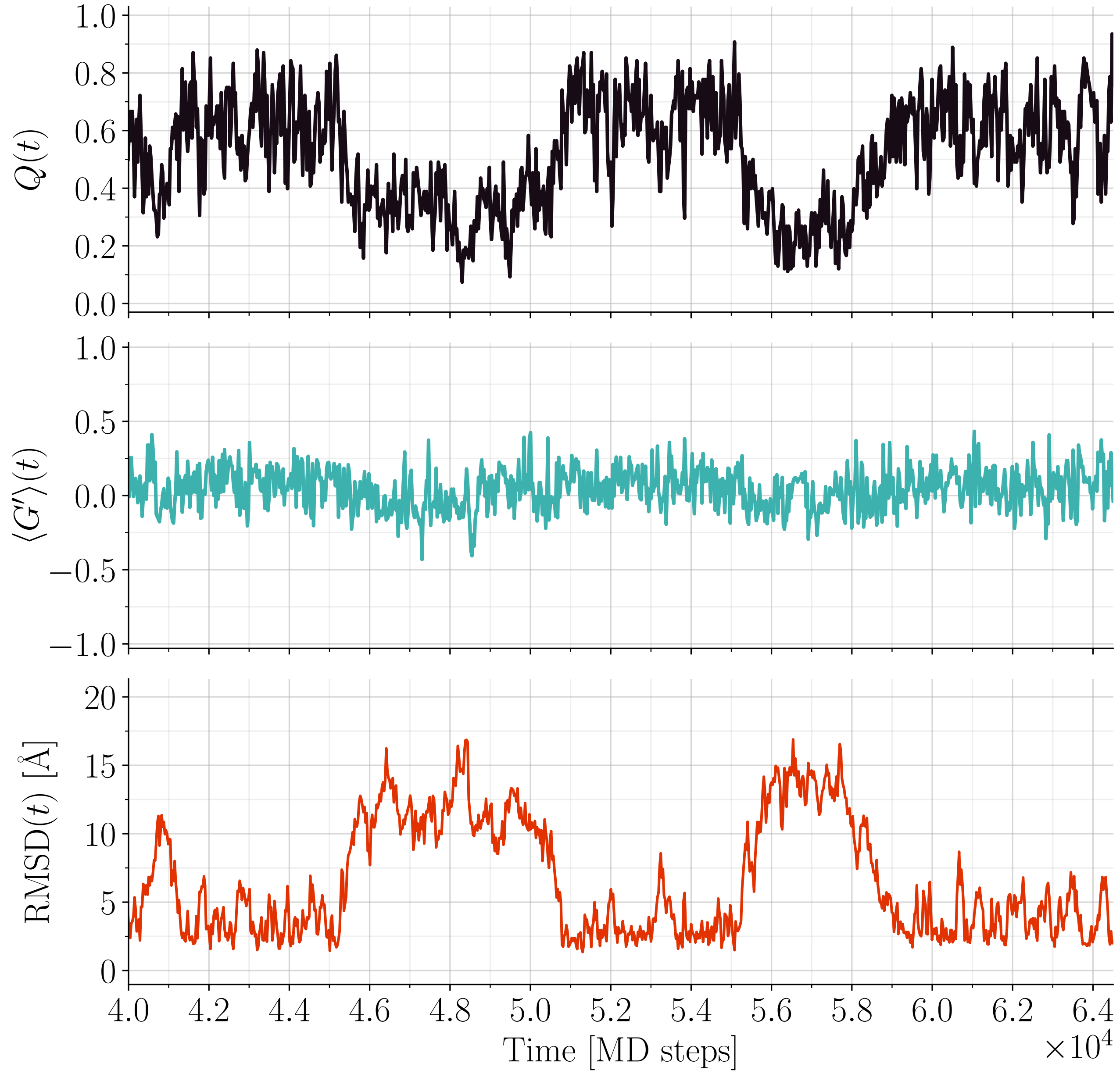

FIG. A. **Time series from equilibrium simulations for the non-entangled SH3 domain at $T = T_f$.** Upper panel: fraction of native contacts Q. Middle panel: entanglement indicator $\langle G' \rangle$. Lower panel: RMSD with the native structure.

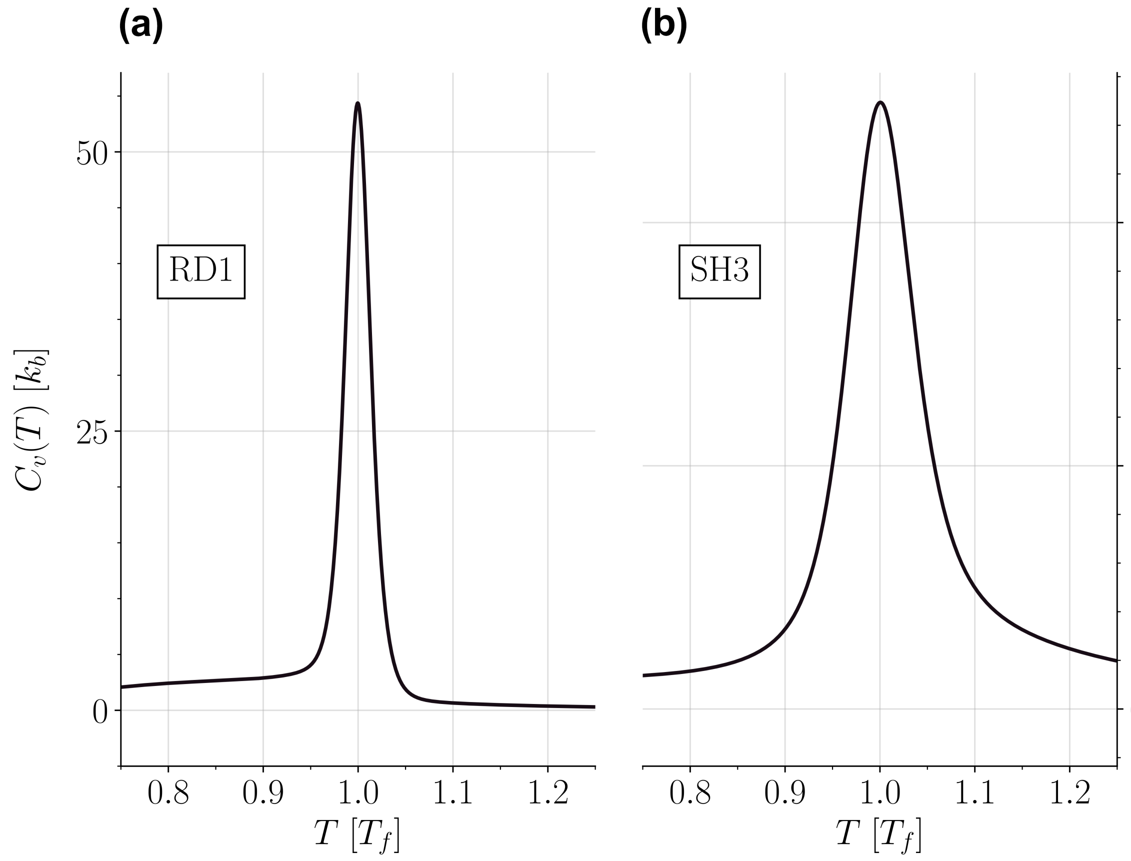

FIG. B. **Specific heat profiles for the entangled RD1 protein and the non-entangled SH3 domain.** Left panel: RD1 protein. Right panel: SH3 domain. Temperature is rescaled in both cases by the folding temperatures T_f identified at the specific heat peak, which are $T_f^{\mathrm{RD1}} = 1.149 \pm 0.005$ and $T_f^{\mathrm{SH3}} = 1.012 \pm 0.001$.

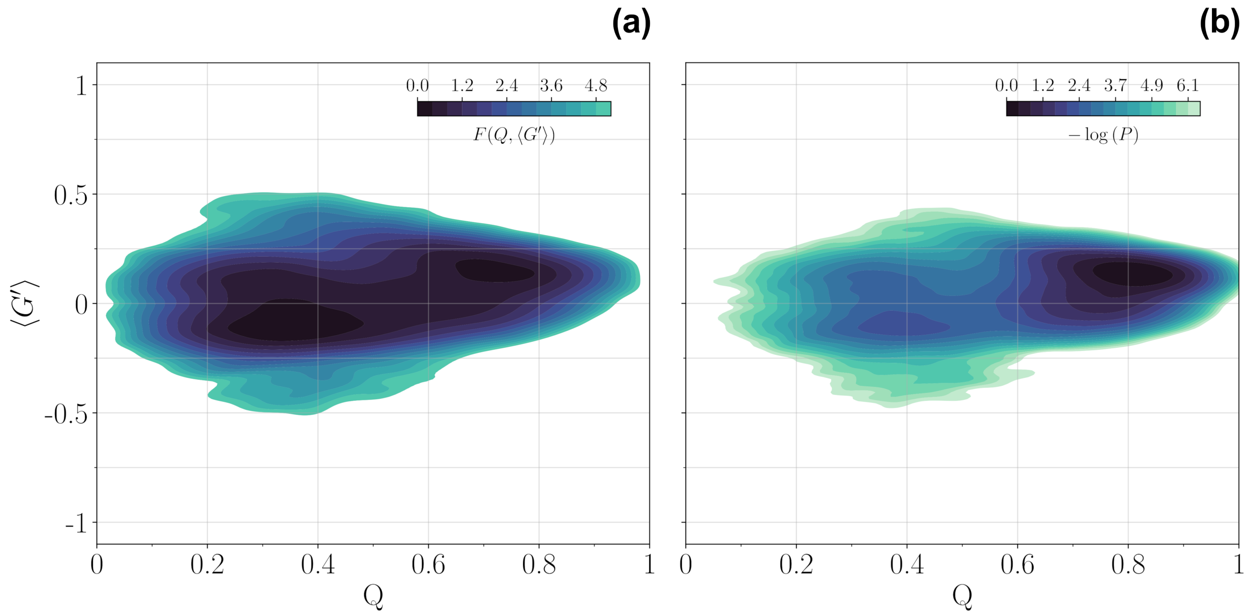

FIG. C. **Log-scale histogram contour plots in the $(Q, \langle G' \rangle)$ plane for the non-entangled SH3 domain.** Histogram negative log-counts are smoothed using KDE (see the Ensemble definition and pathway classification subsection) and shifted in order for their minimum to be 0. Contour levels and the colour scale are the same in both panels and are the same as in Fig 3 and in Fig 5 in the main text. (a): Data collected from 8 long equilibrium trajectories at $T = T_f$; the contour plot represents a dimensionless free energy surface (b): Data collected from 100 trajectories refolding from an initial unfolded configuration at $T = 0.9T_f$; the unfolded state is populated transiently and the contour plot does *not* represent a free energy surface.

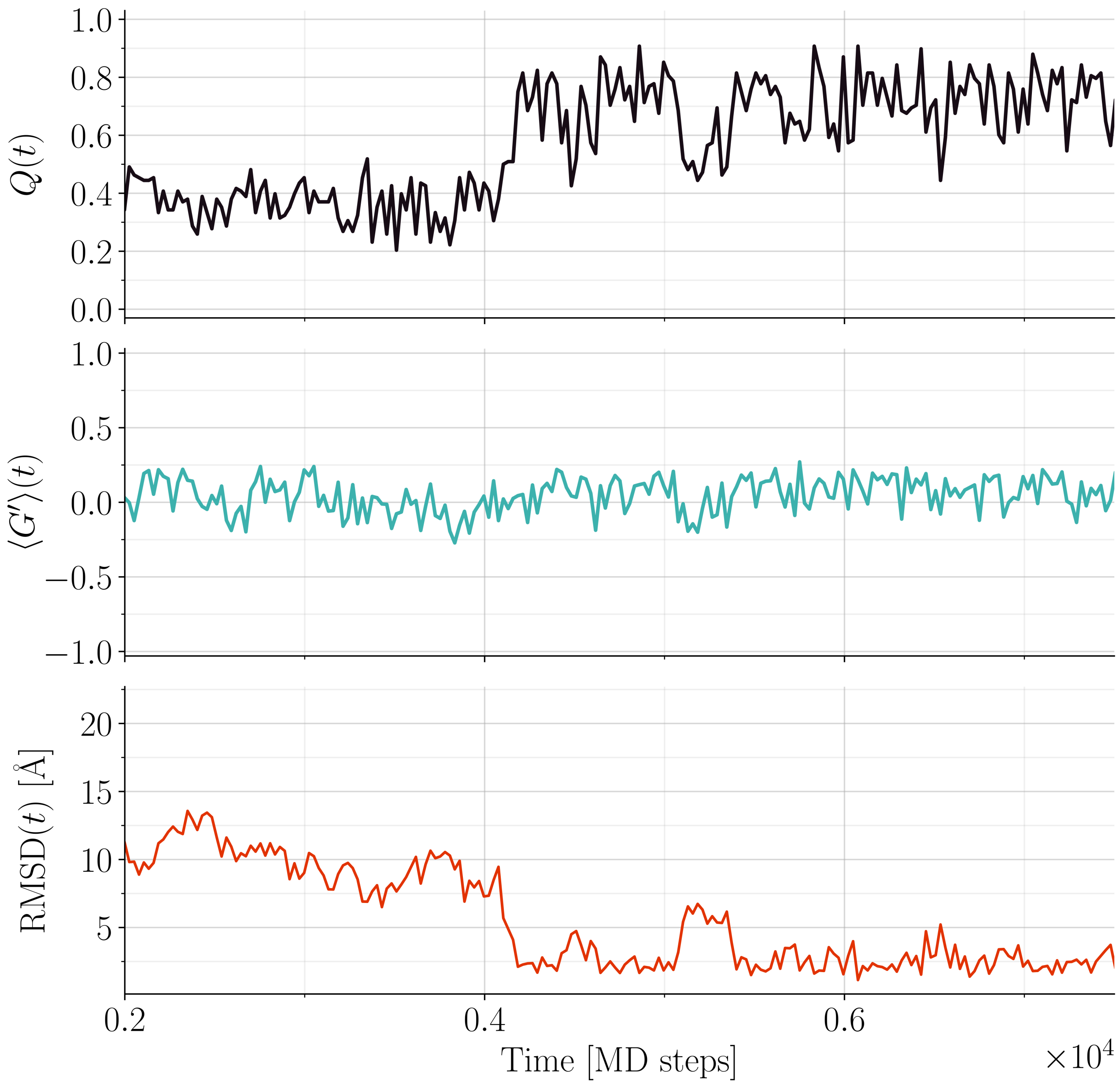

FIG. D. **Time series from refolding simulations for the non-entangled SH3 domain at $T = 0.9T_f$.** Upper panel: fraction of native contacts Q. Middle panel: entanglement indicator $\langle G' \rangle$. Lower panel: RMSD with the native structure.

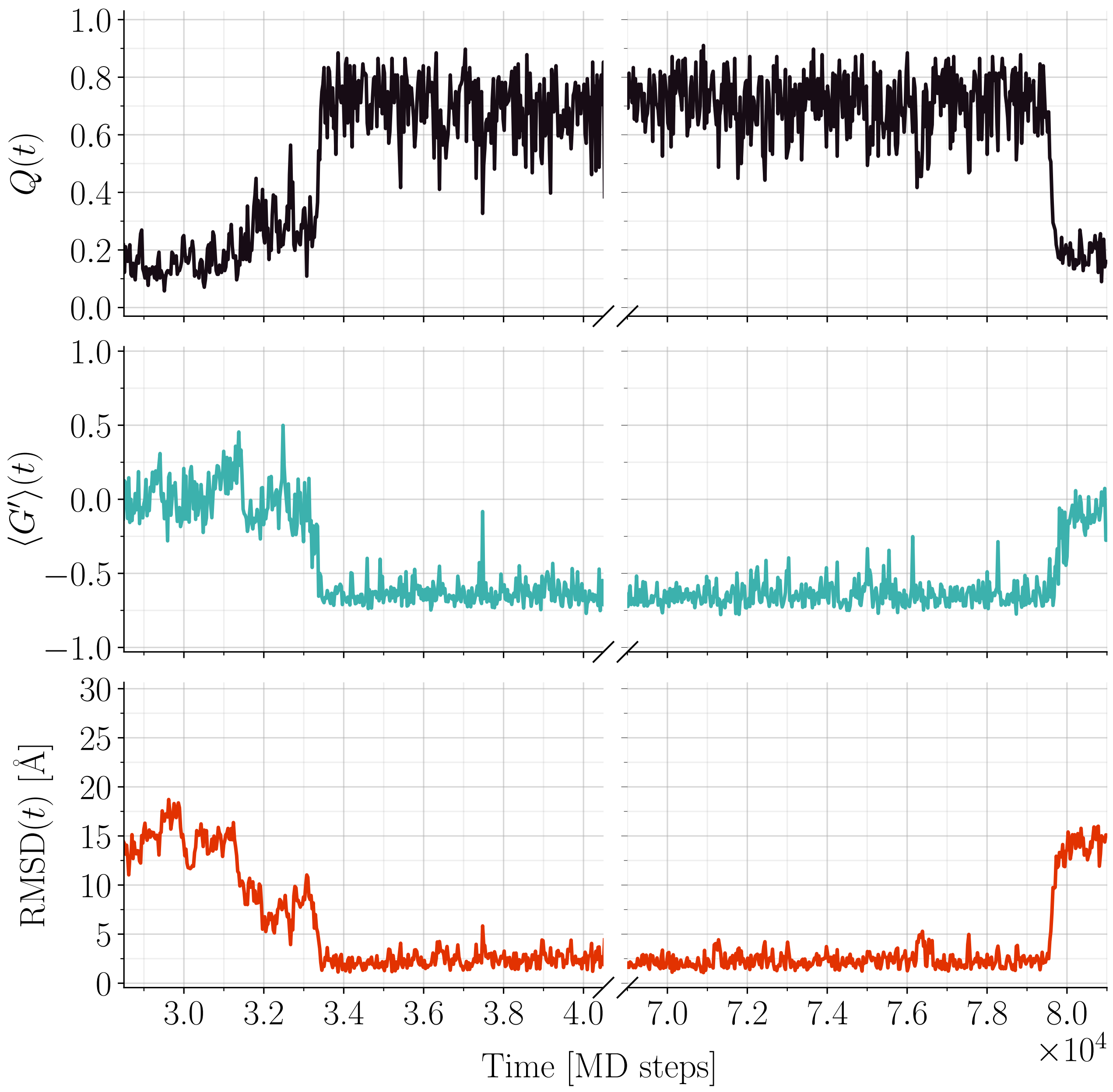

FIG. E. **Time series from equilibrium simulations for the entangled RD1 protein at $T = T_f$.** Upper panel: fraction of native contacts Q. Middle panel: entanglement indicator $\langle G' \rangle$. Lower panel: RMSD with the native structure.

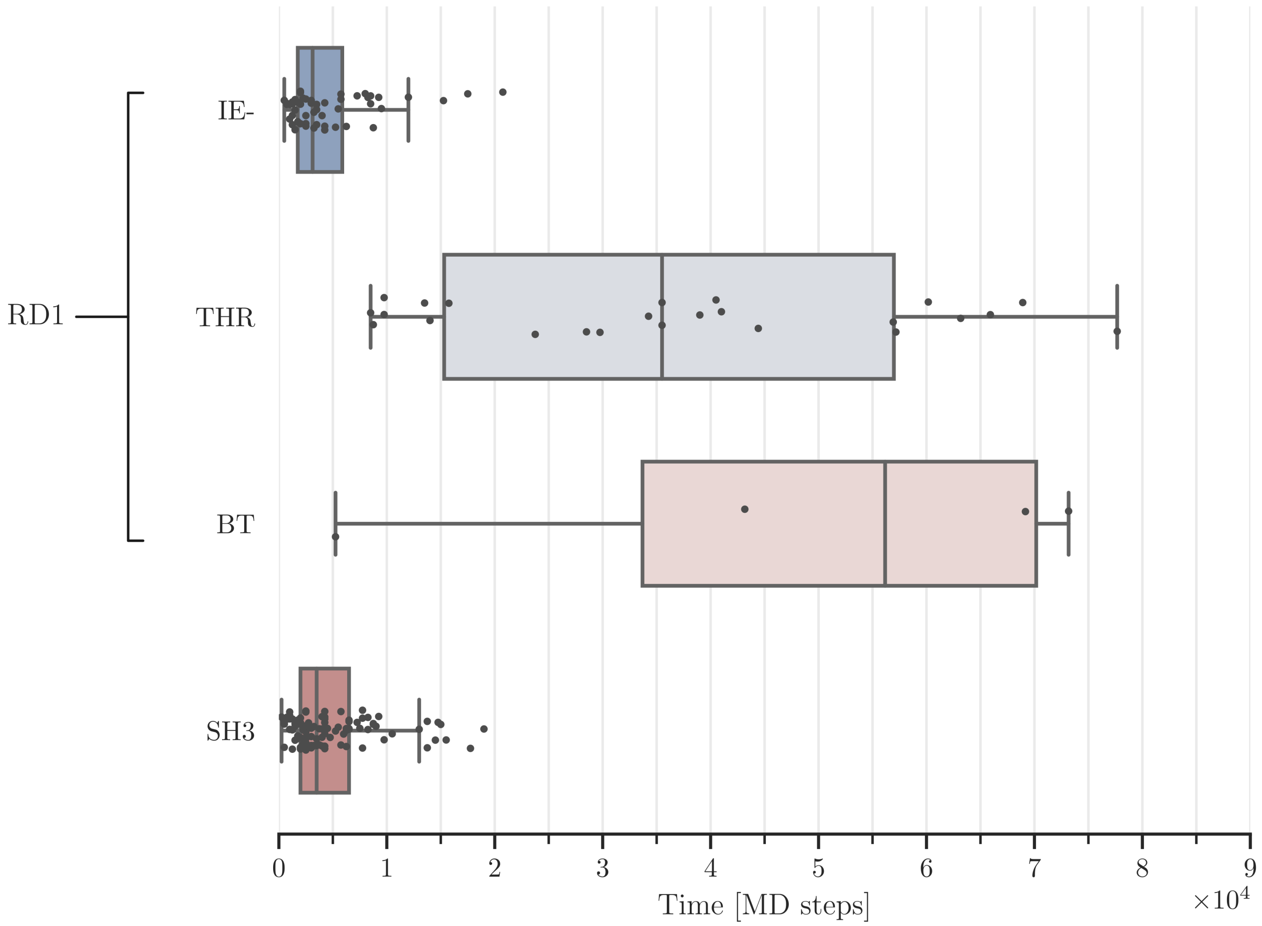

FIG. F. **Distributions of folding times for the different types of refolding trajectories for the entangled RD1 protein and for the non-entangled SH3 domain.** The 81 trajectories that achieved successful refolding for the RD1 protein are partitioned into the "fast" channel IE_- (52 trajectories going through the short-lived entangled intermediate IE_- that are never trapped in IT), the "threading" channel THR (25 trajectories that fold through a final IT→F transition) and the backtracking channel BT (4 trajectories that finally backtrack to the fast channel after trapping in IT). All the 100 refolding trajectories for the SH3 domain are grouped together. Boxplots show the median, the quartile and the first and ninth decile values. The folding time of a given trajectory is defined as the first time for which $Q \geq 0.75$ and $\langle G' \rangle \leq -0.5$ for RD1 and $Q \geq 0.7$ for SH3.

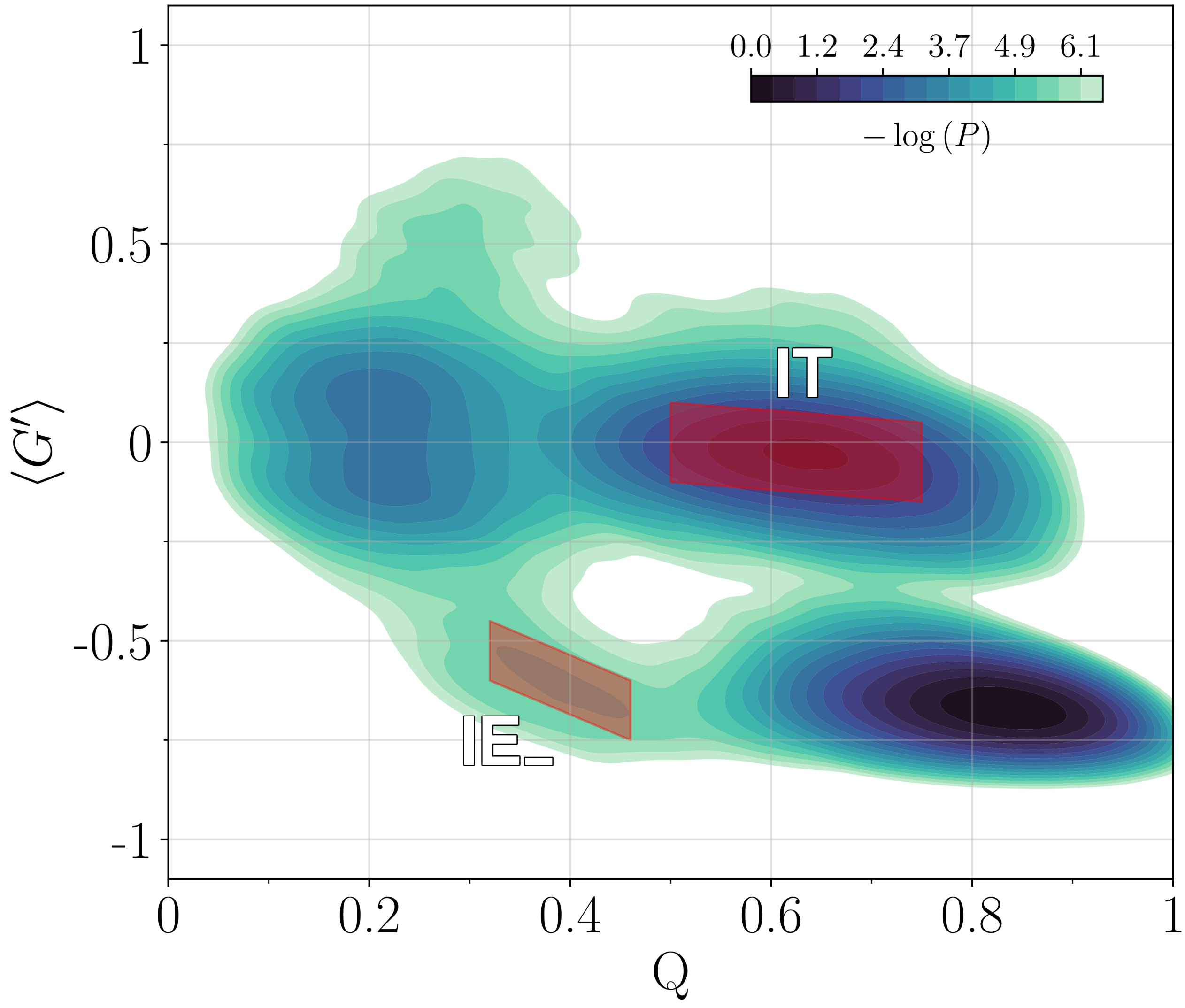

FIG. G. **Identification of the intermediate state ensembles for the entangled RD1 protein.** Log-scale histogram contour plot in the $(Q, \langle G' \rangle)$ plane from 100 refolding trajectories for the RD1 protein at $T = 0.9T_f$. Colored areas define configurations classified as part of one of the intermediate ensembles. The darker shaded area corresponds to the IT ensemble, while the lighter one to IE_- ensemble.

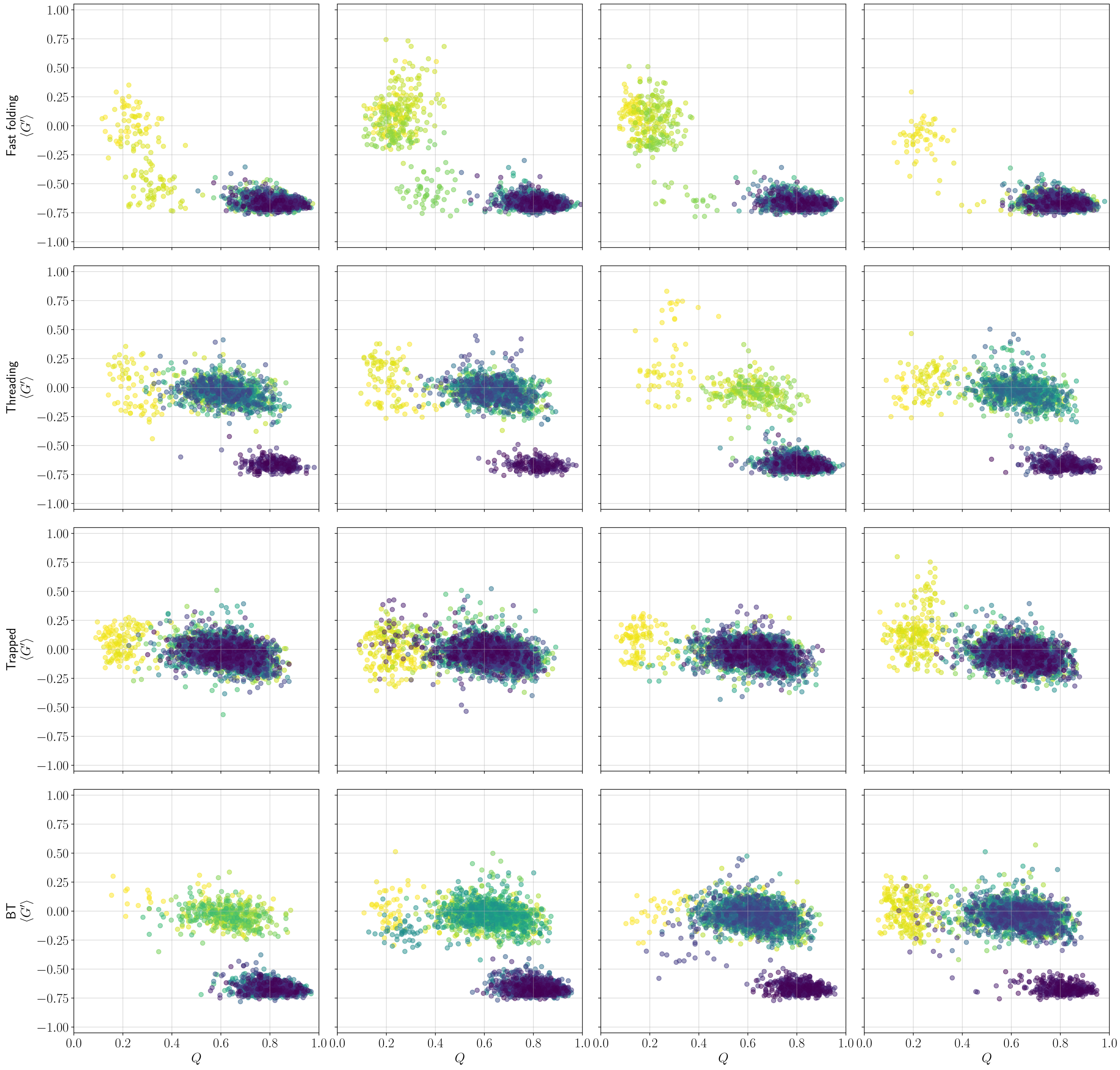

FIG. H. **Examples of refolding RD1 trajectories in the $(Q, \langle G' \rangle)$ for each pathway.** For each possible pathway, as defined in Material and Methods and in Table 1 of the main text, four example trajectories are shown in the $(Q, \langle G' \rangle)$ plane. Color code represents time, where brighter colors are related to early times and darker ones to later stages of the refolding trajectories.
