# Supplementary figures and images for: Folding kinetics of an entangled protein
Source: PLoS Comput Biol. 2023 Nov 13;19(11):e1011107. doi: 10.1371/journal.pcbi.1011107 (PMC10681328; doi:10.1371/journal.pcbi.1011107)

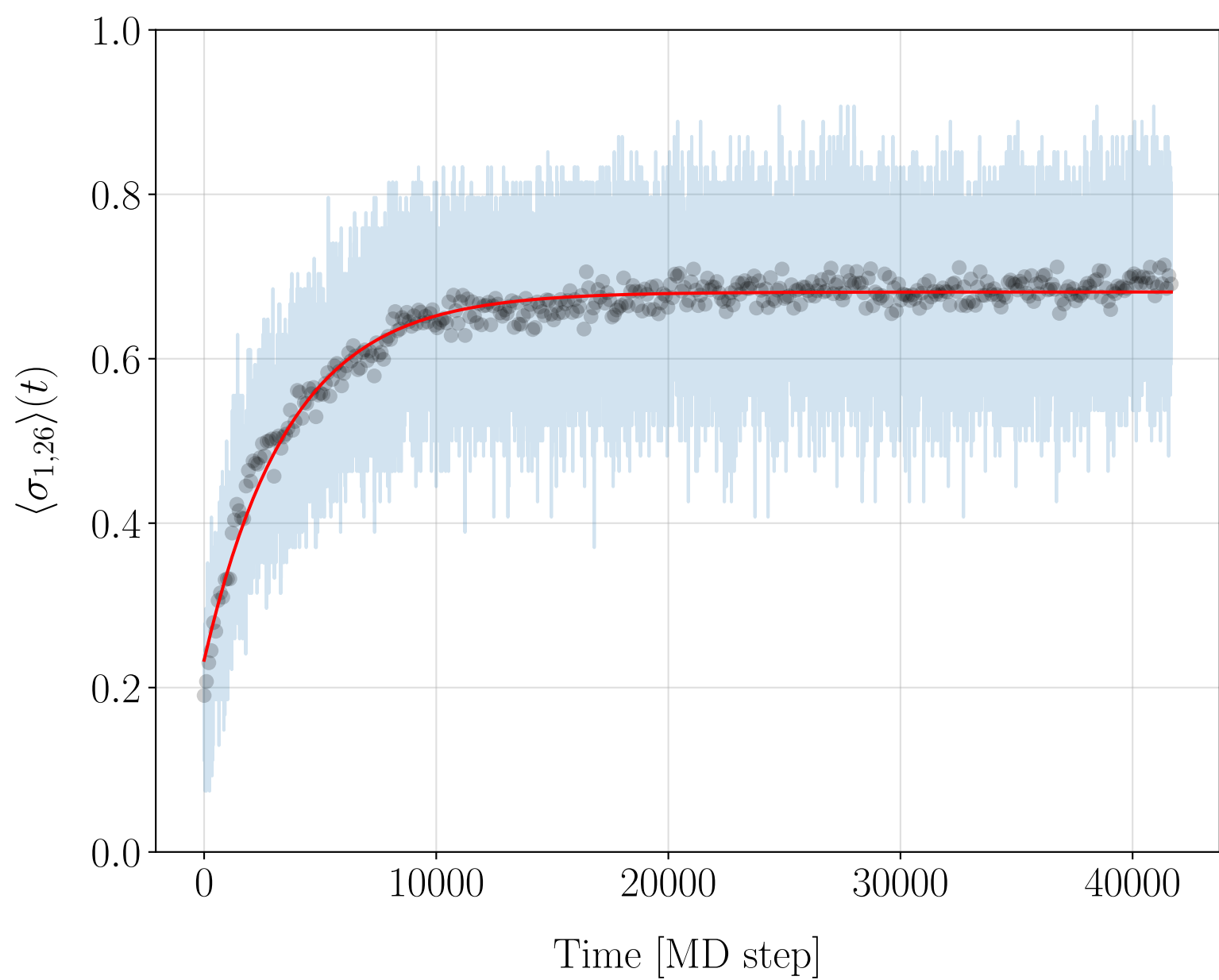

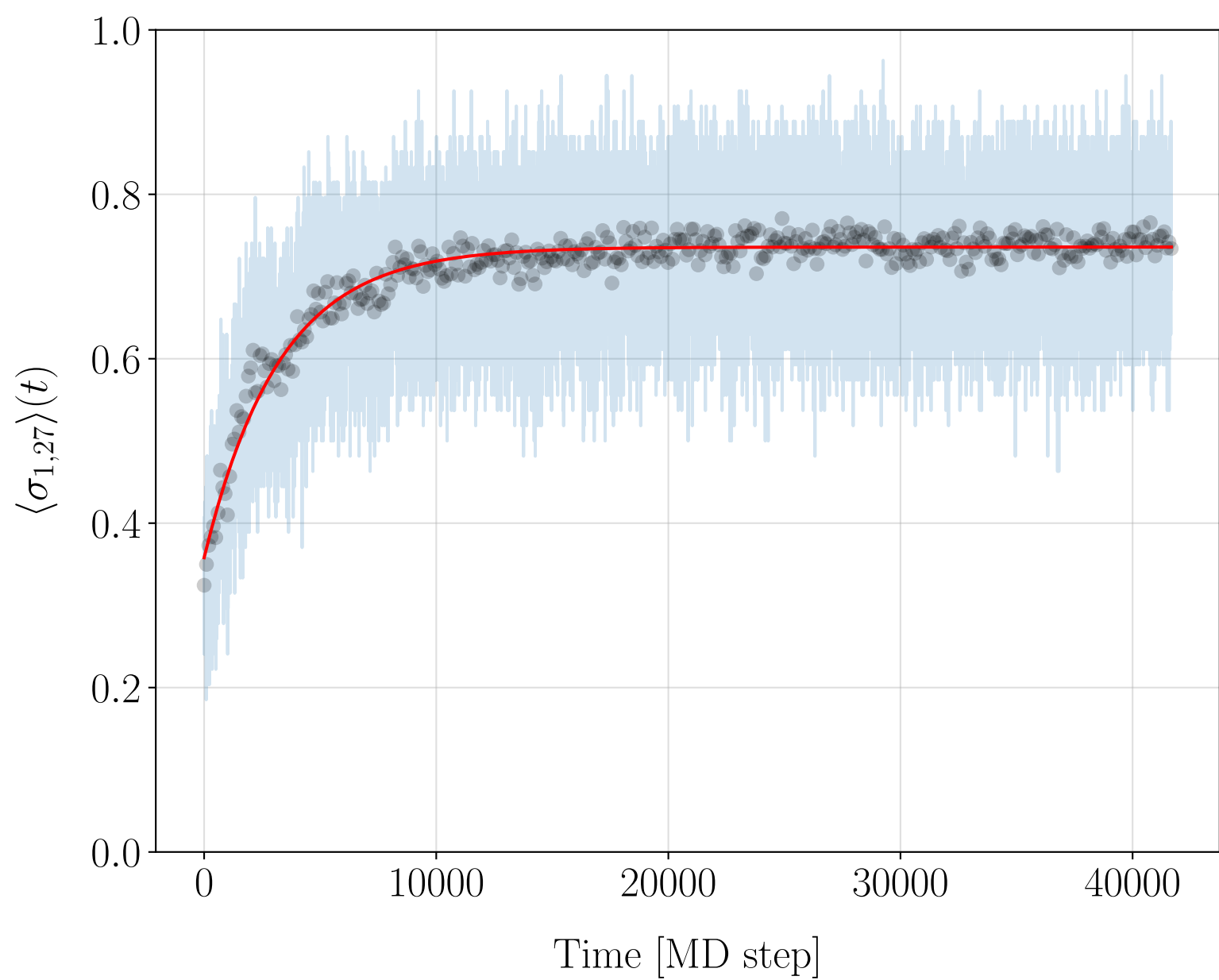

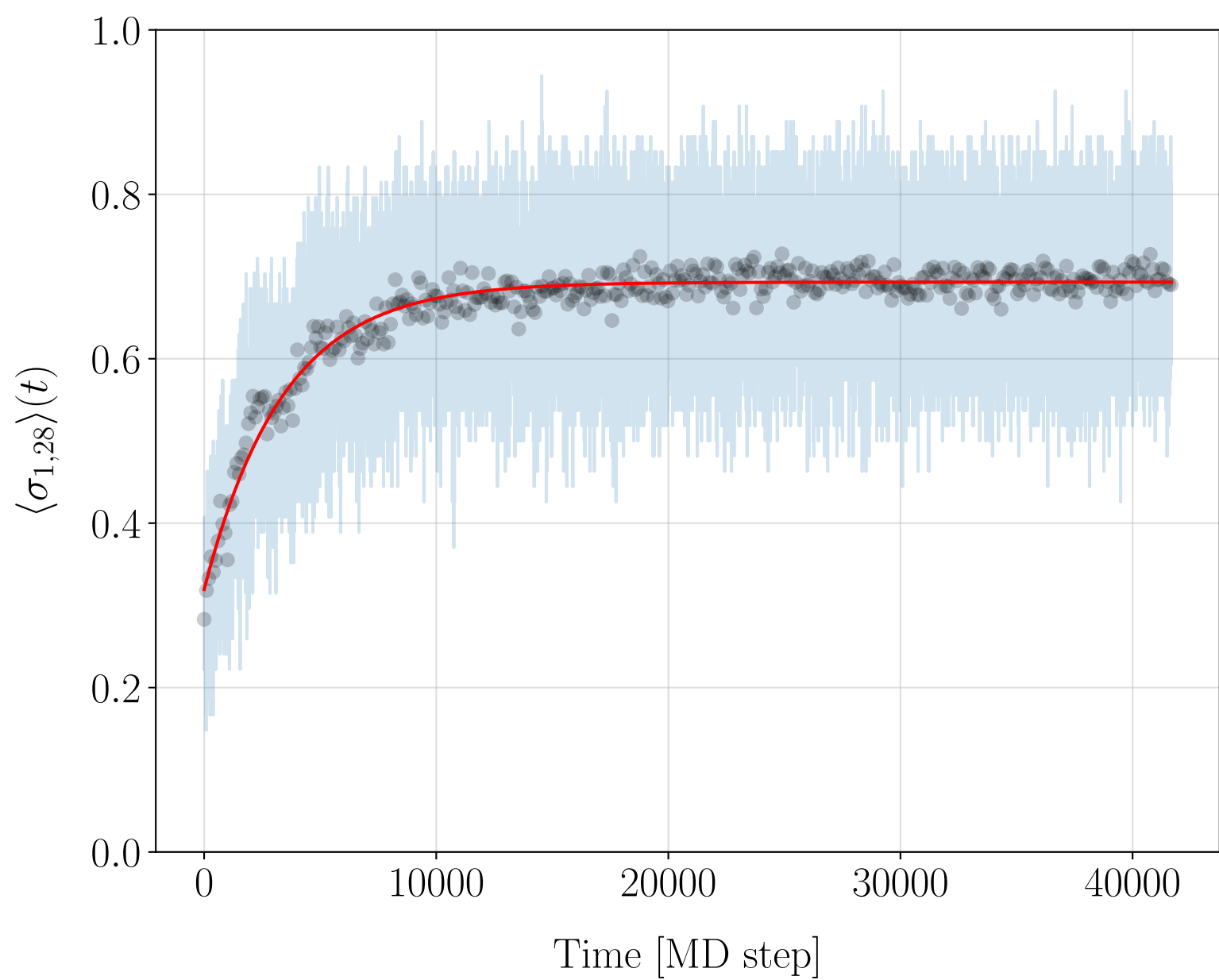

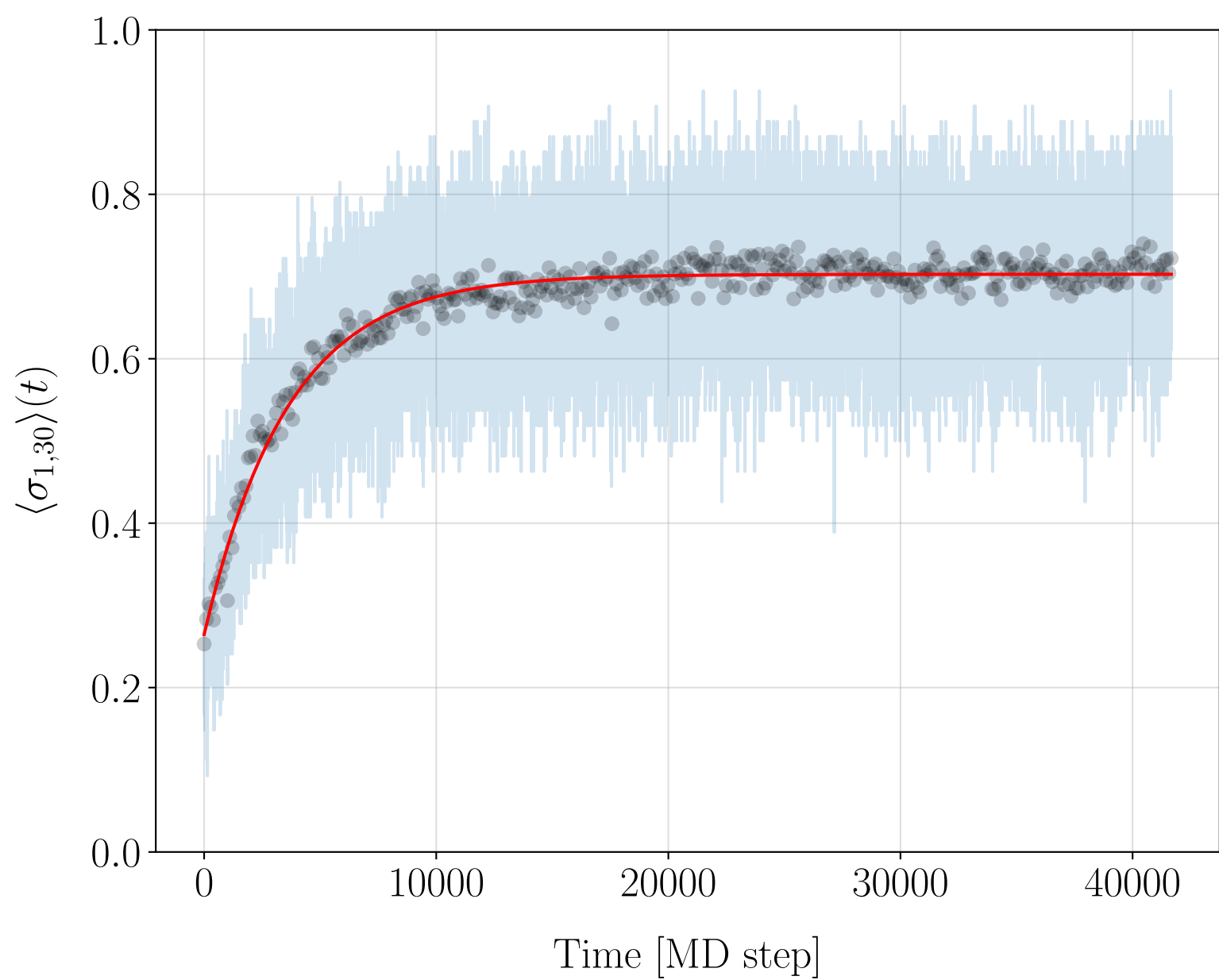

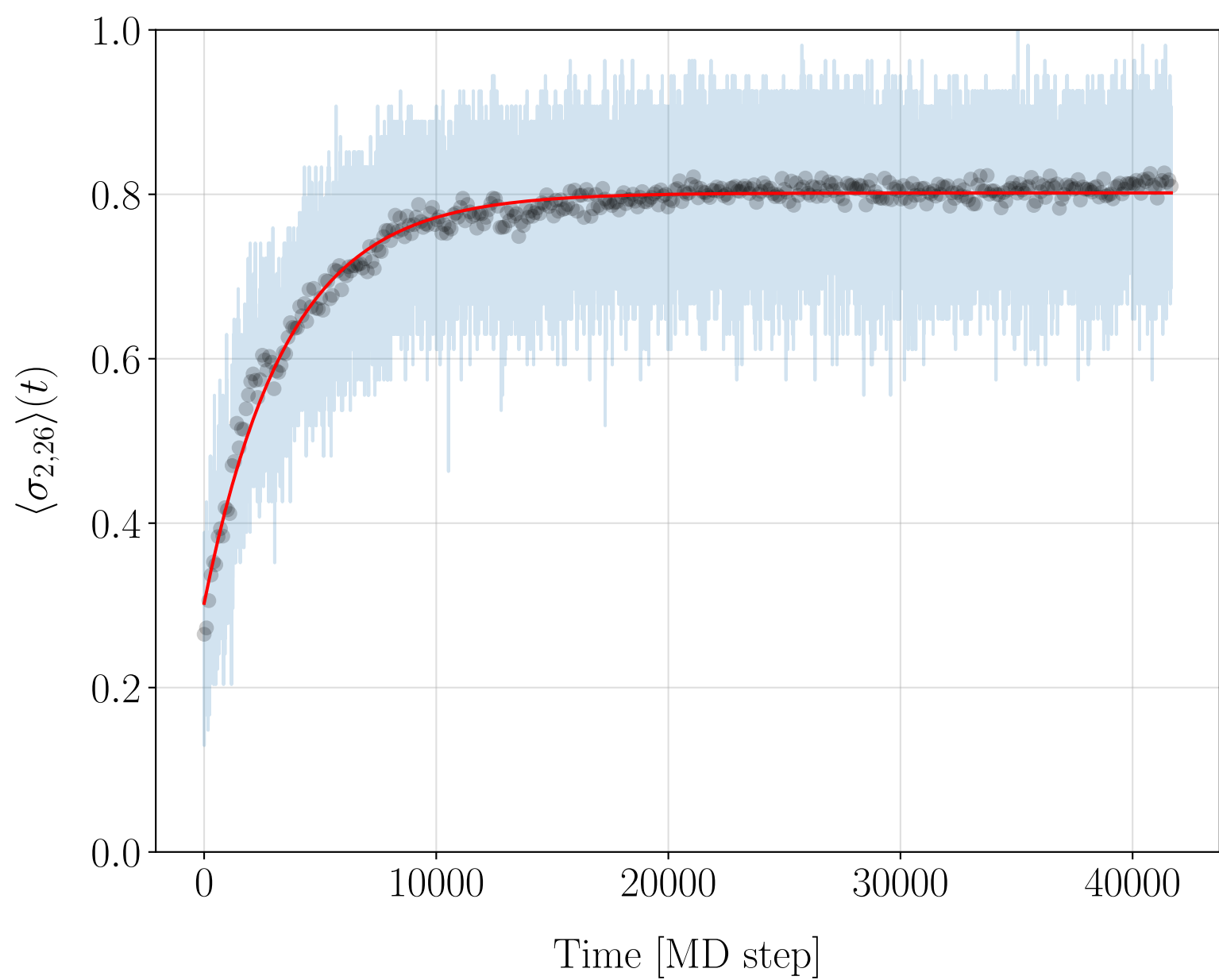

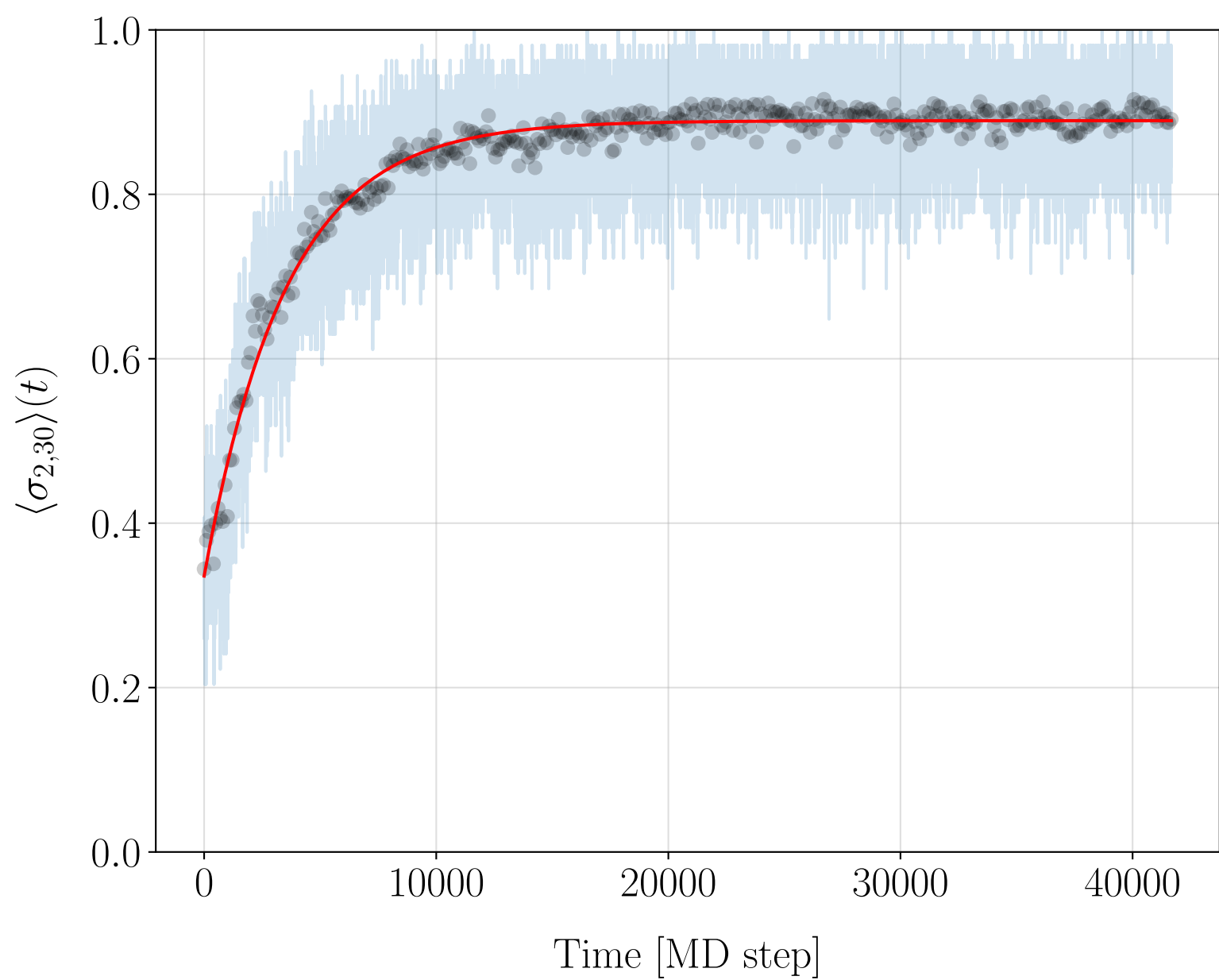

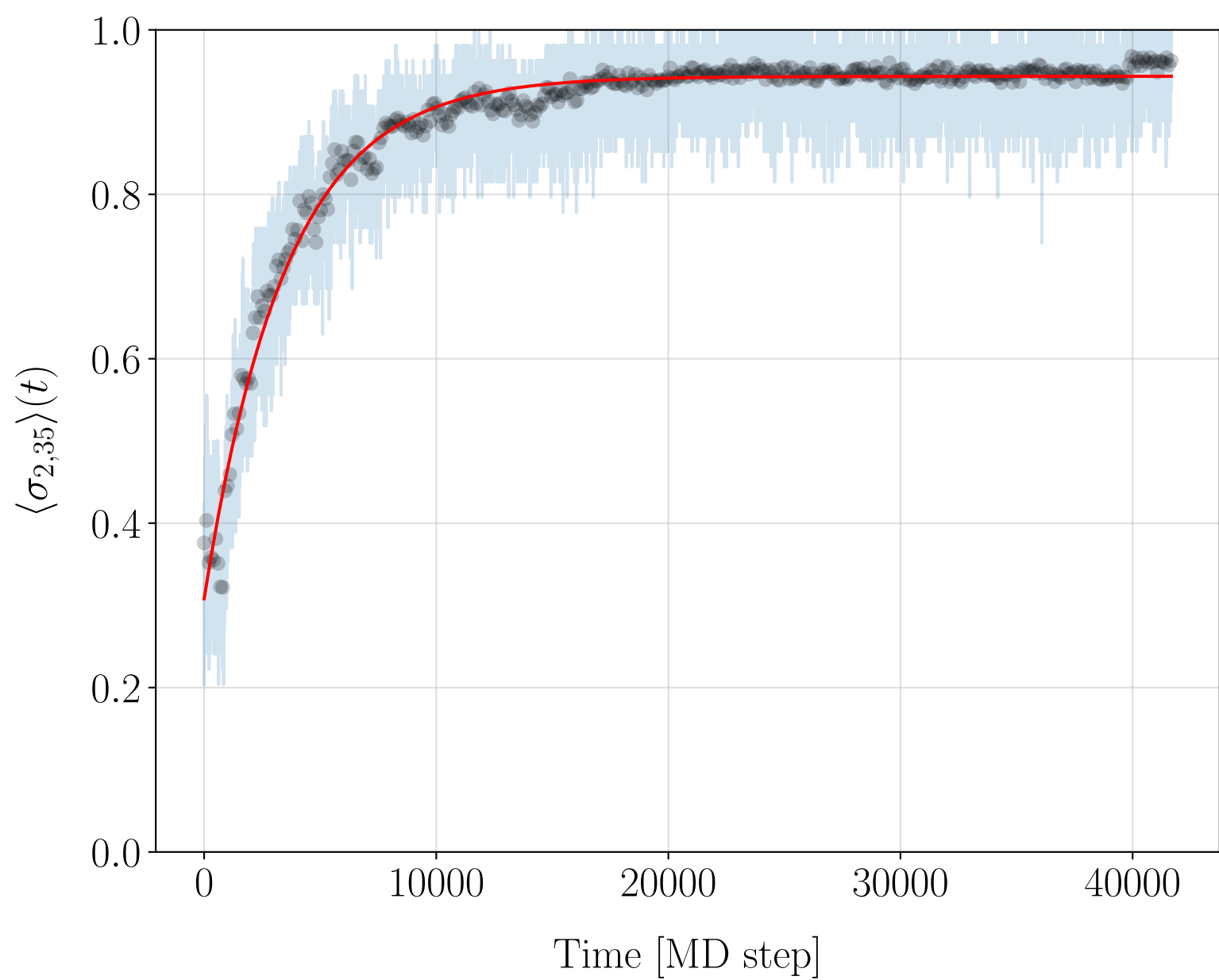

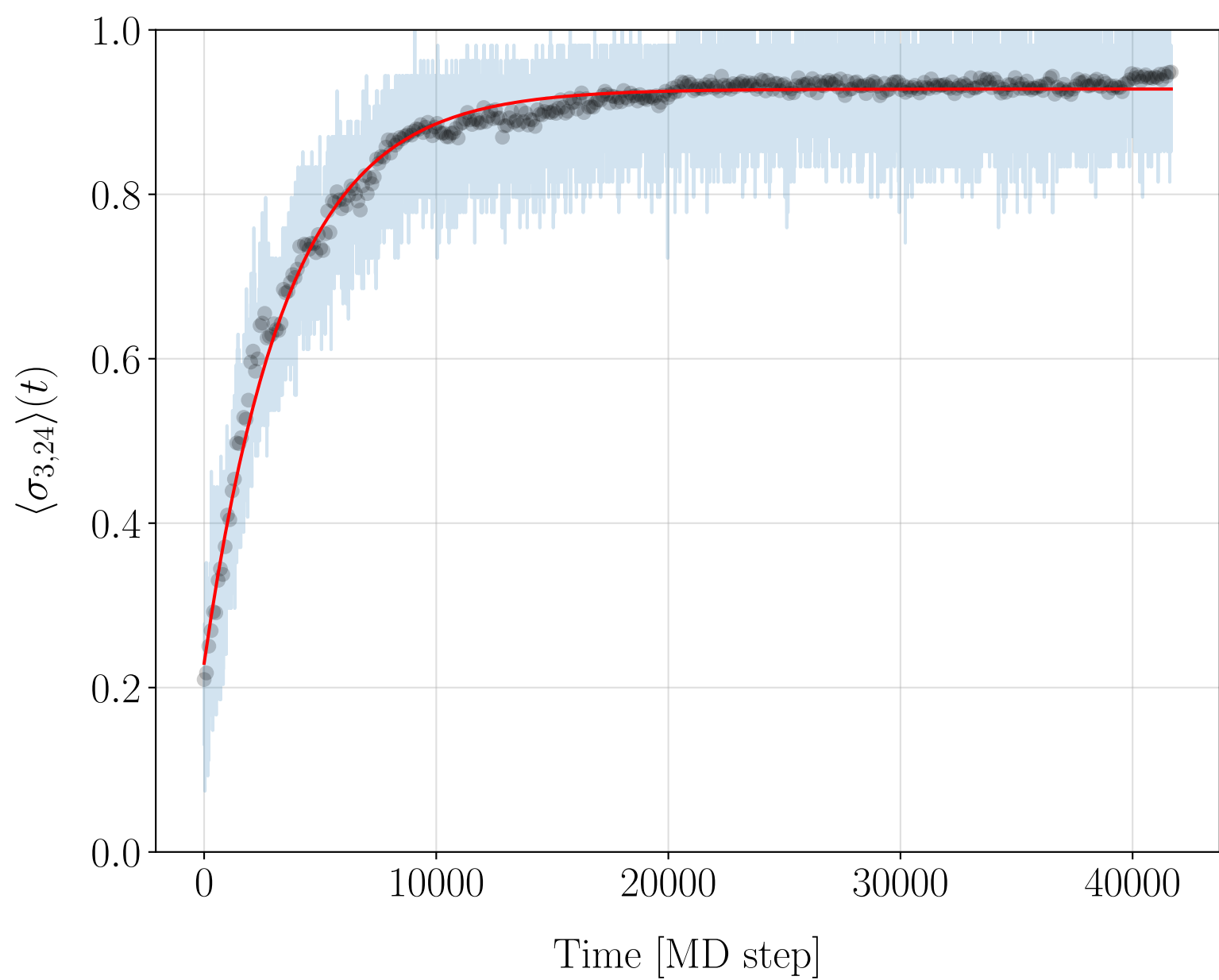

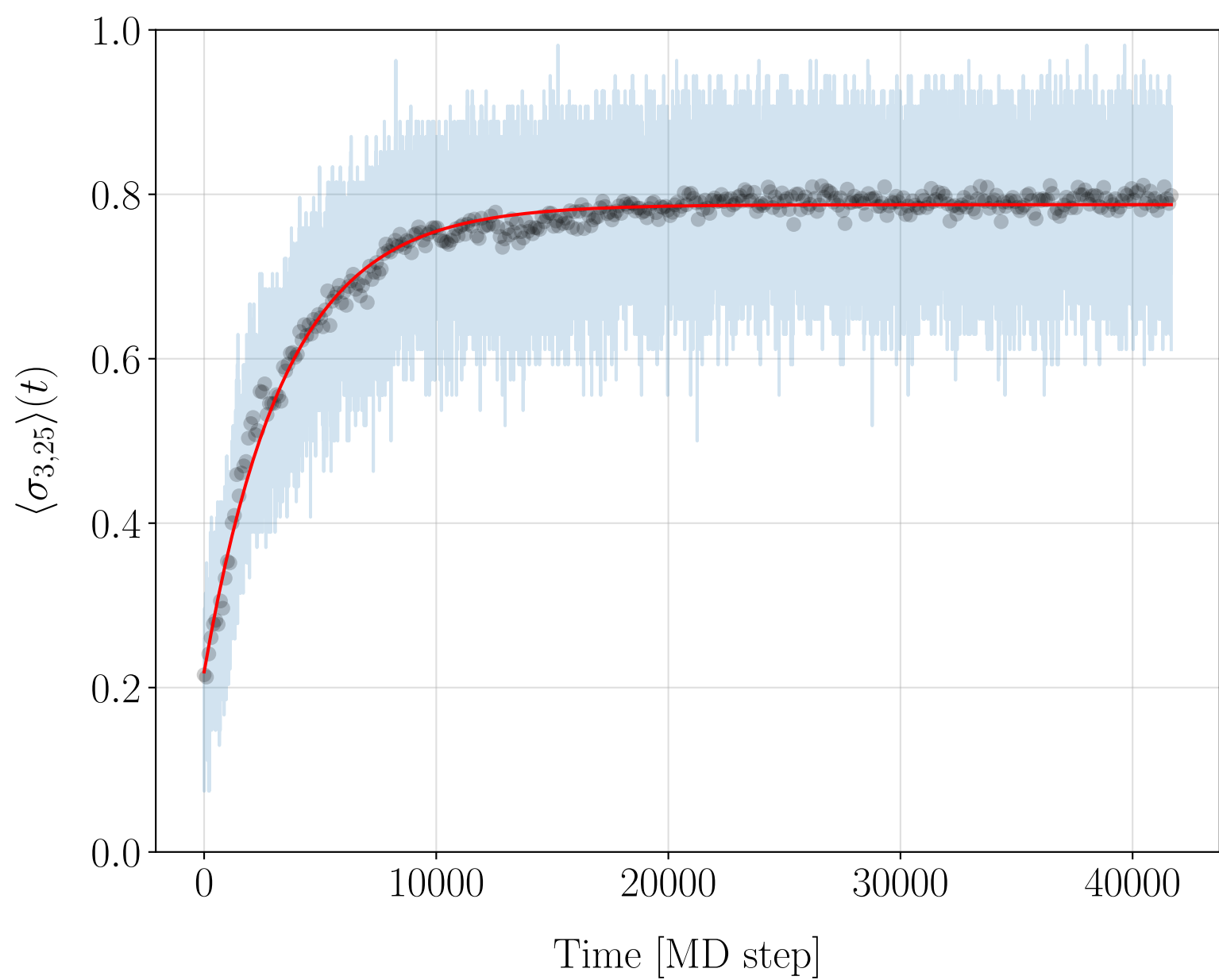

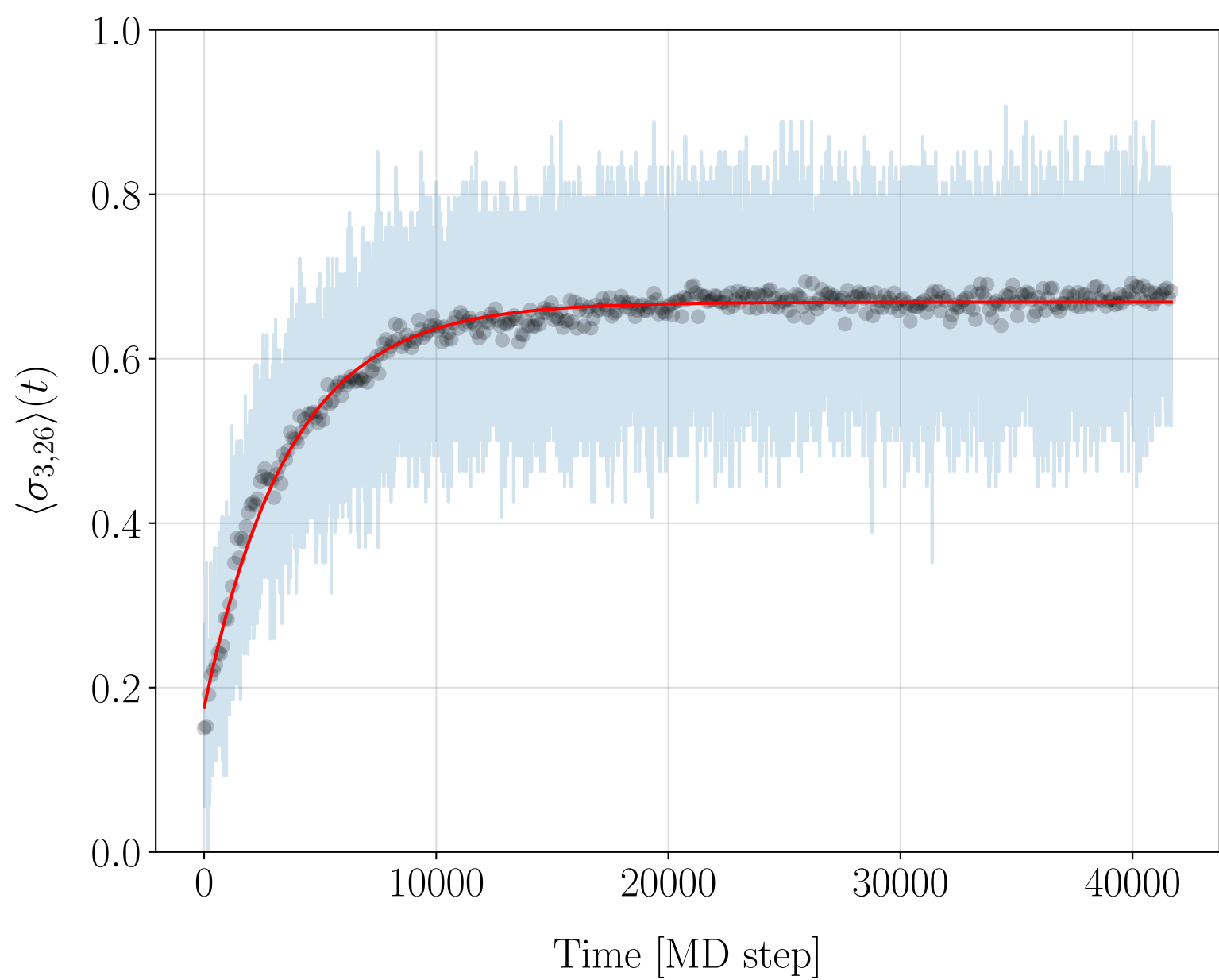

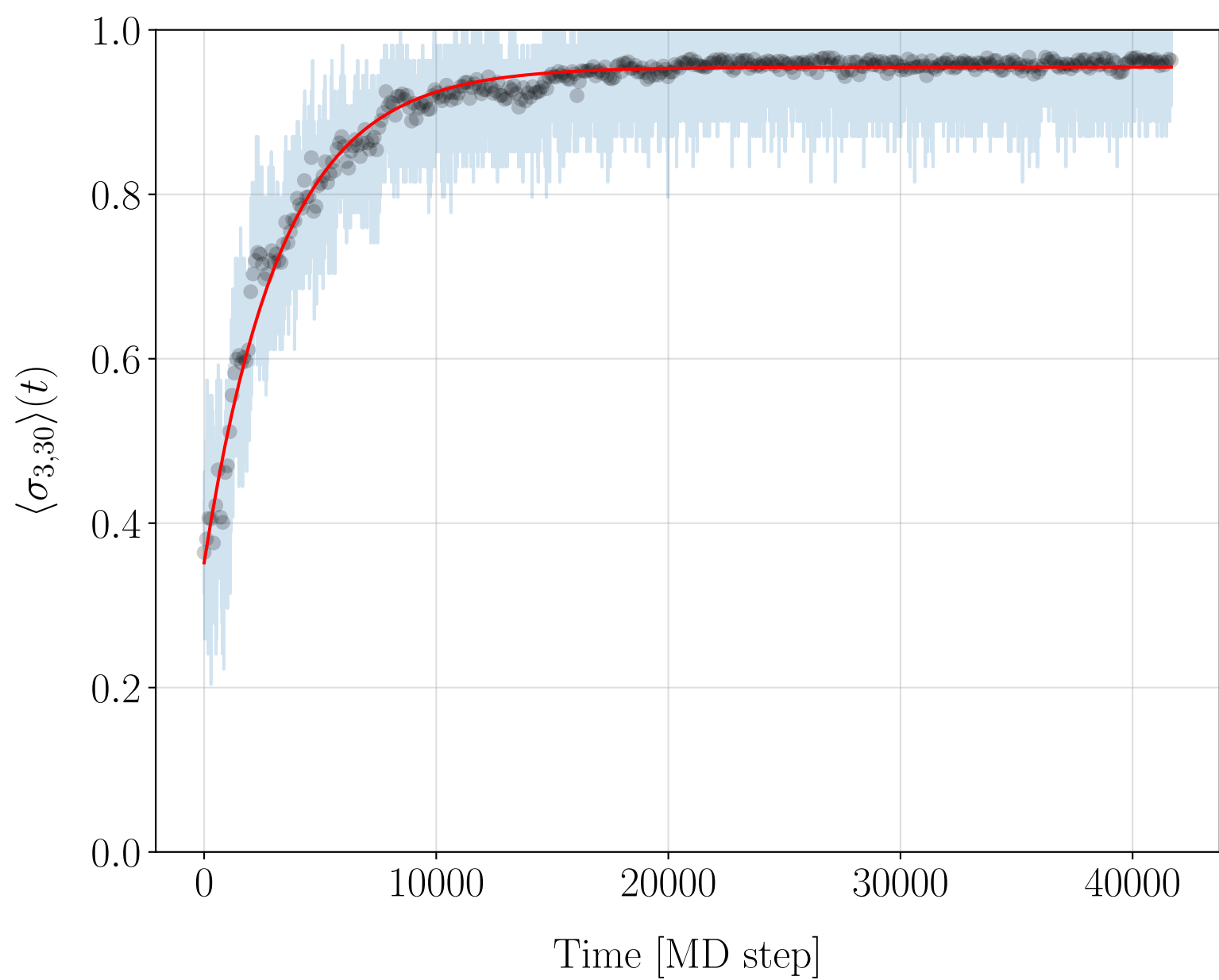

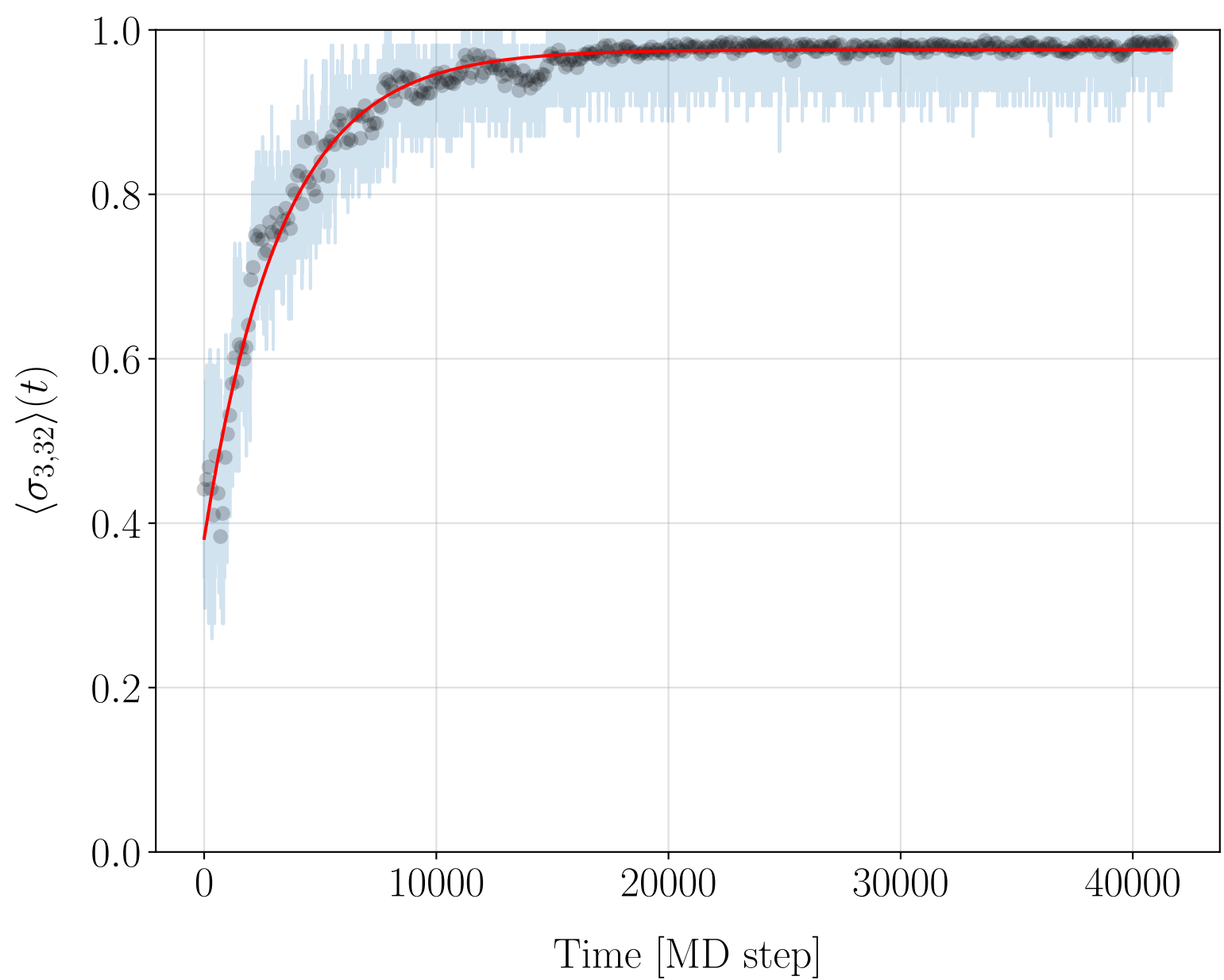

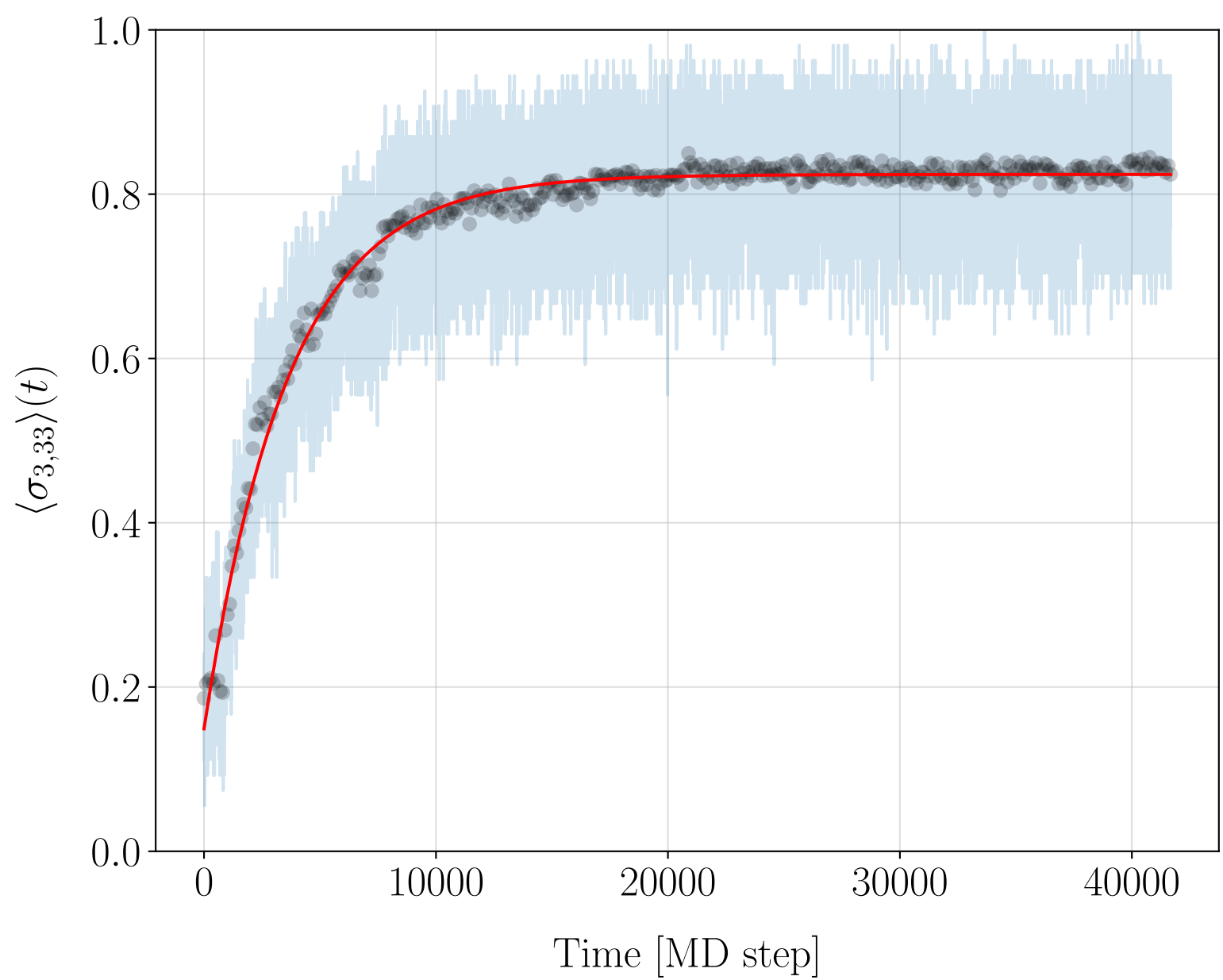

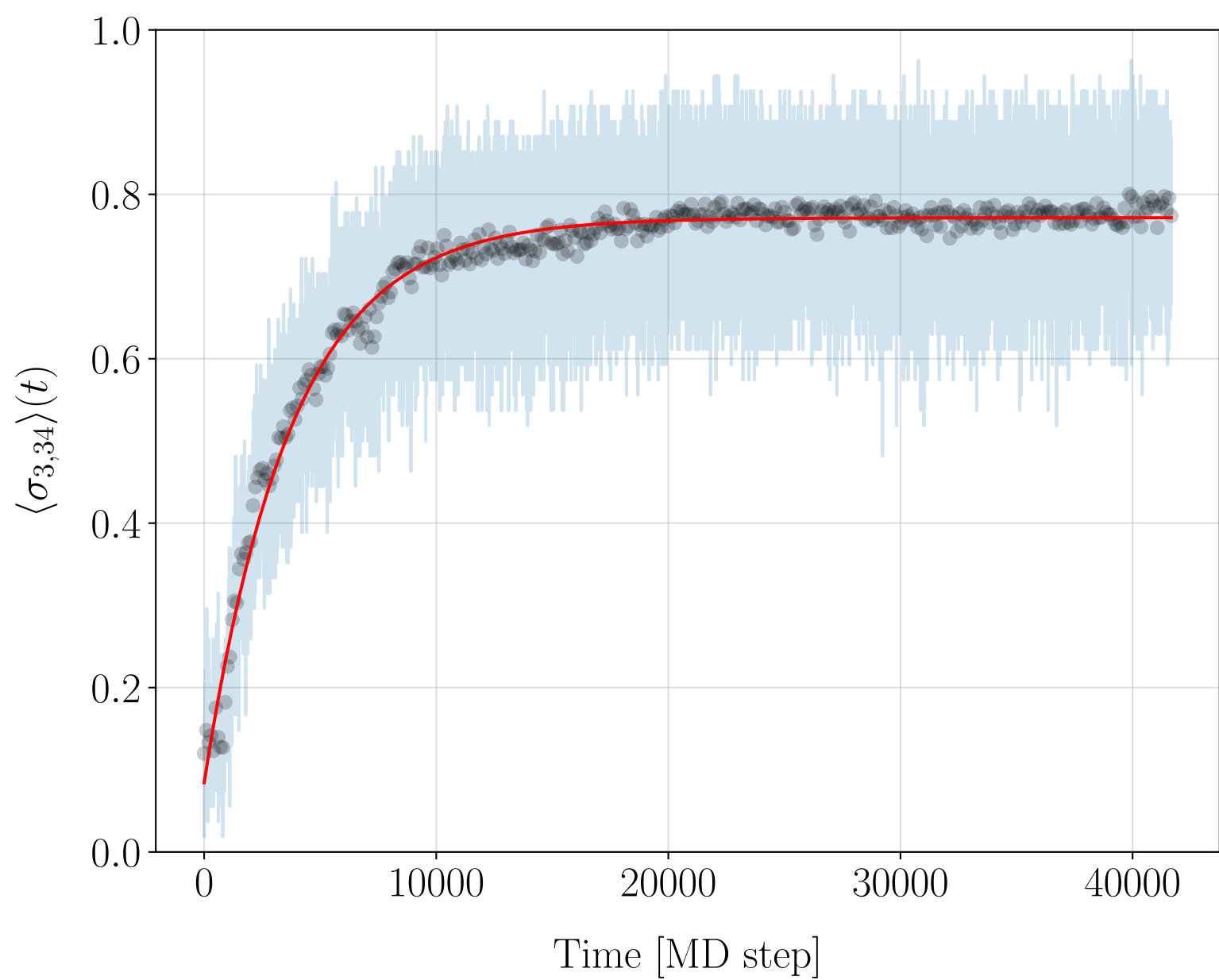

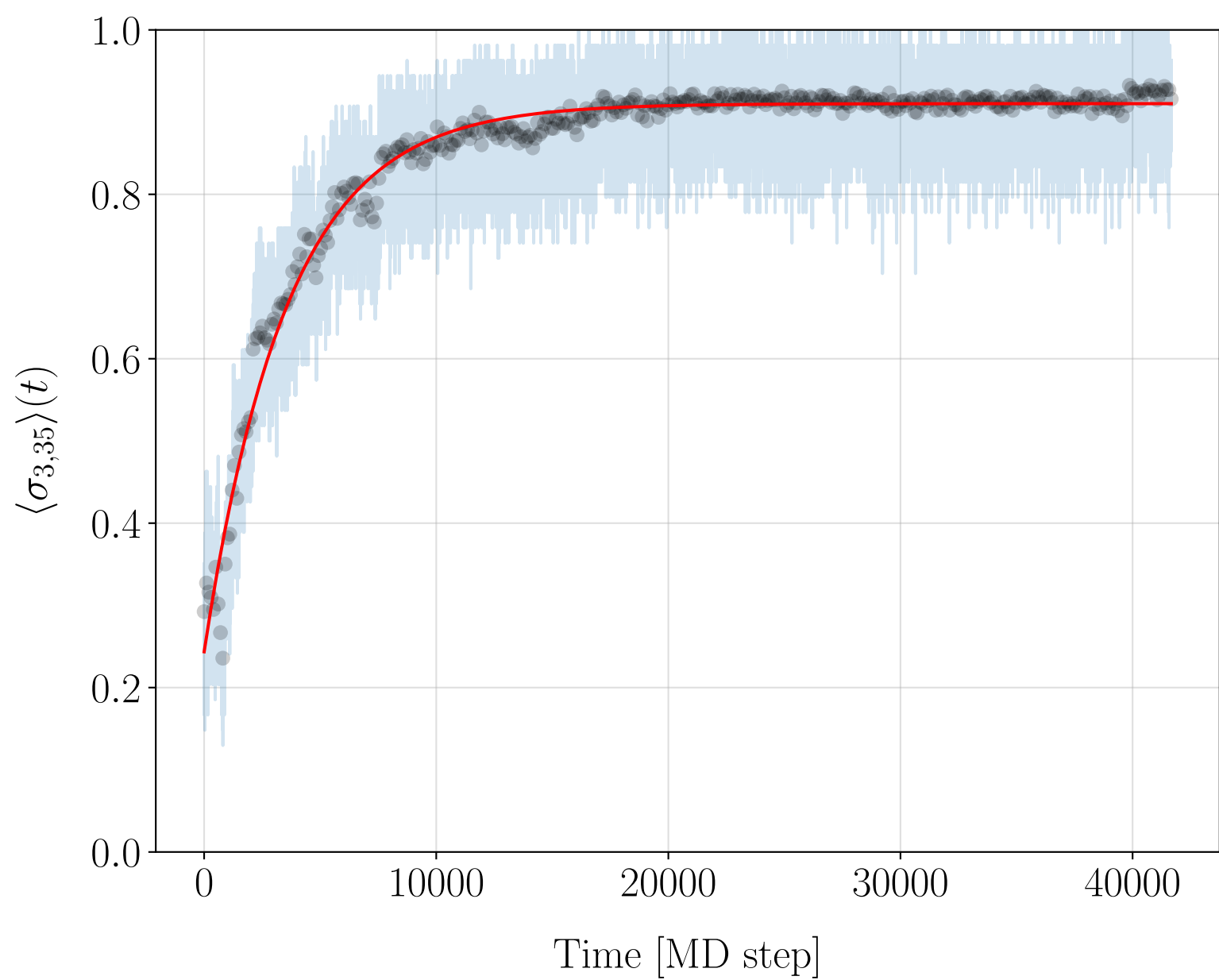

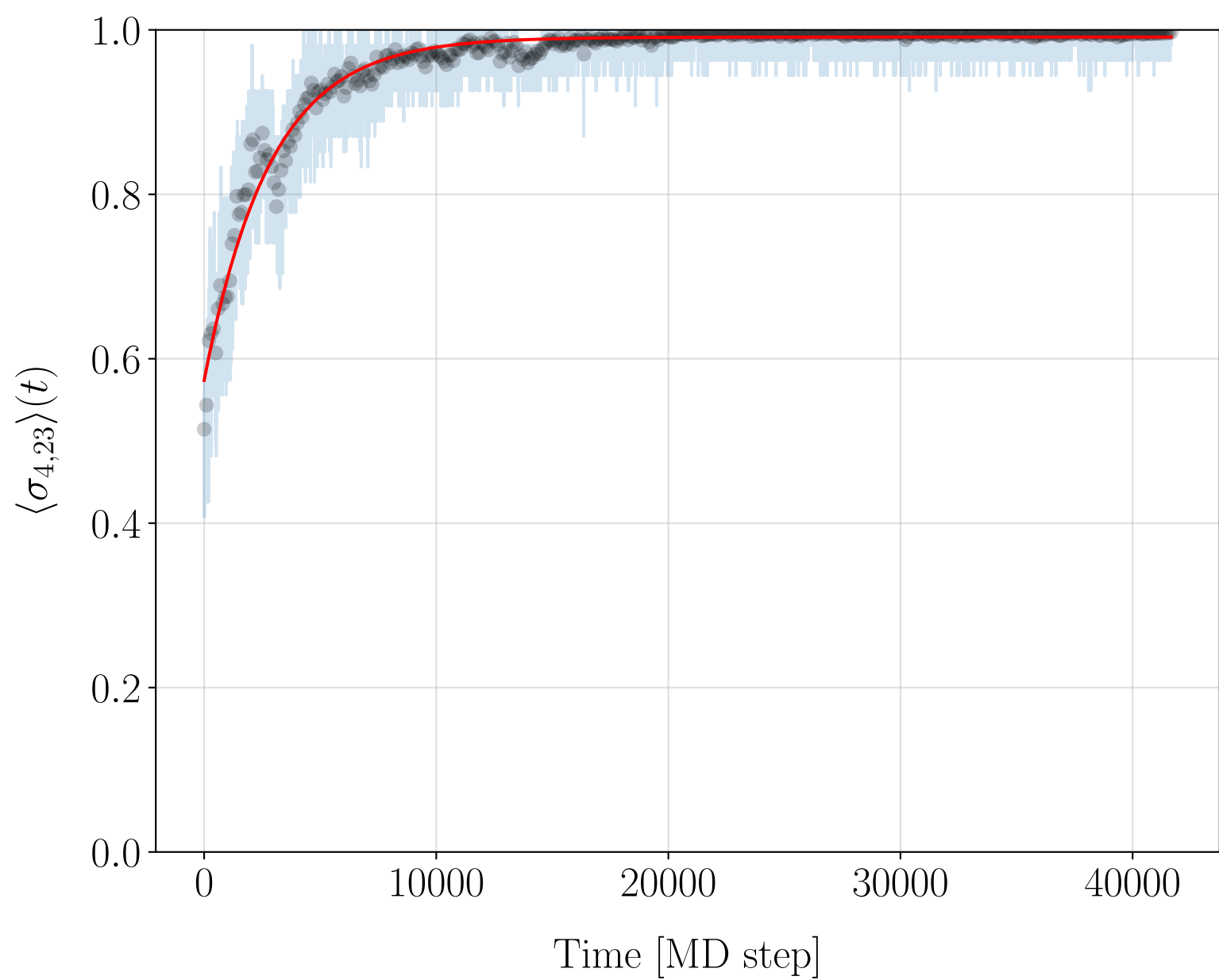

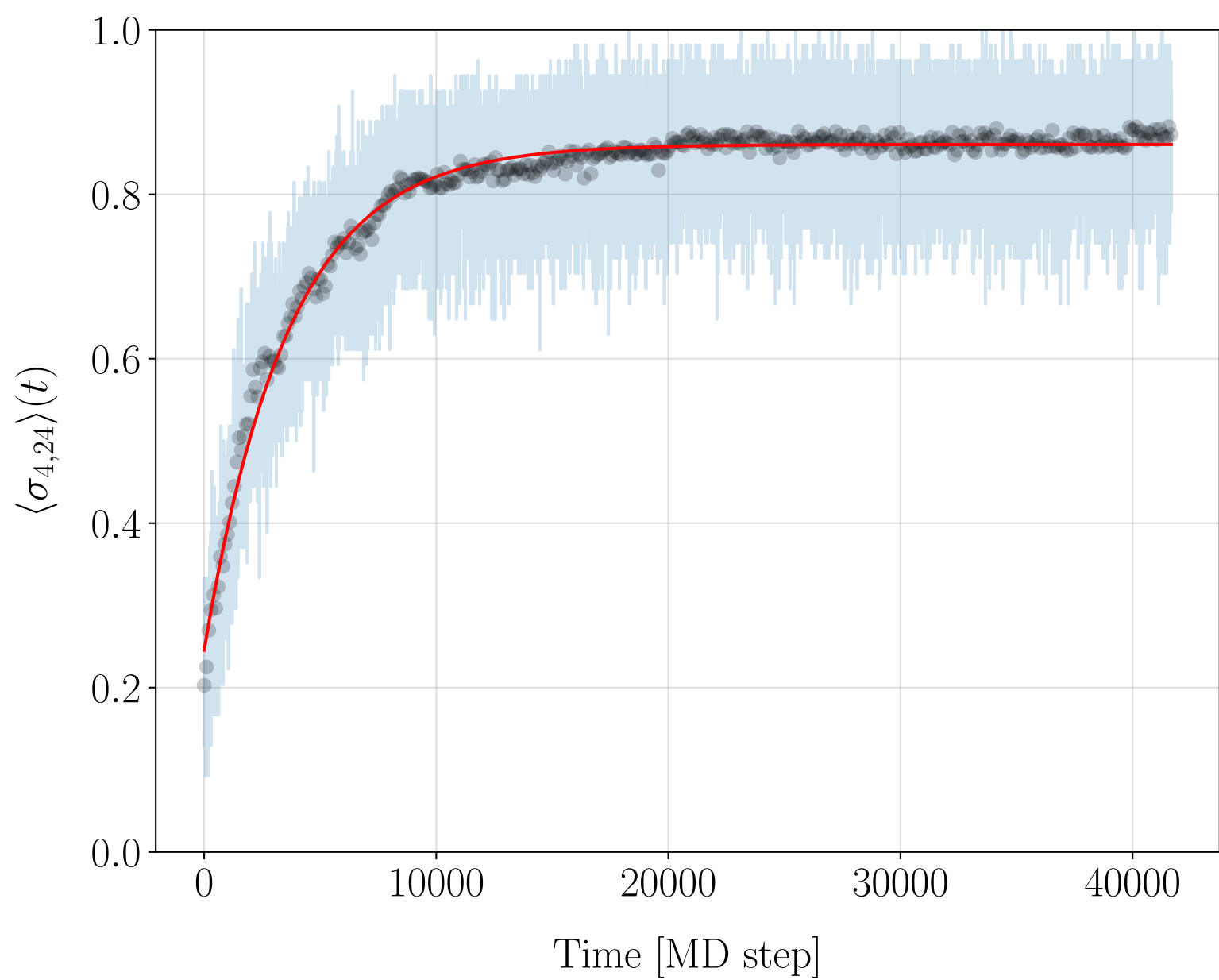

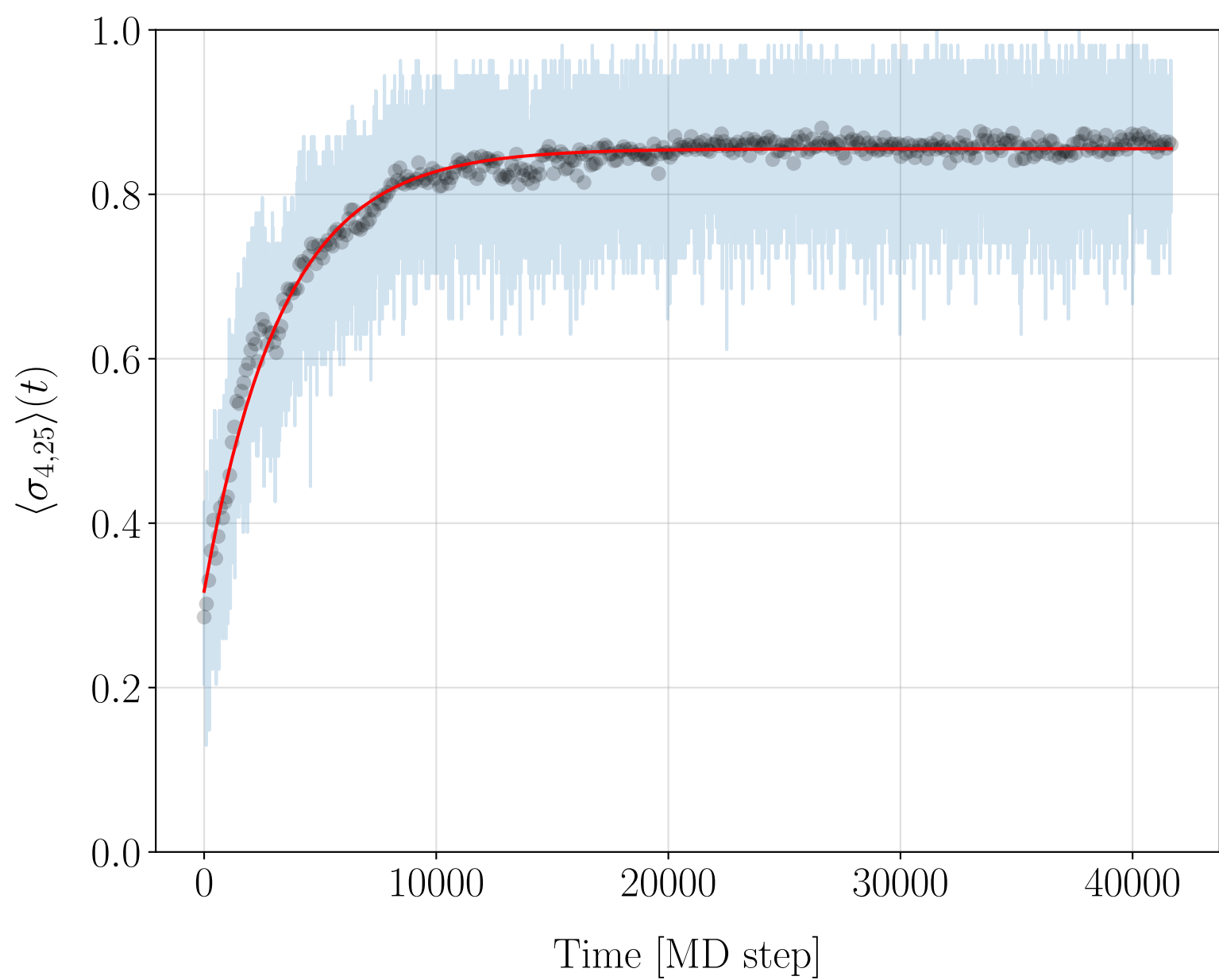

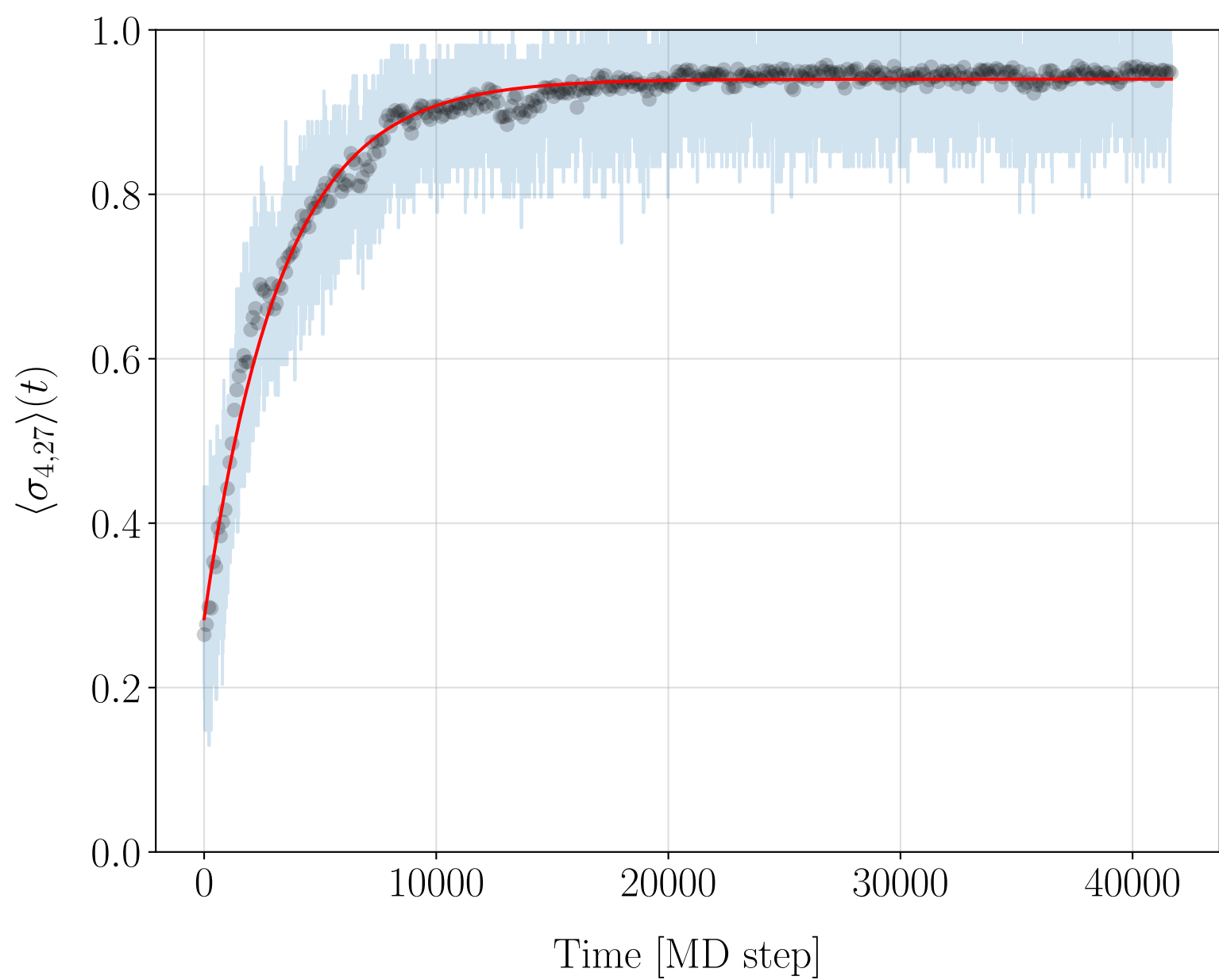

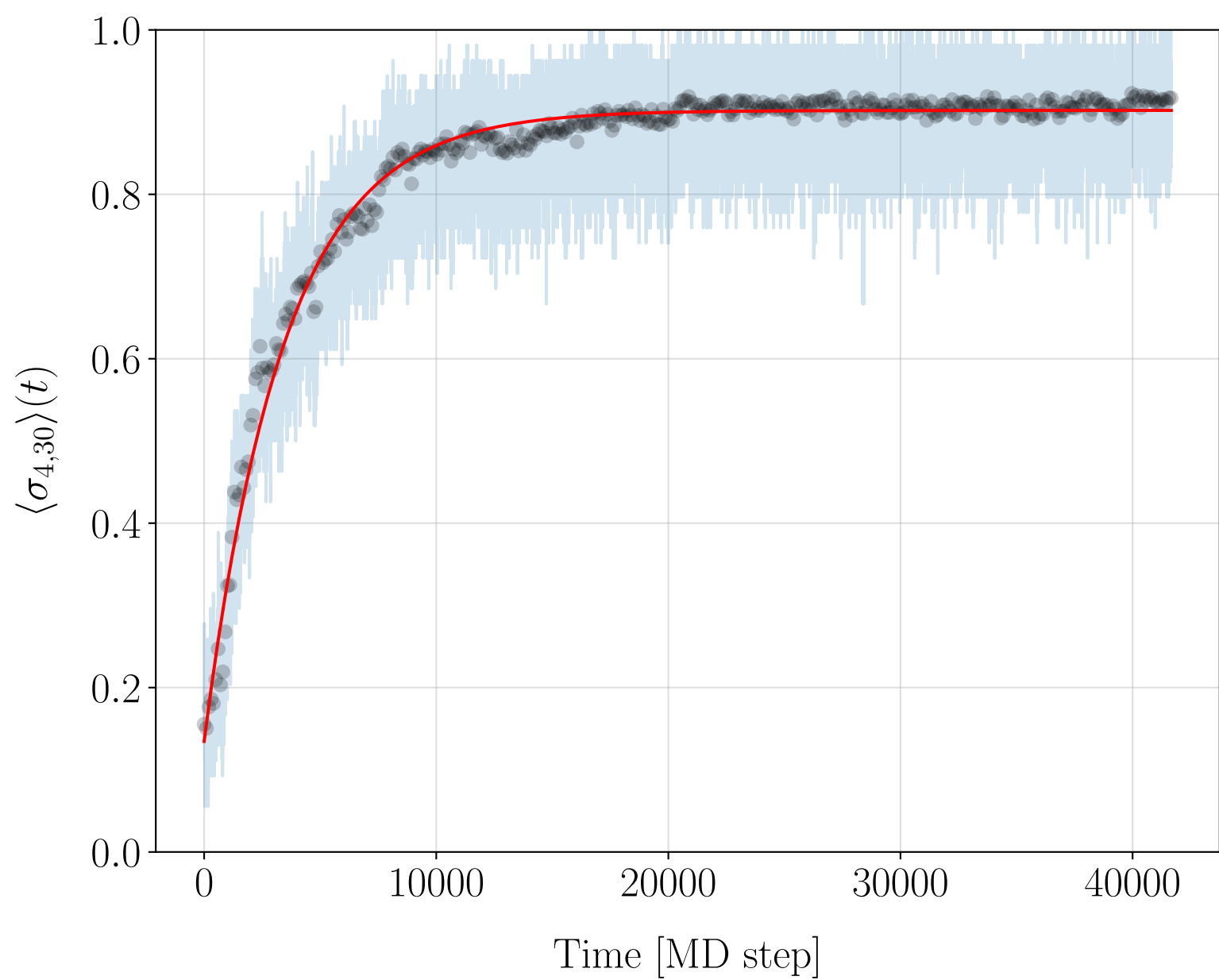

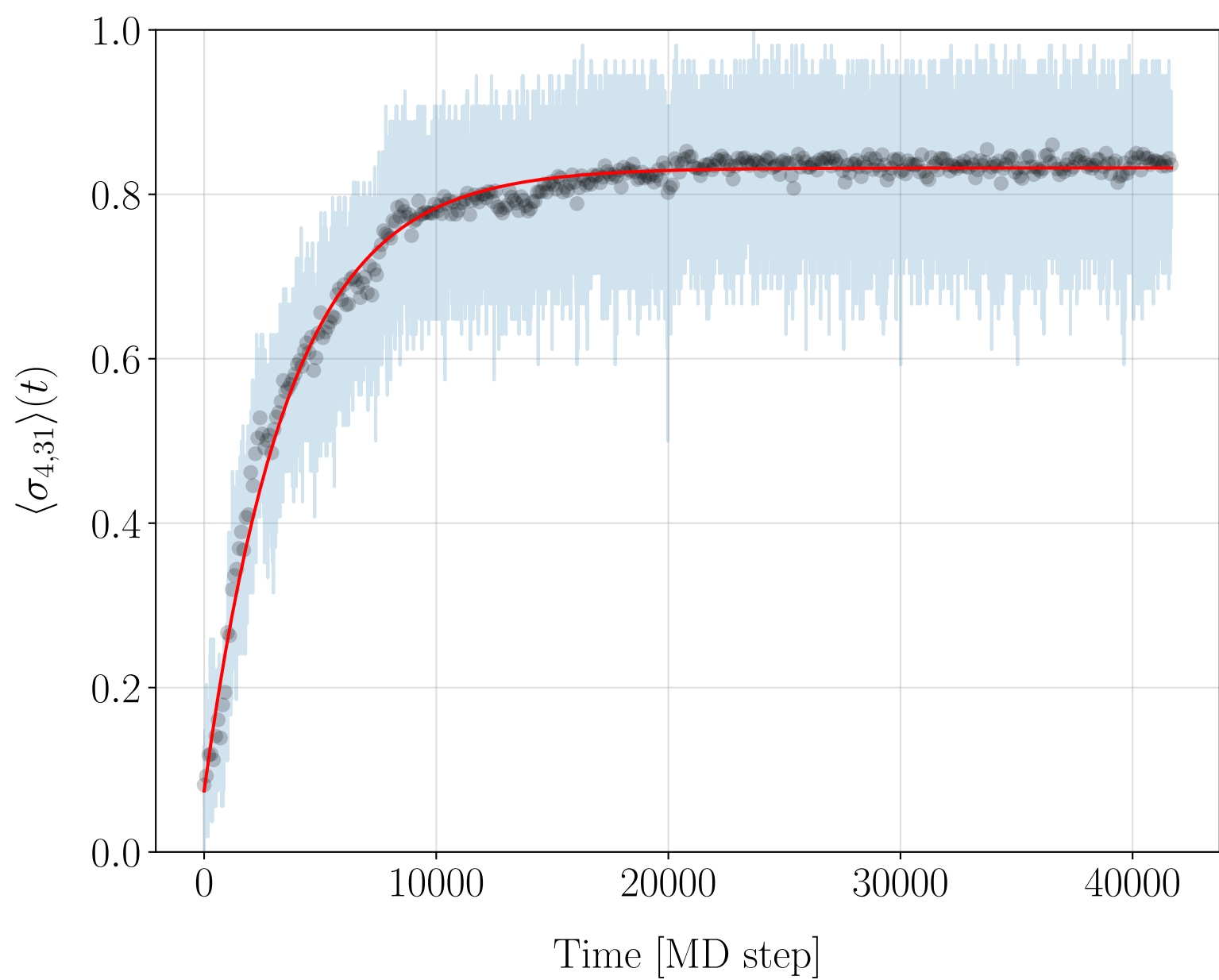

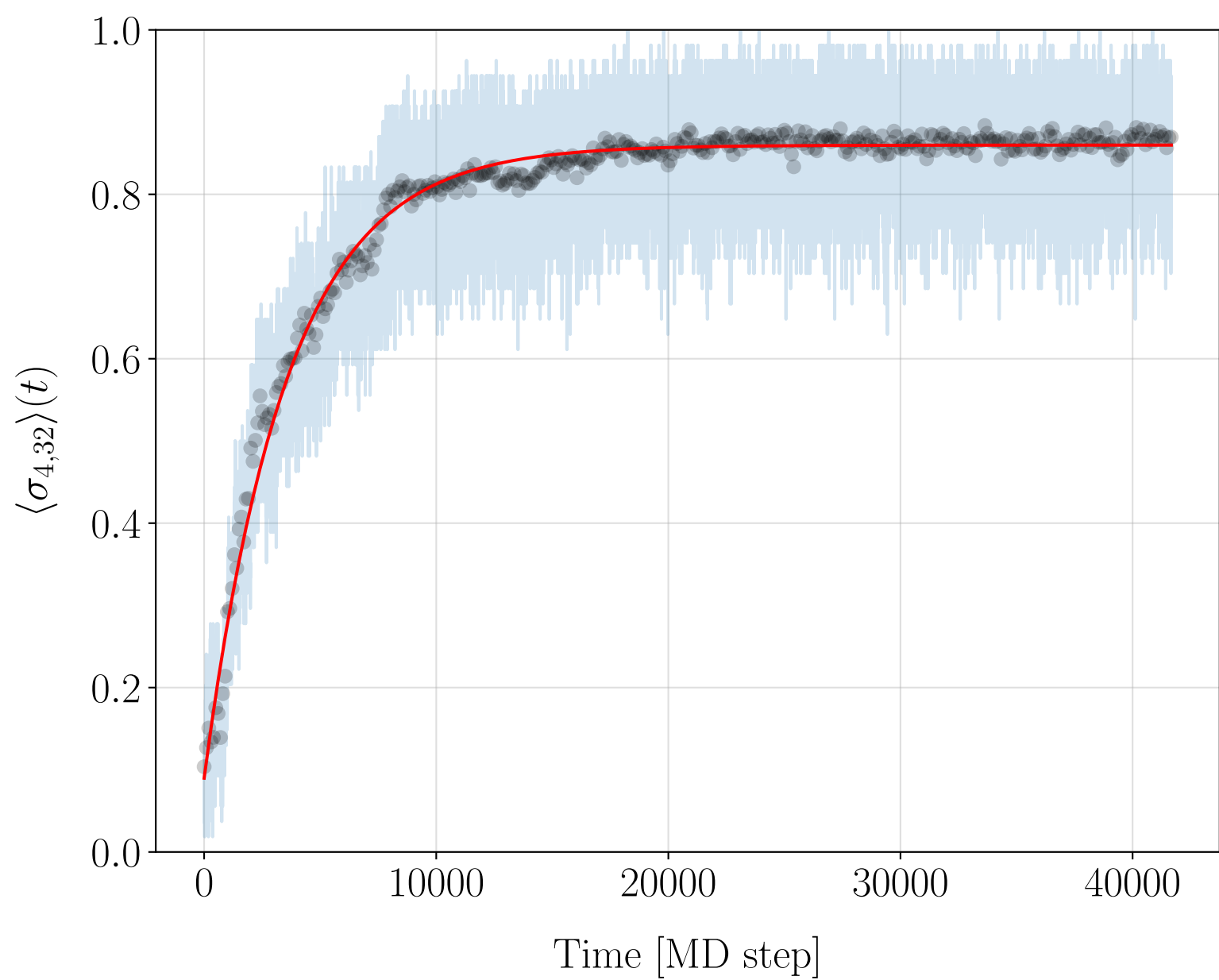

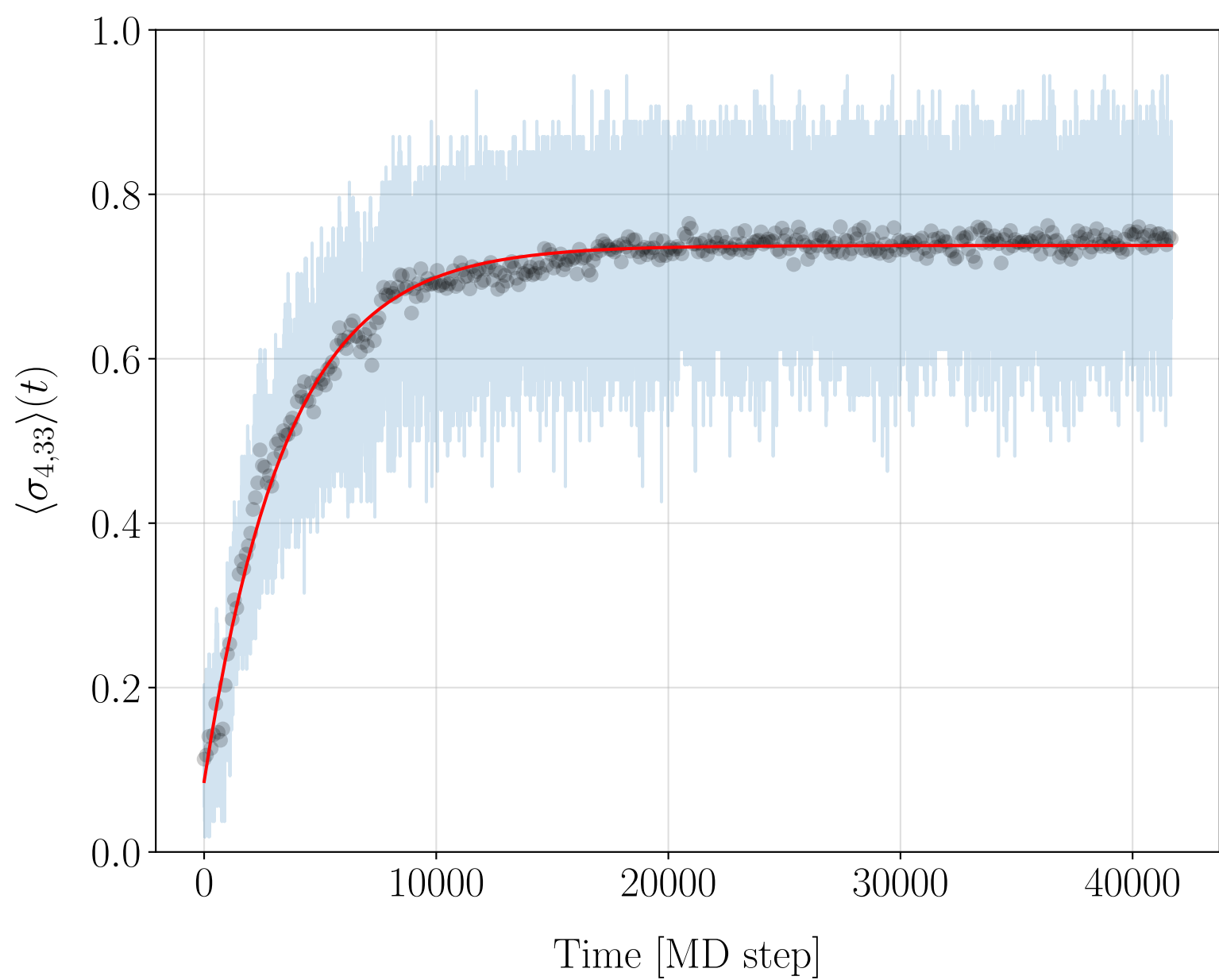

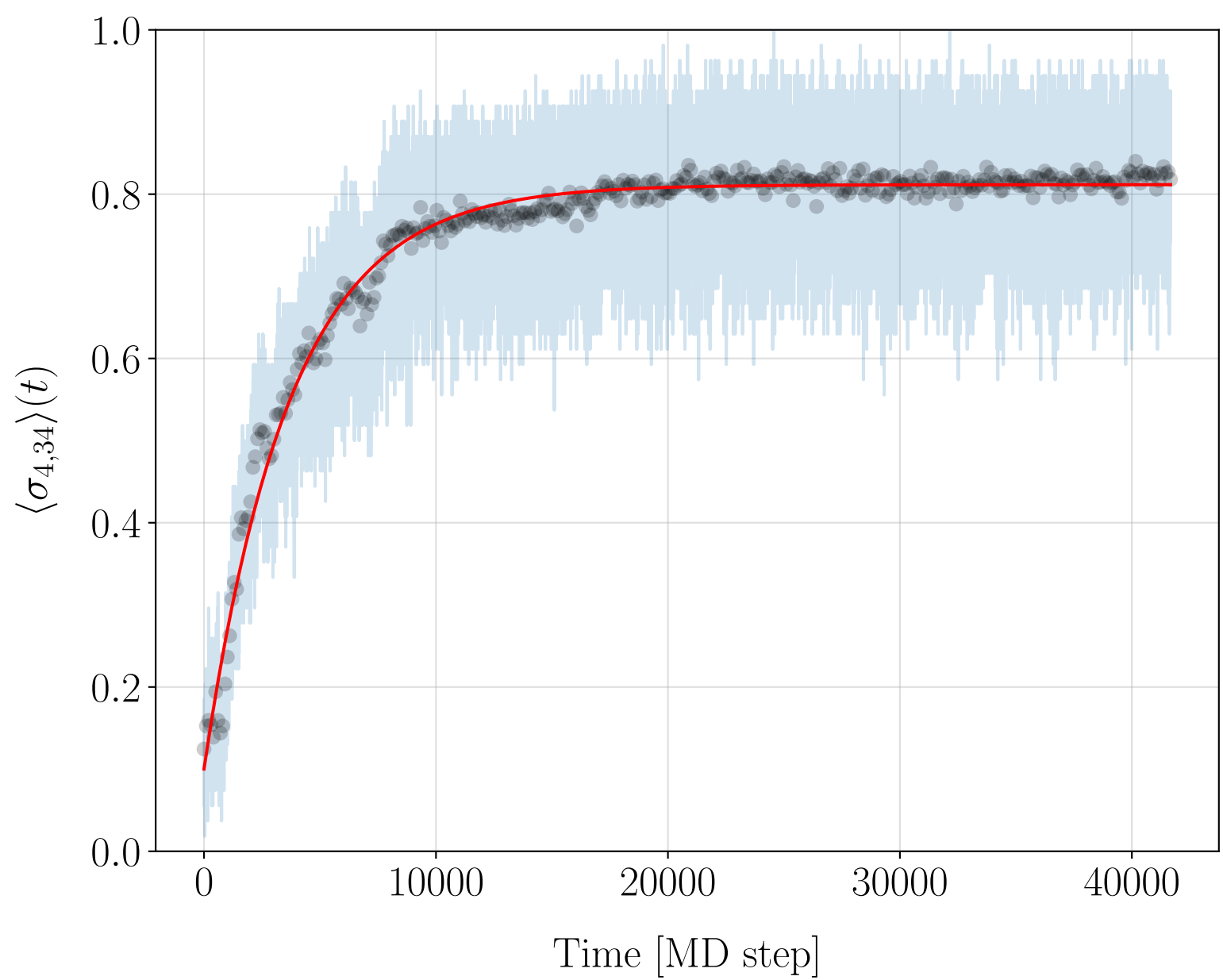

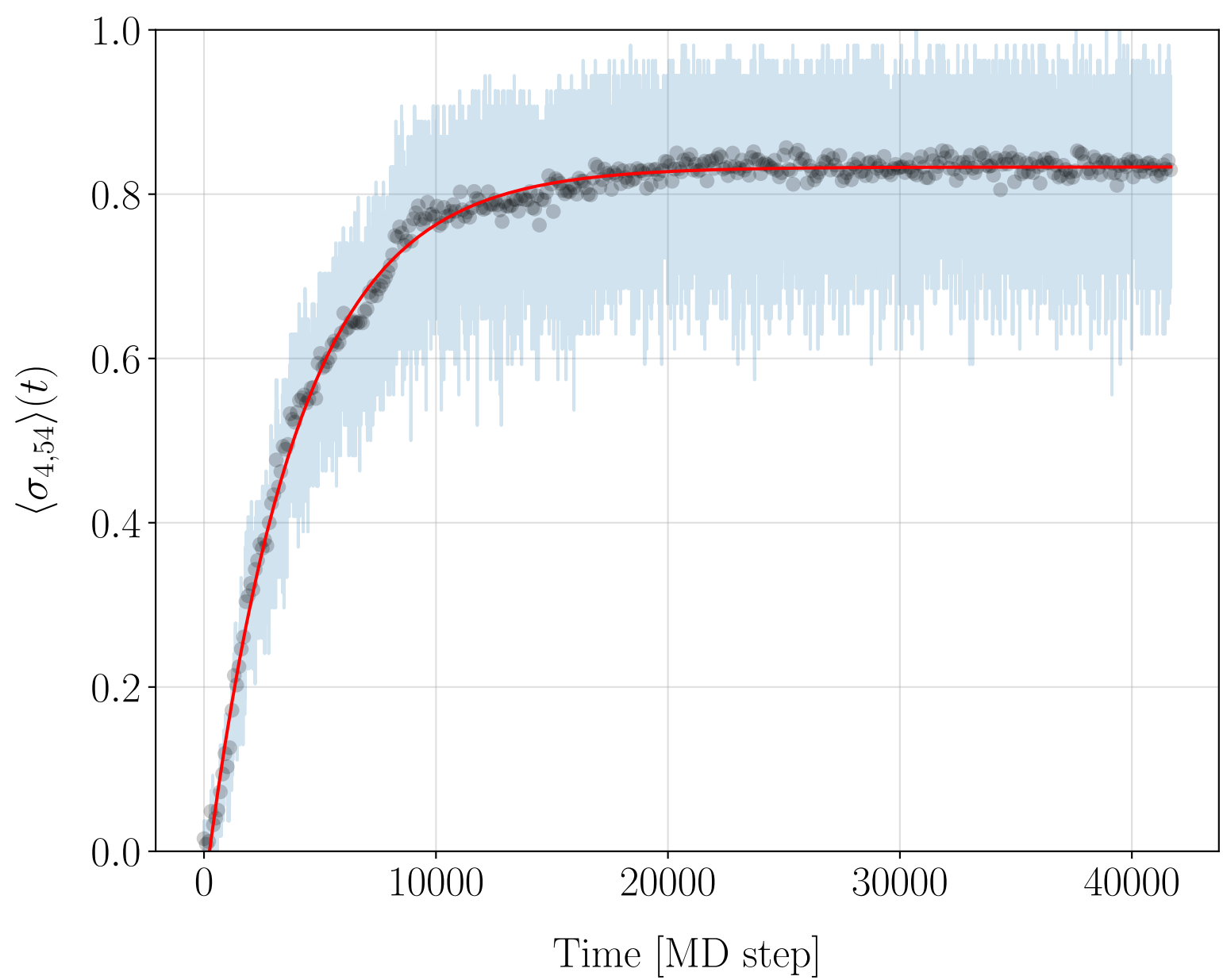

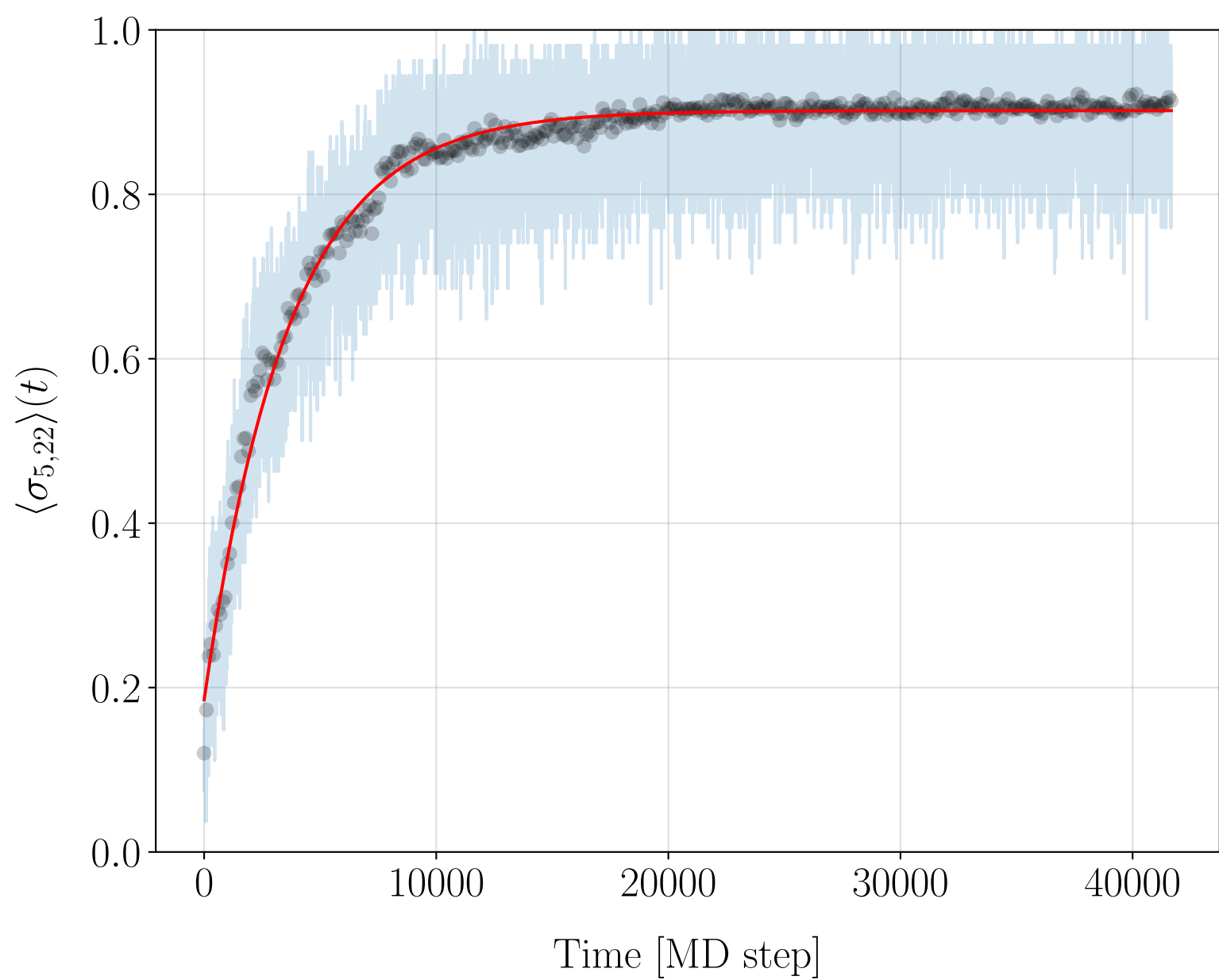

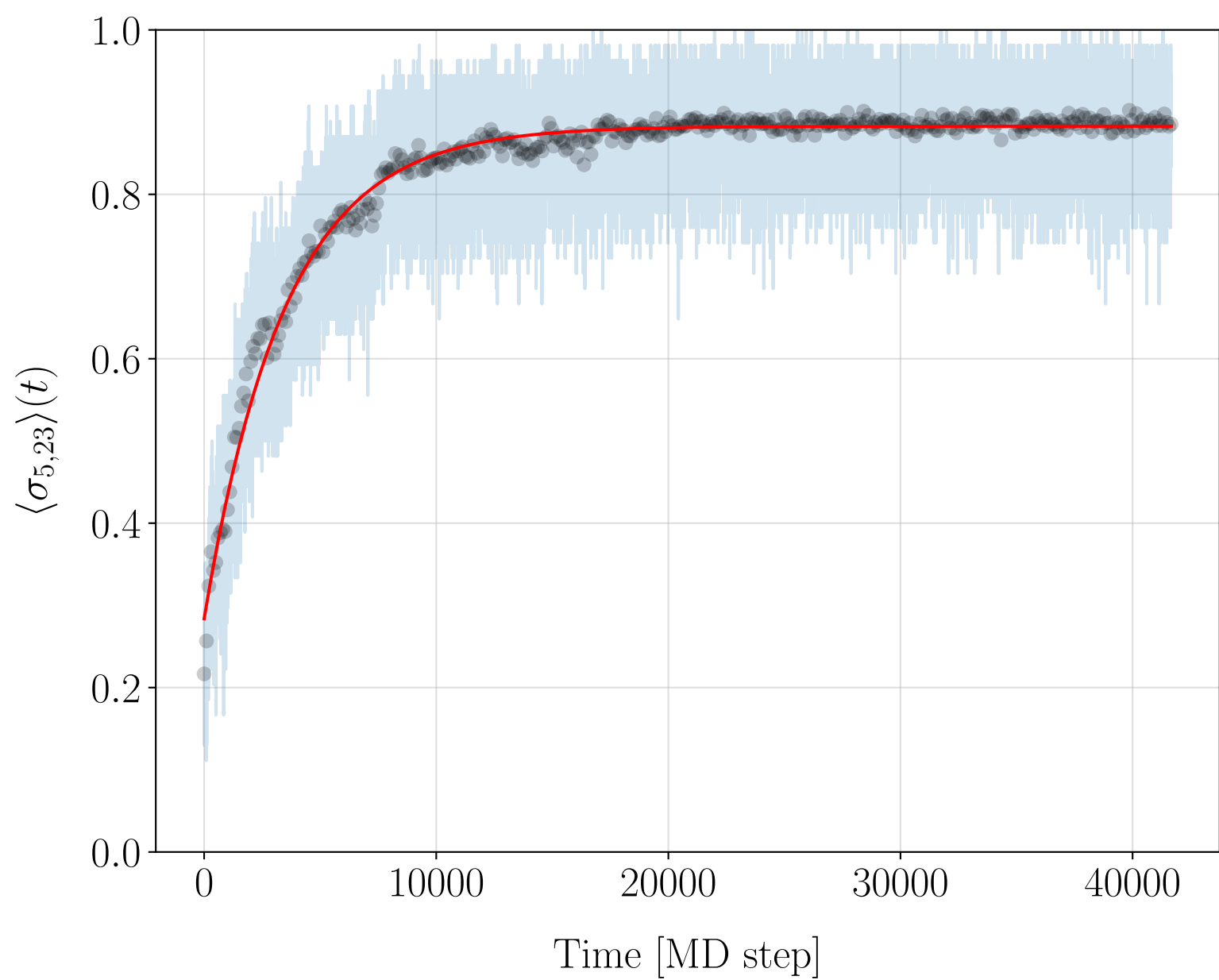

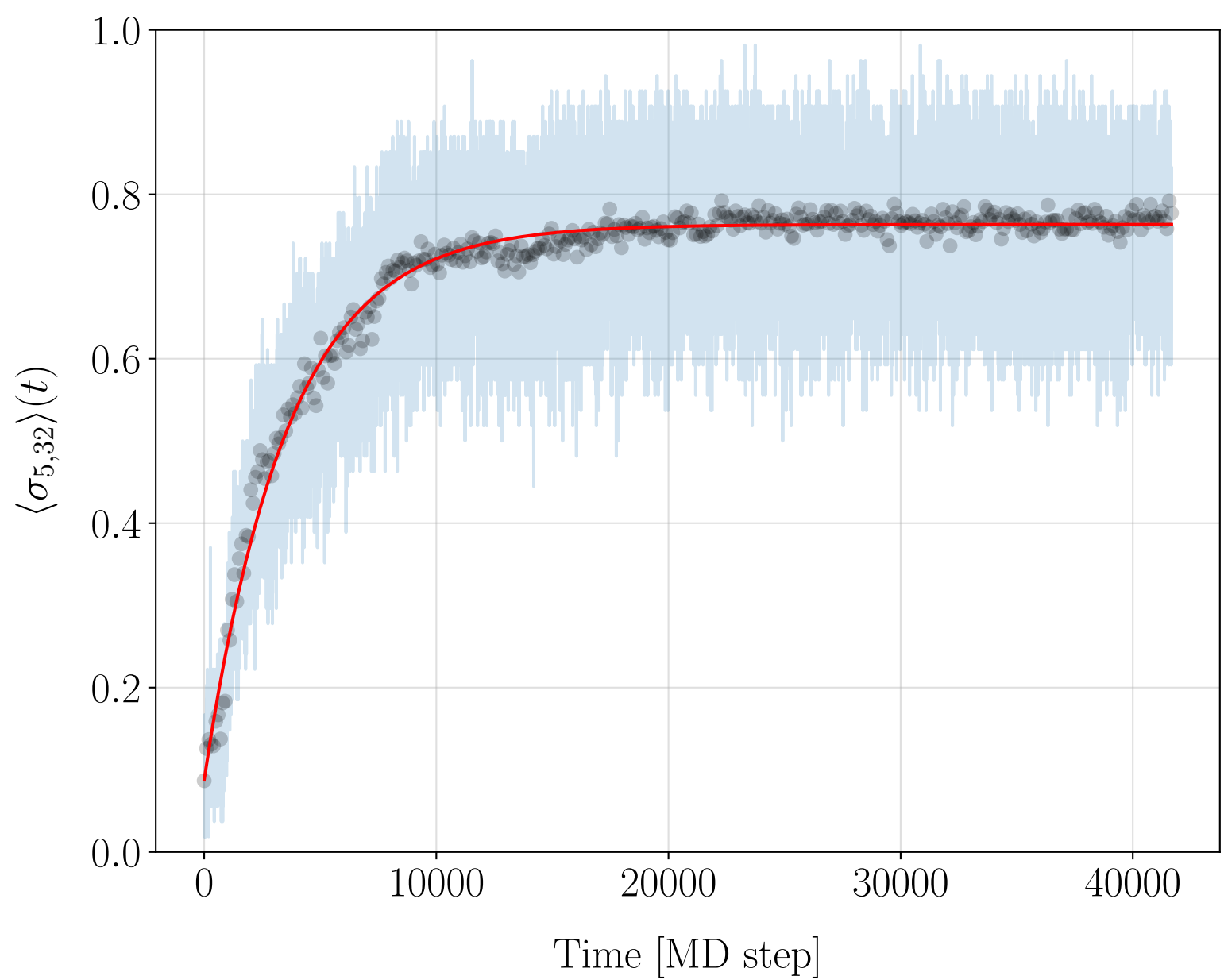

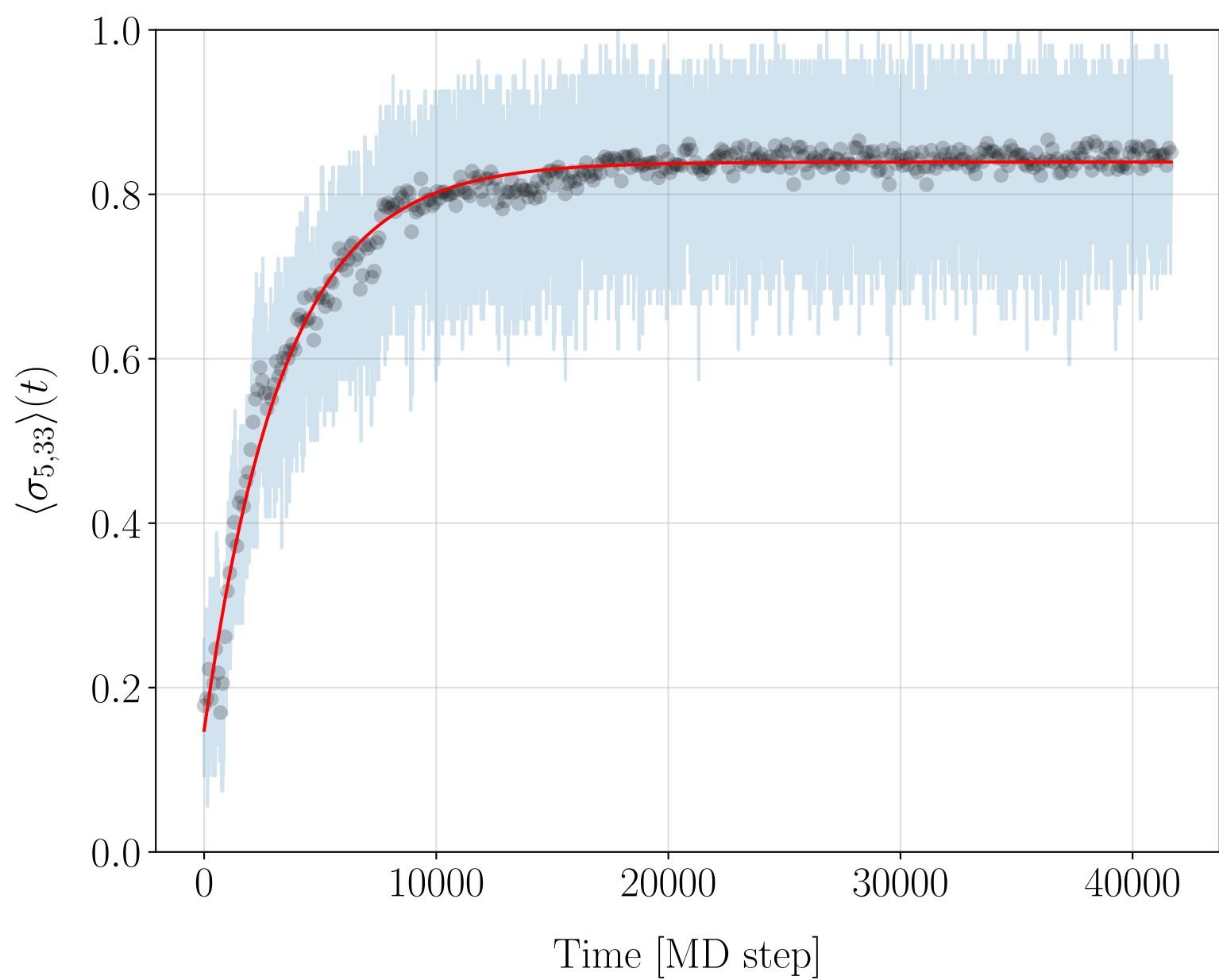

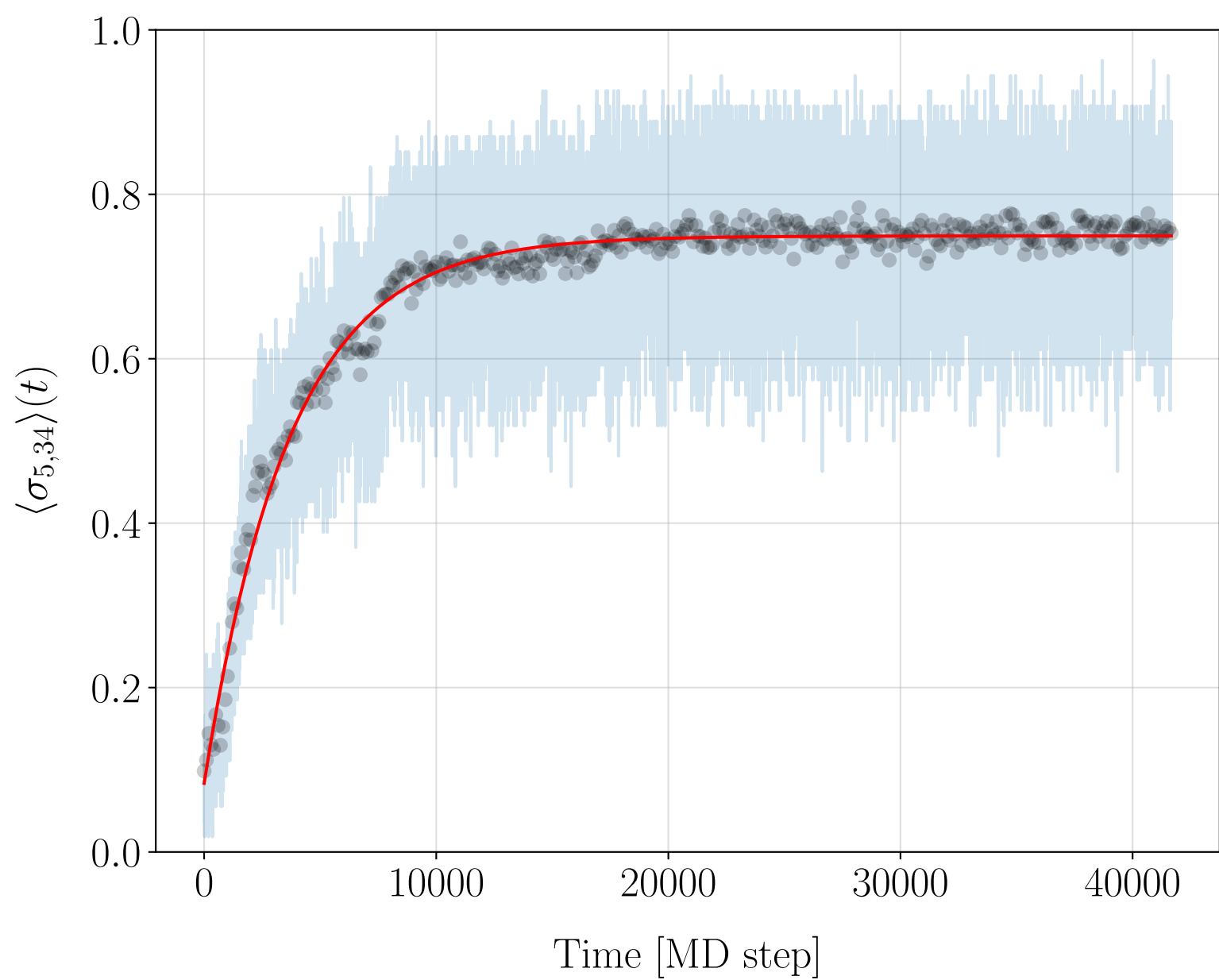

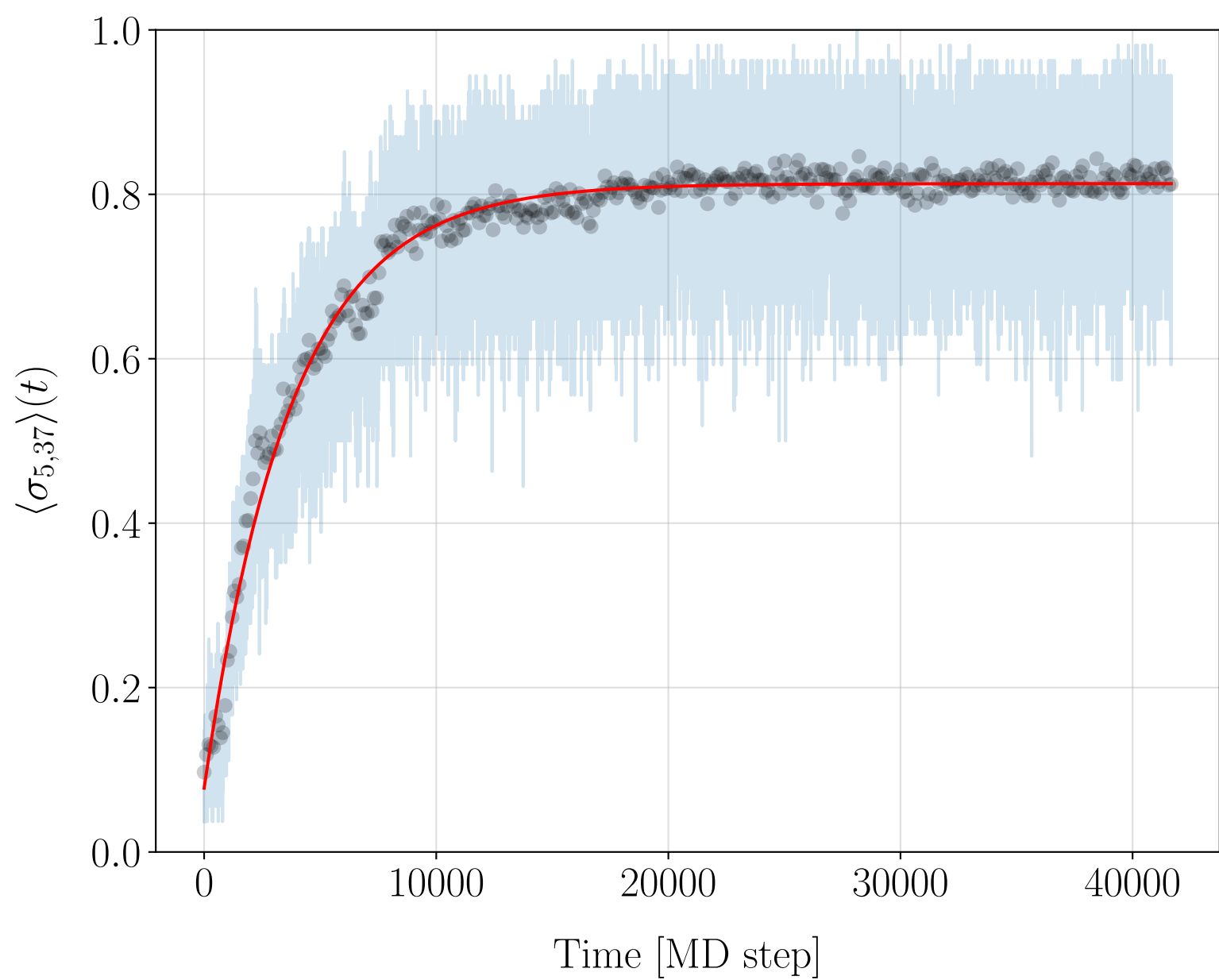

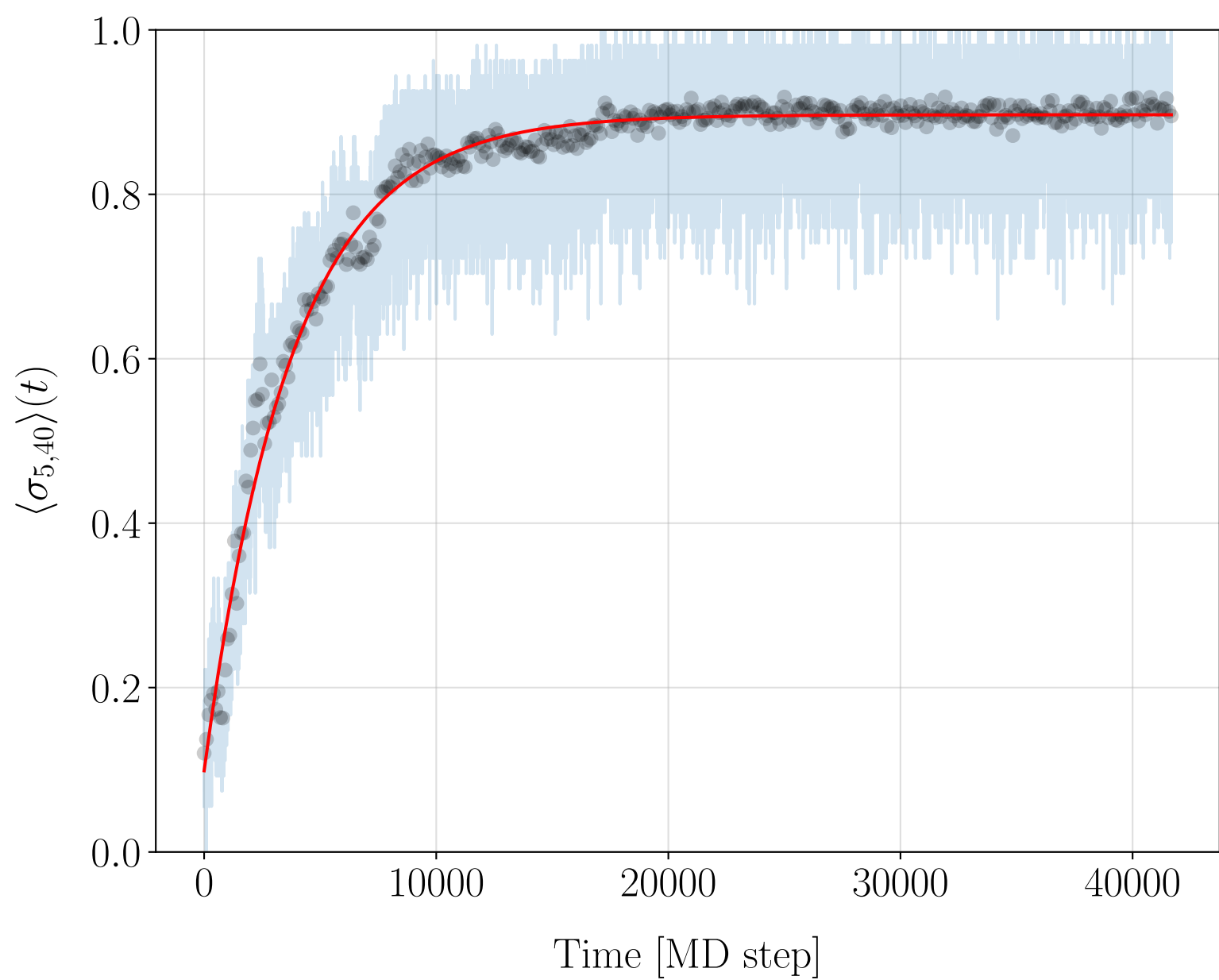

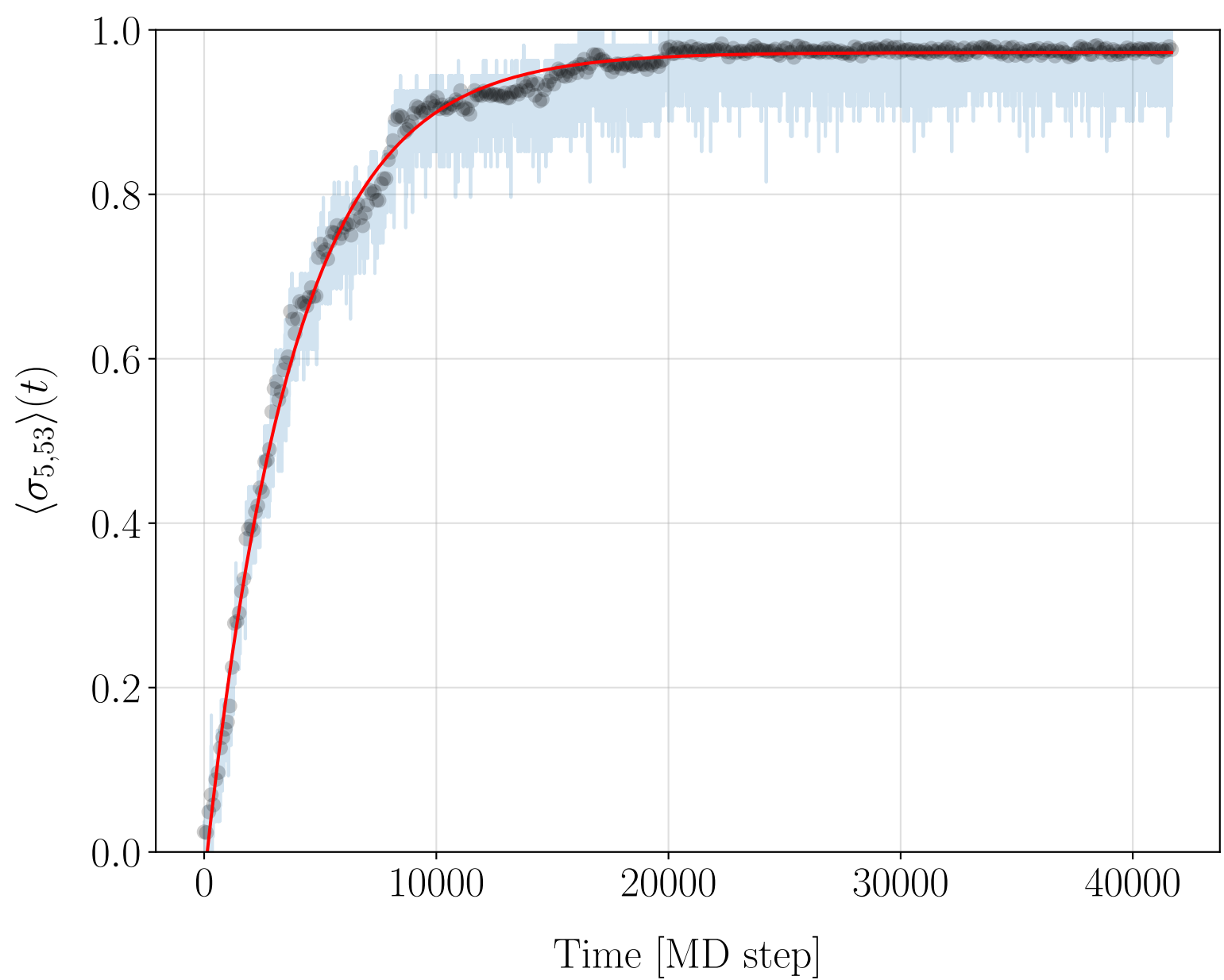

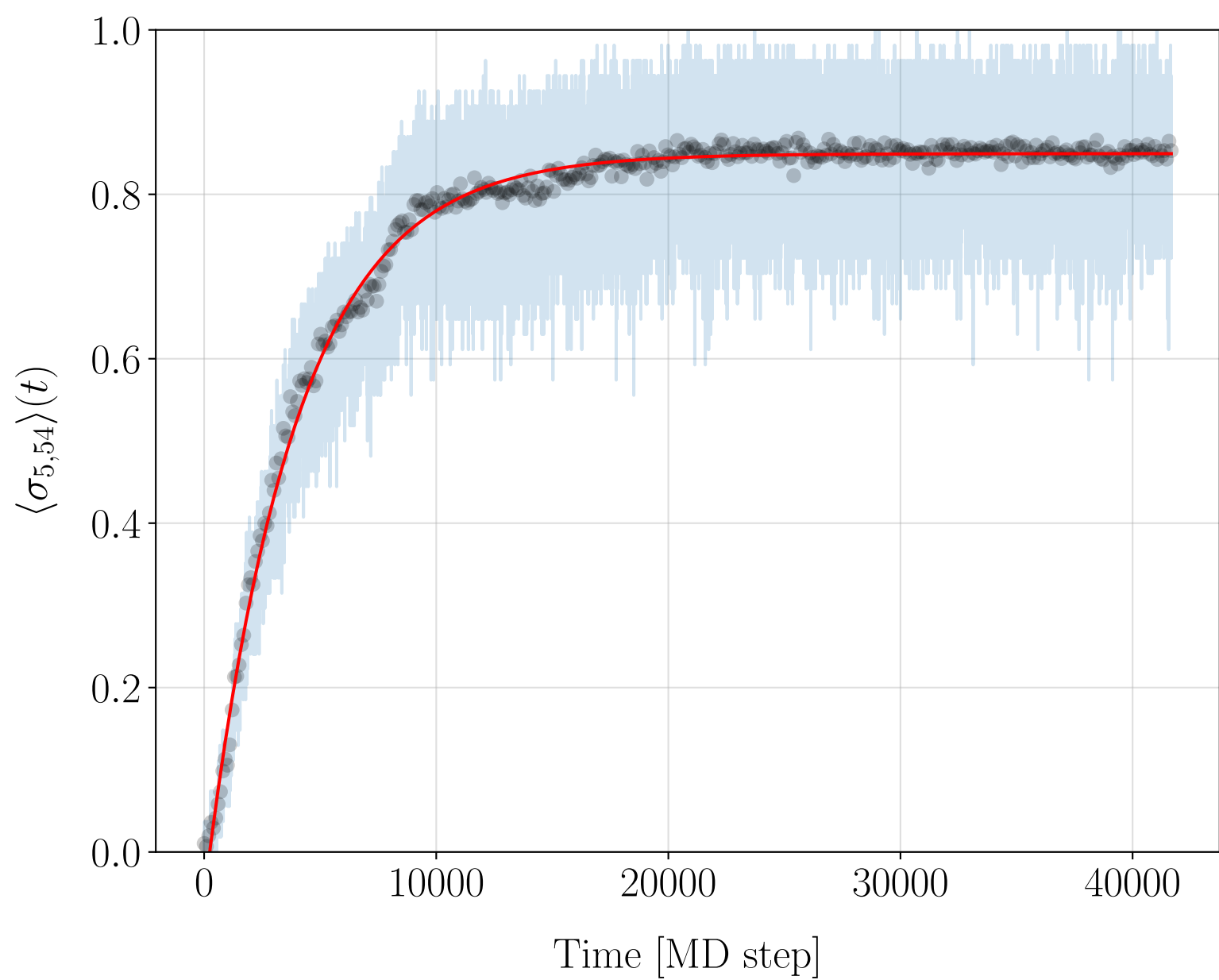

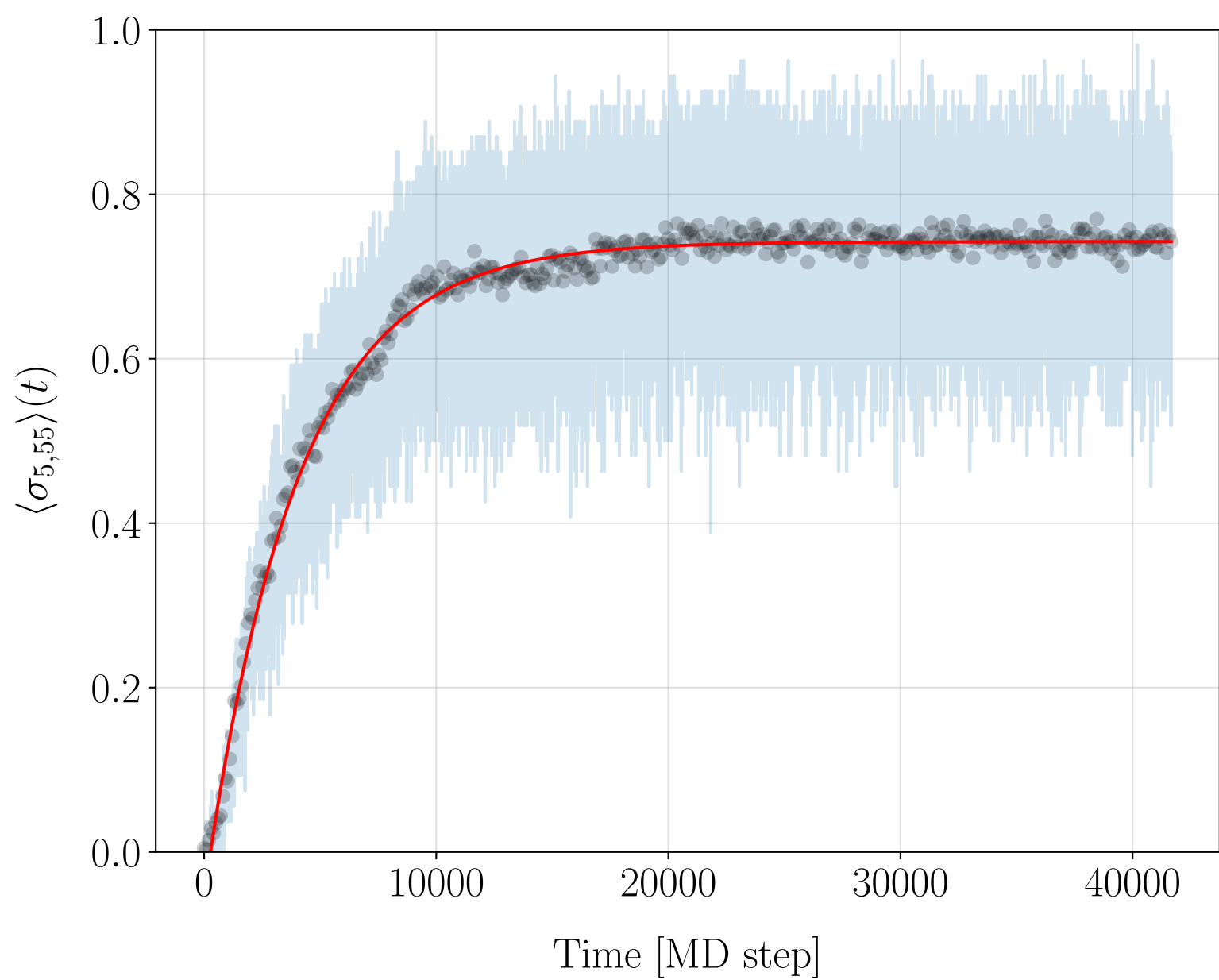

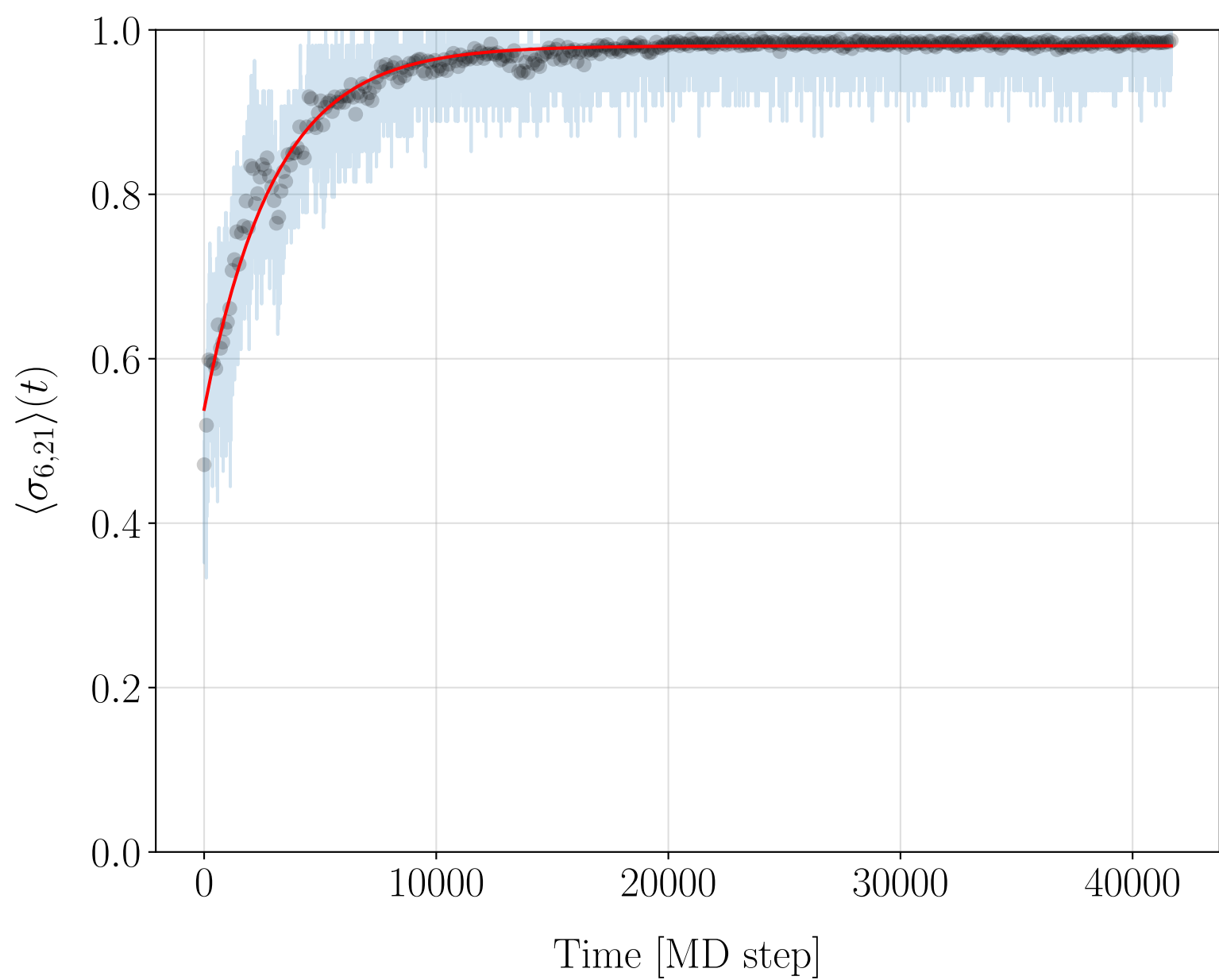

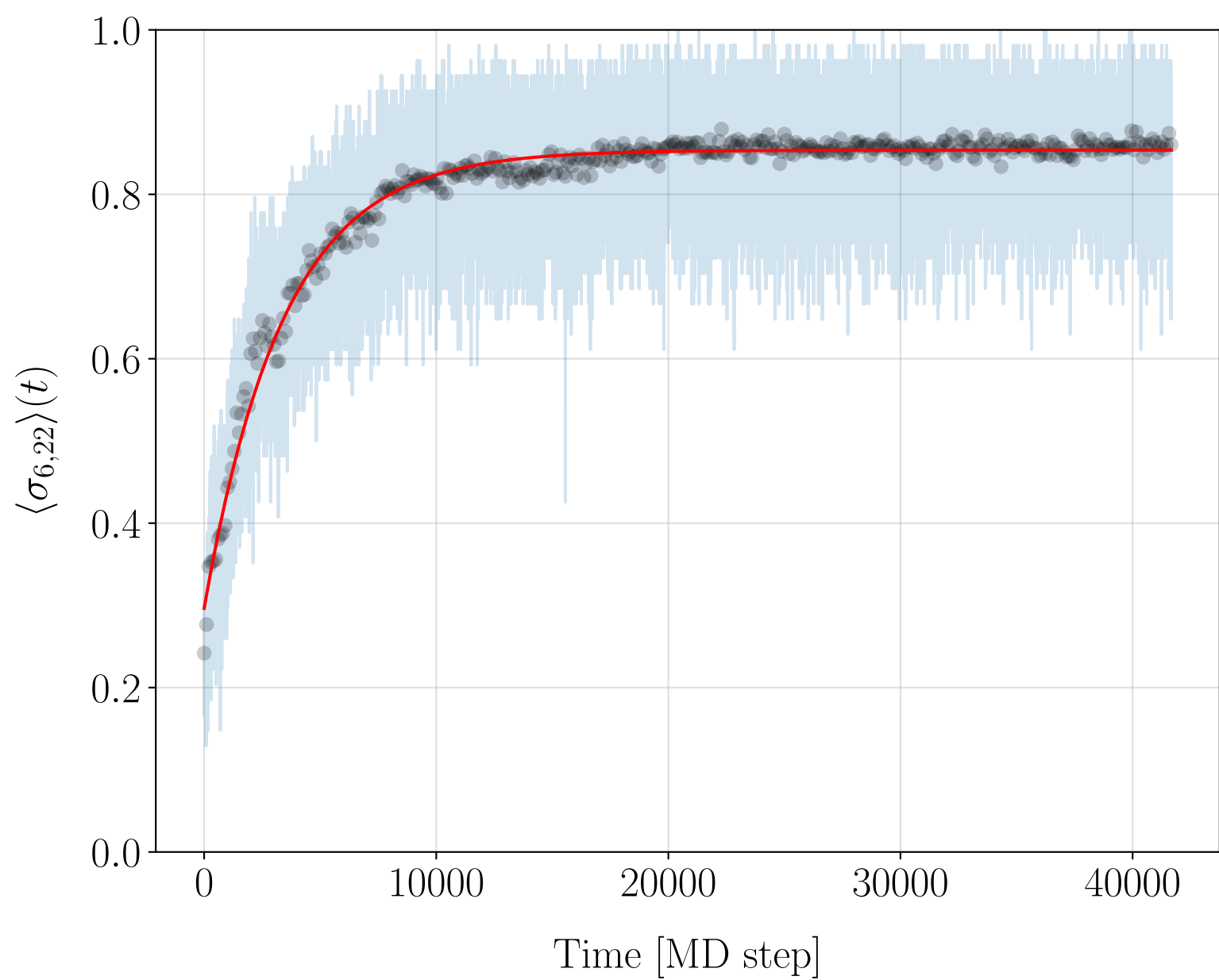

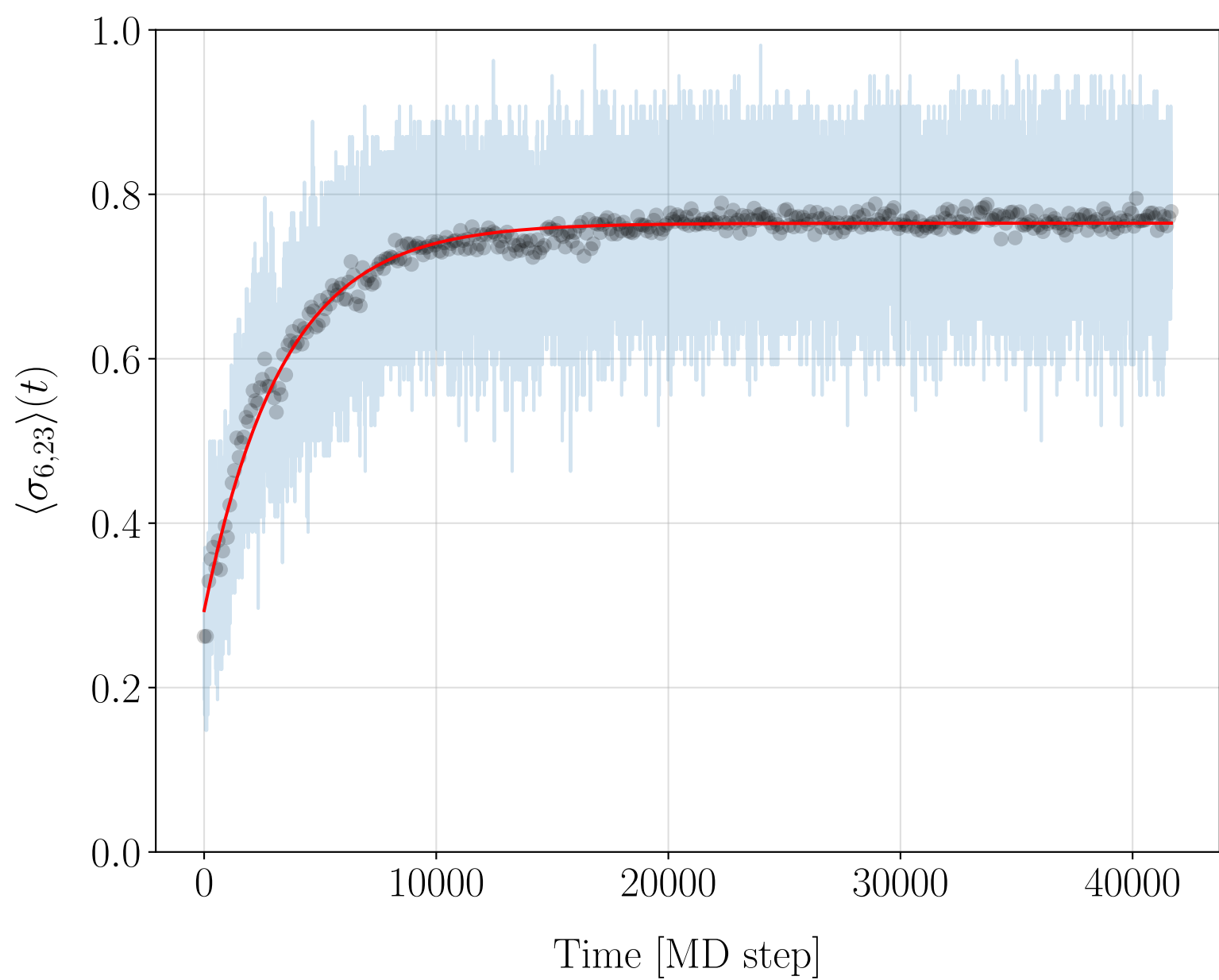

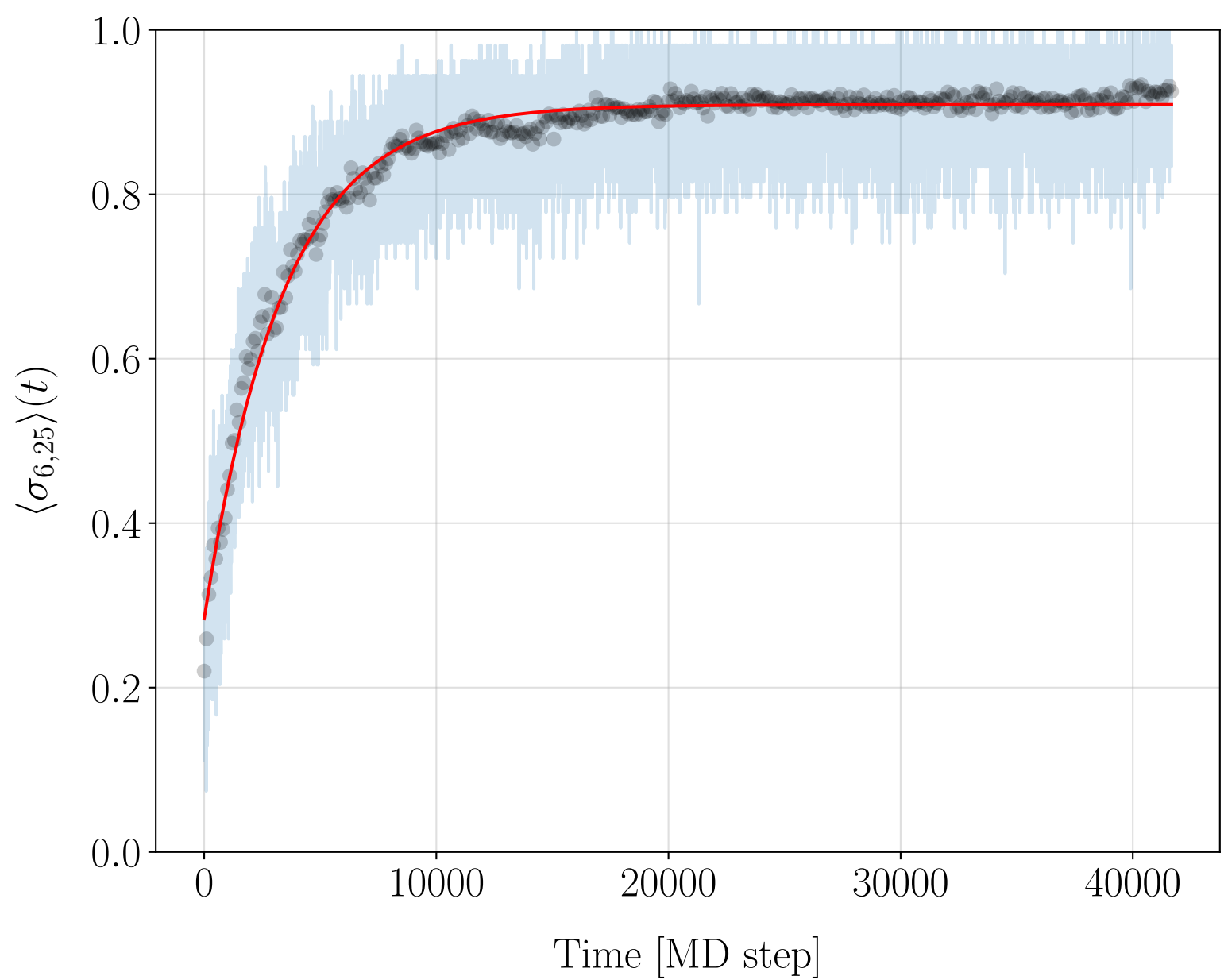

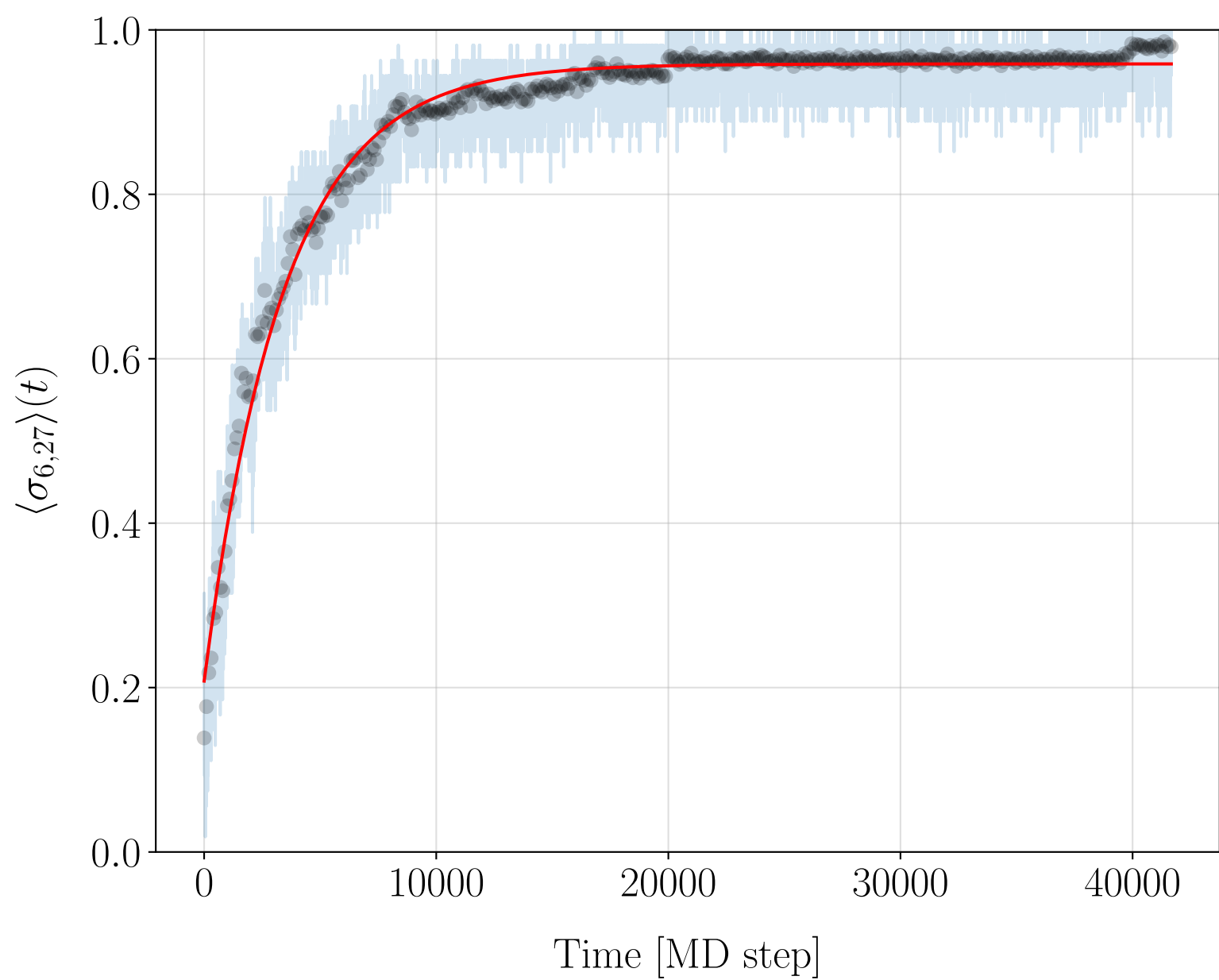

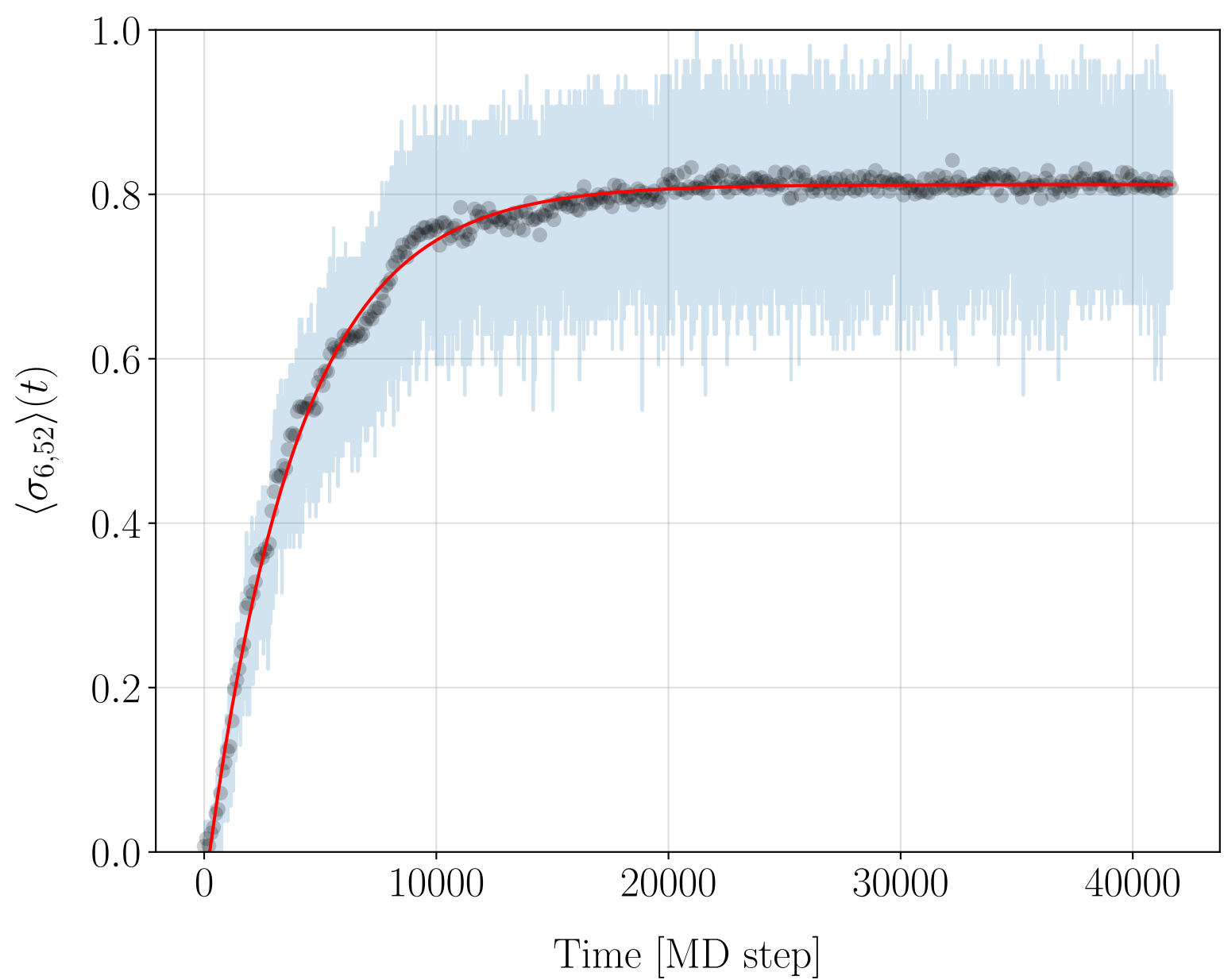

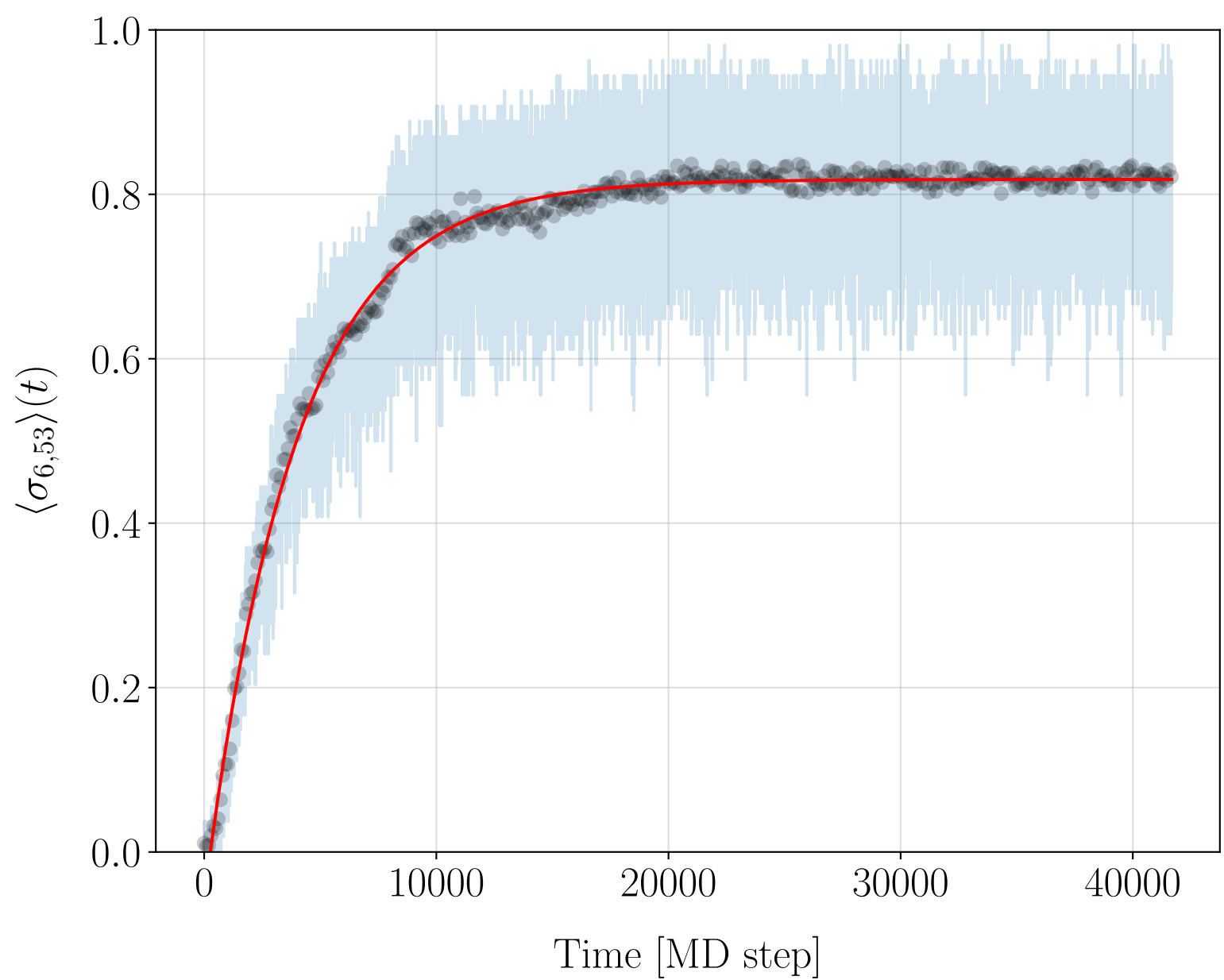

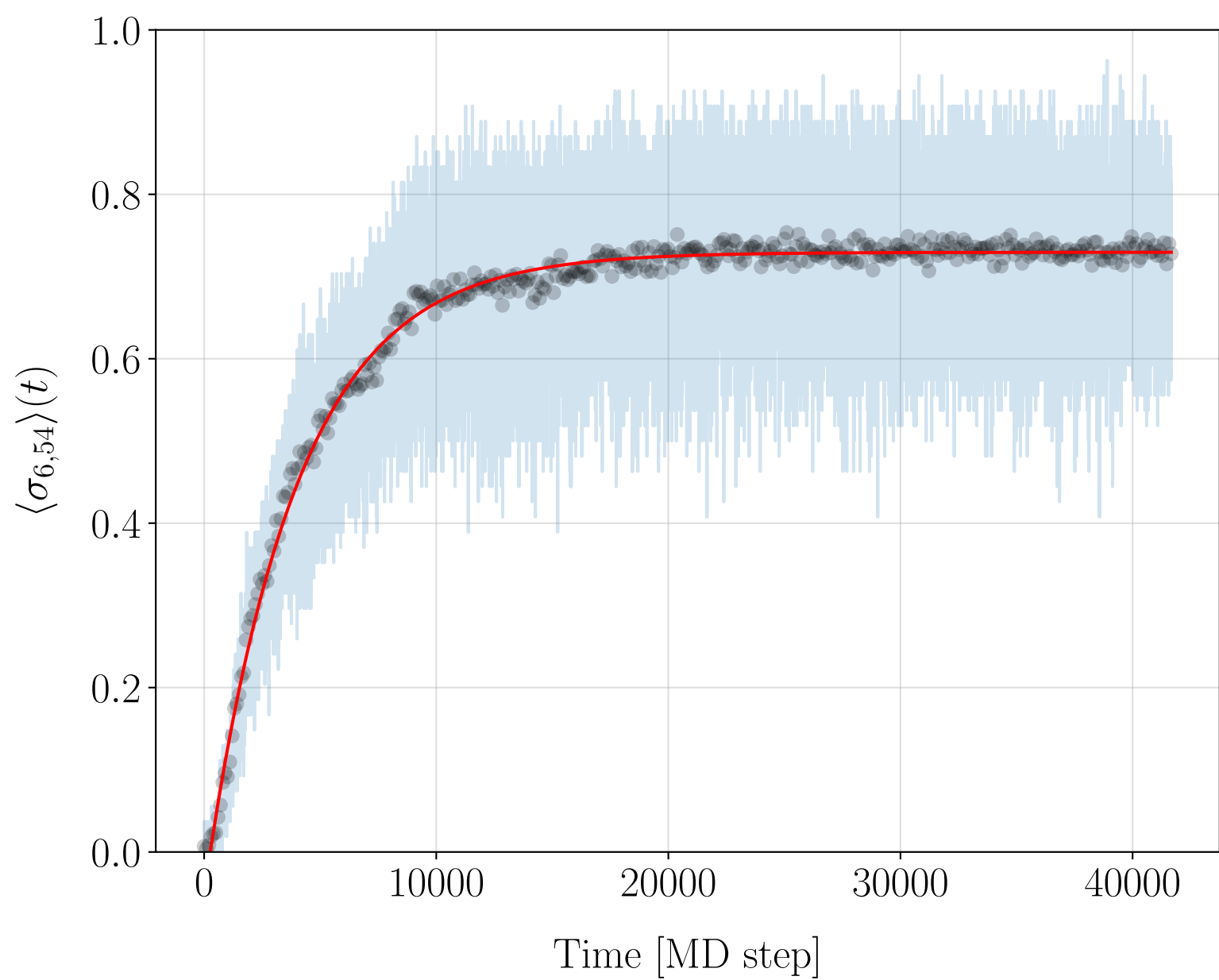

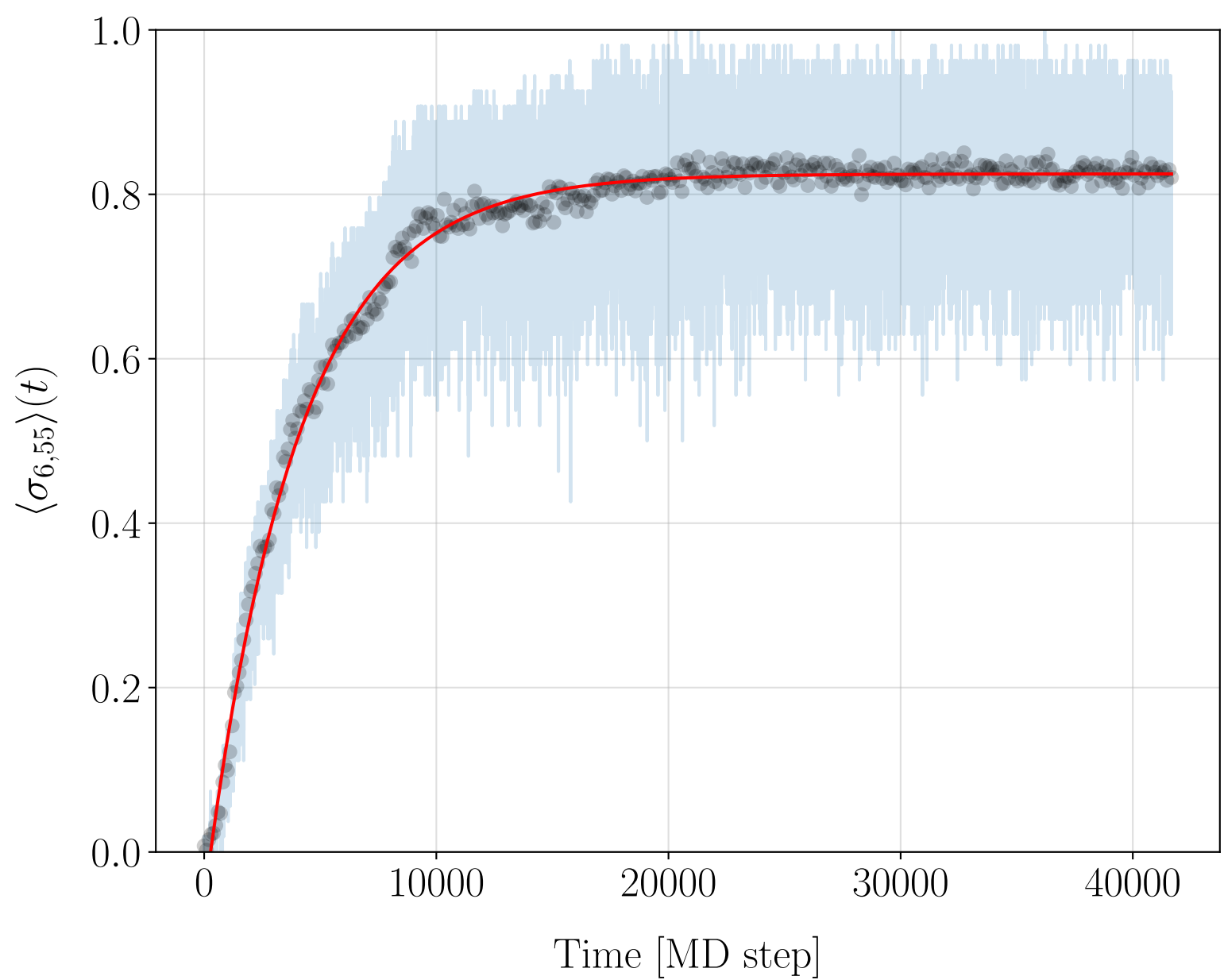

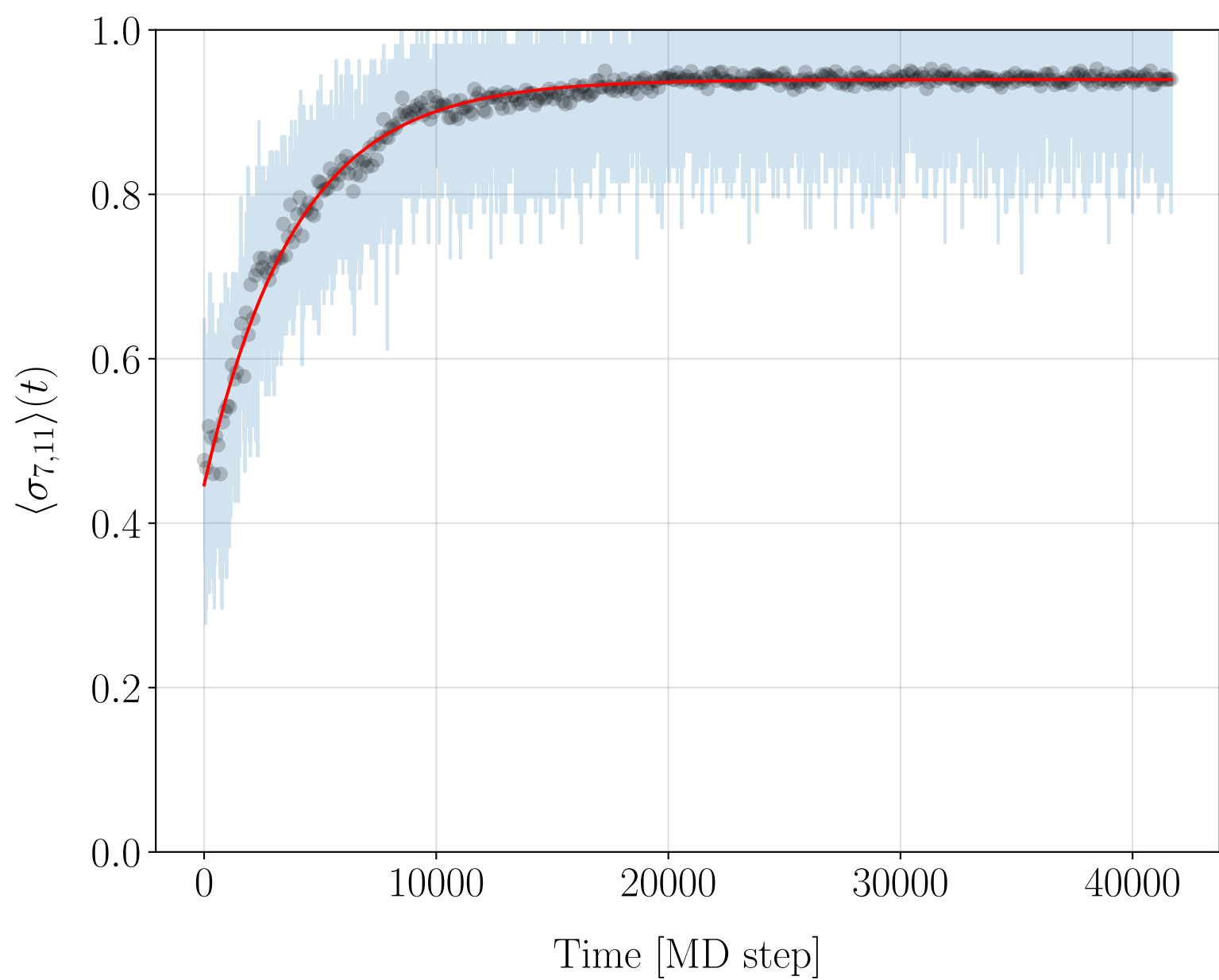

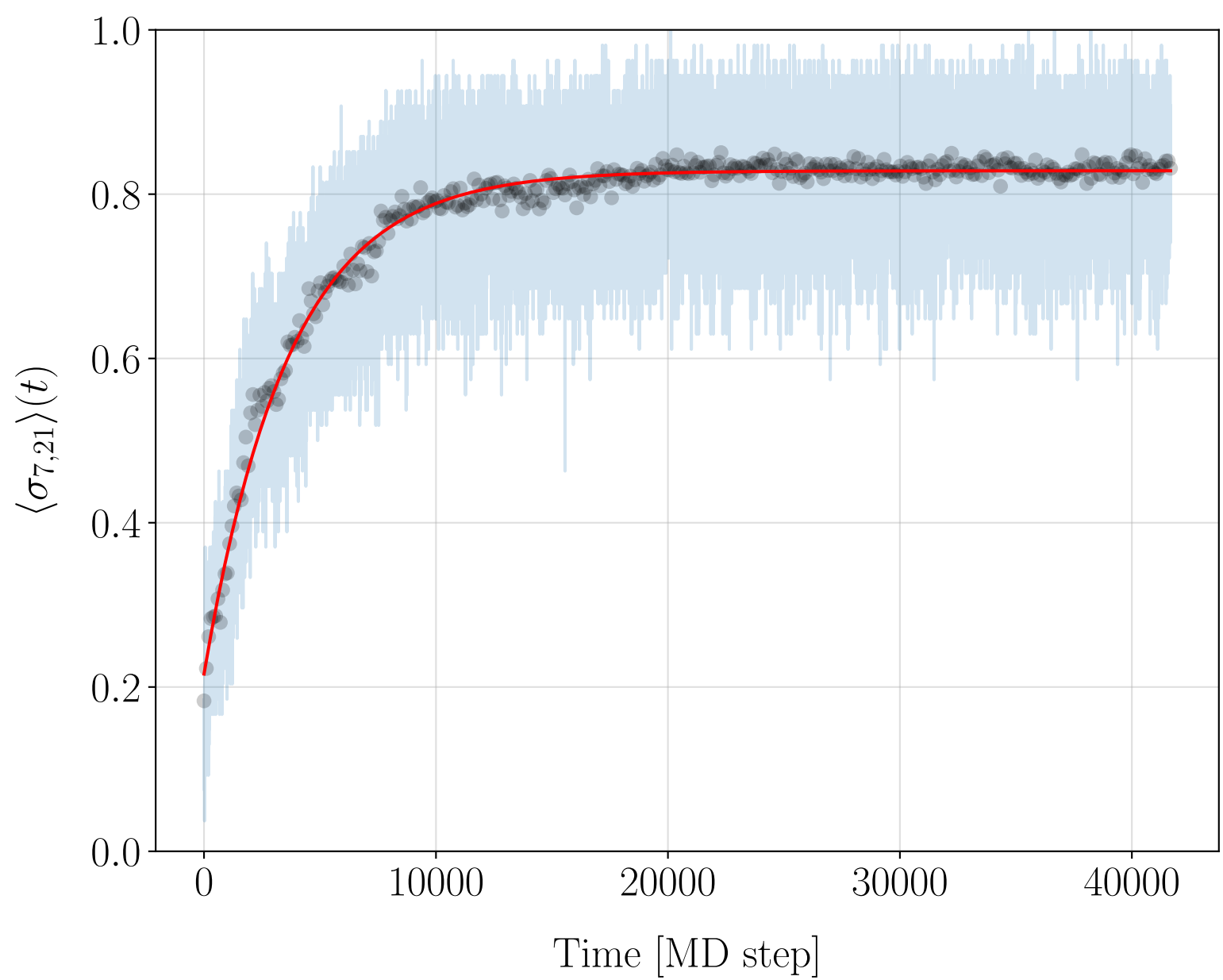

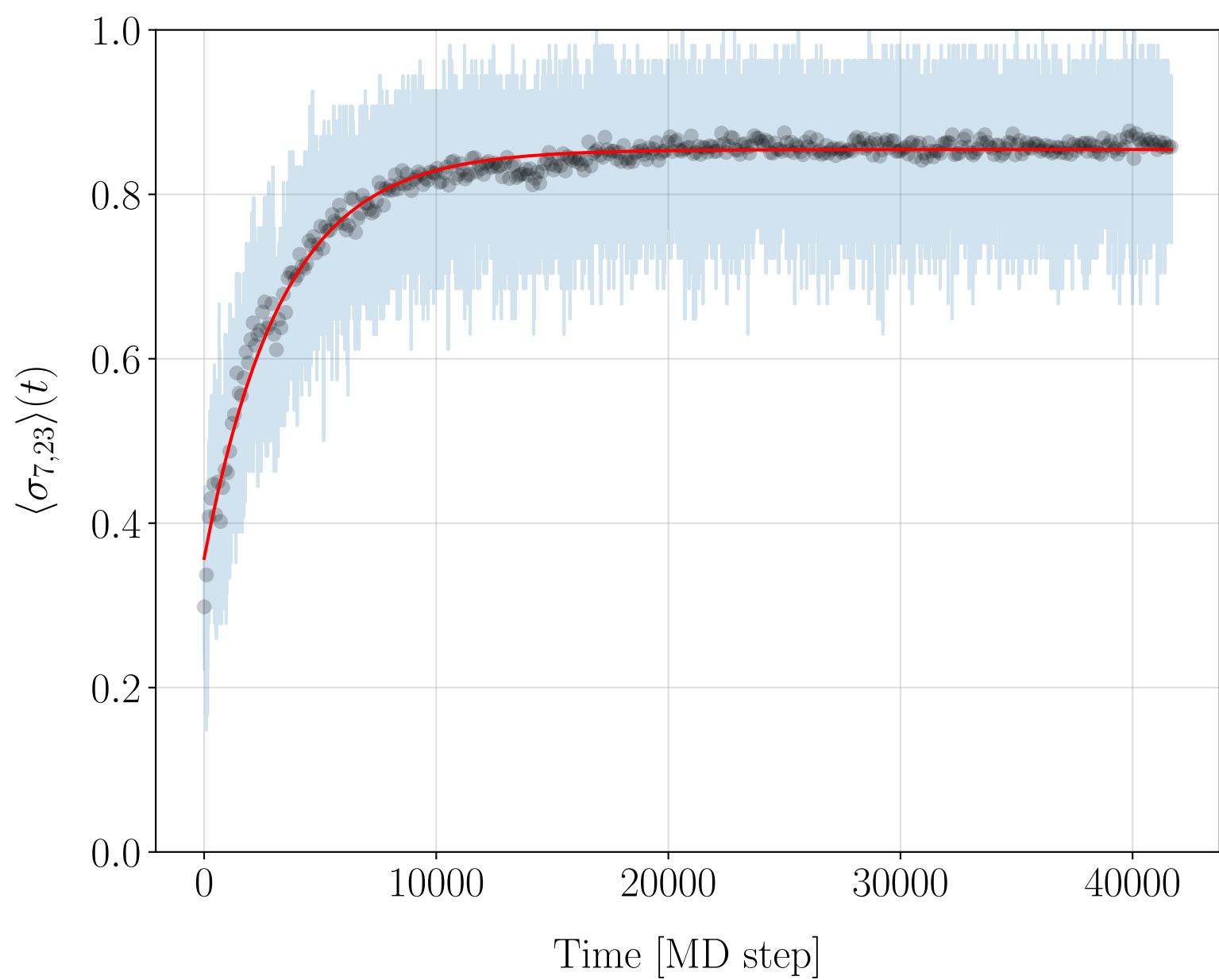

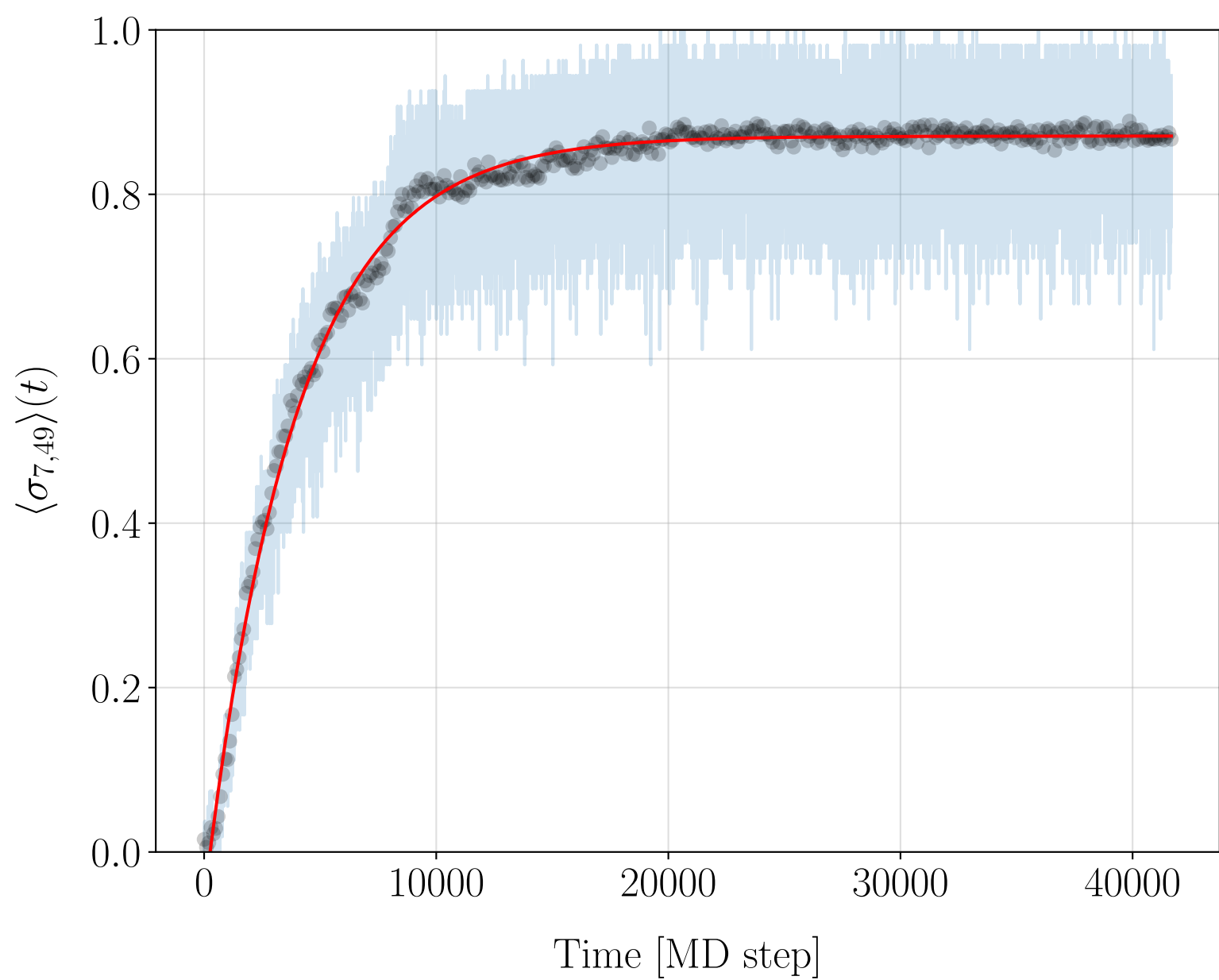

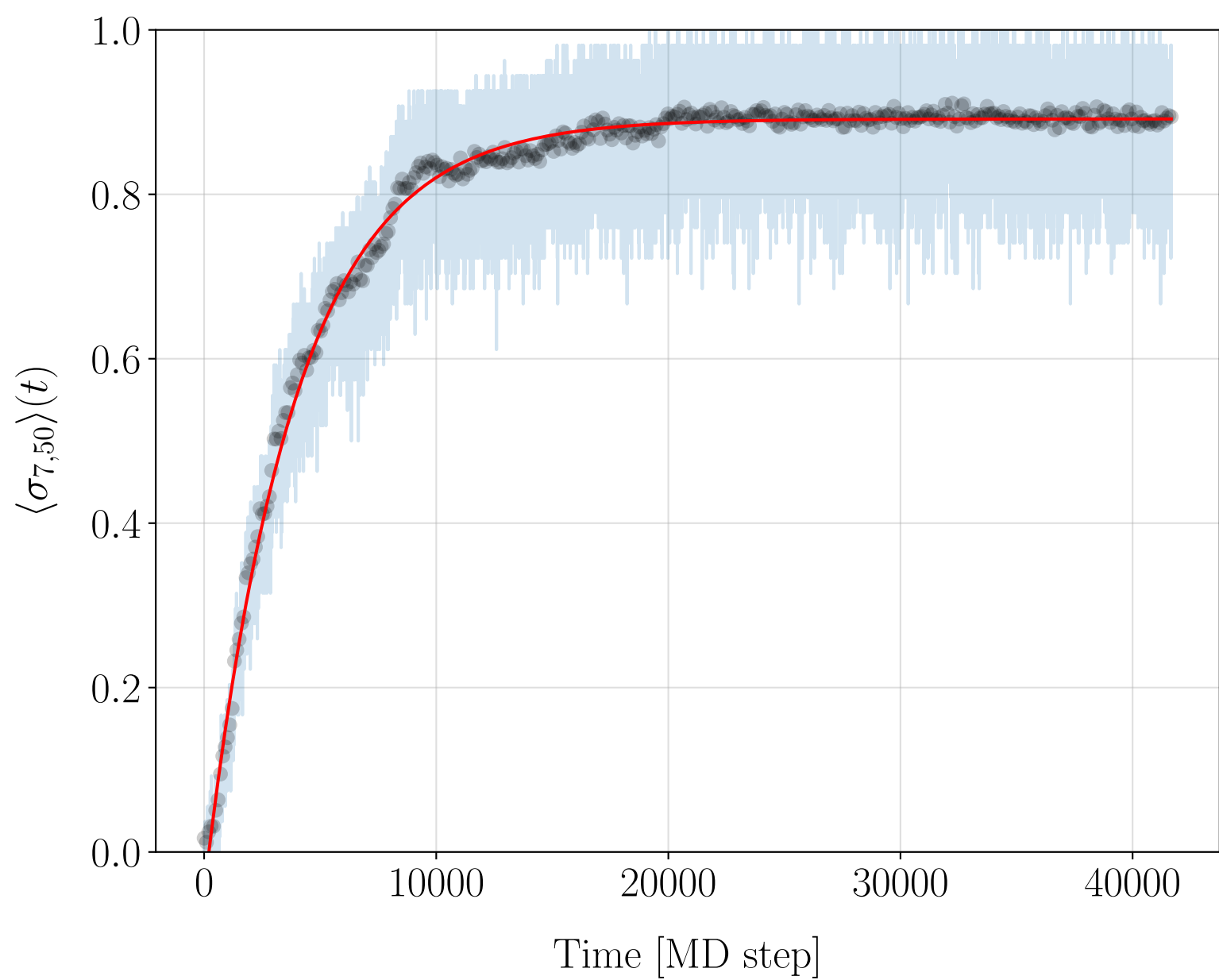

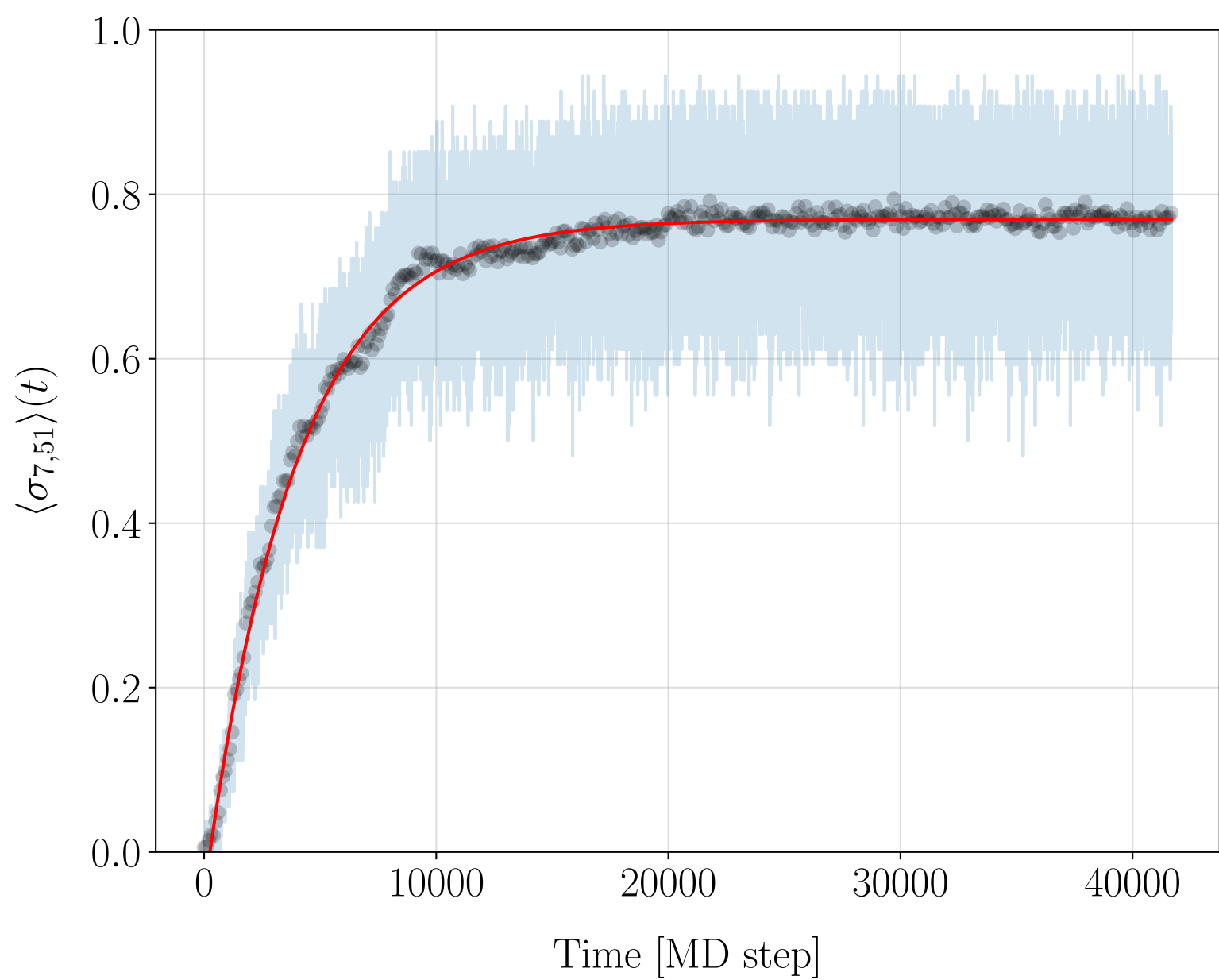

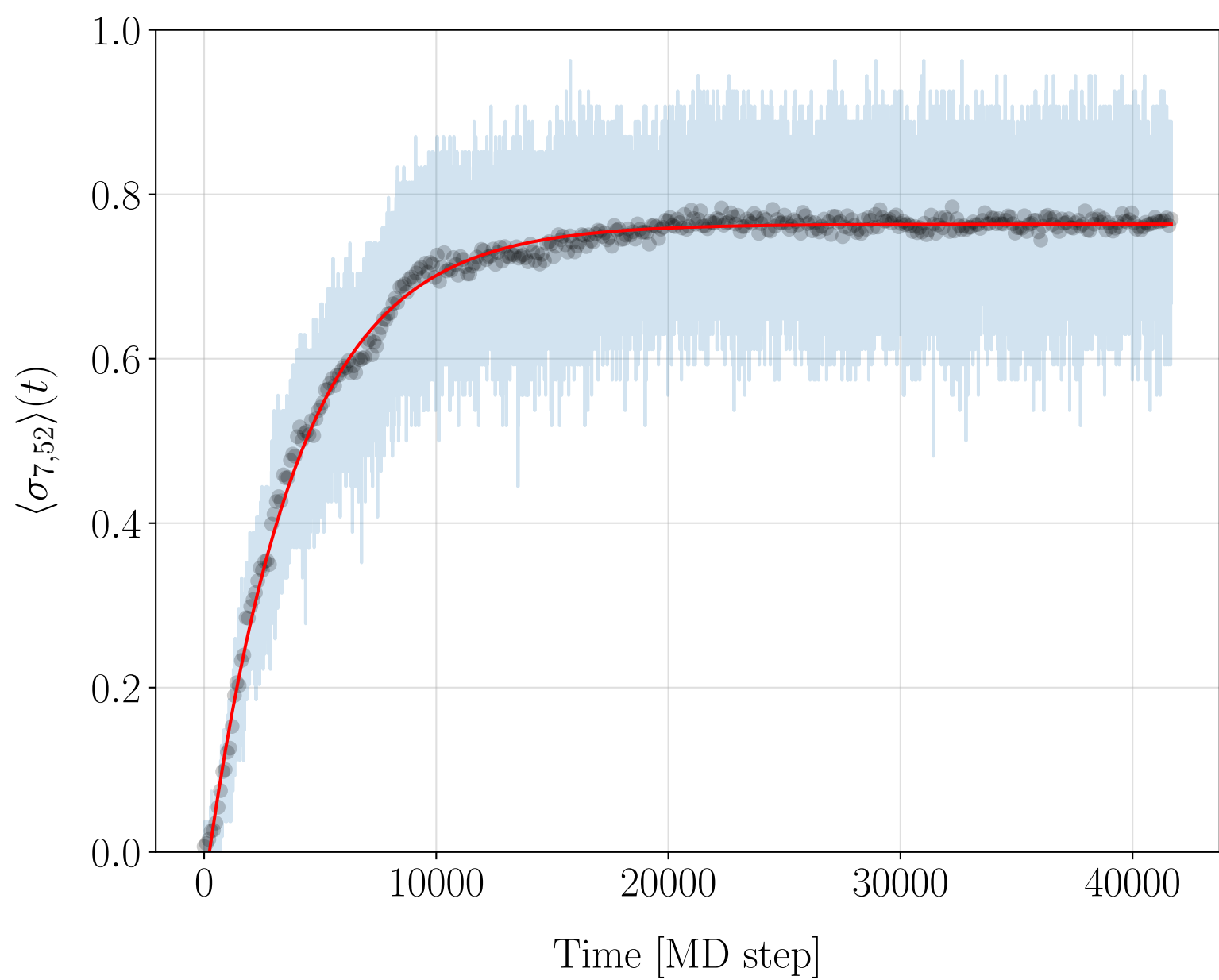

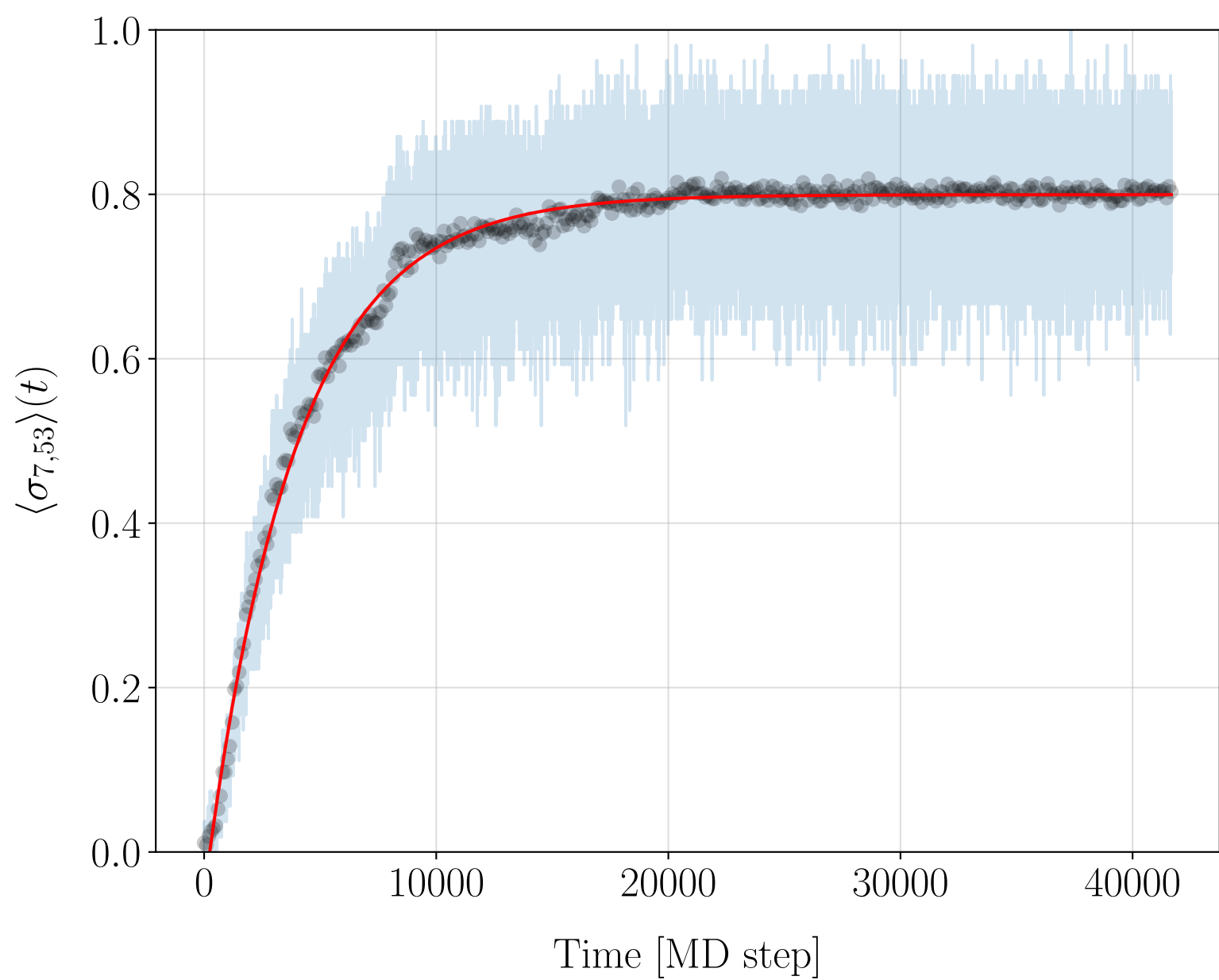

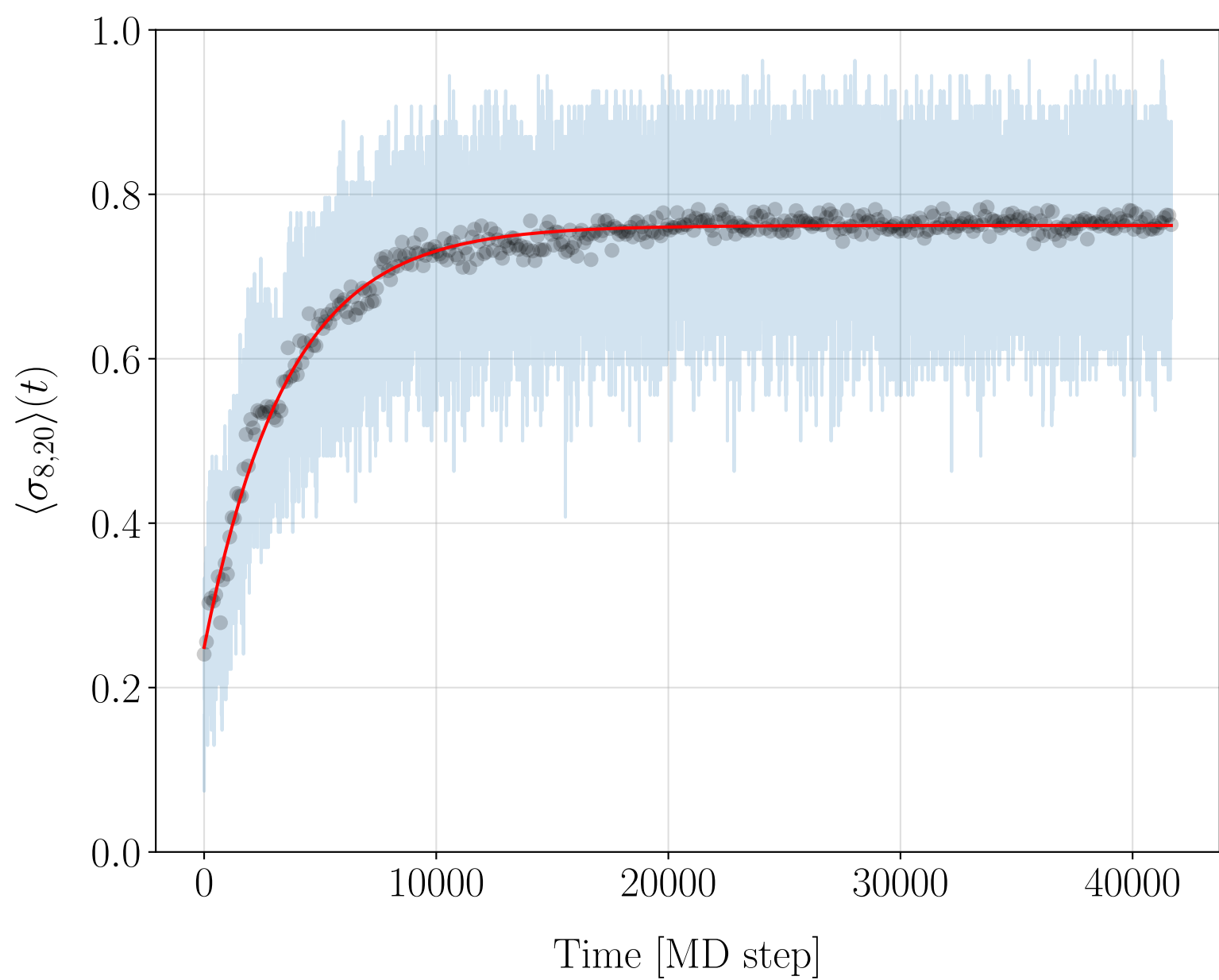

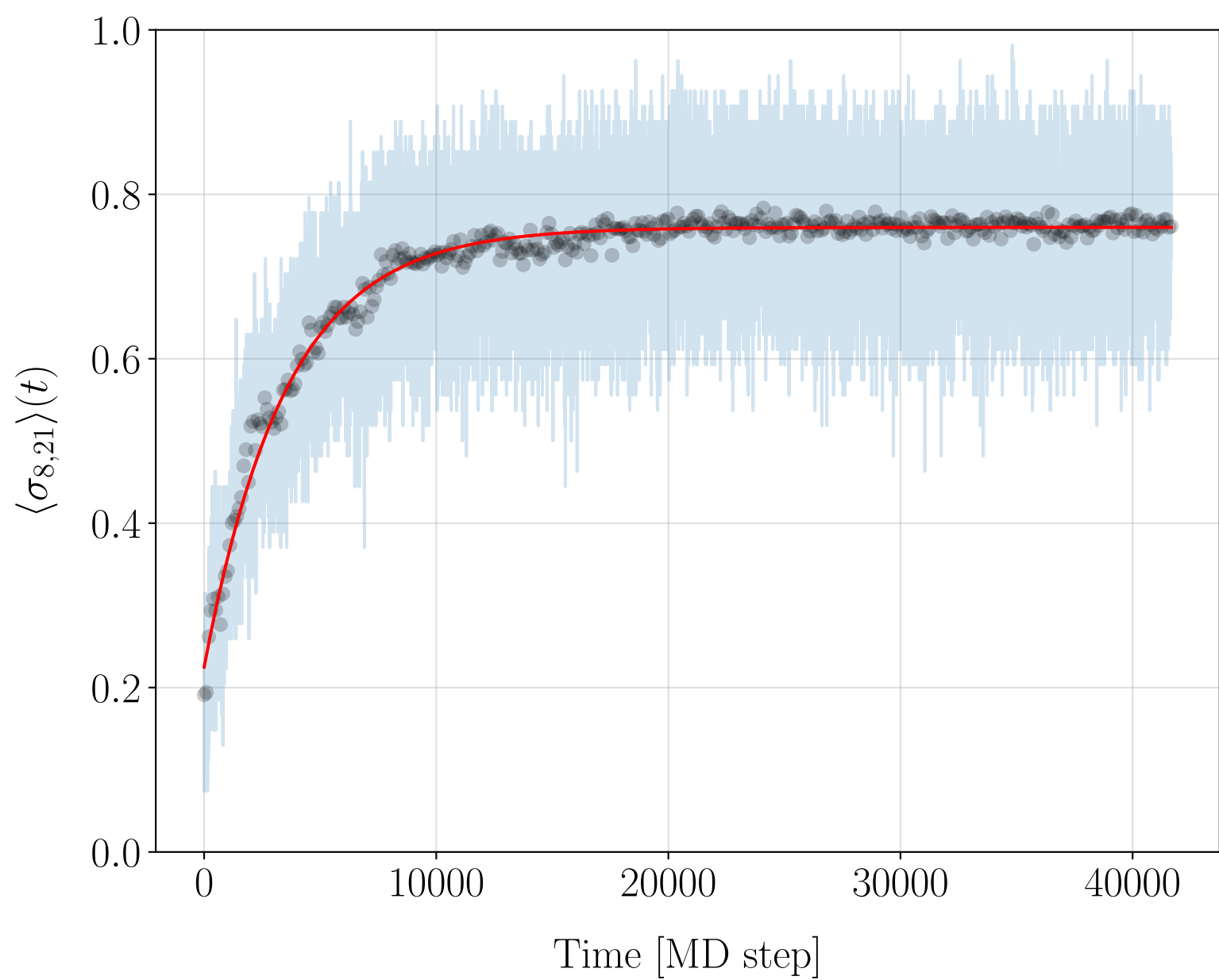

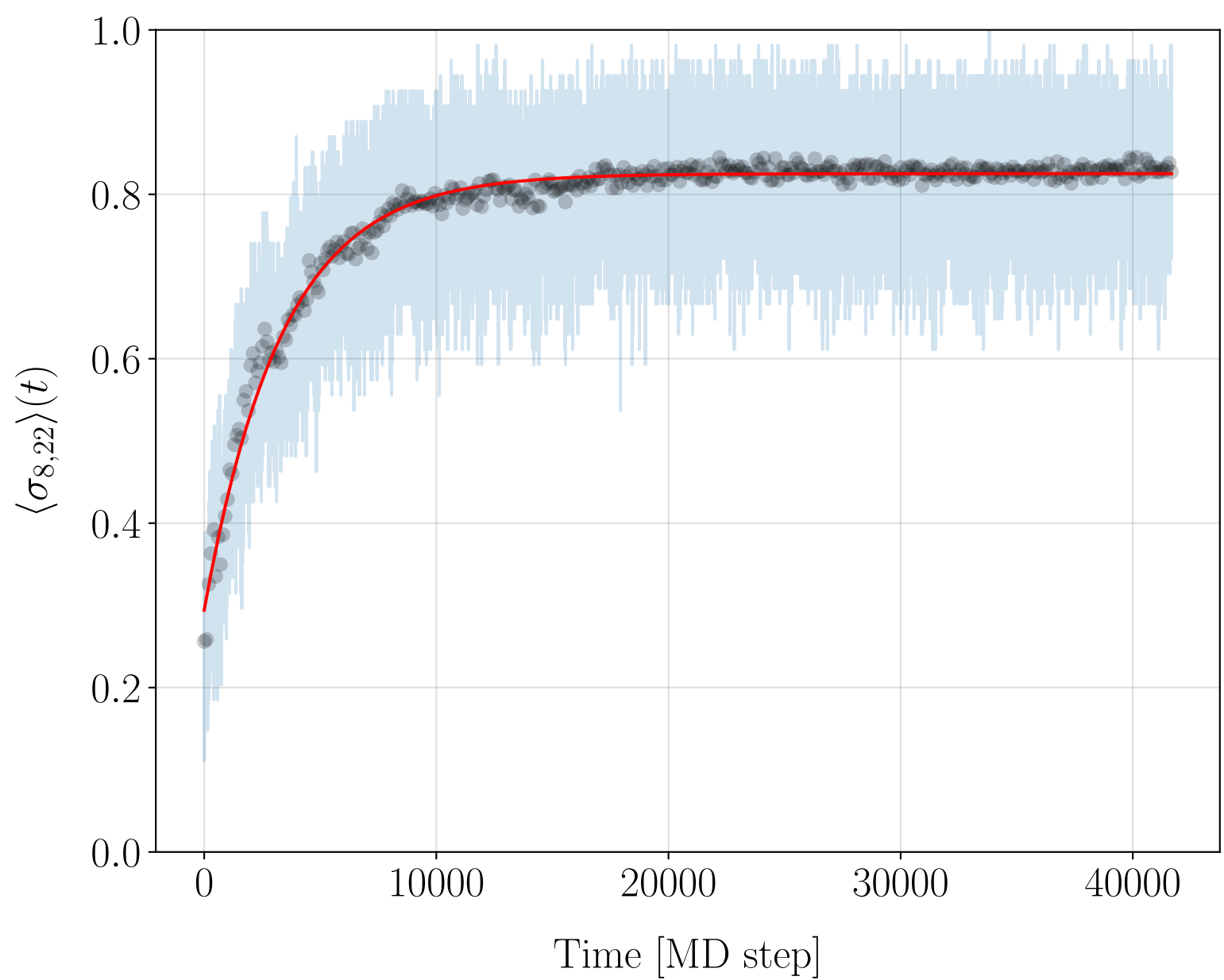

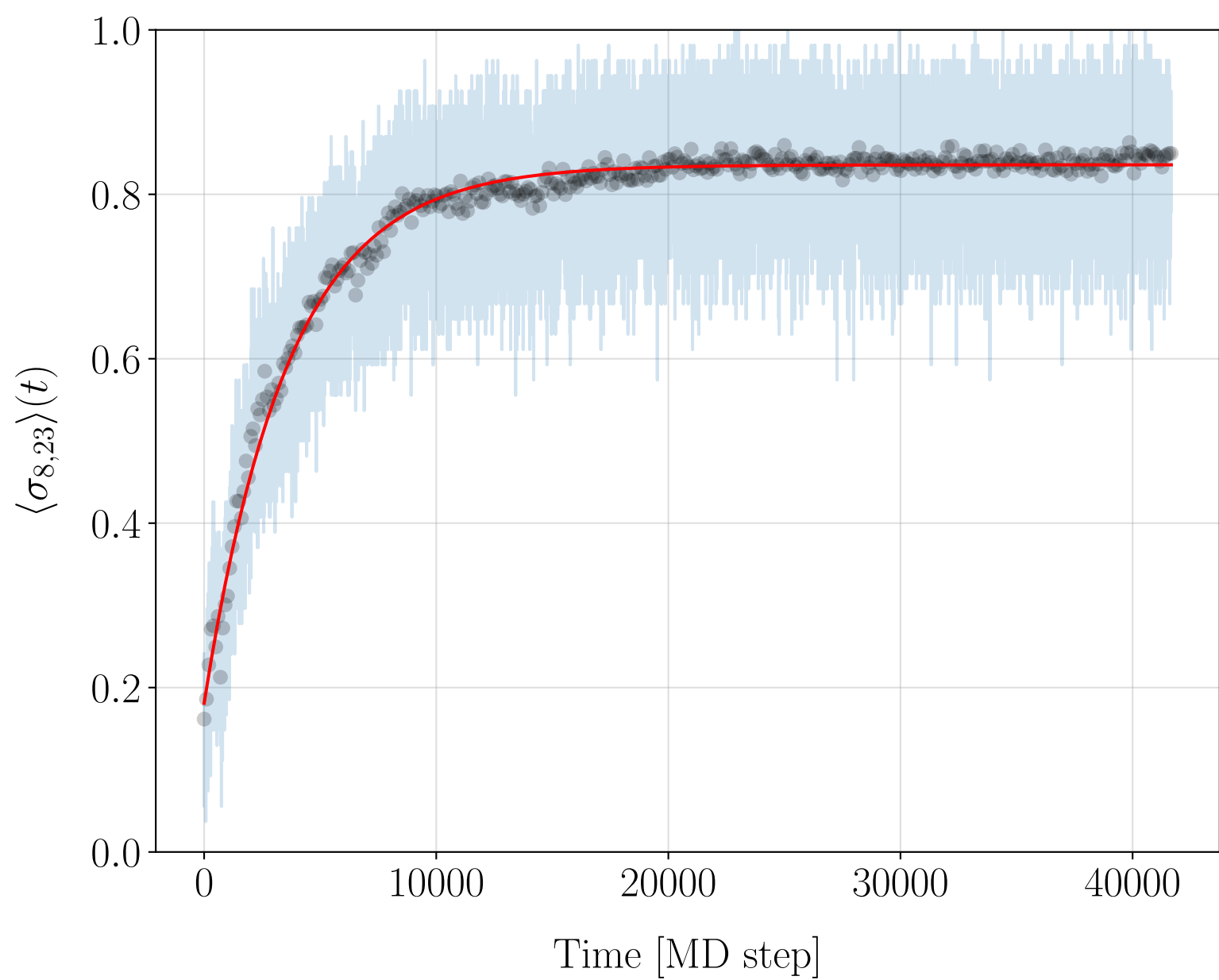

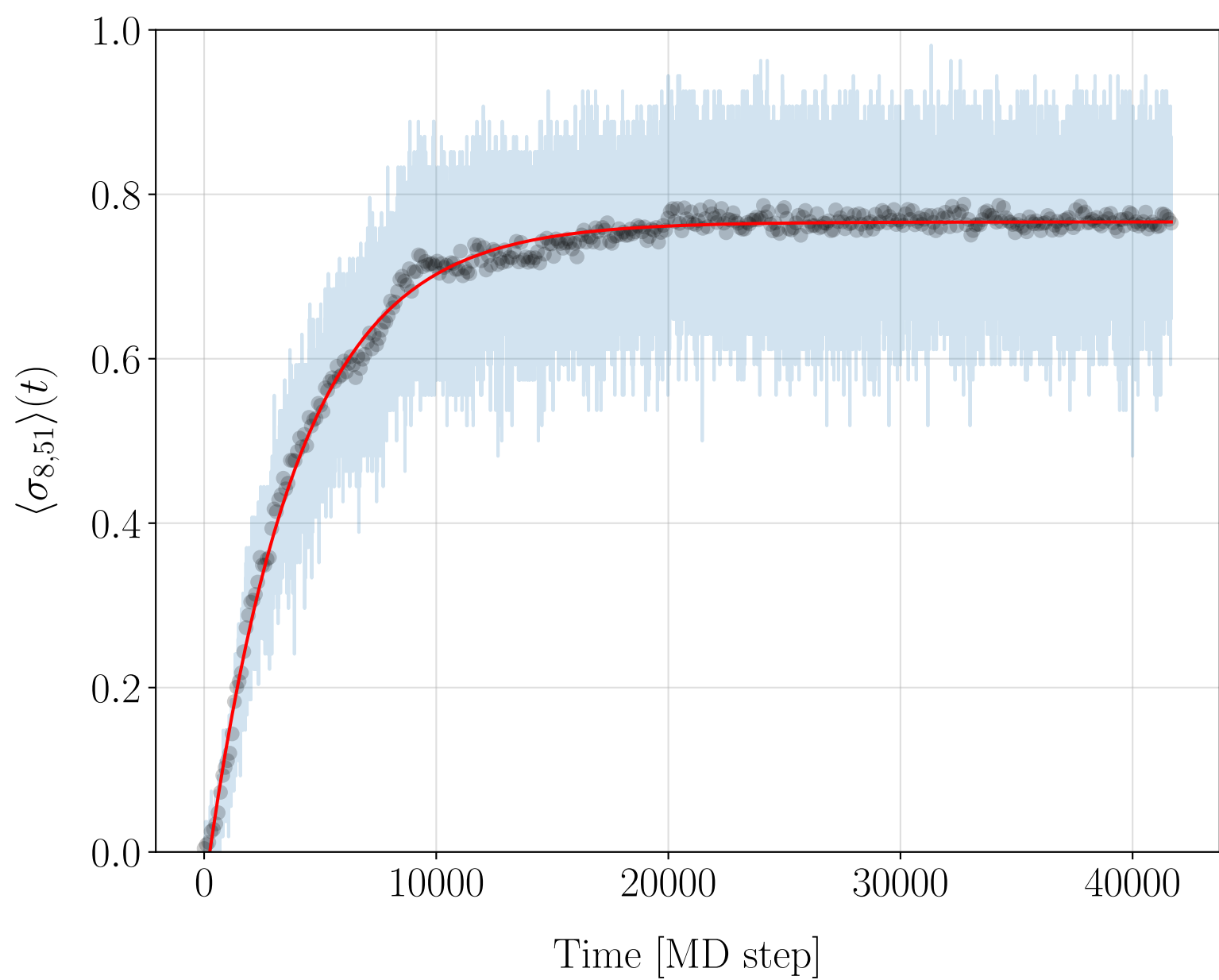

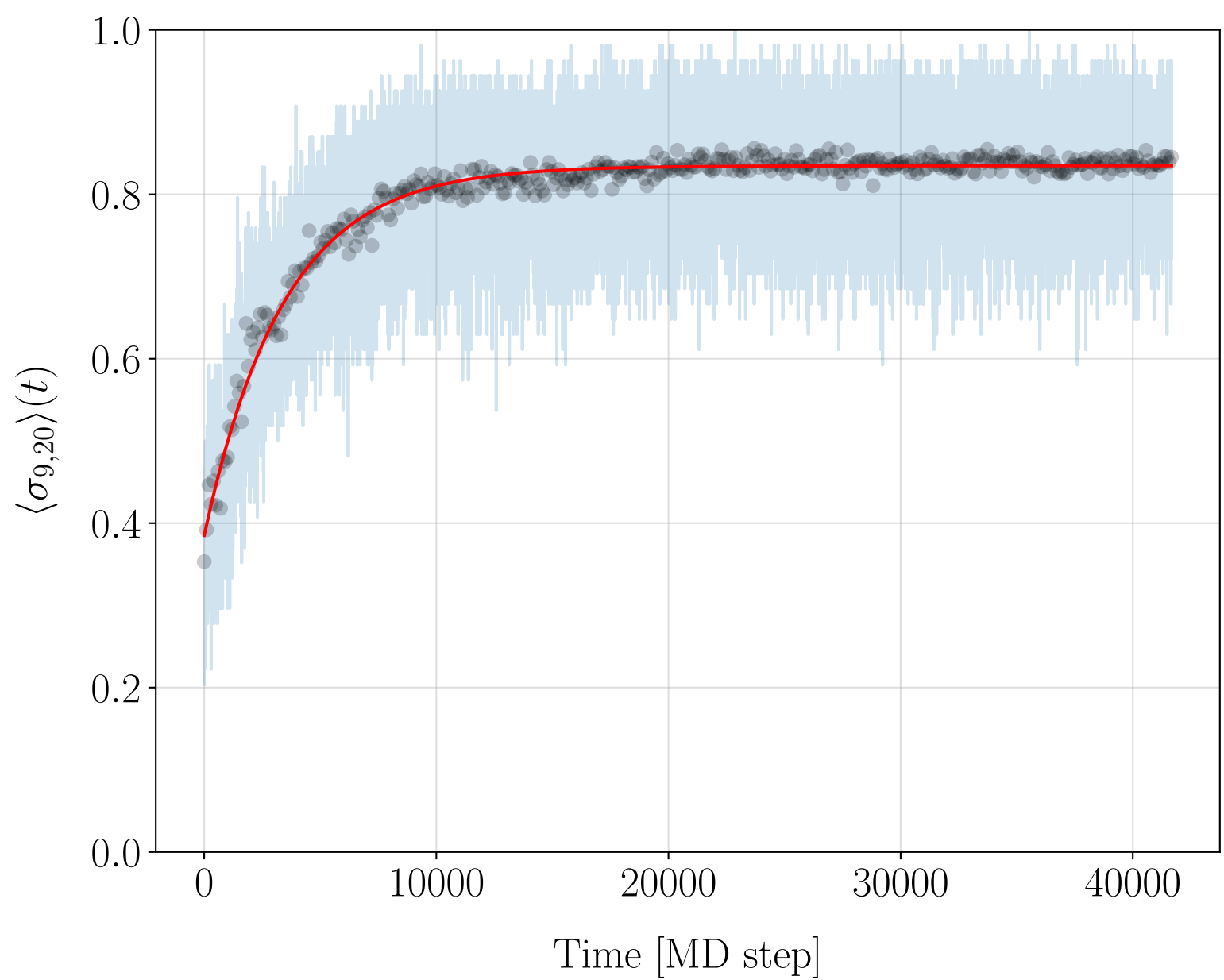

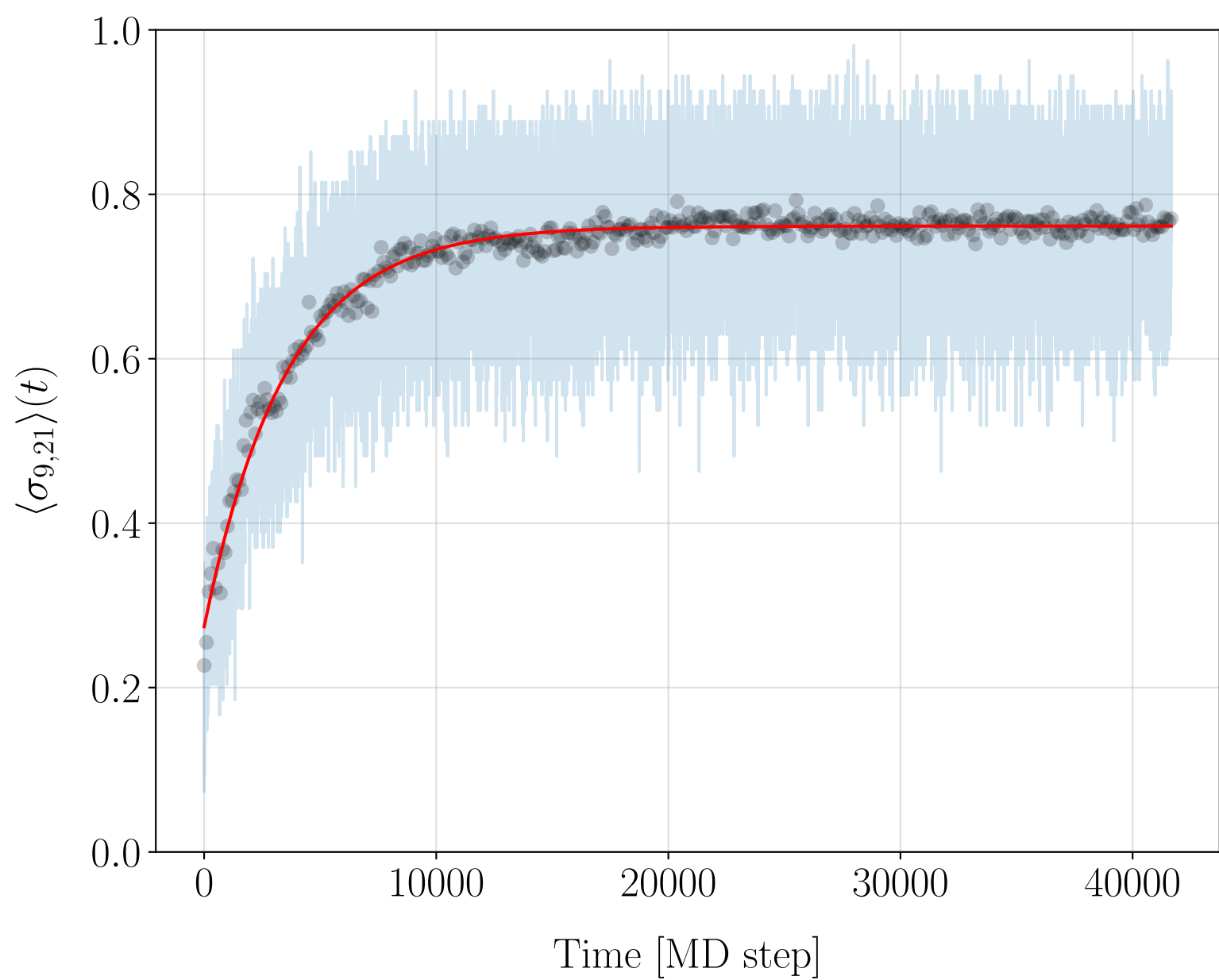

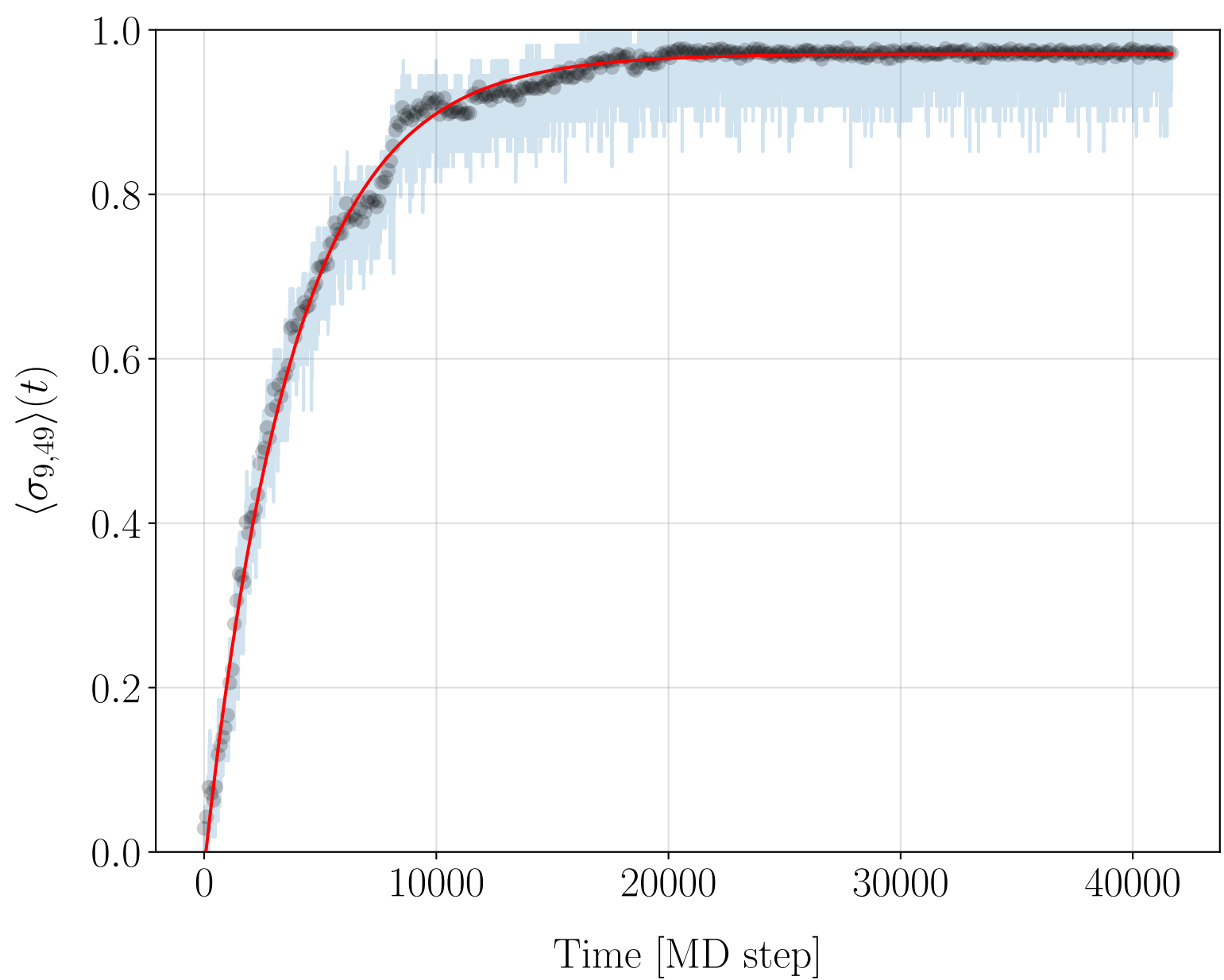

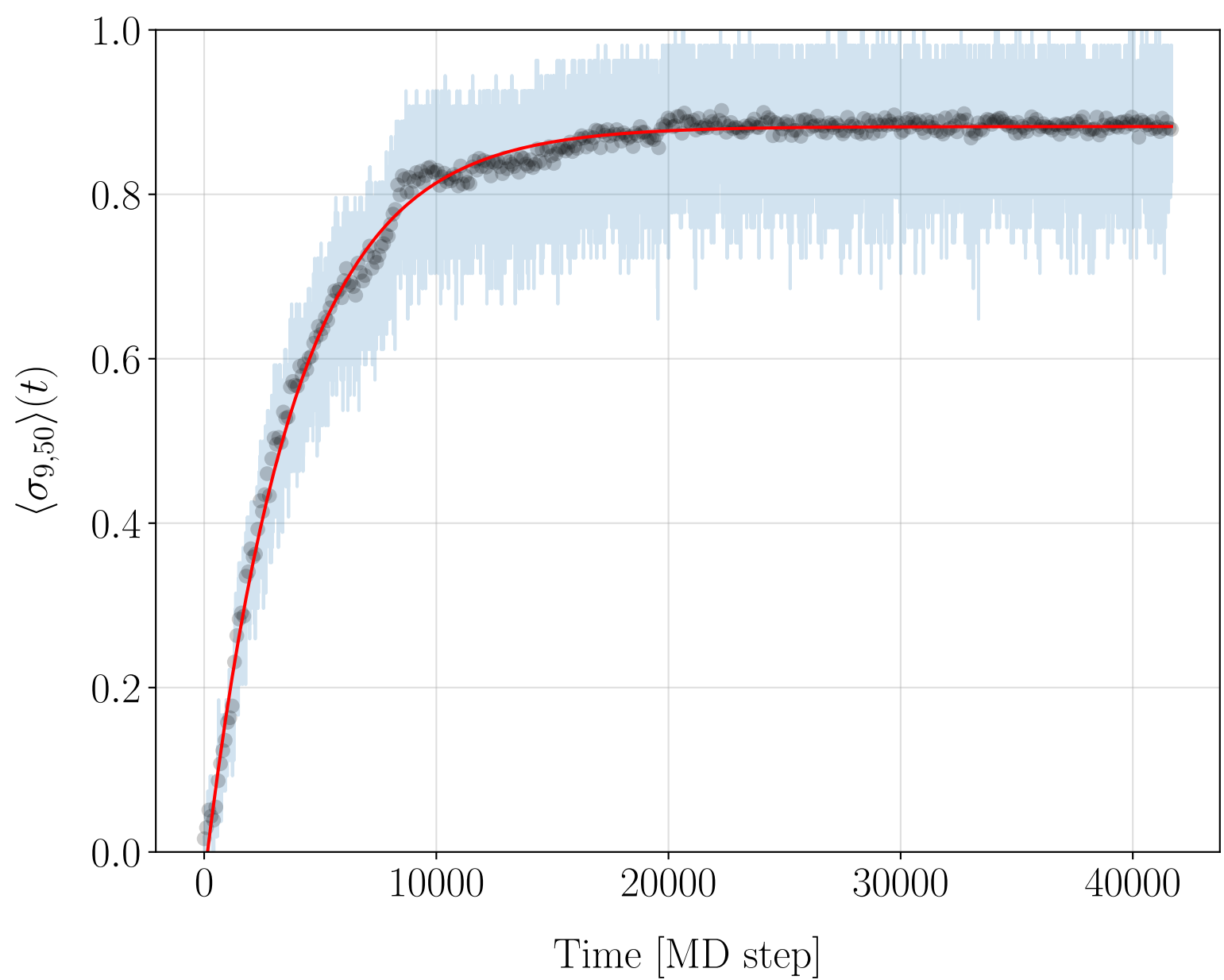

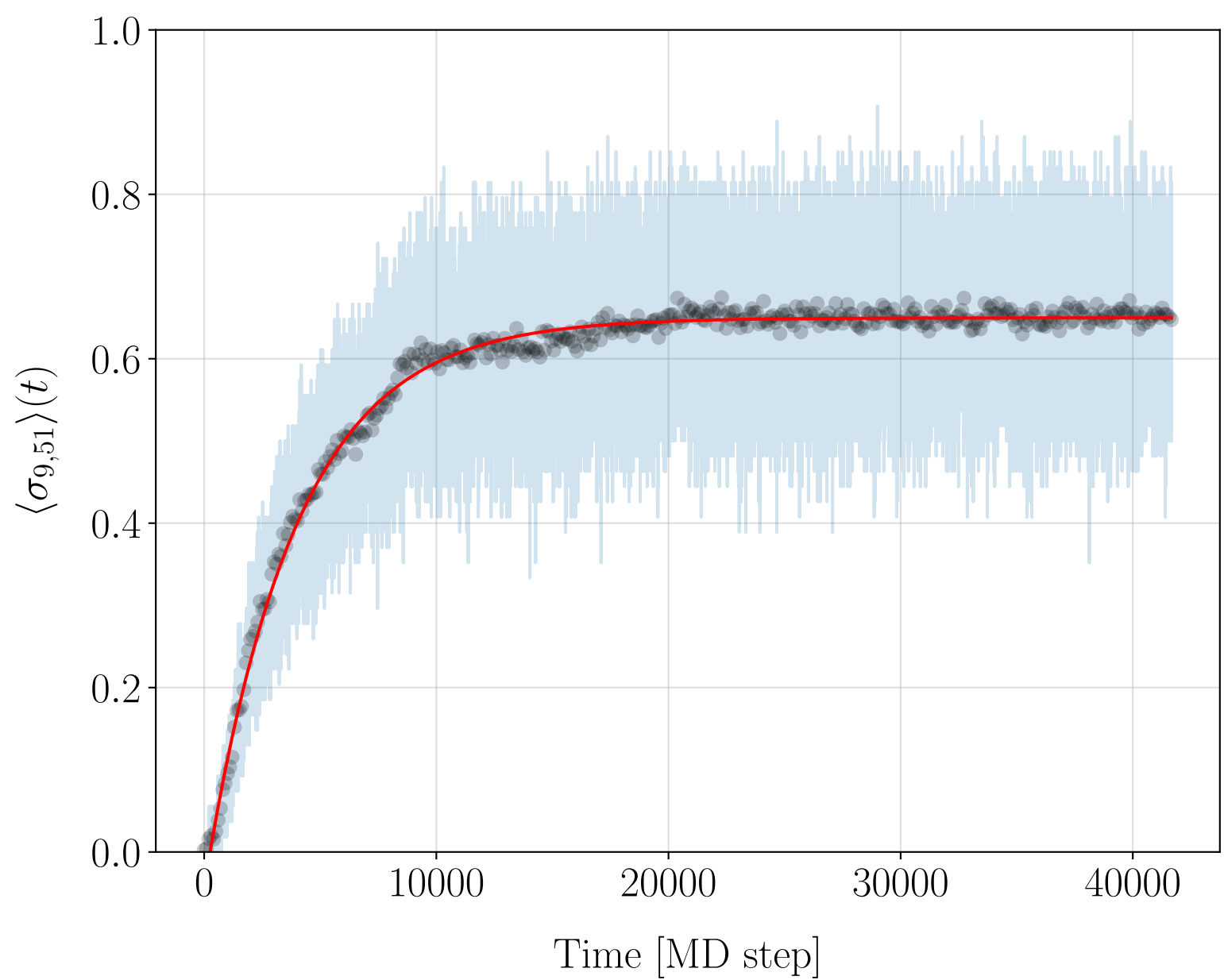

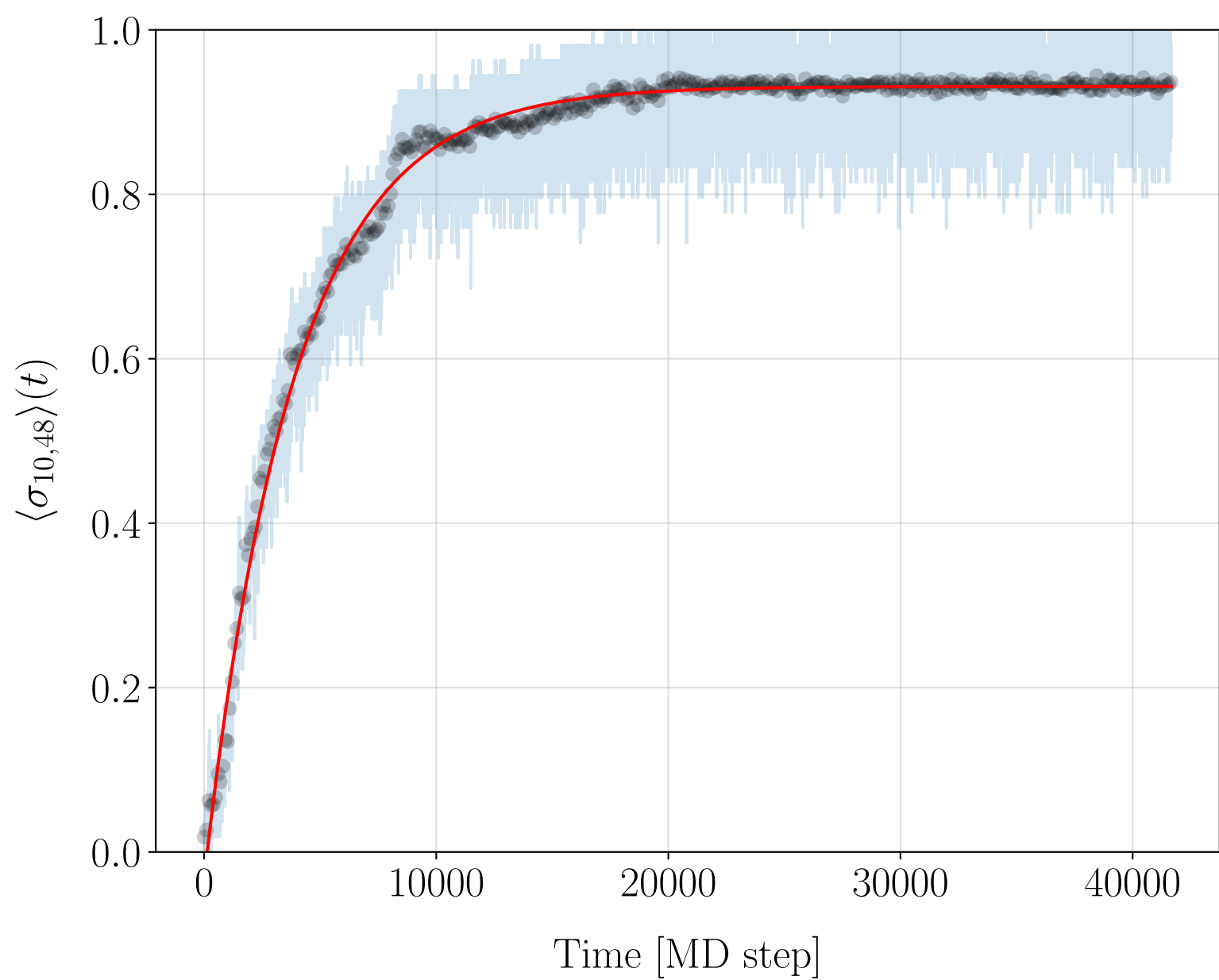

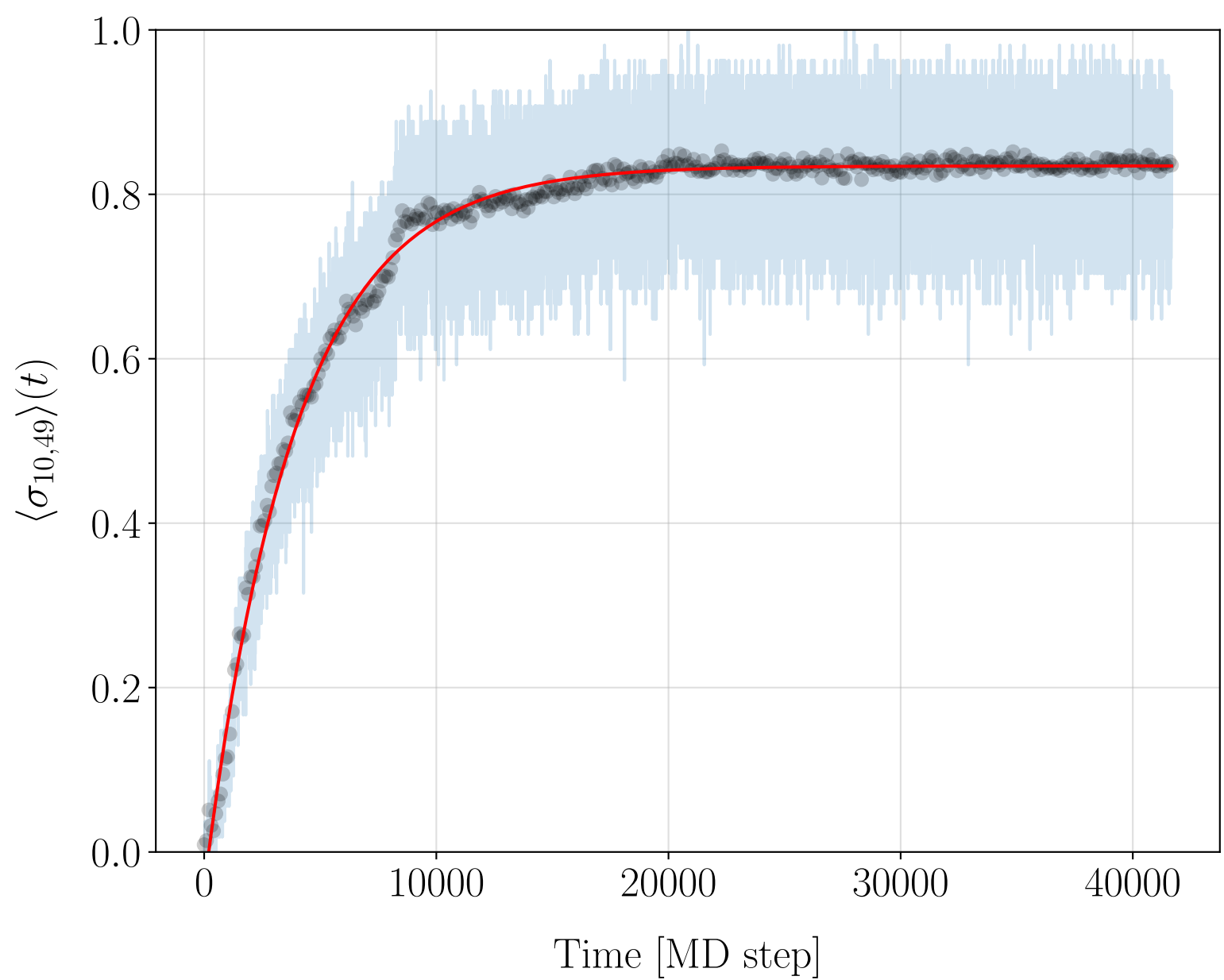

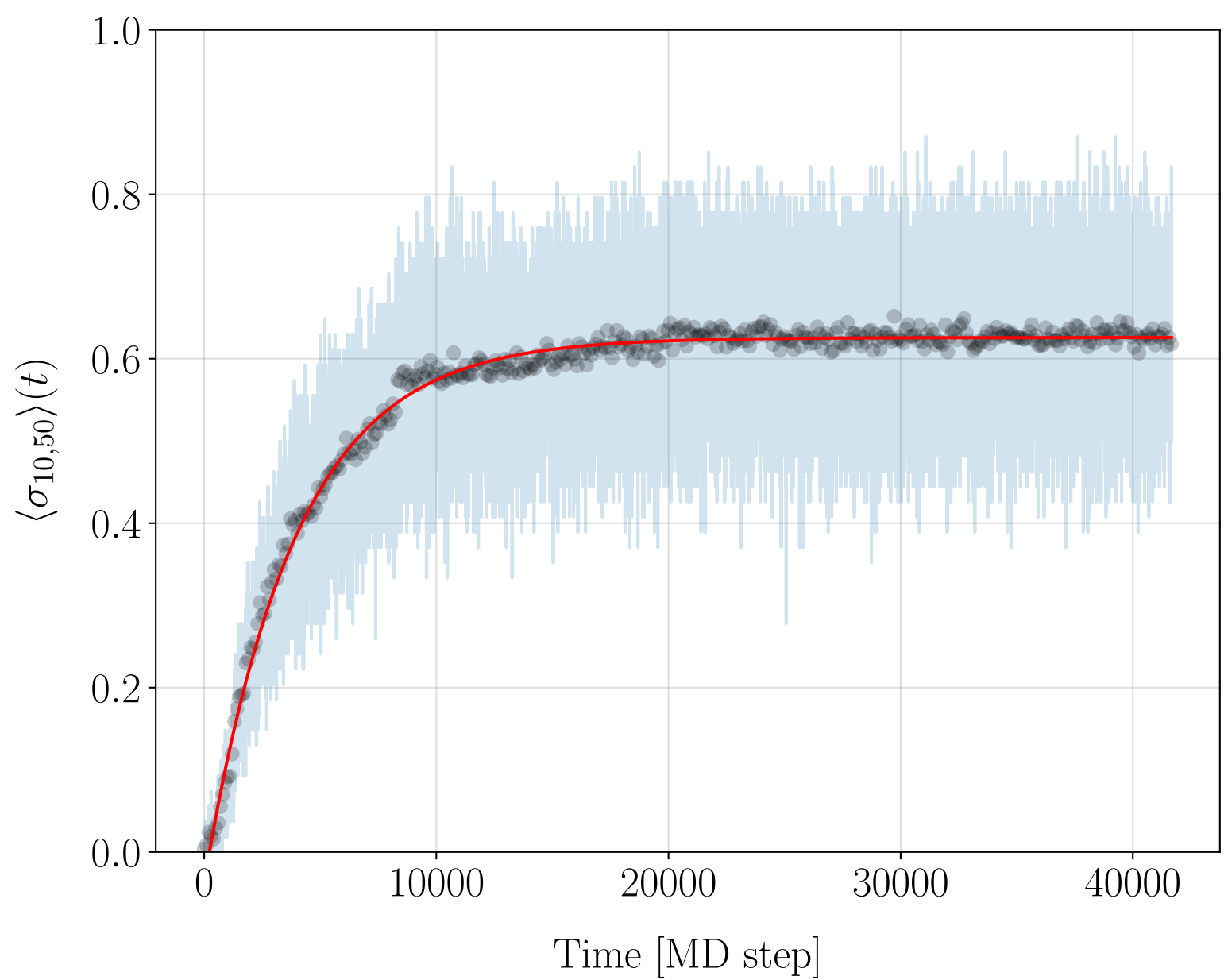

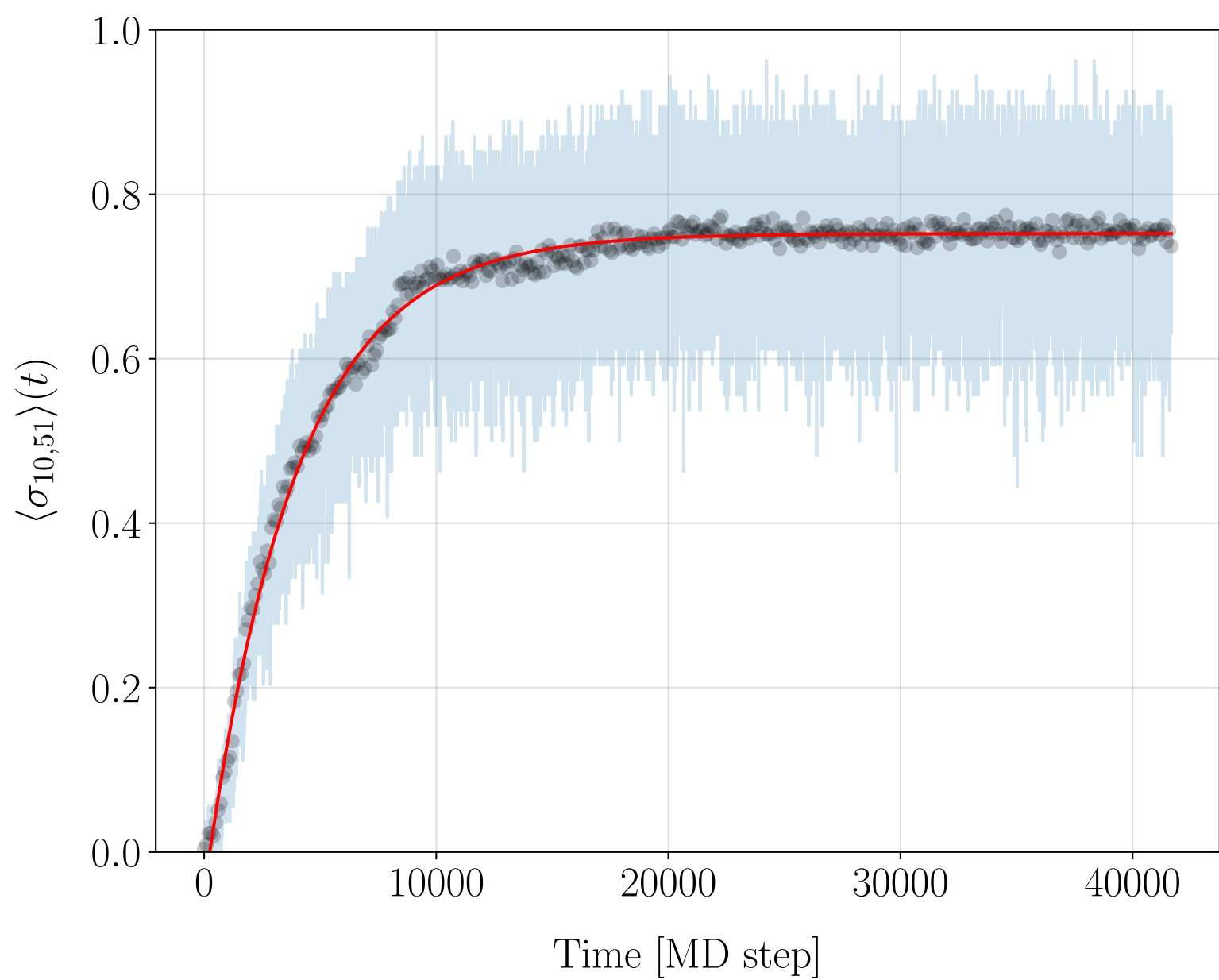

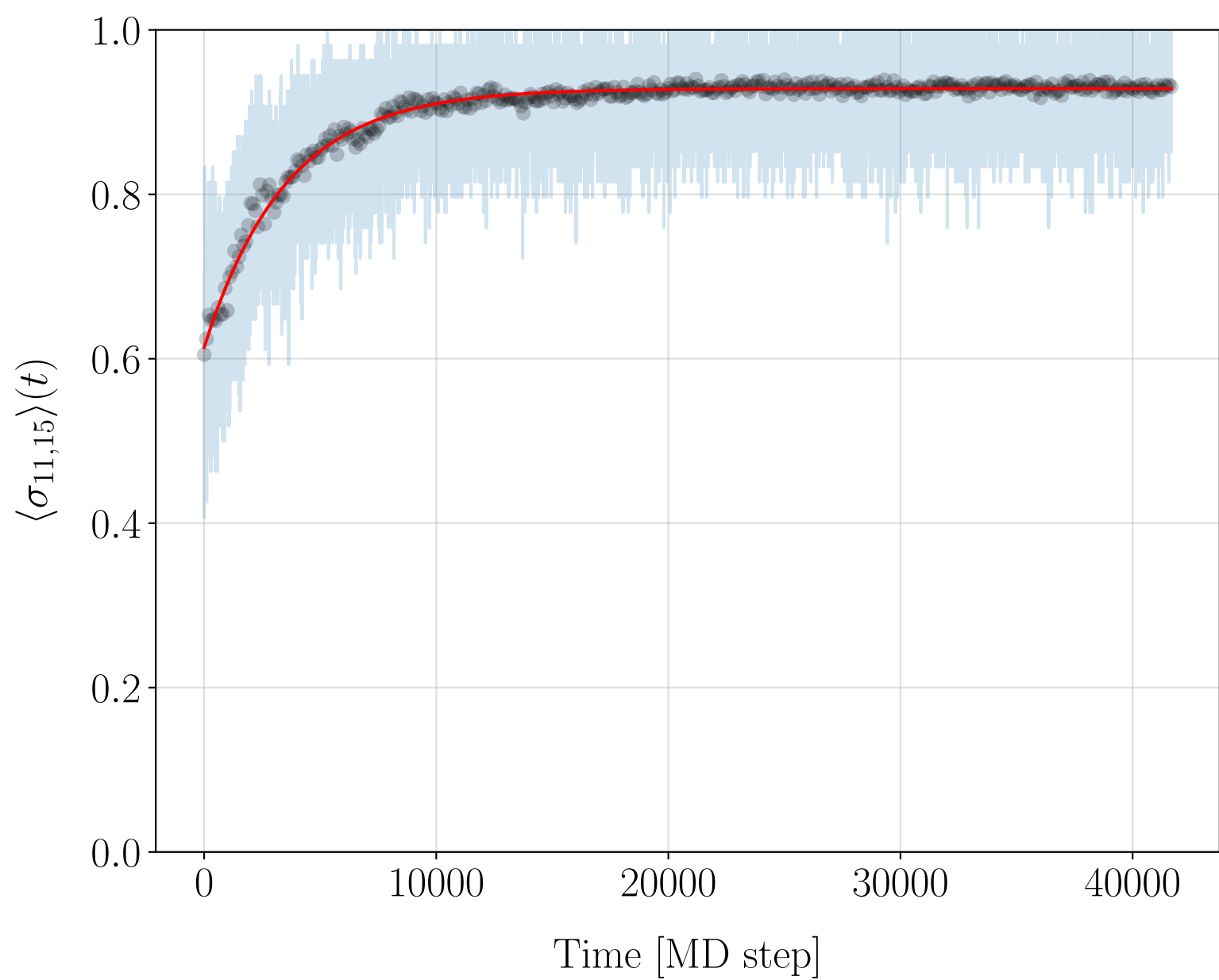

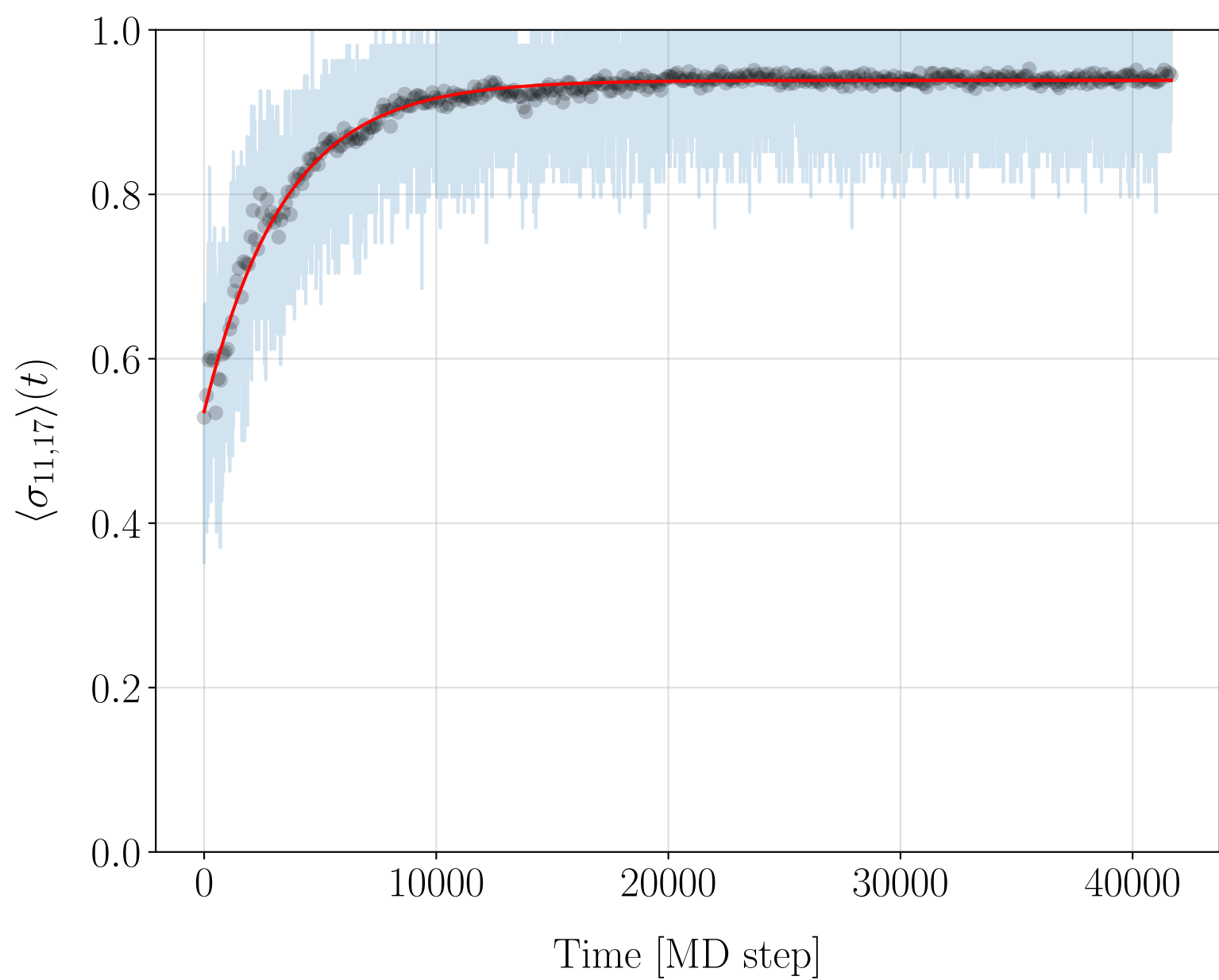

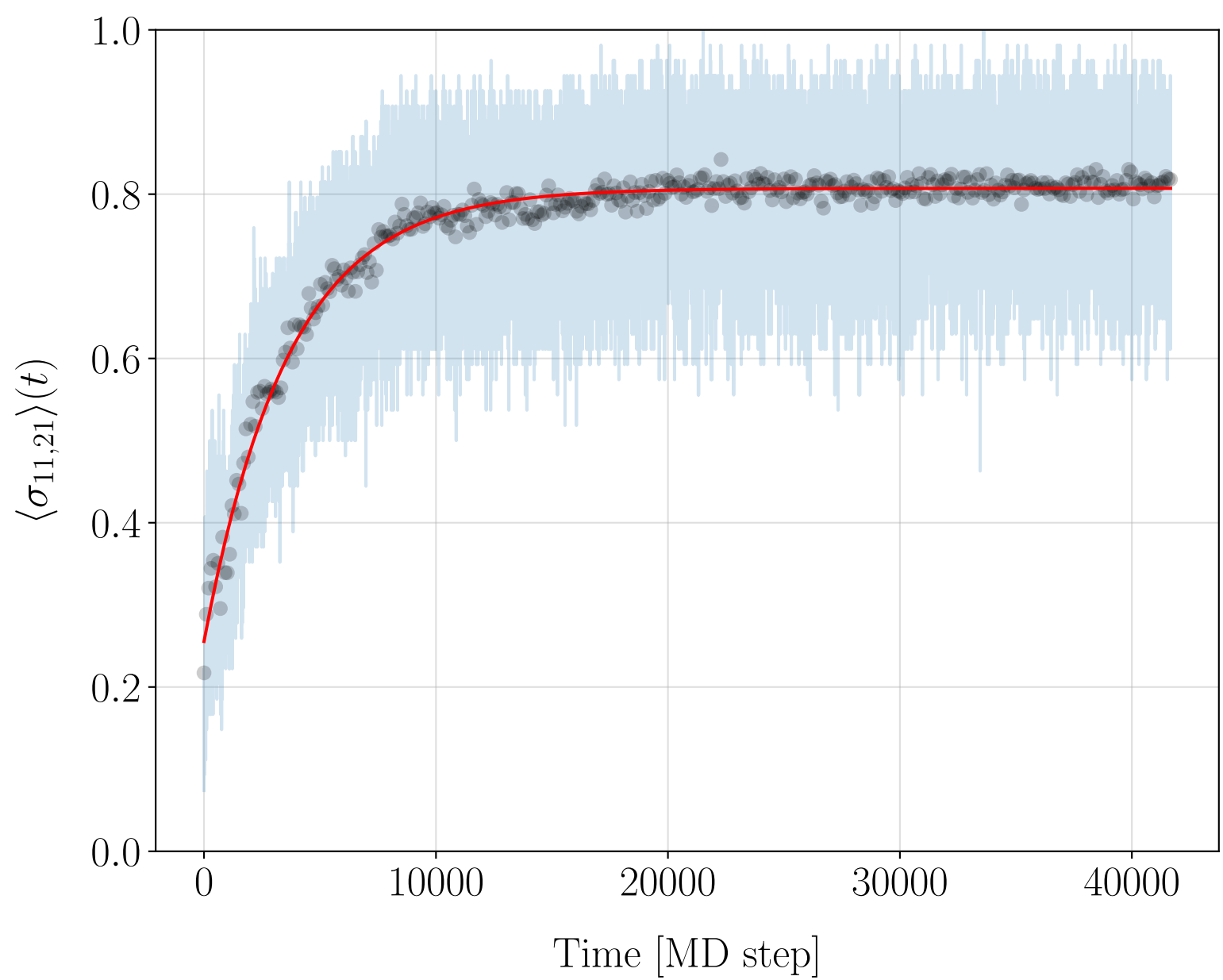

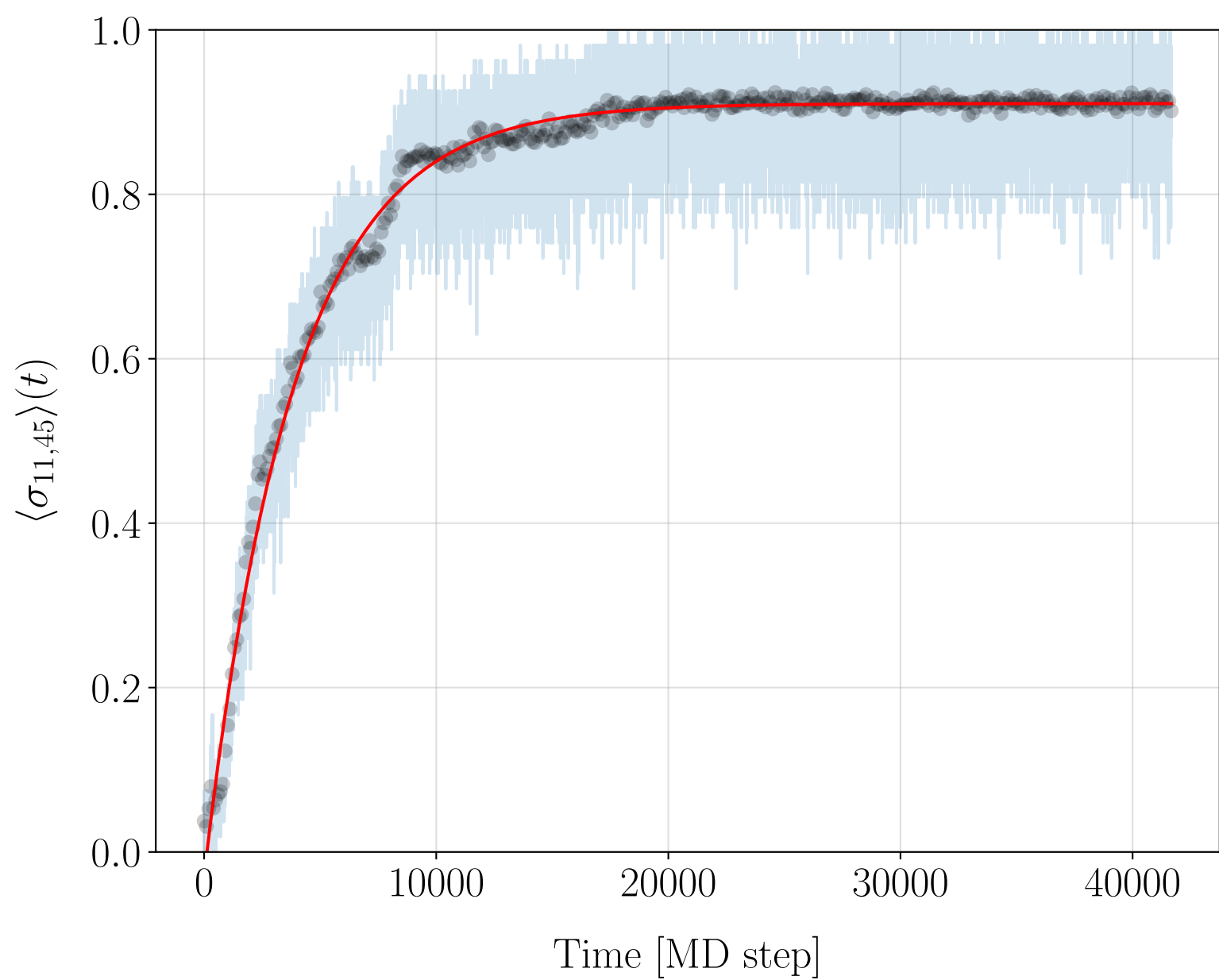

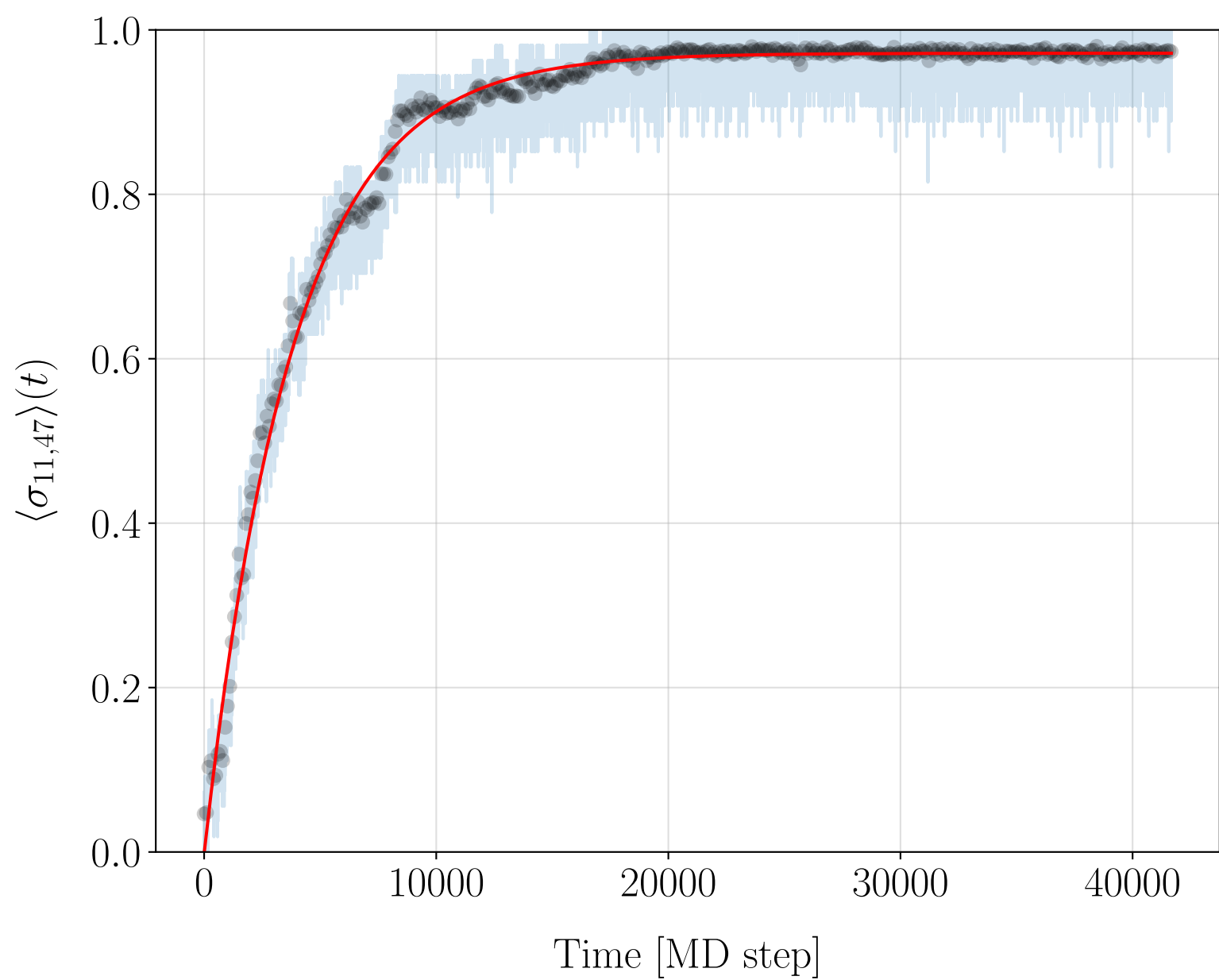

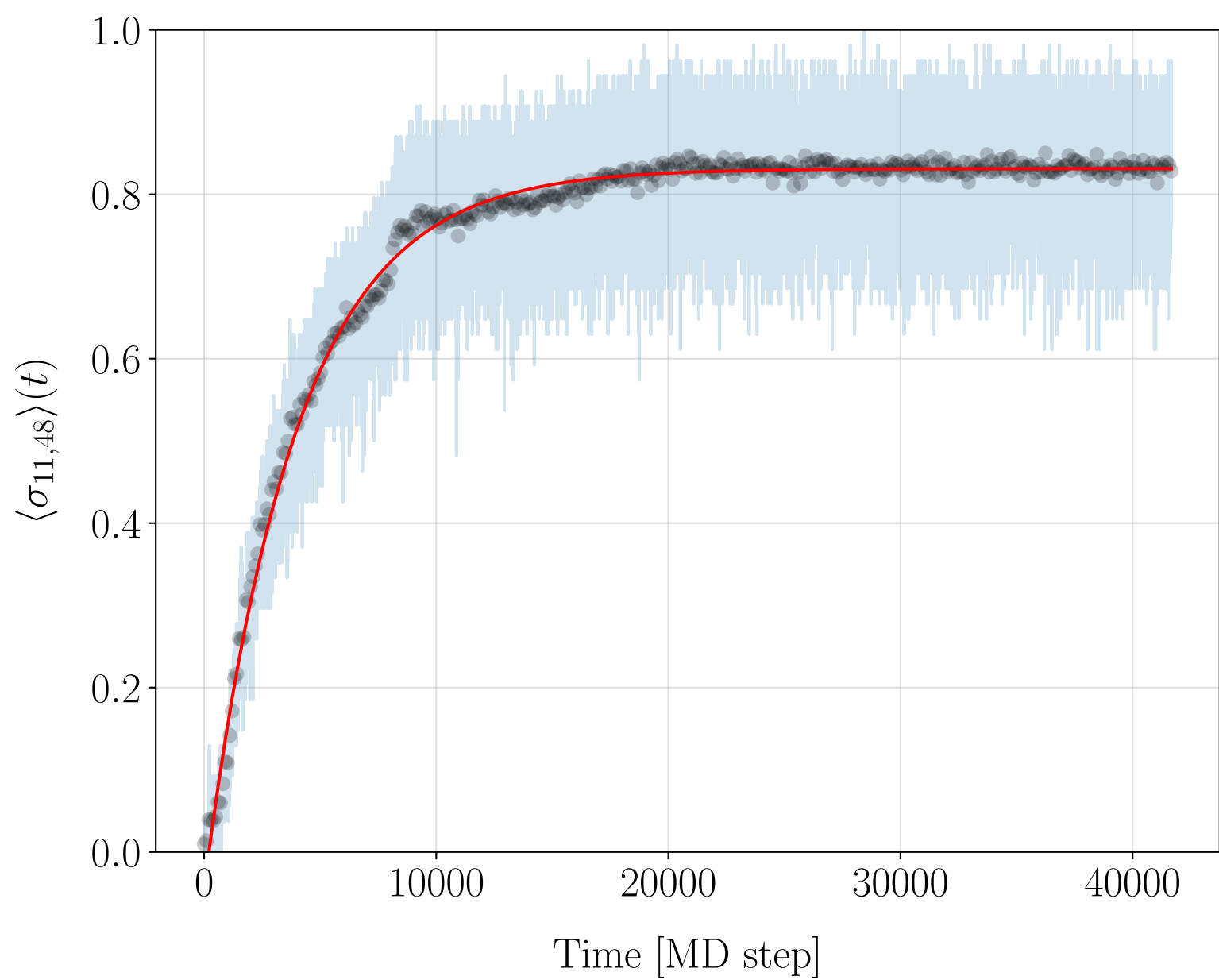

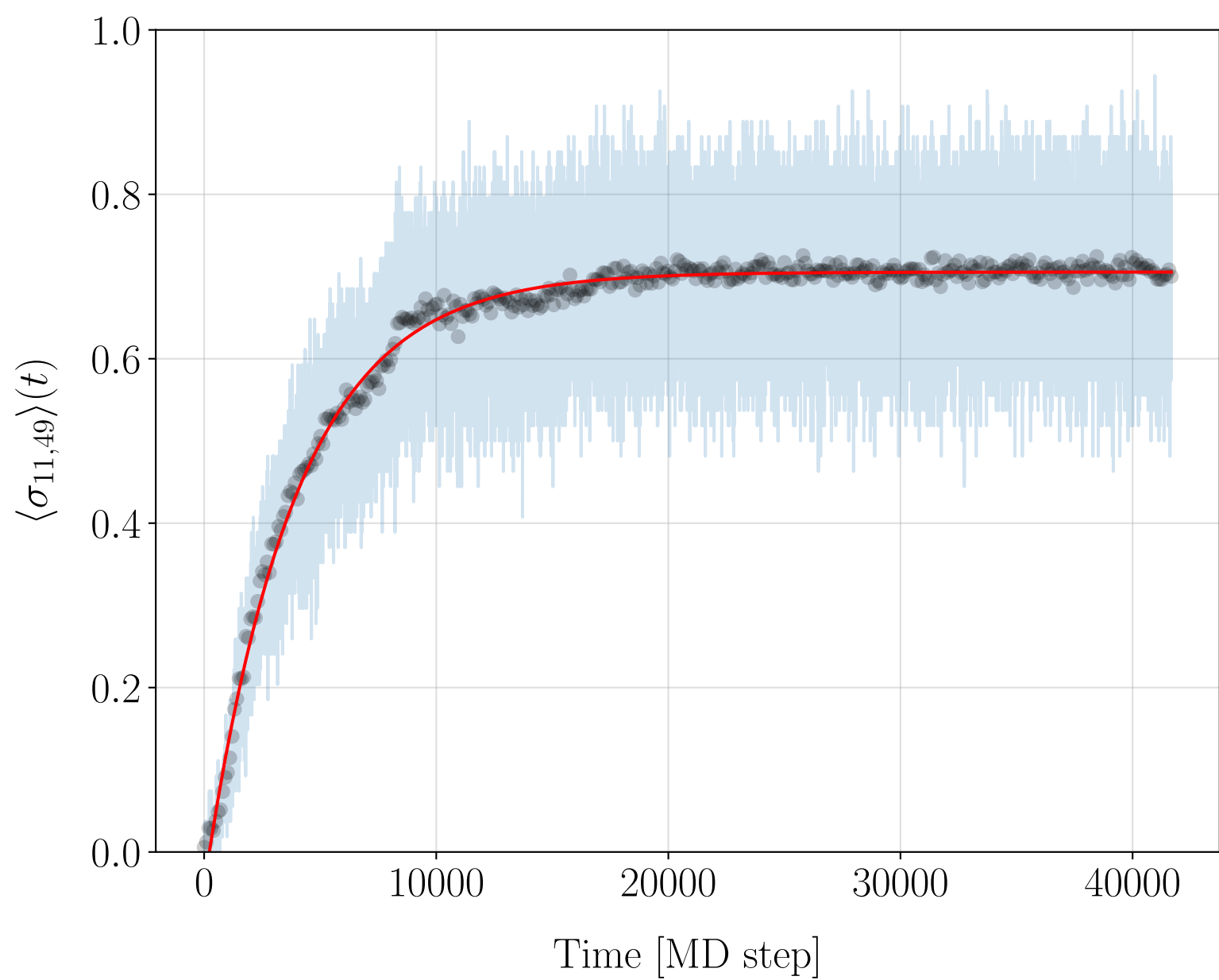

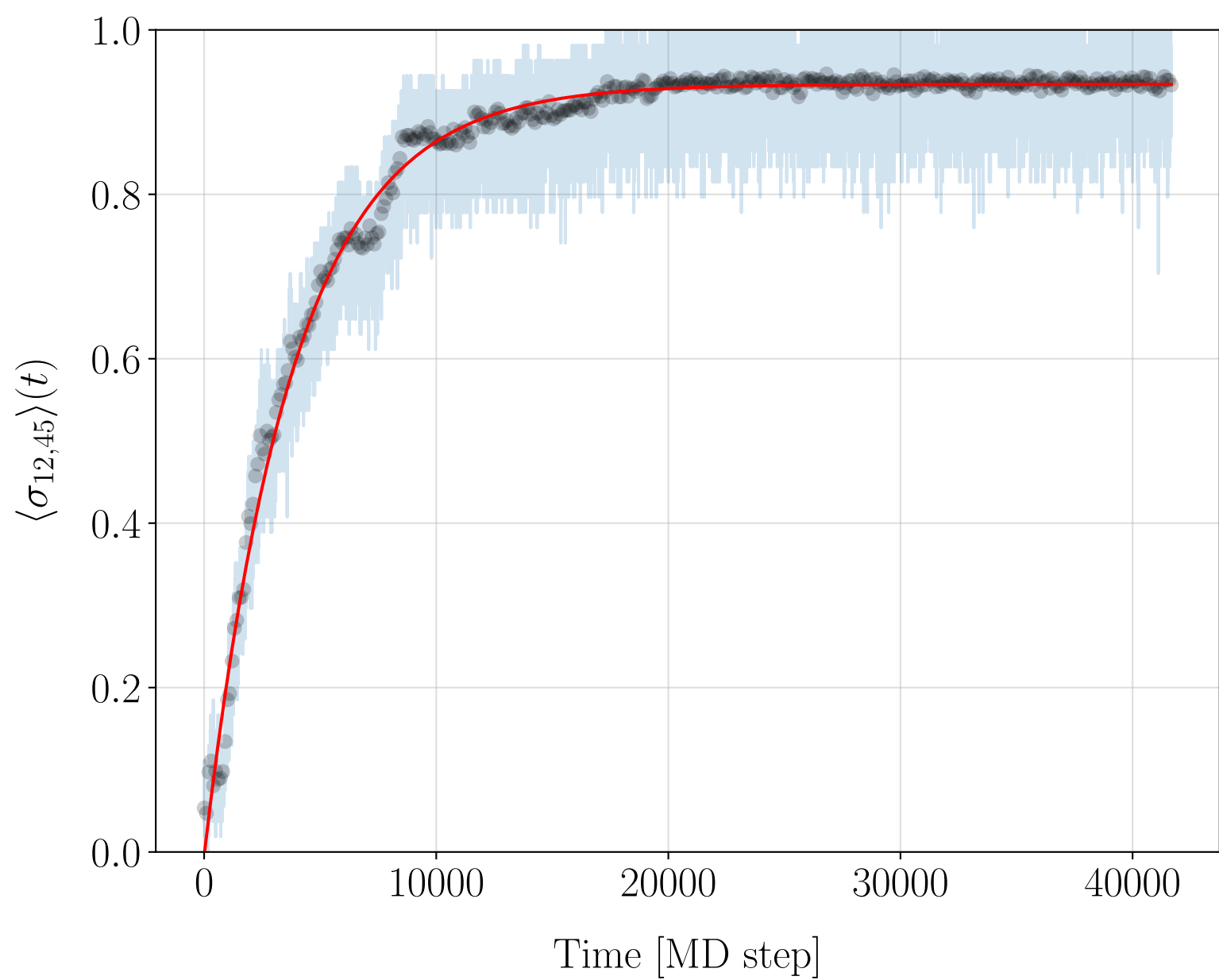

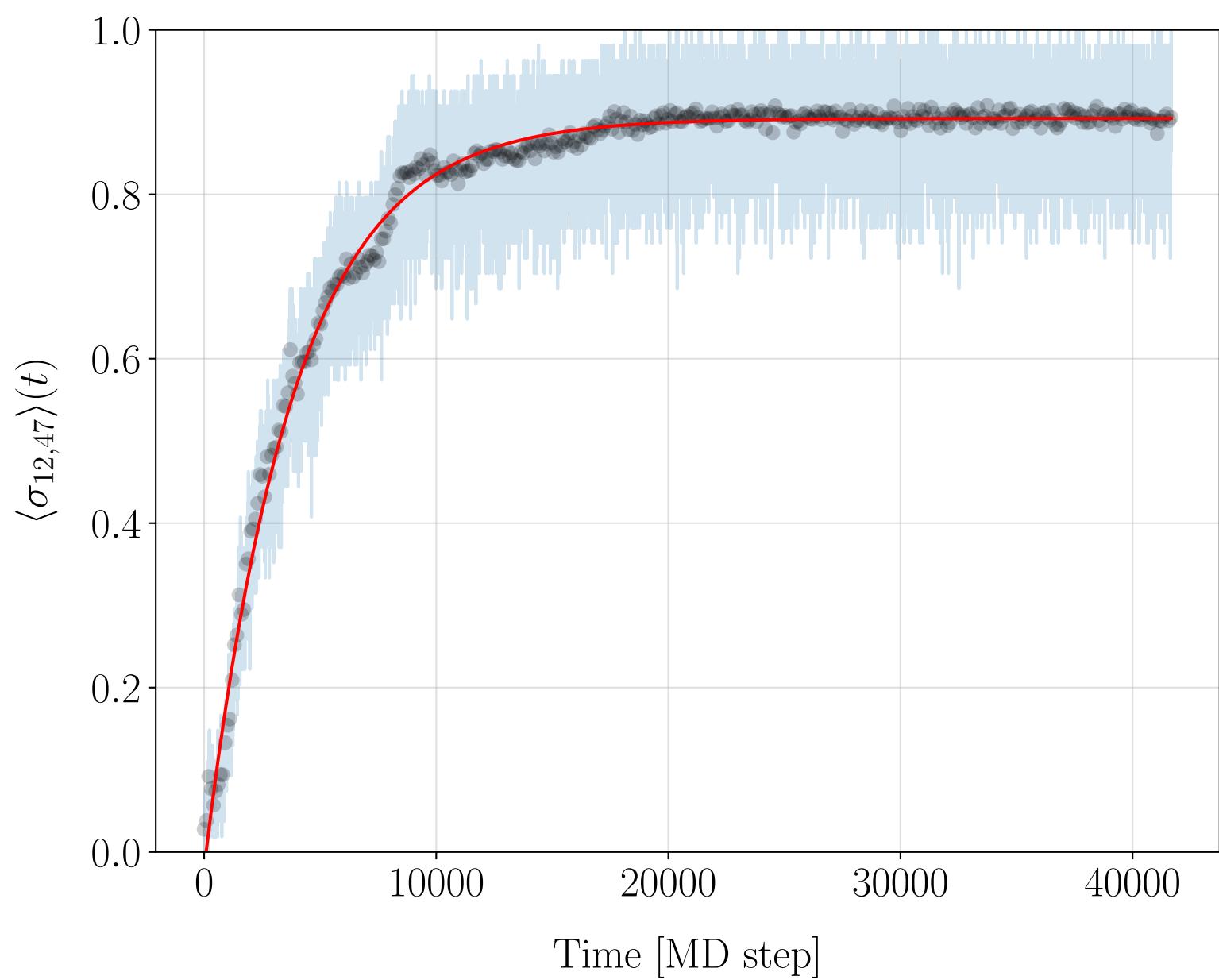

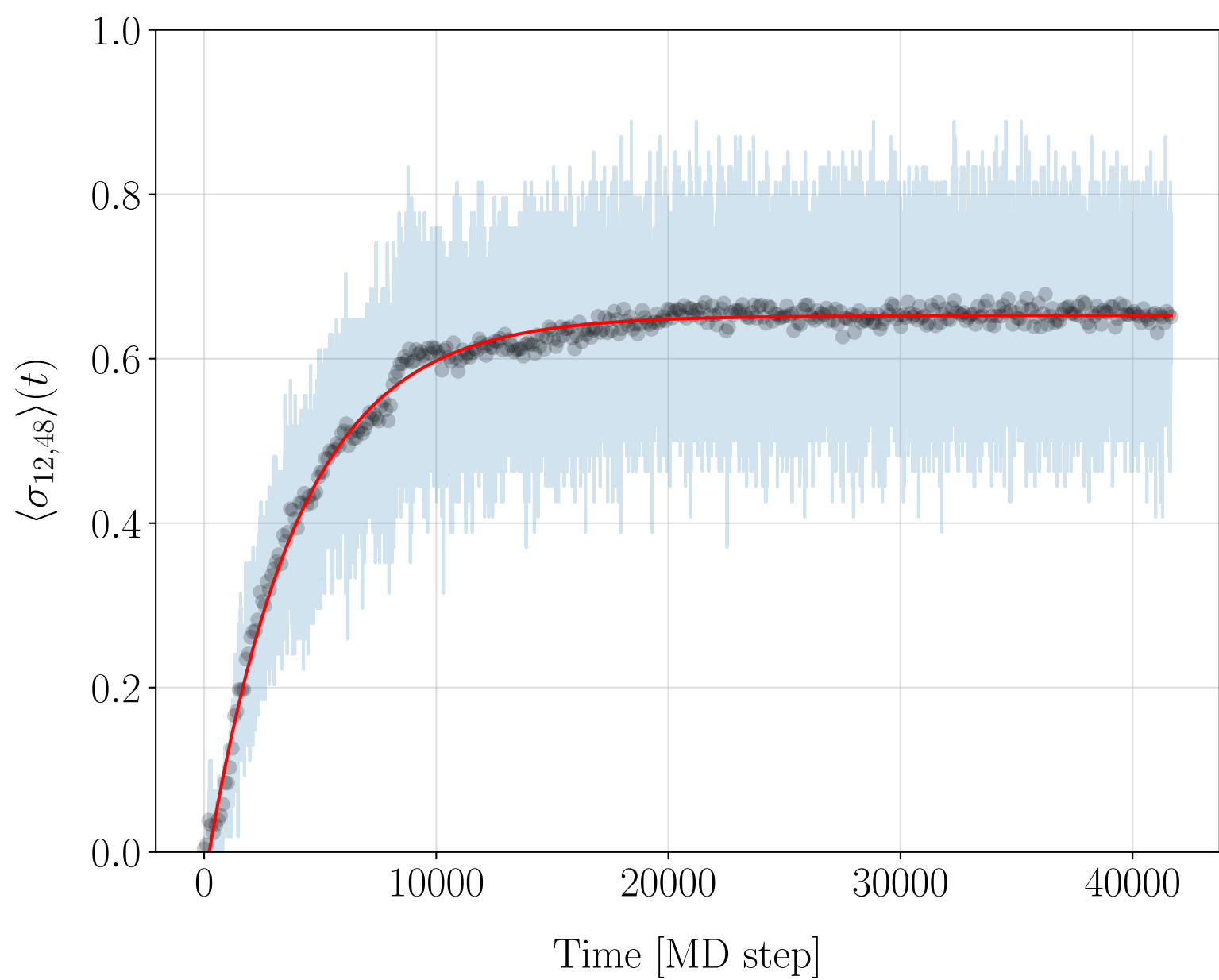

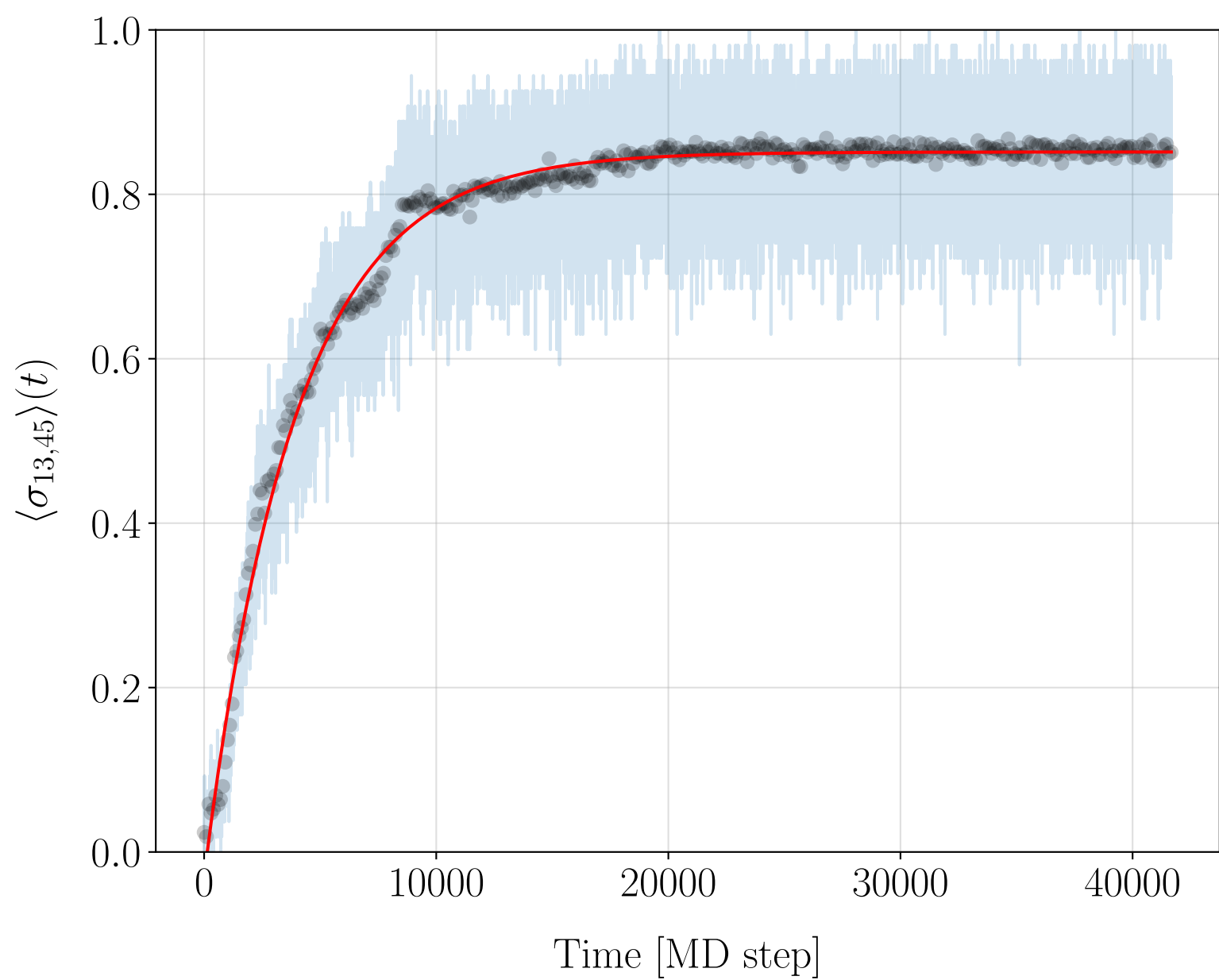

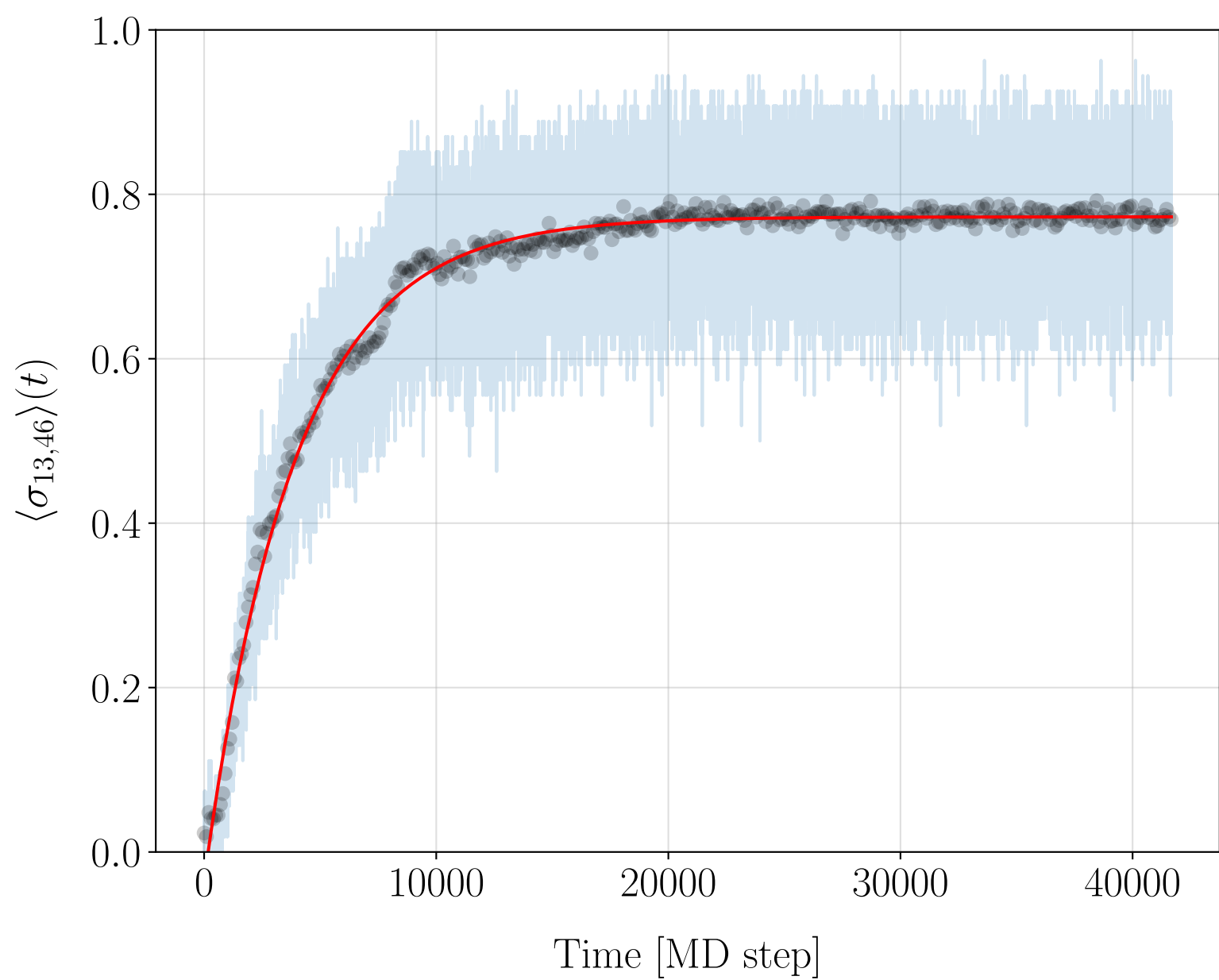

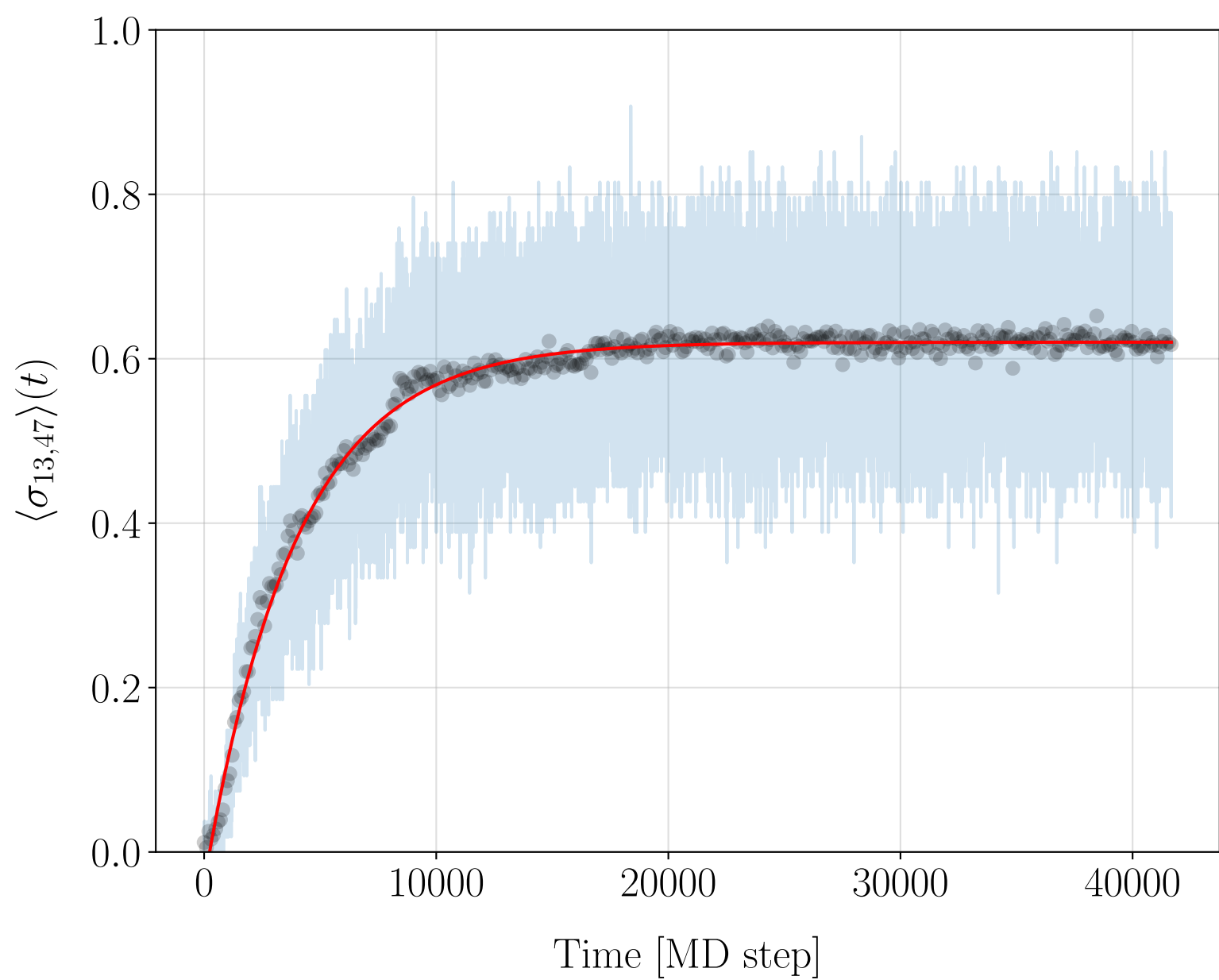

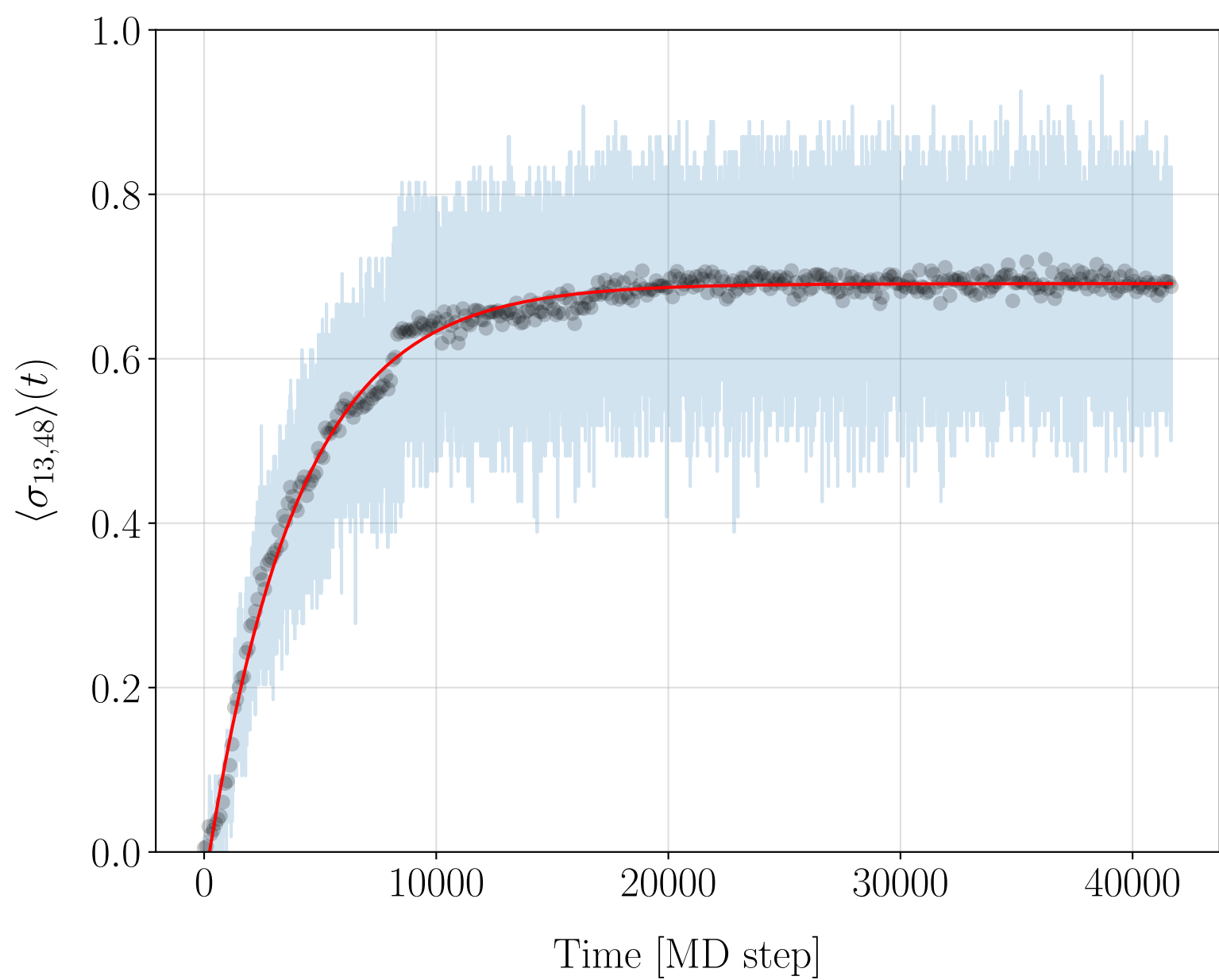

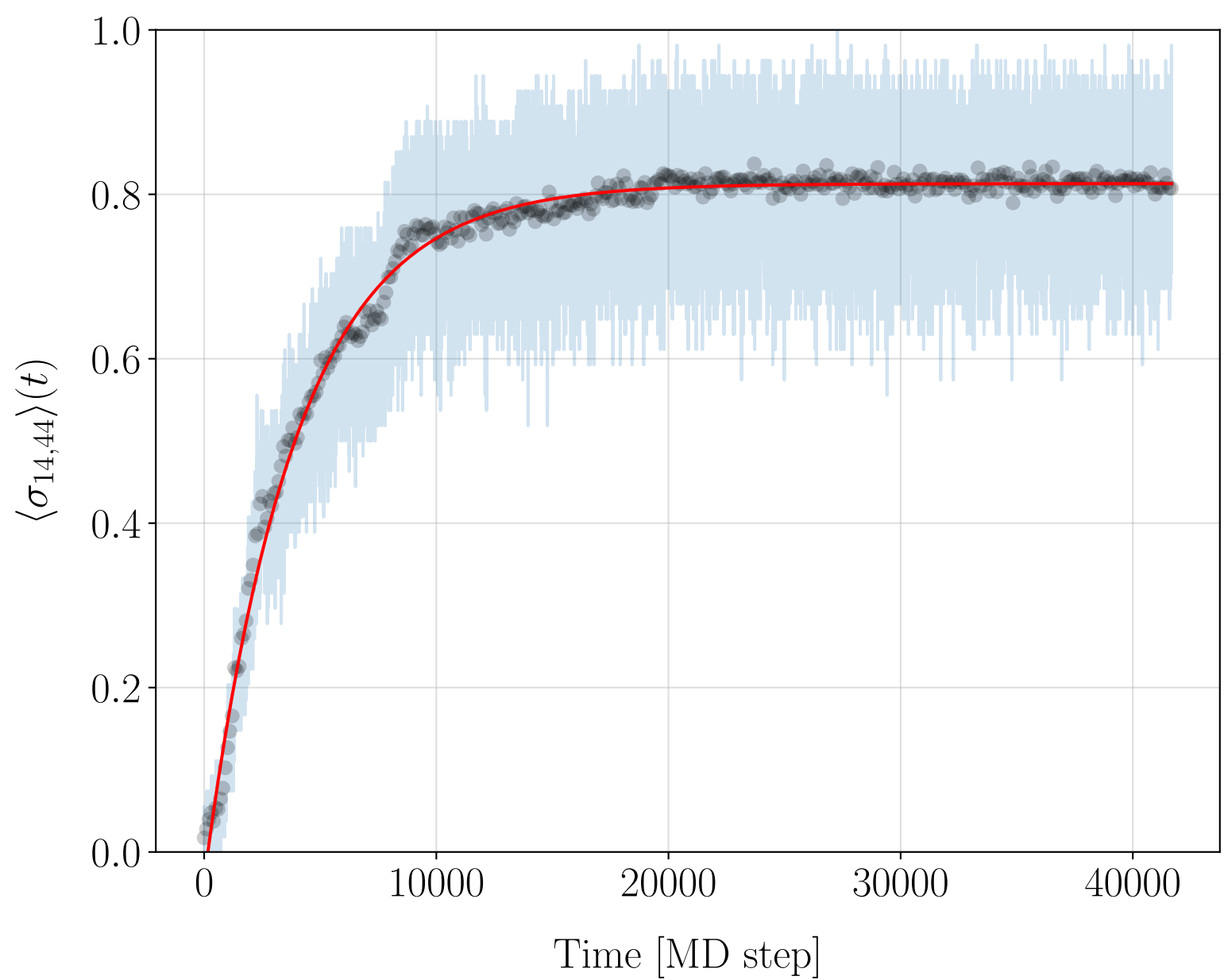

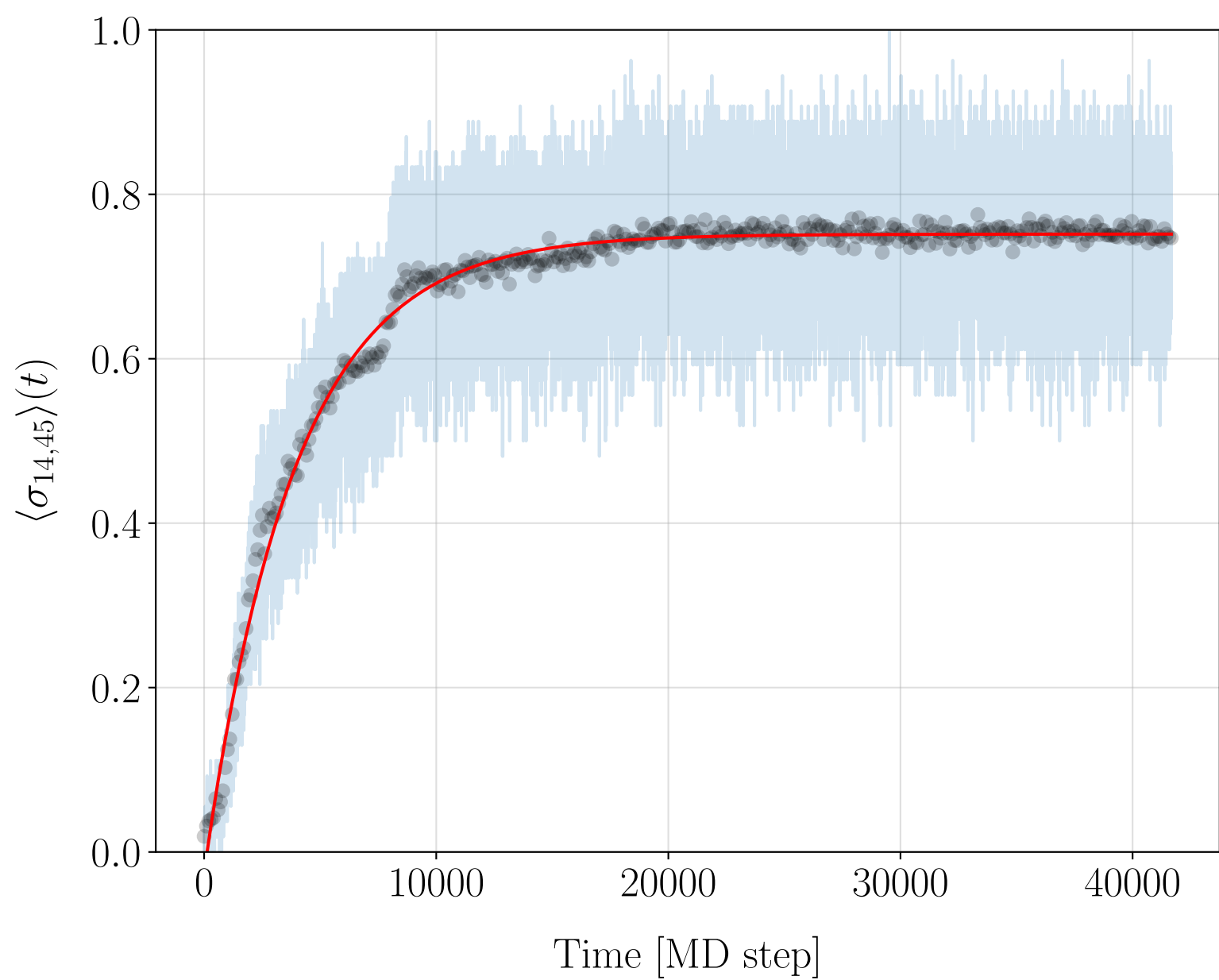

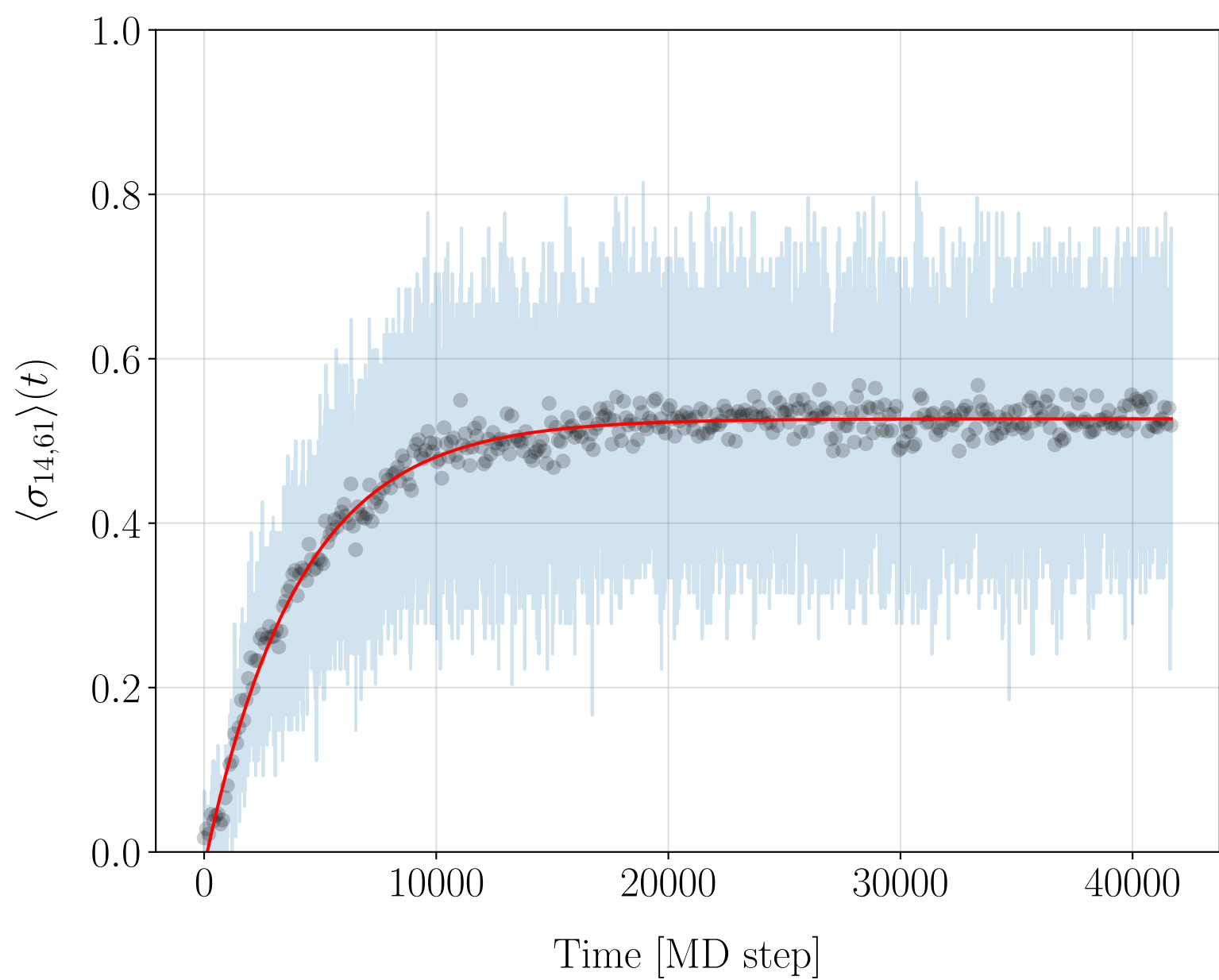

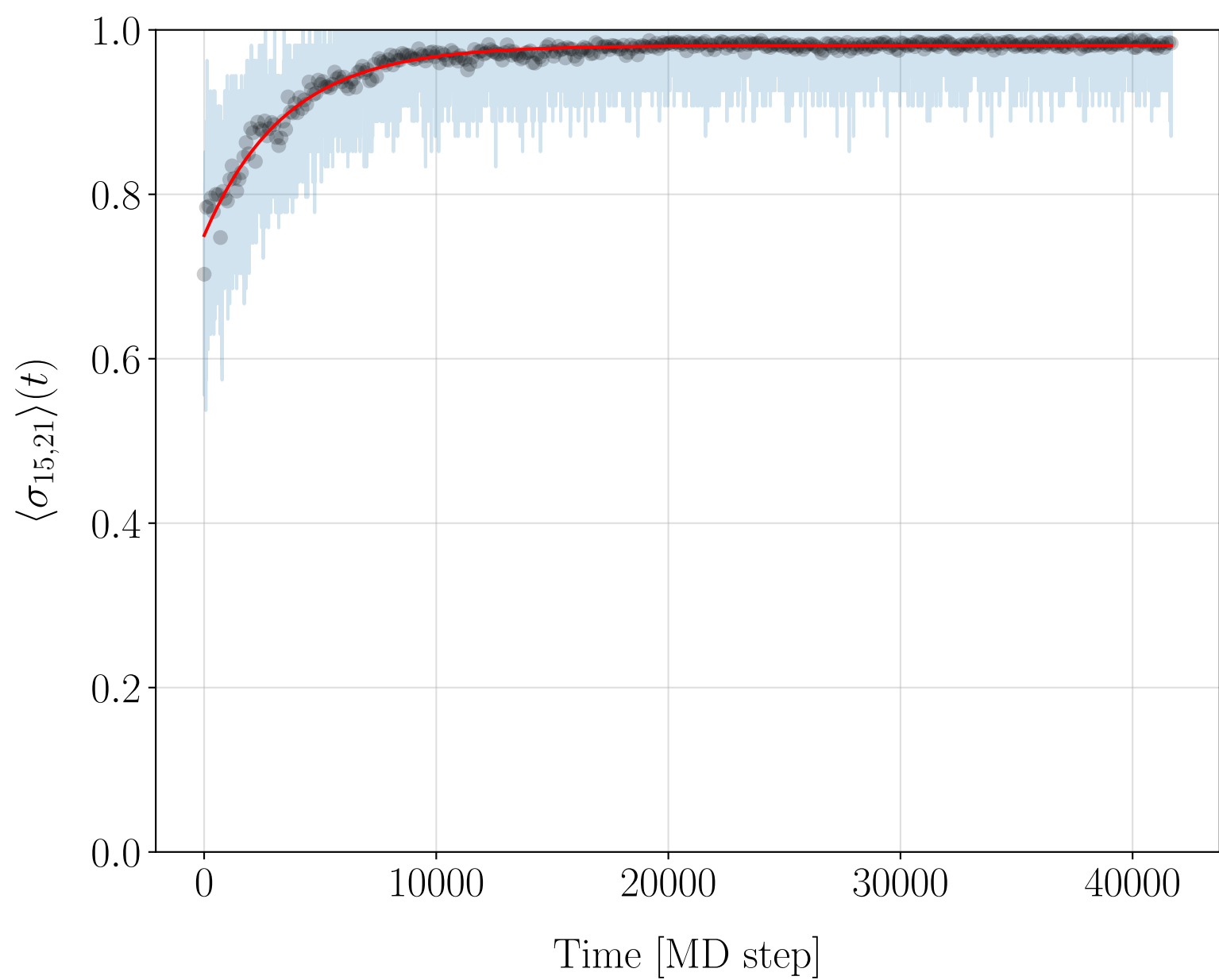

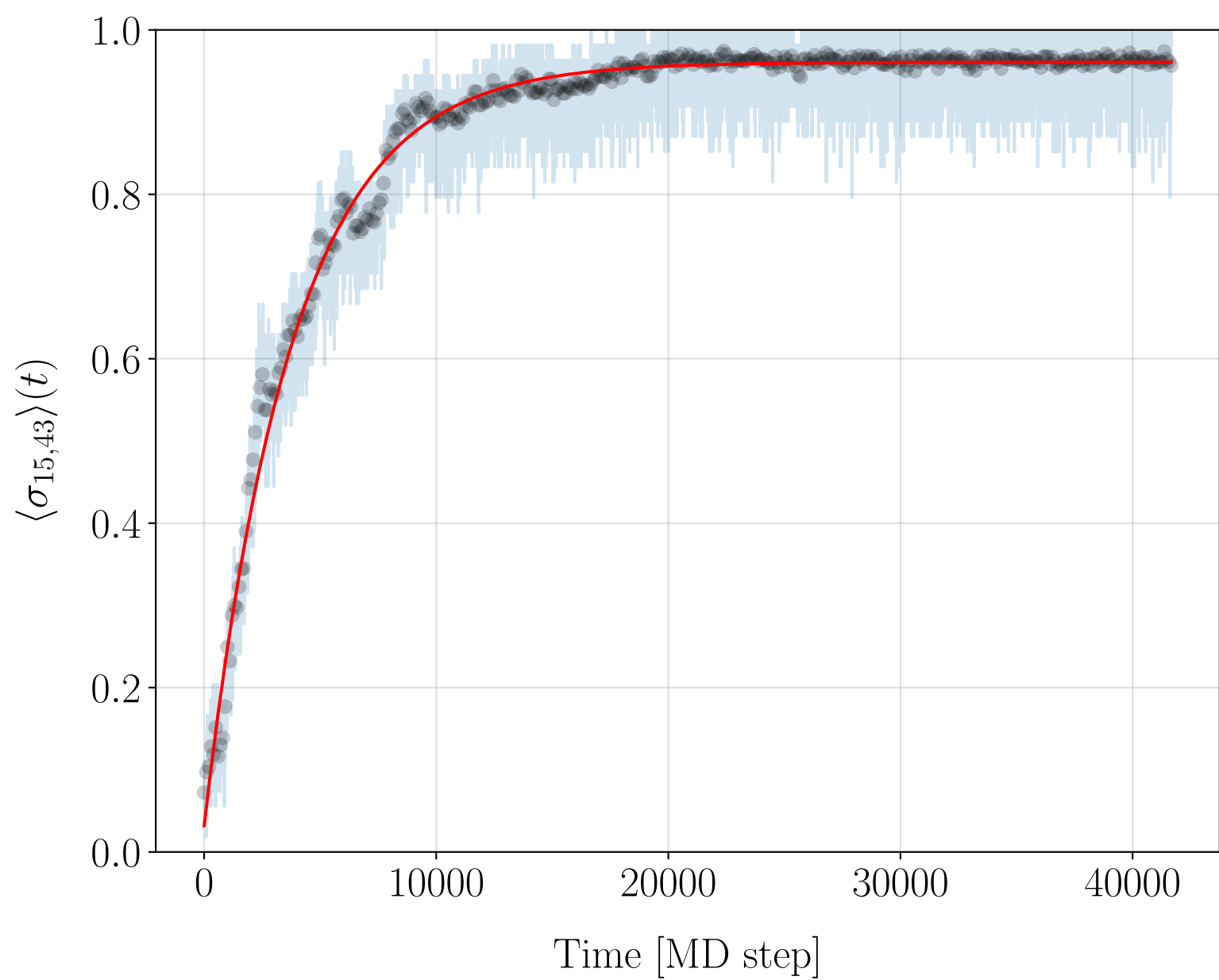

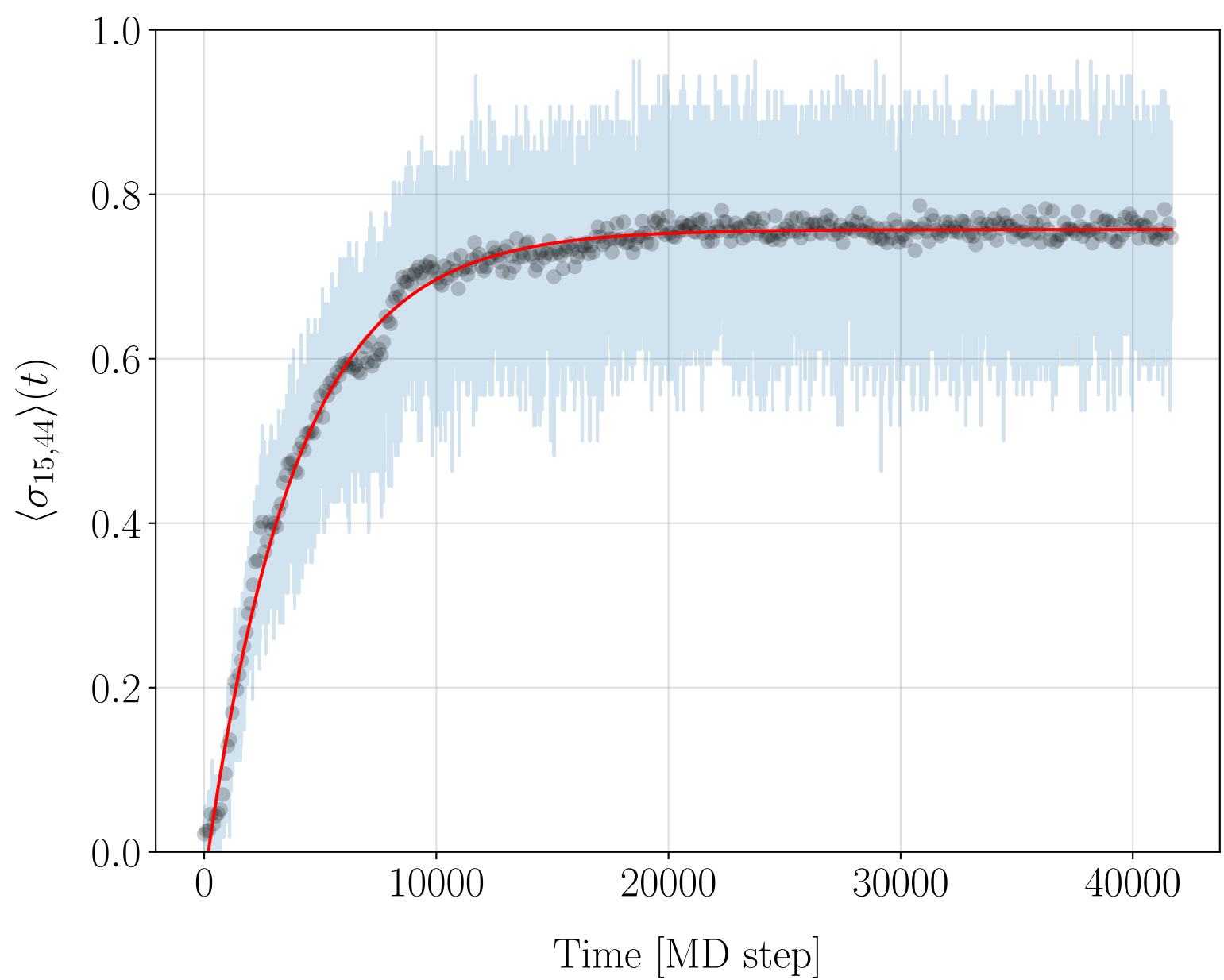

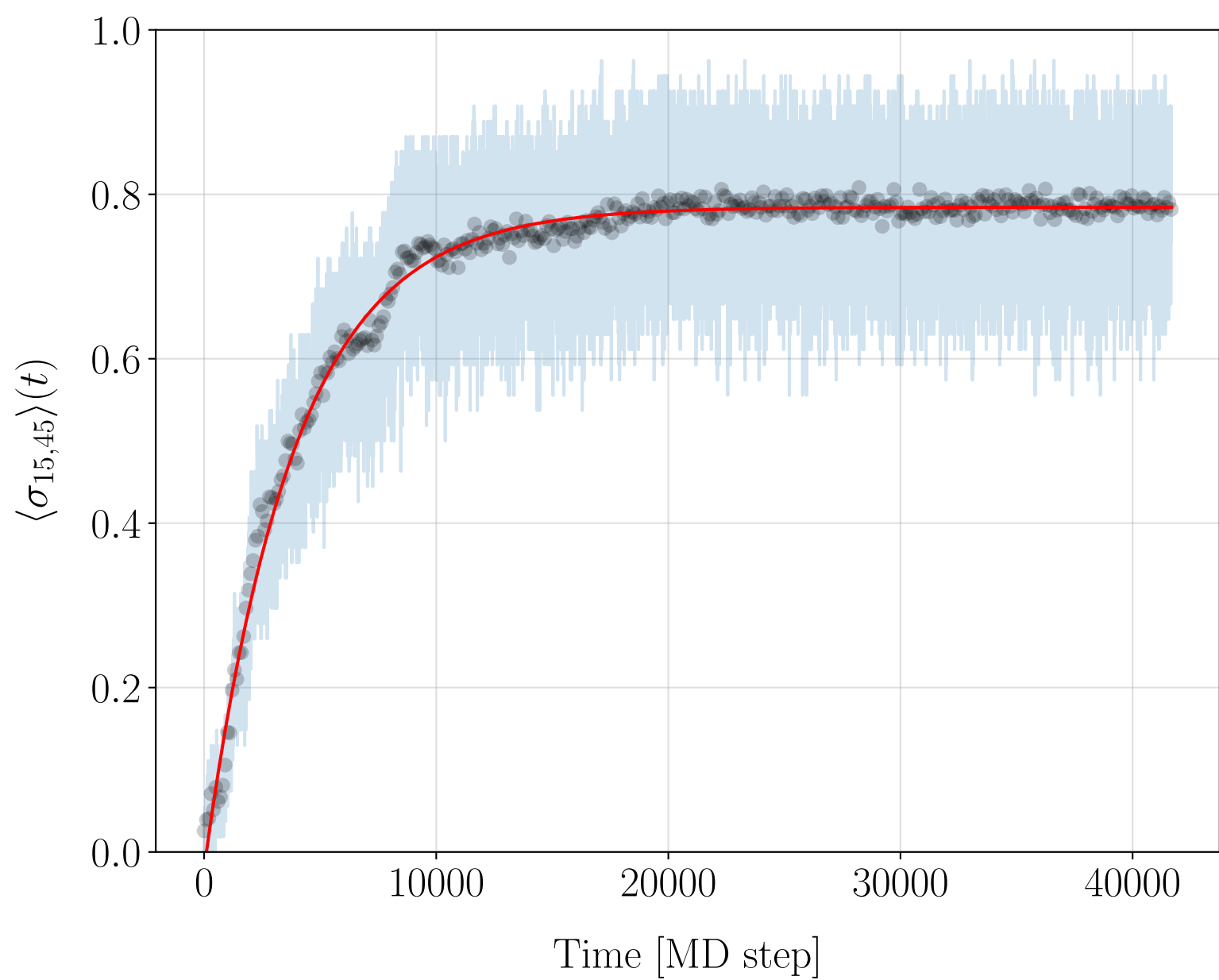

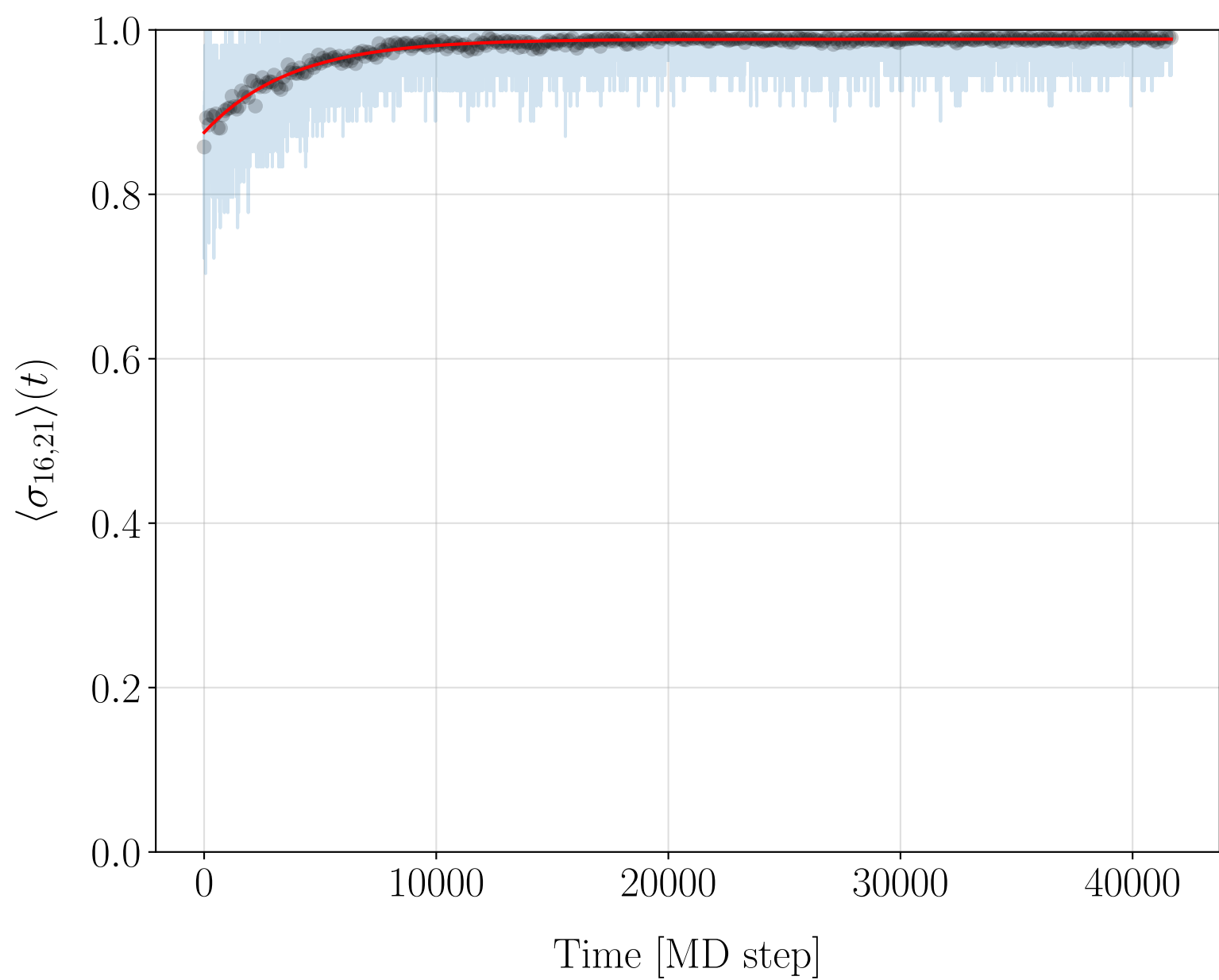

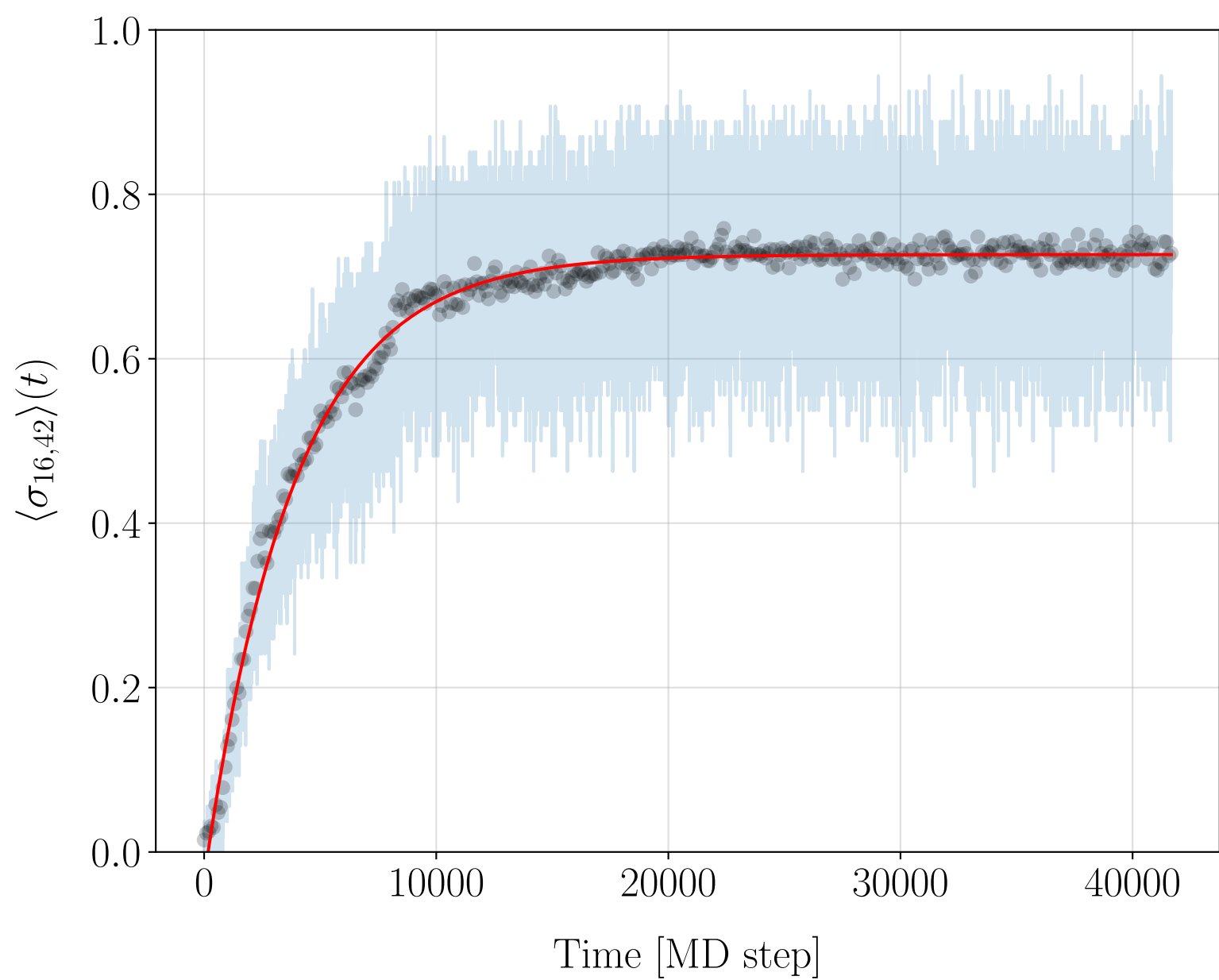

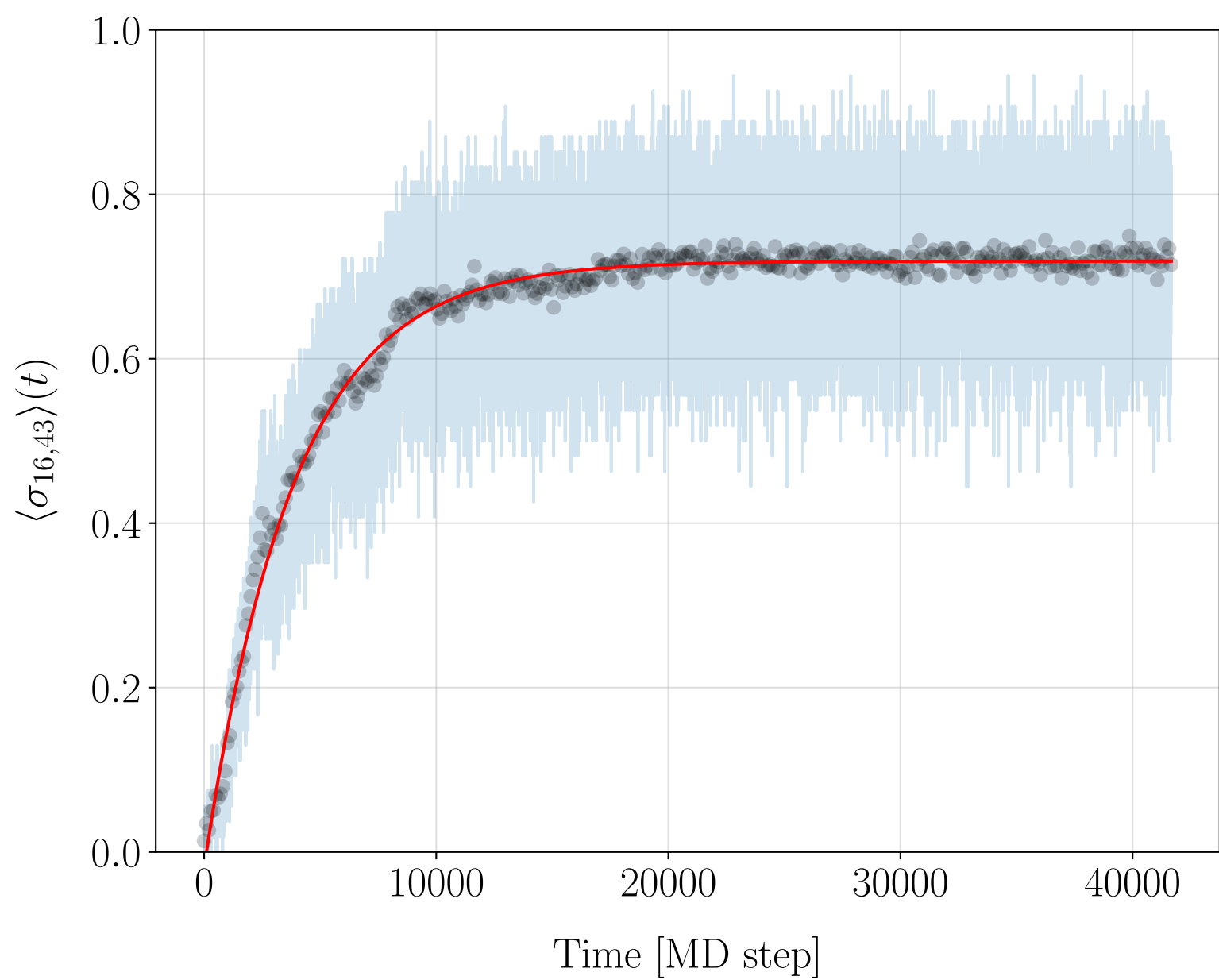

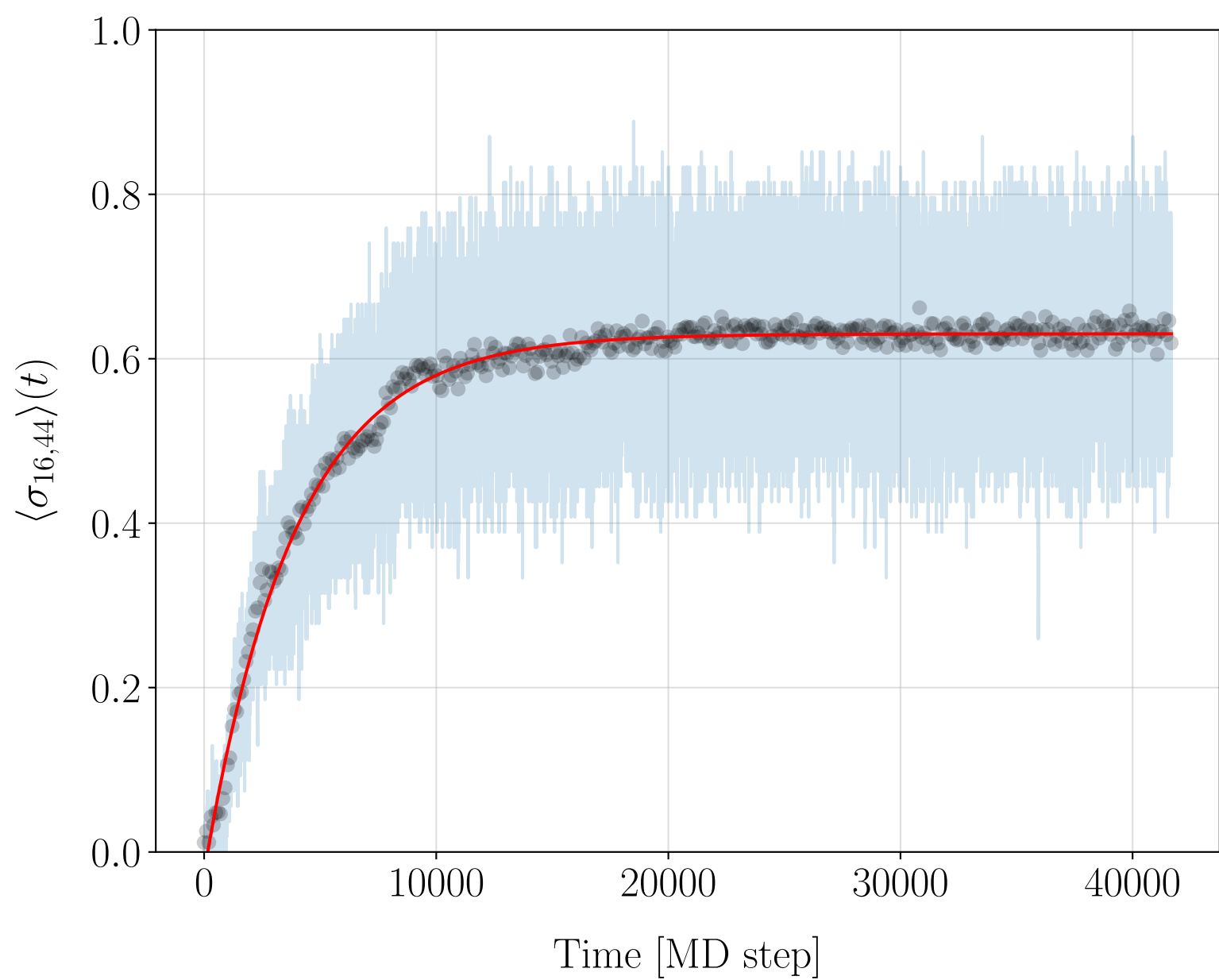

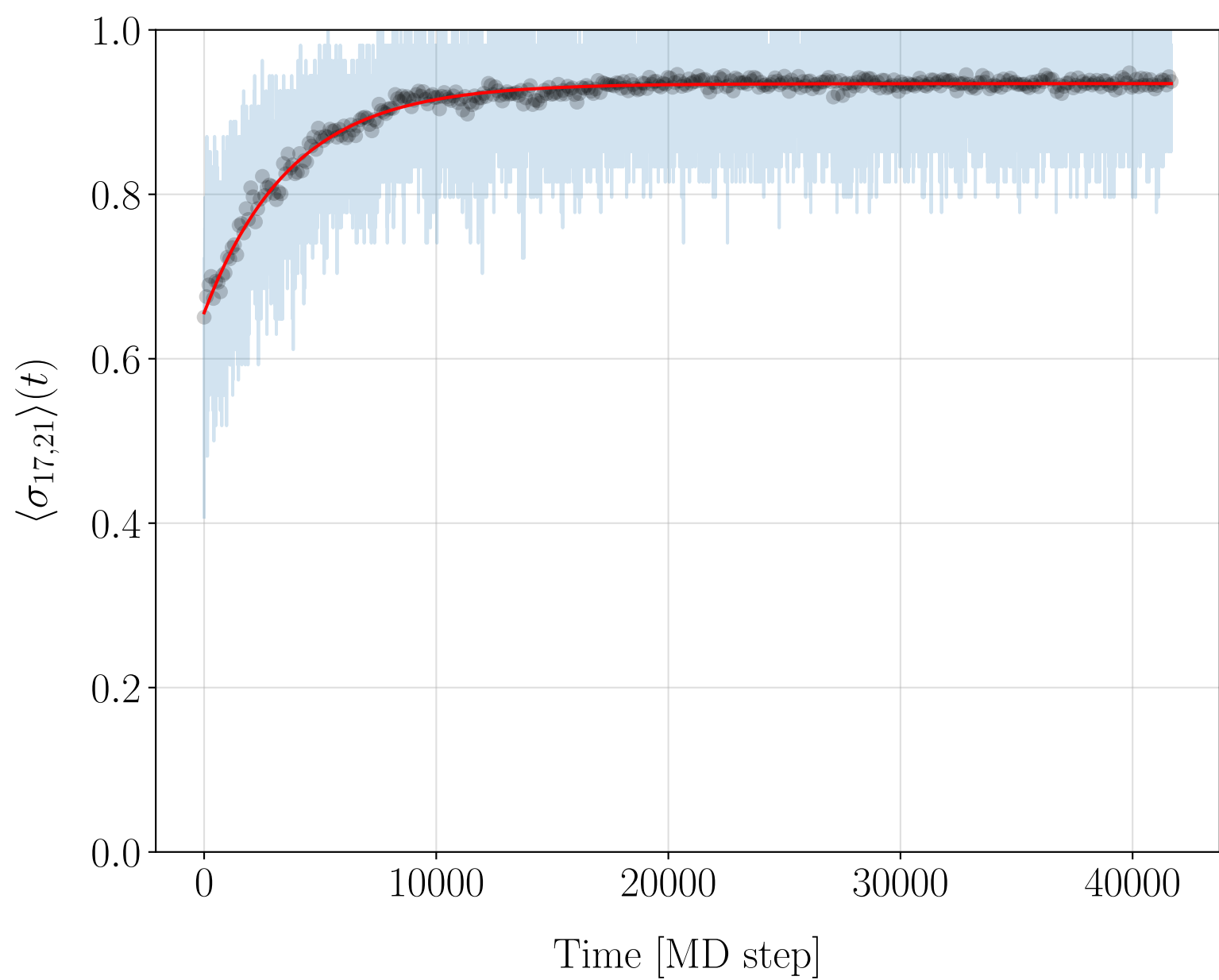

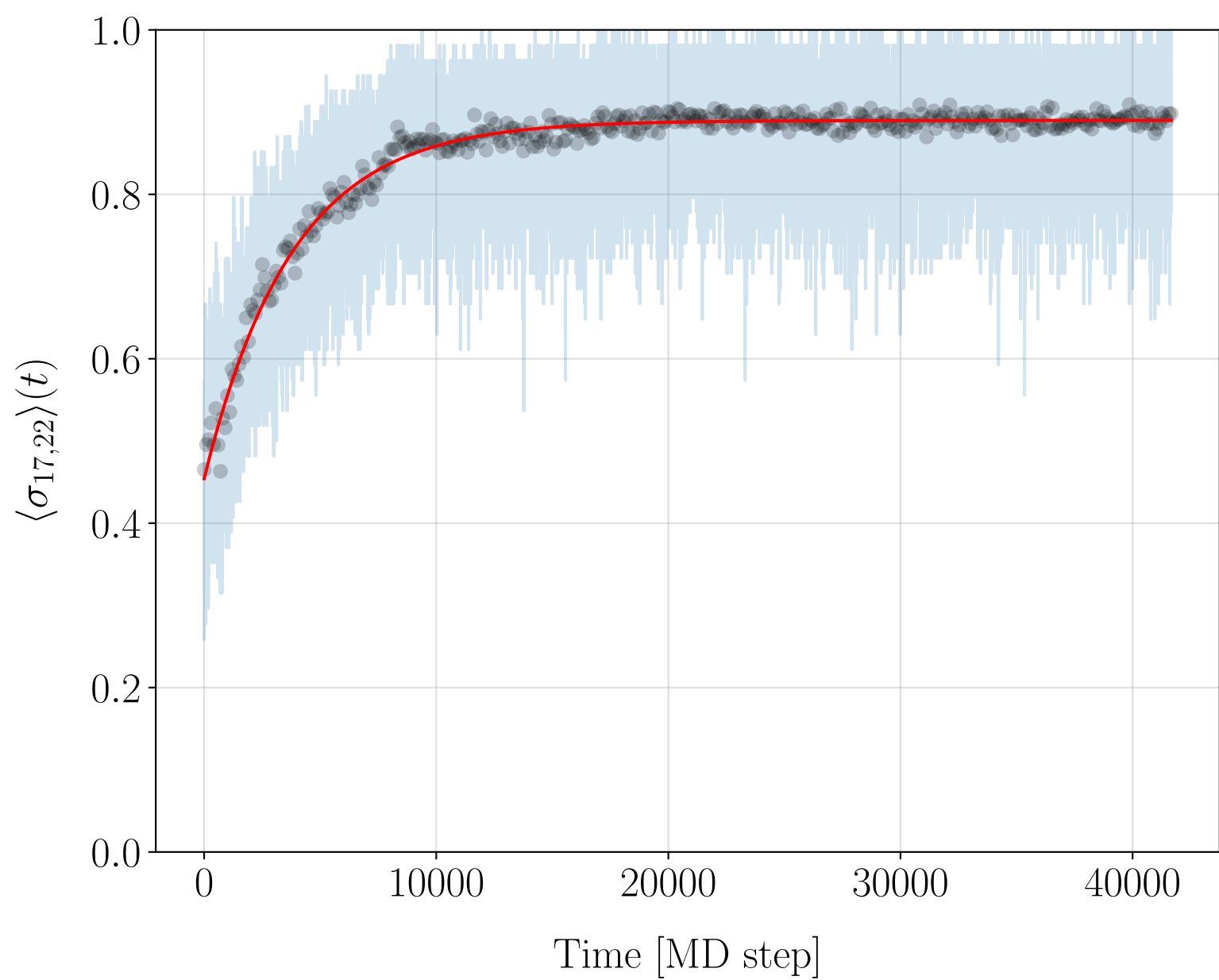

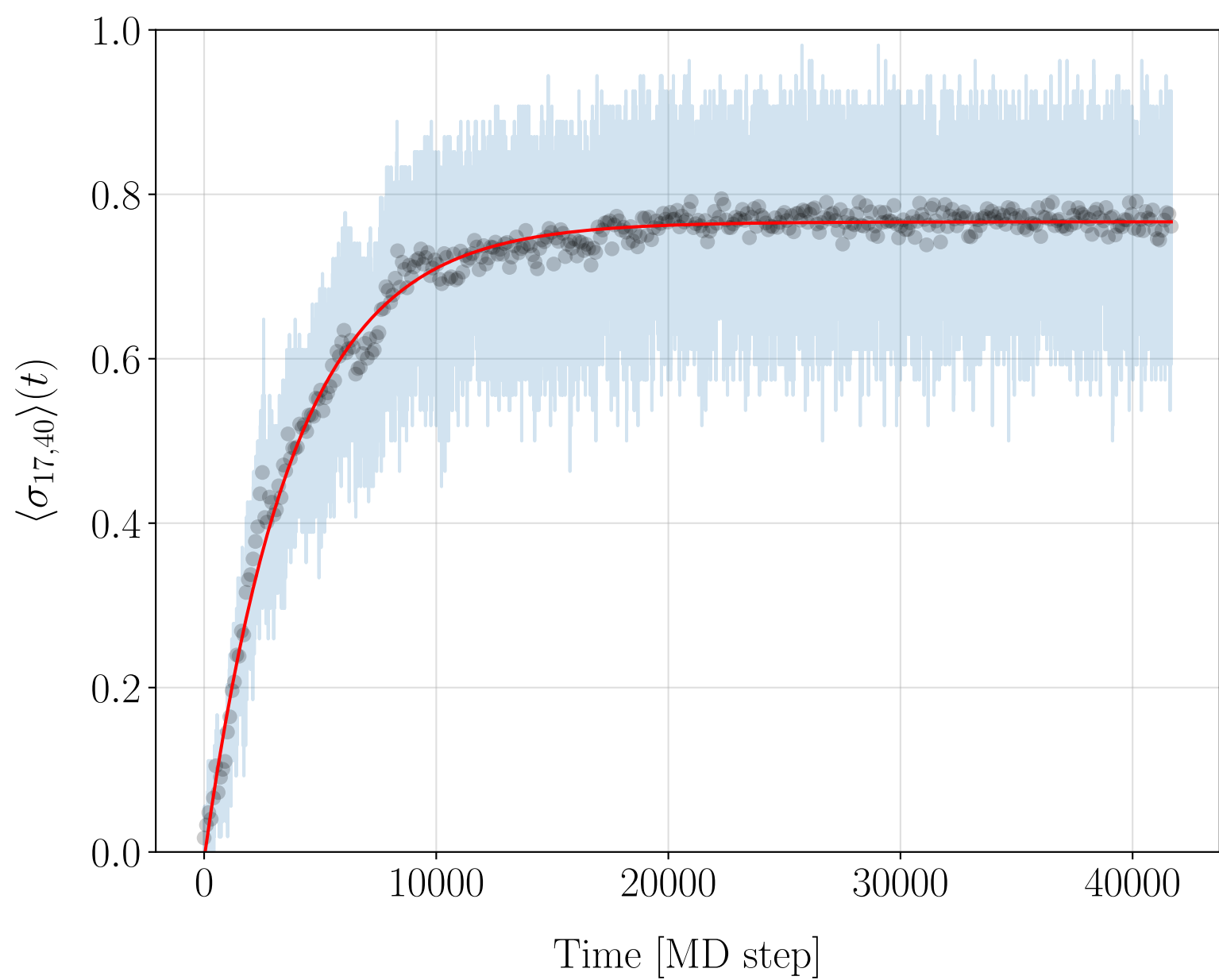

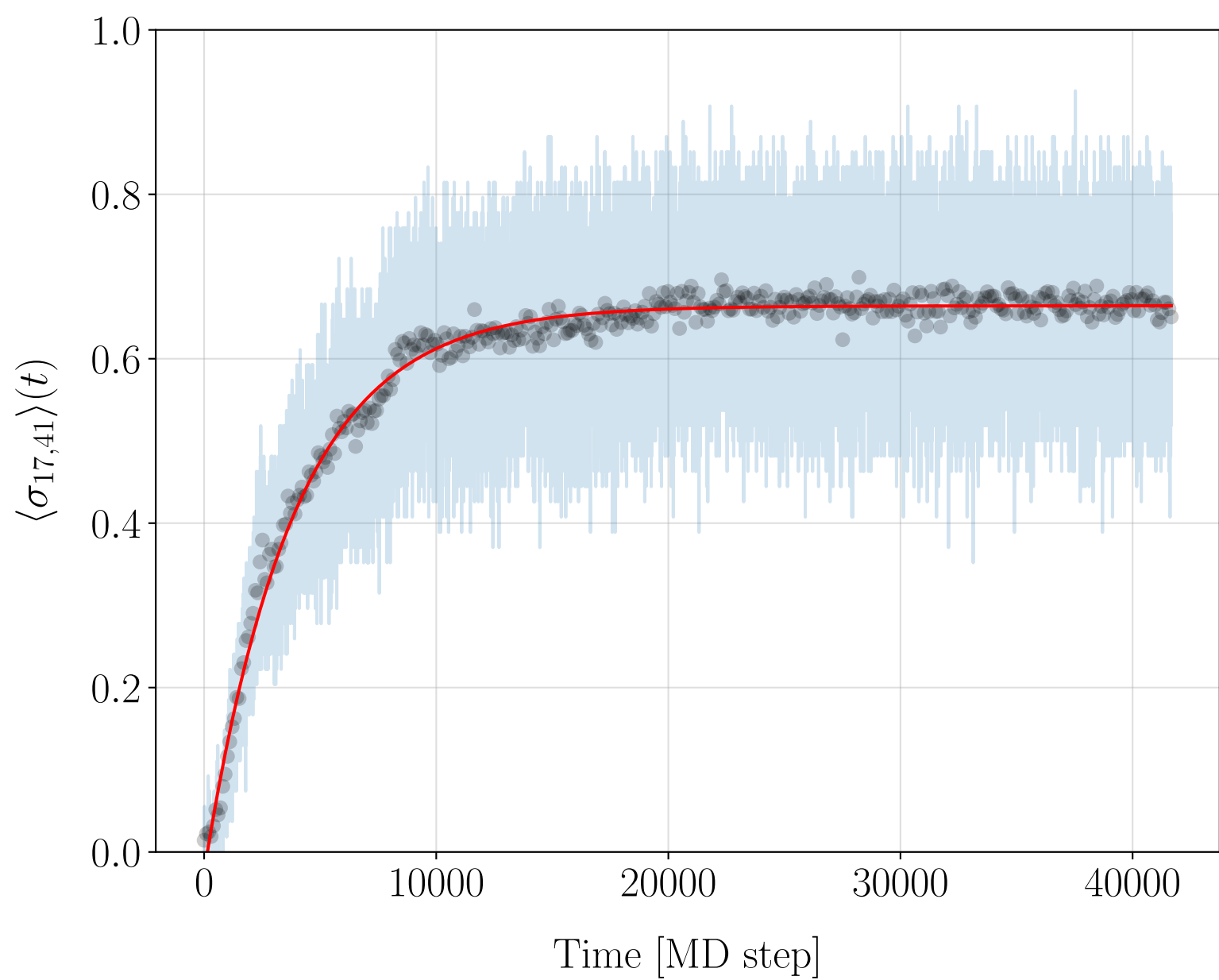

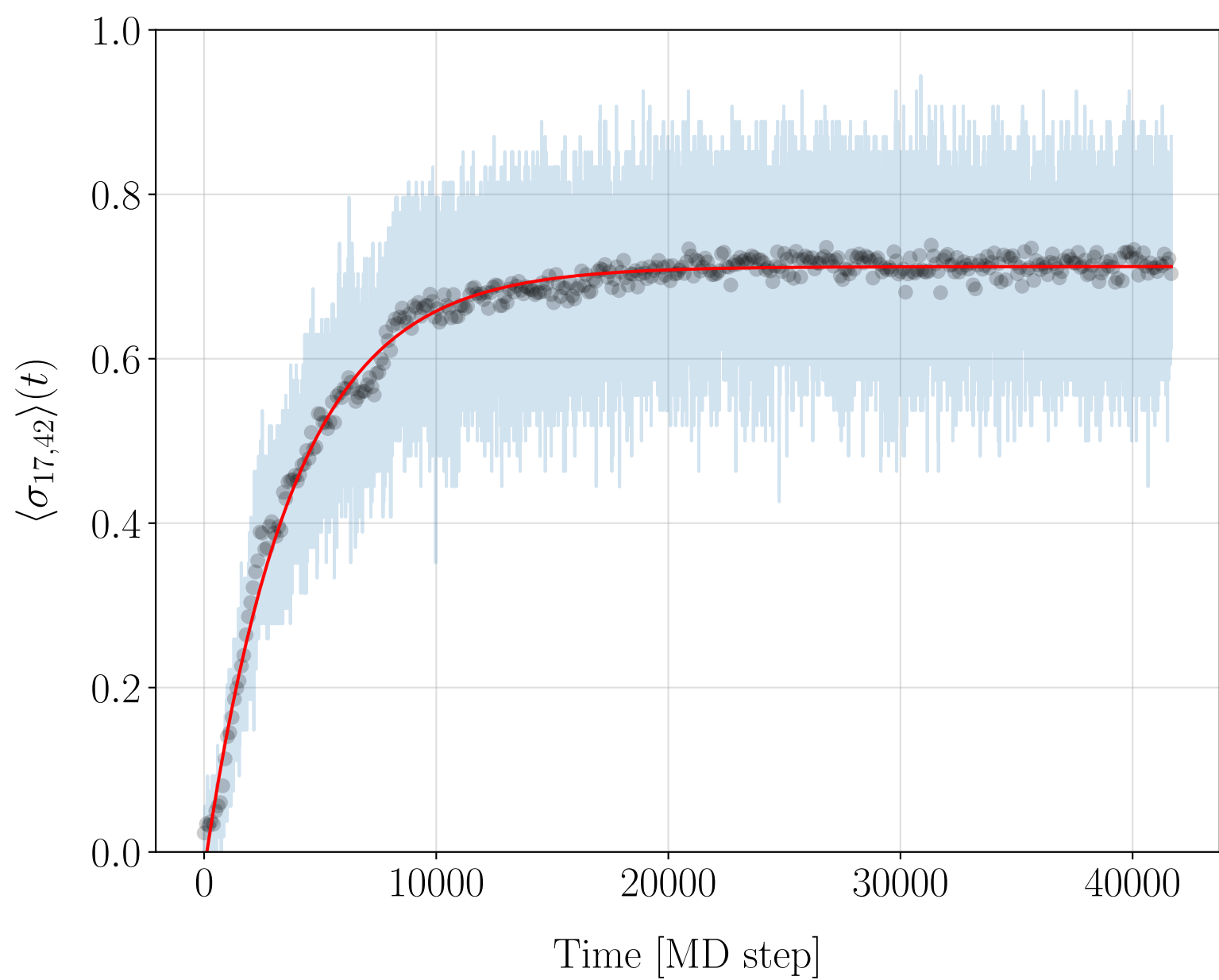

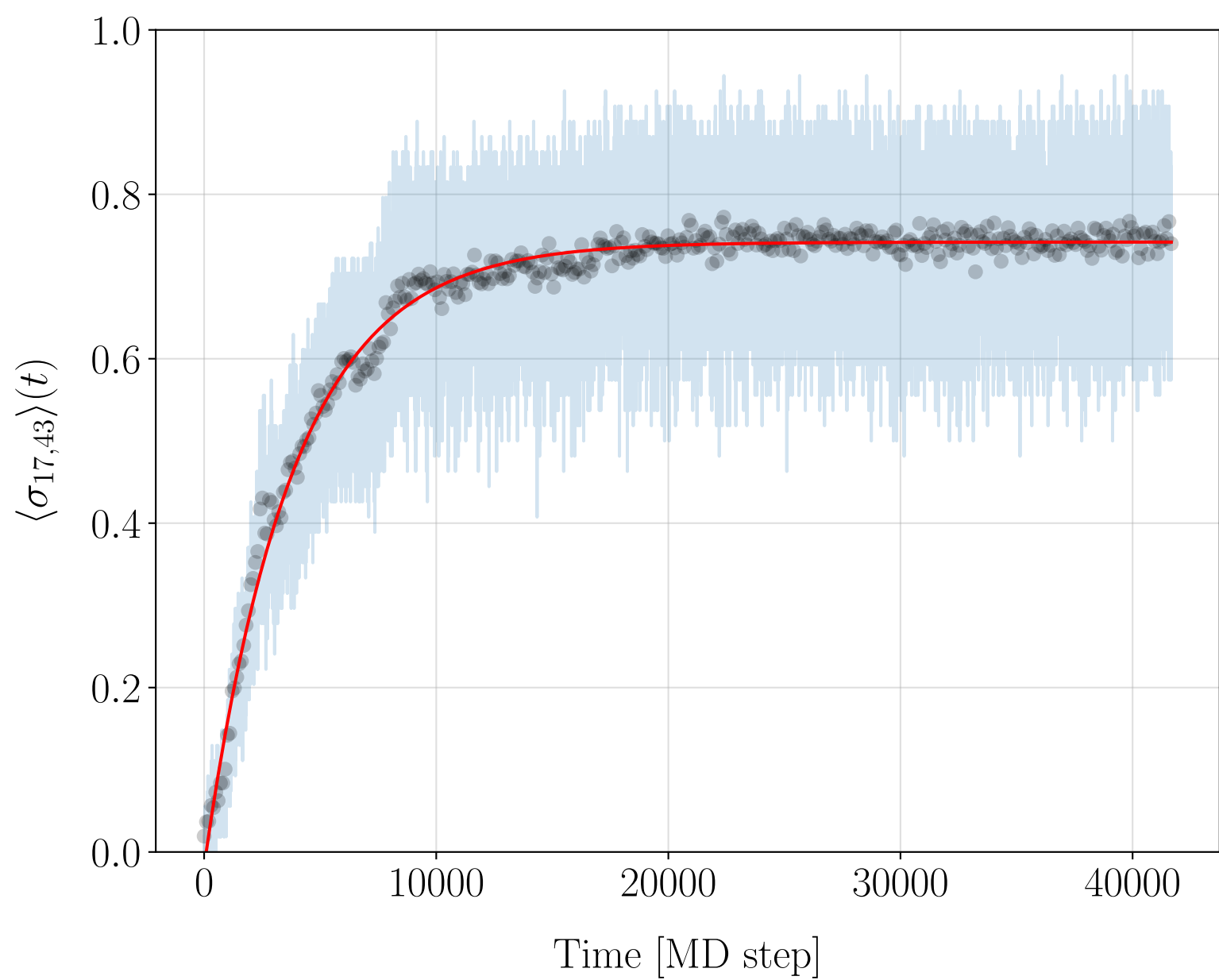

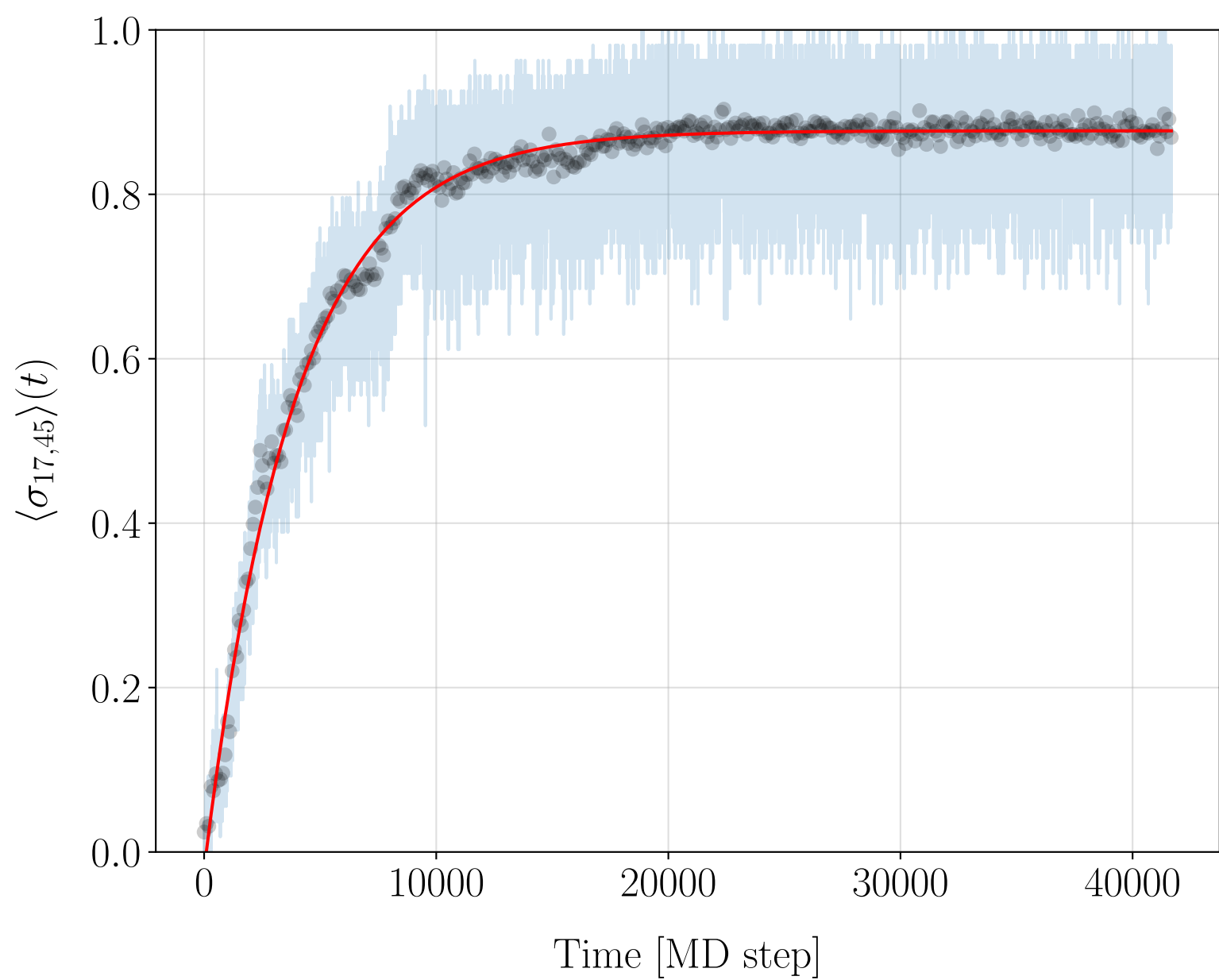

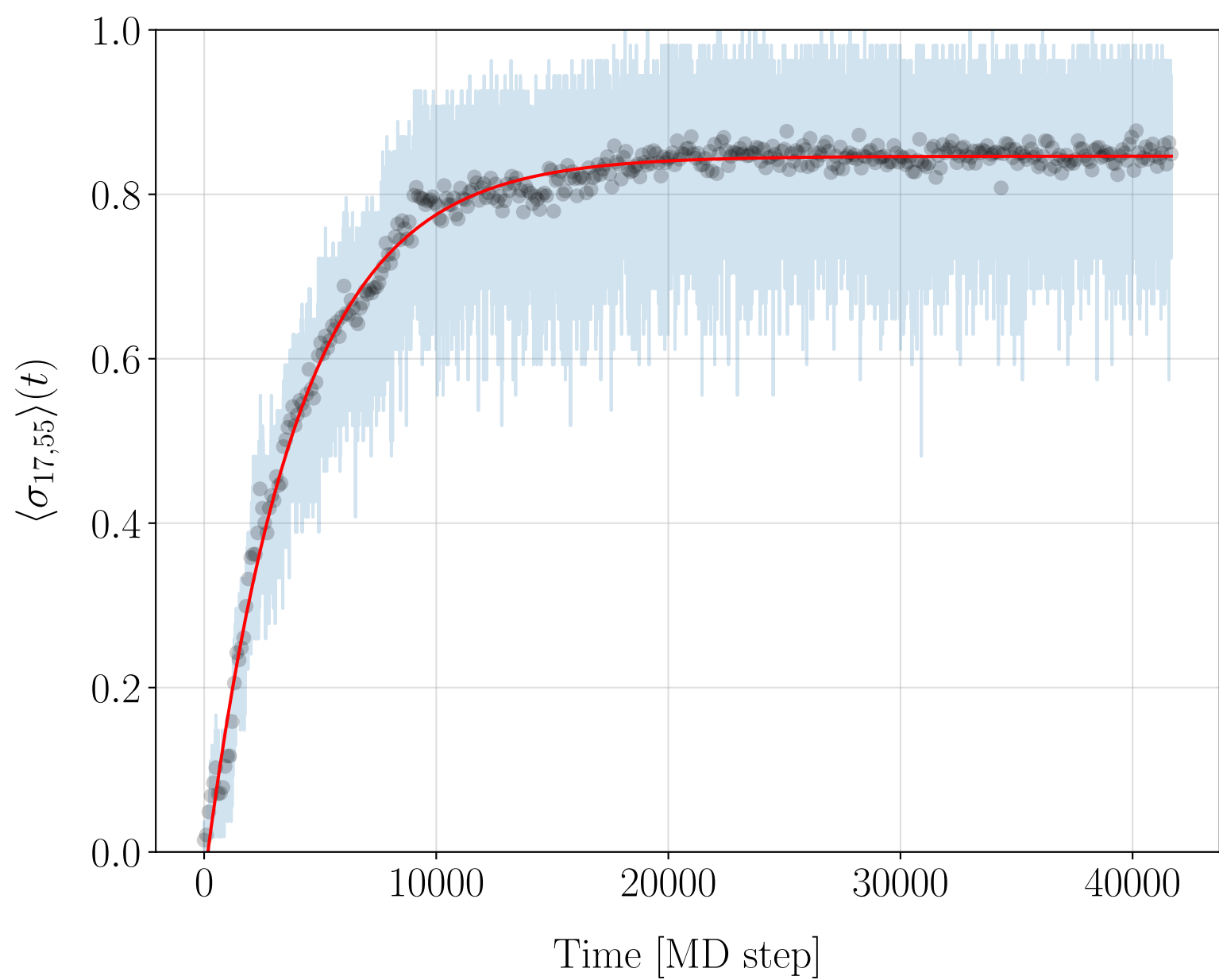

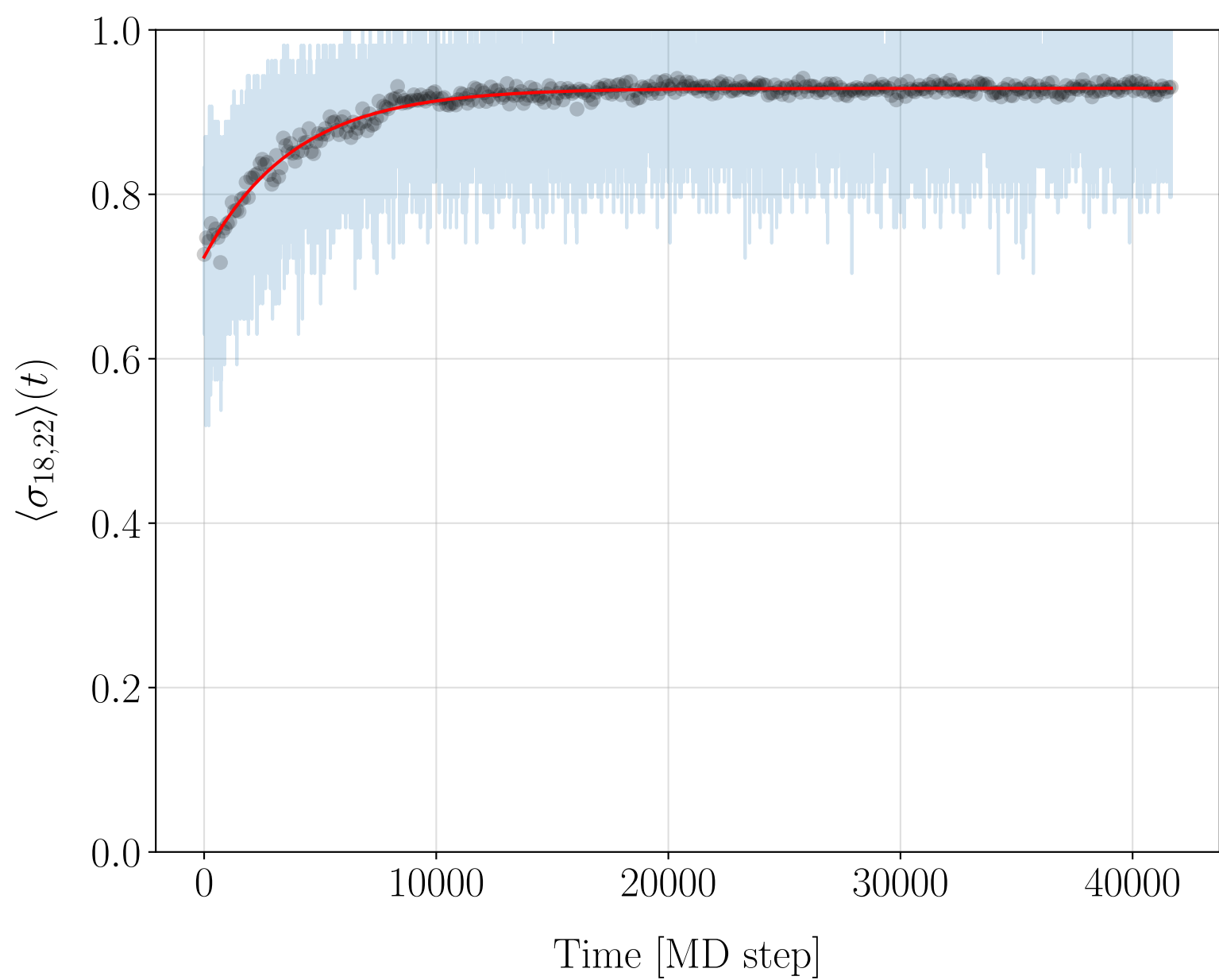

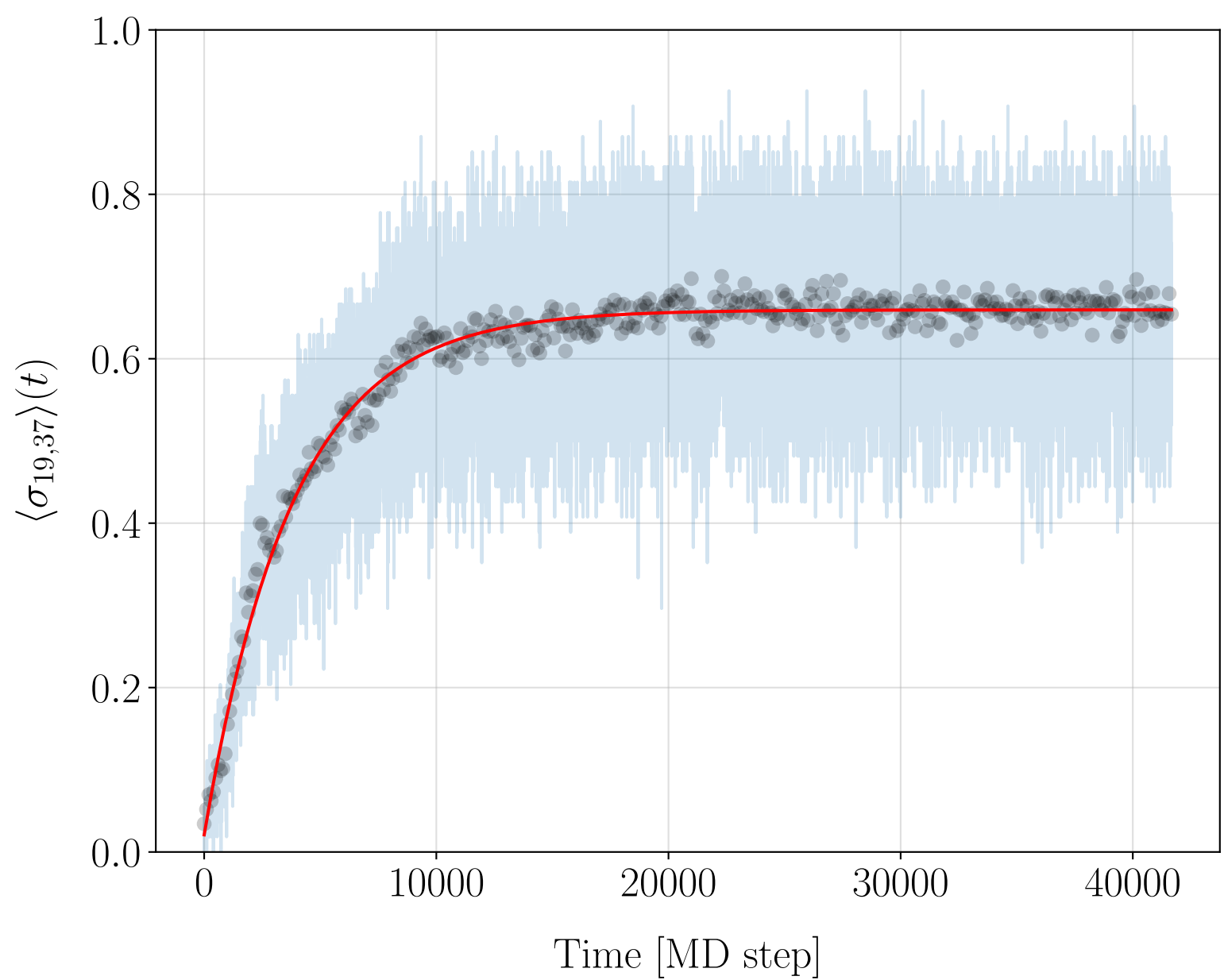

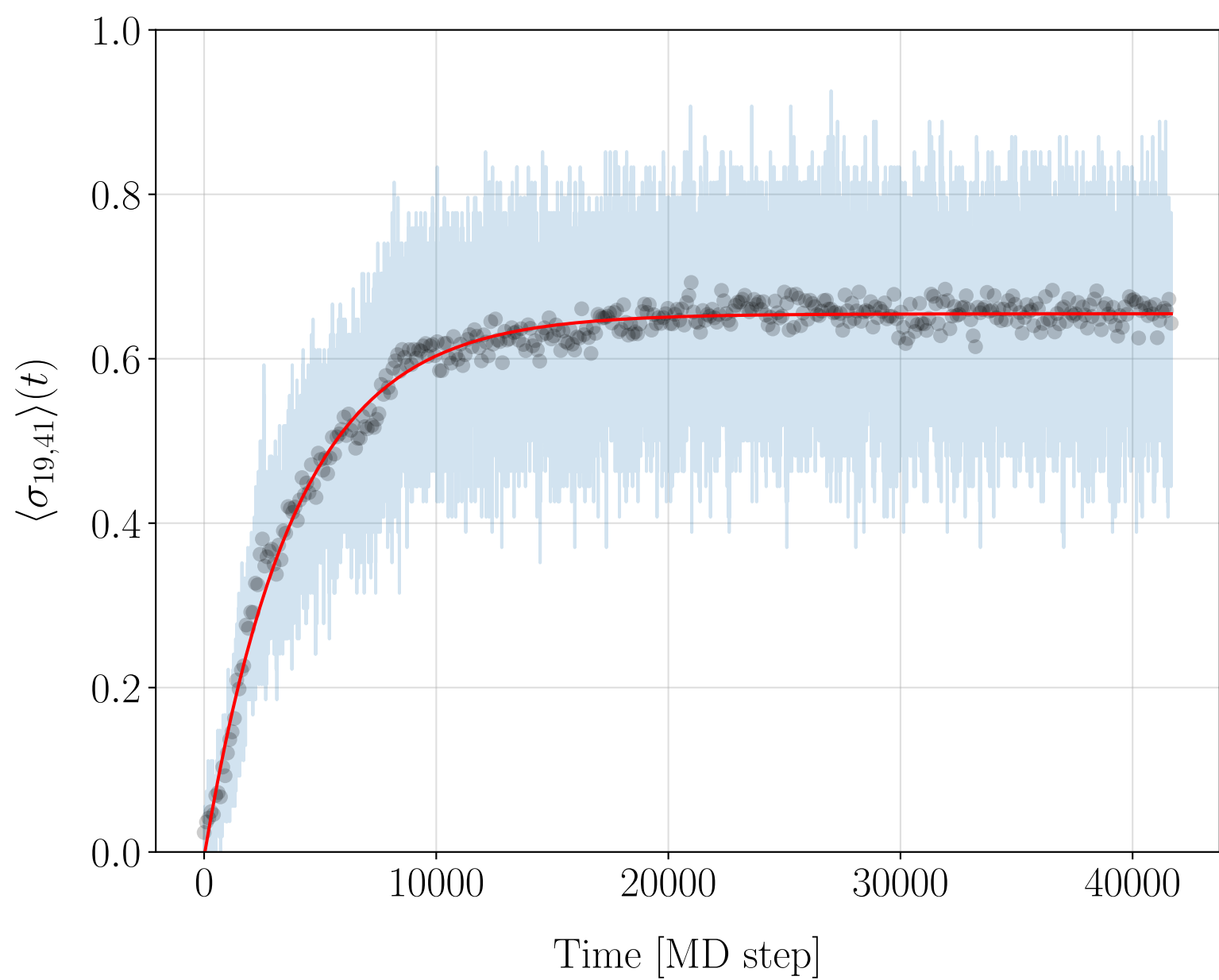

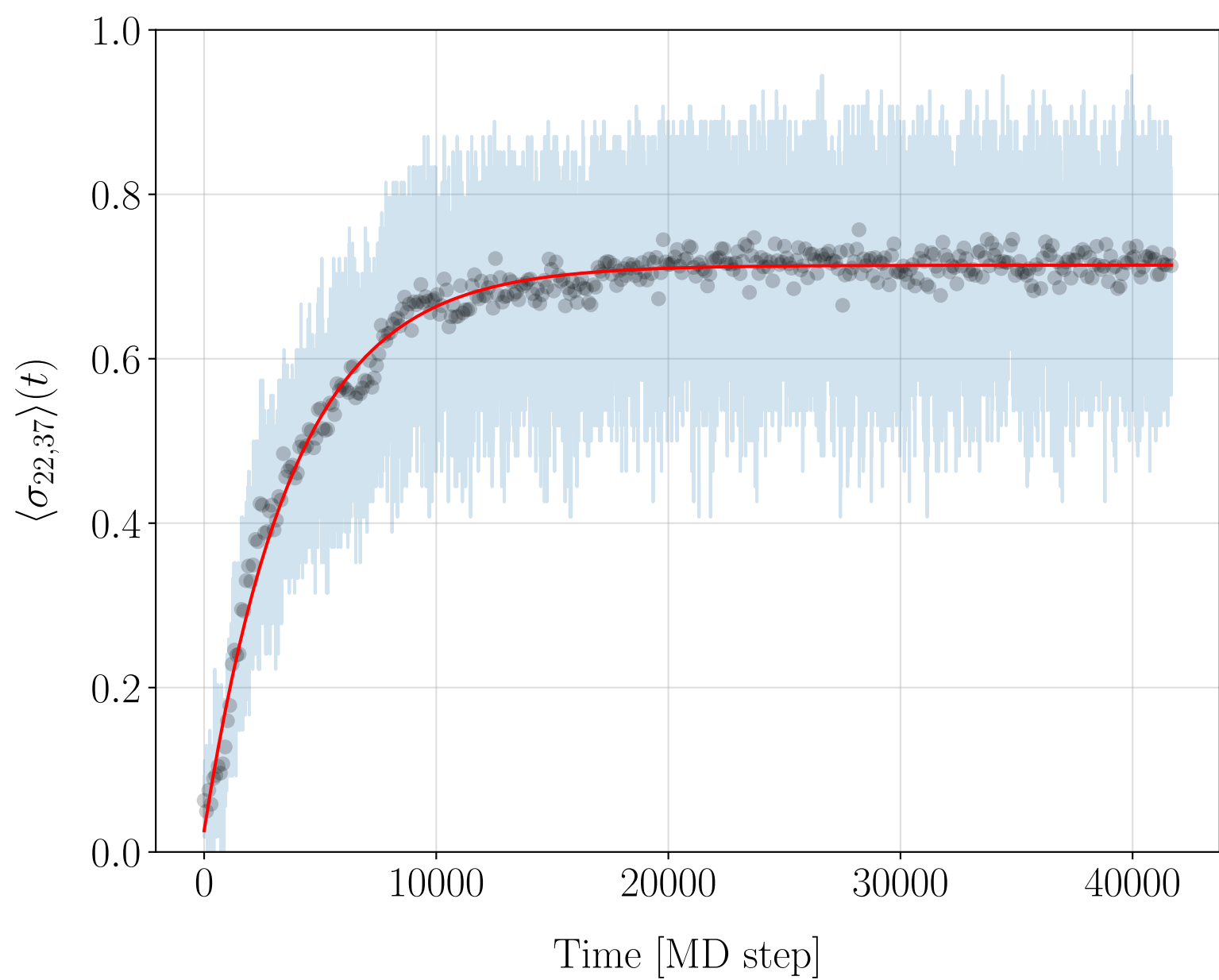

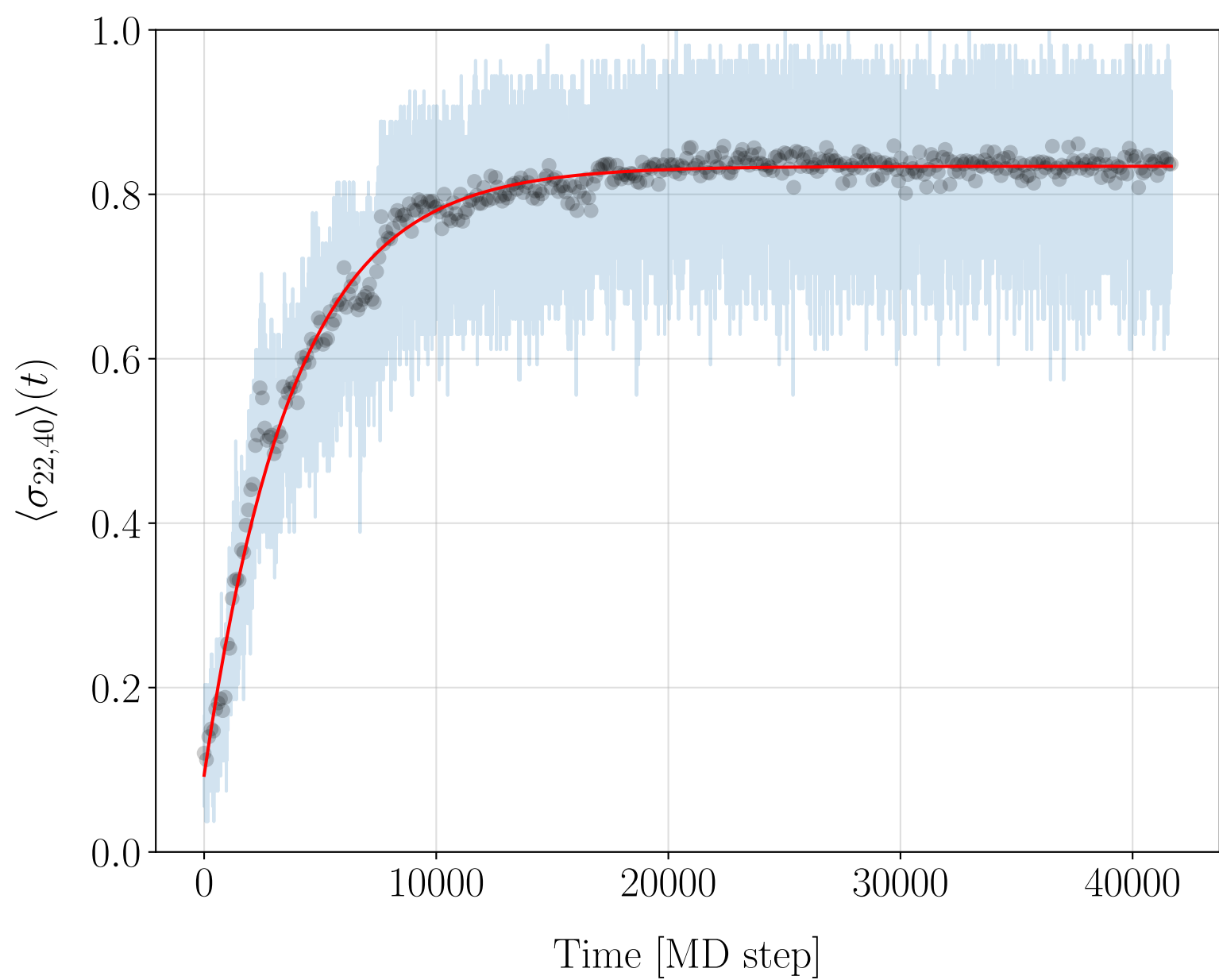

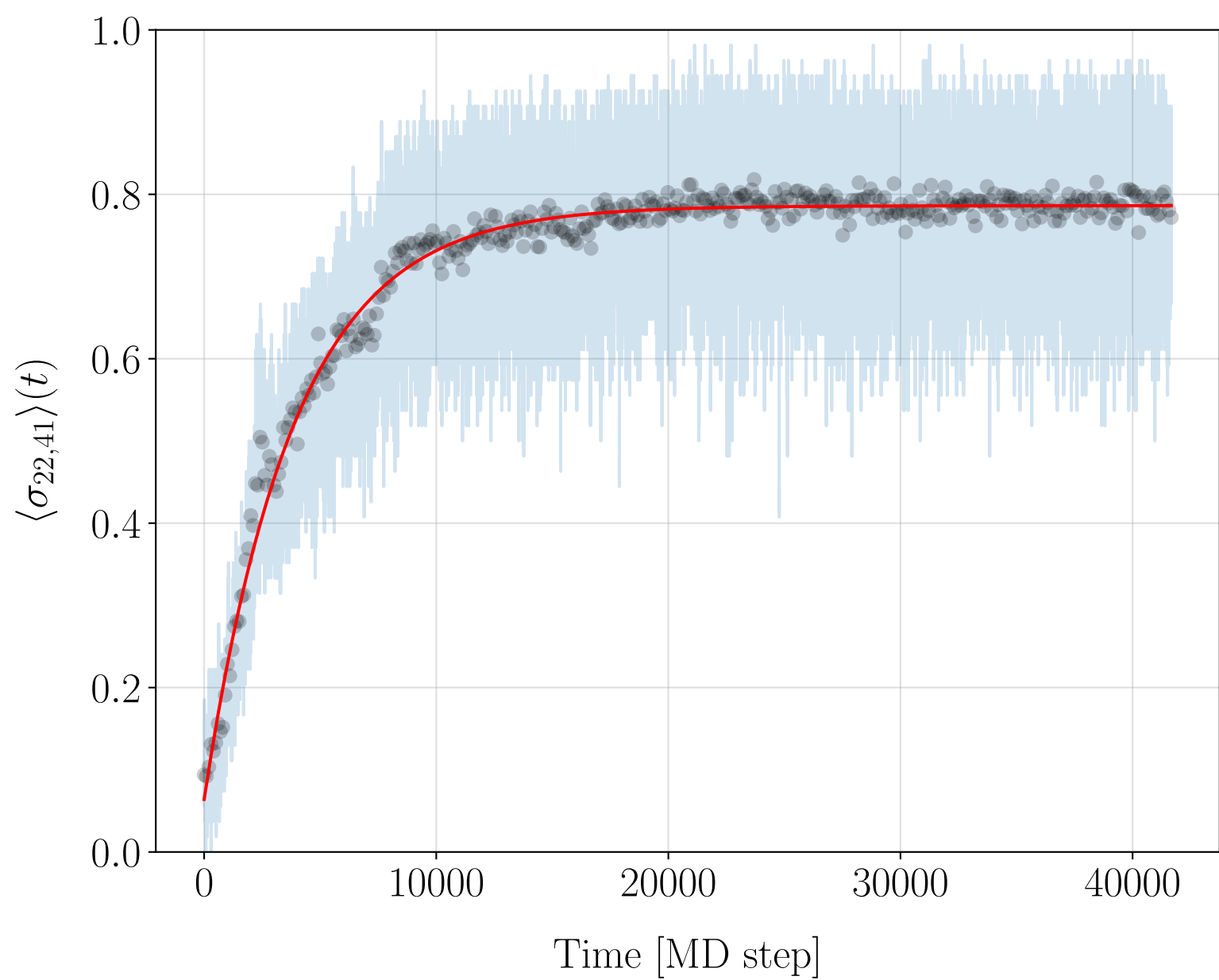

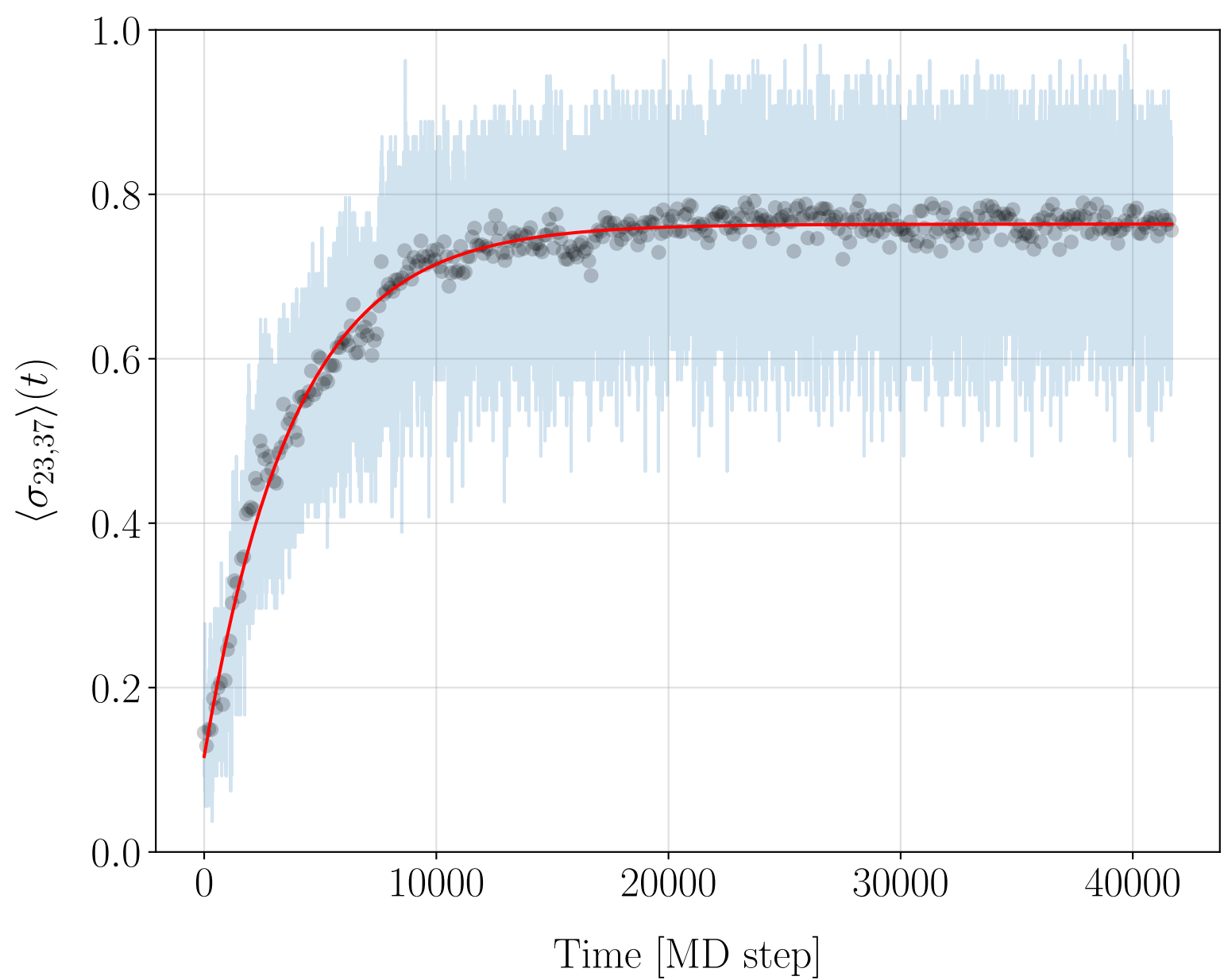

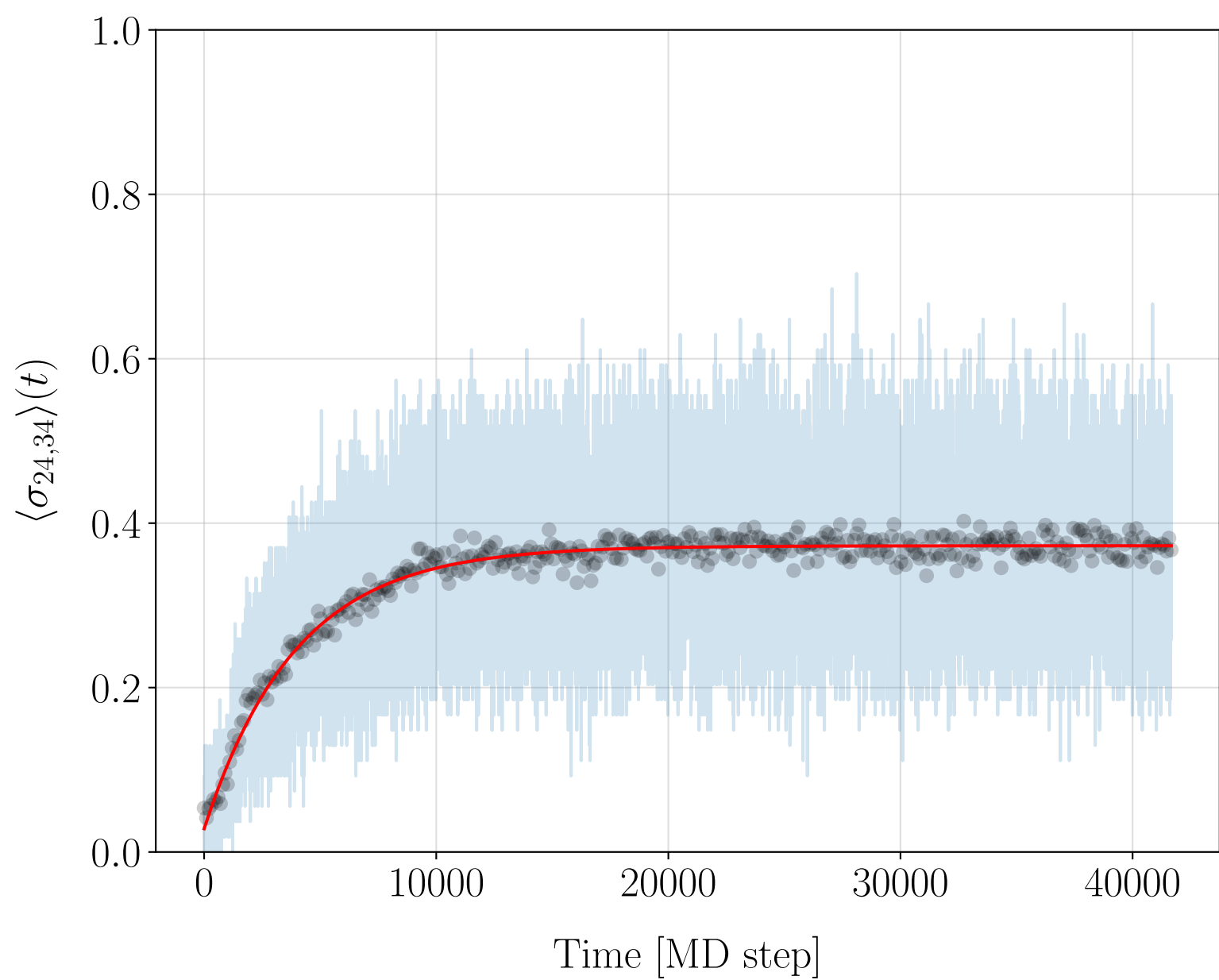

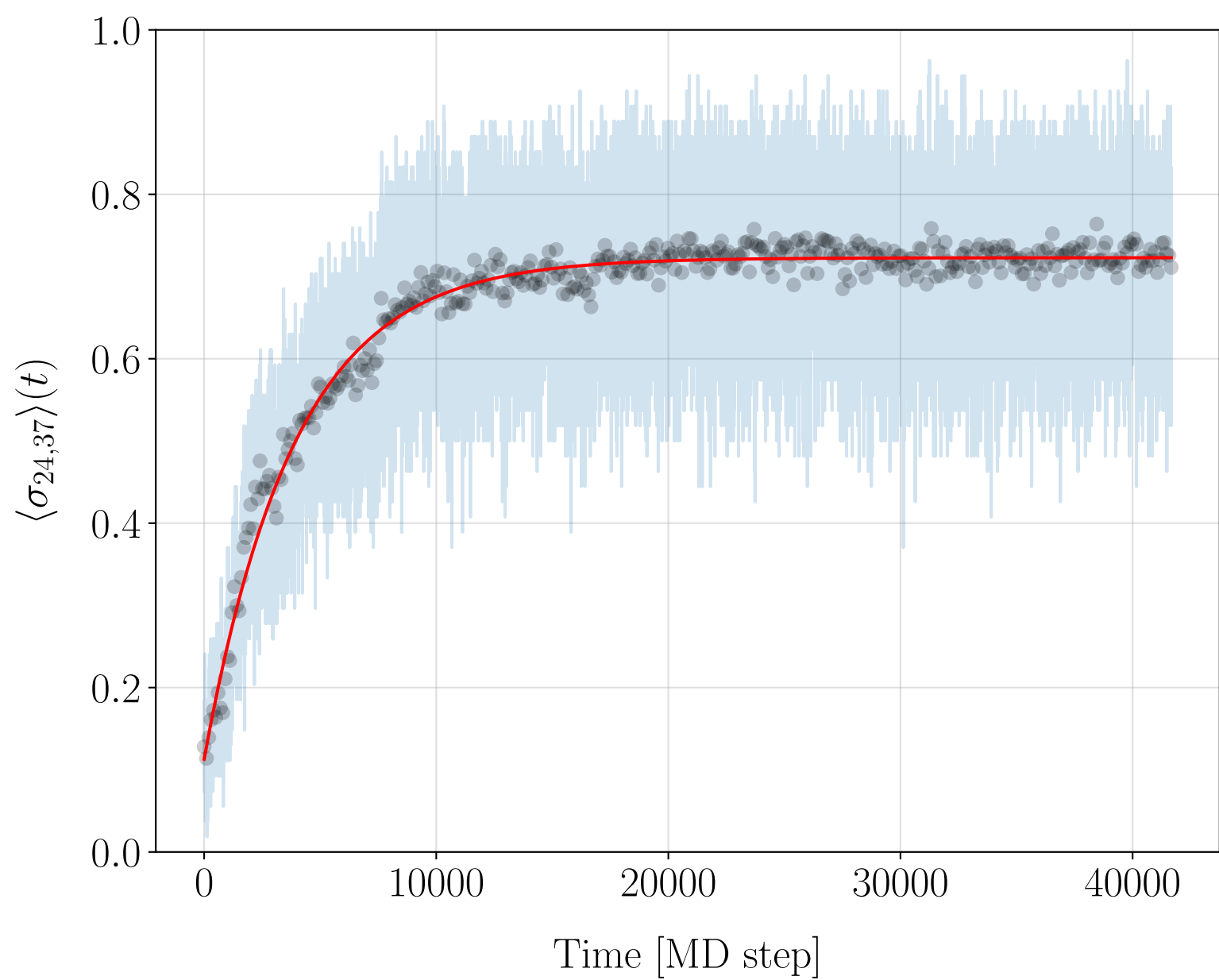

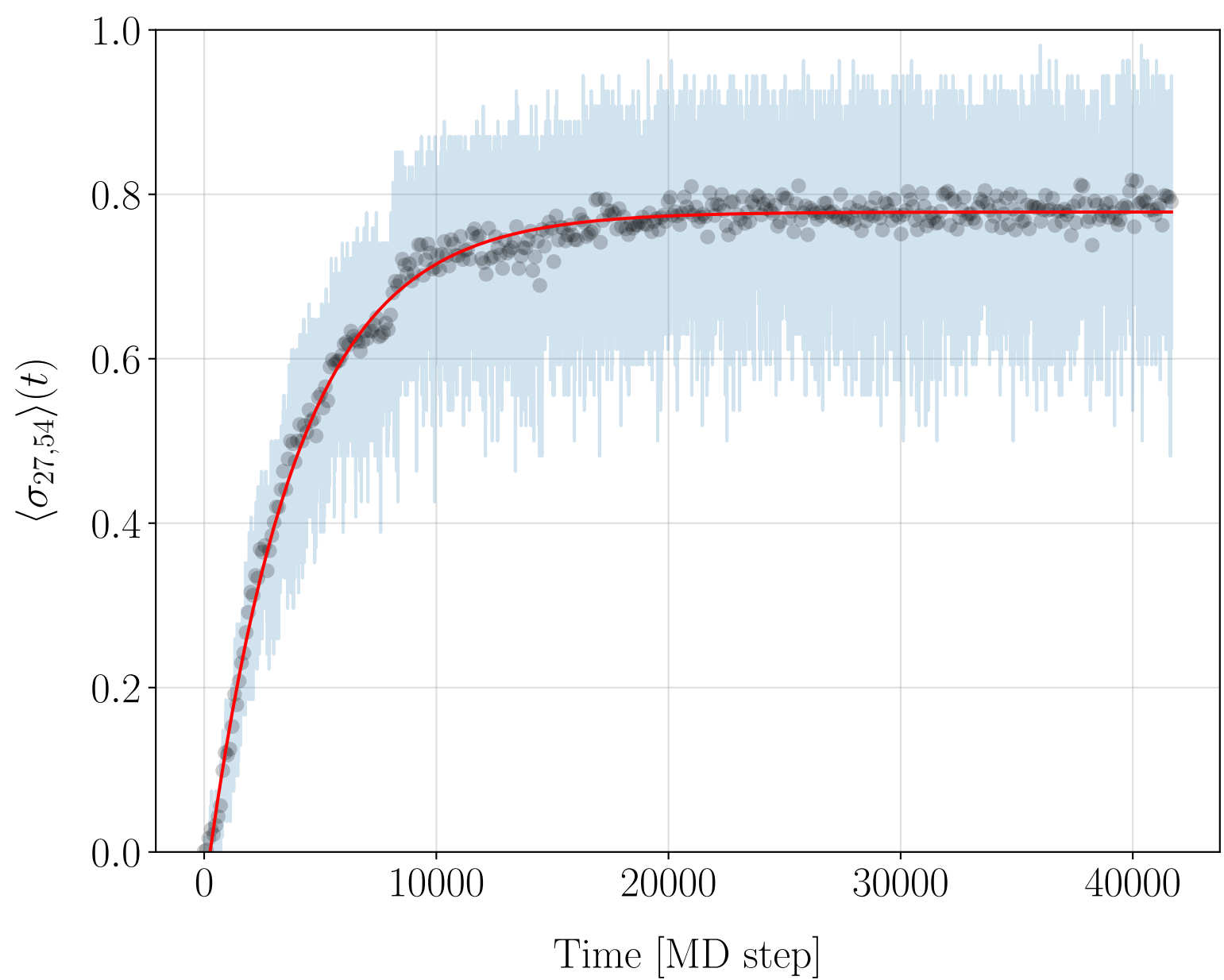

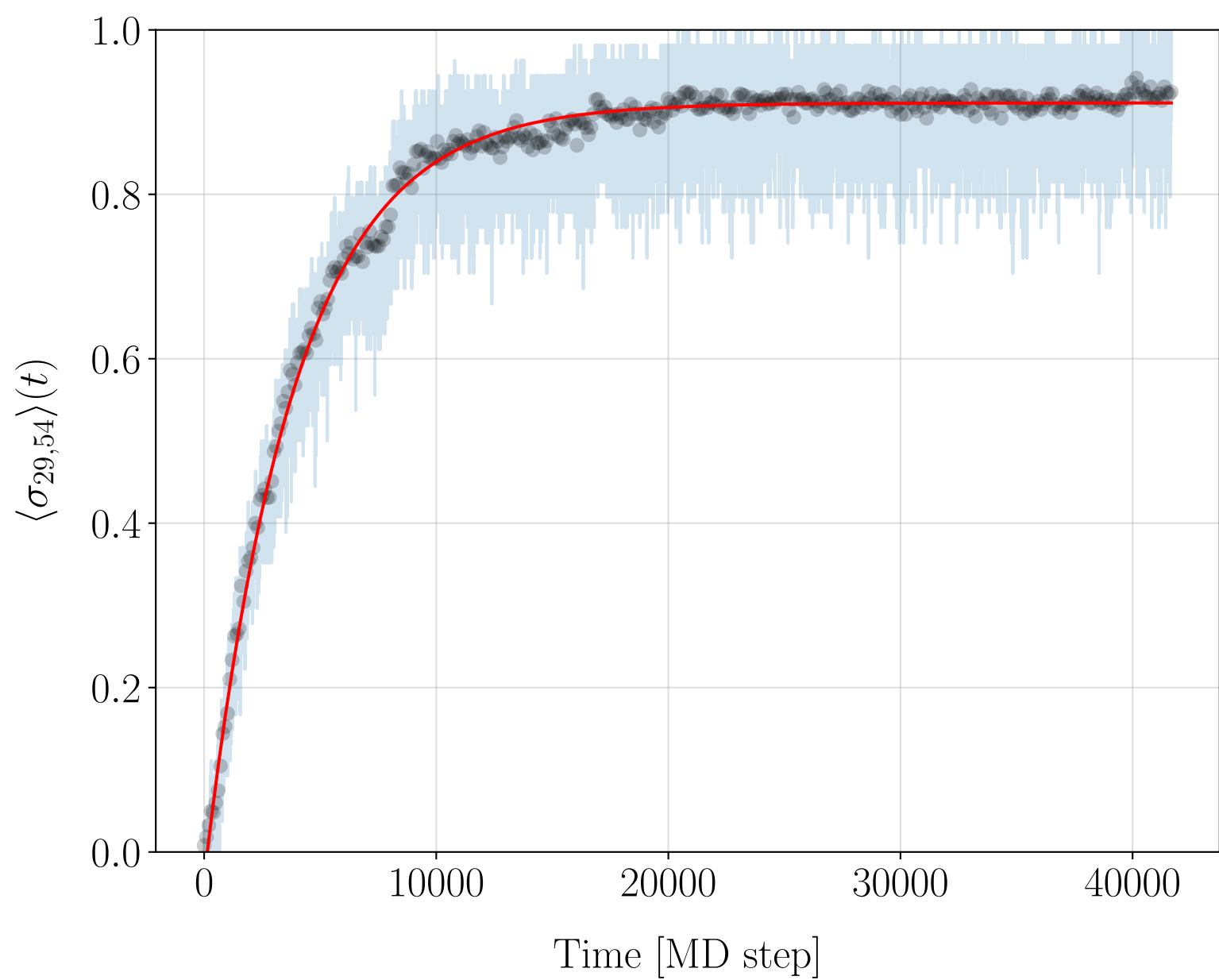

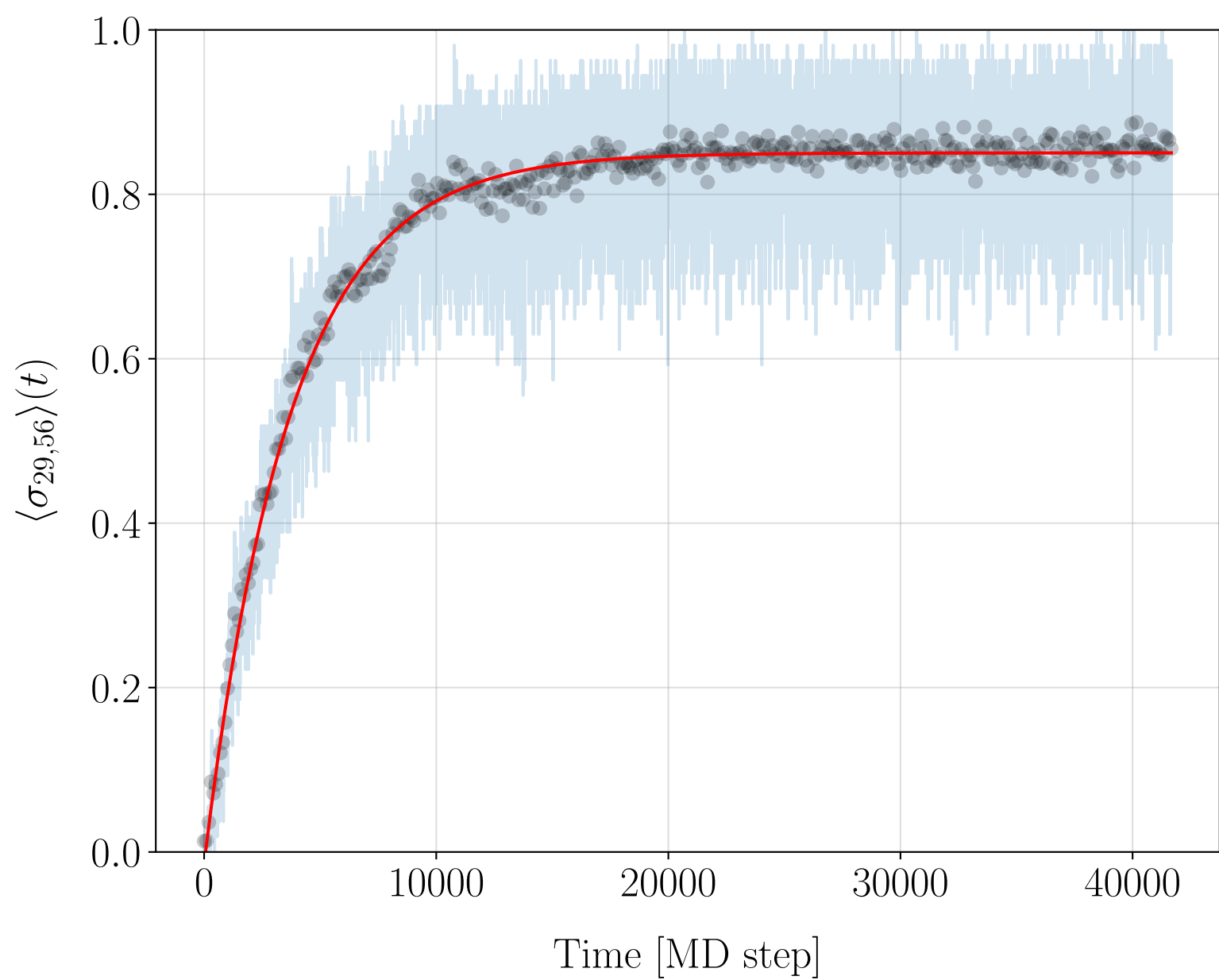

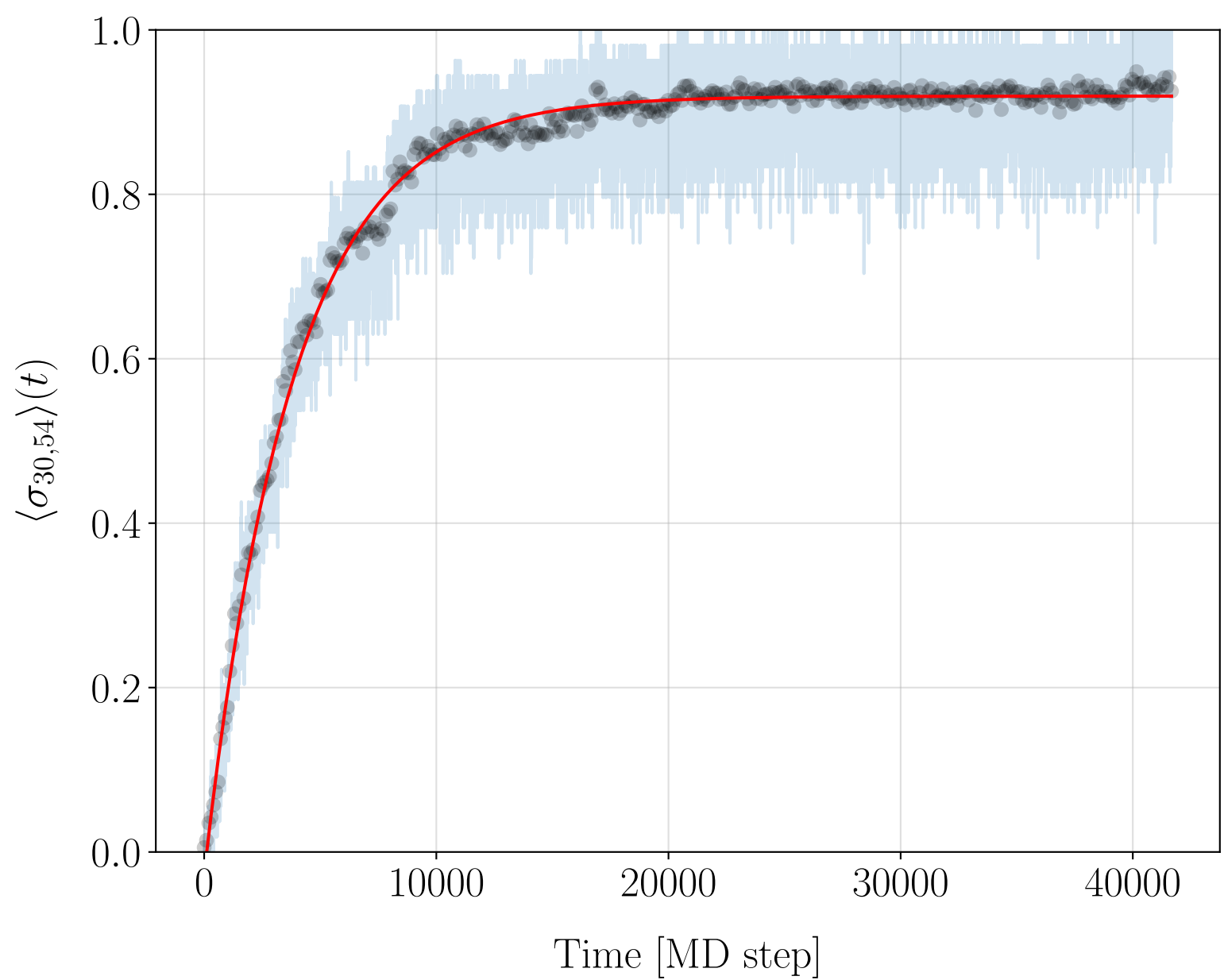

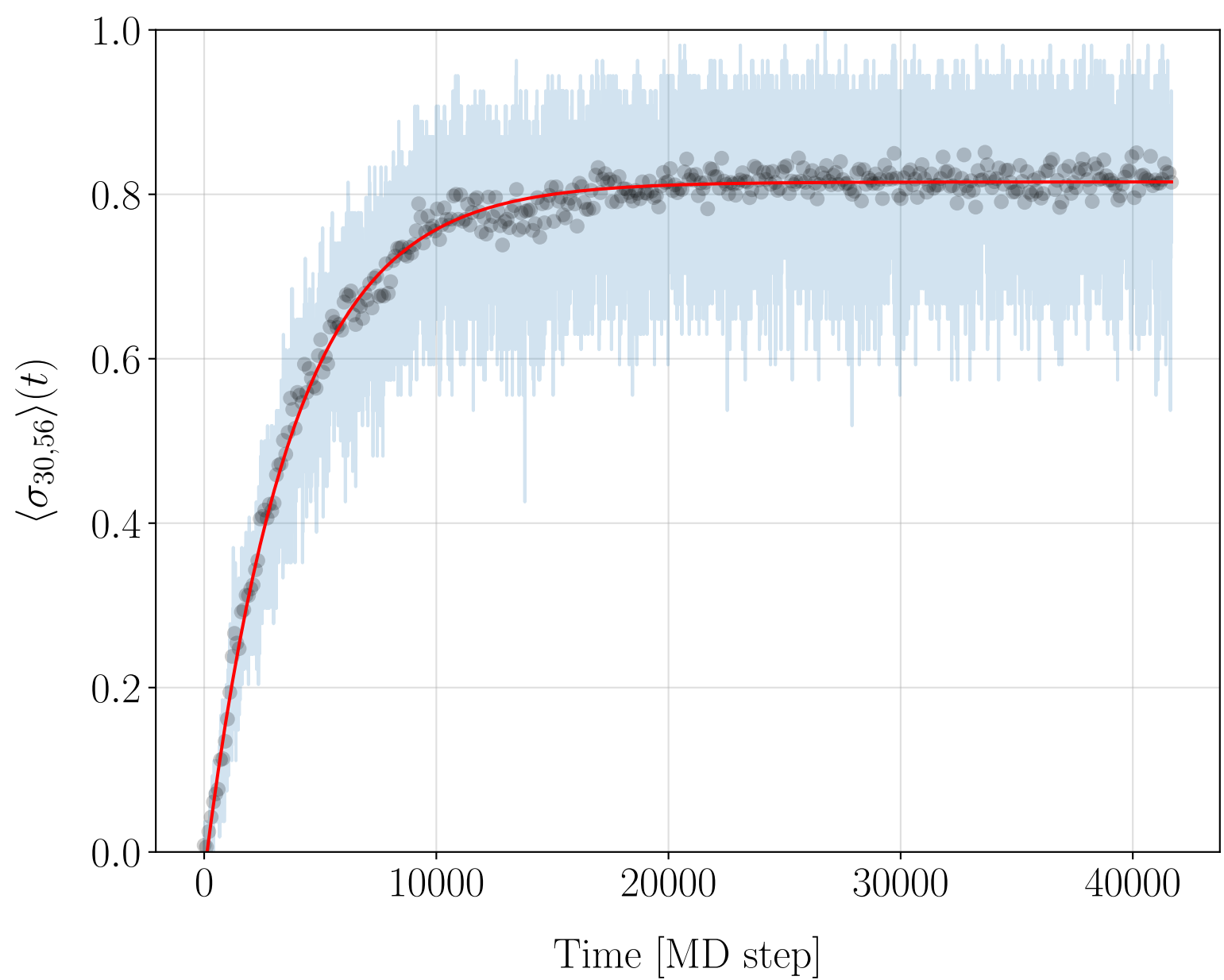

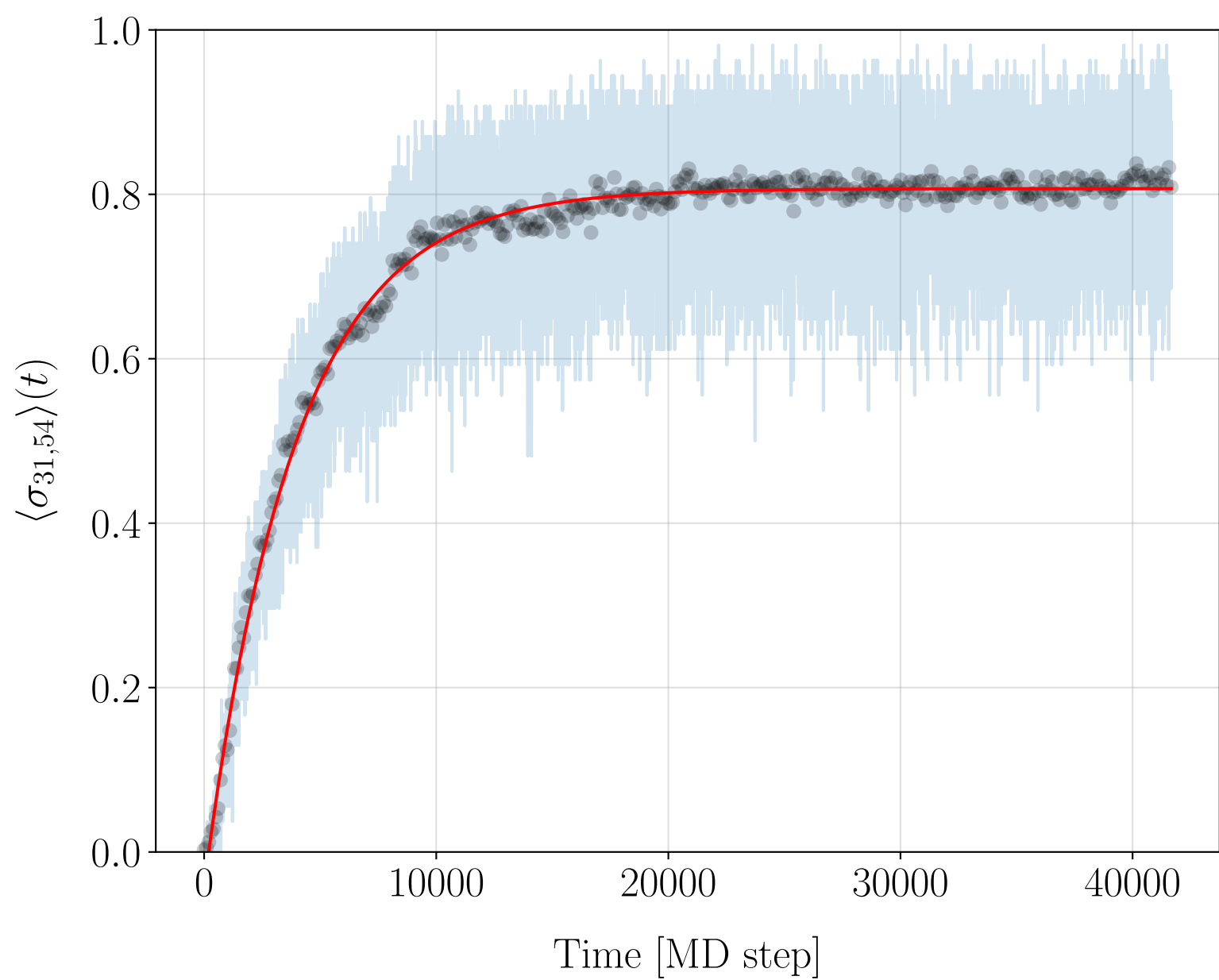

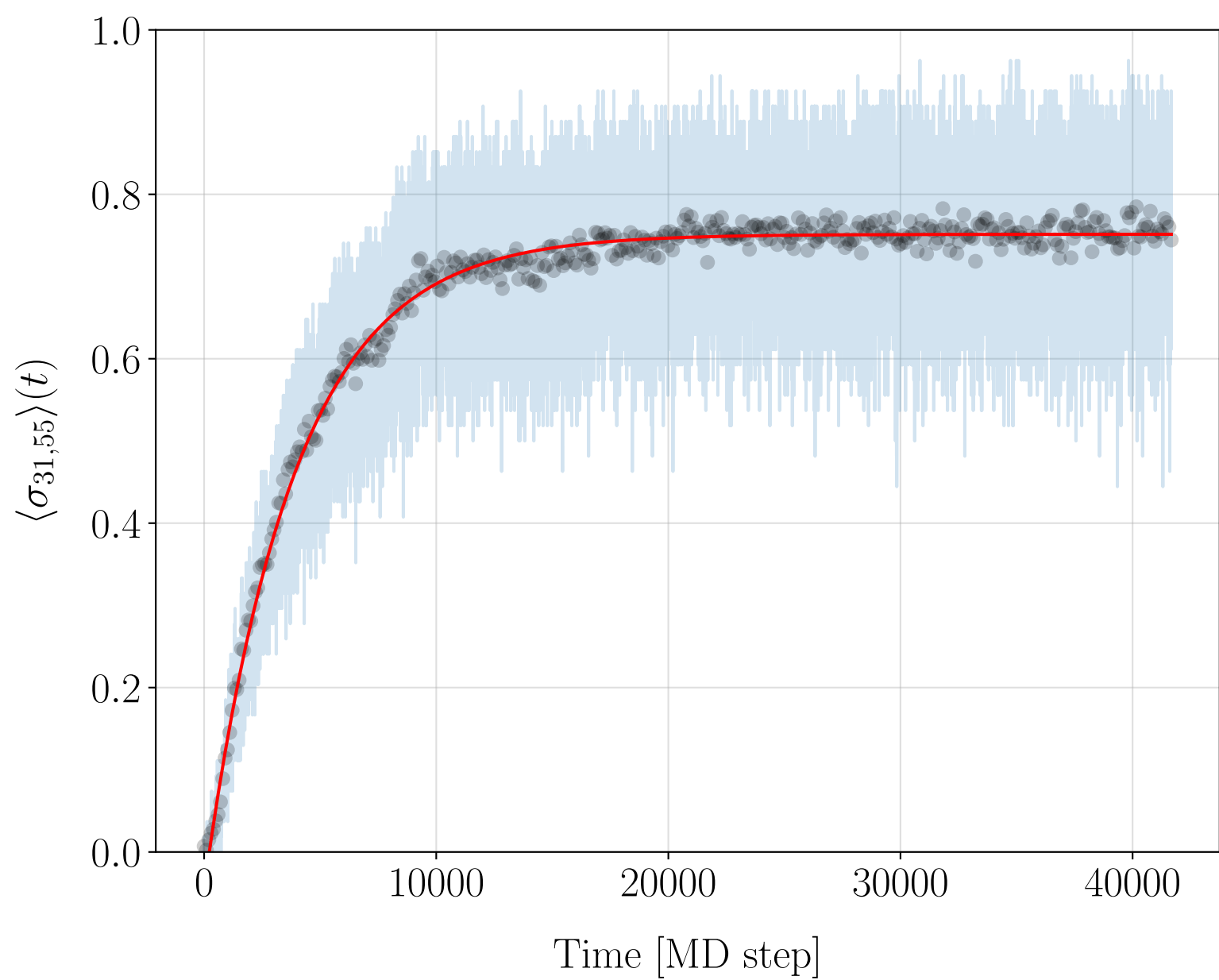

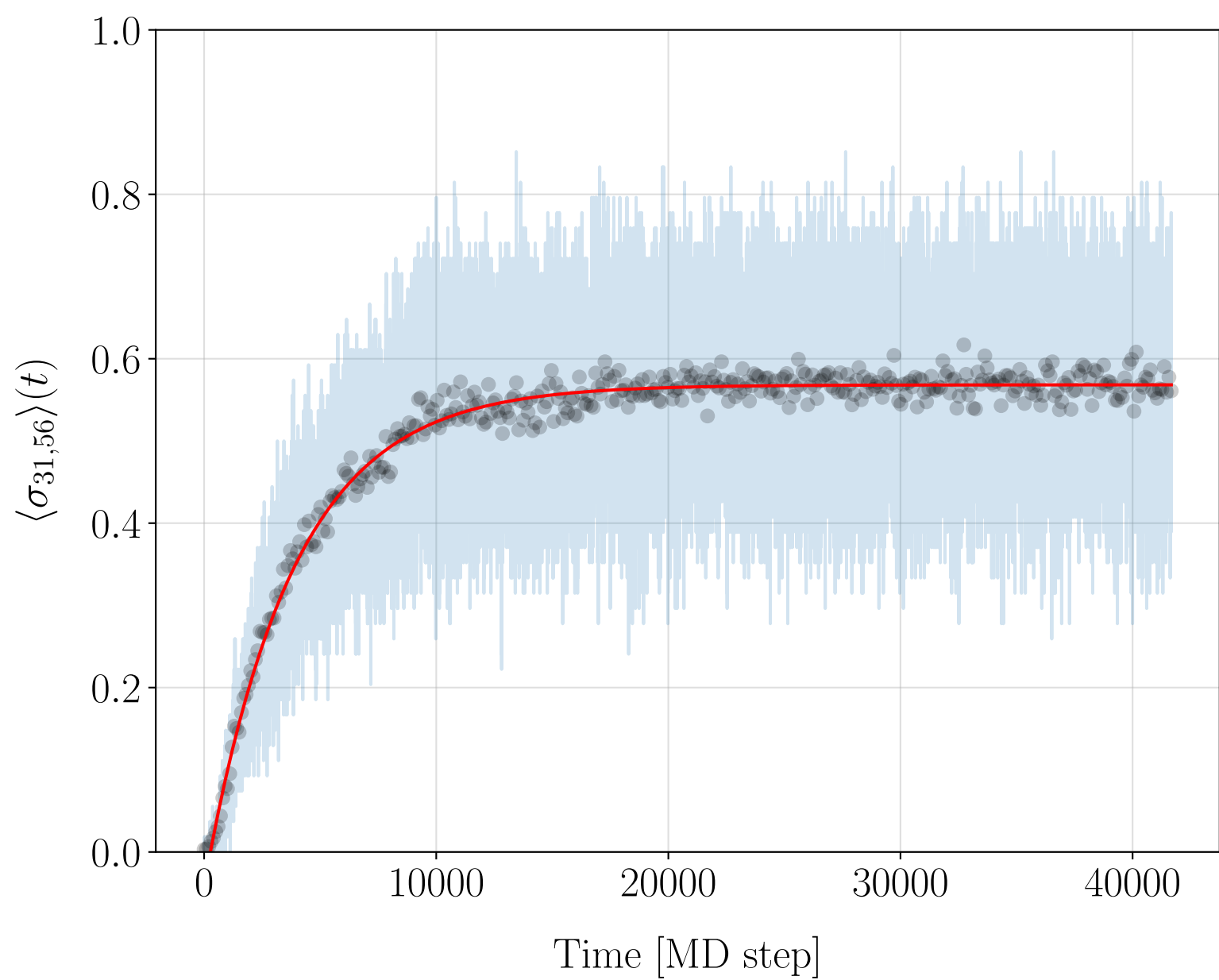

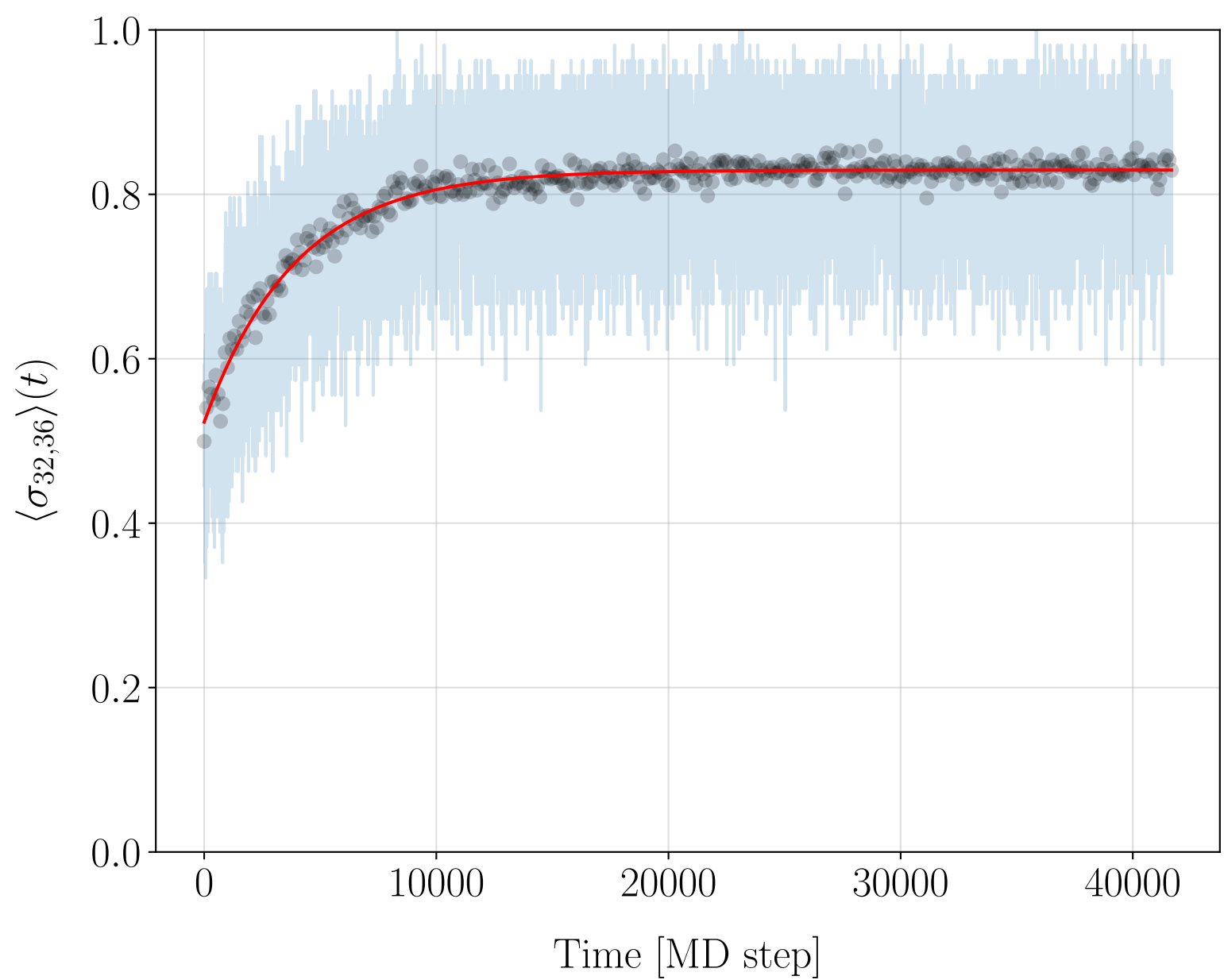

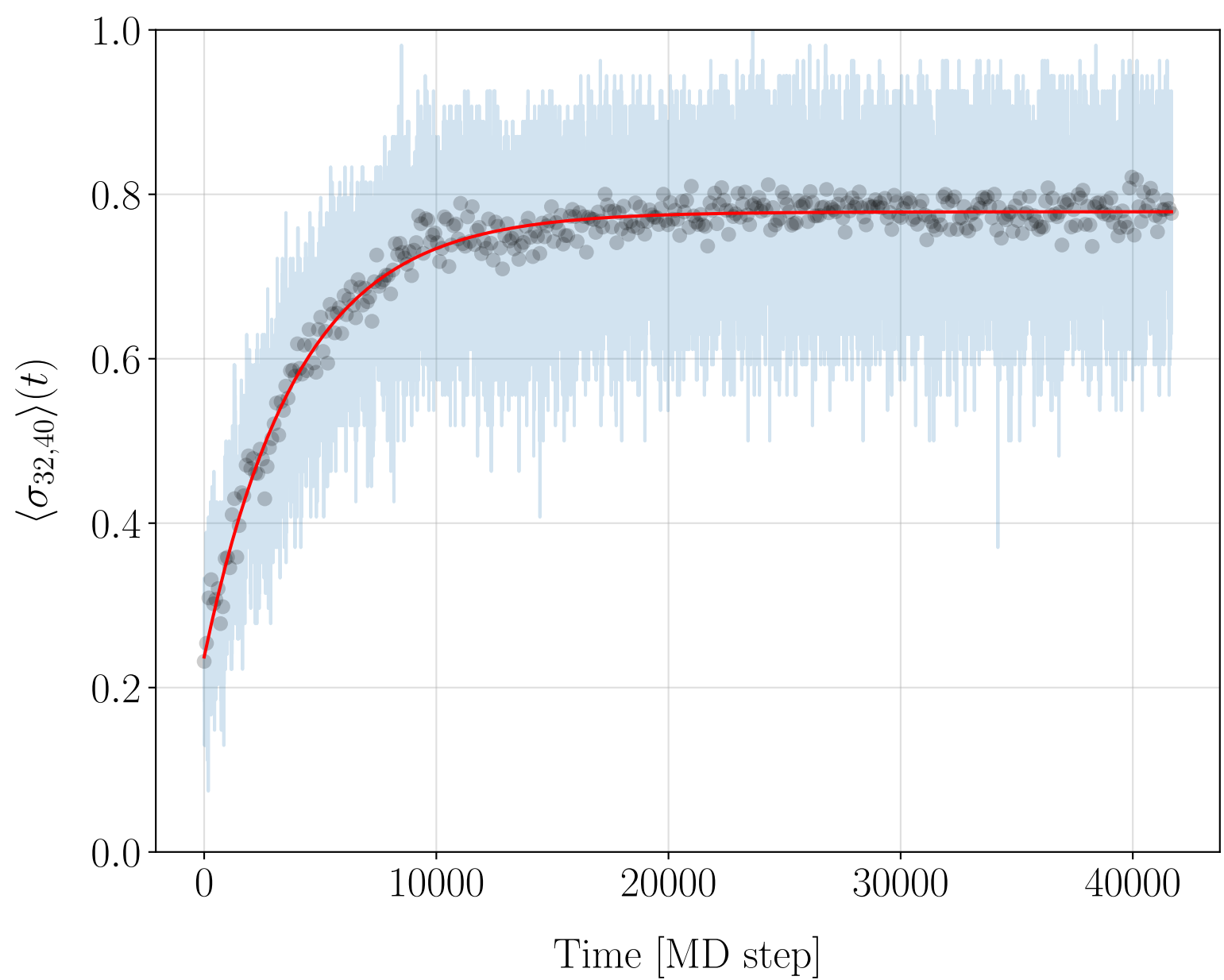

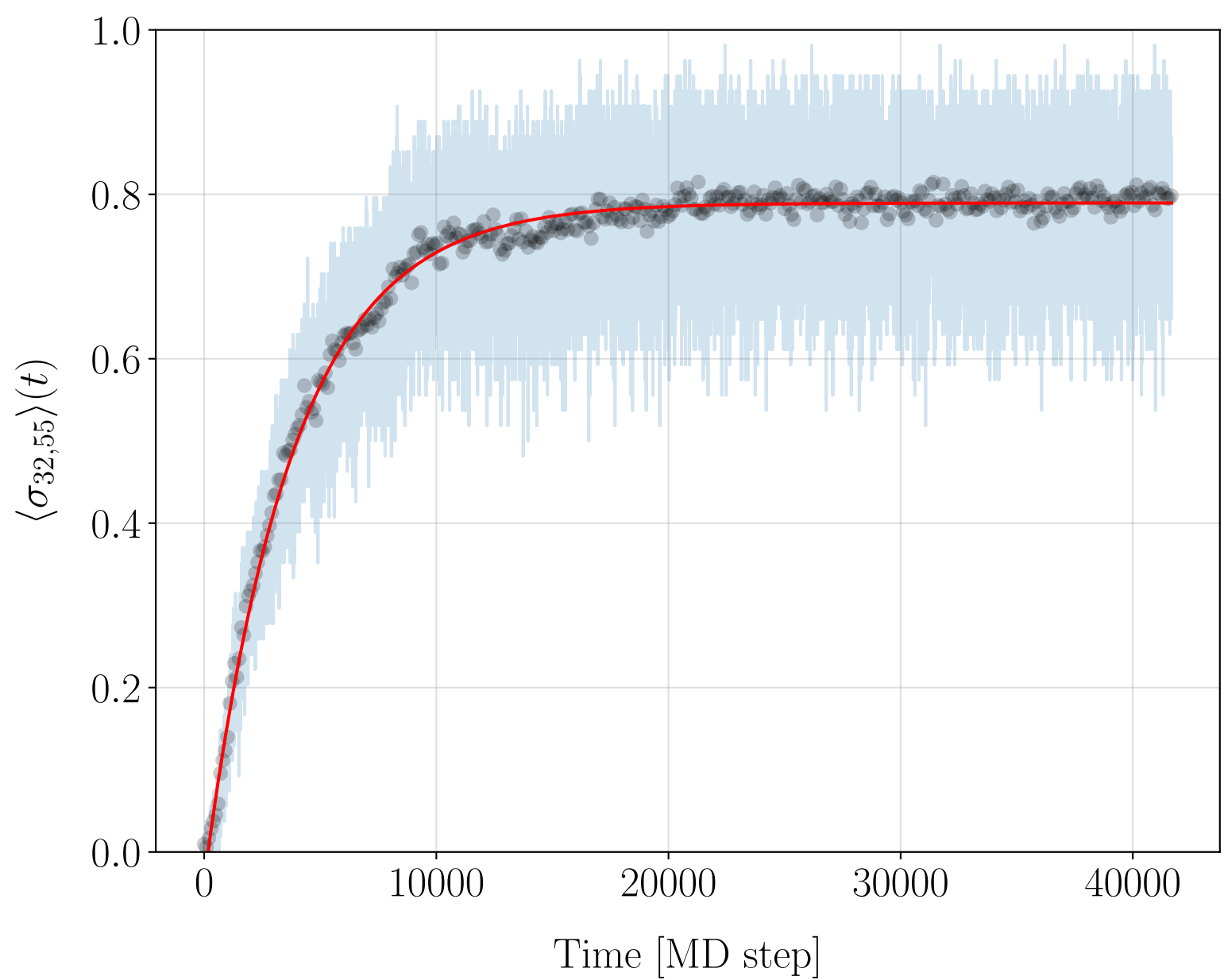

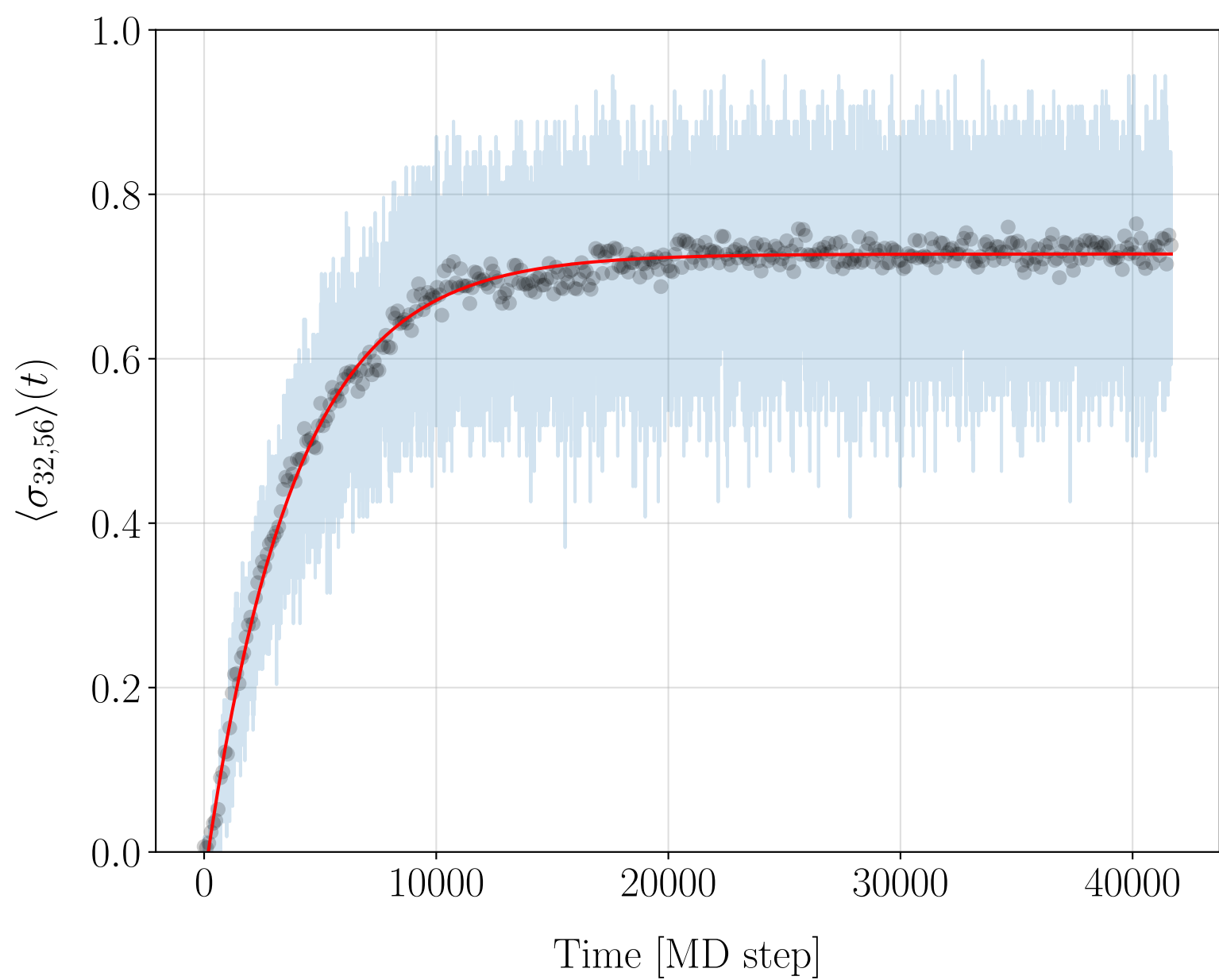

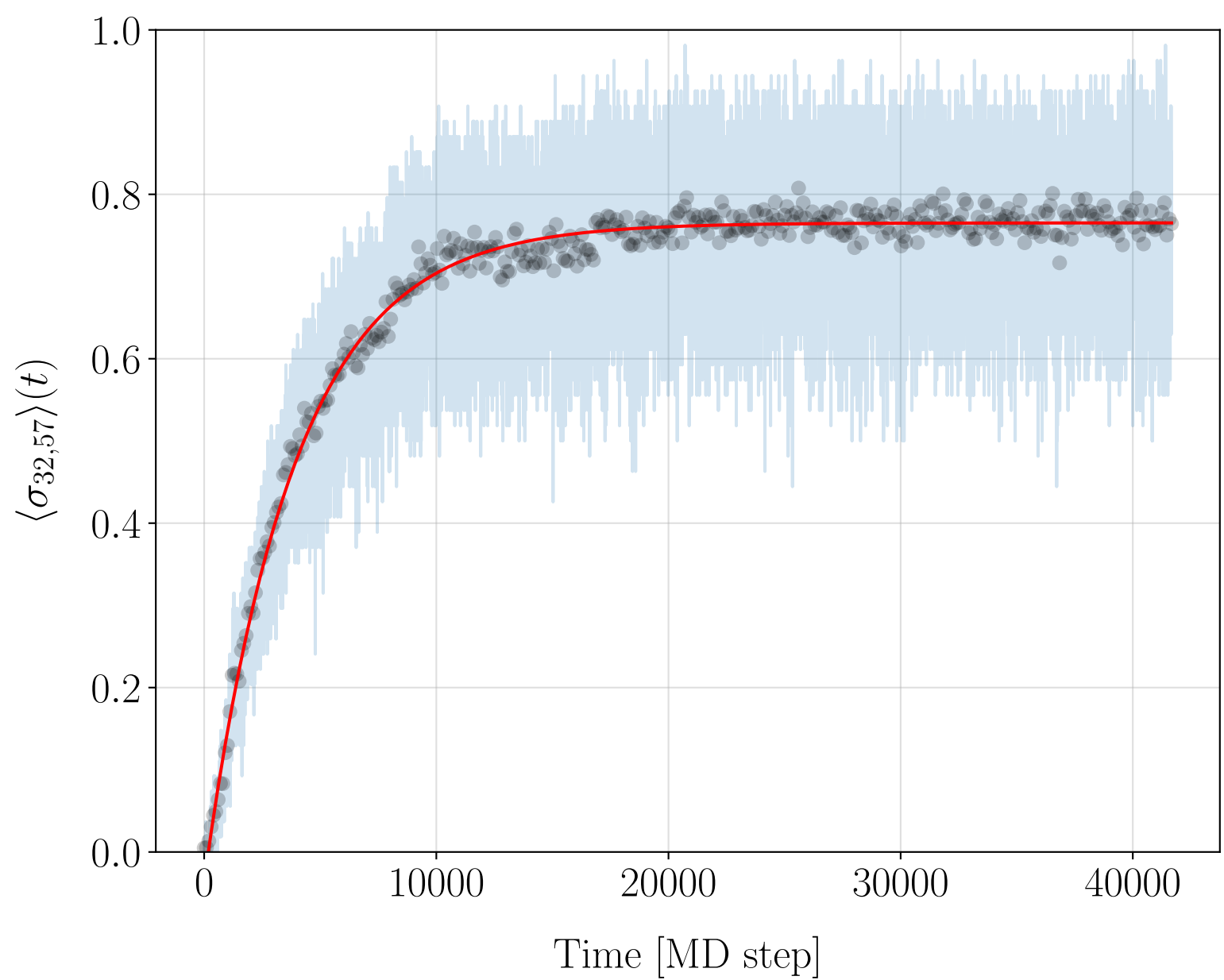

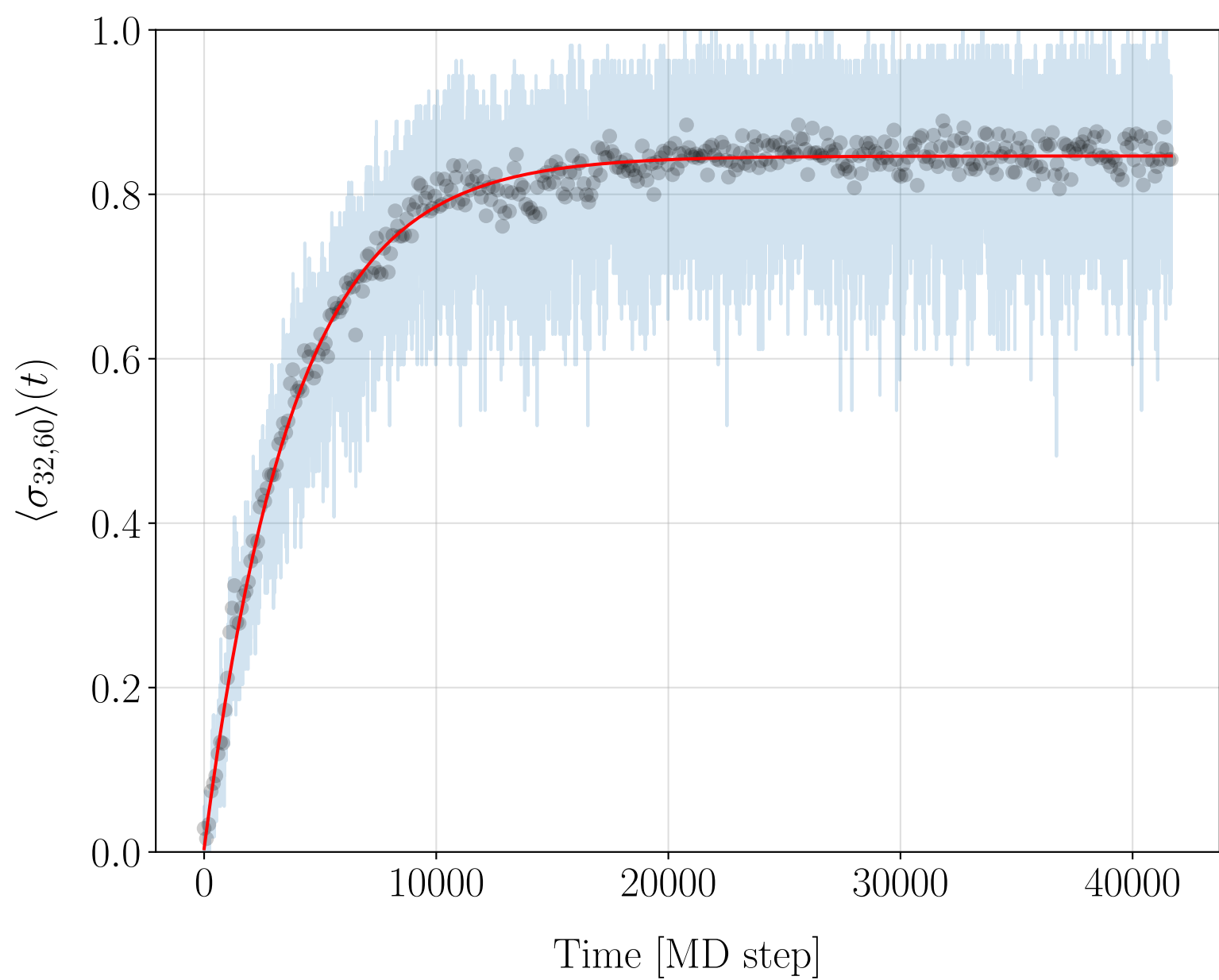

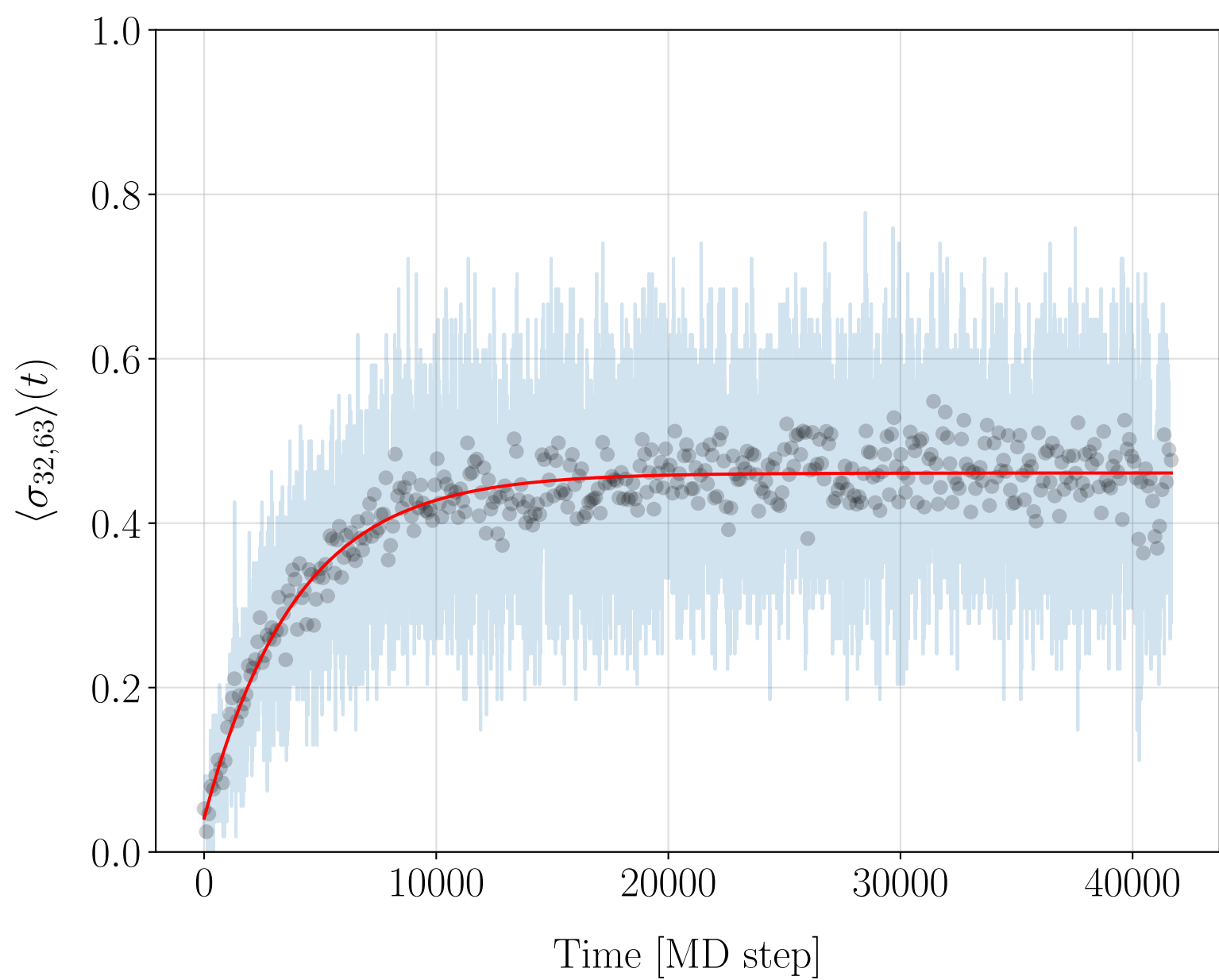

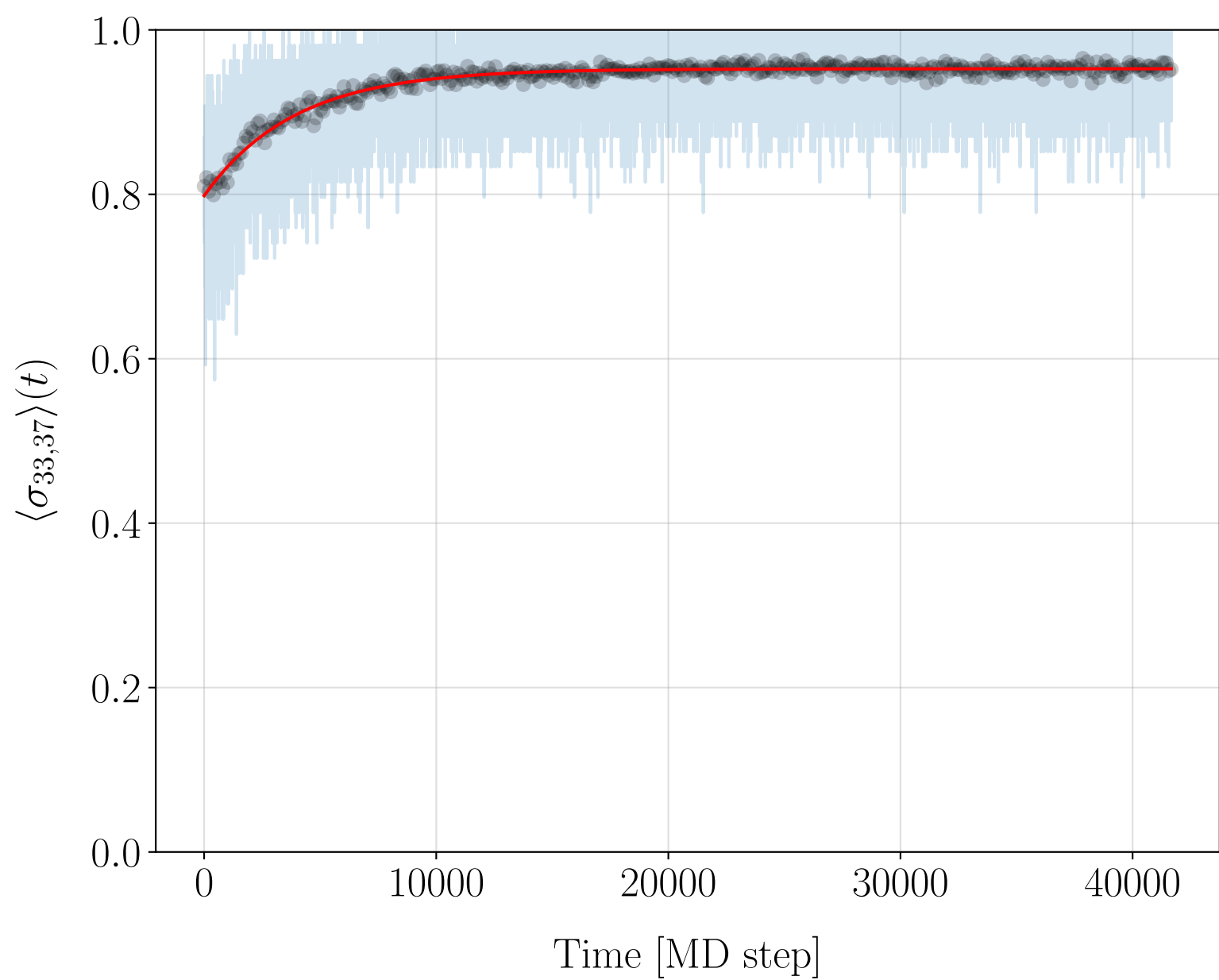

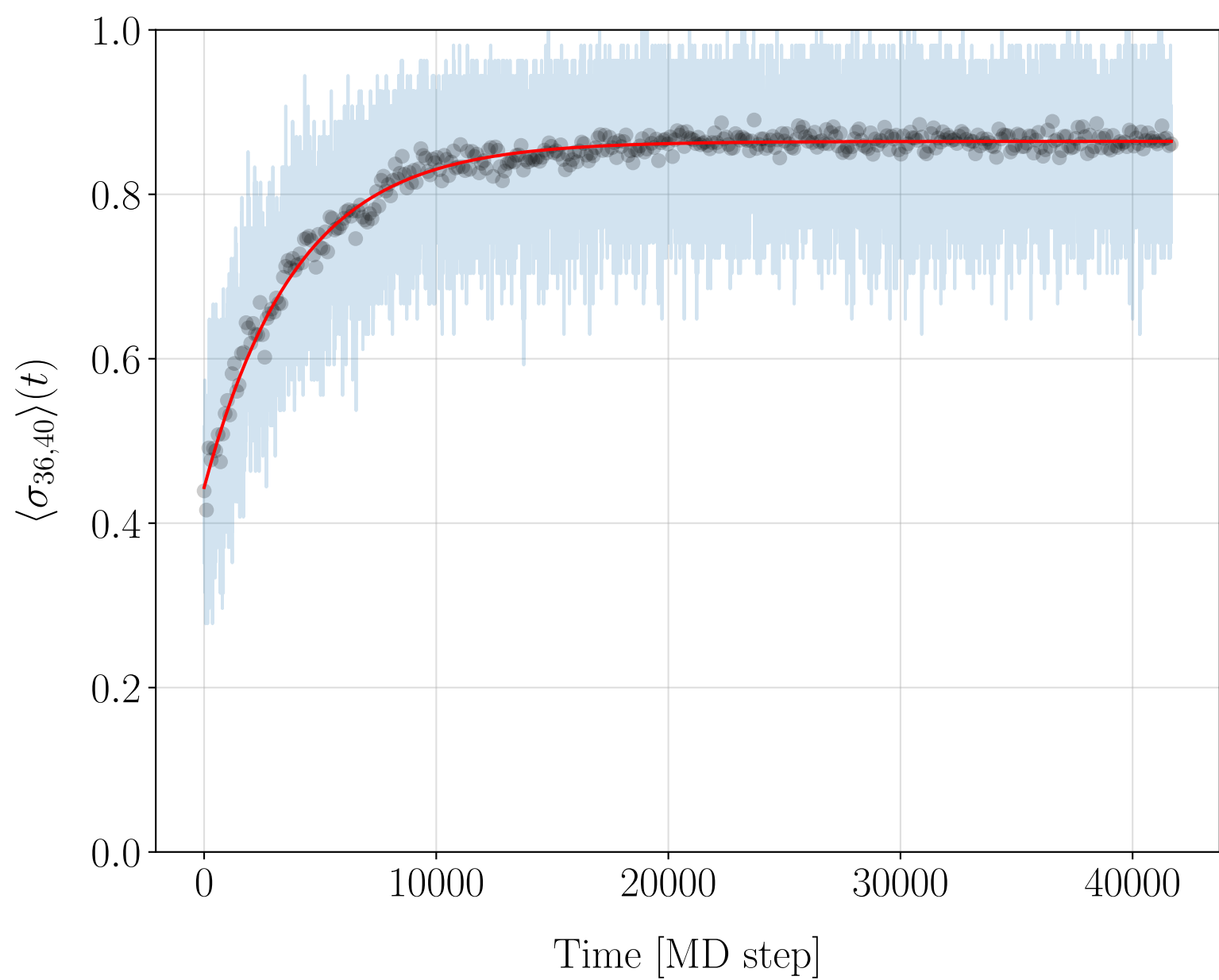

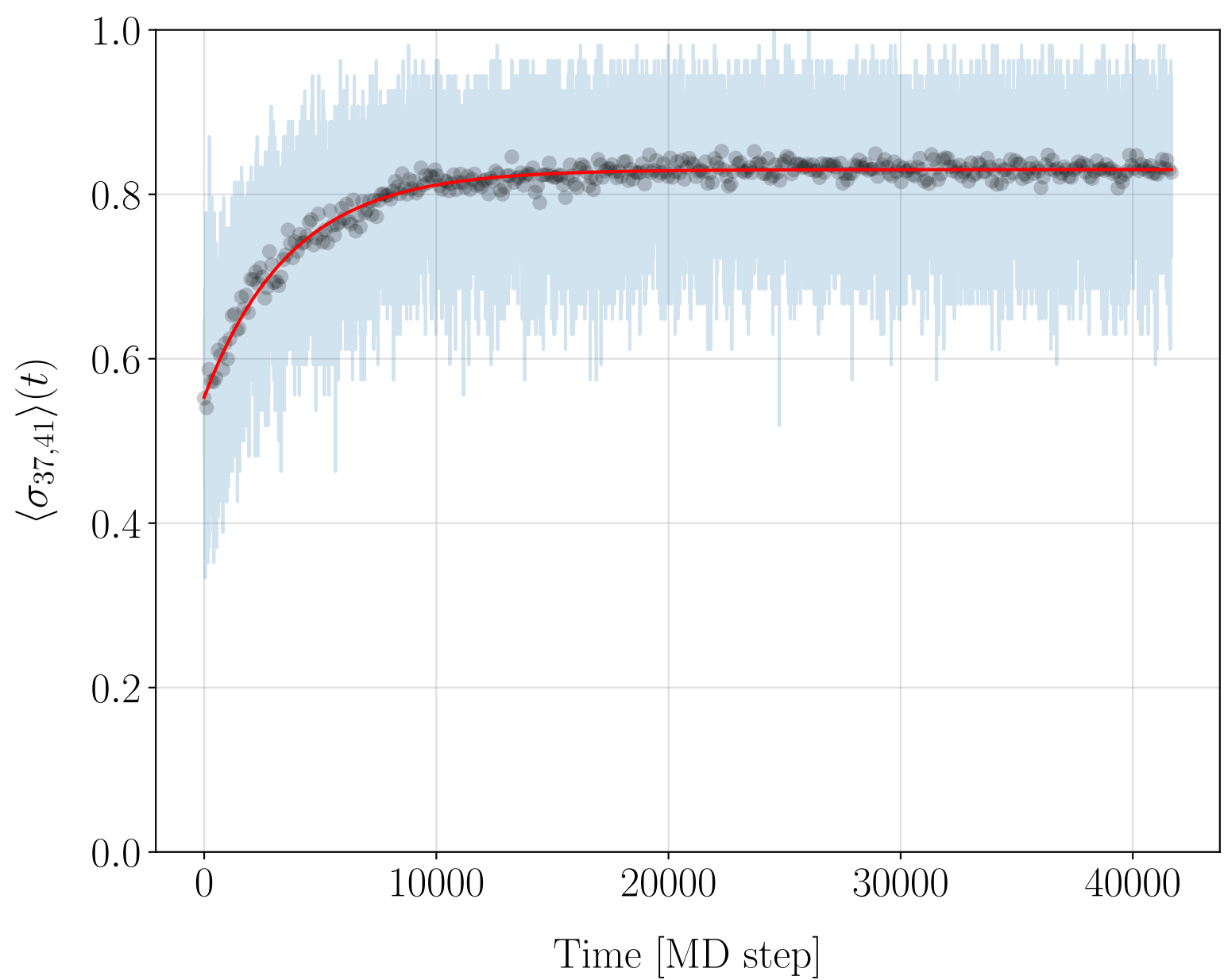

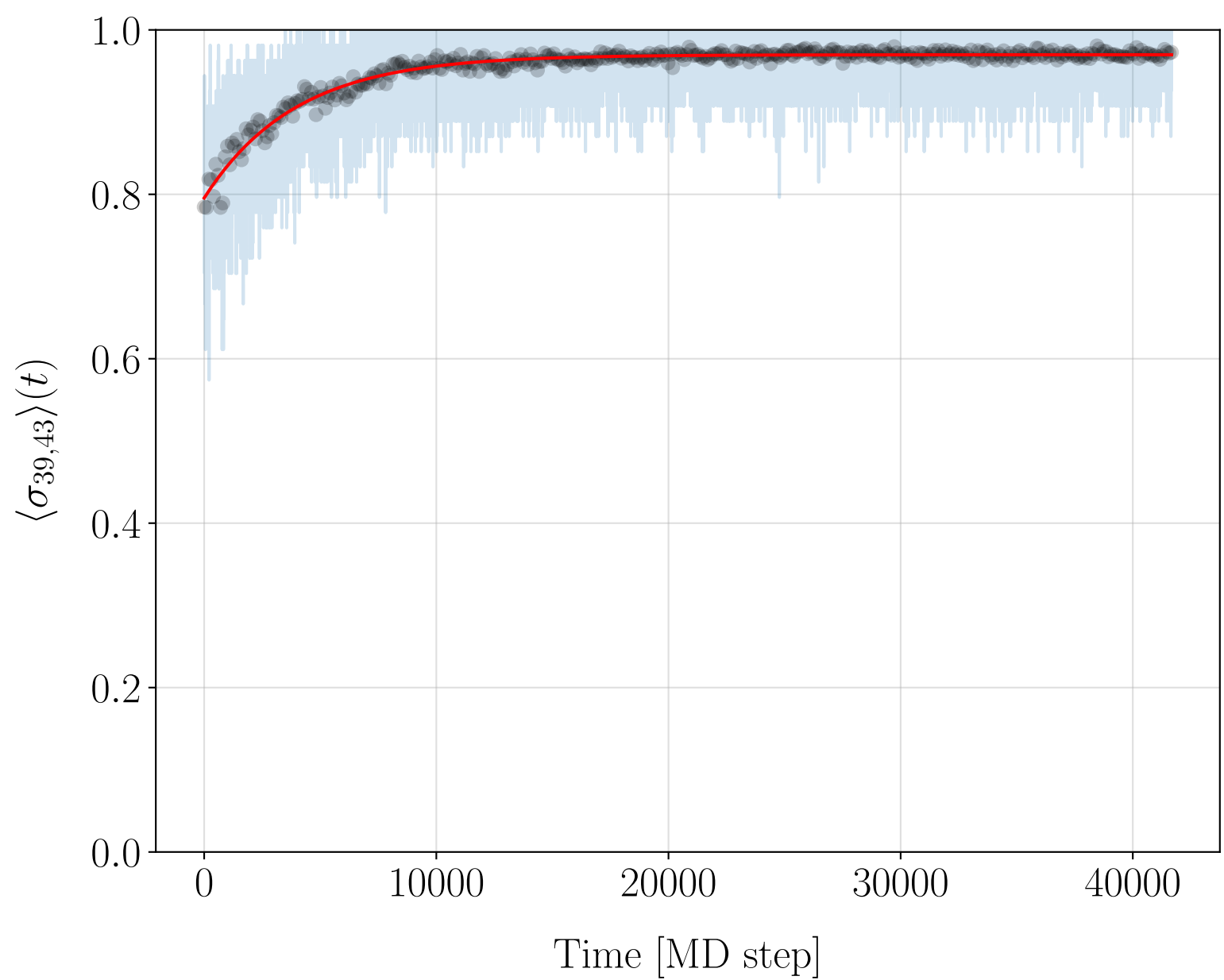

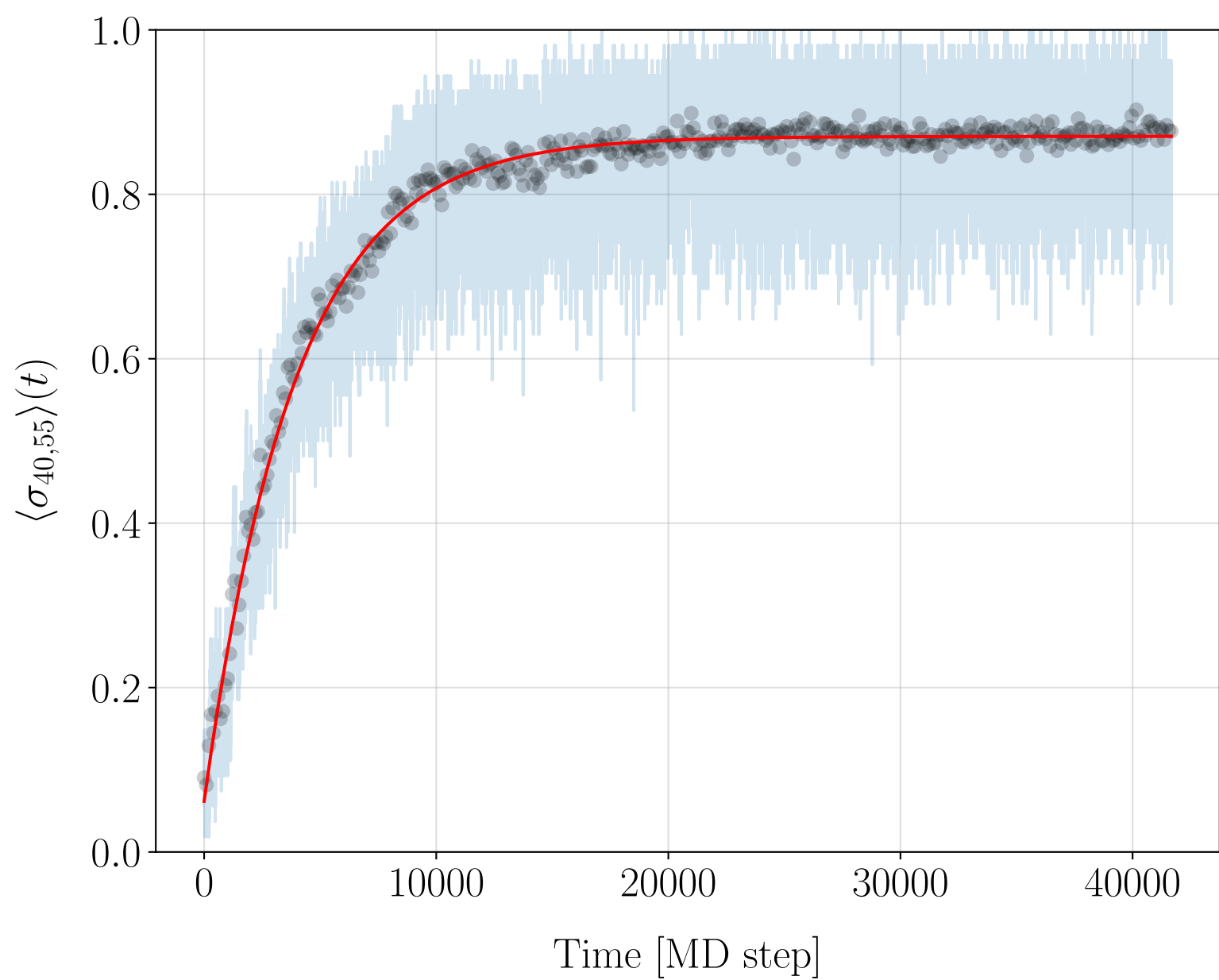

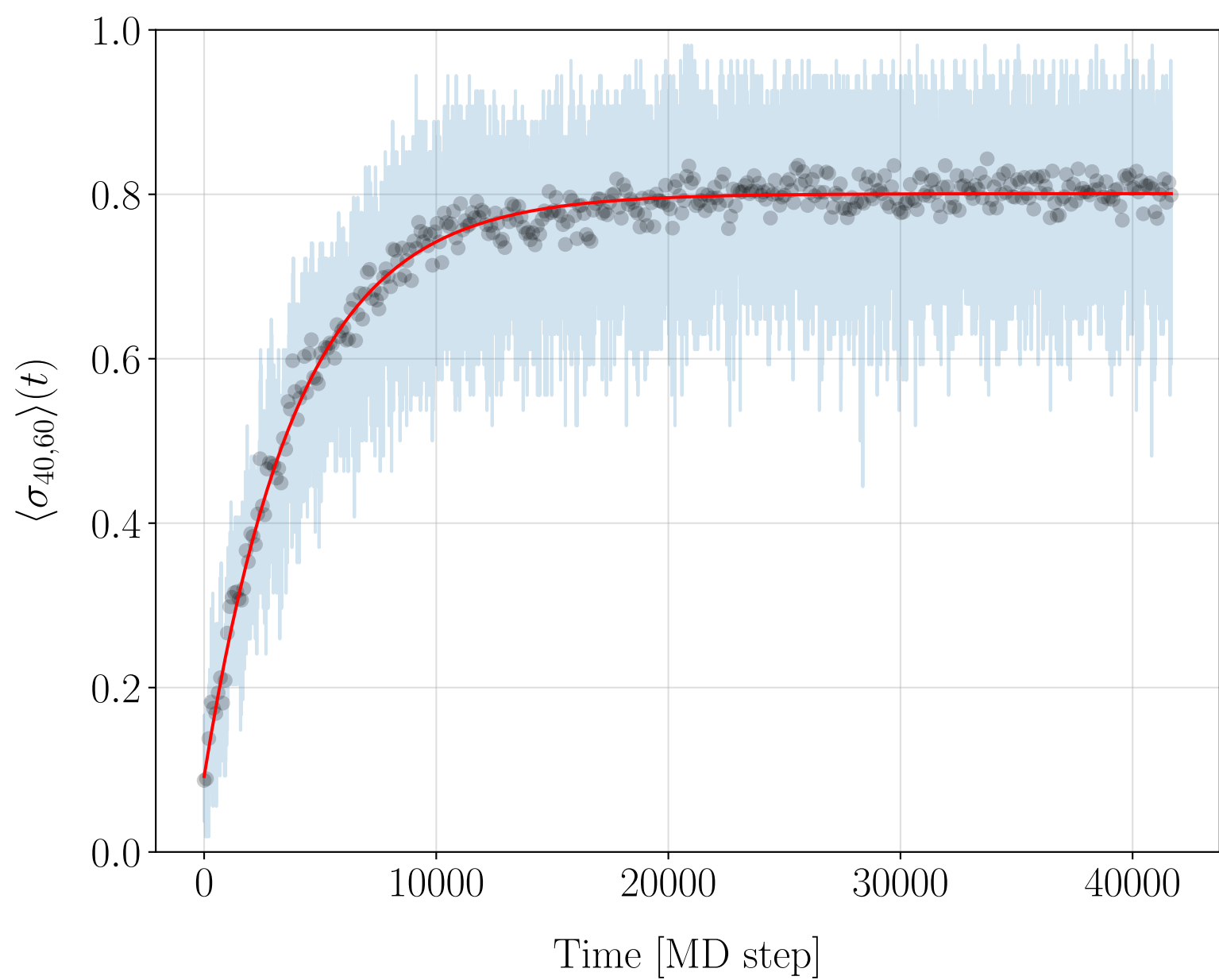

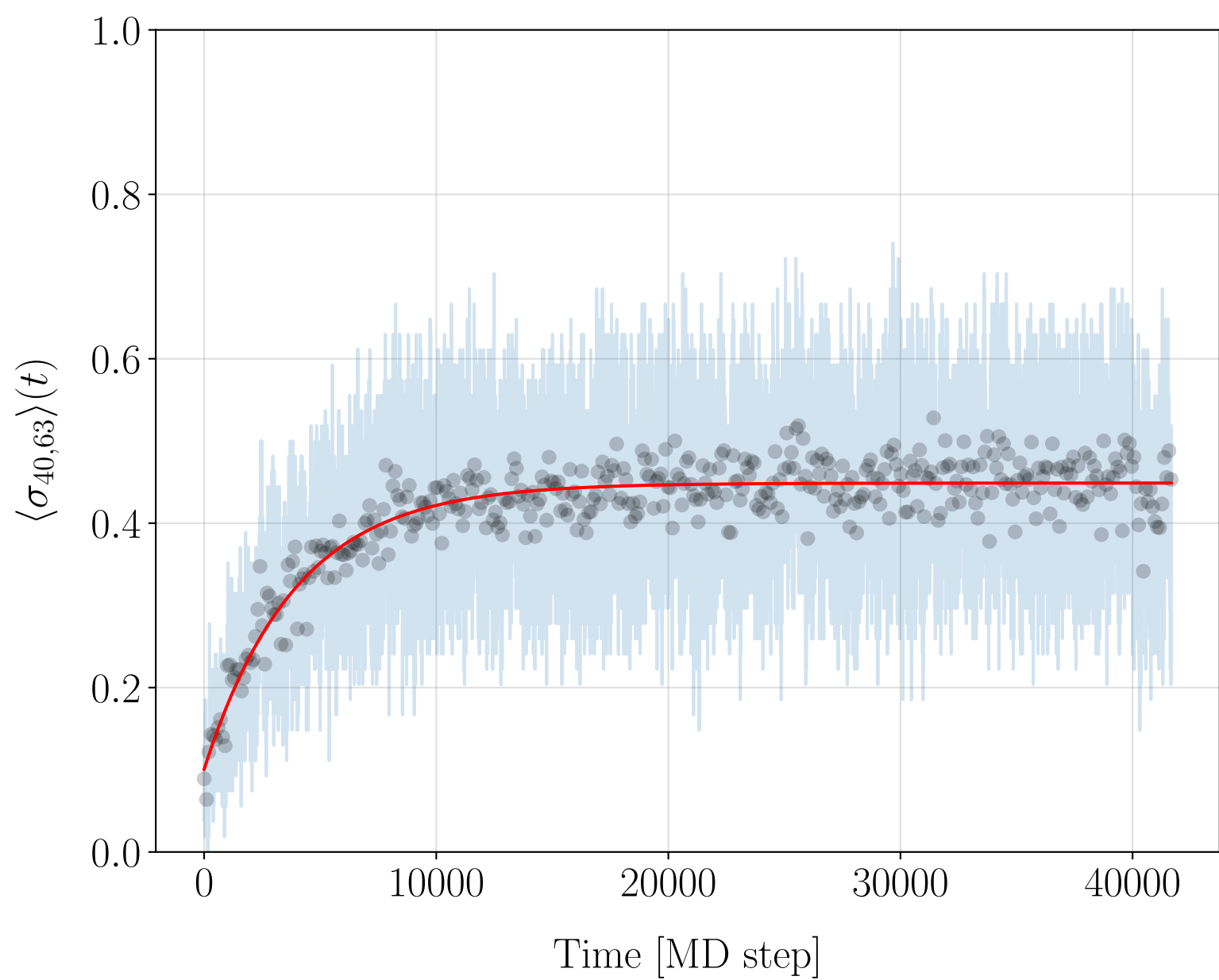

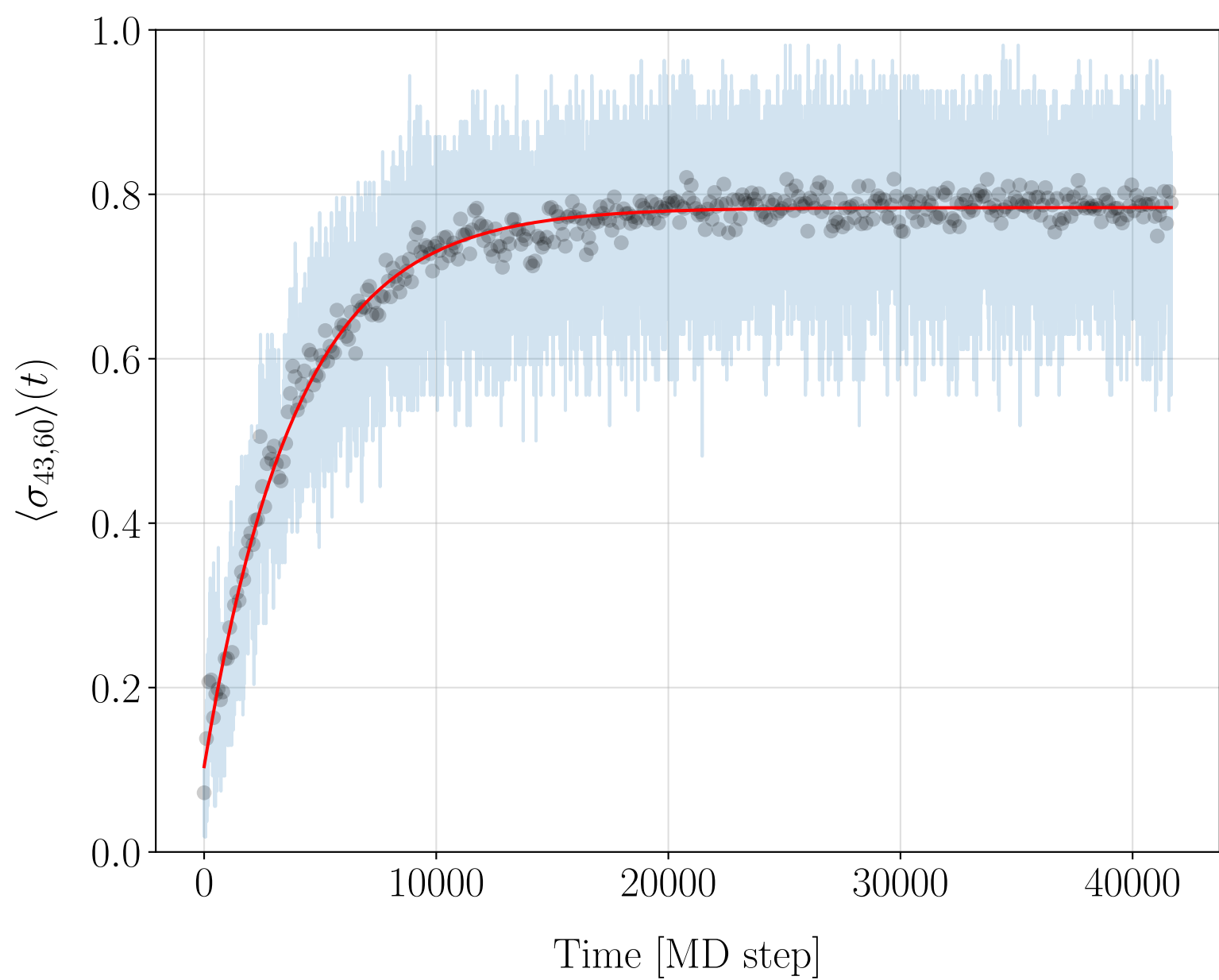

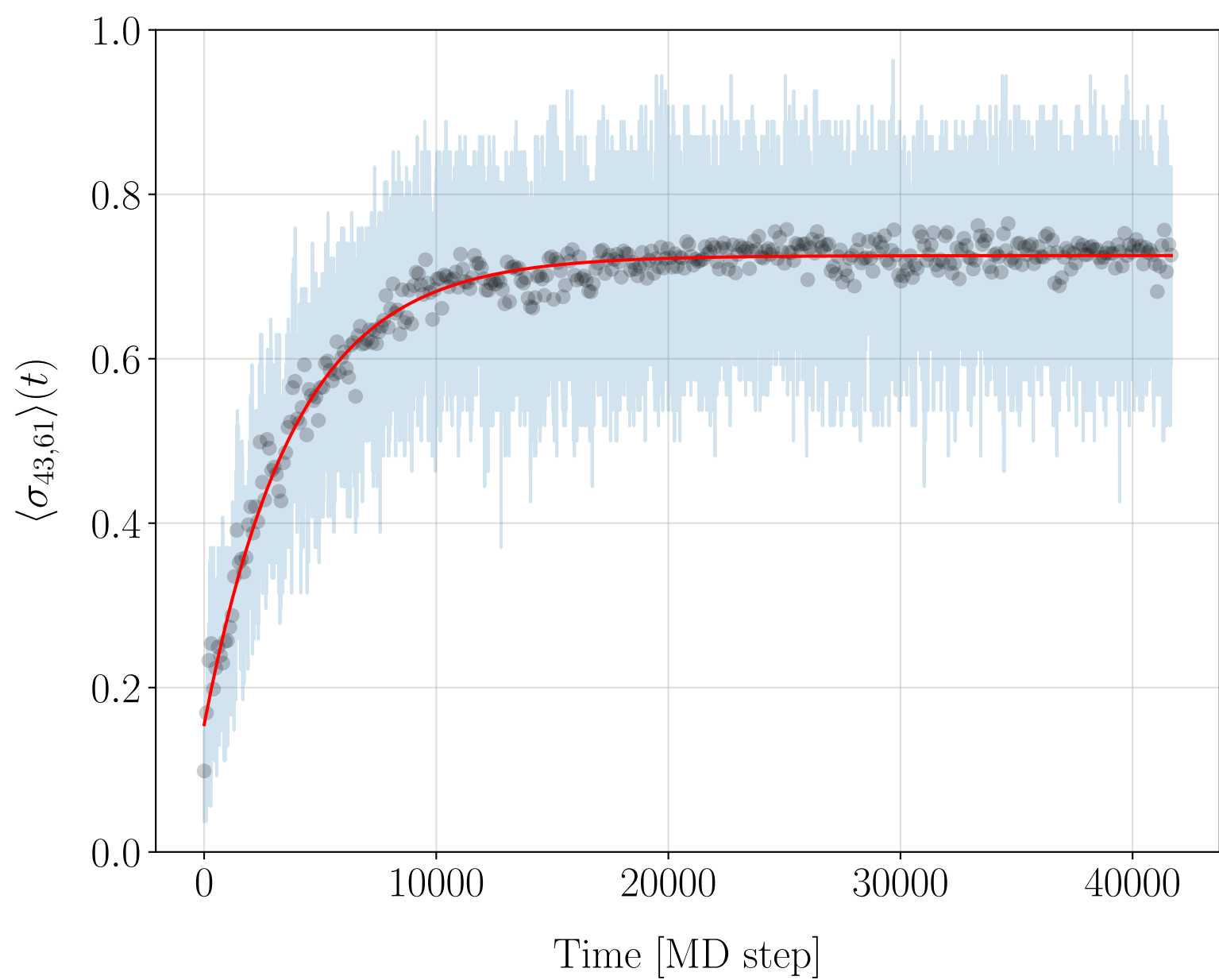

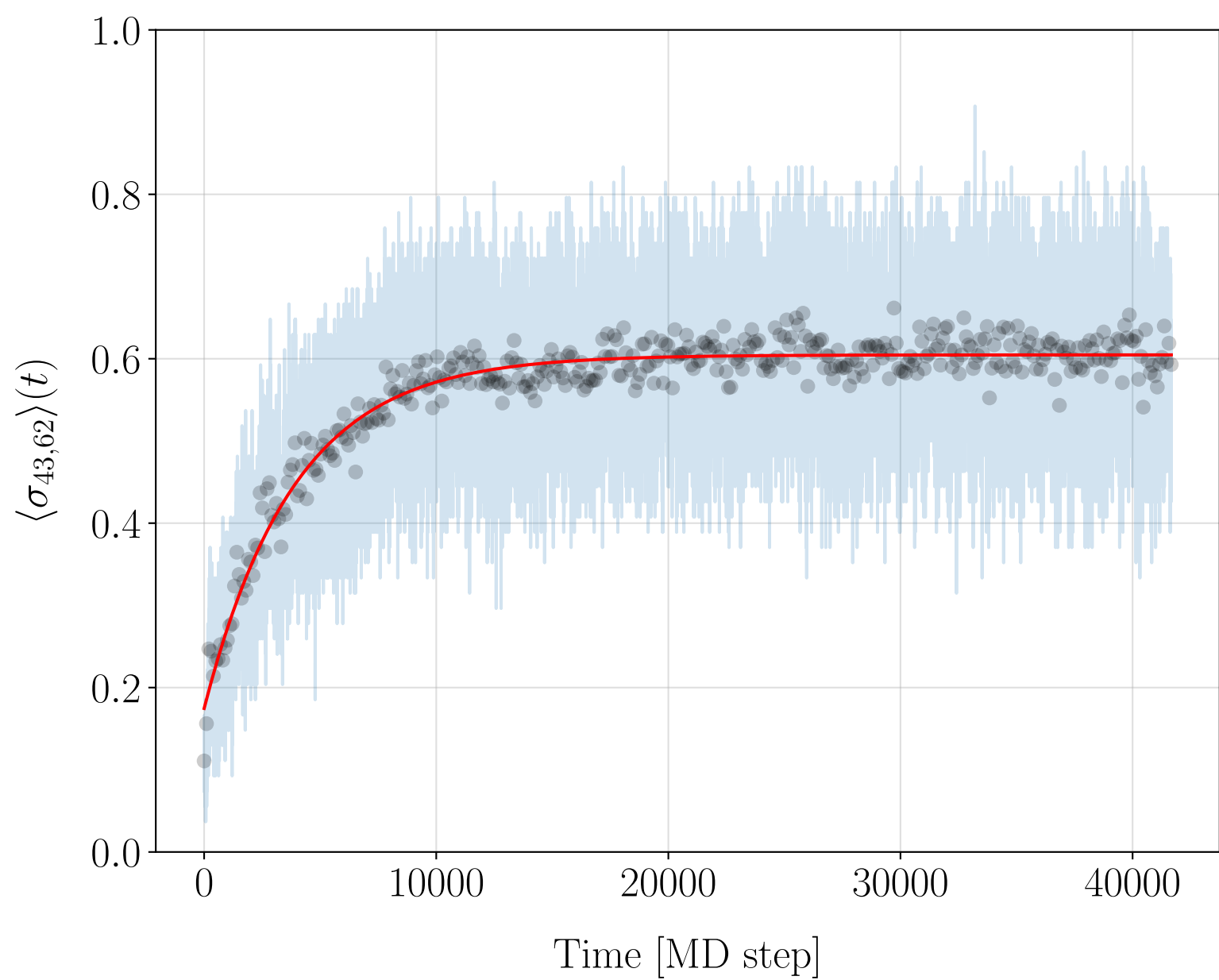

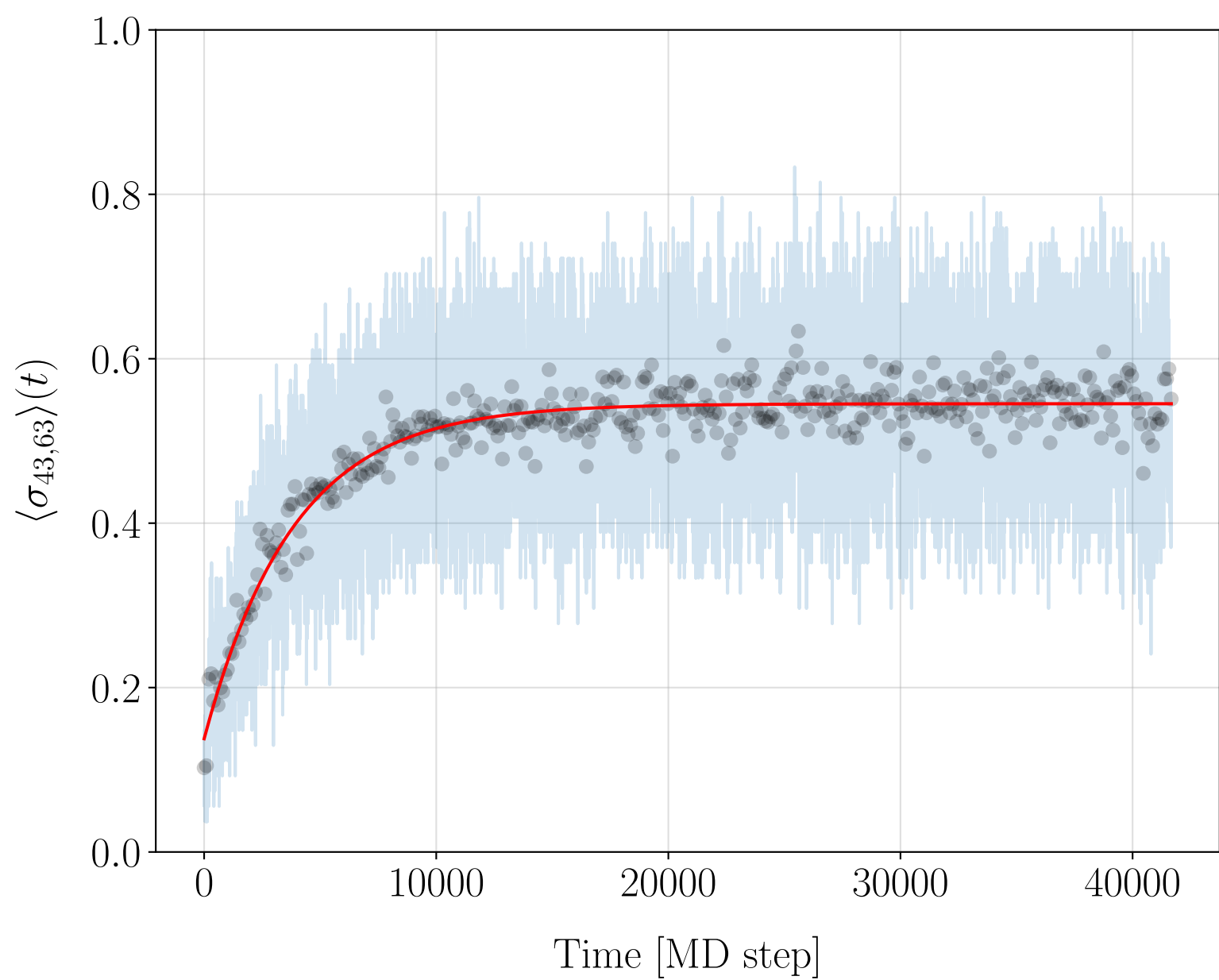

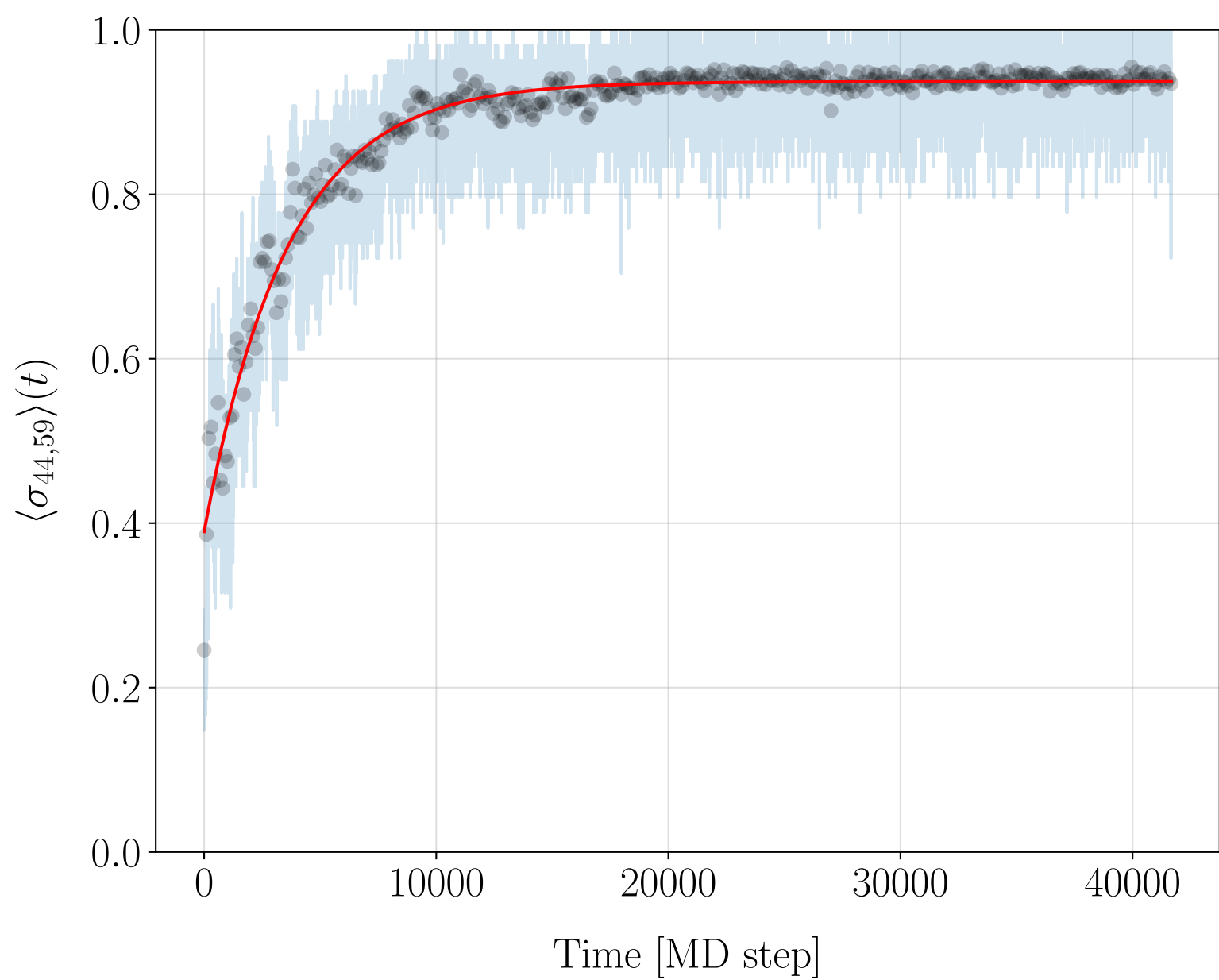

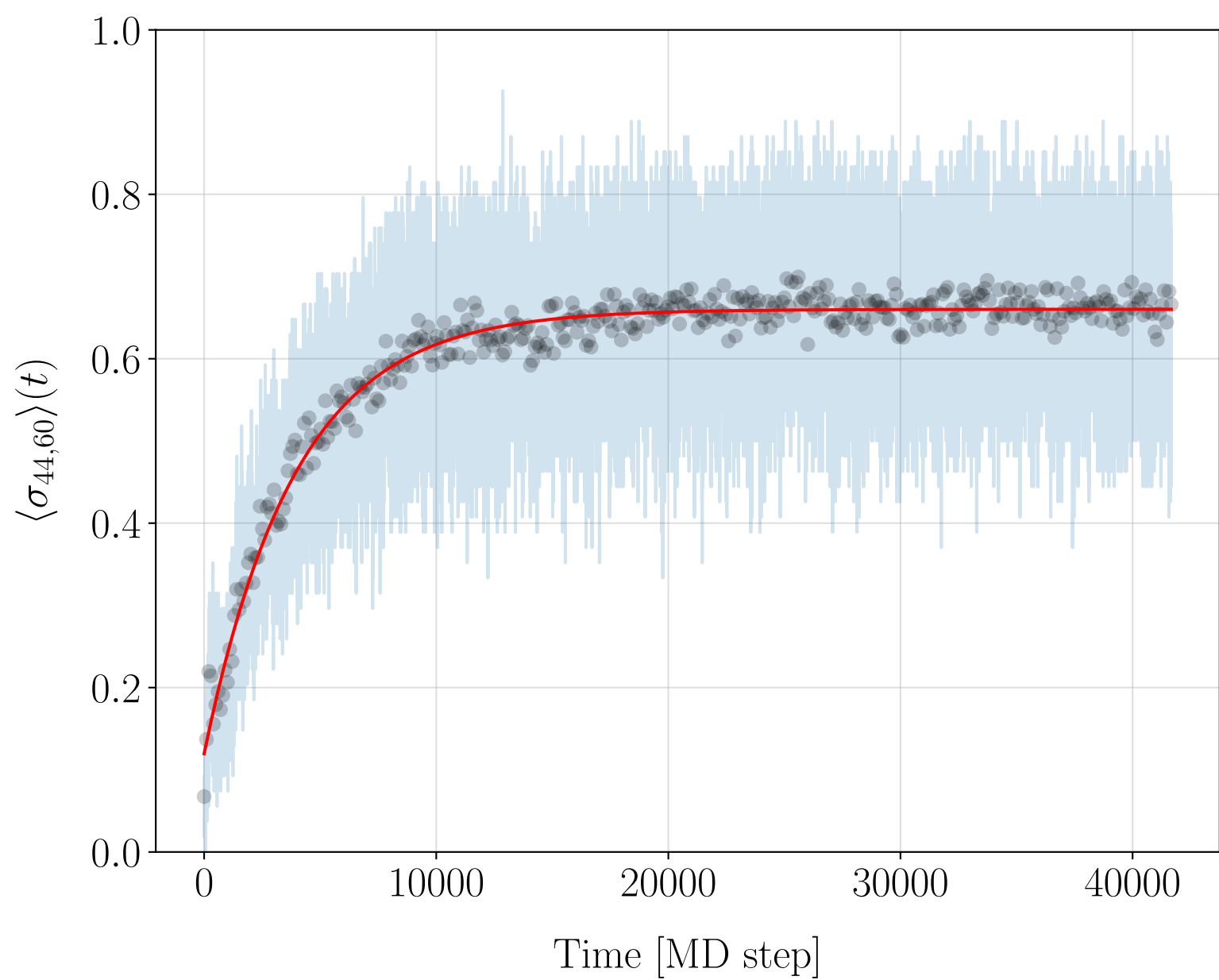

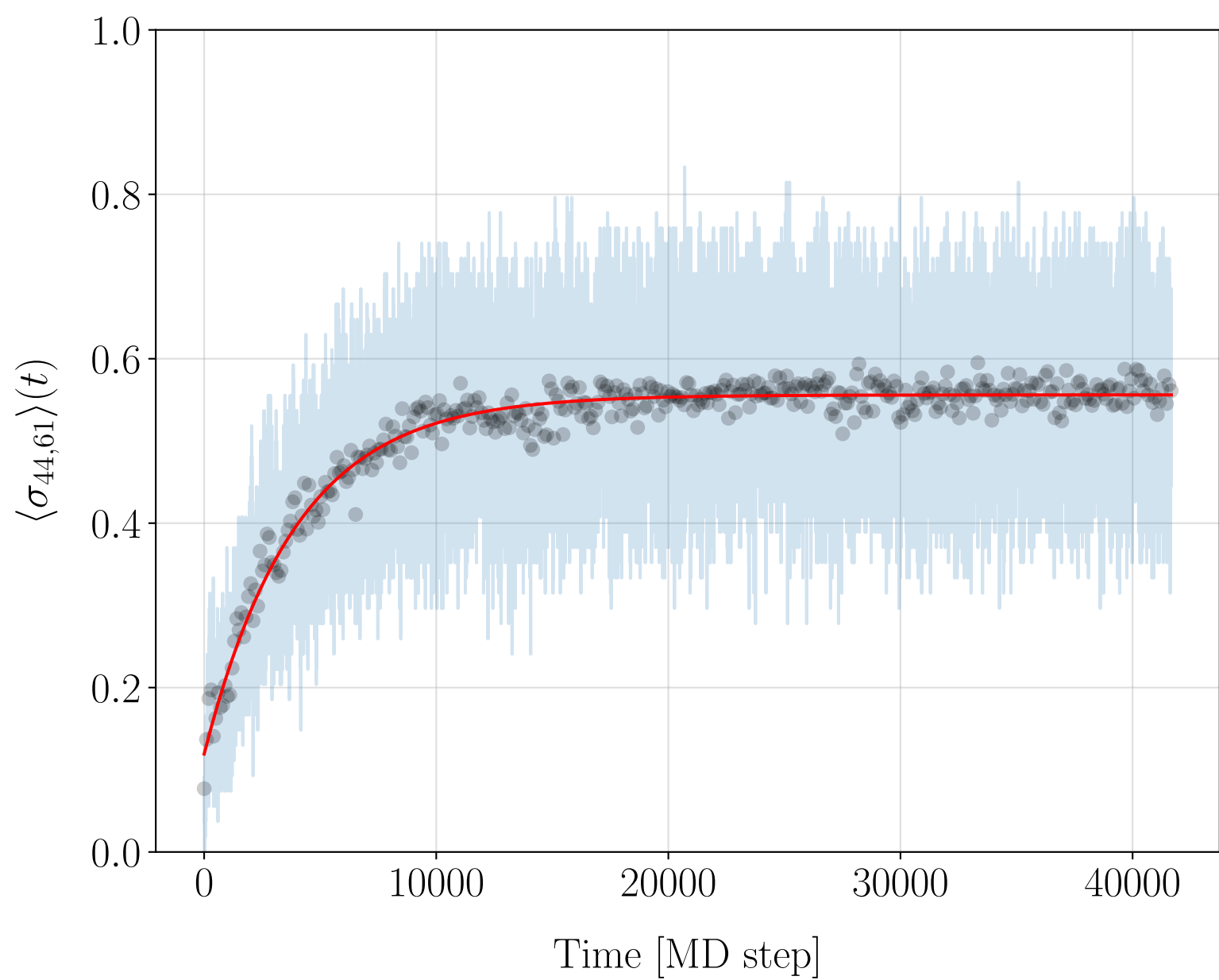

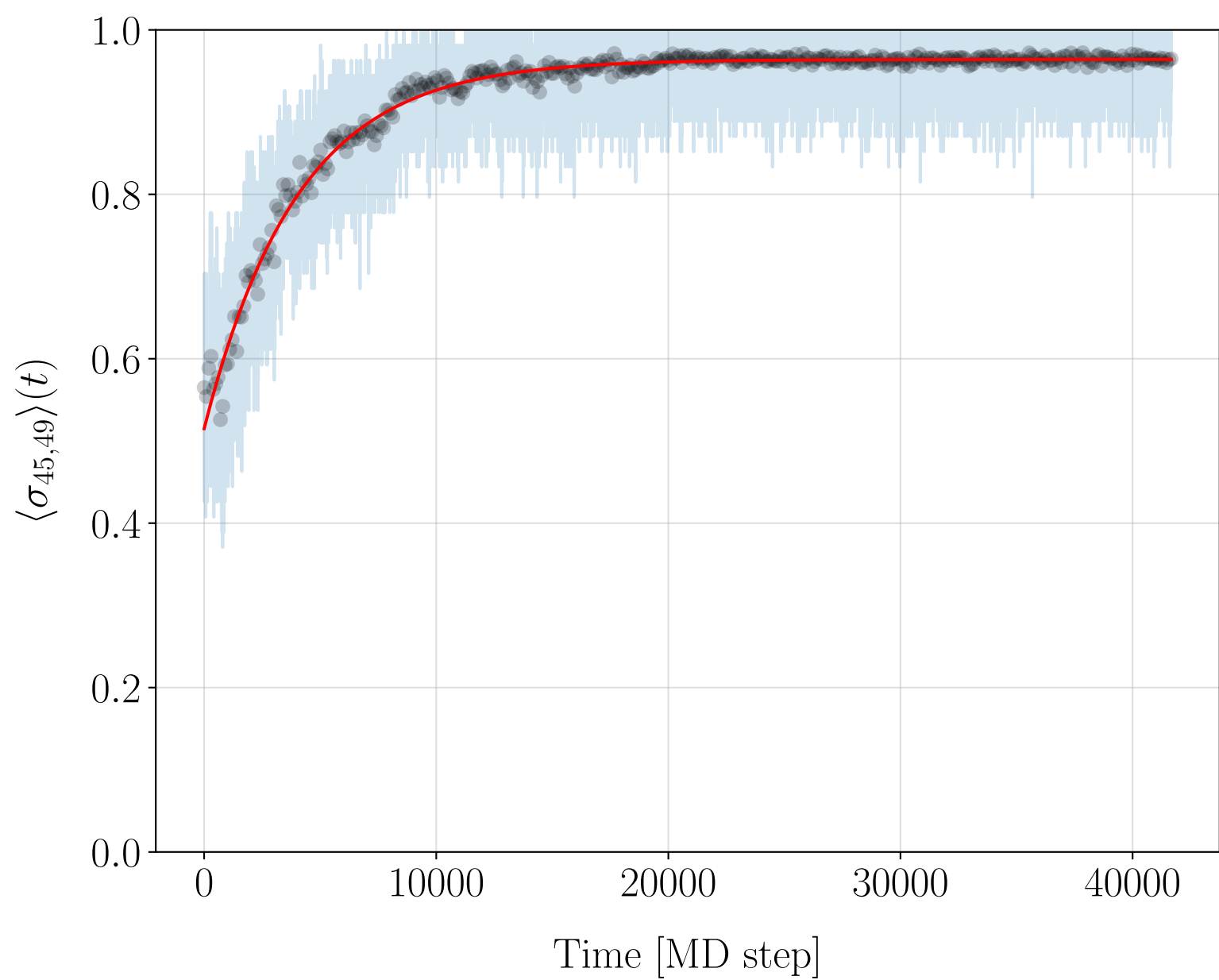

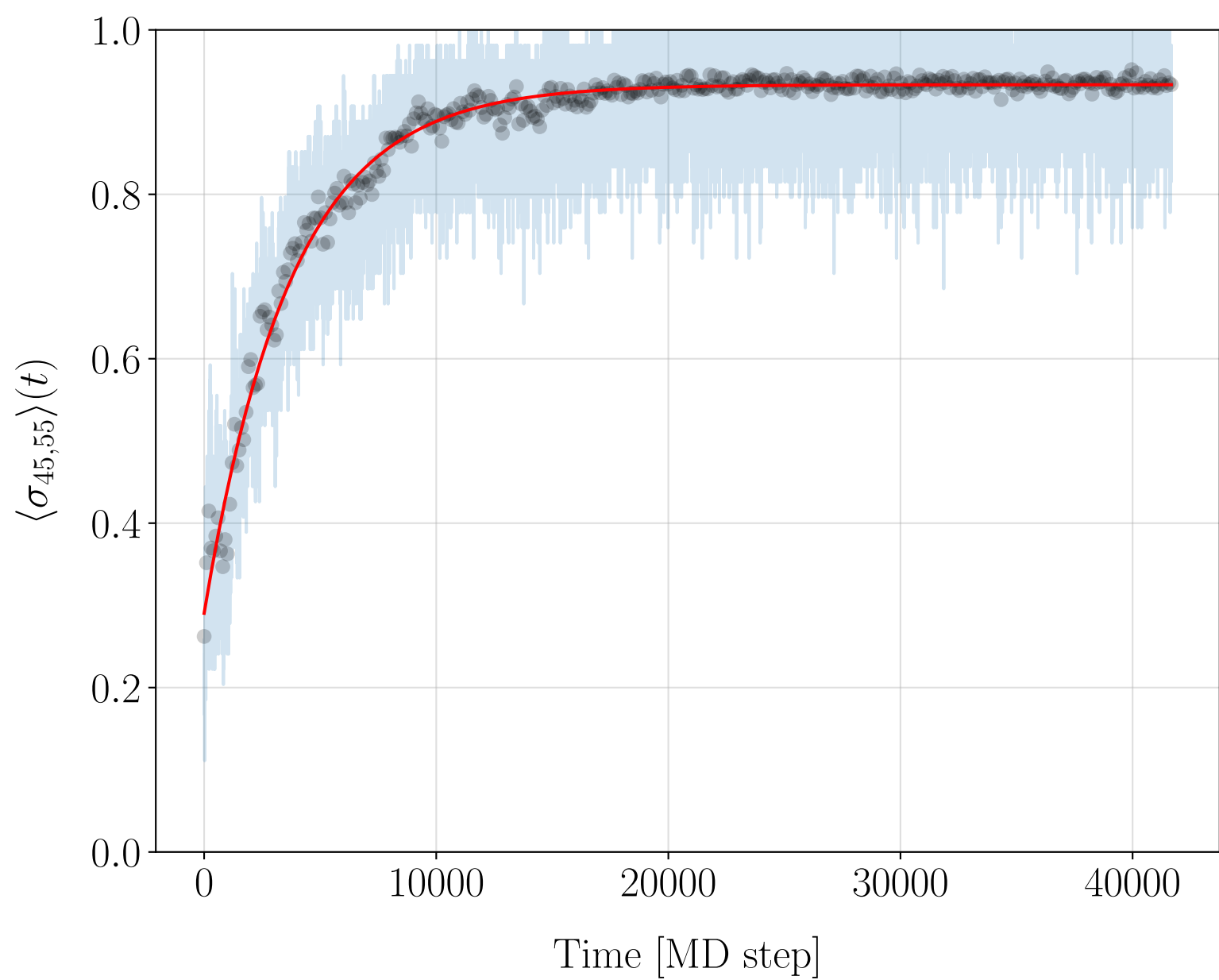

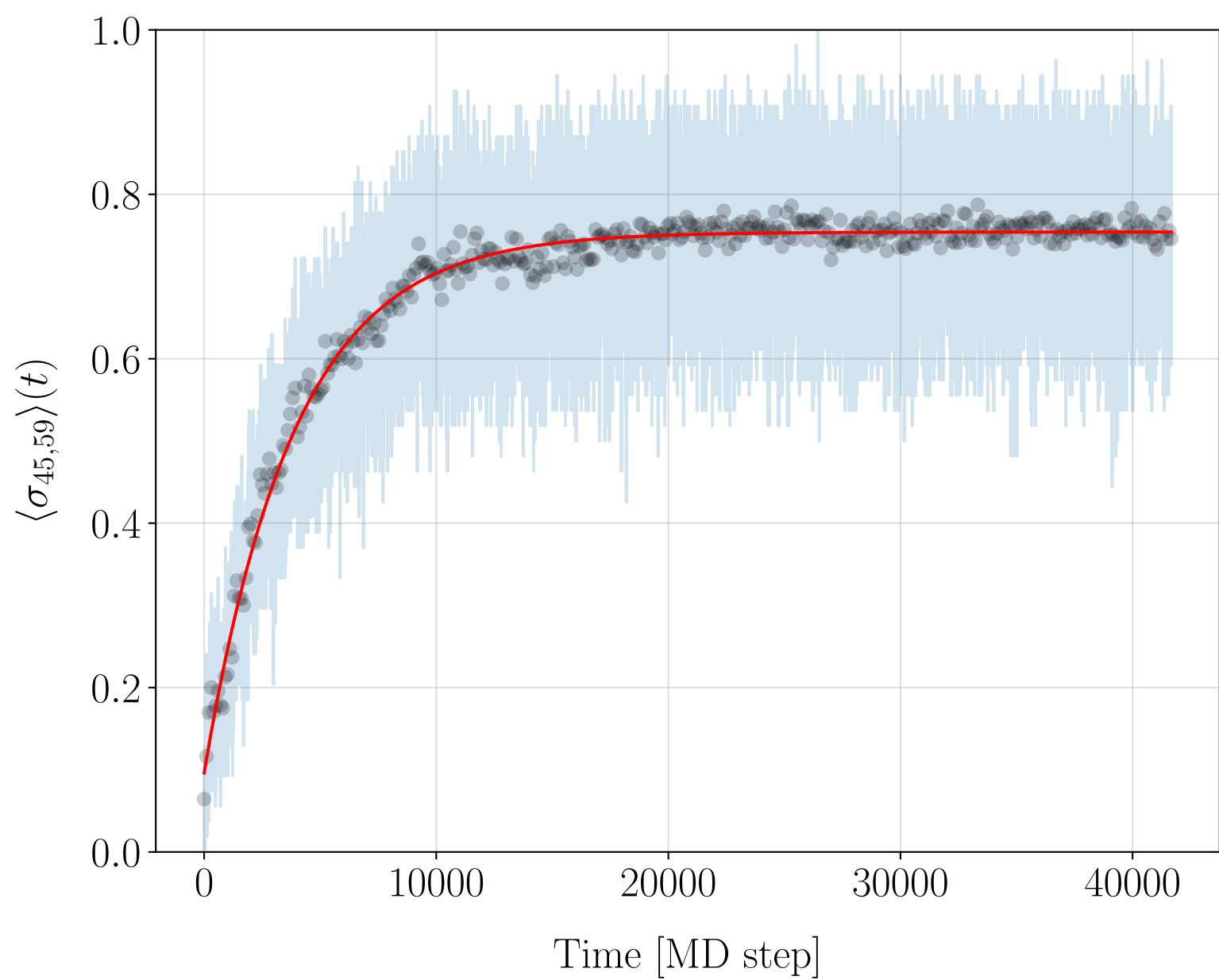

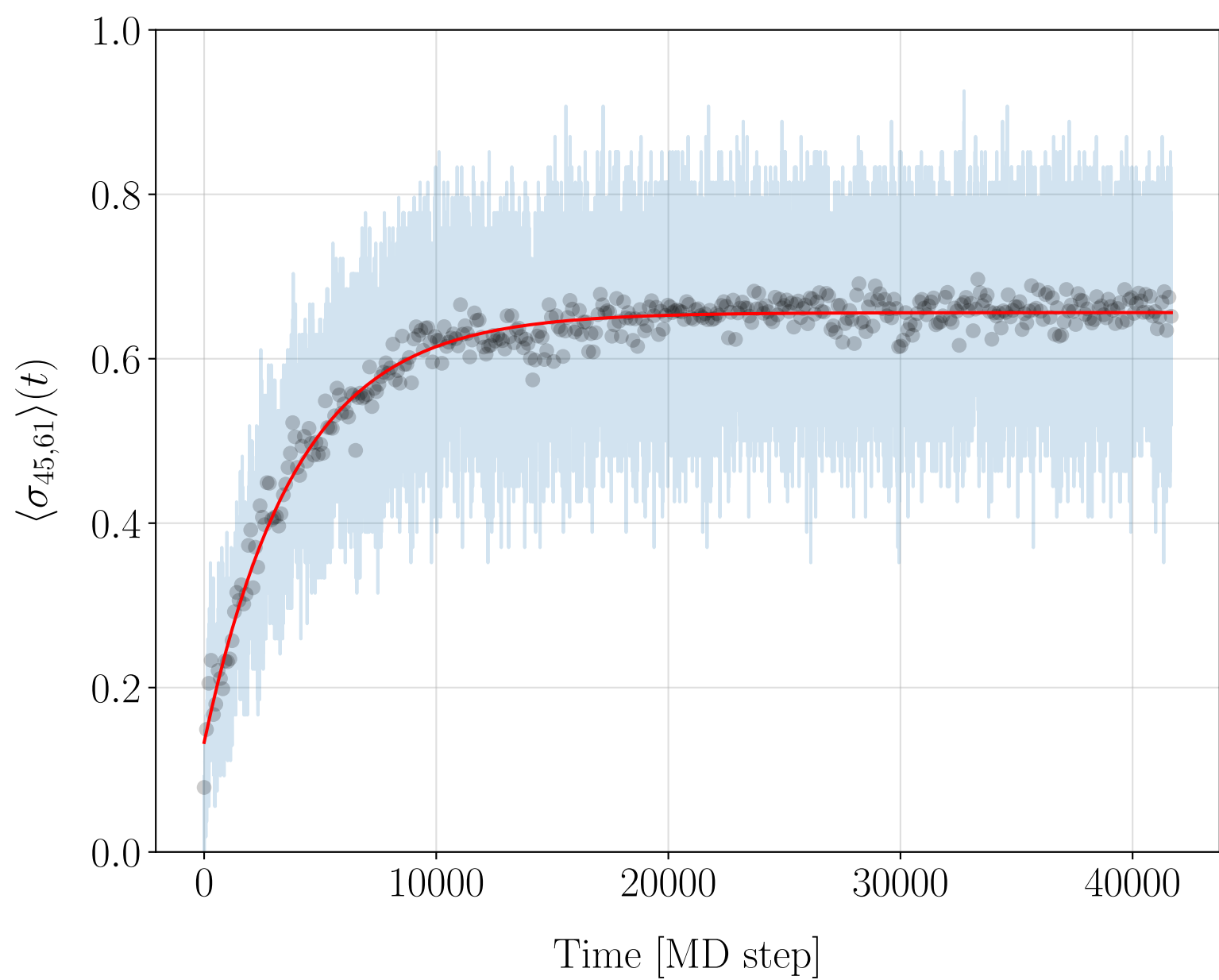

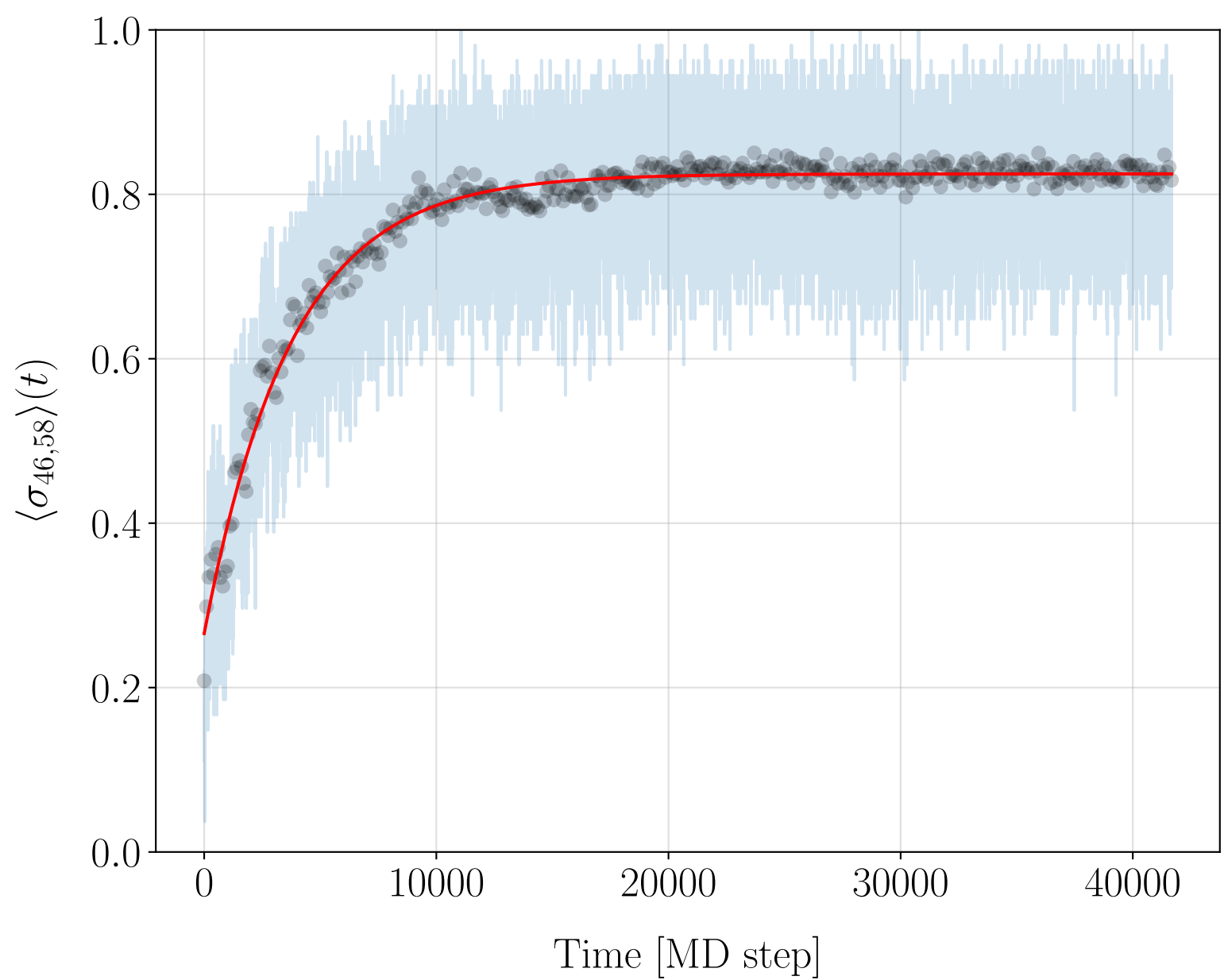

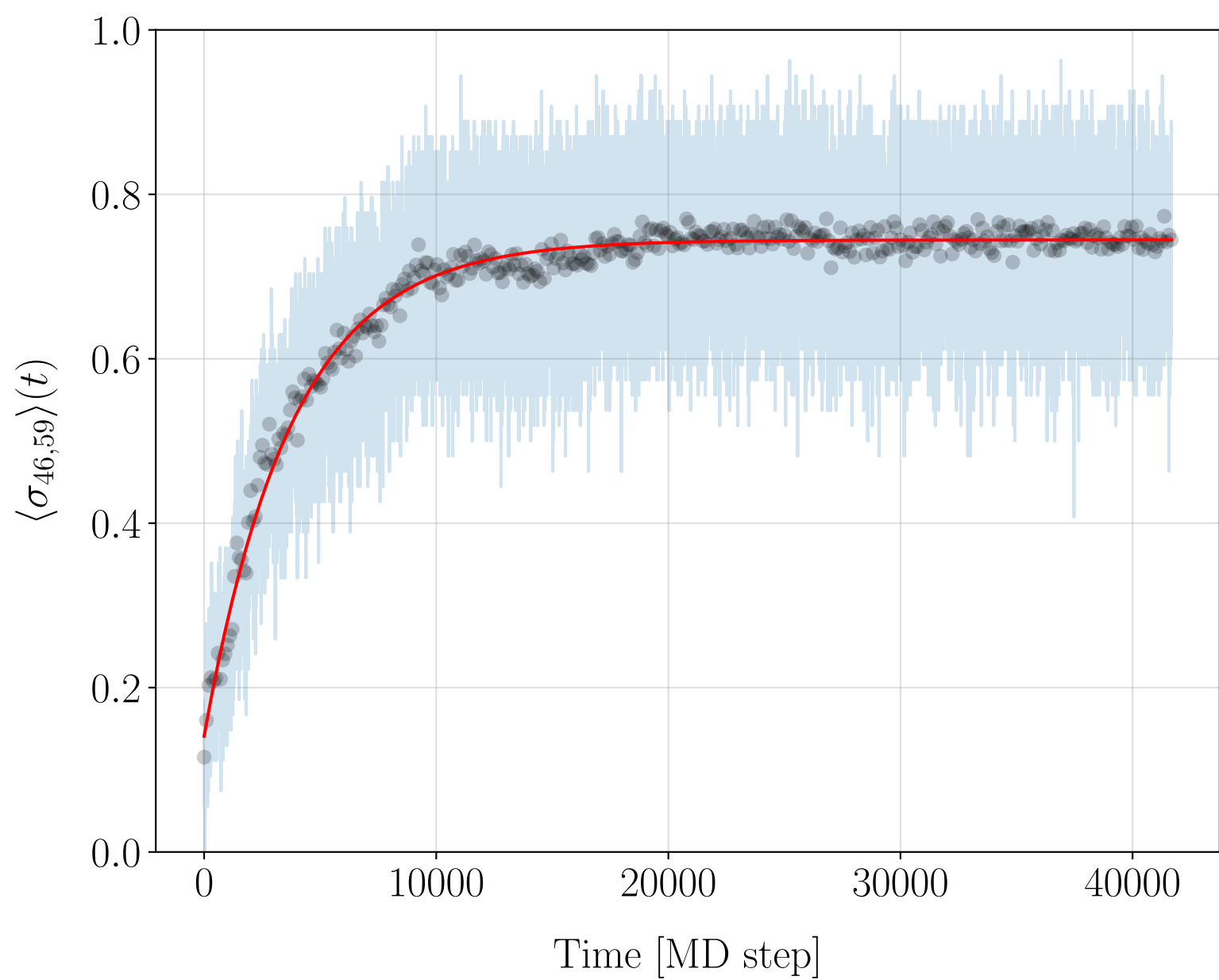

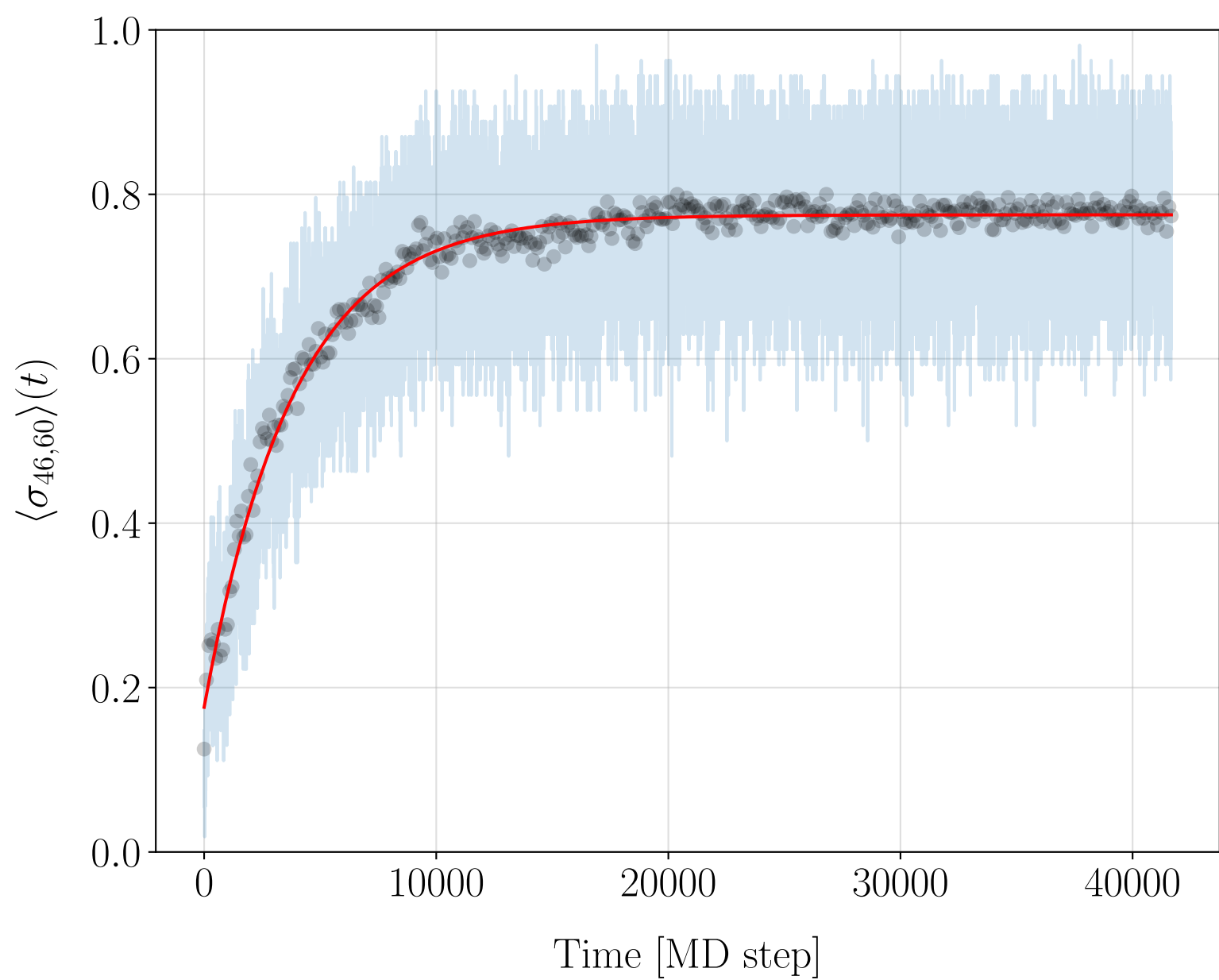

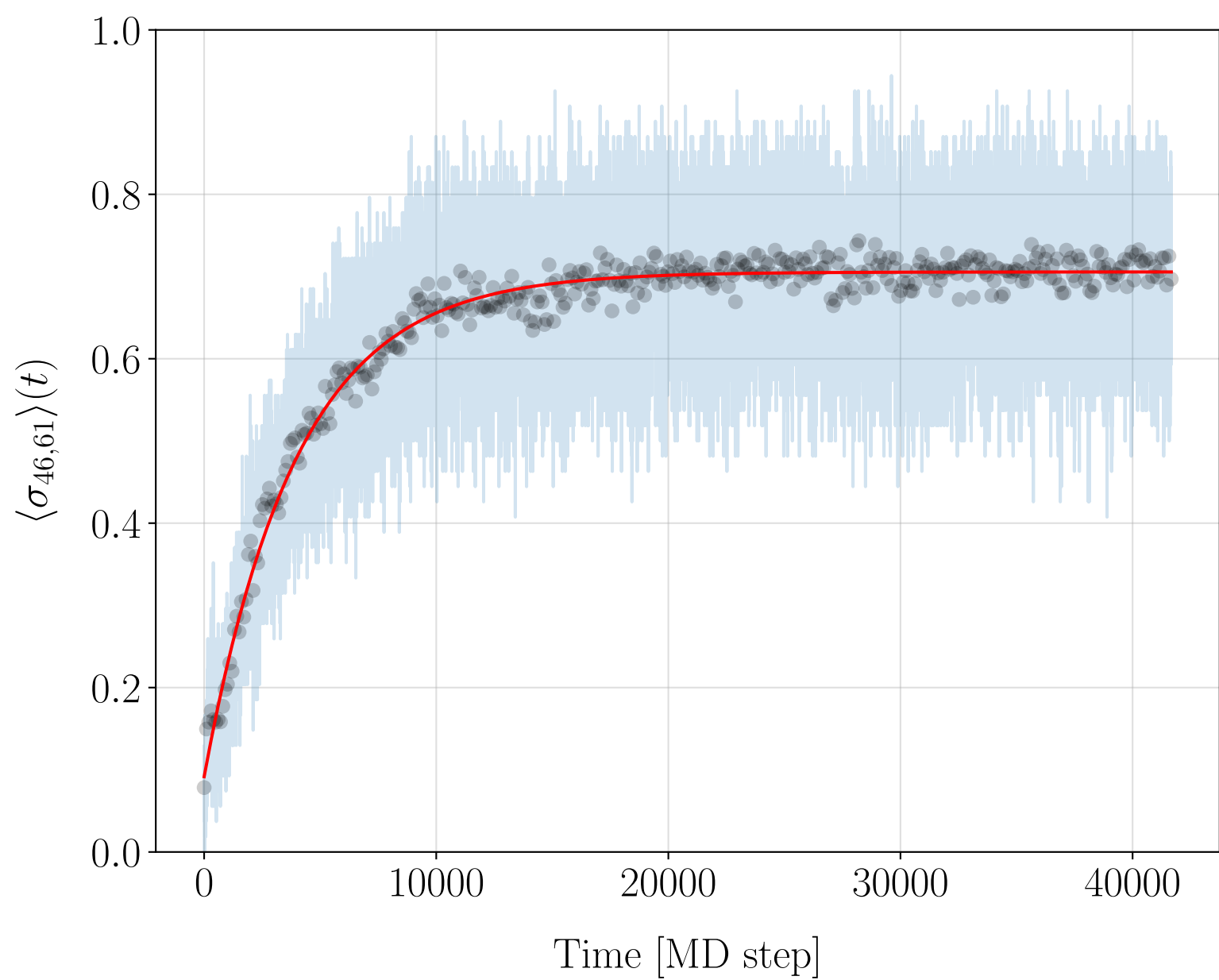

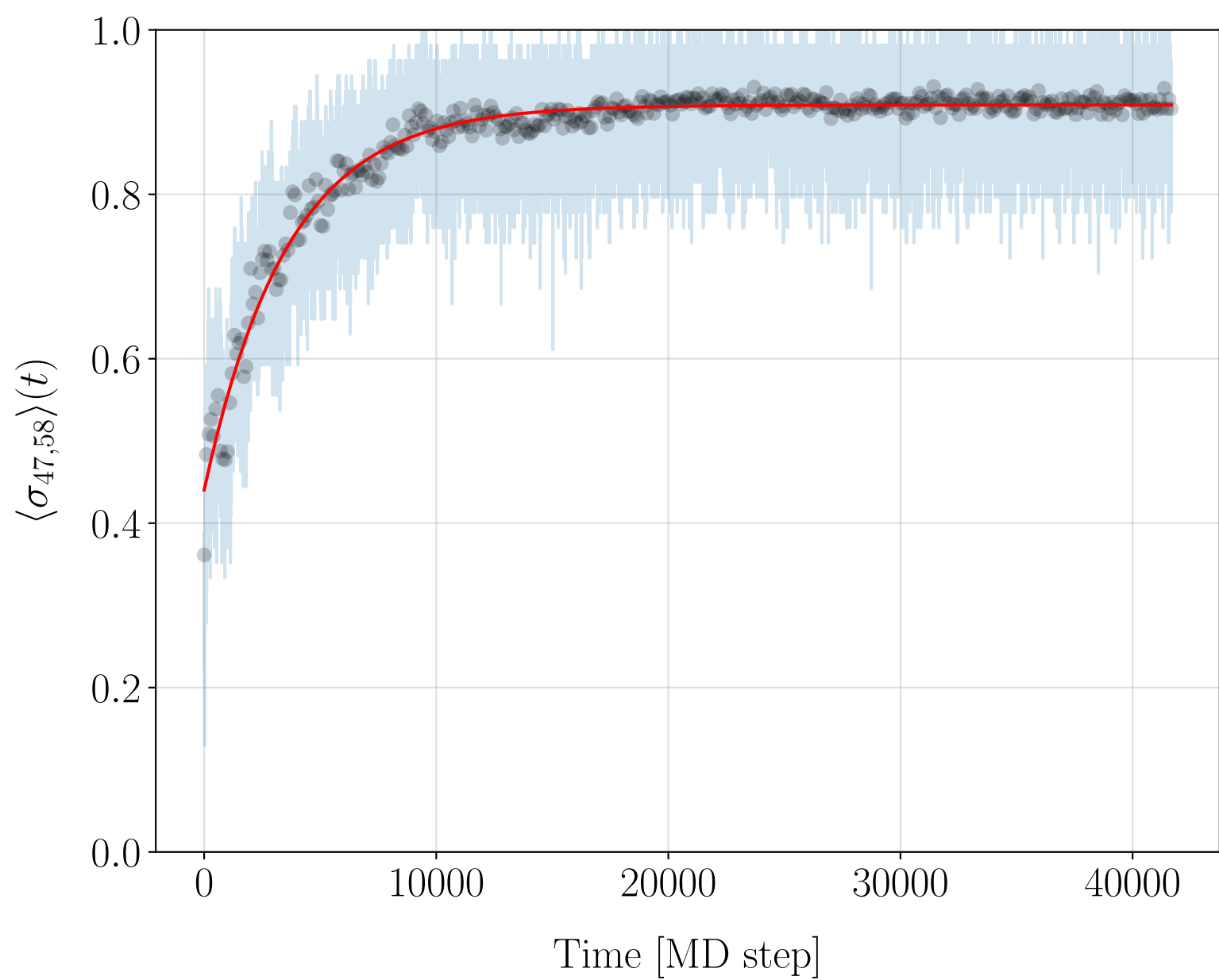

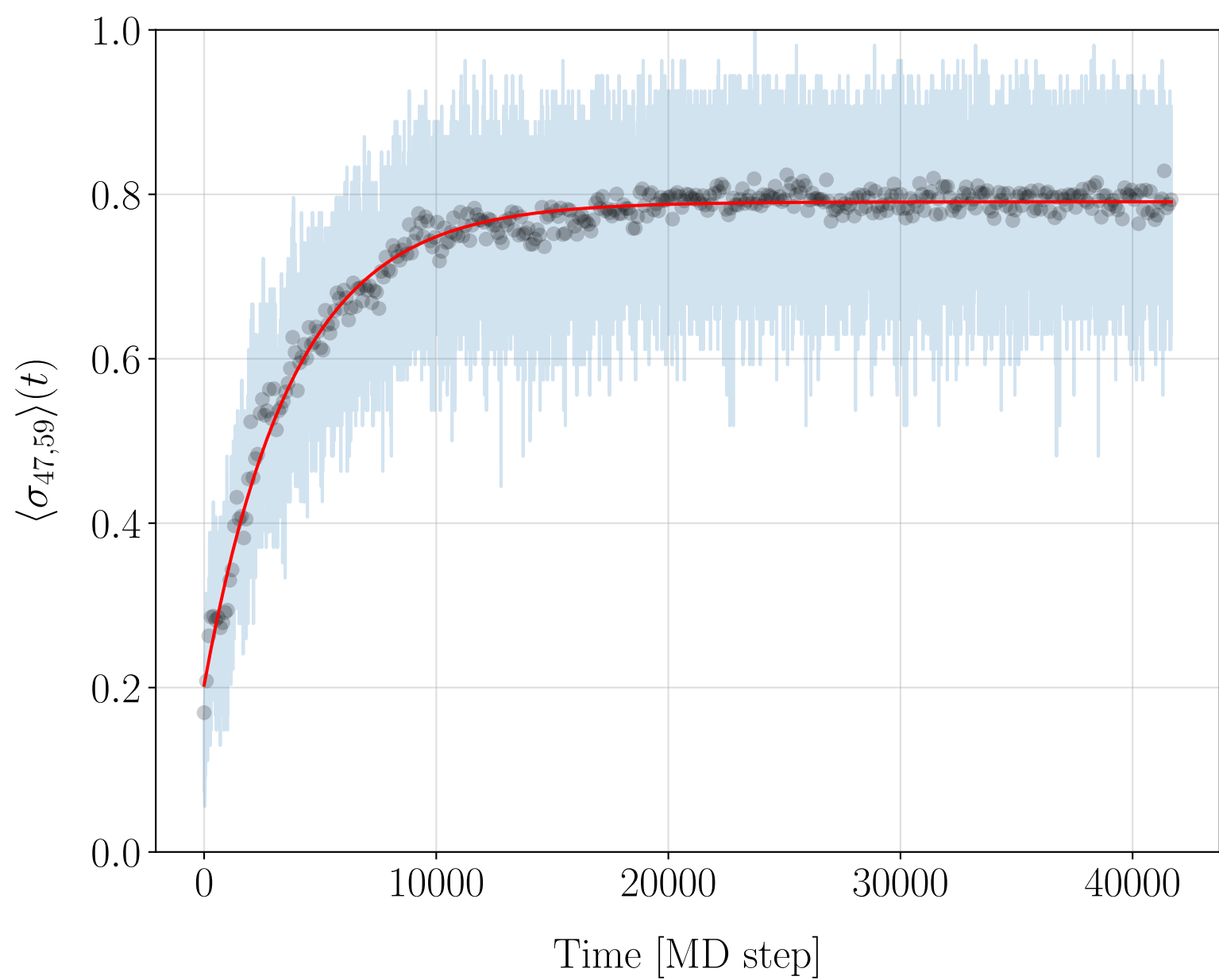

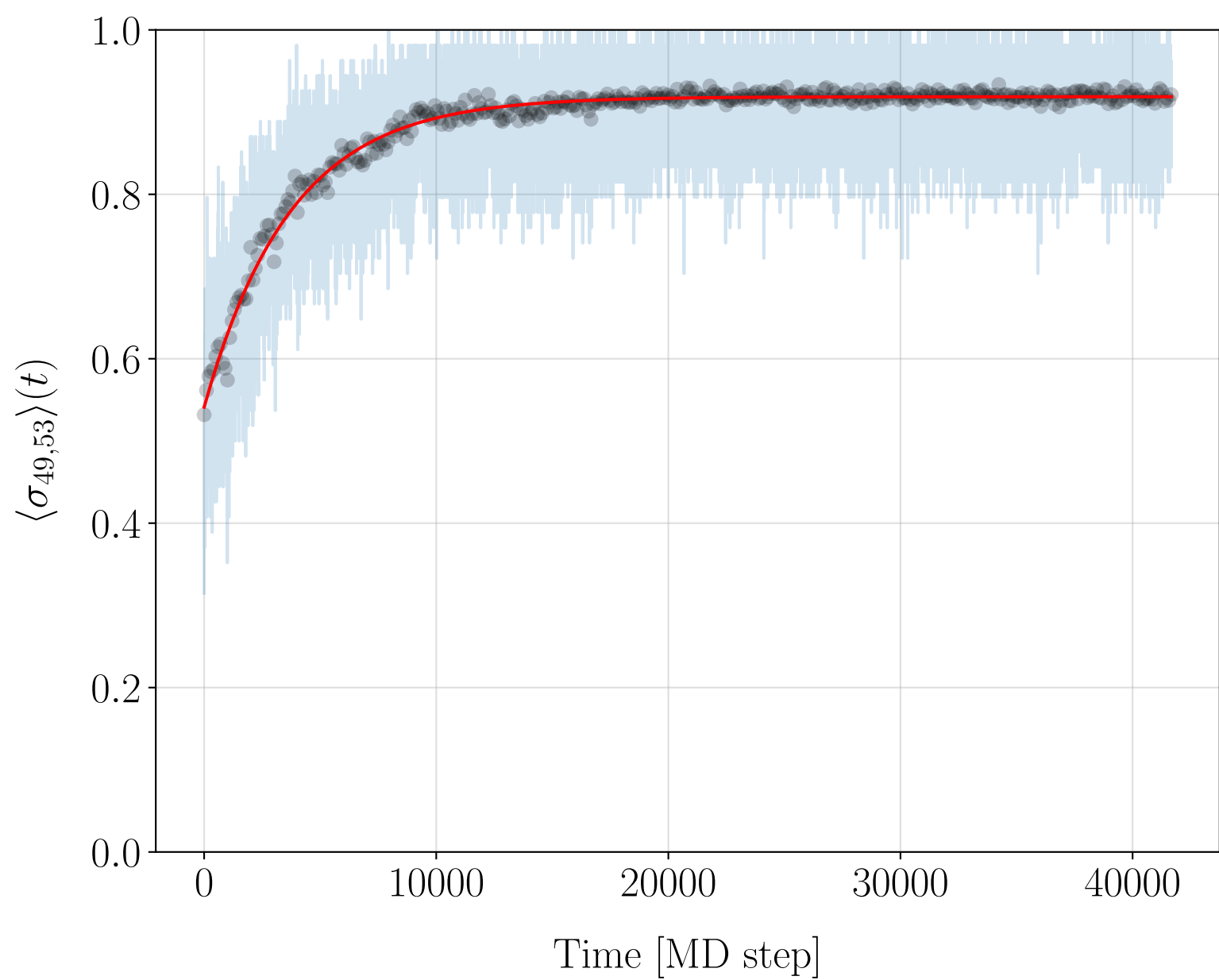

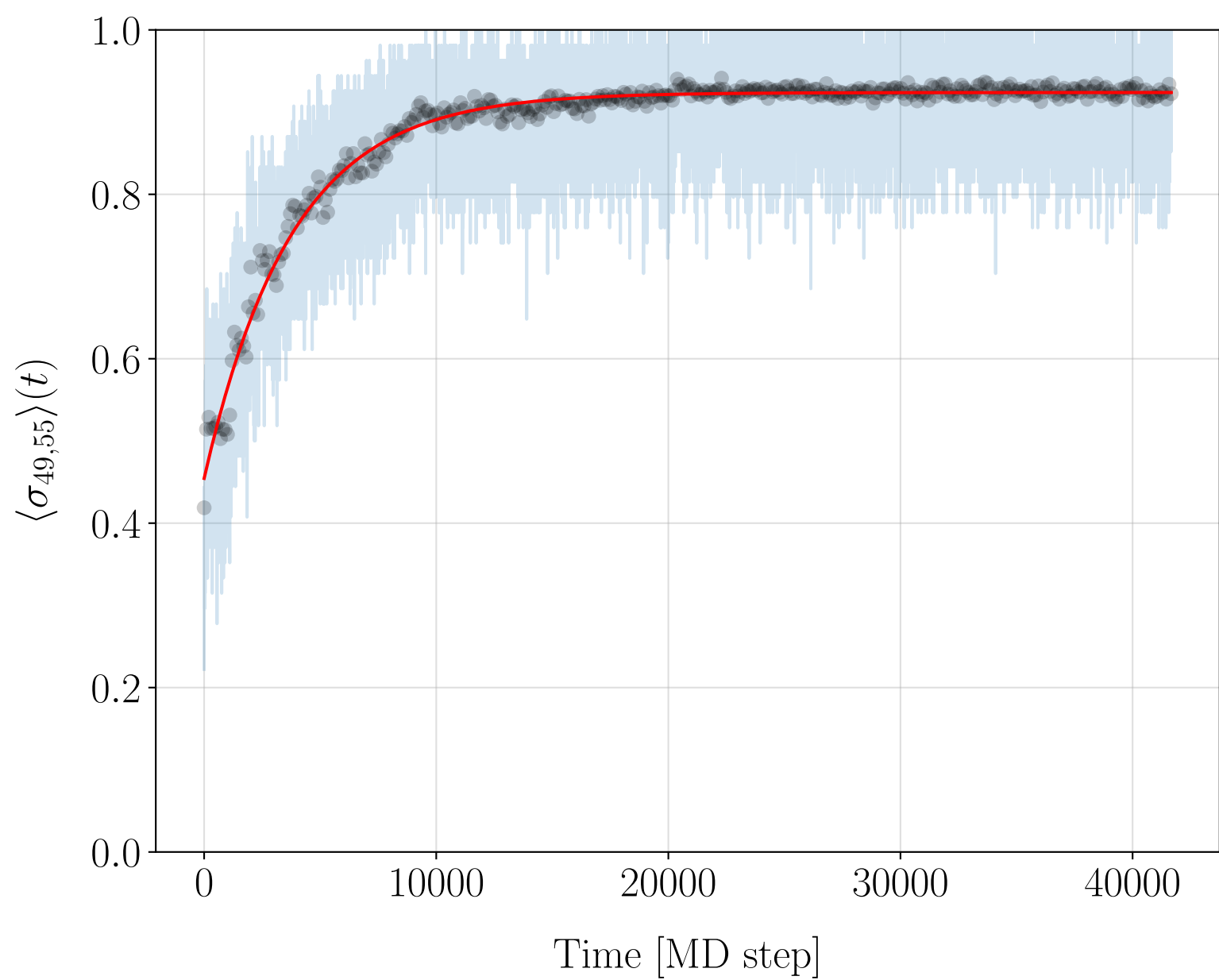

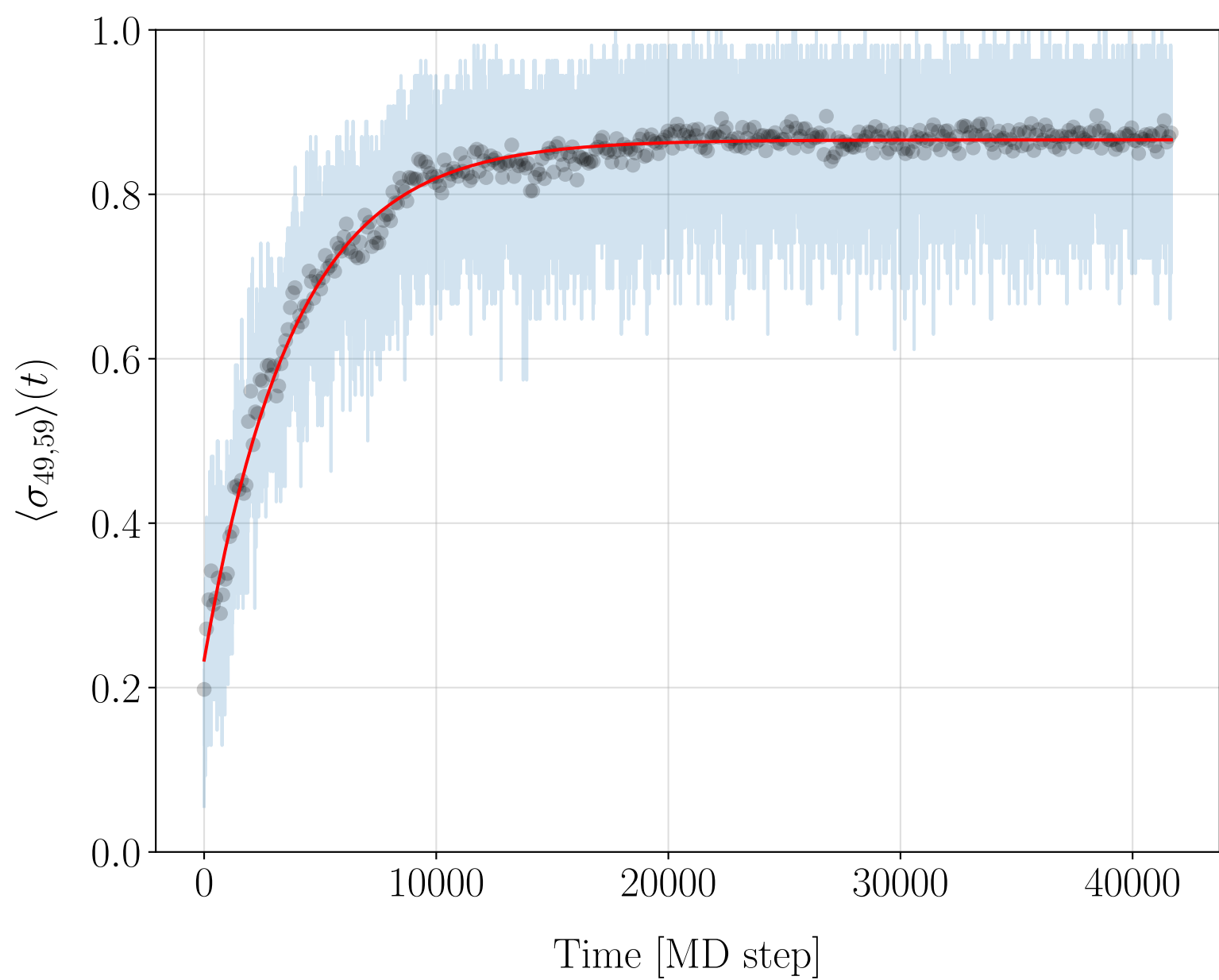

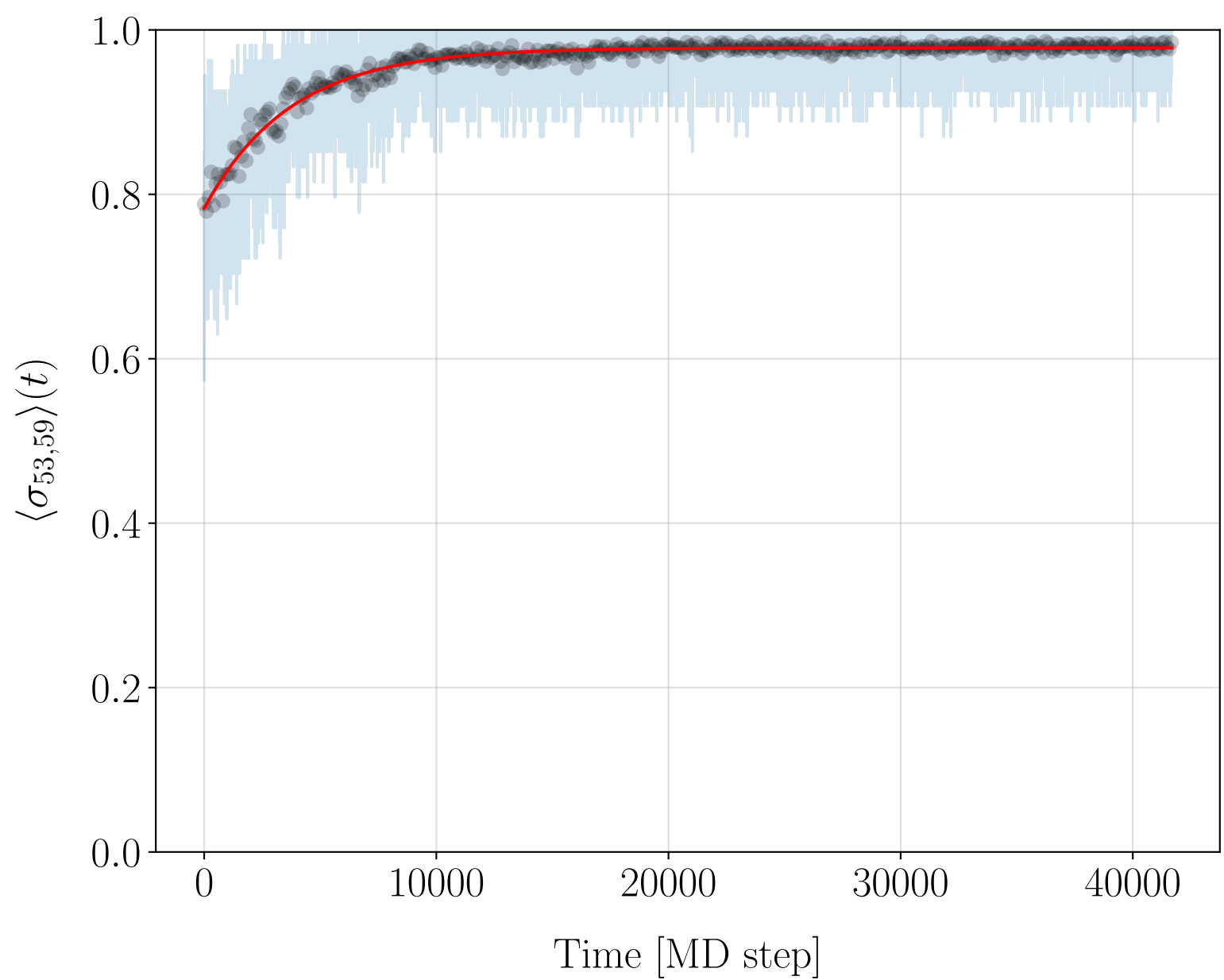

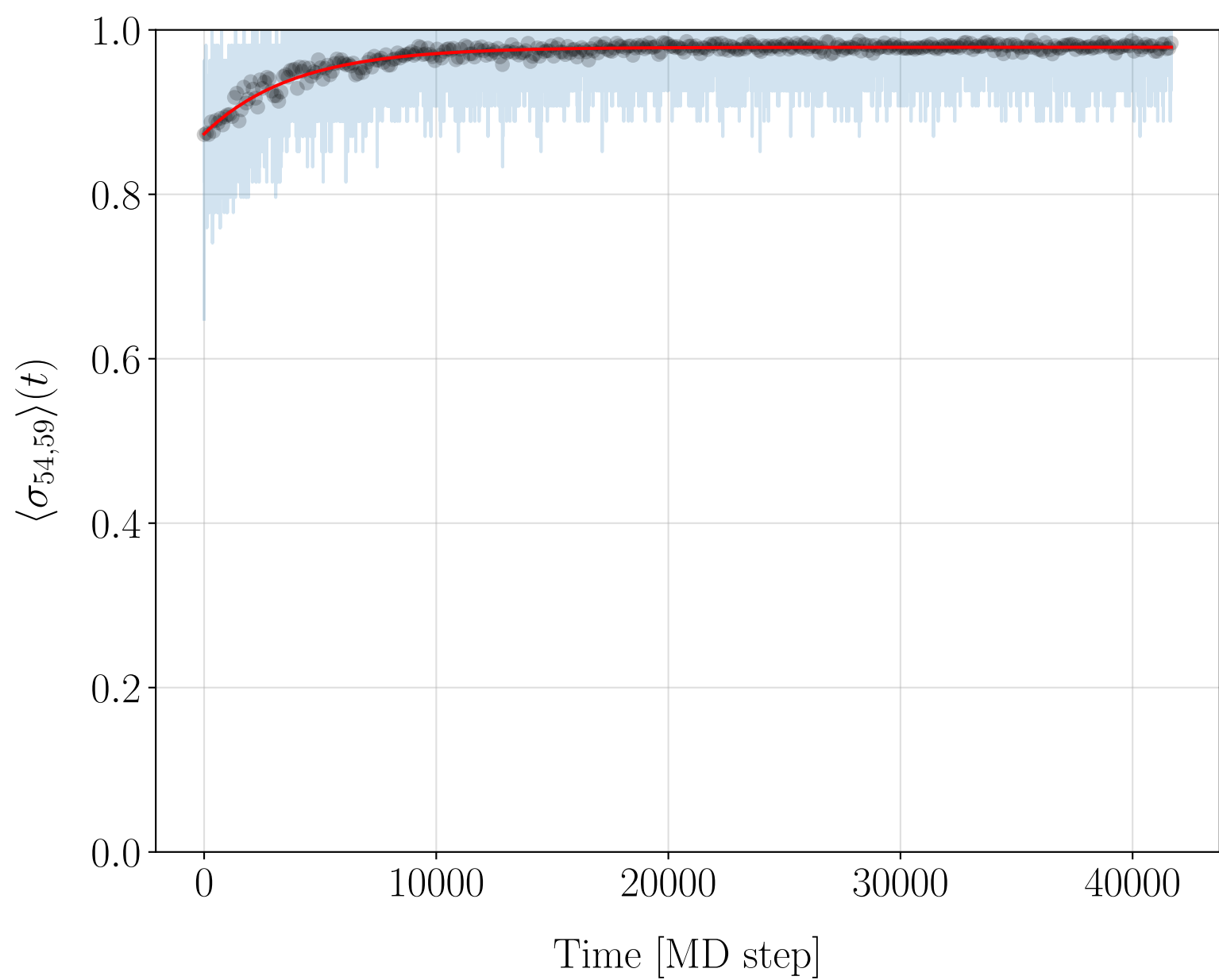

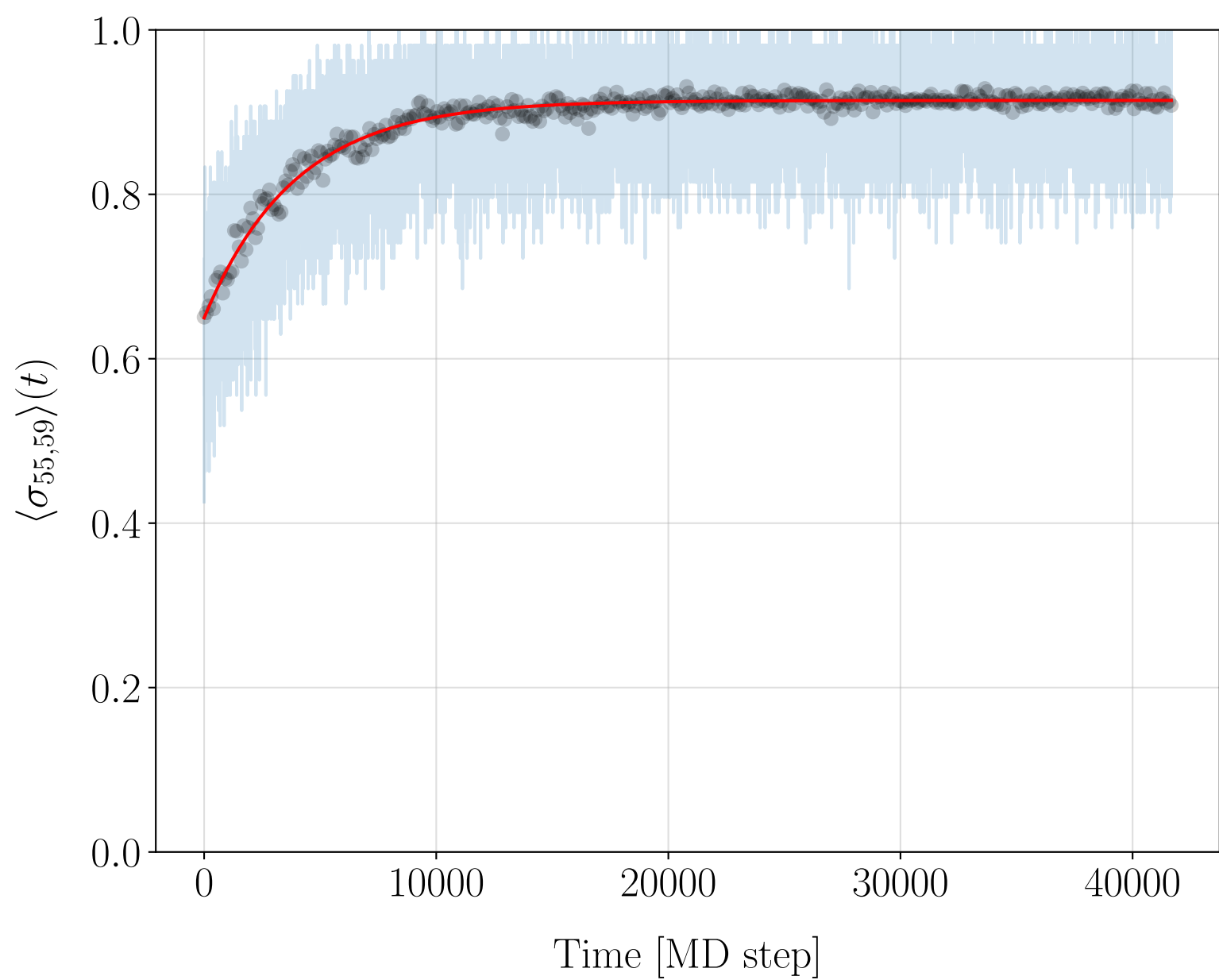

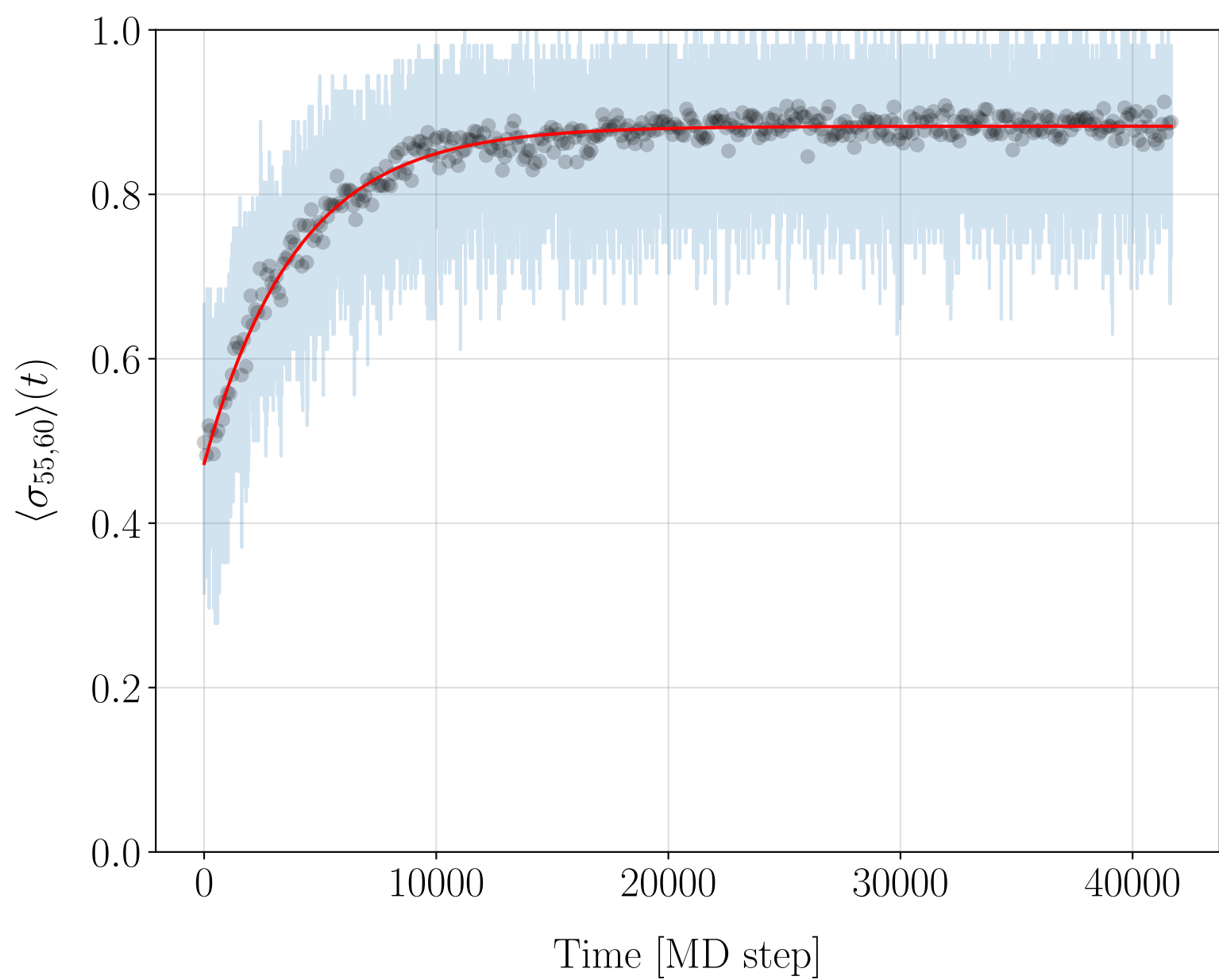

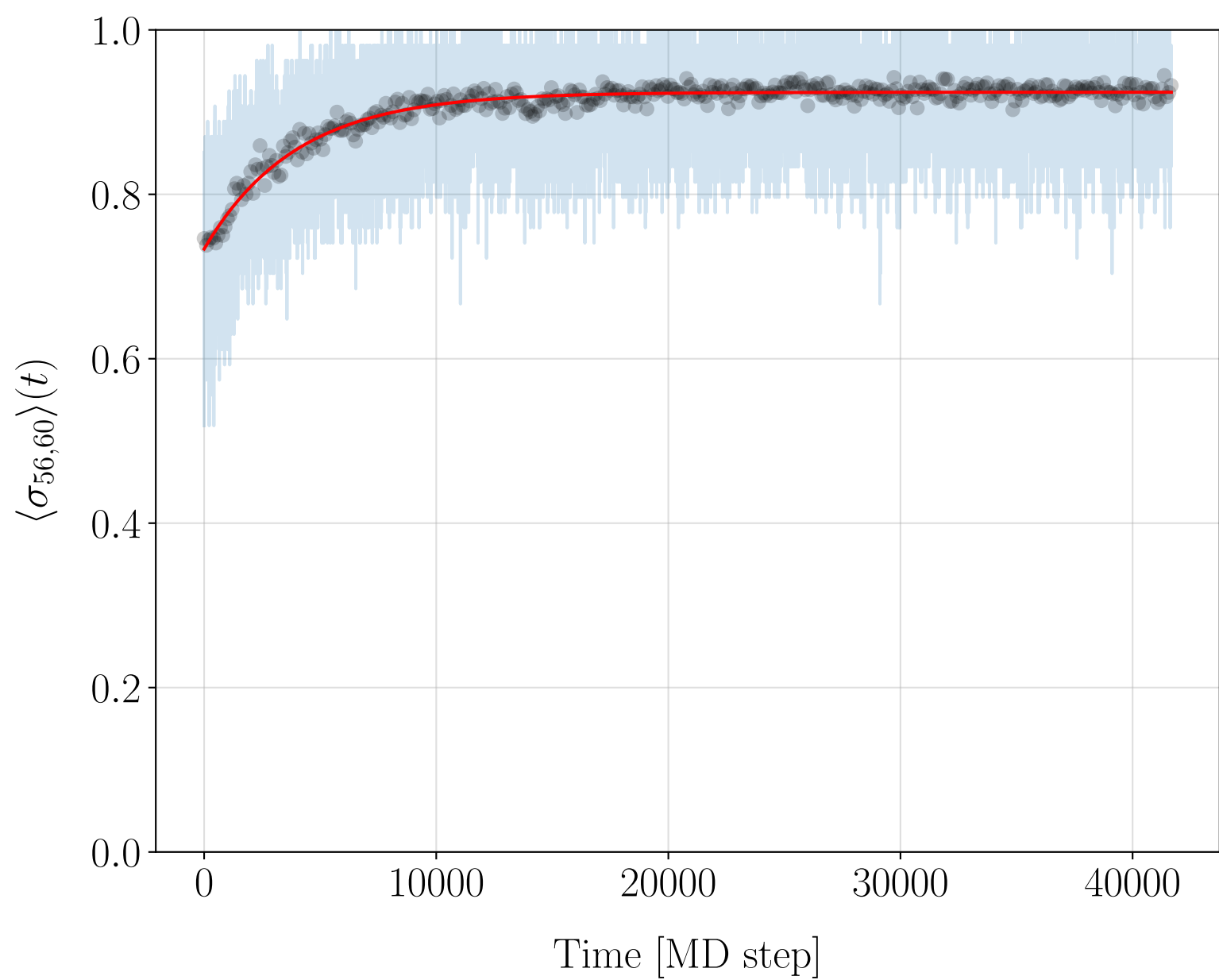

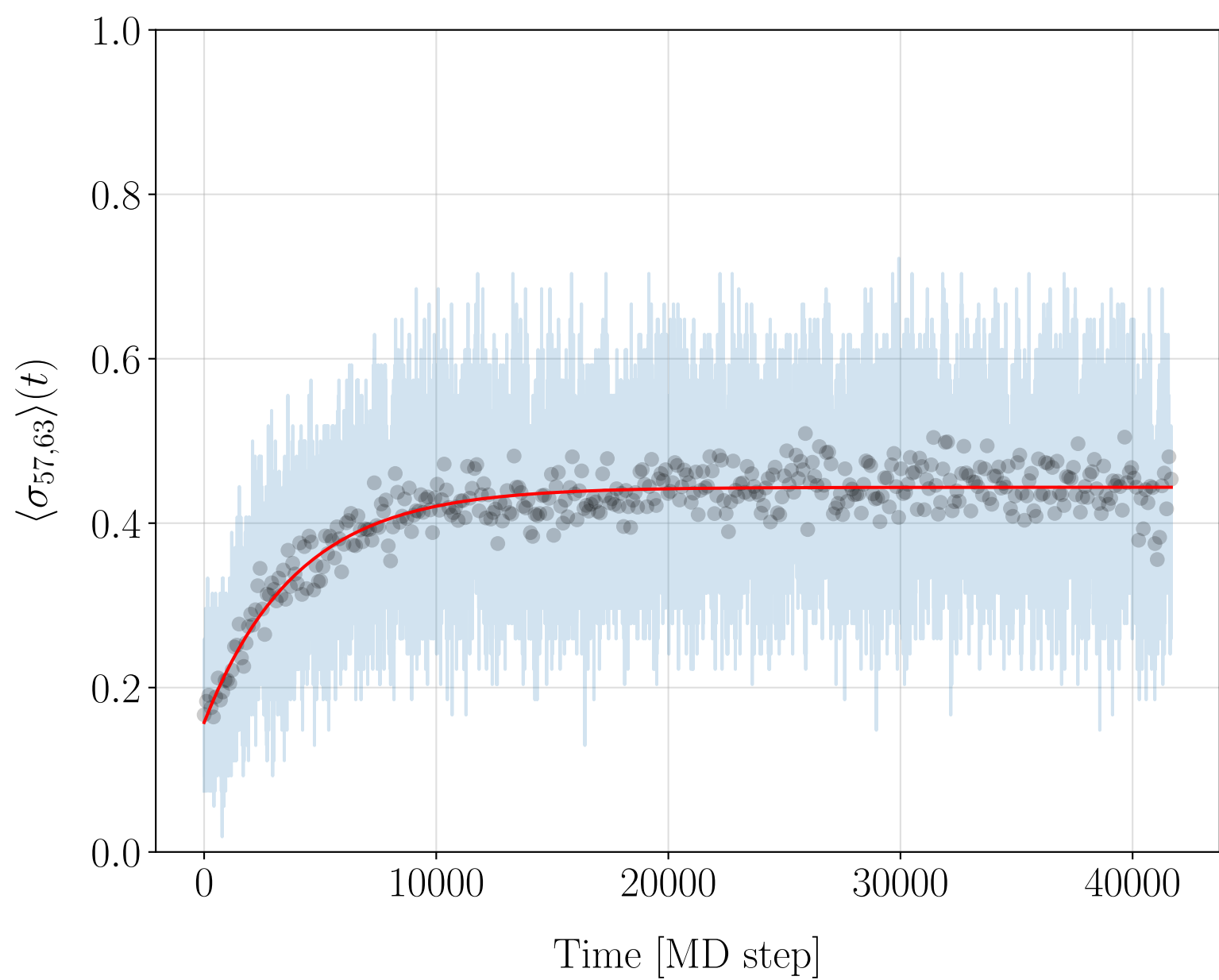

Supplement: S1 File — Only the 52 trajectories refolding at t = 0.9Tf through the fast channel are used to compute contact formation probabilities. All plots in this file are drawn as explained in the caption of Fig 7. (PDF) [file pcbi.1011107.s002.pdf]

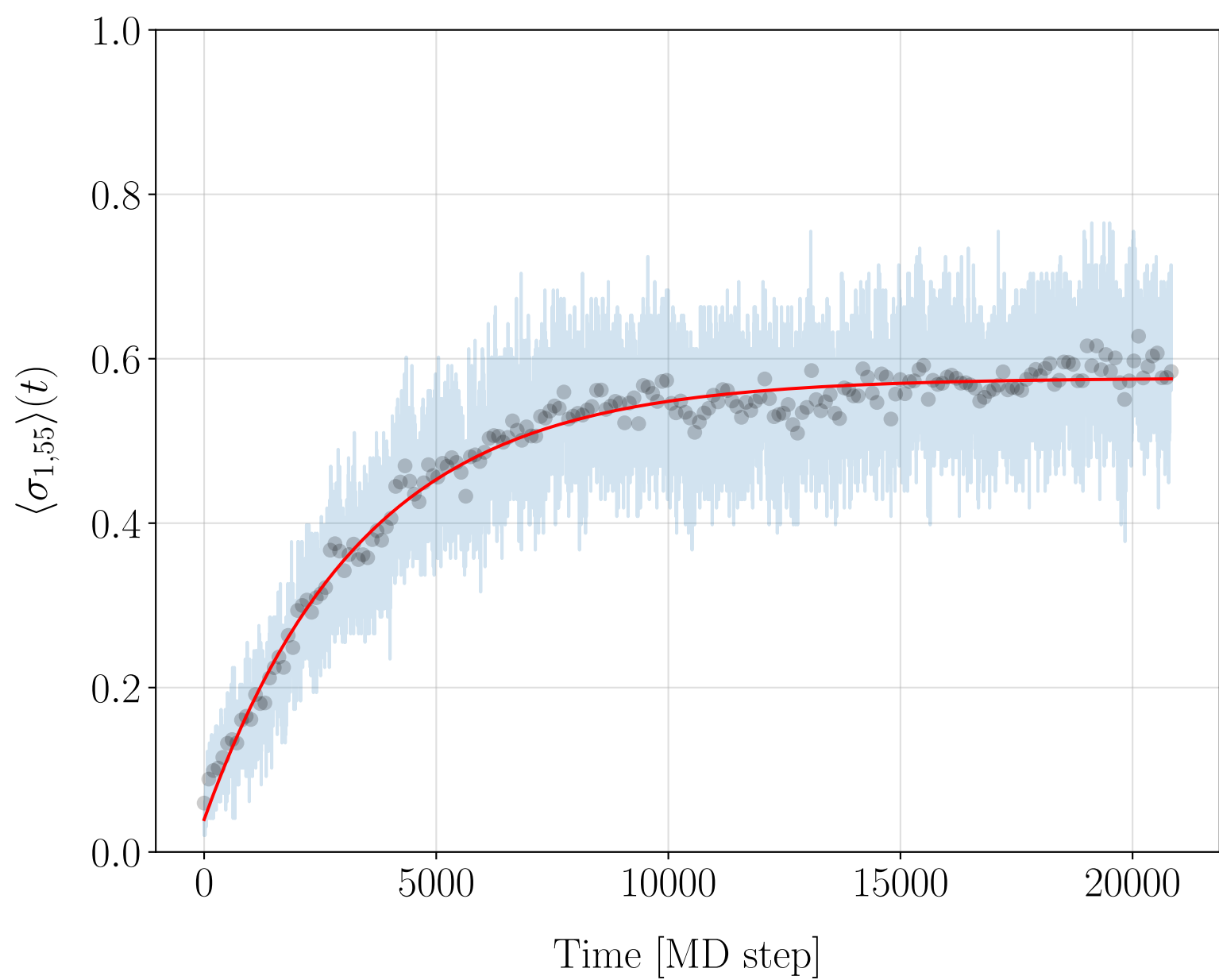

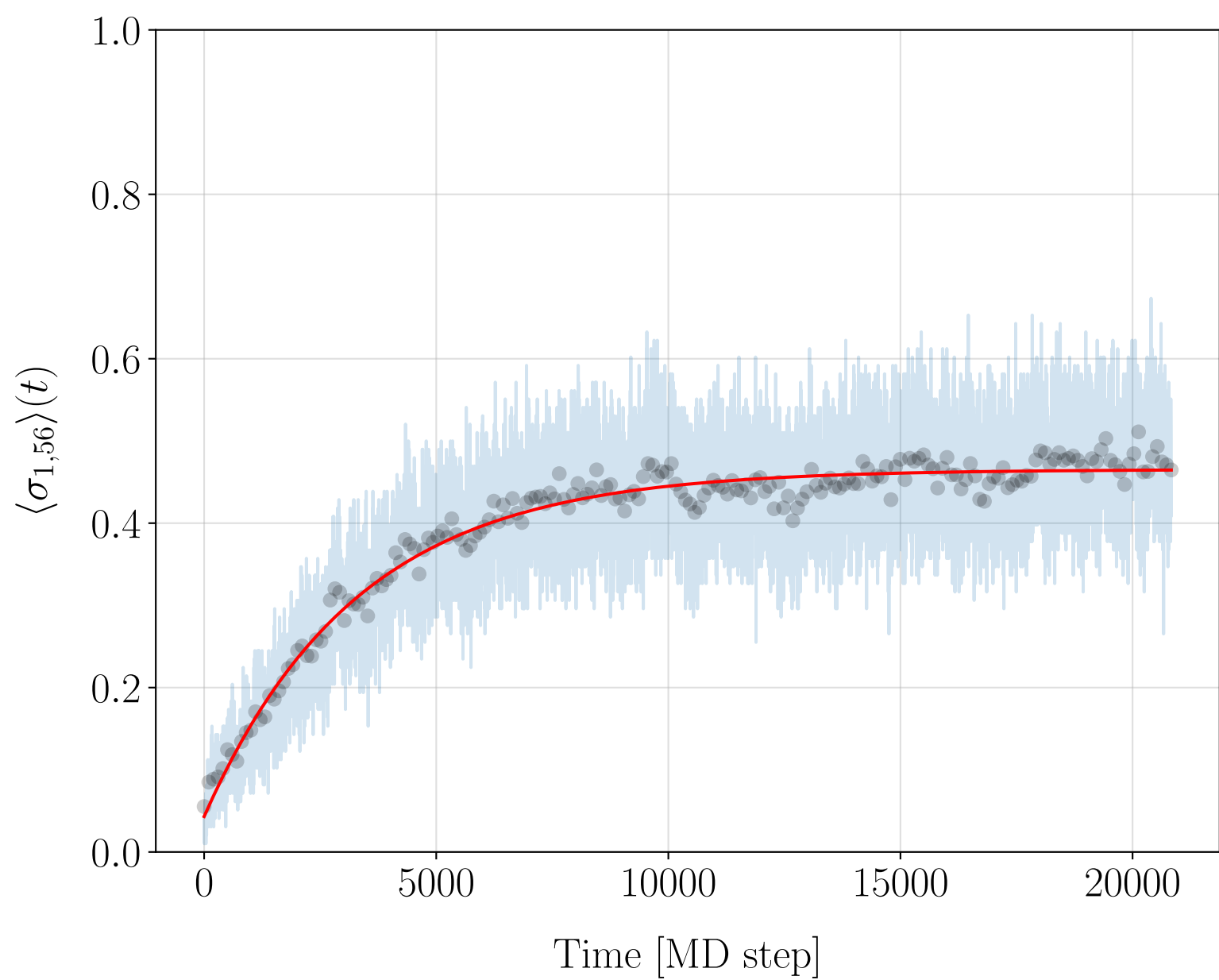

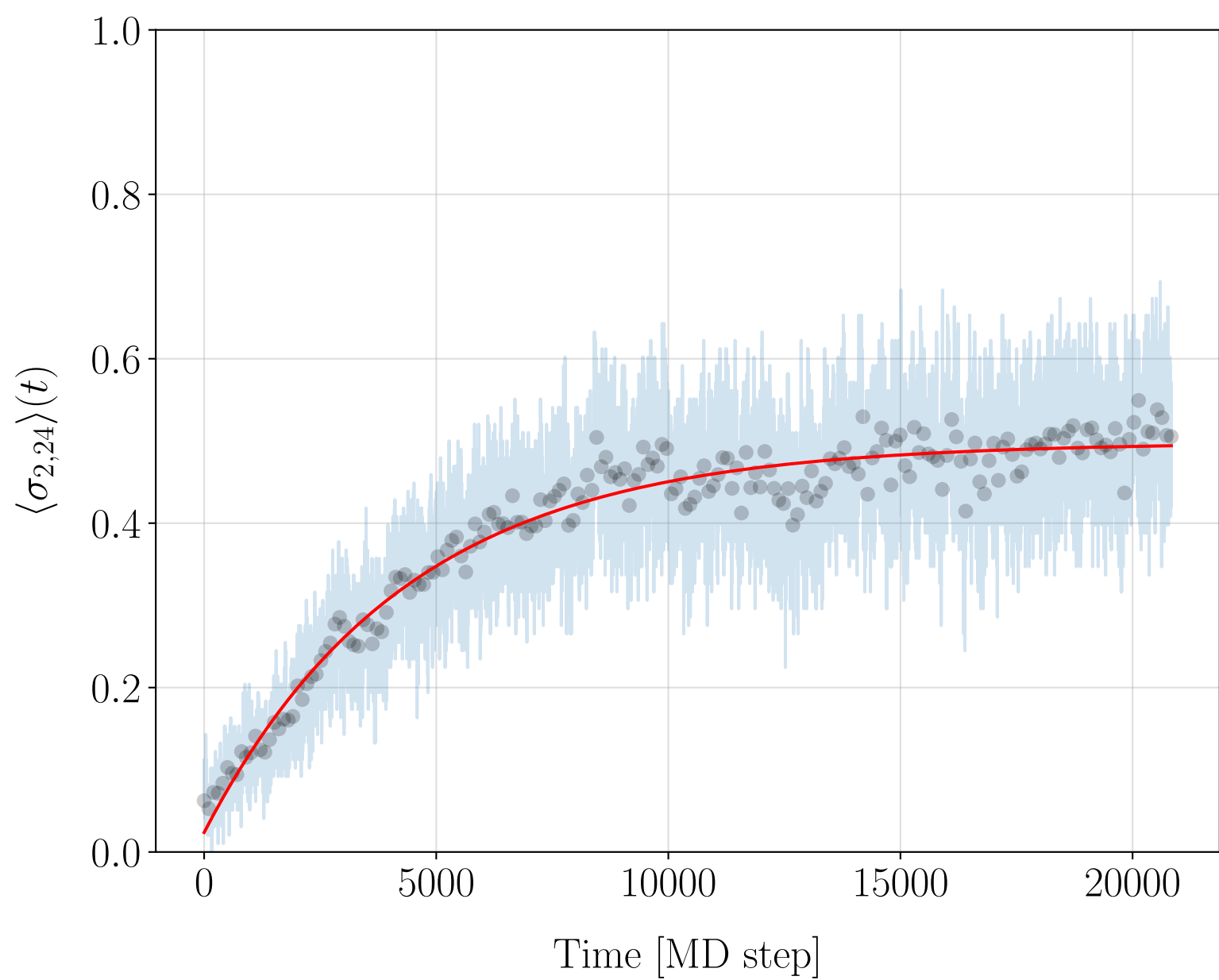

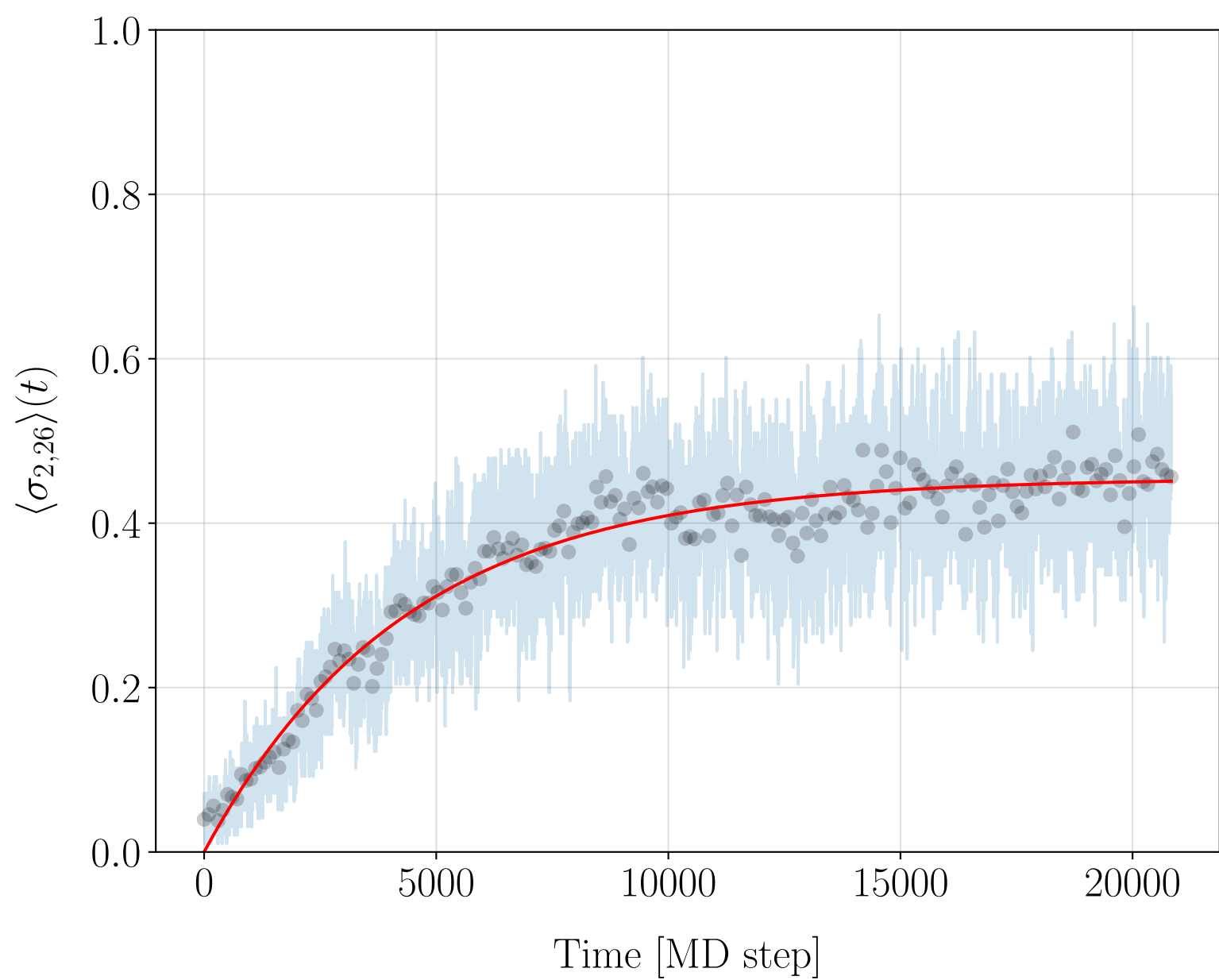

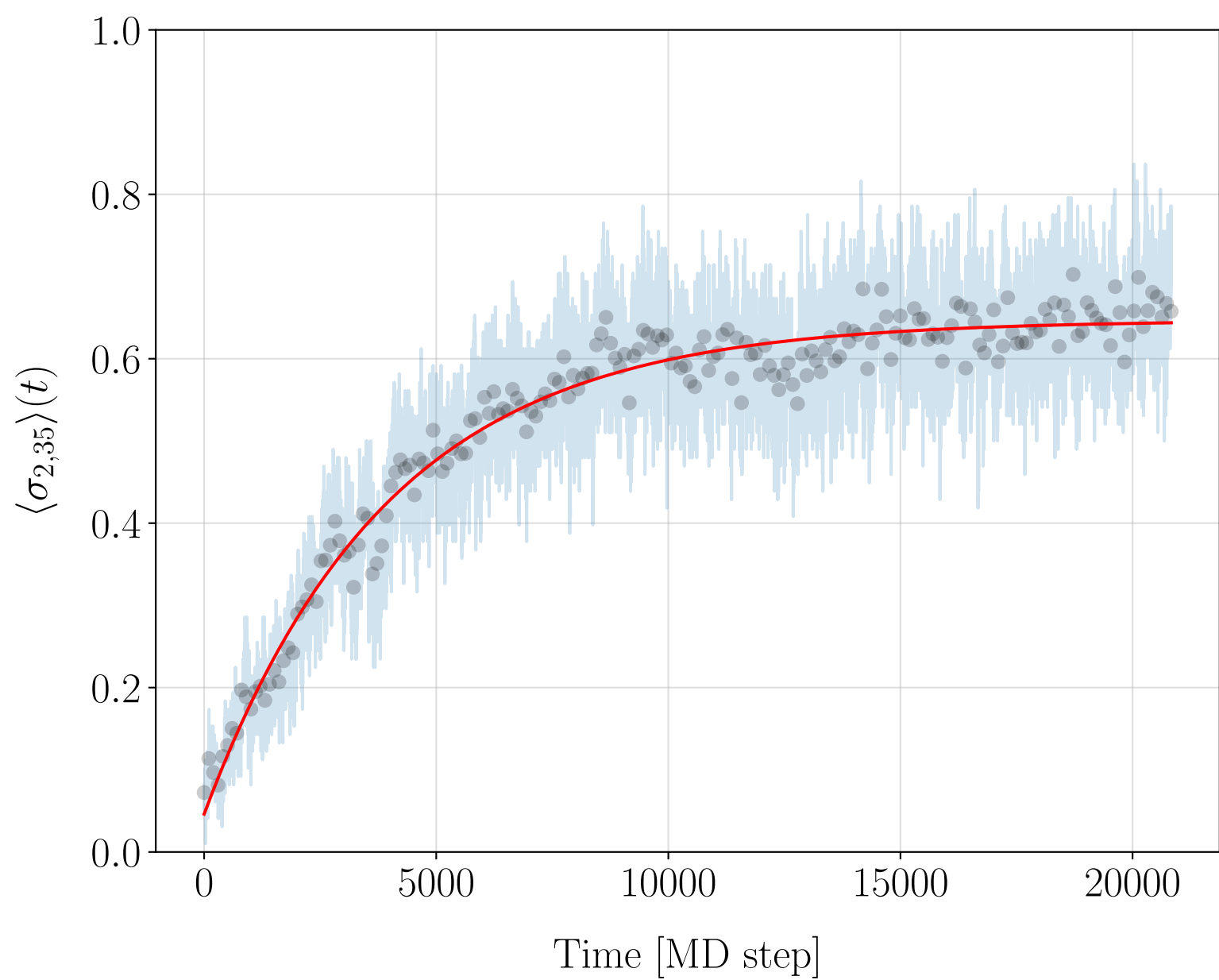

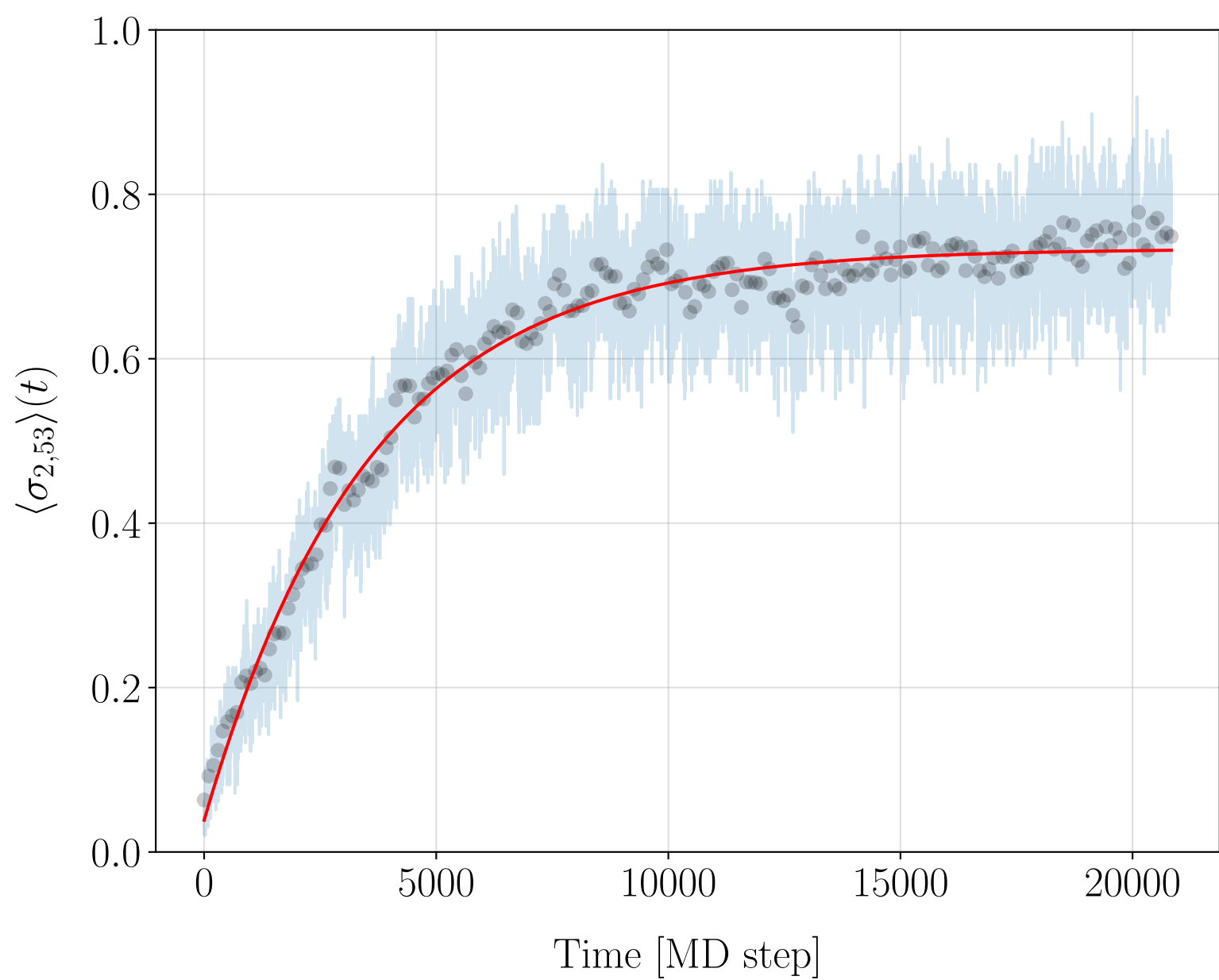

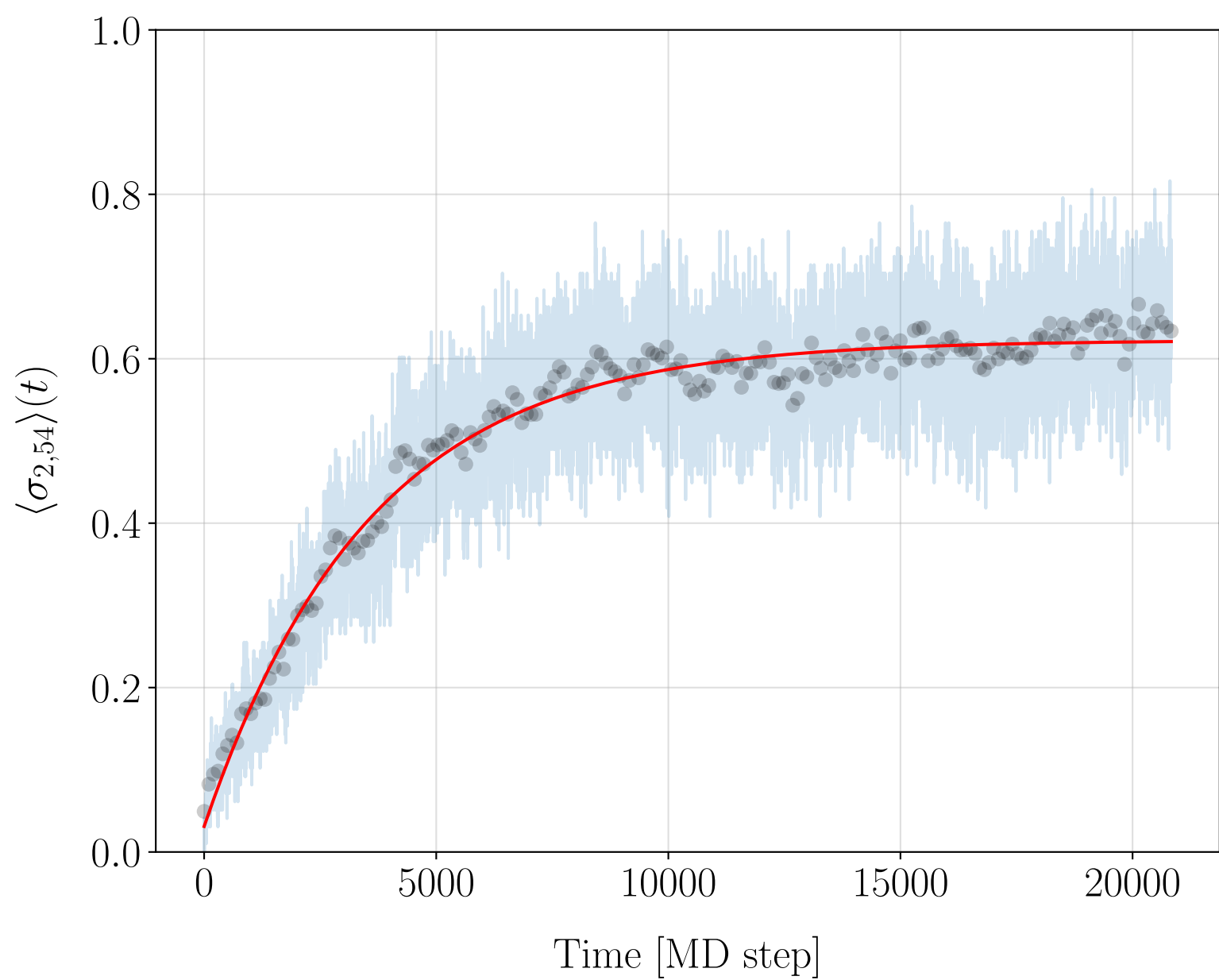

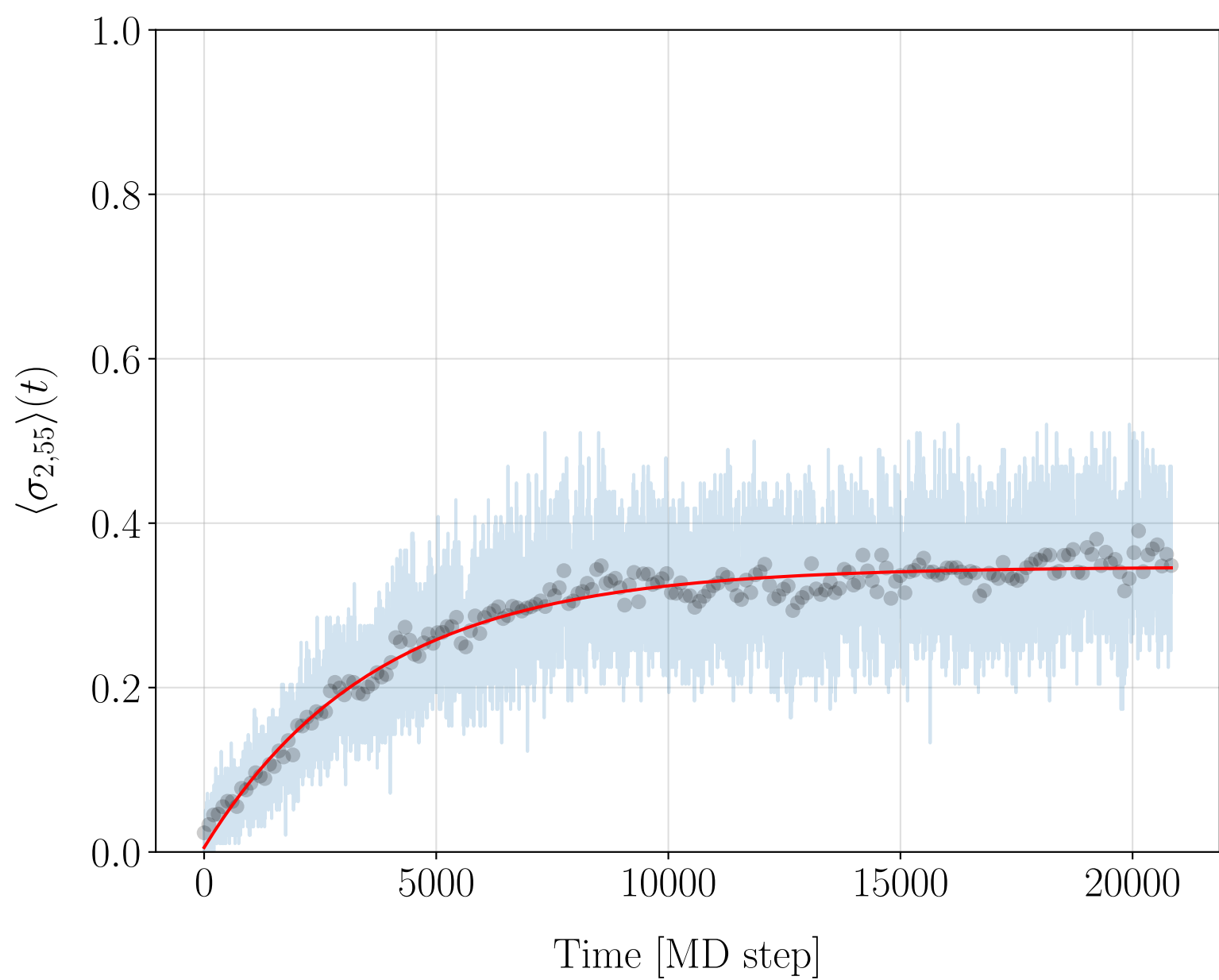

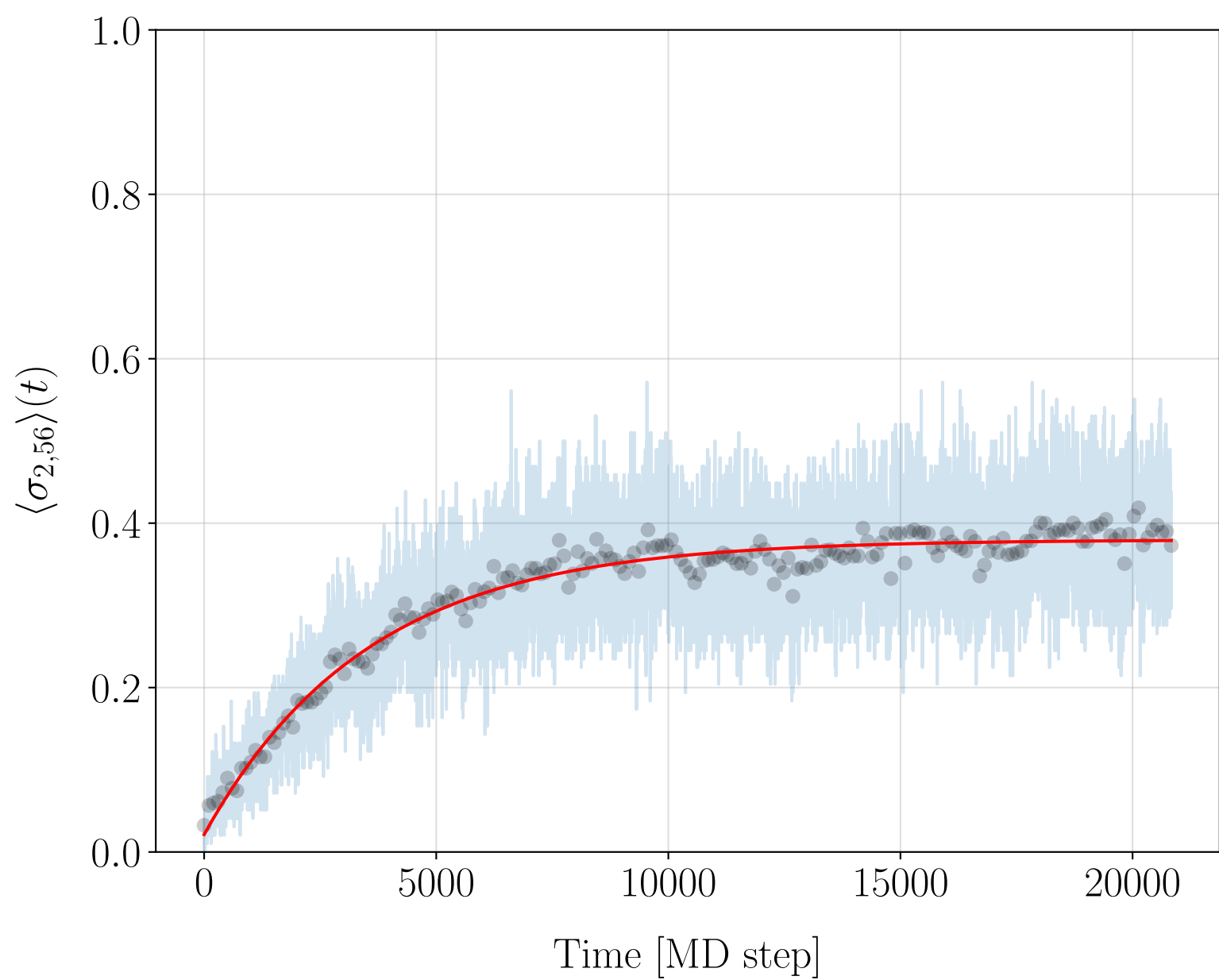

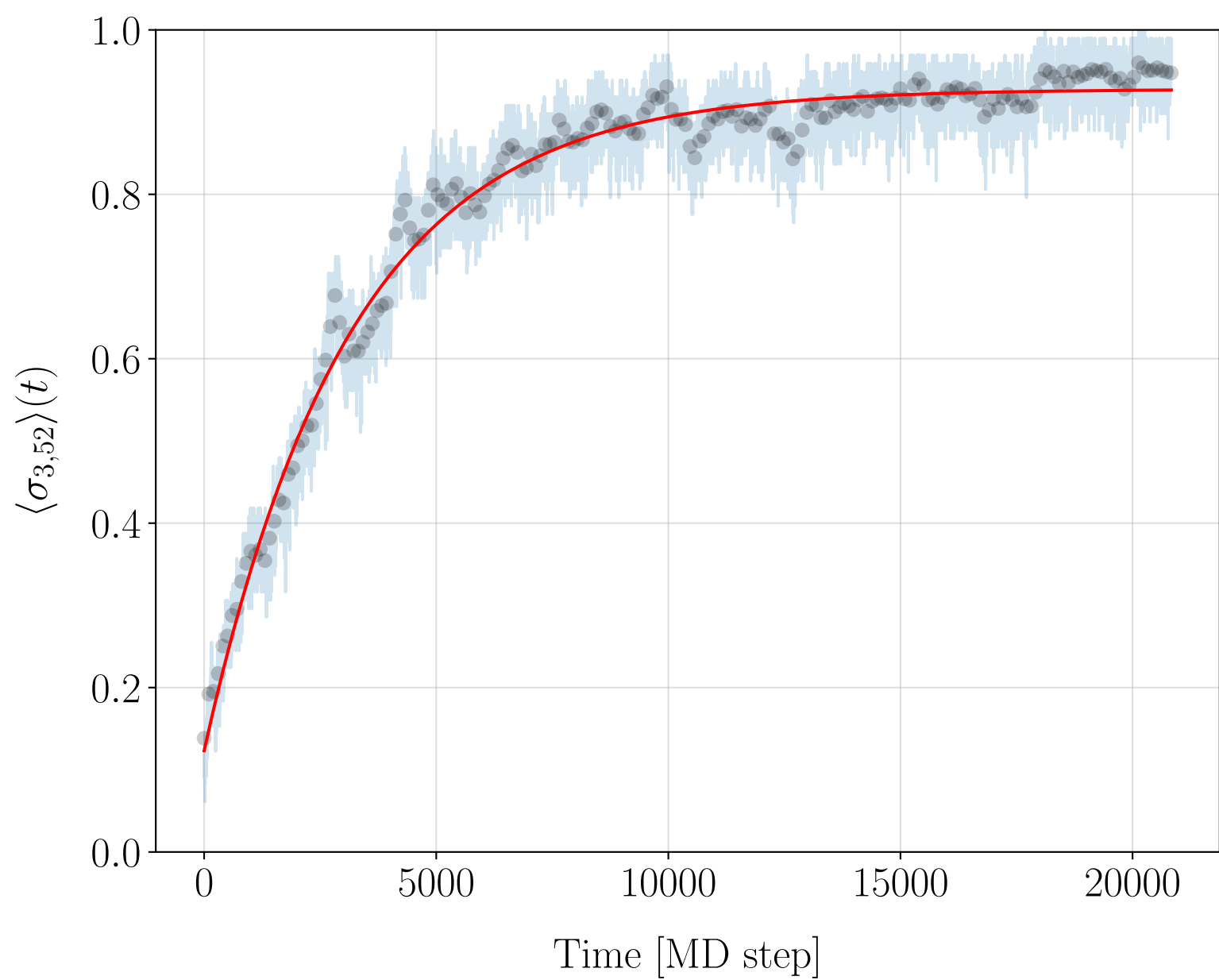

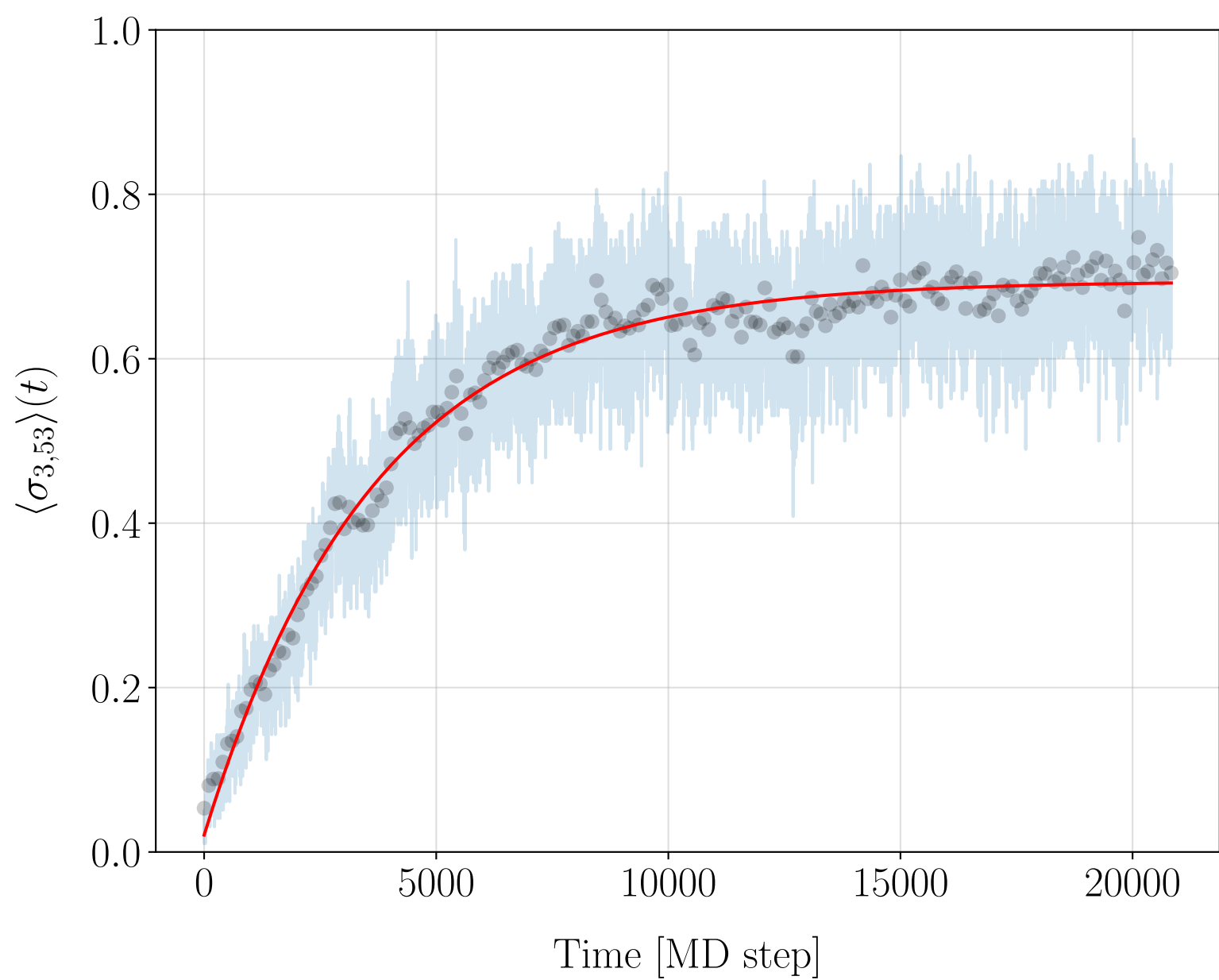

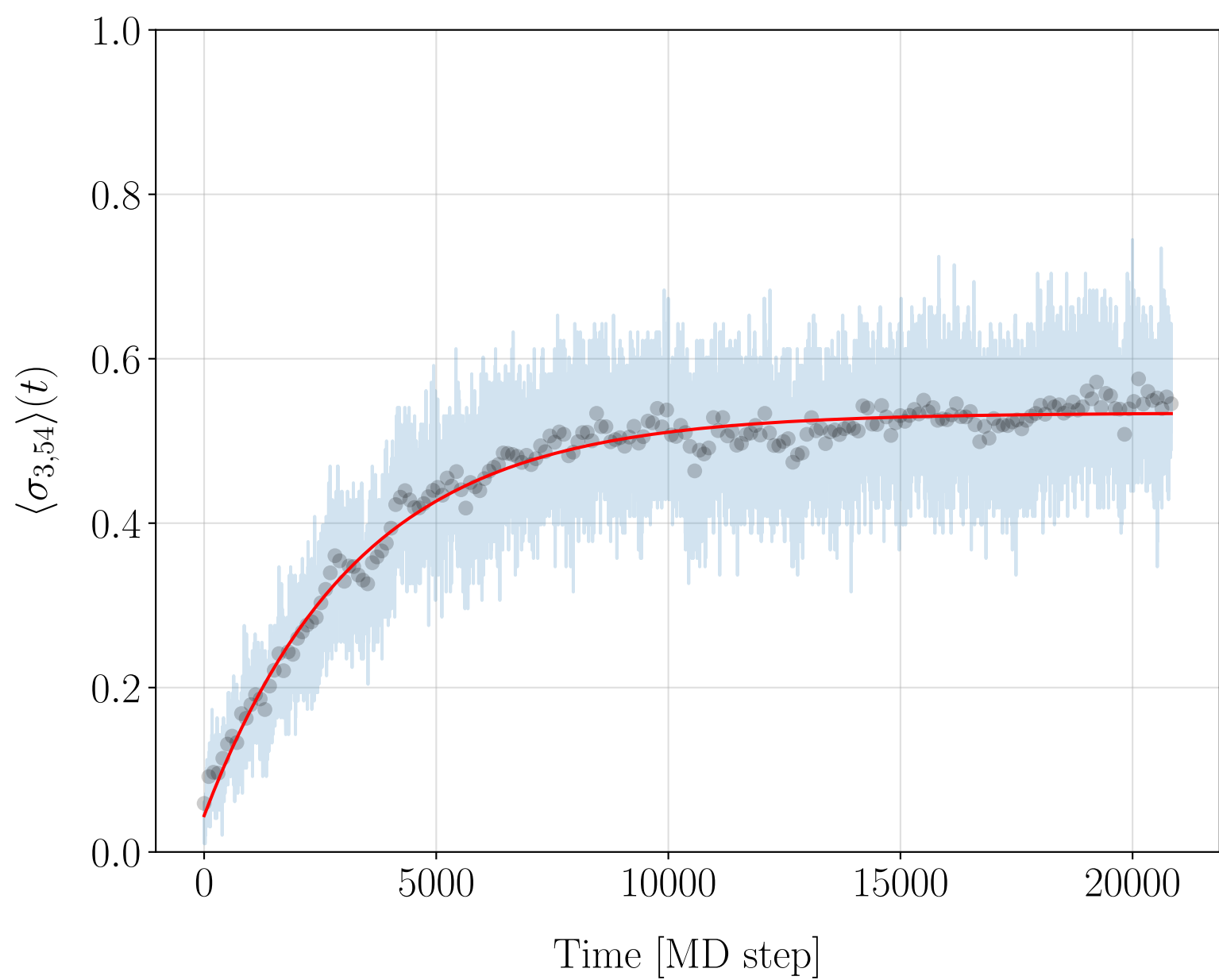

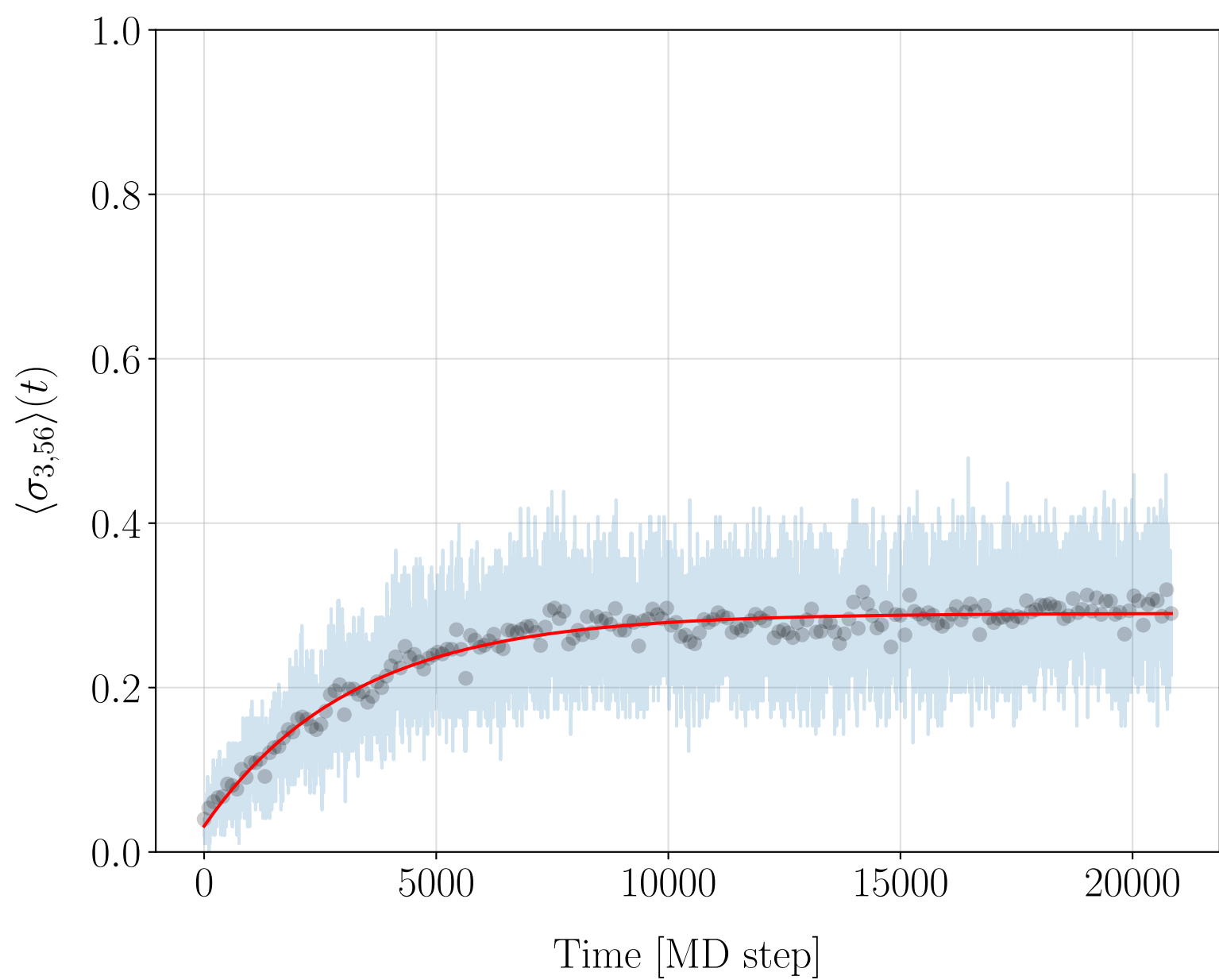

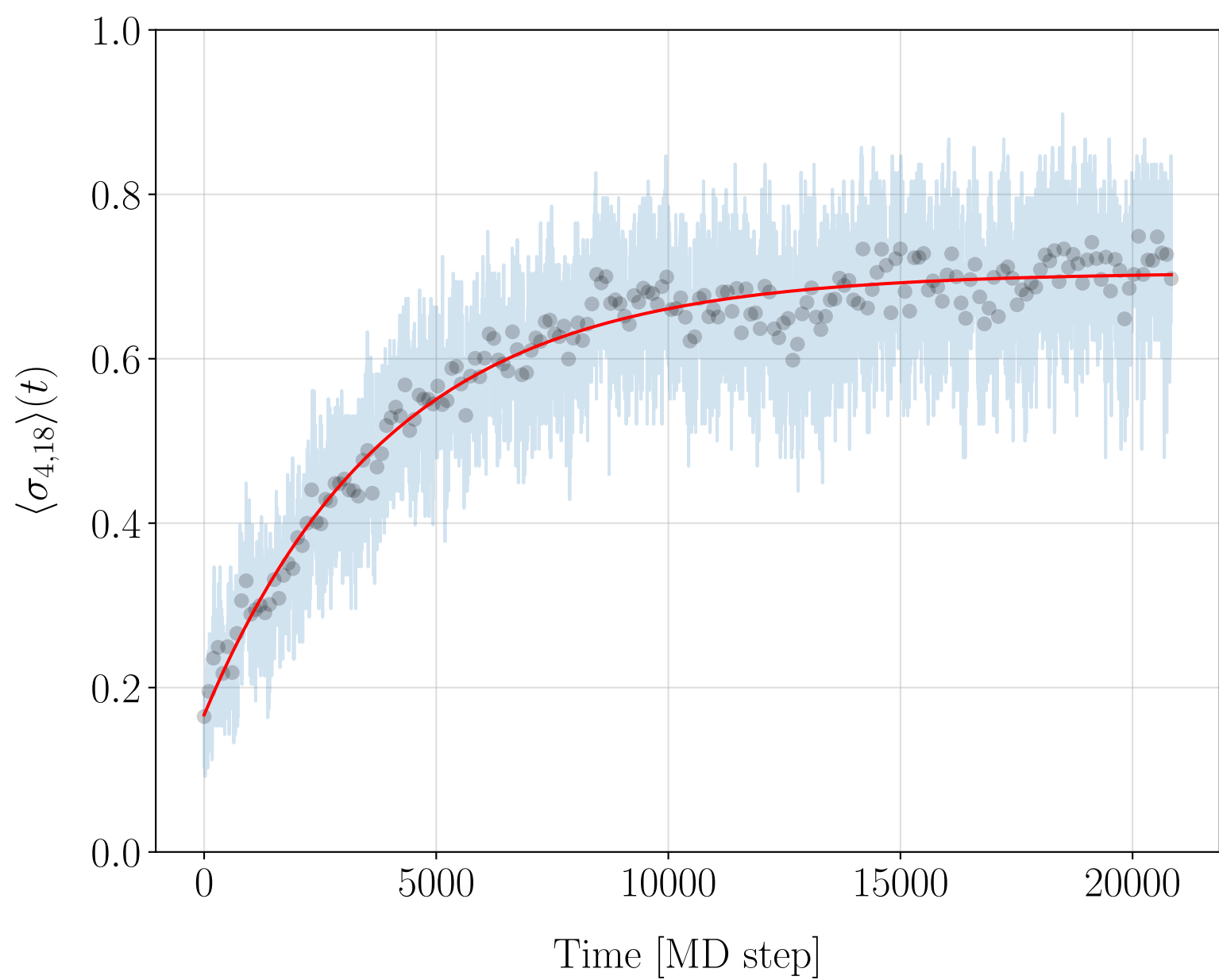

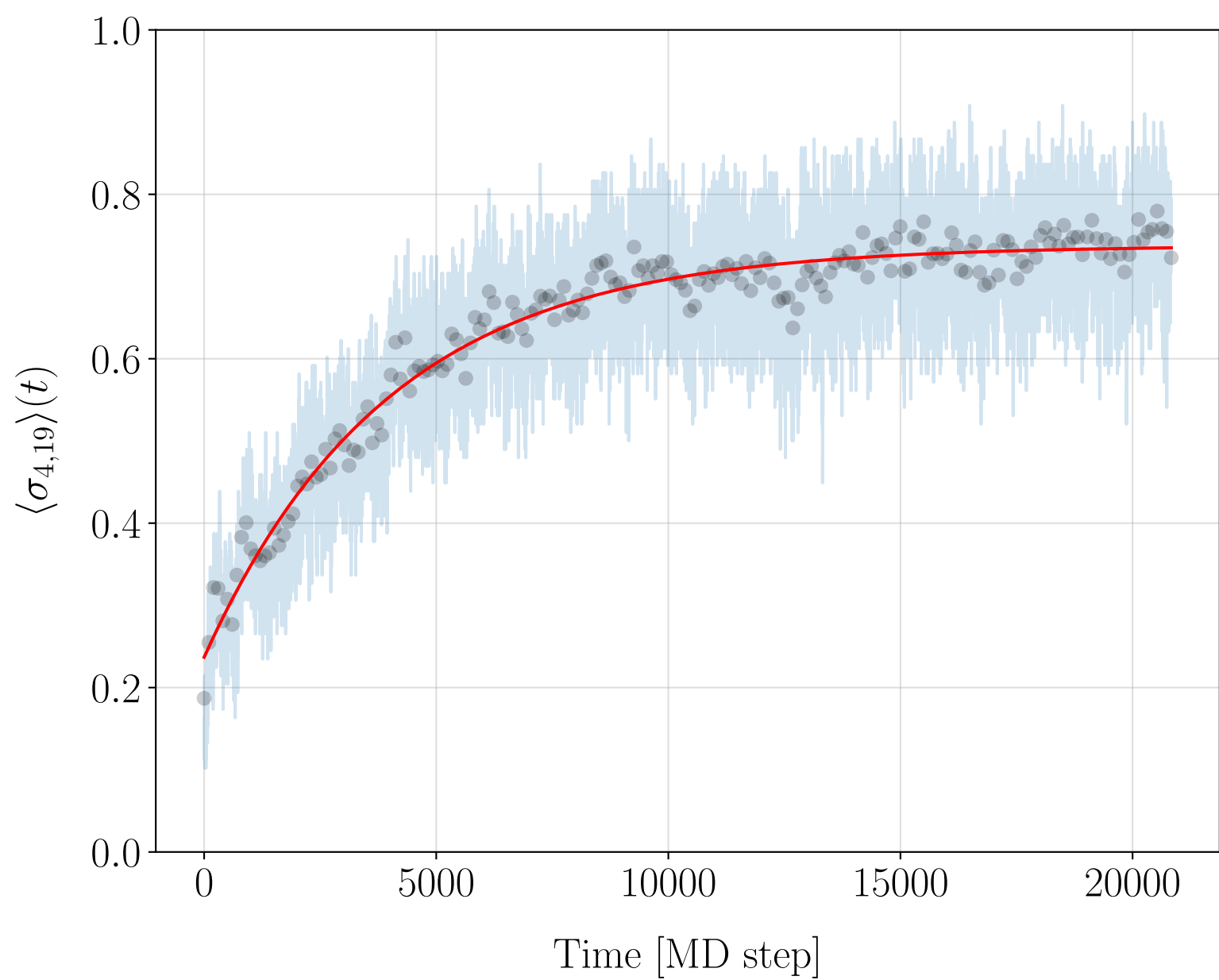

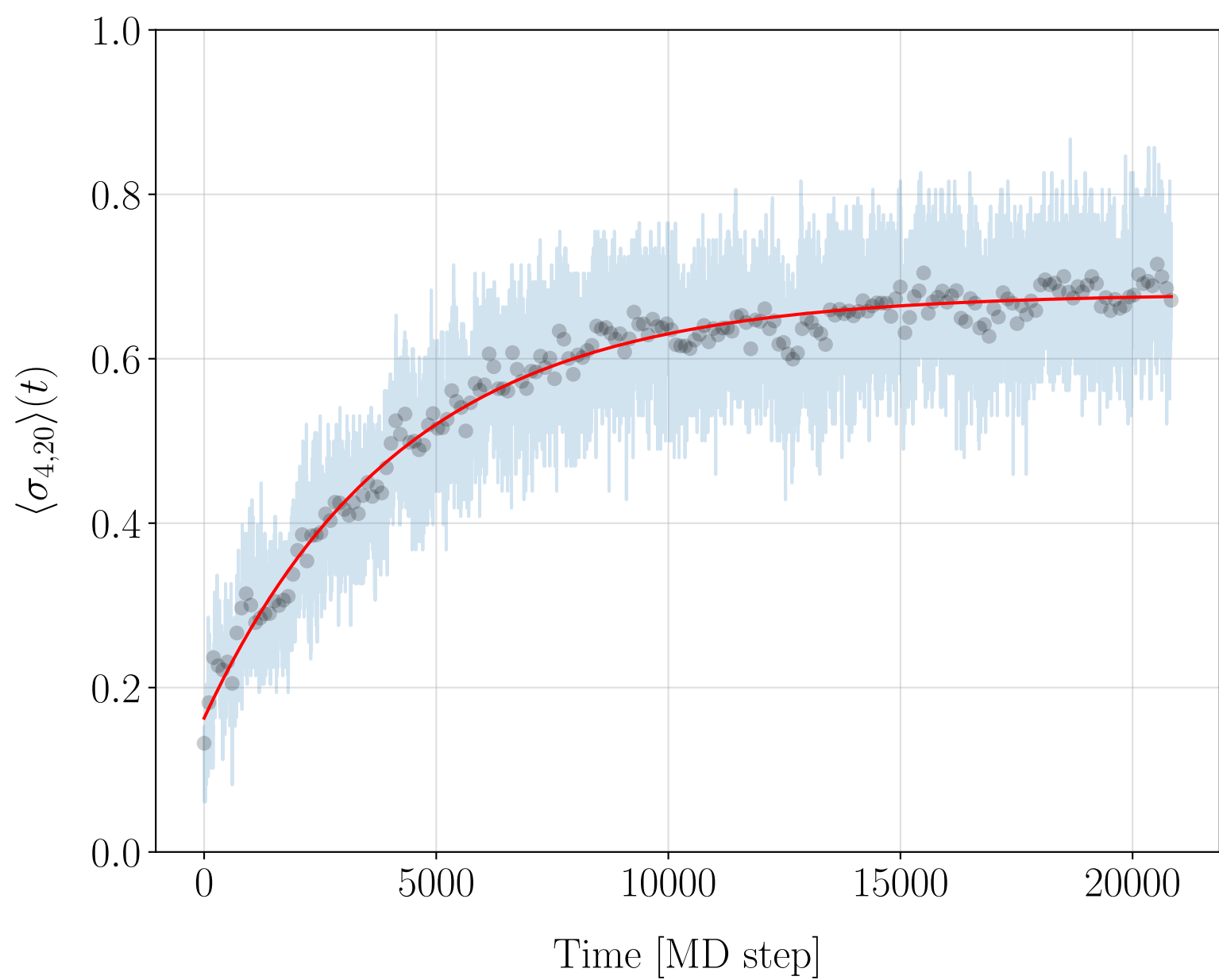

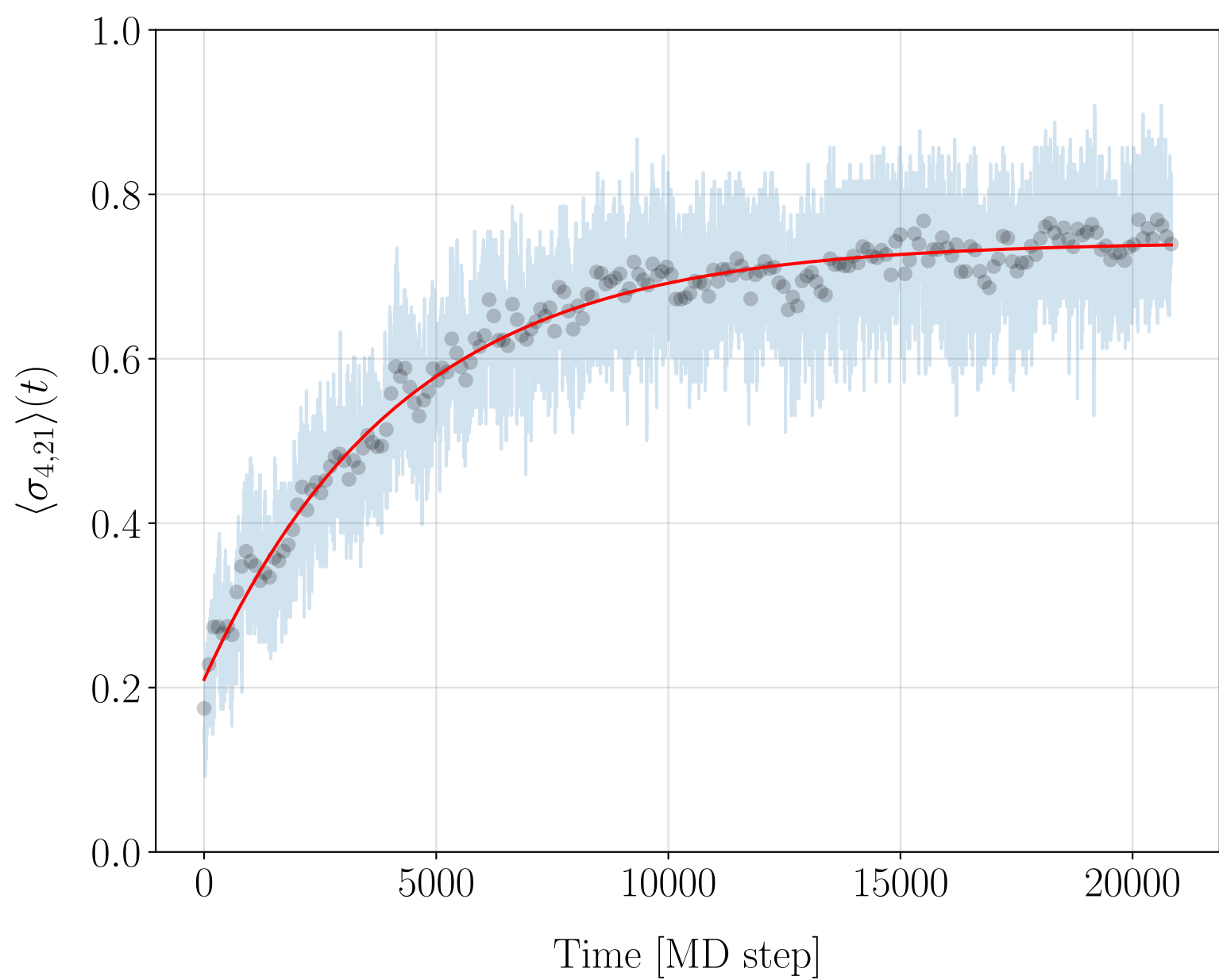

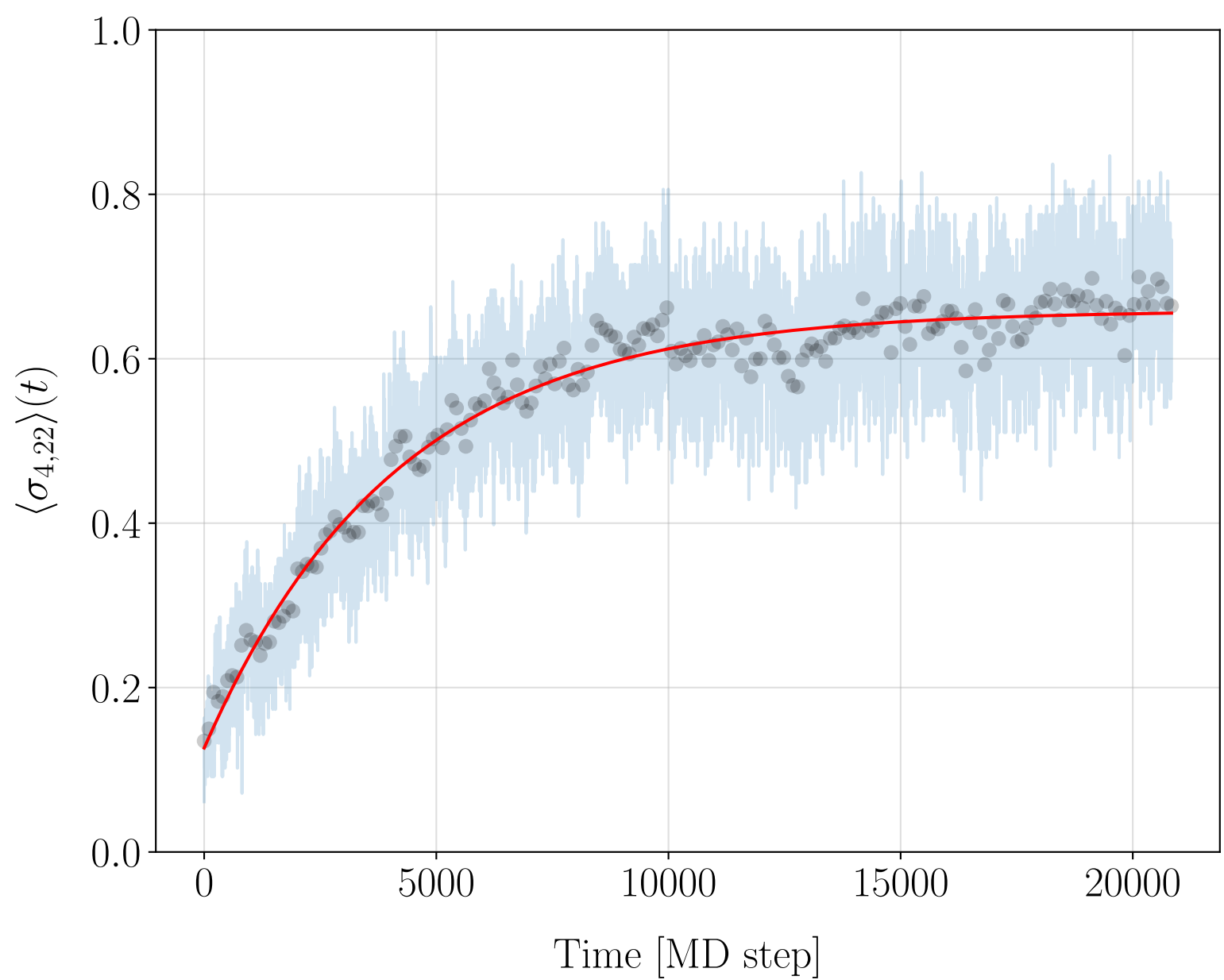

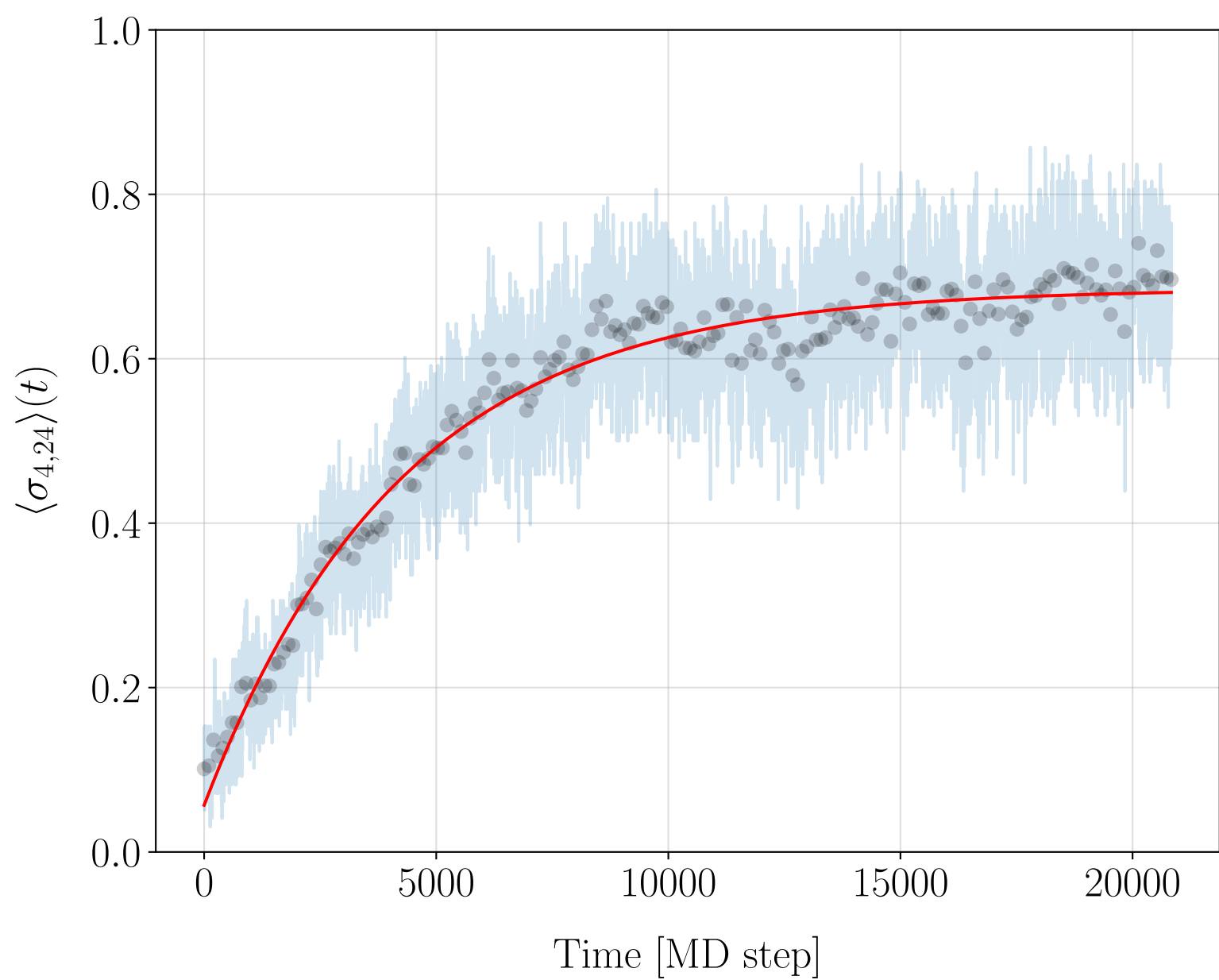

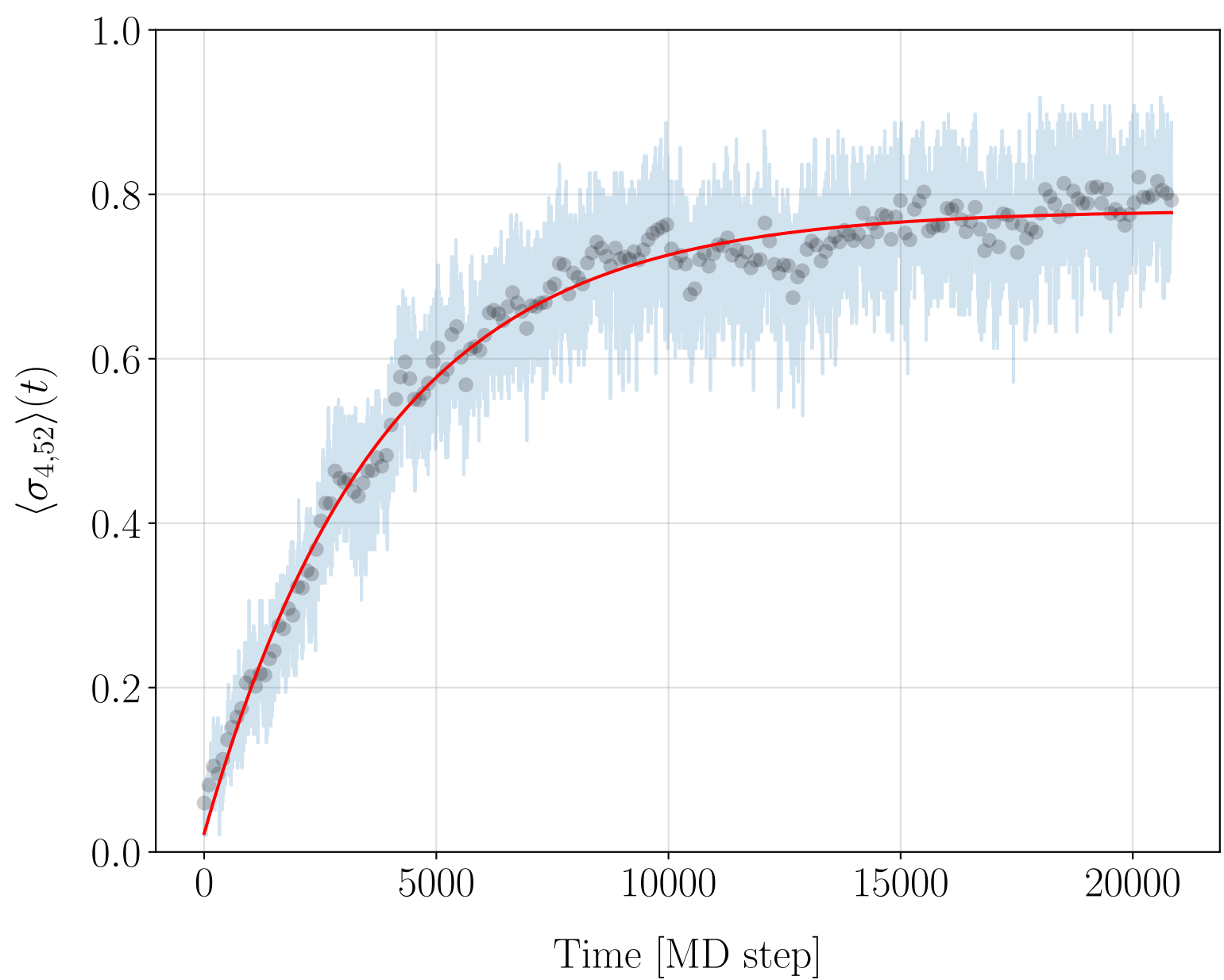

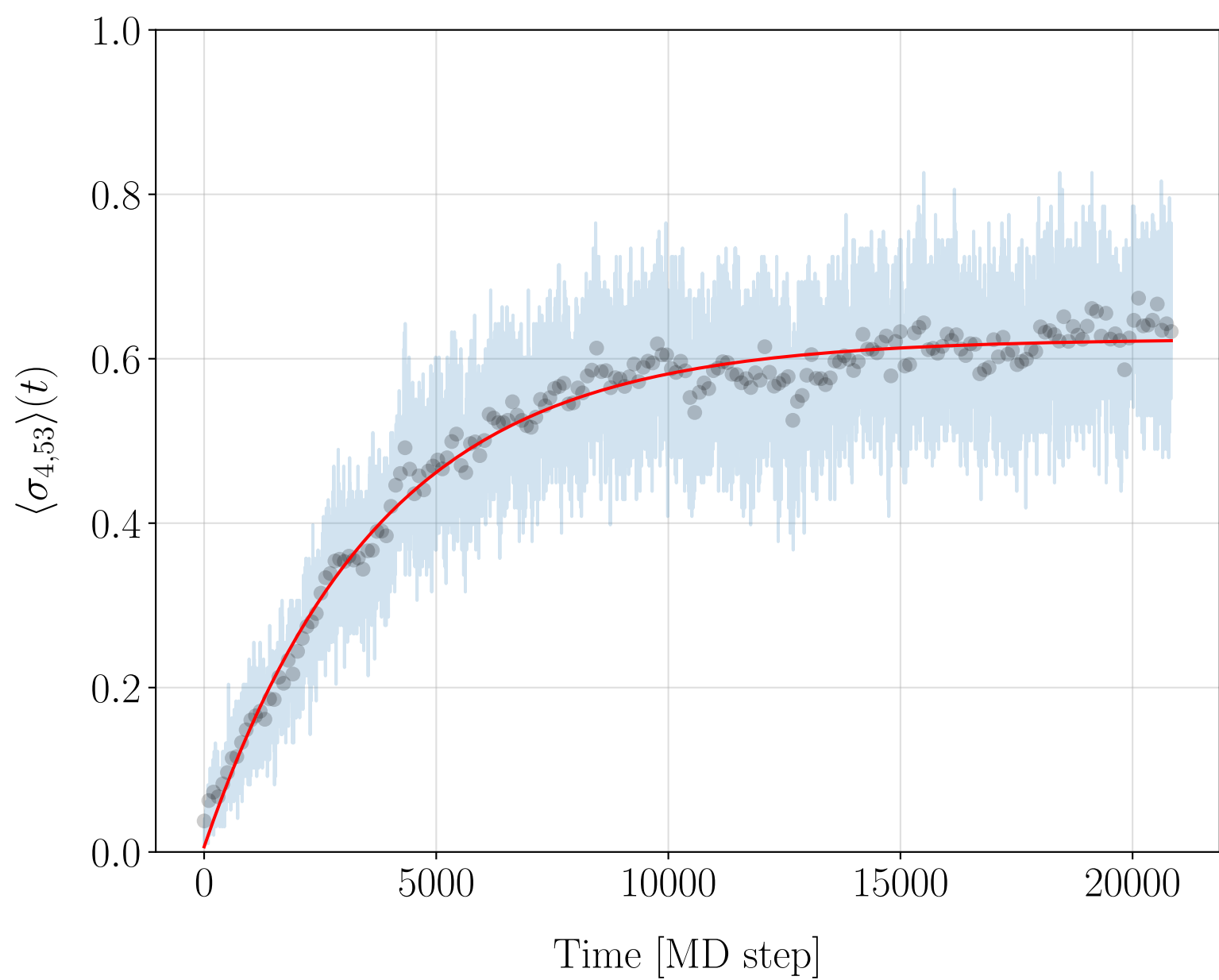

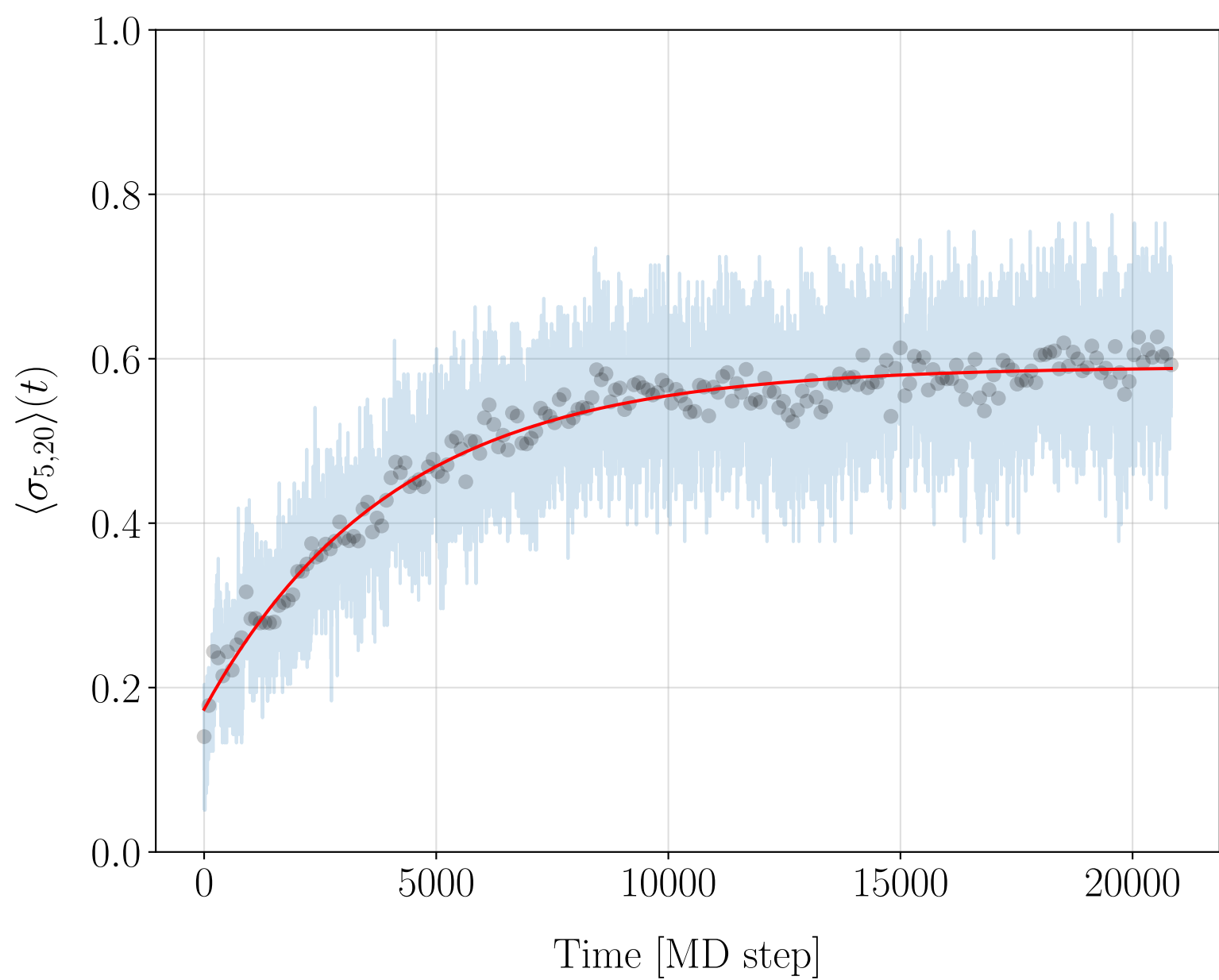

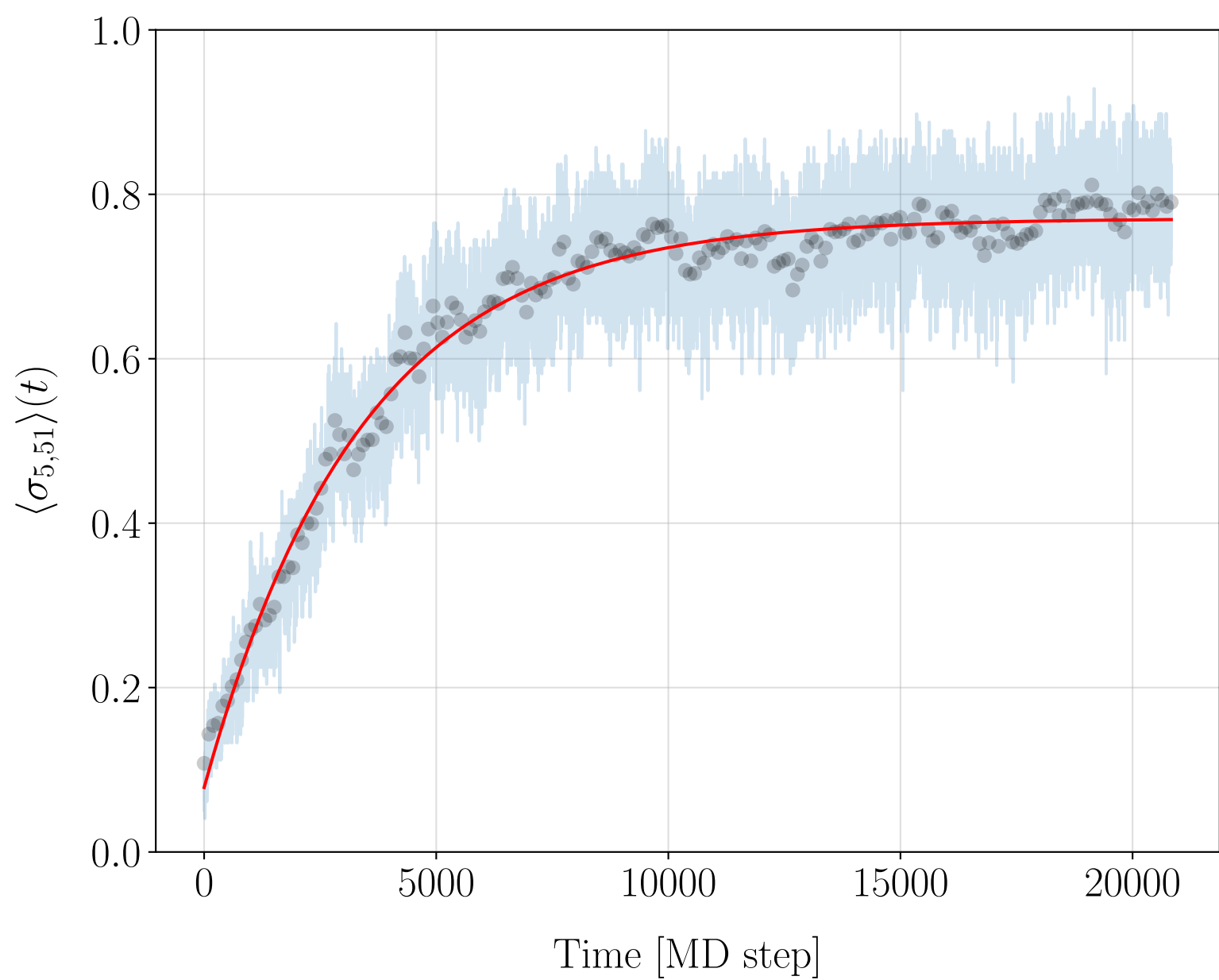

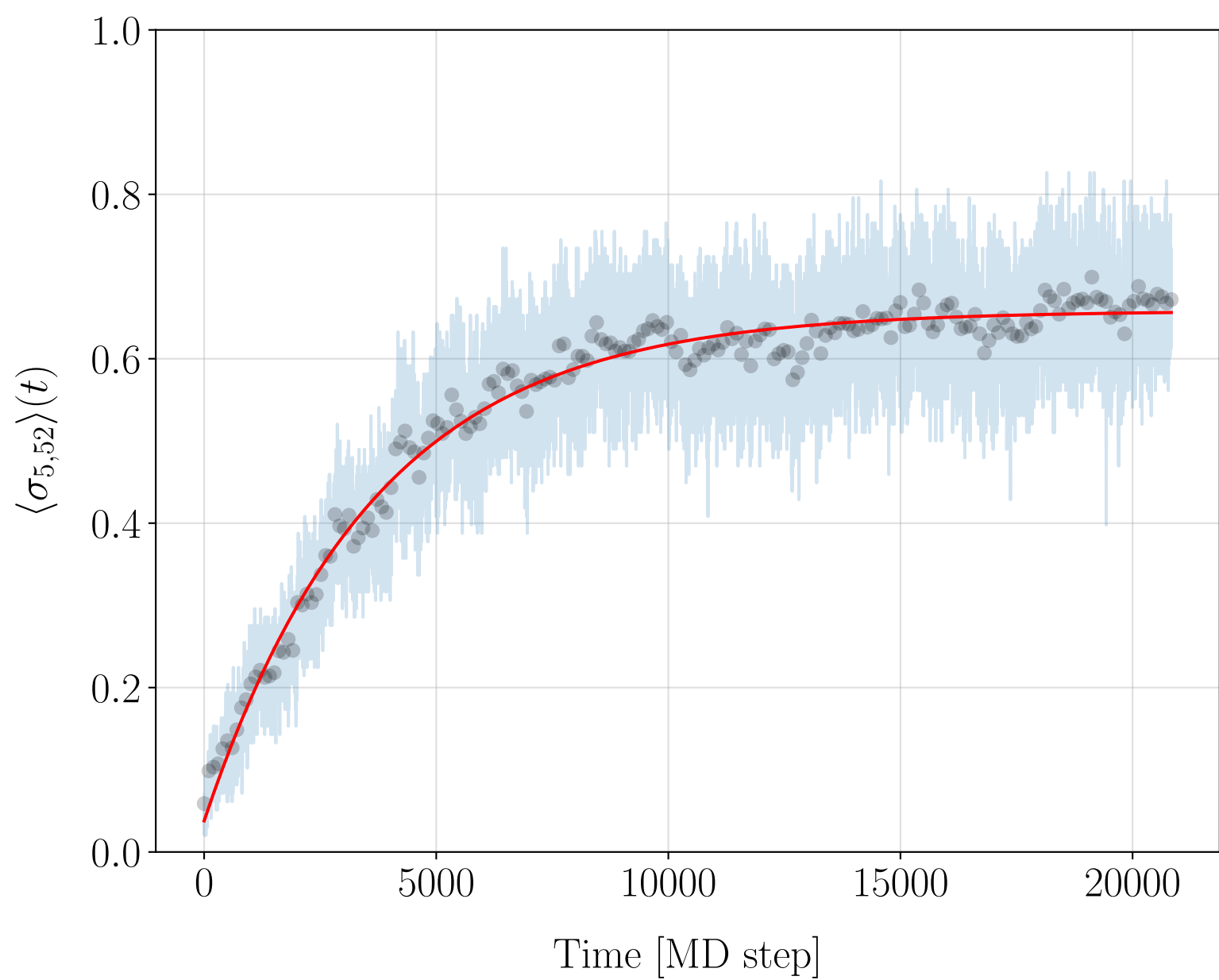

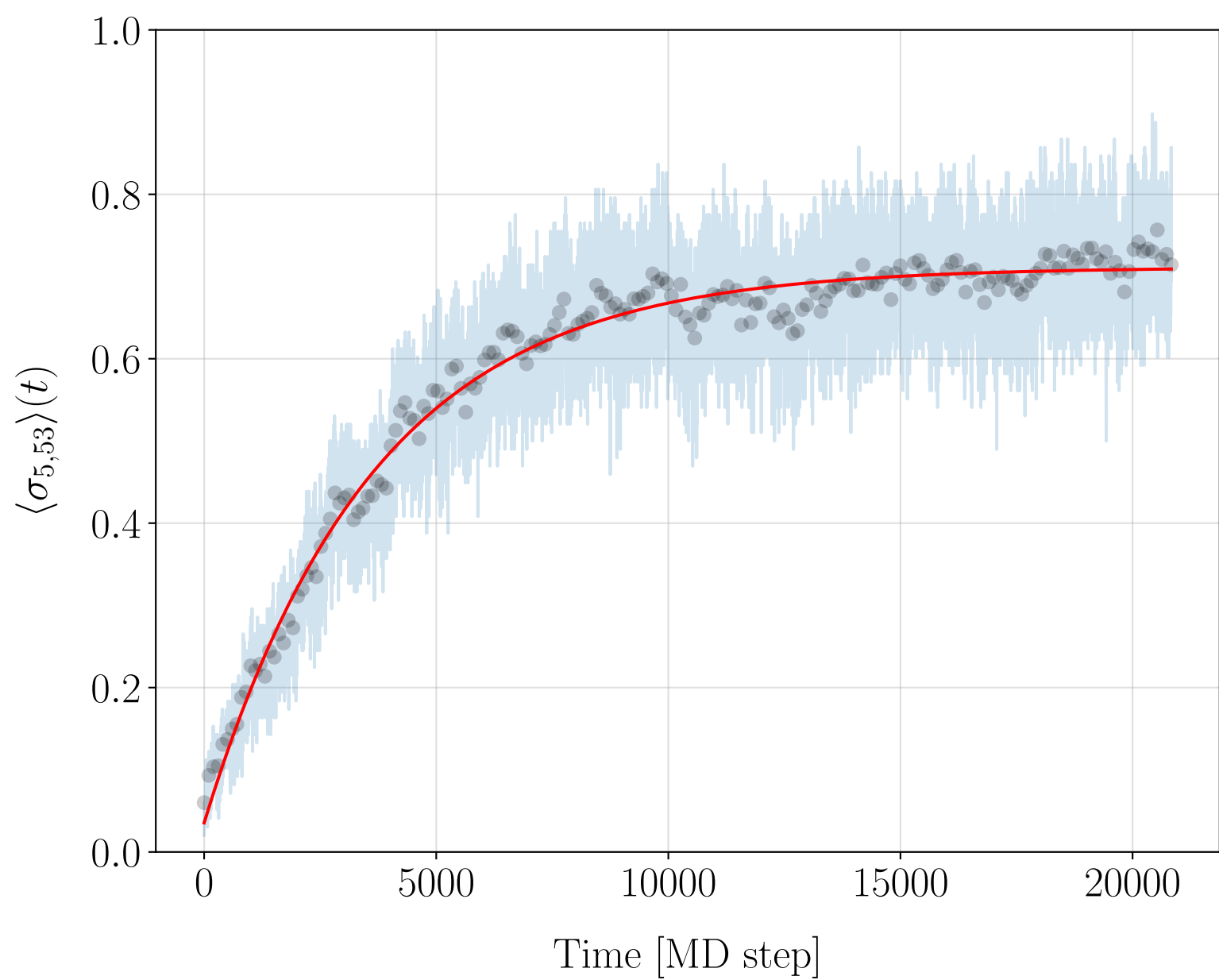

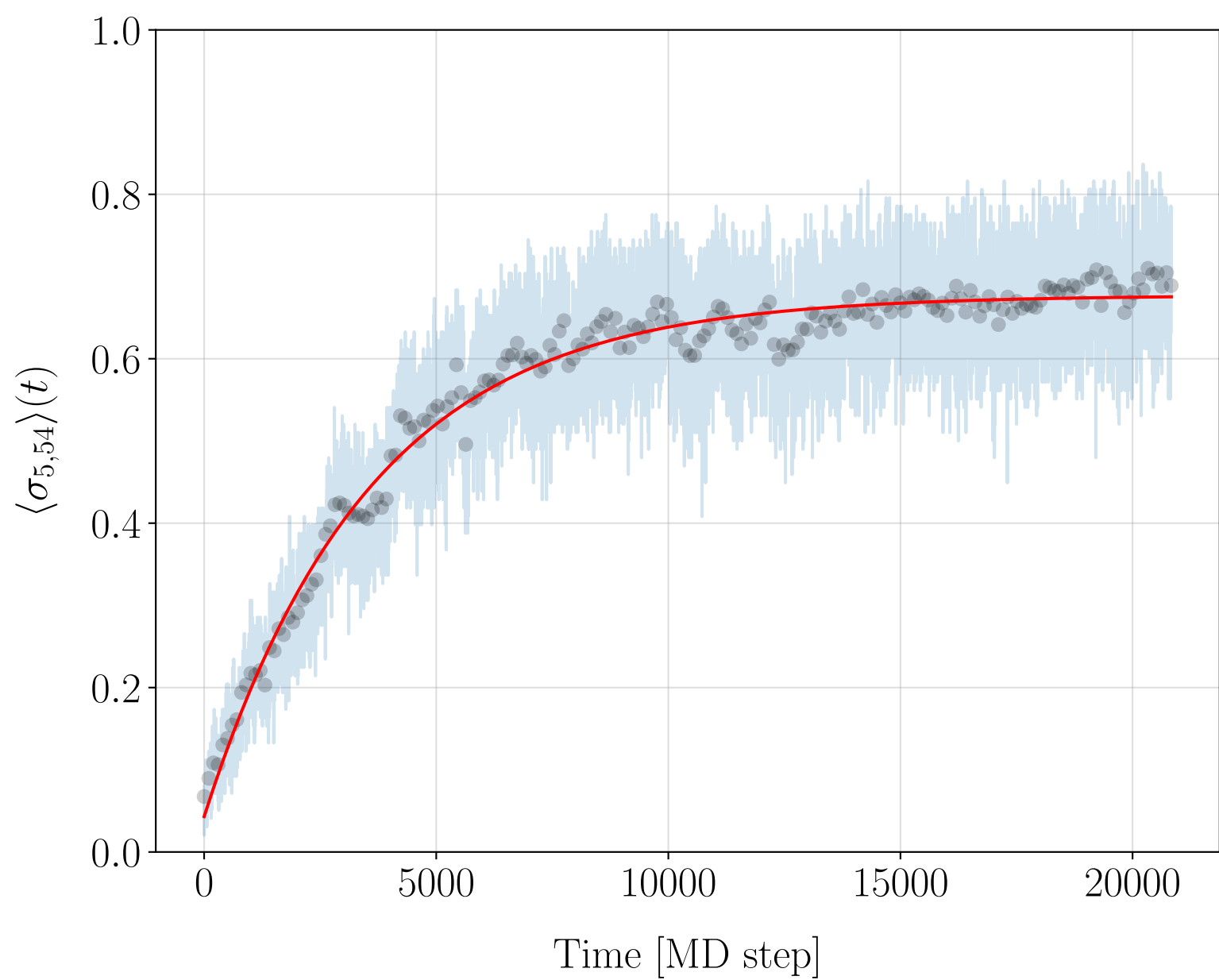

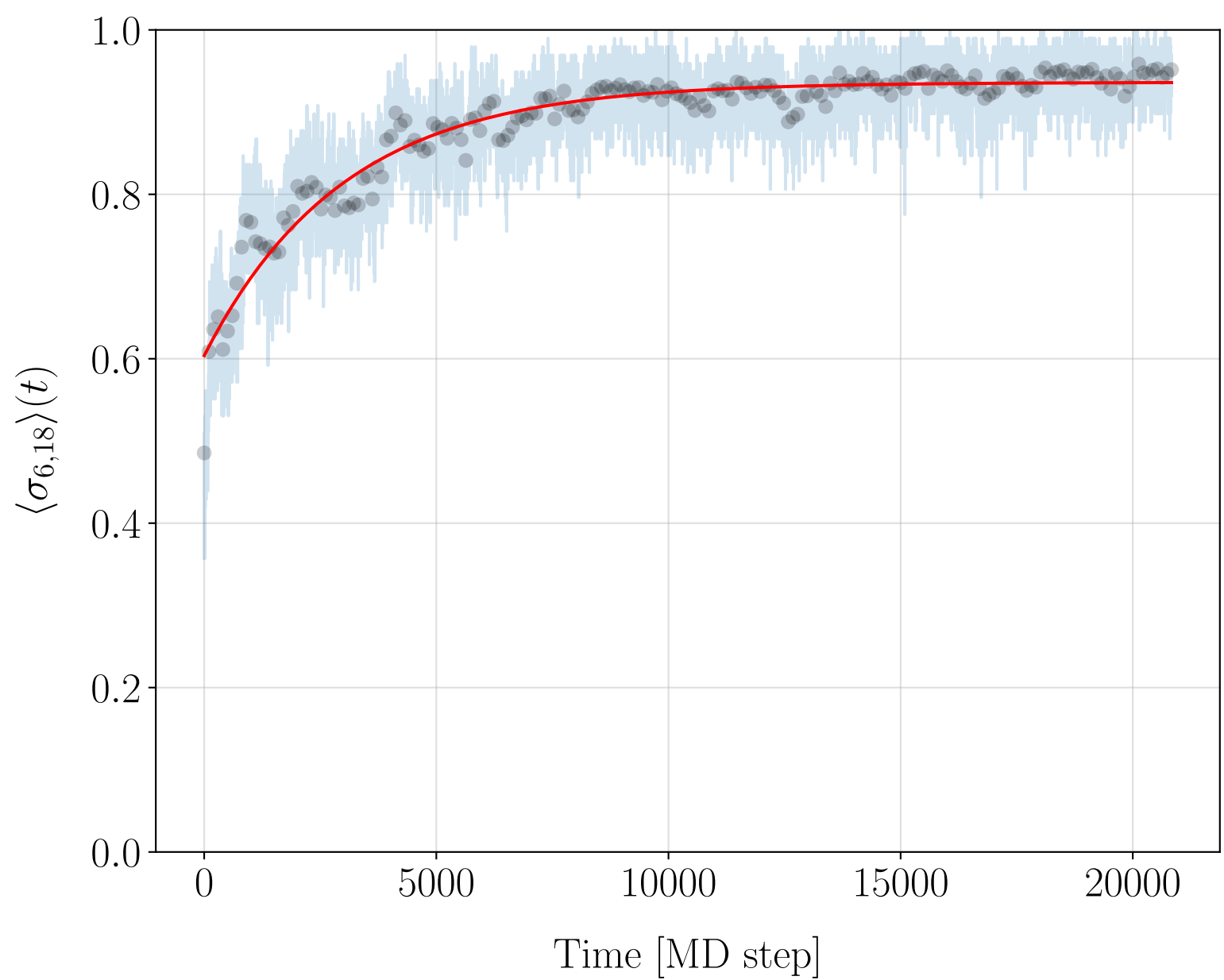

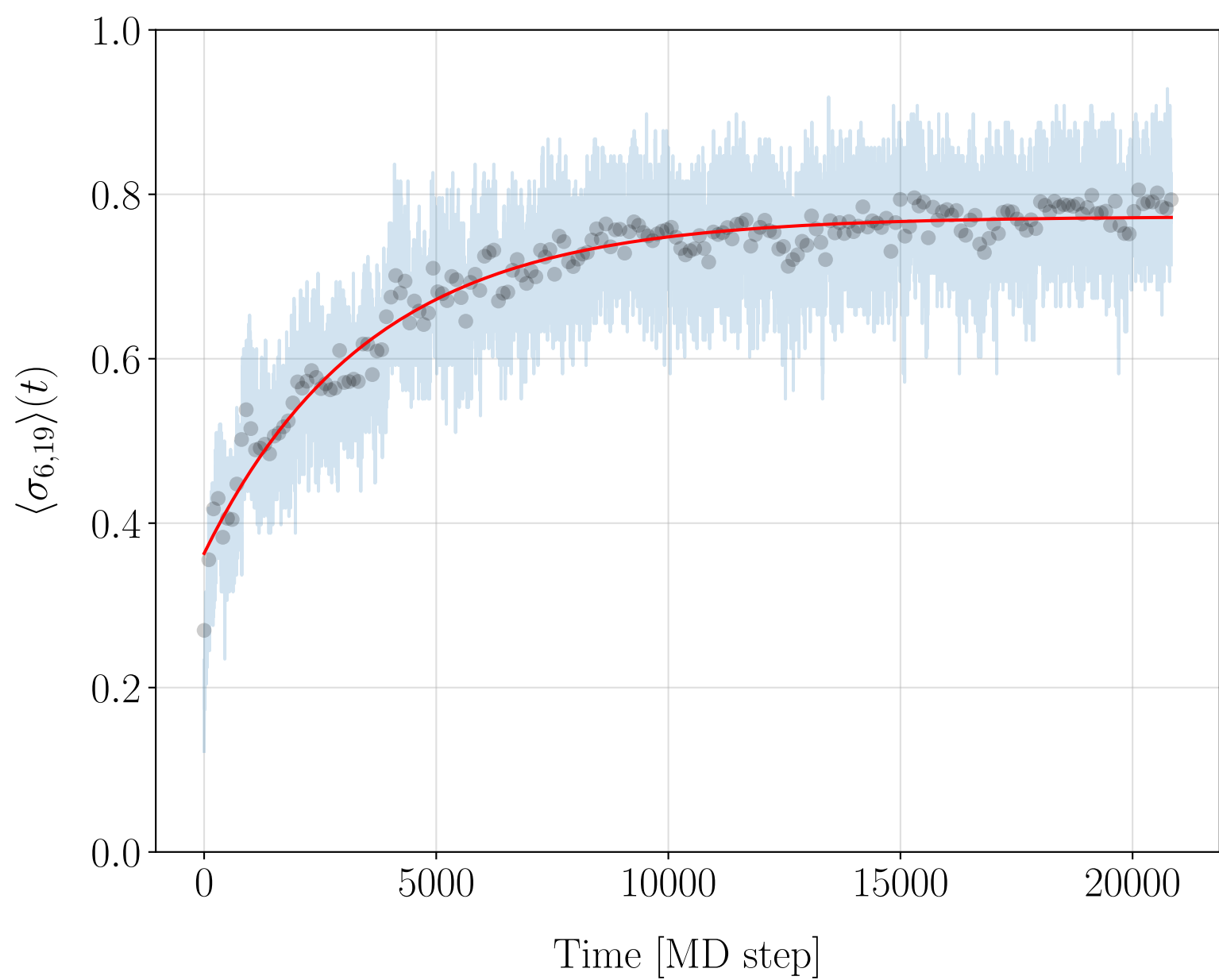

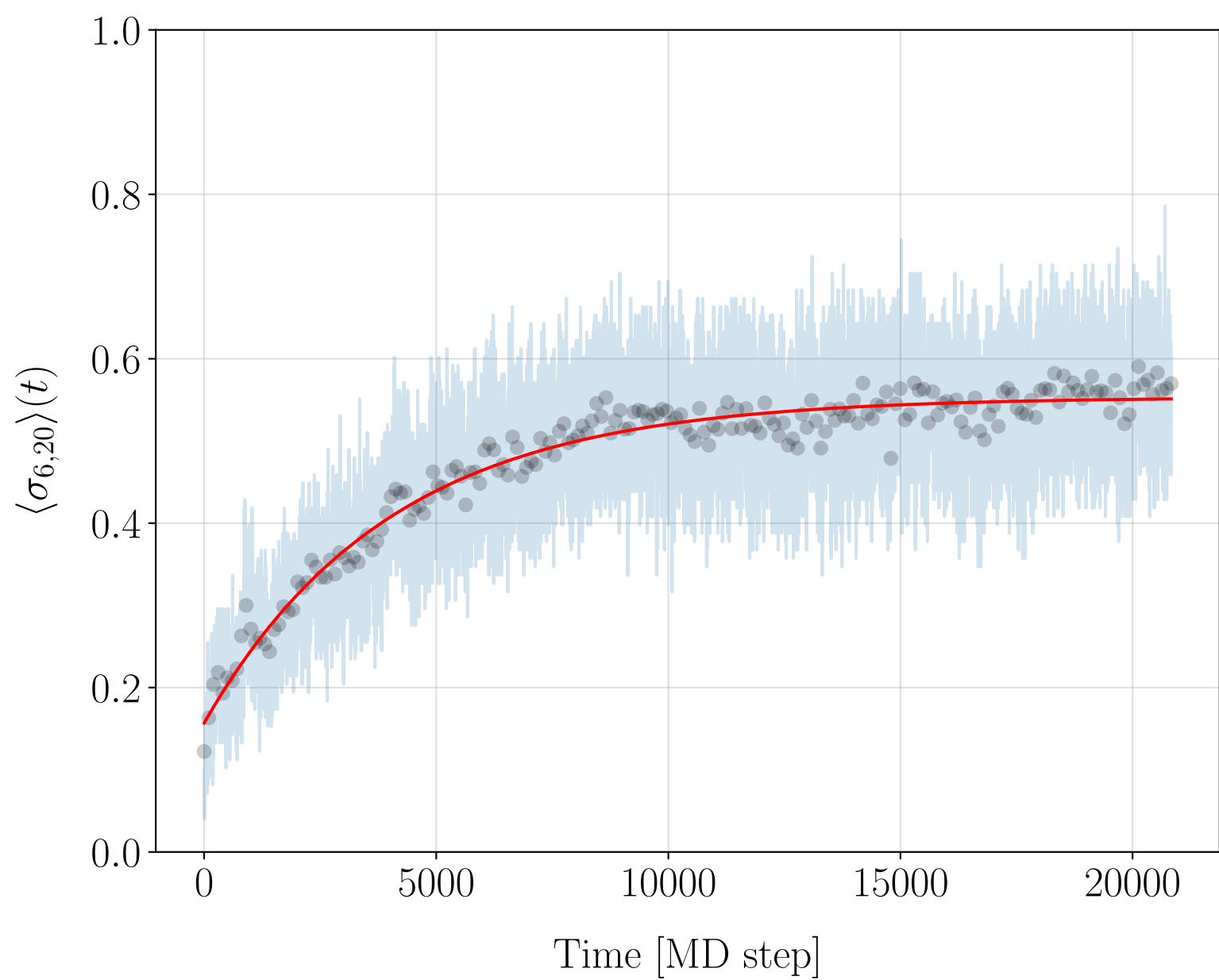

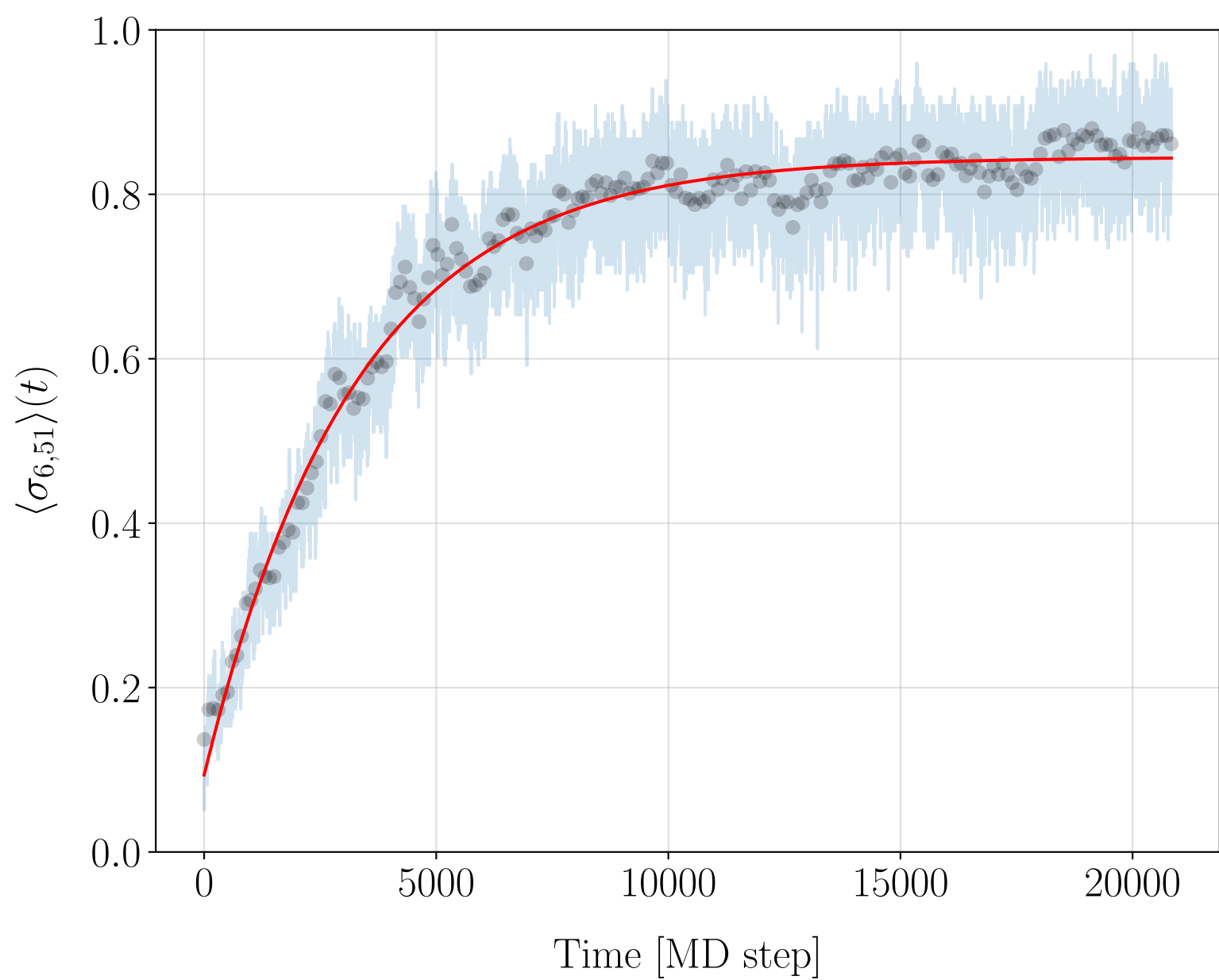

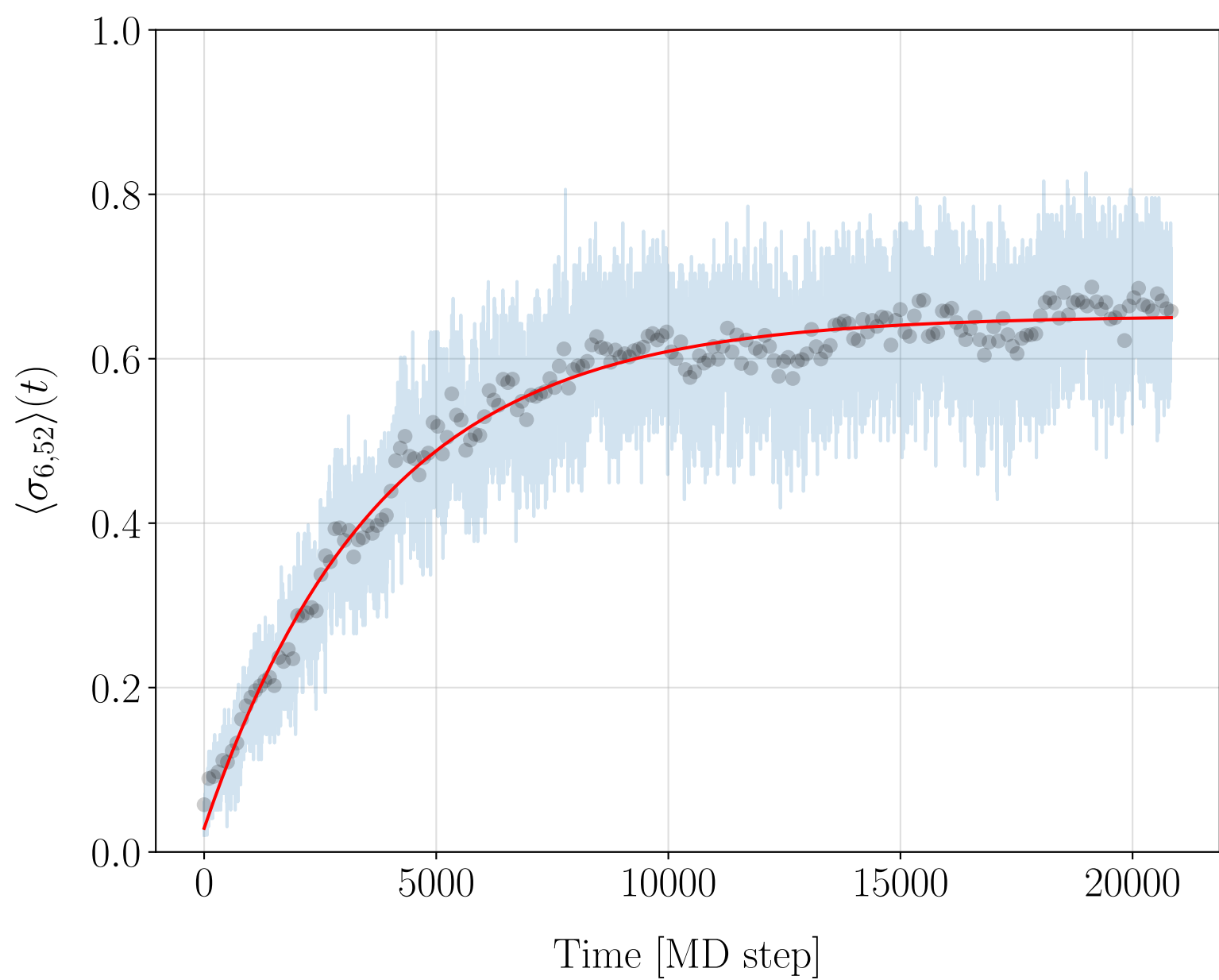

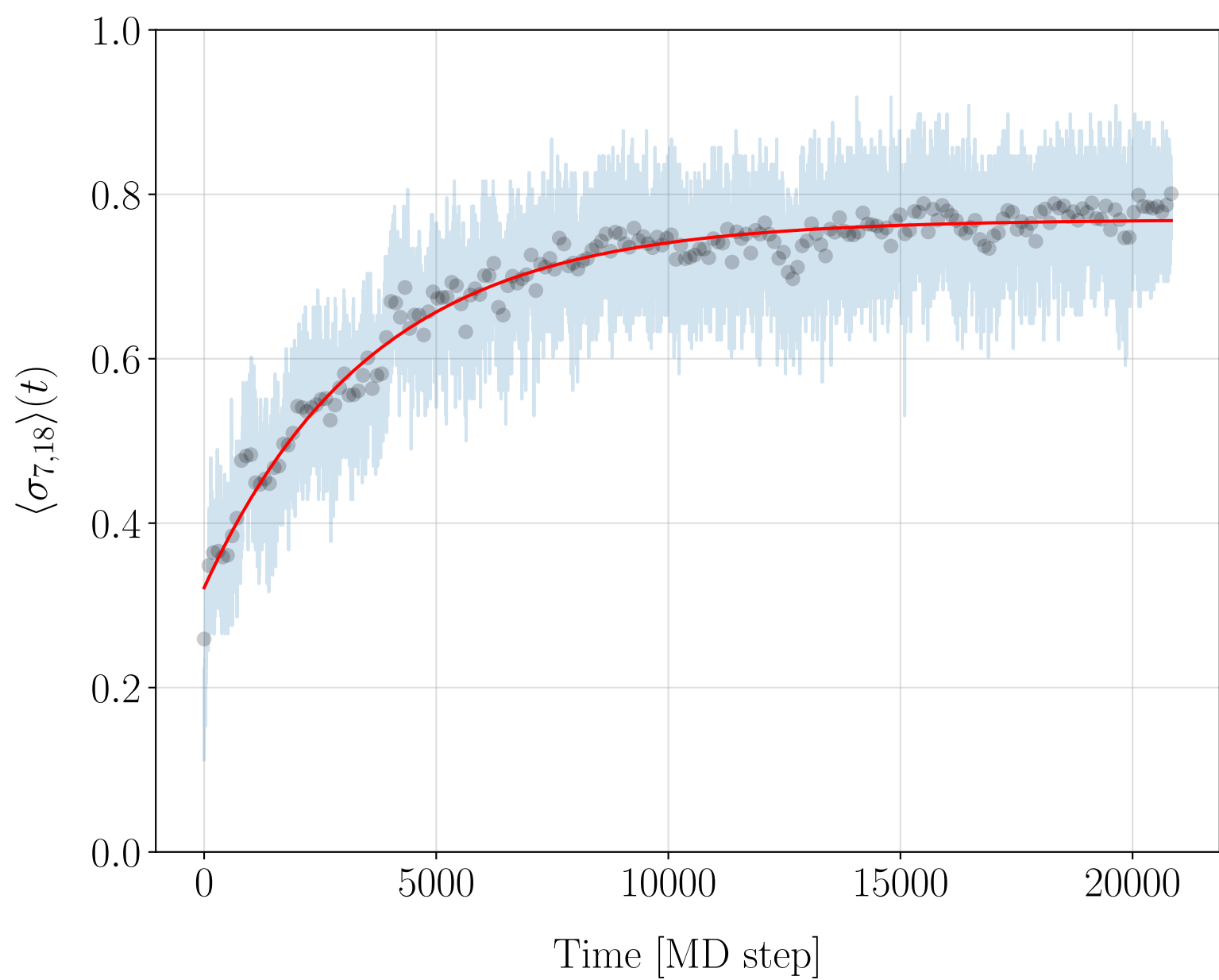

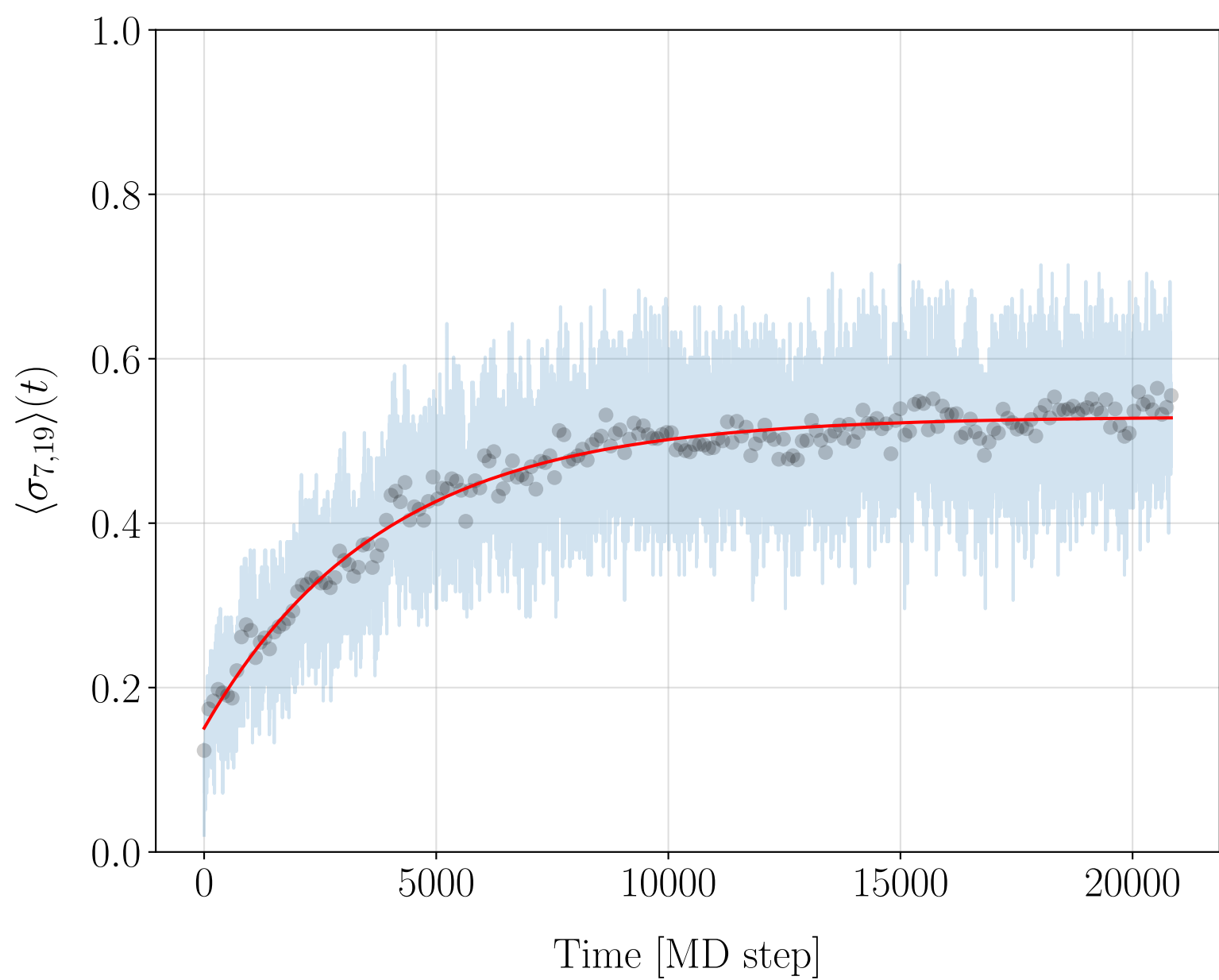

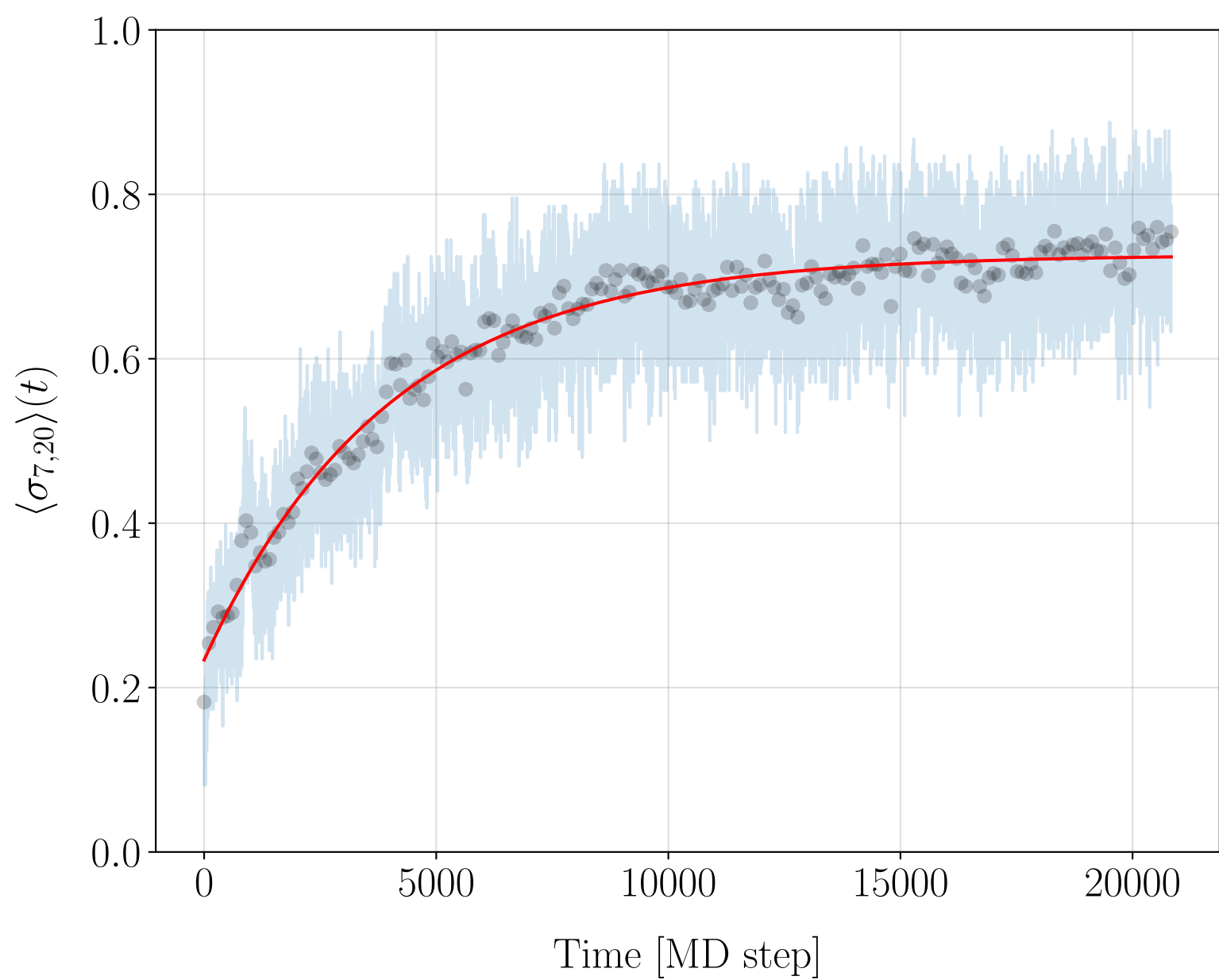

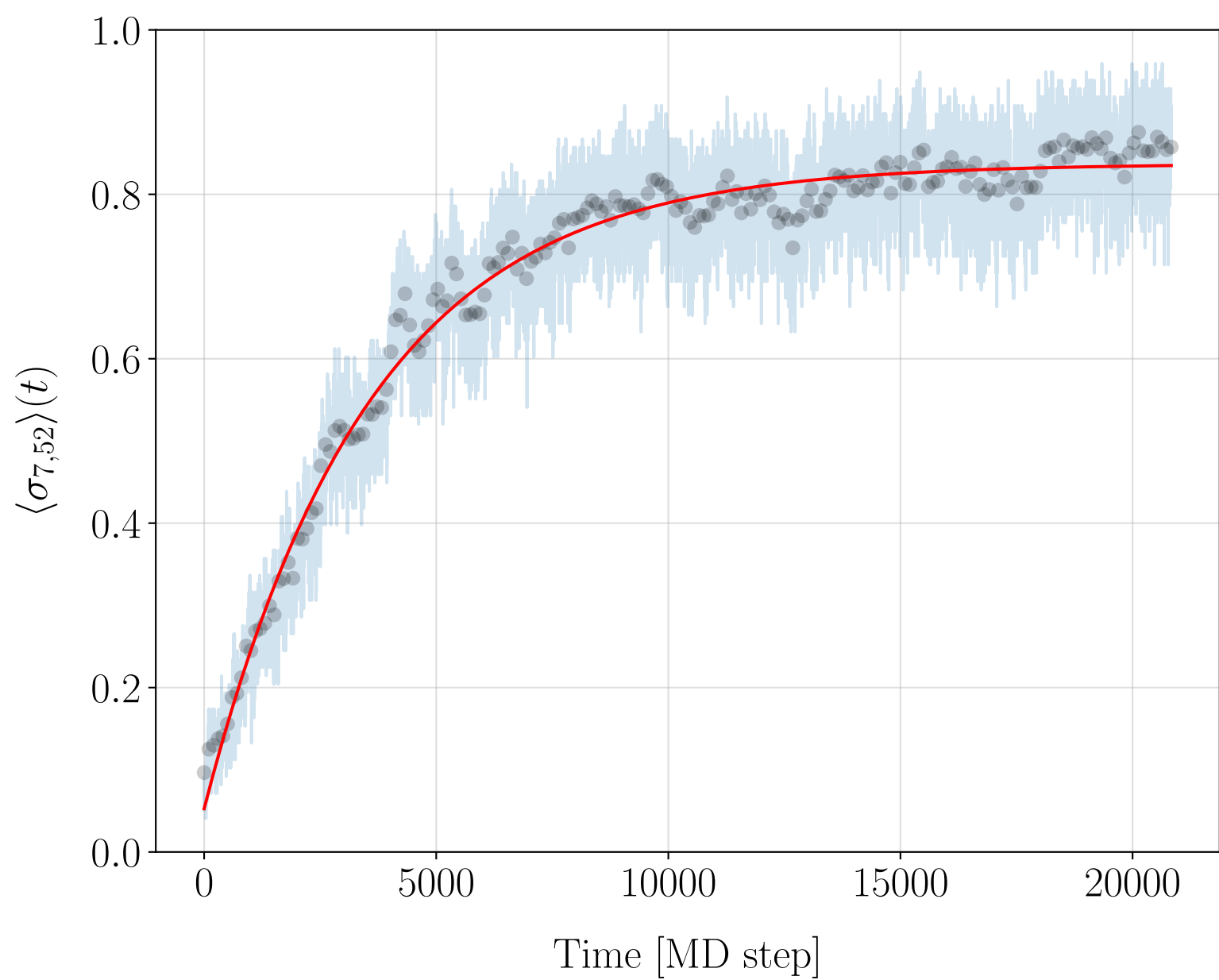

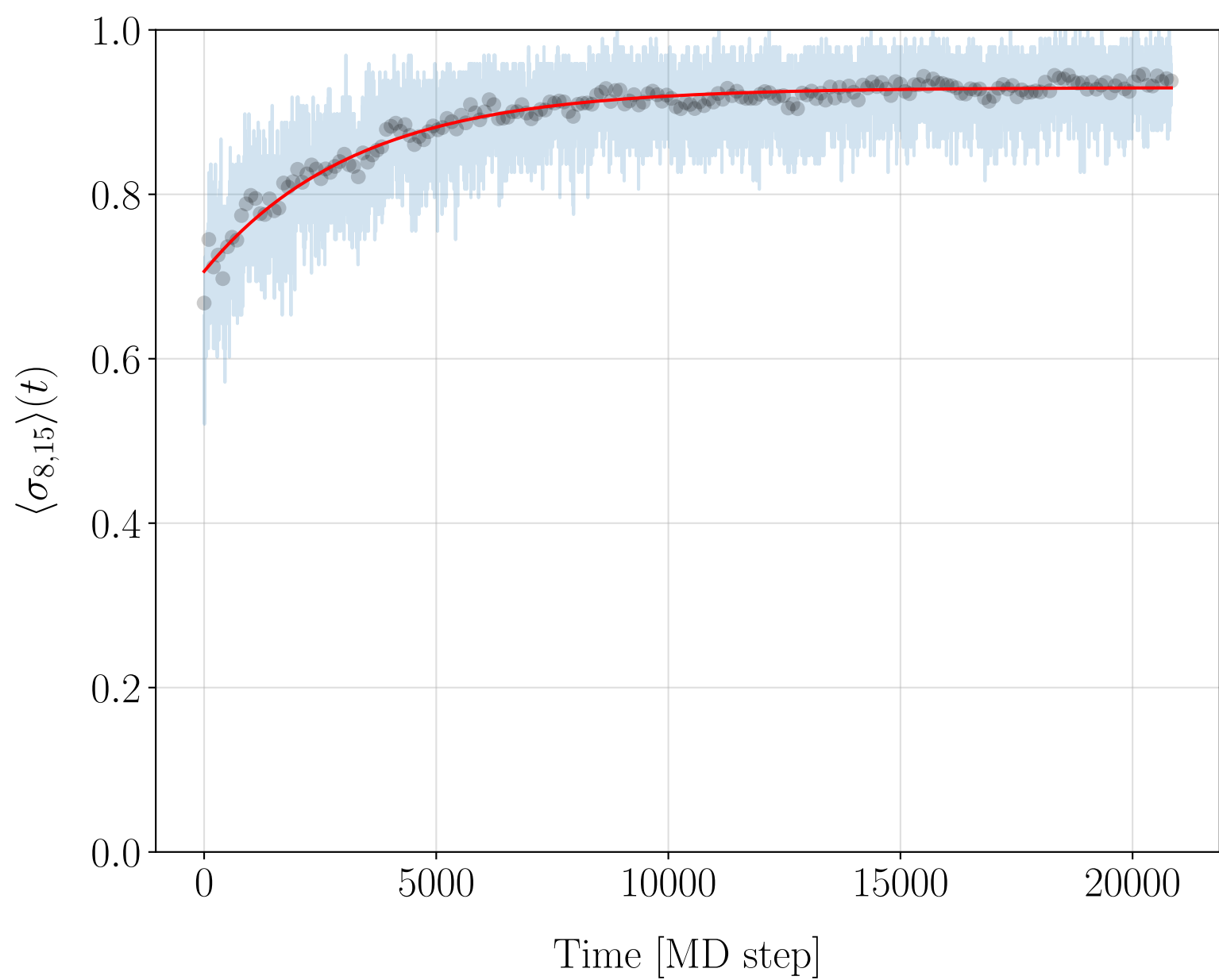

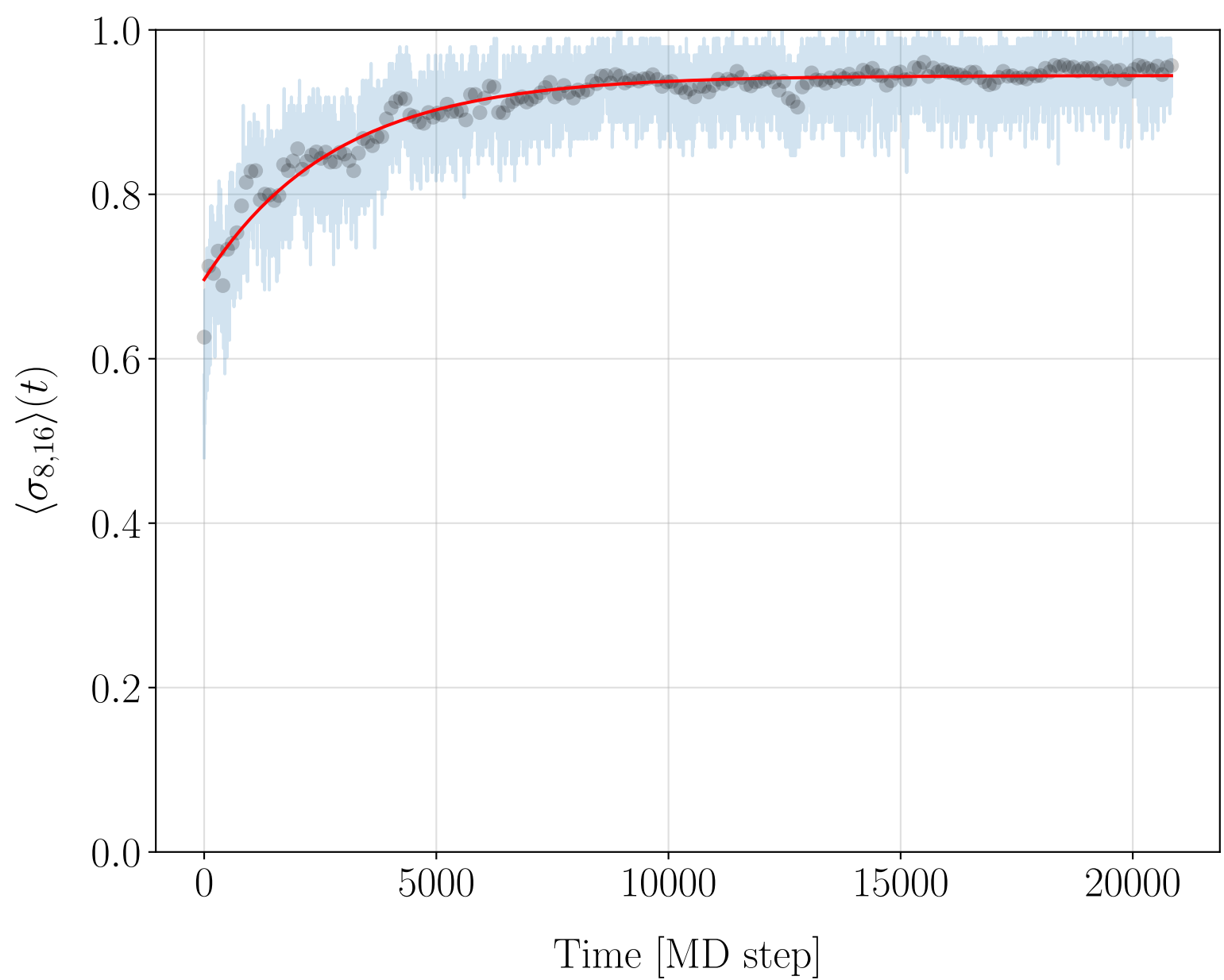

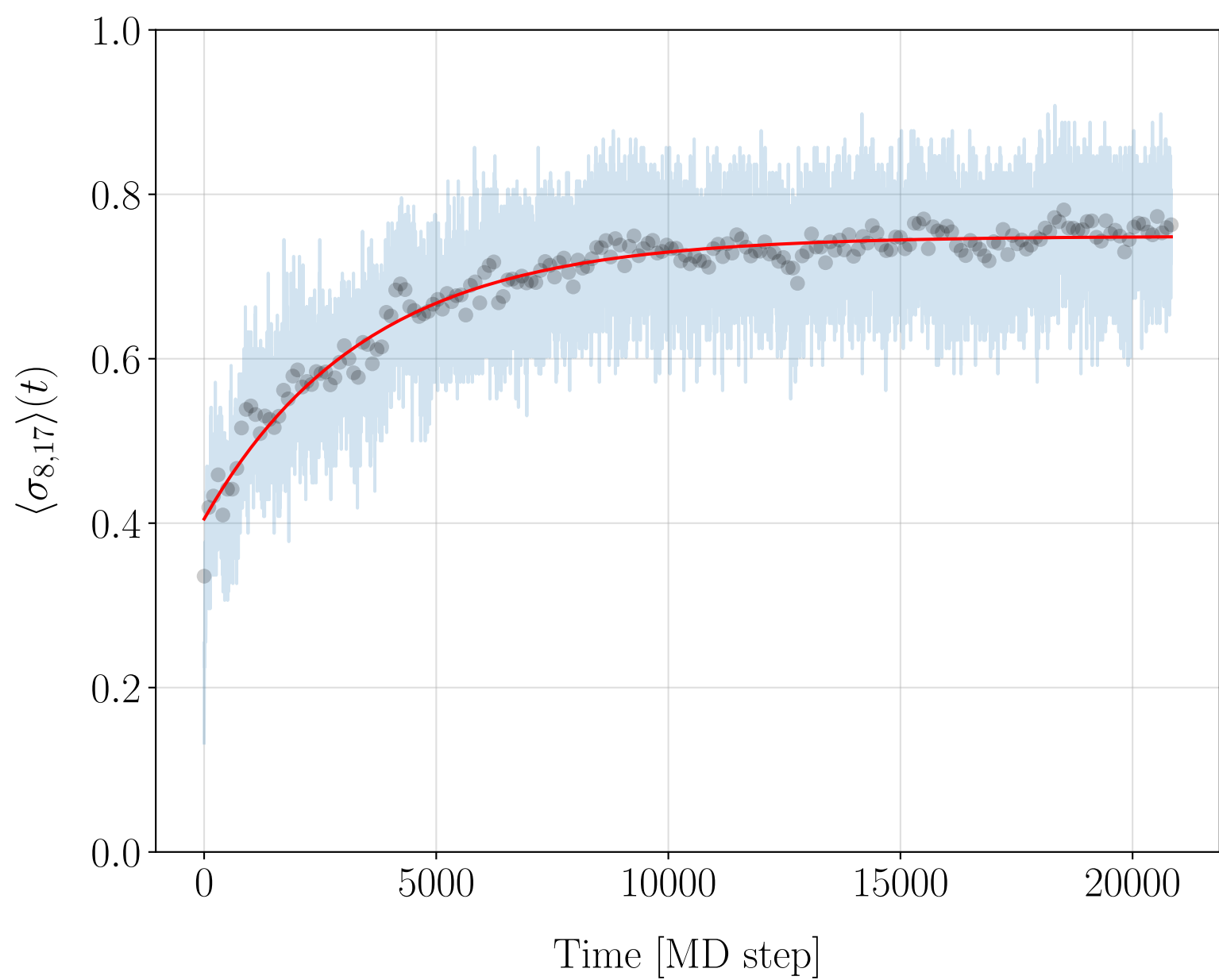

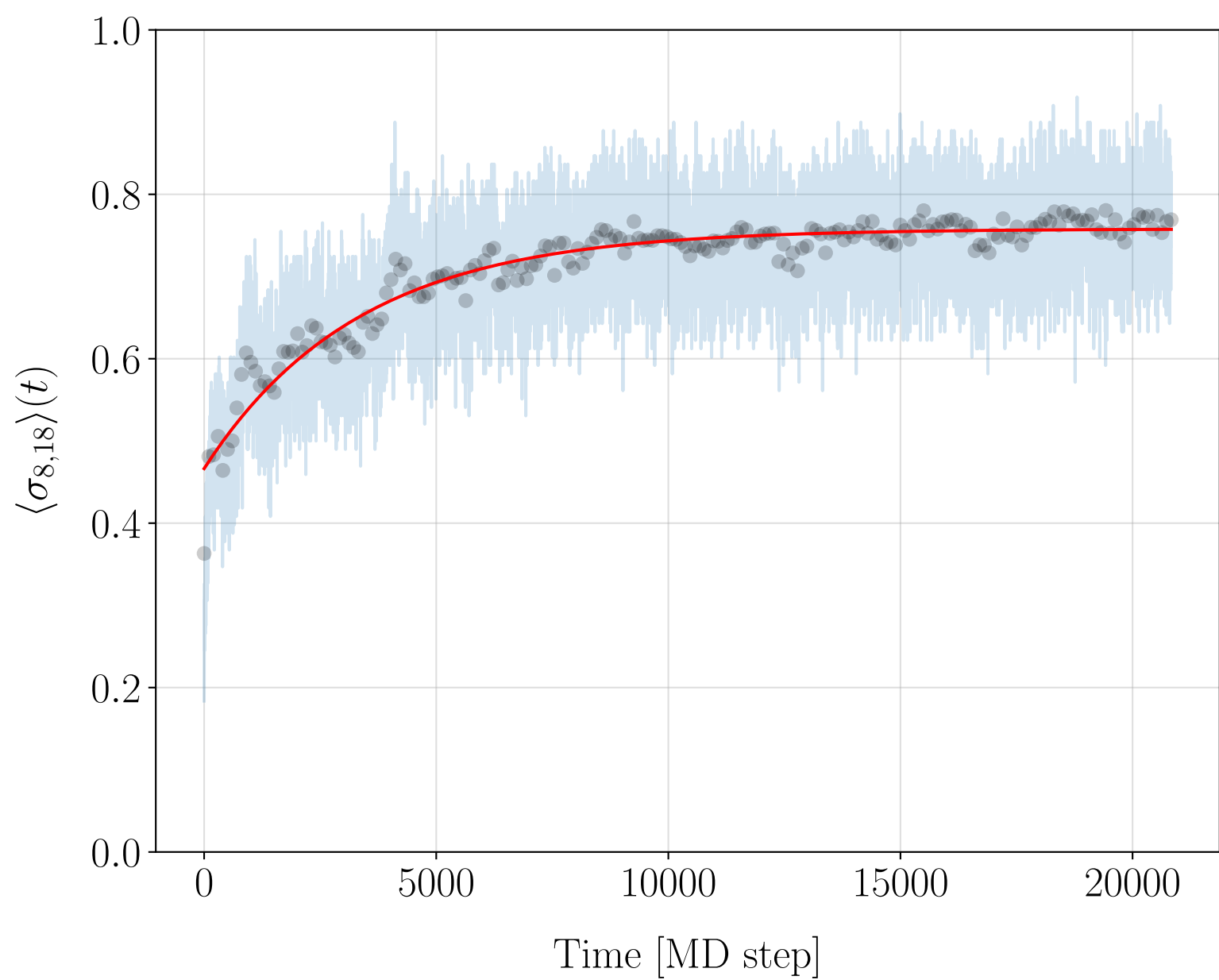

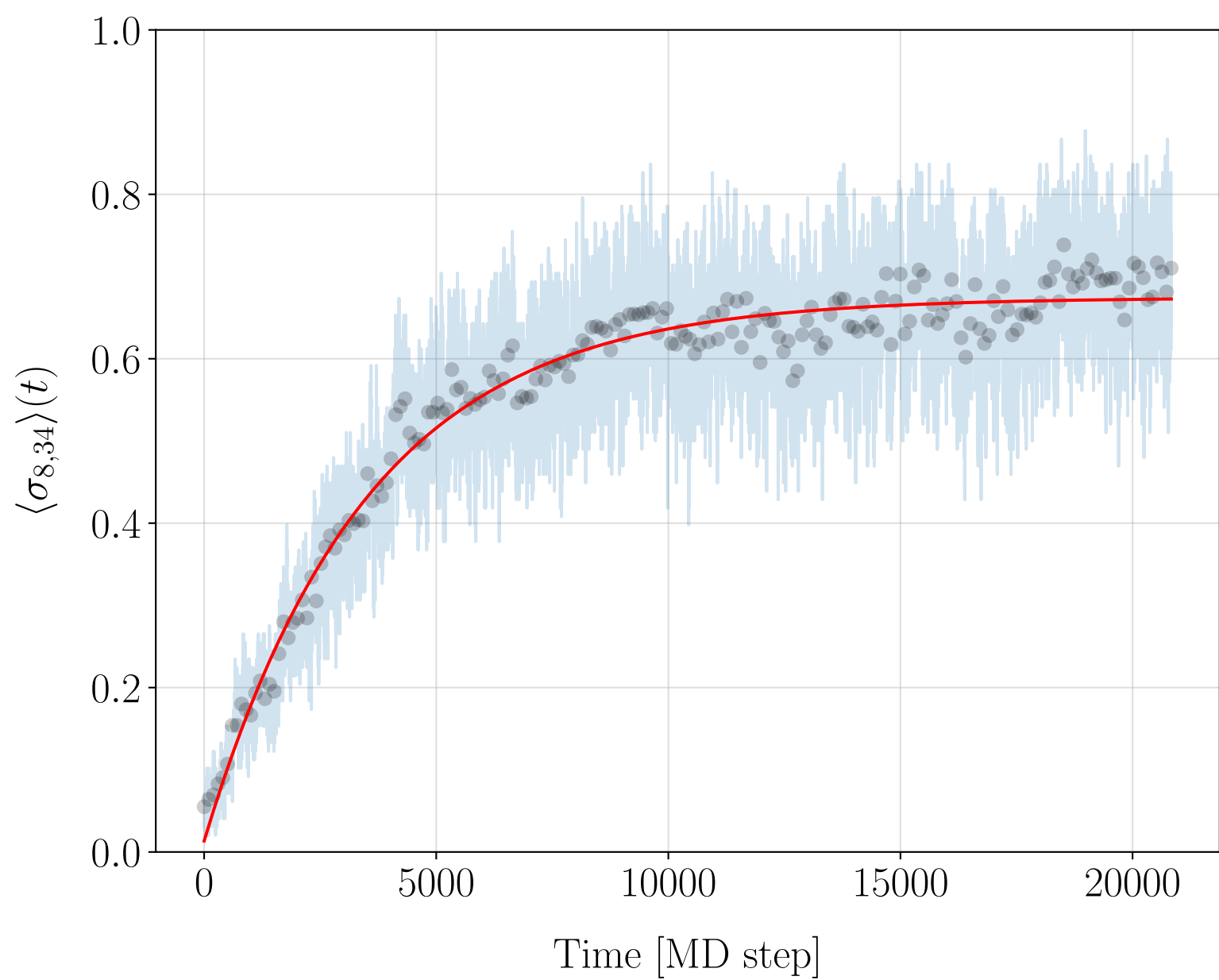

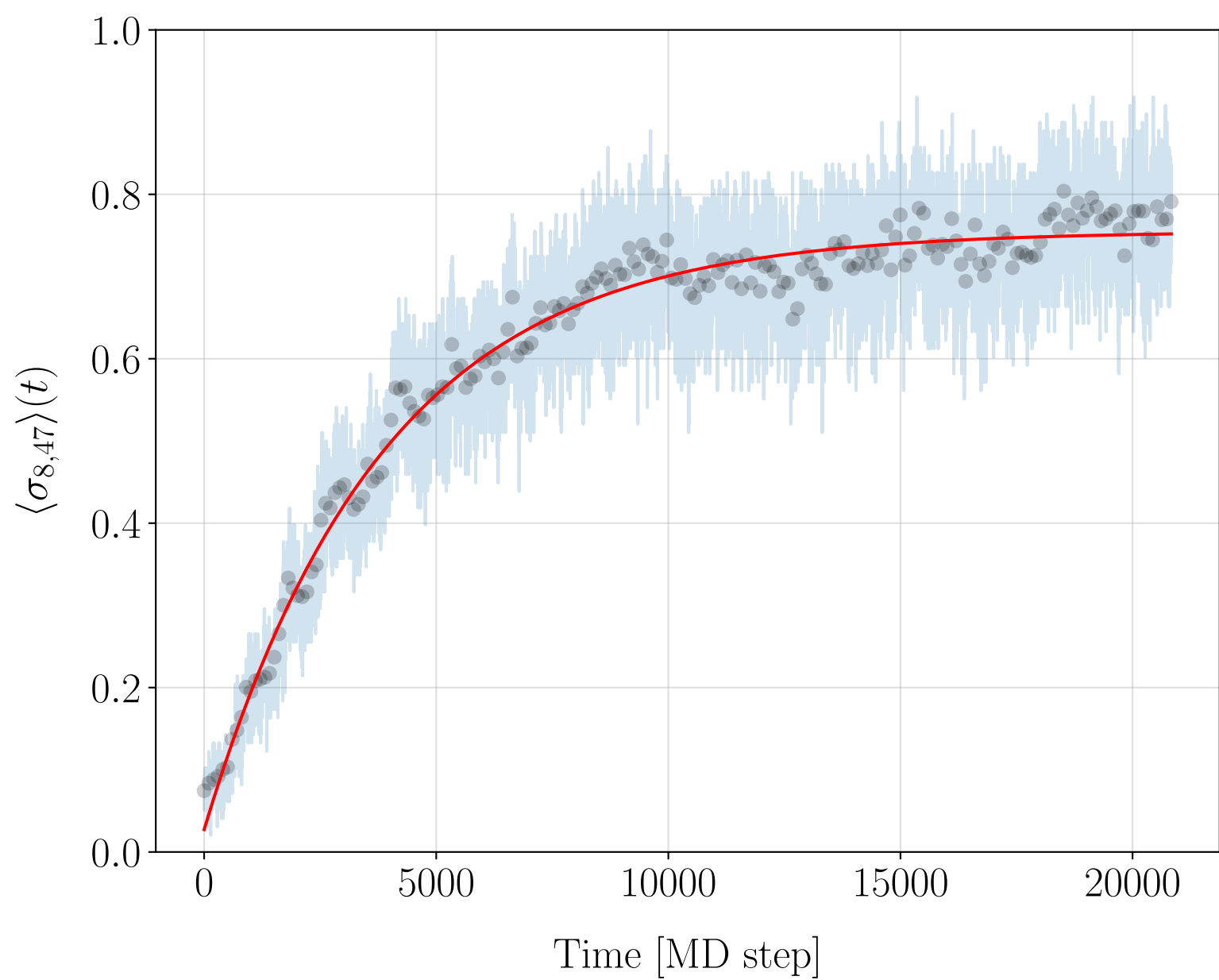

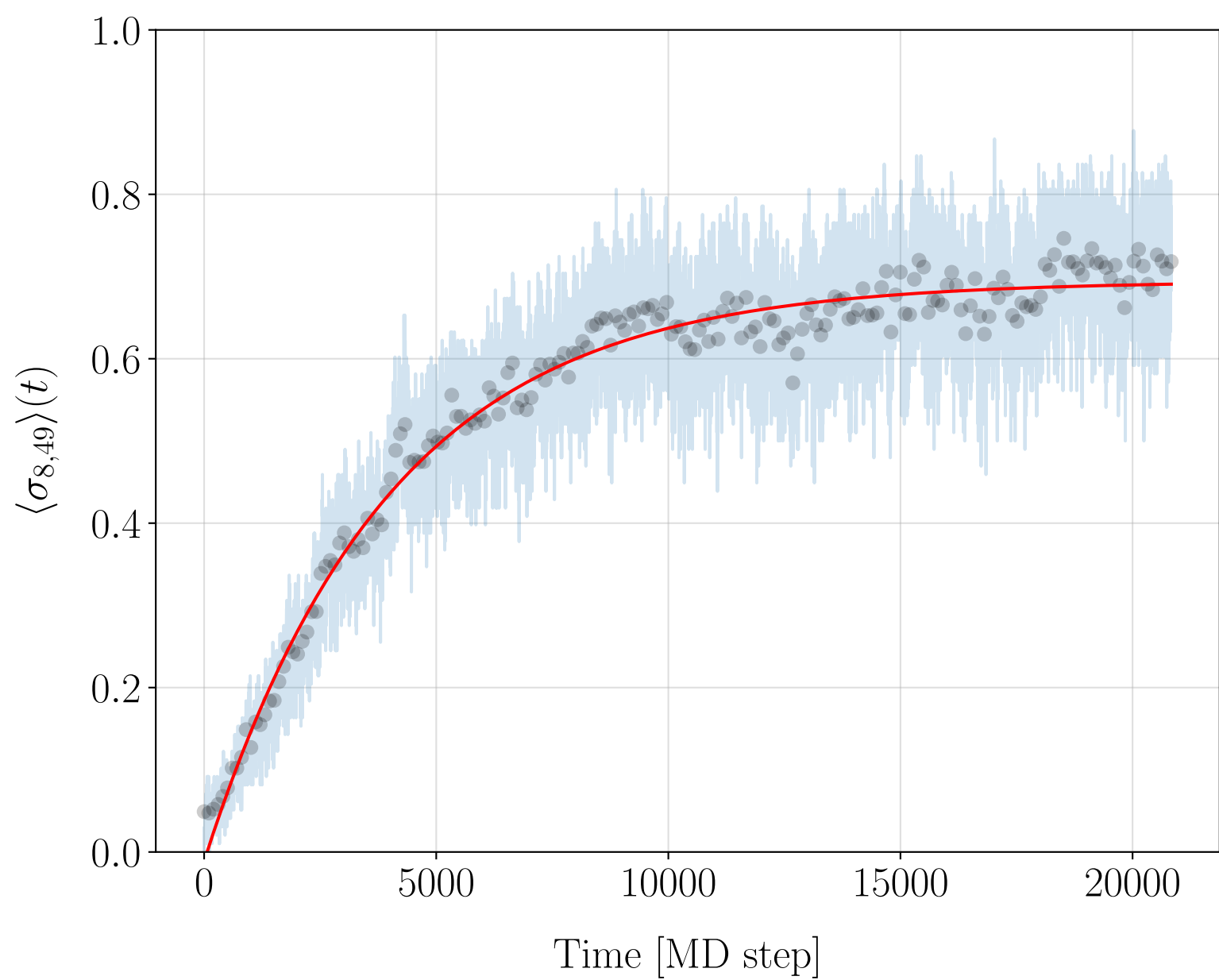

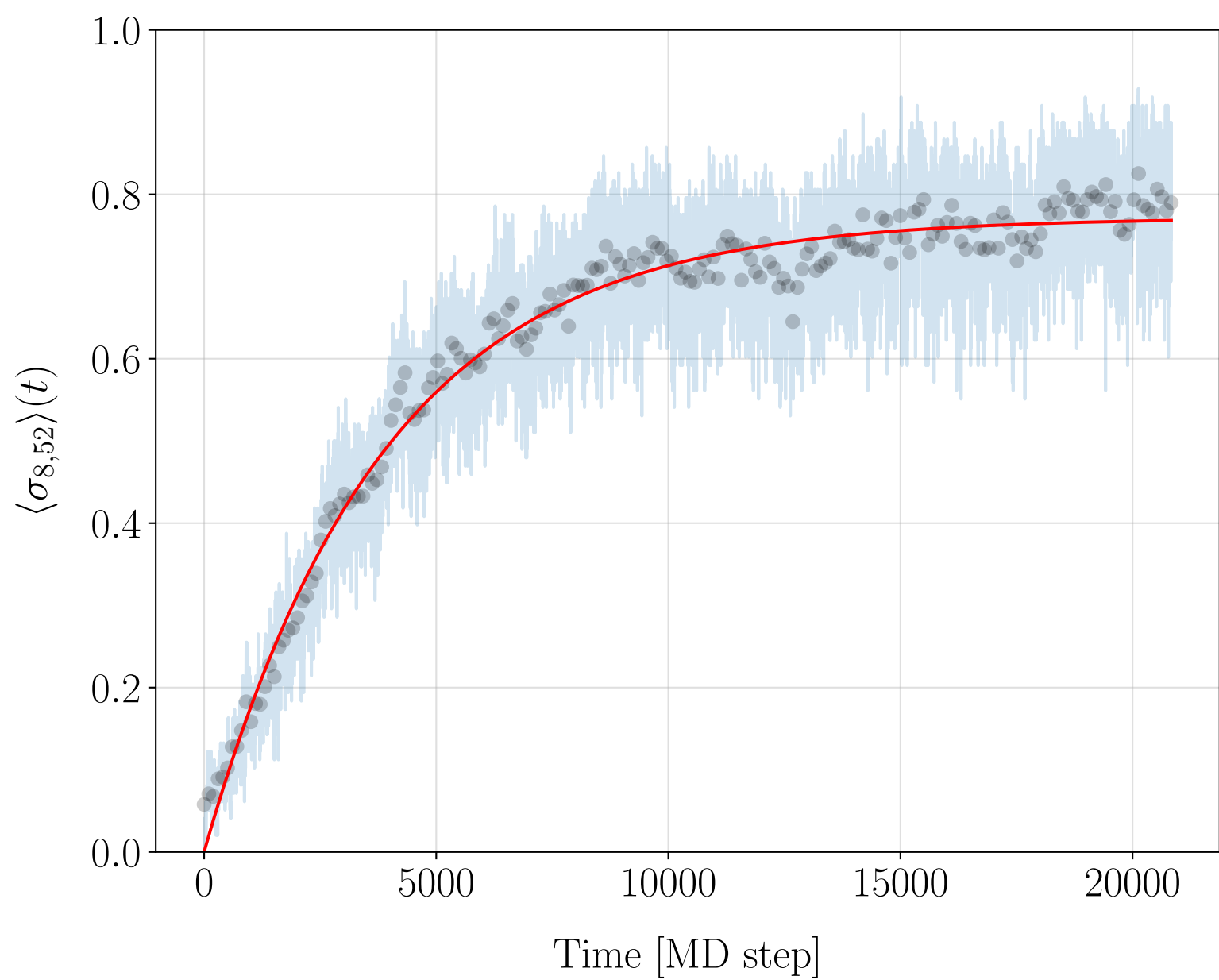

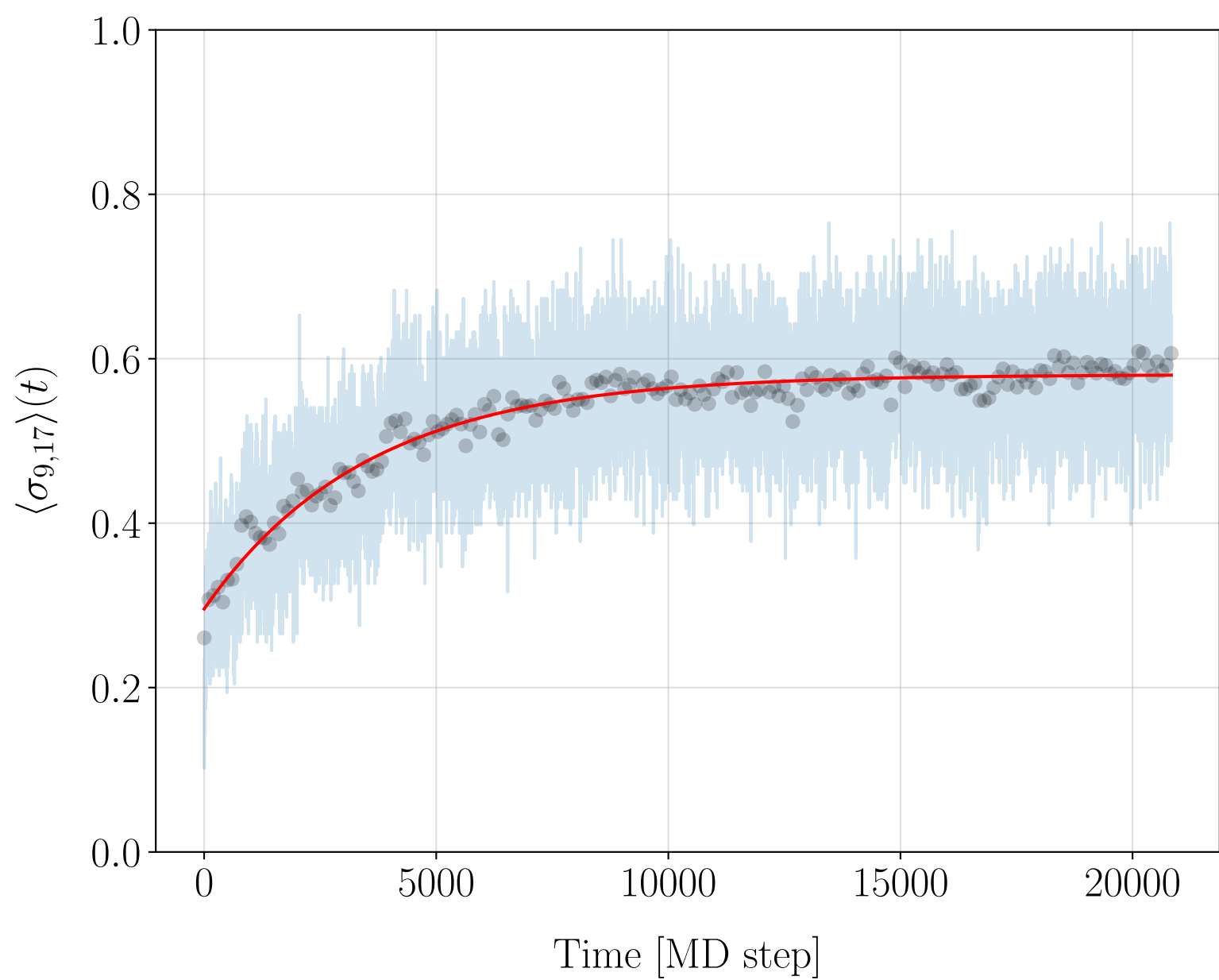

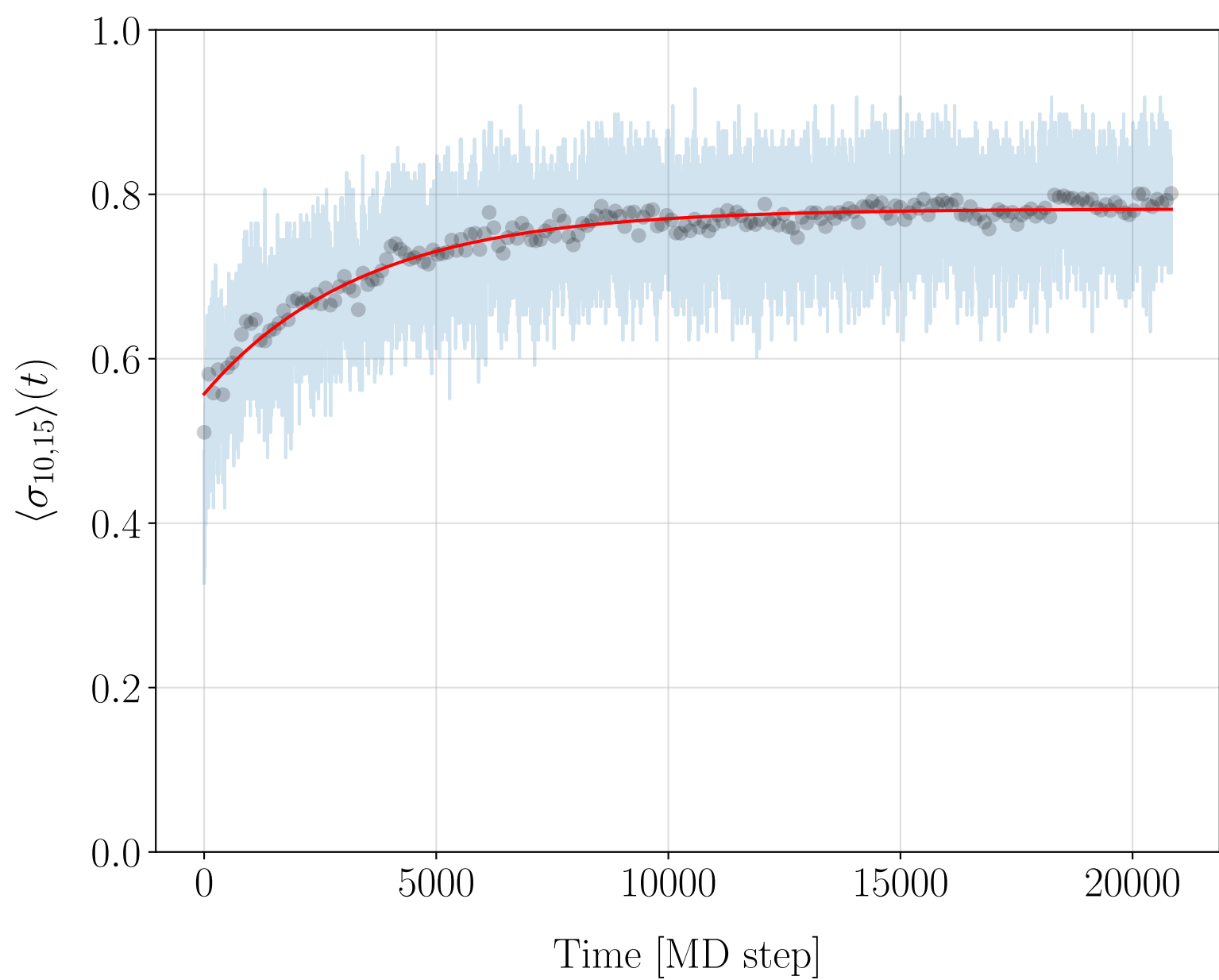

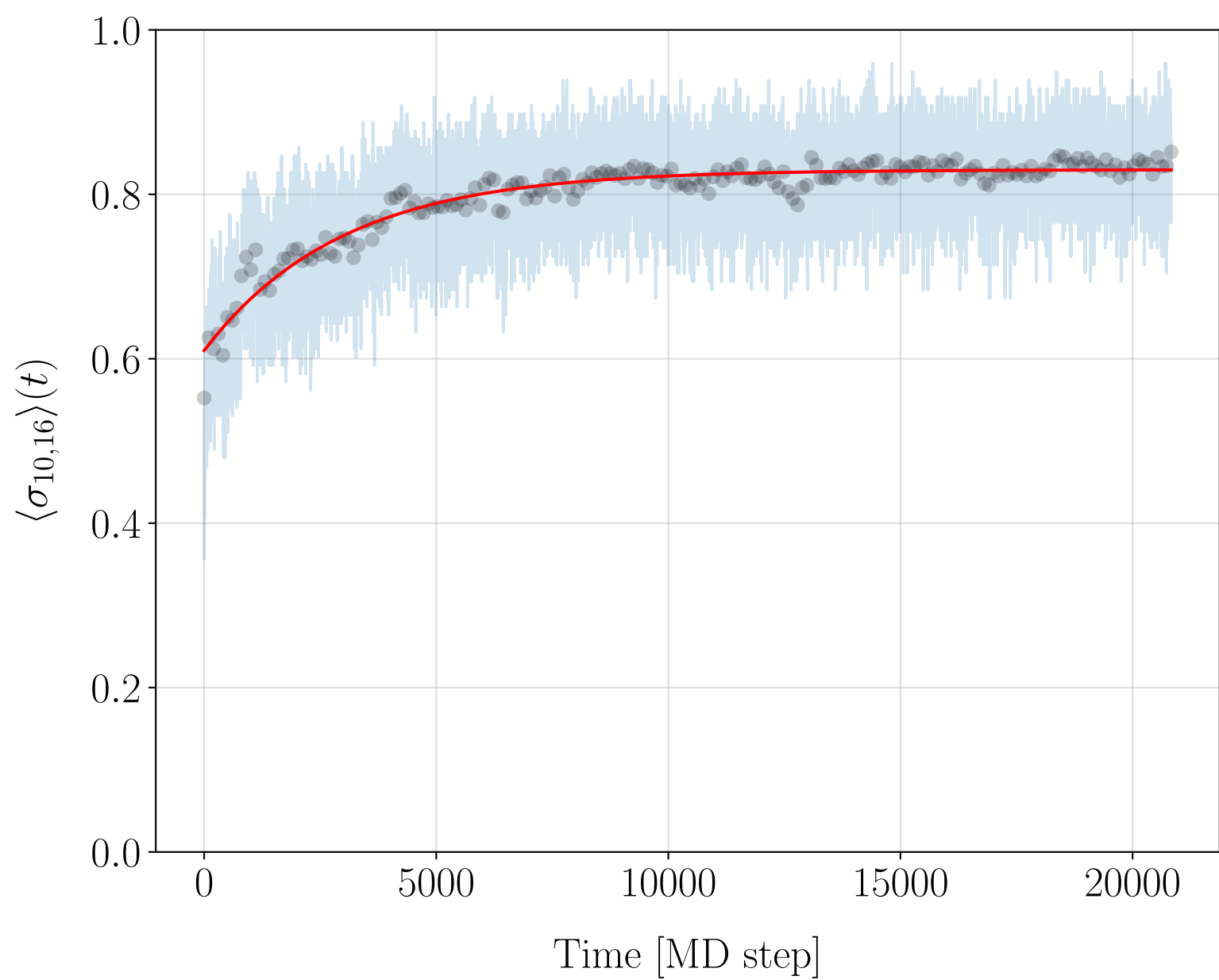

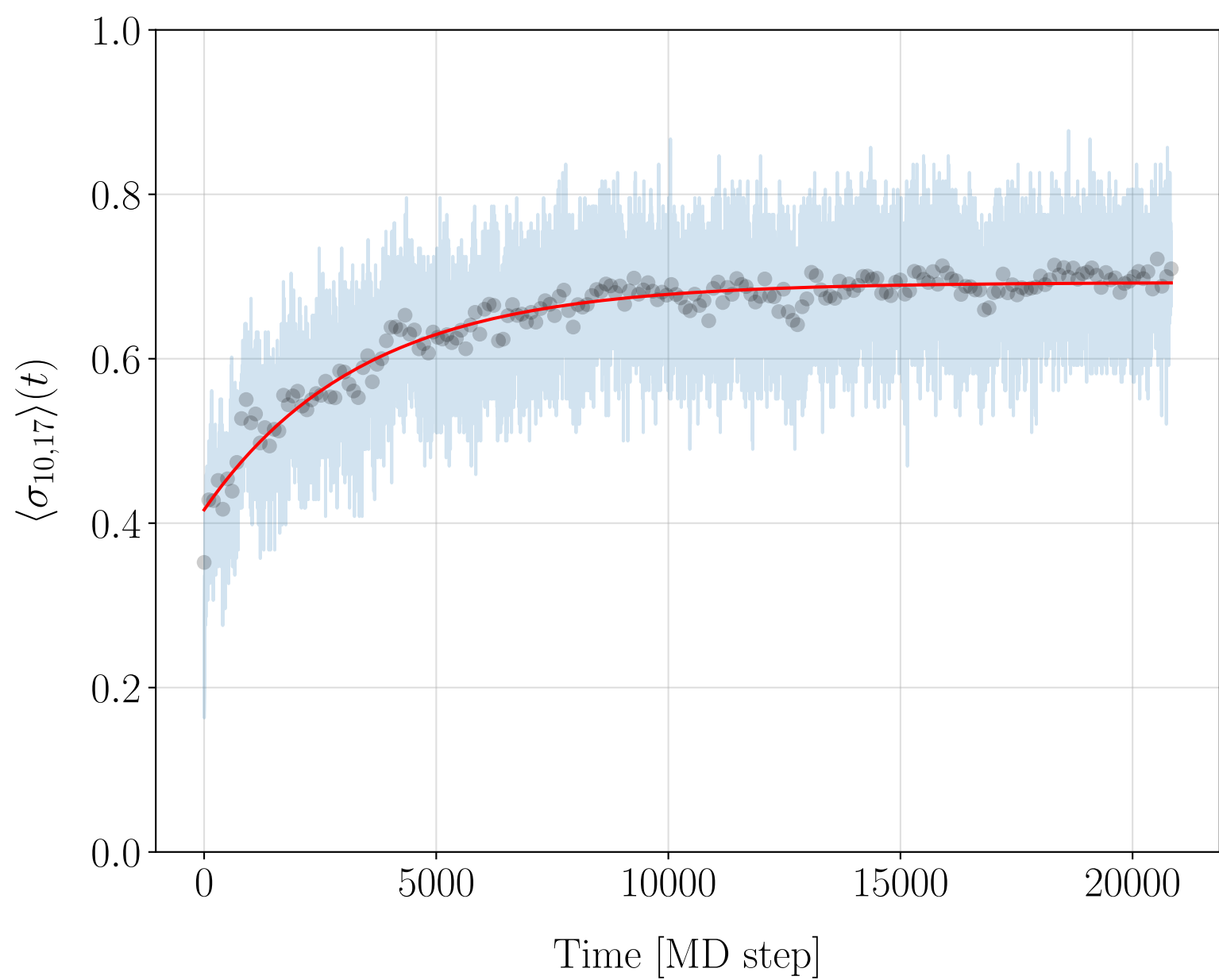

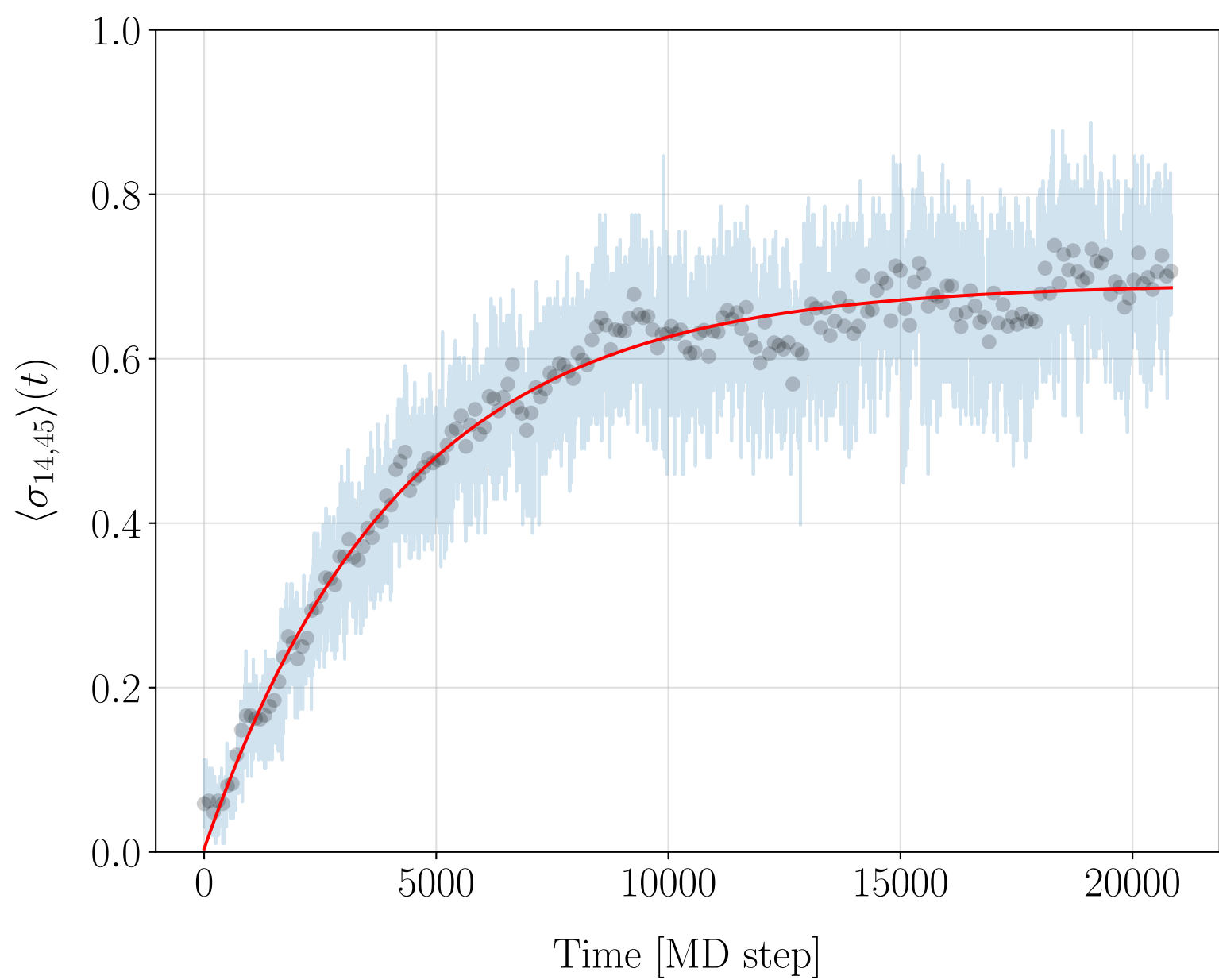

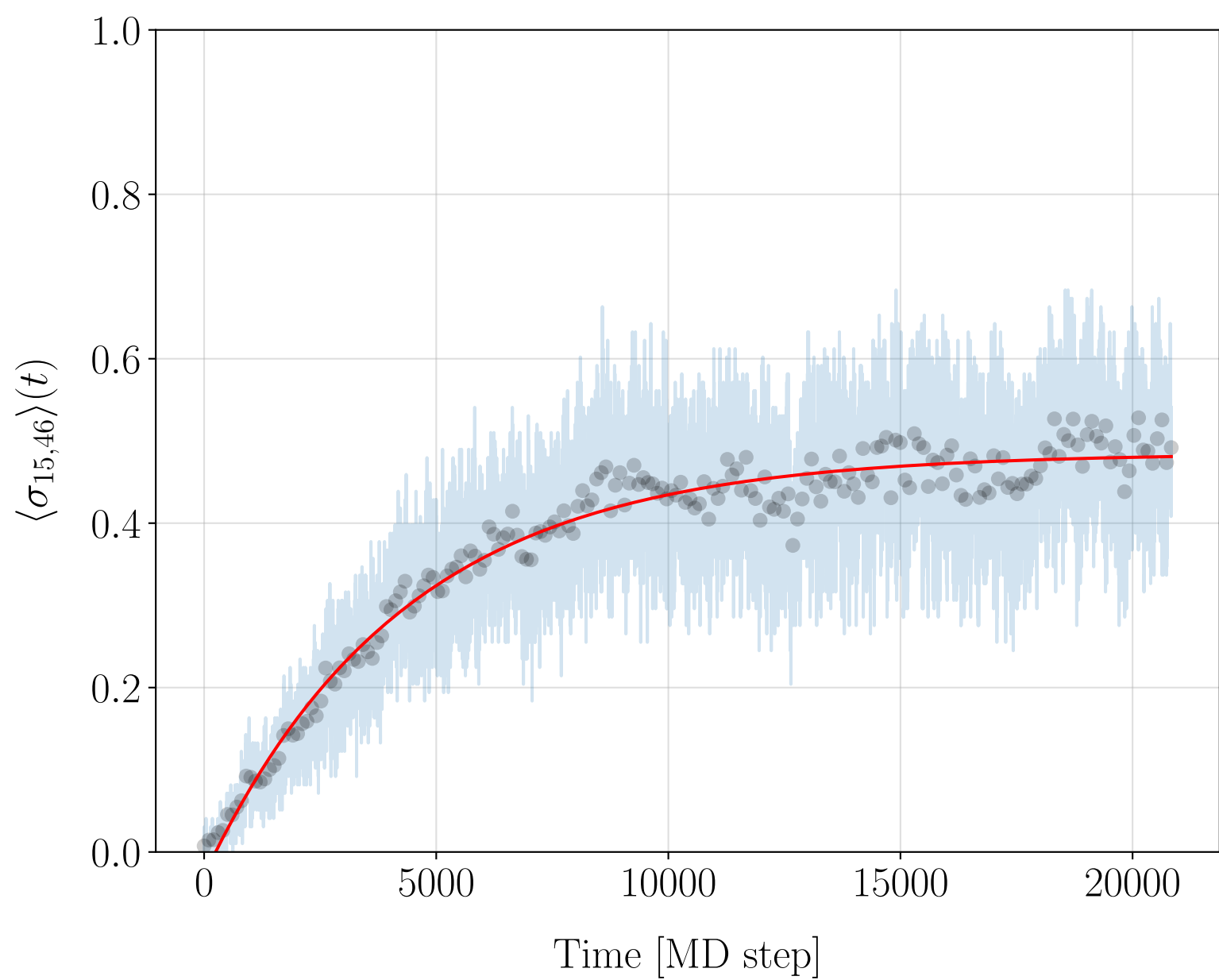

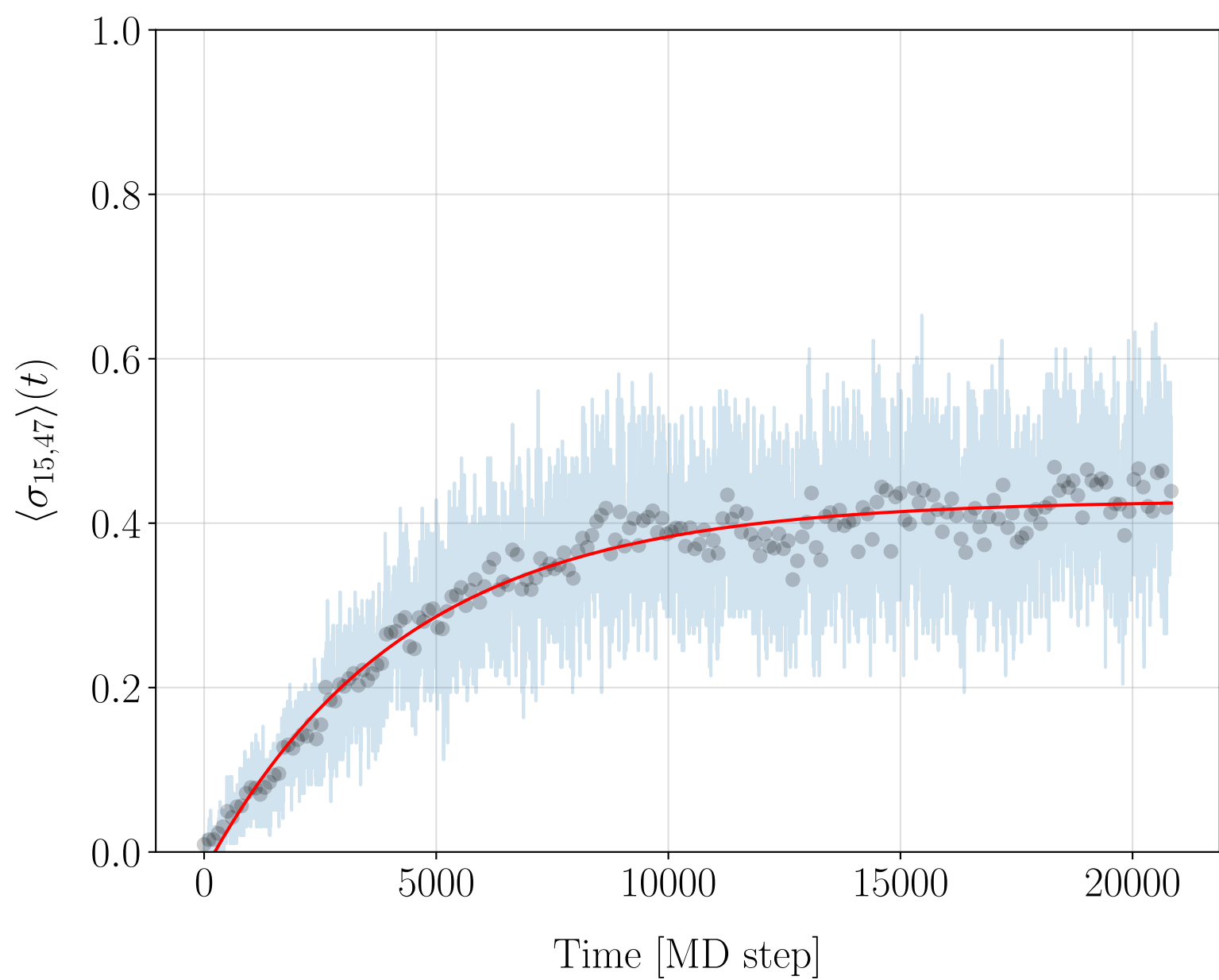

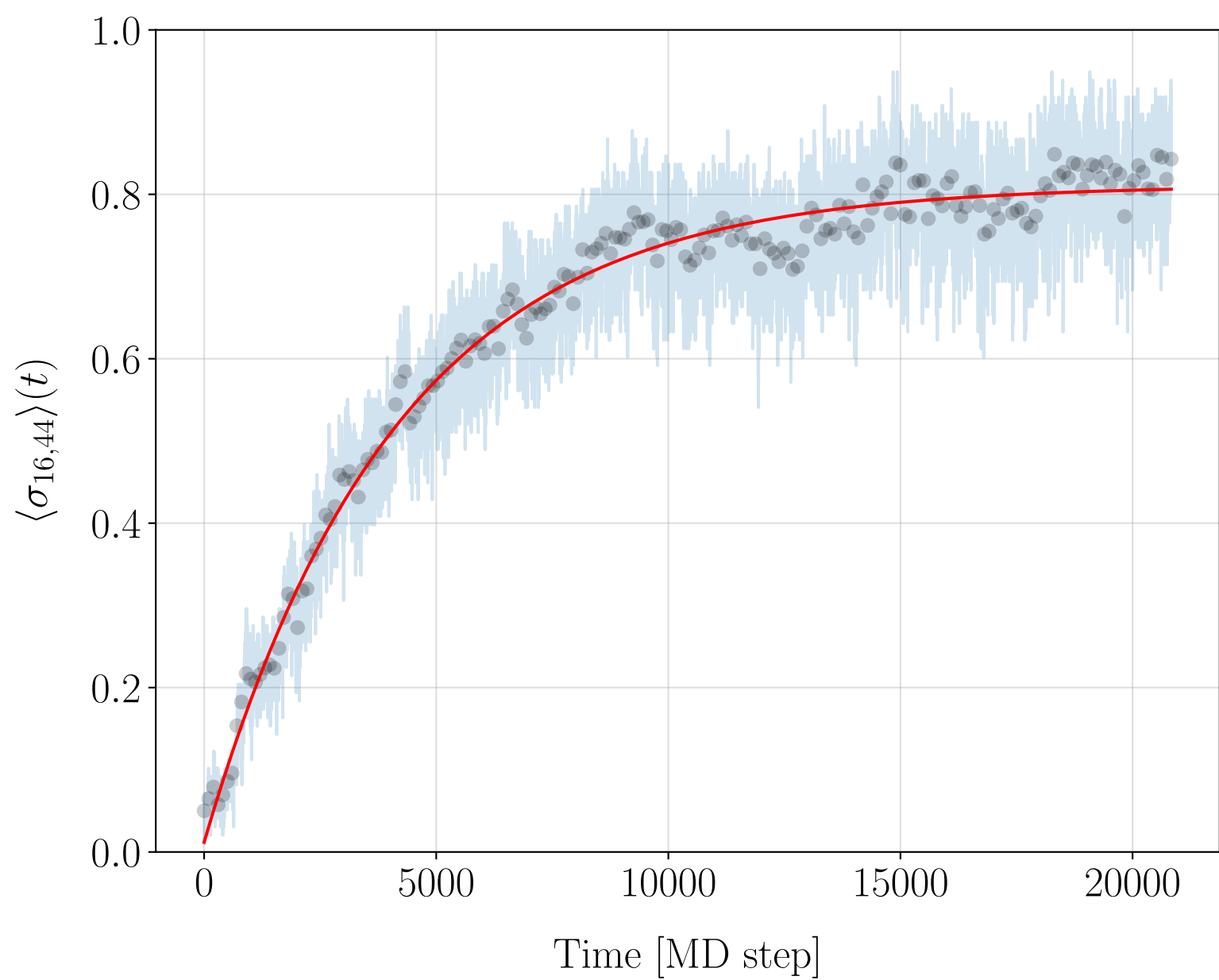

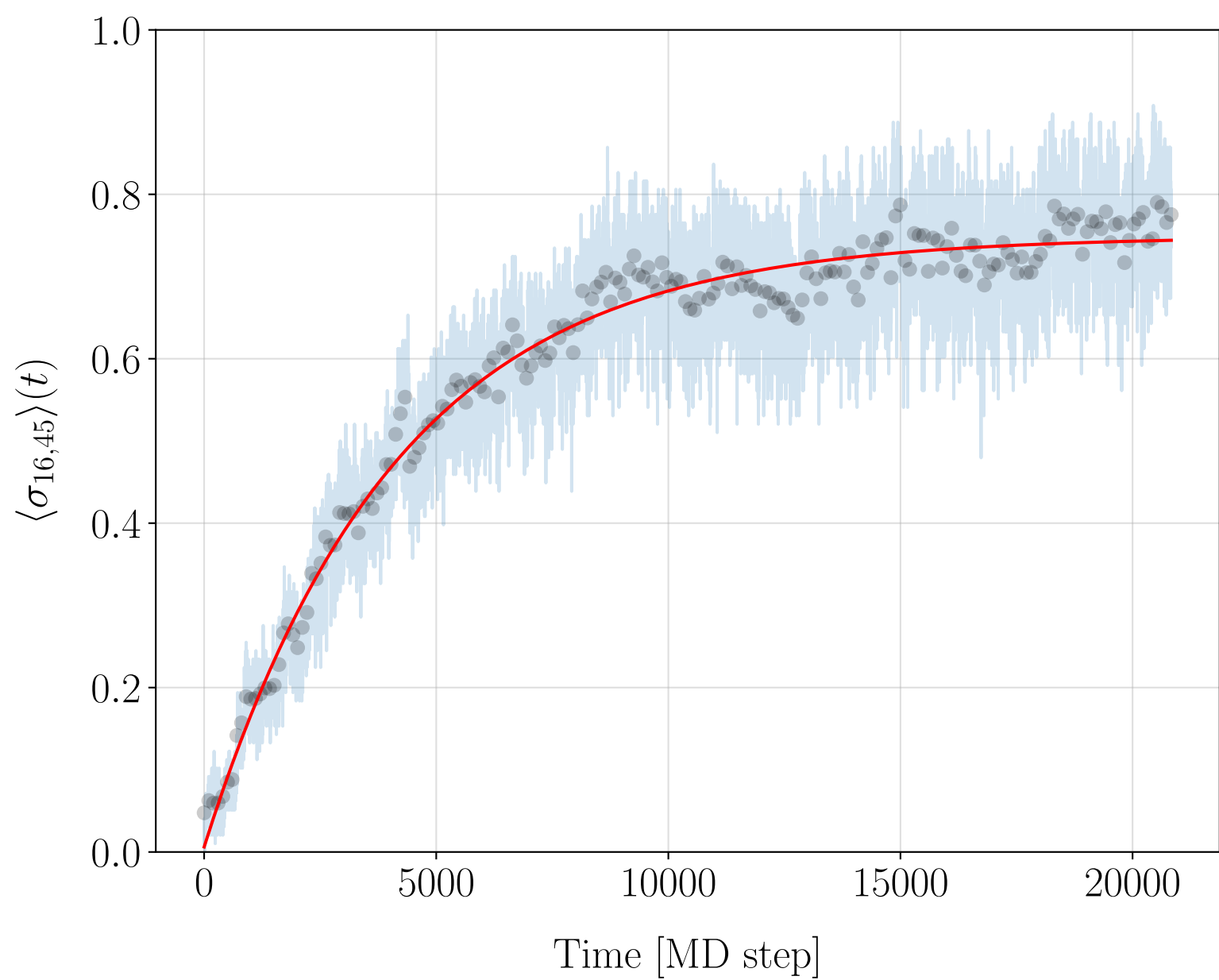

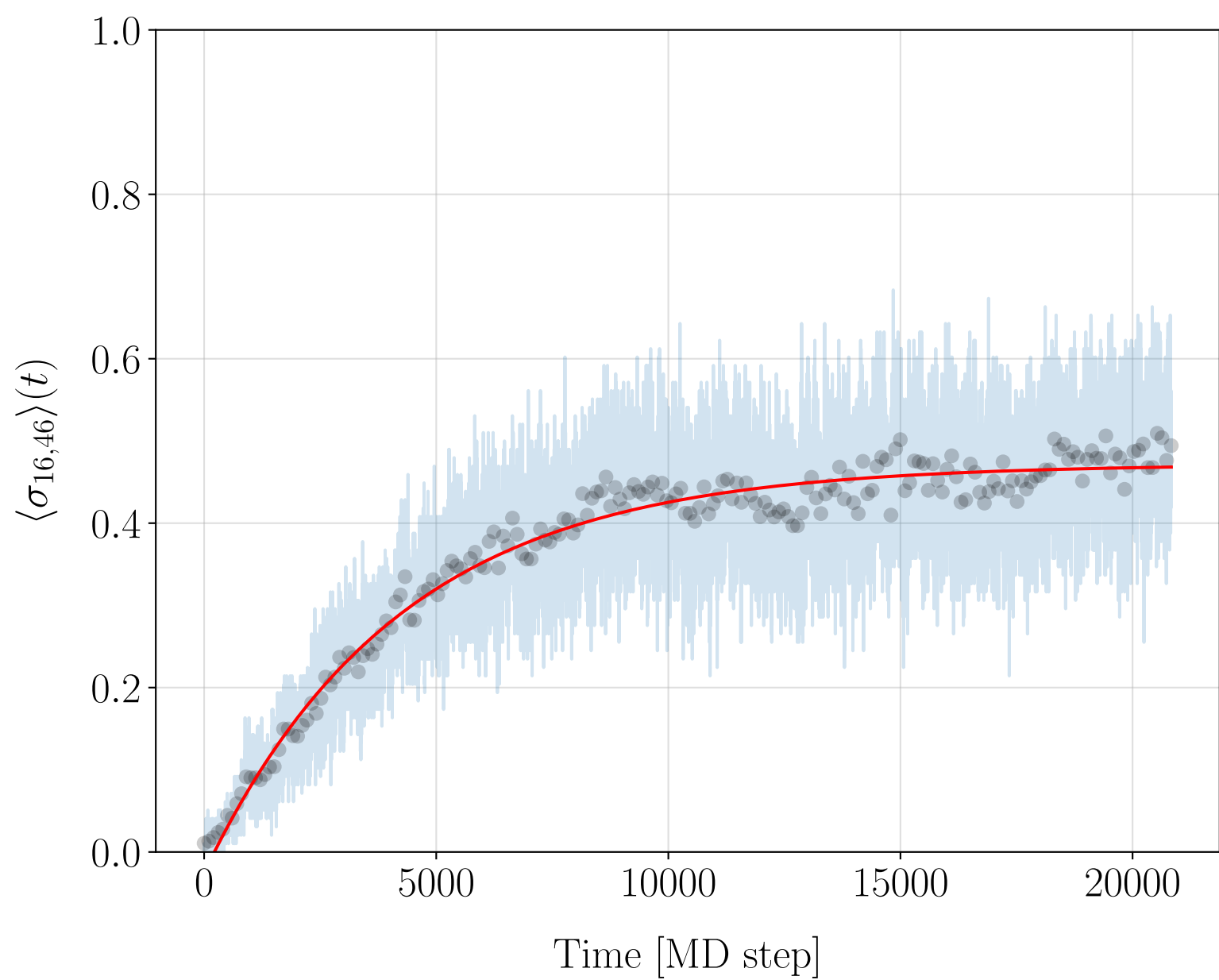

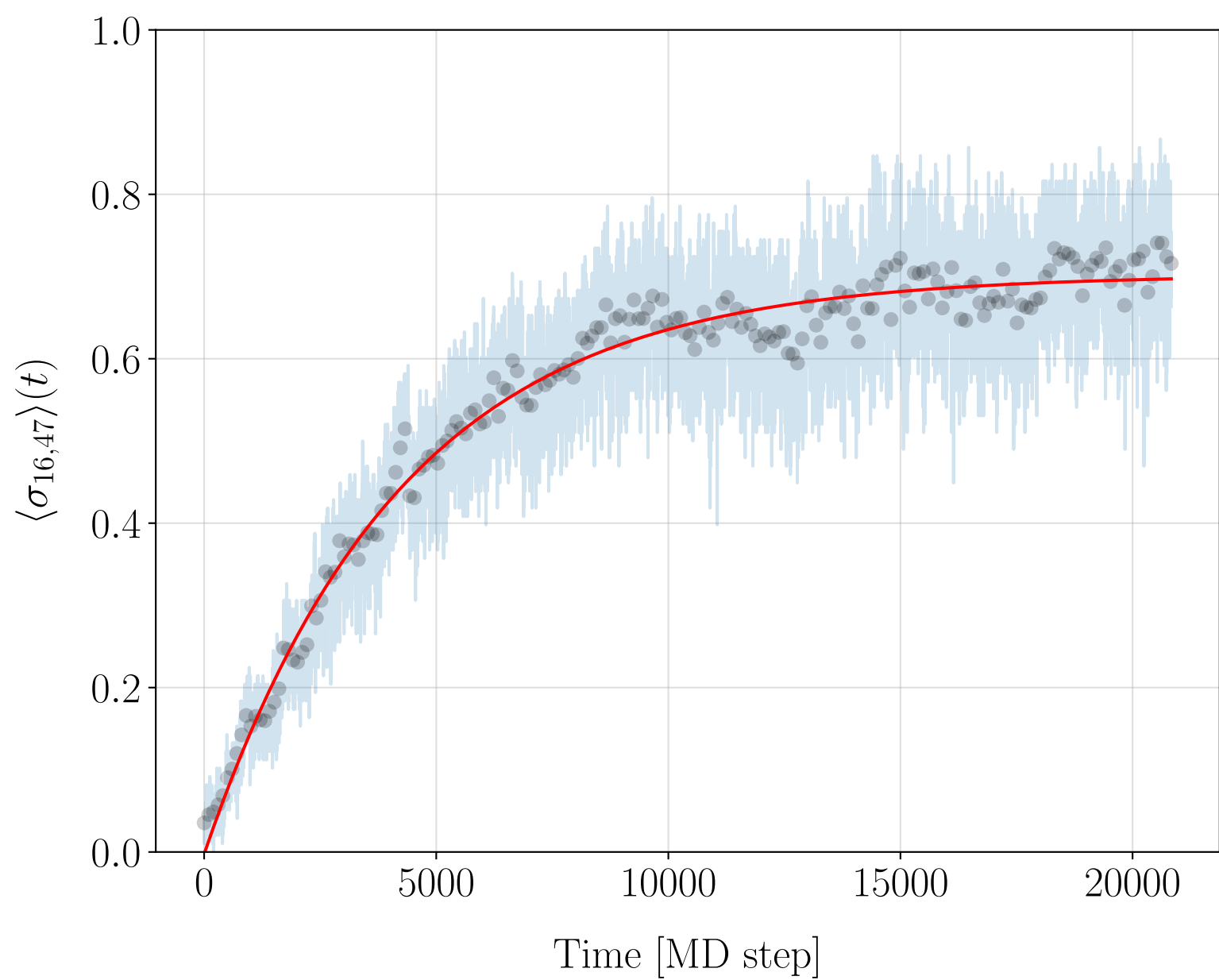

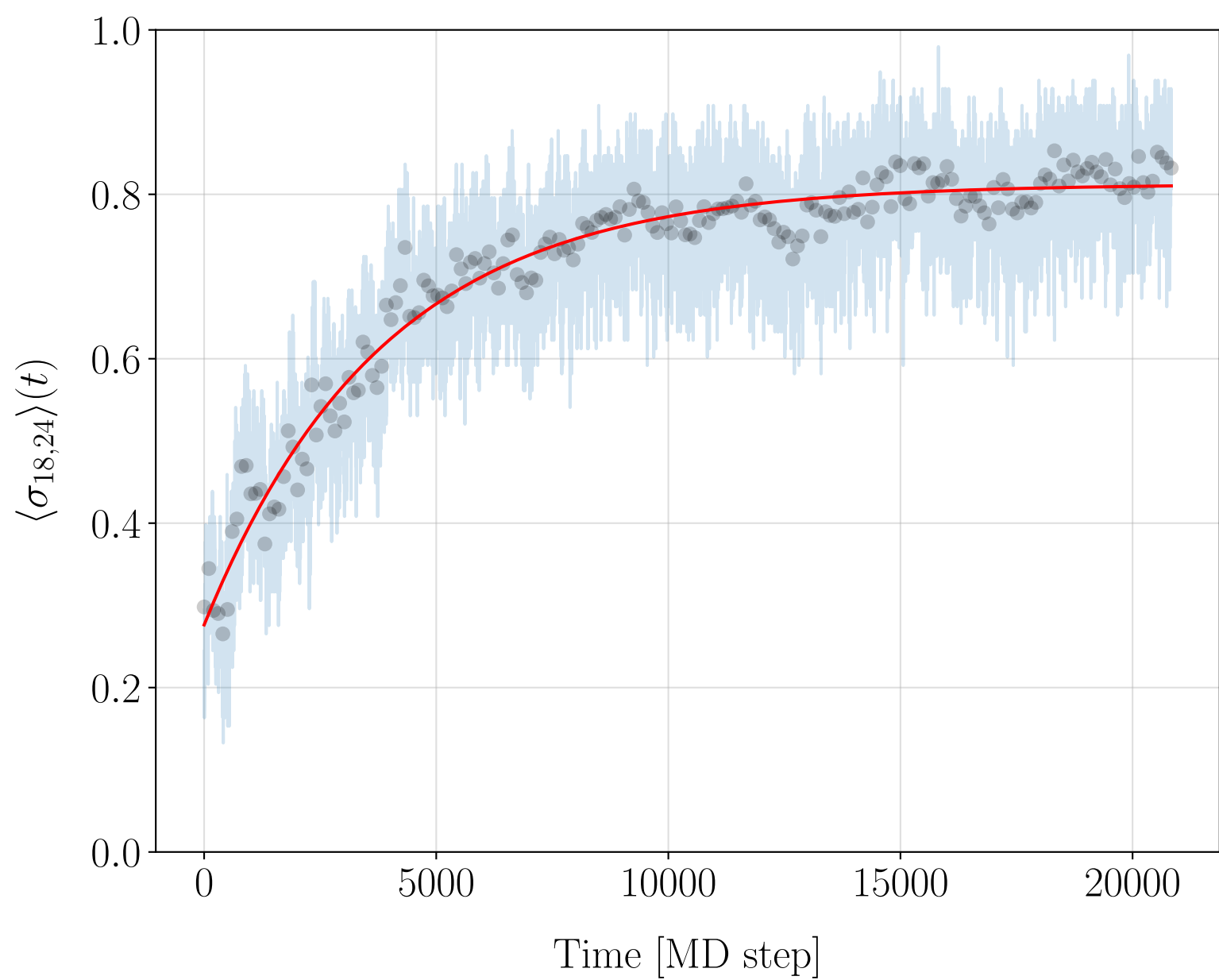

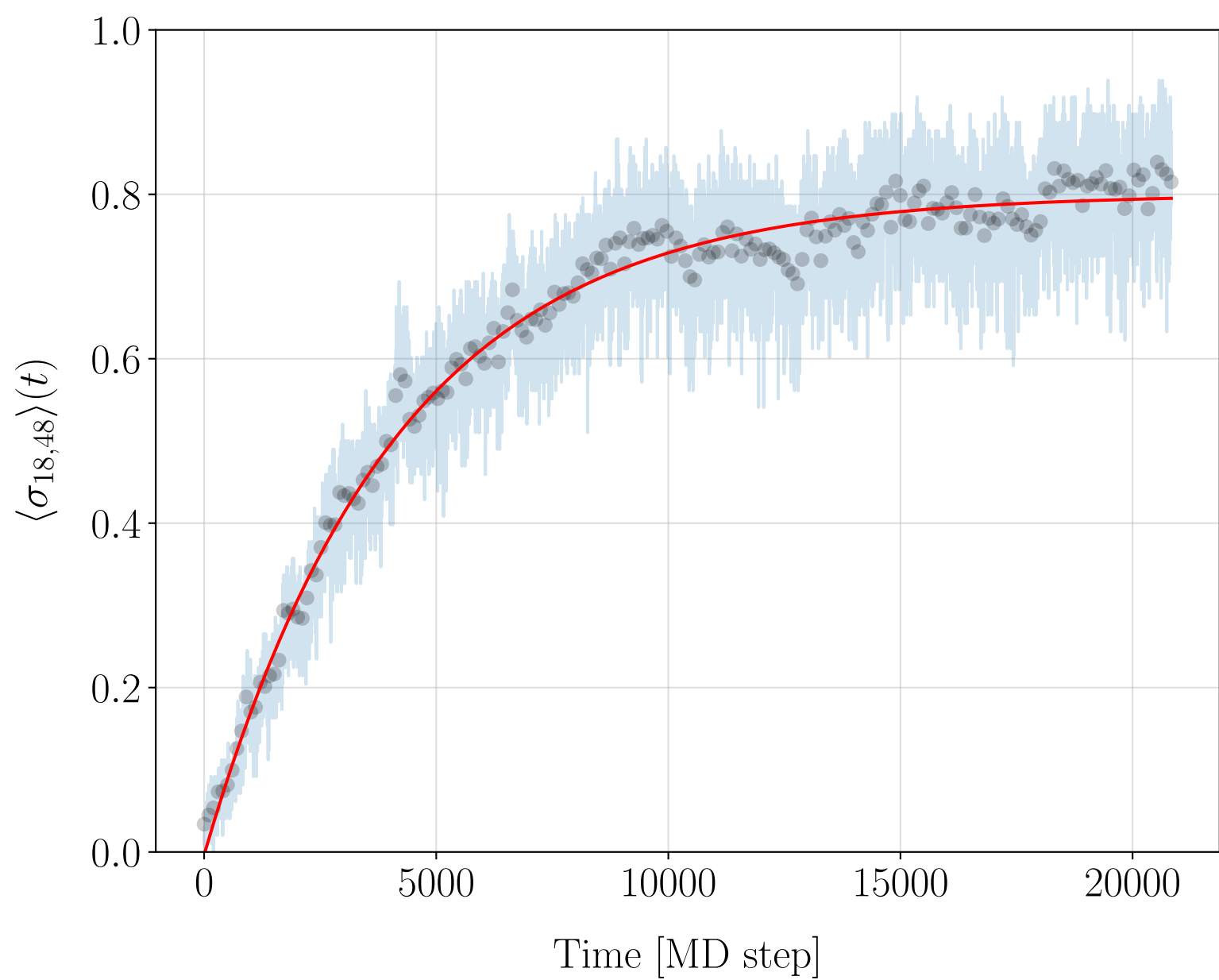

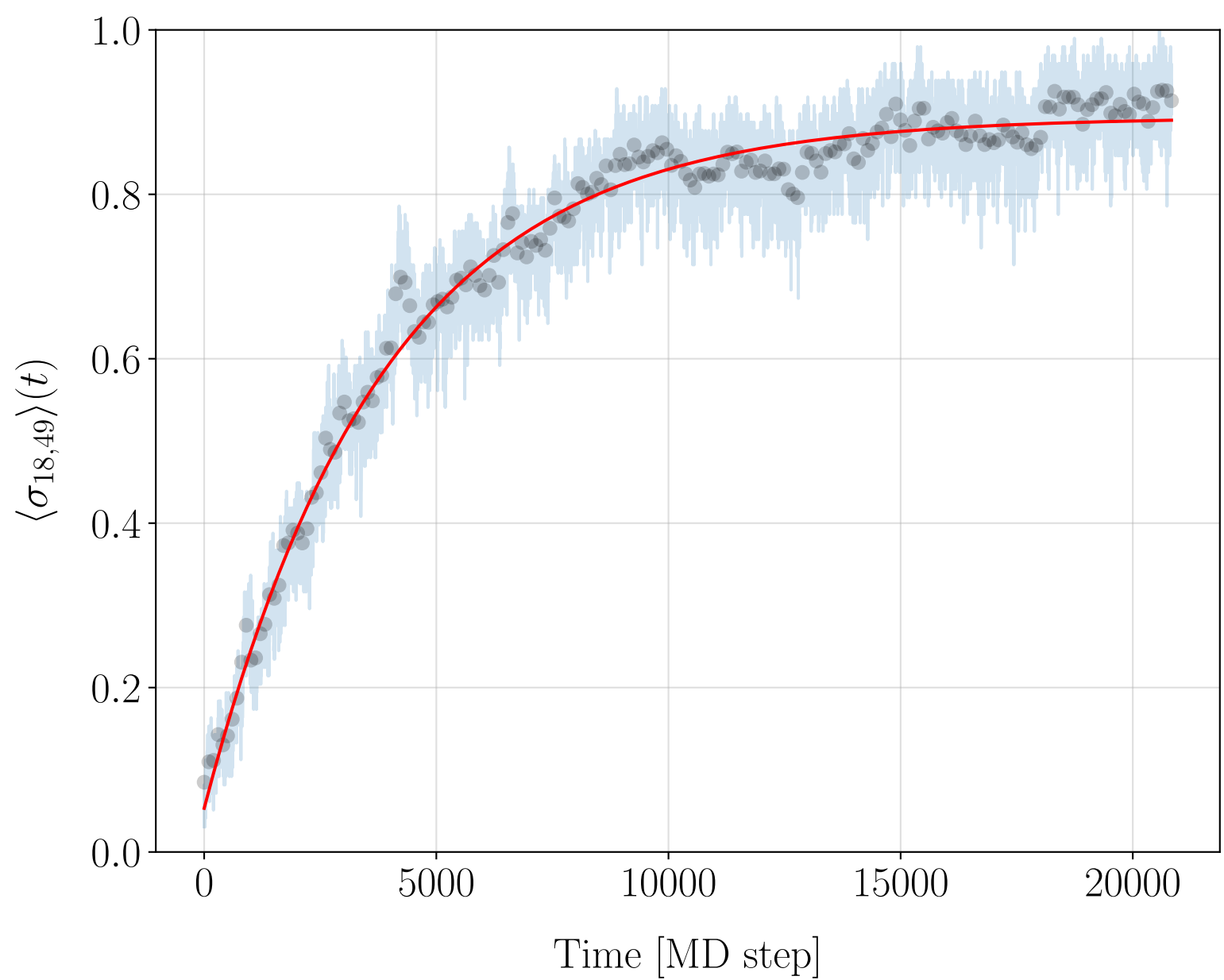

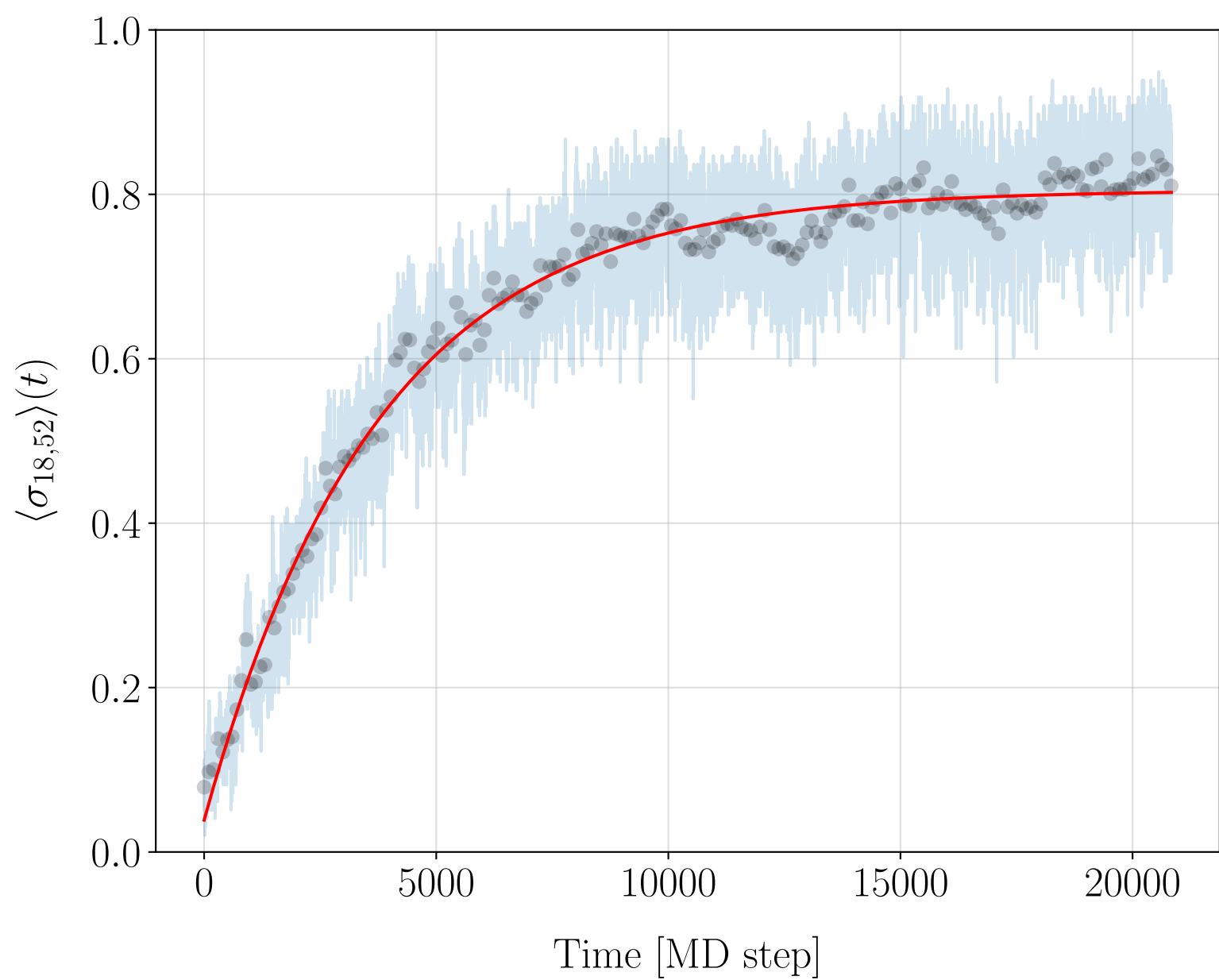

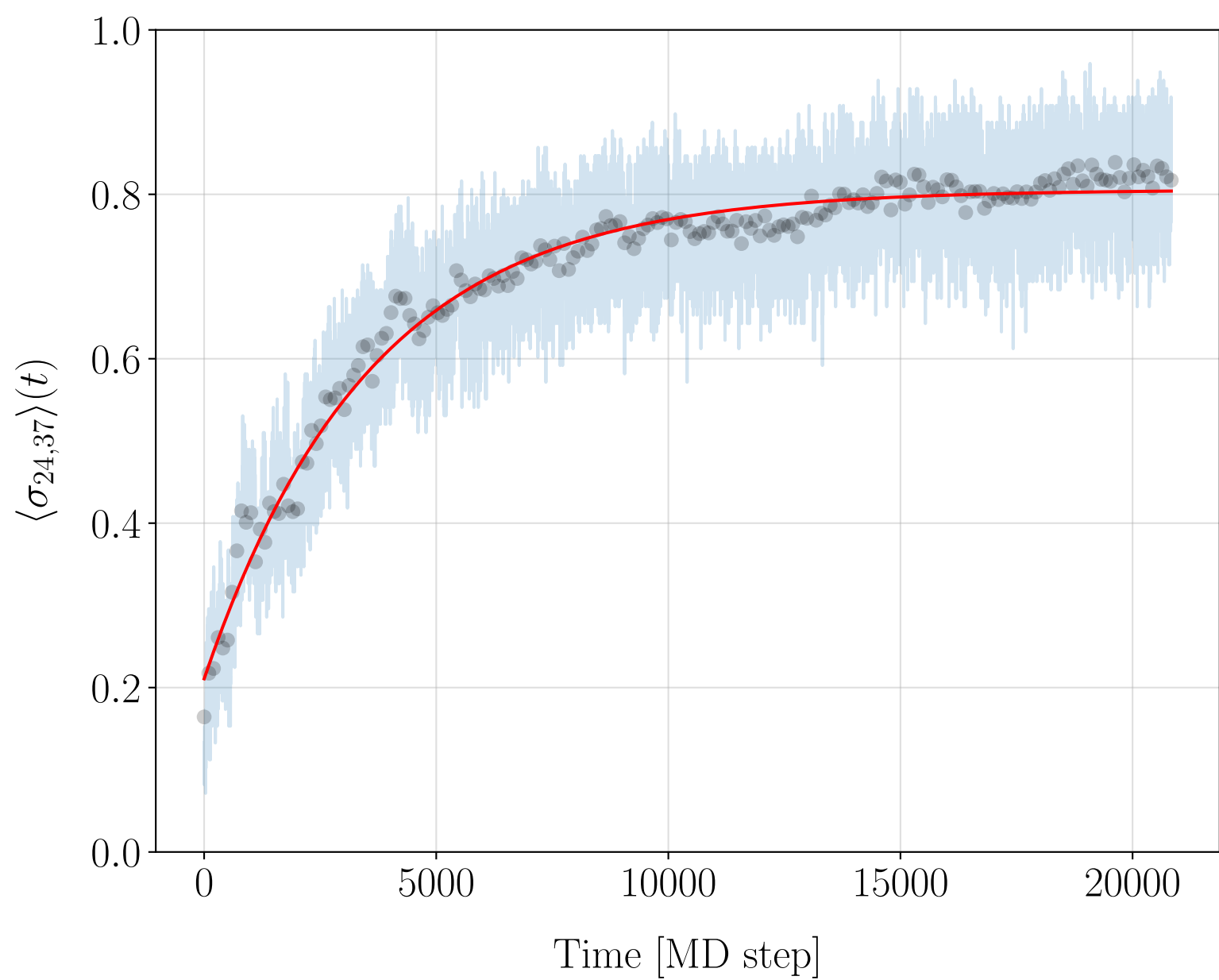

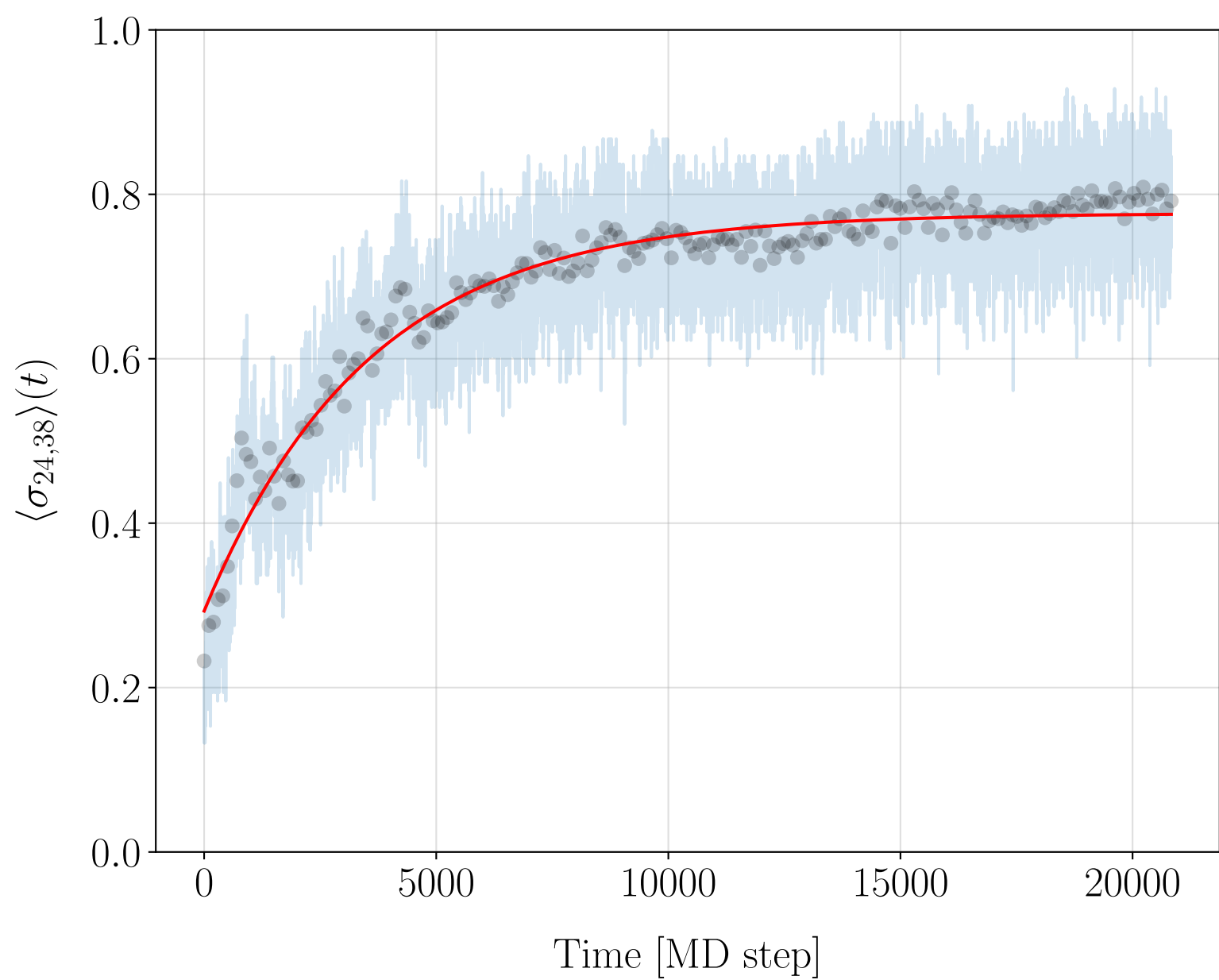

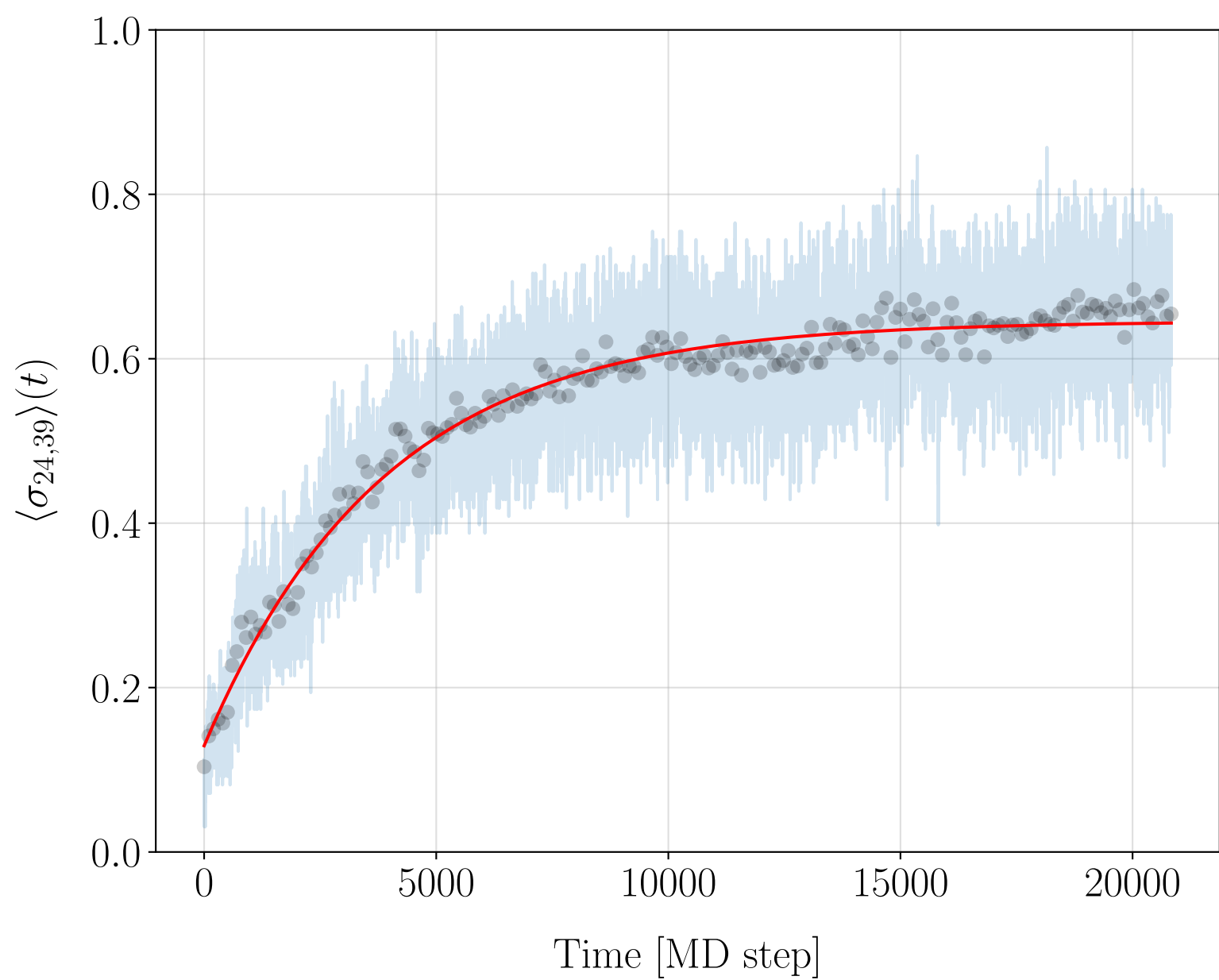

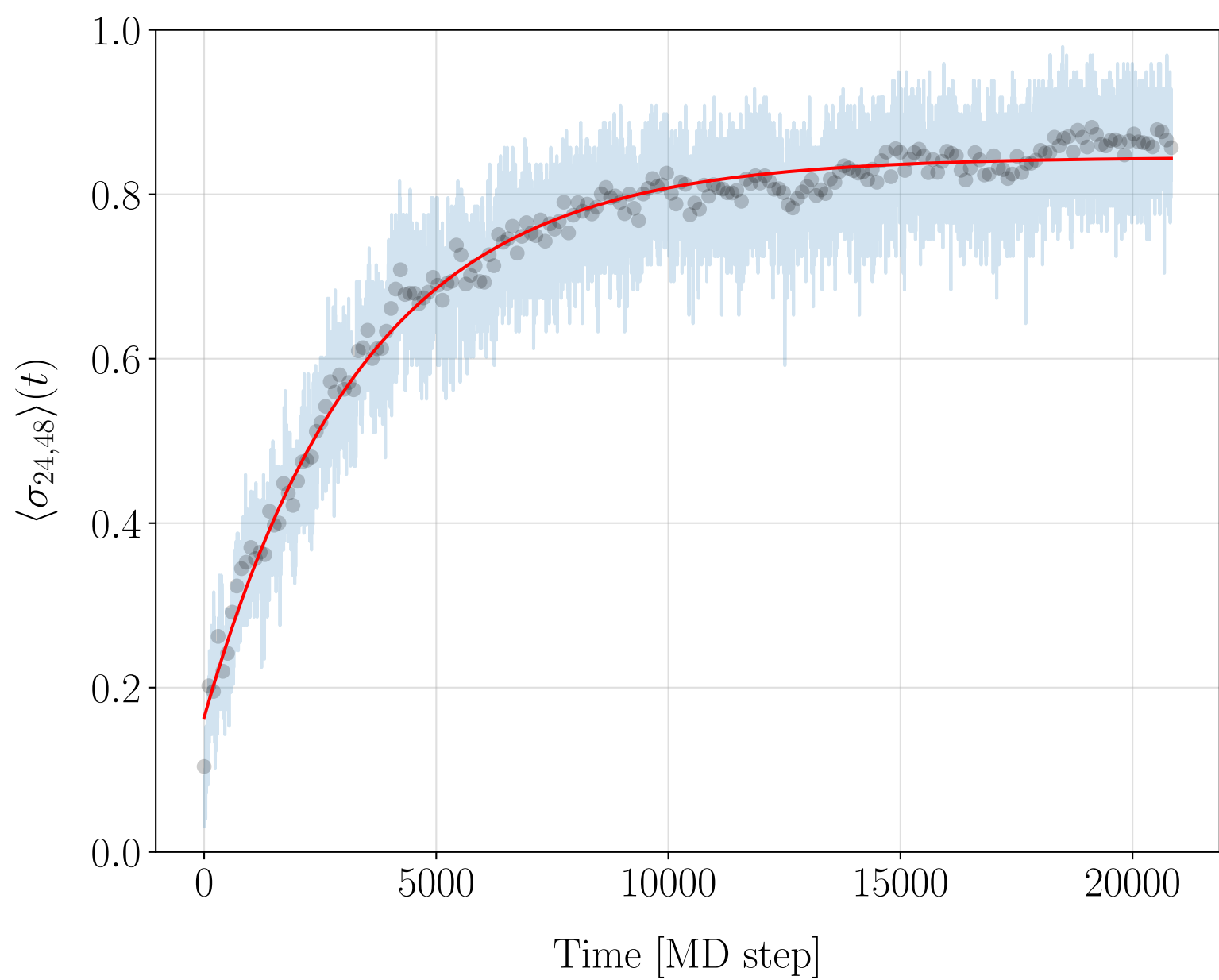

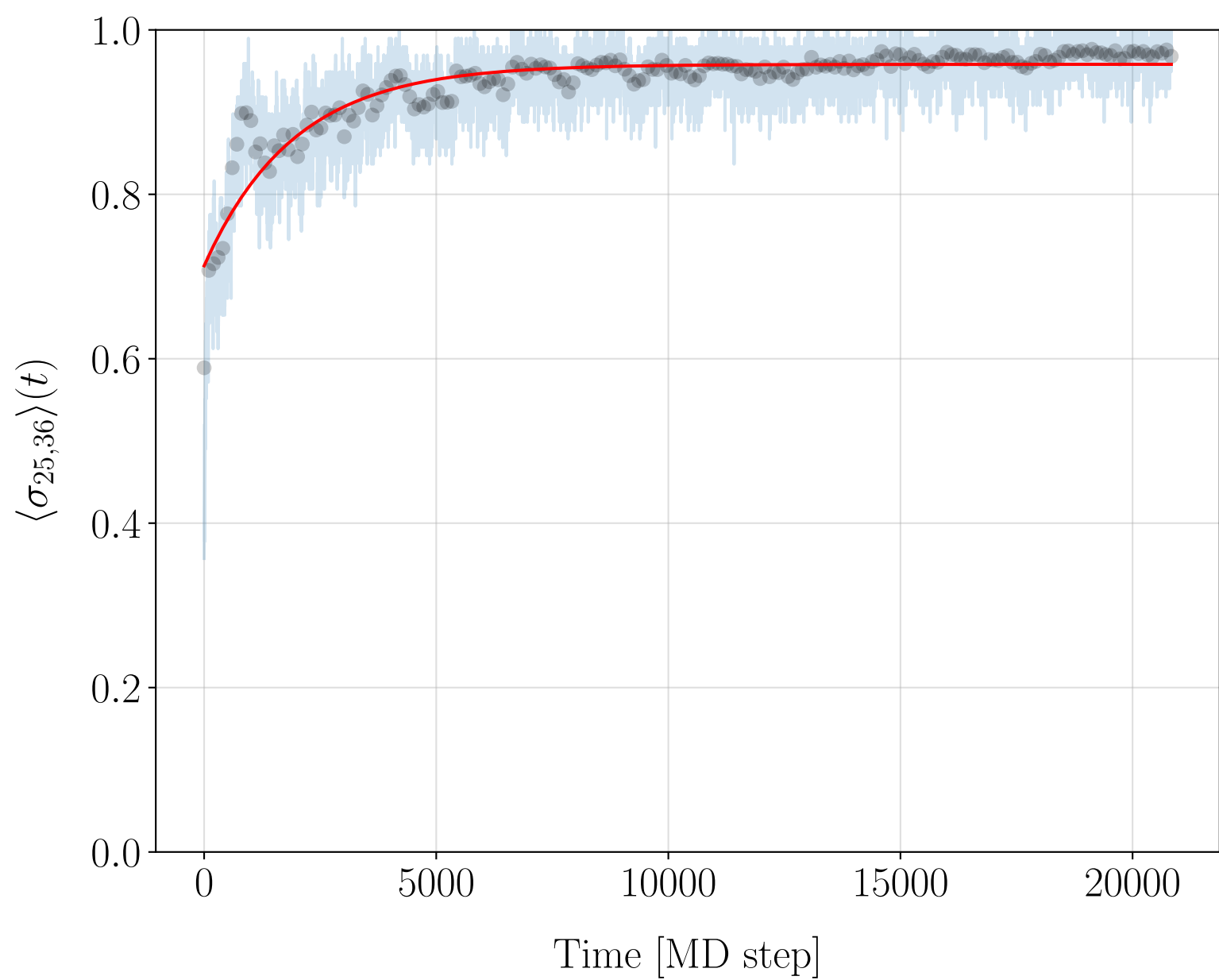

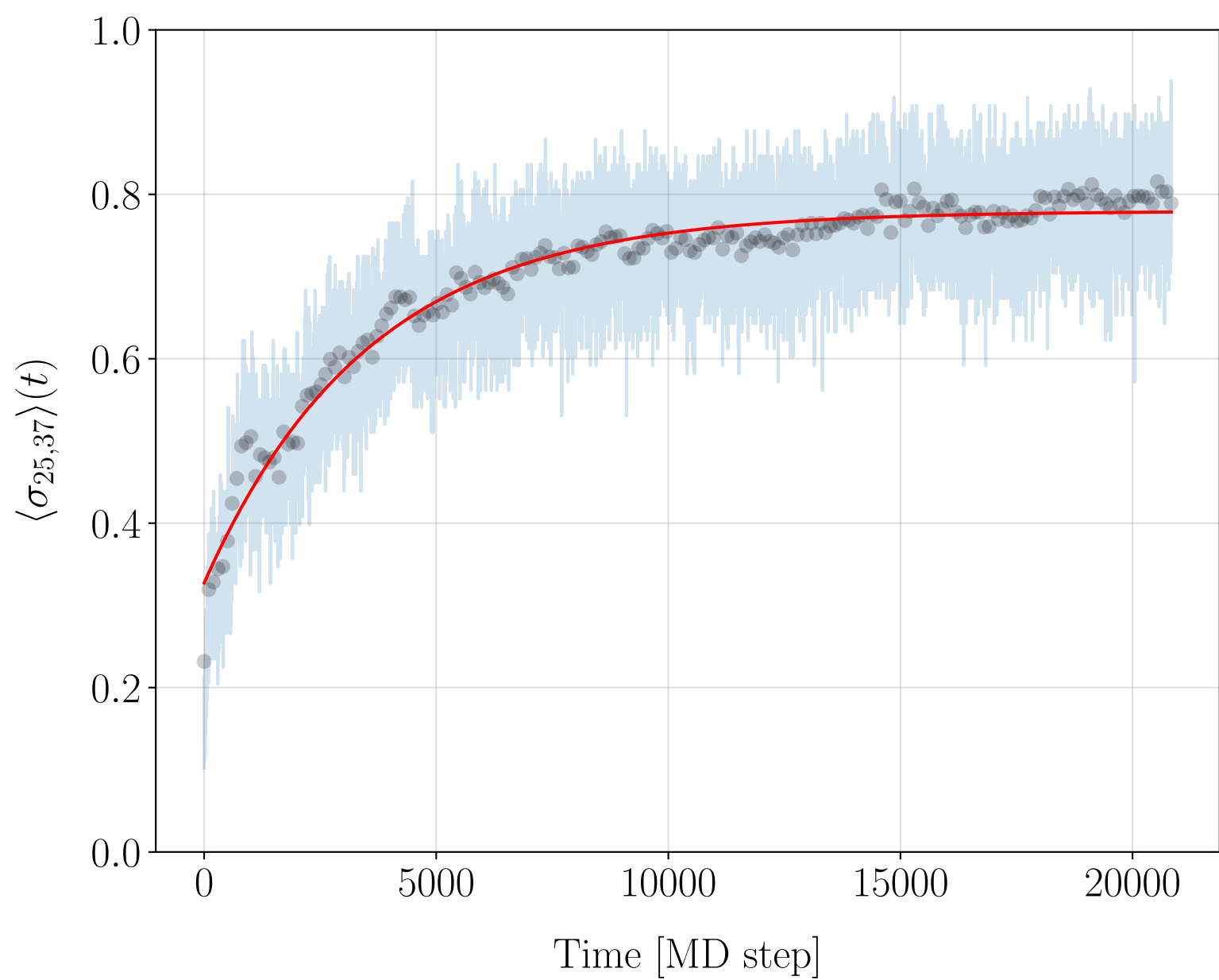

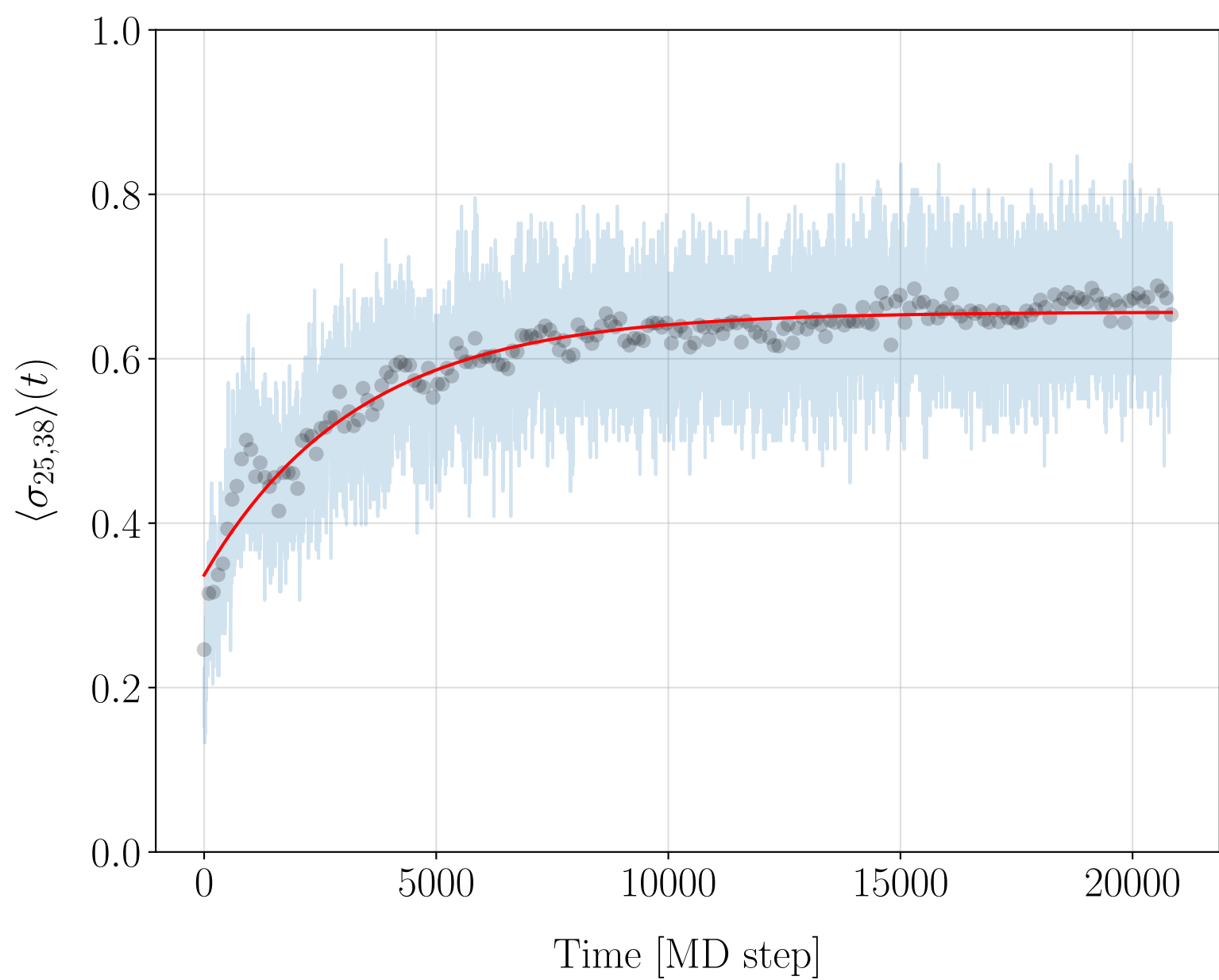

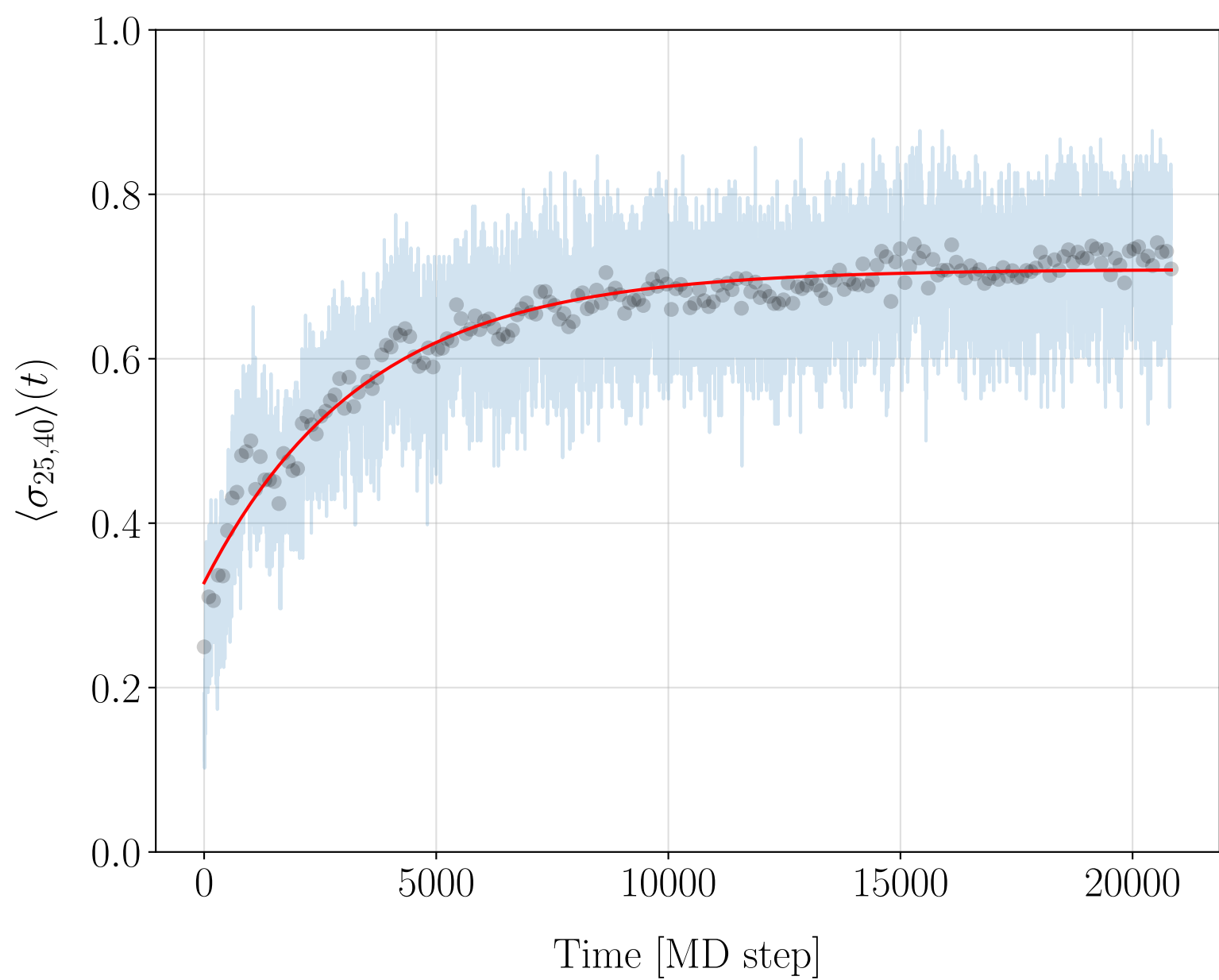

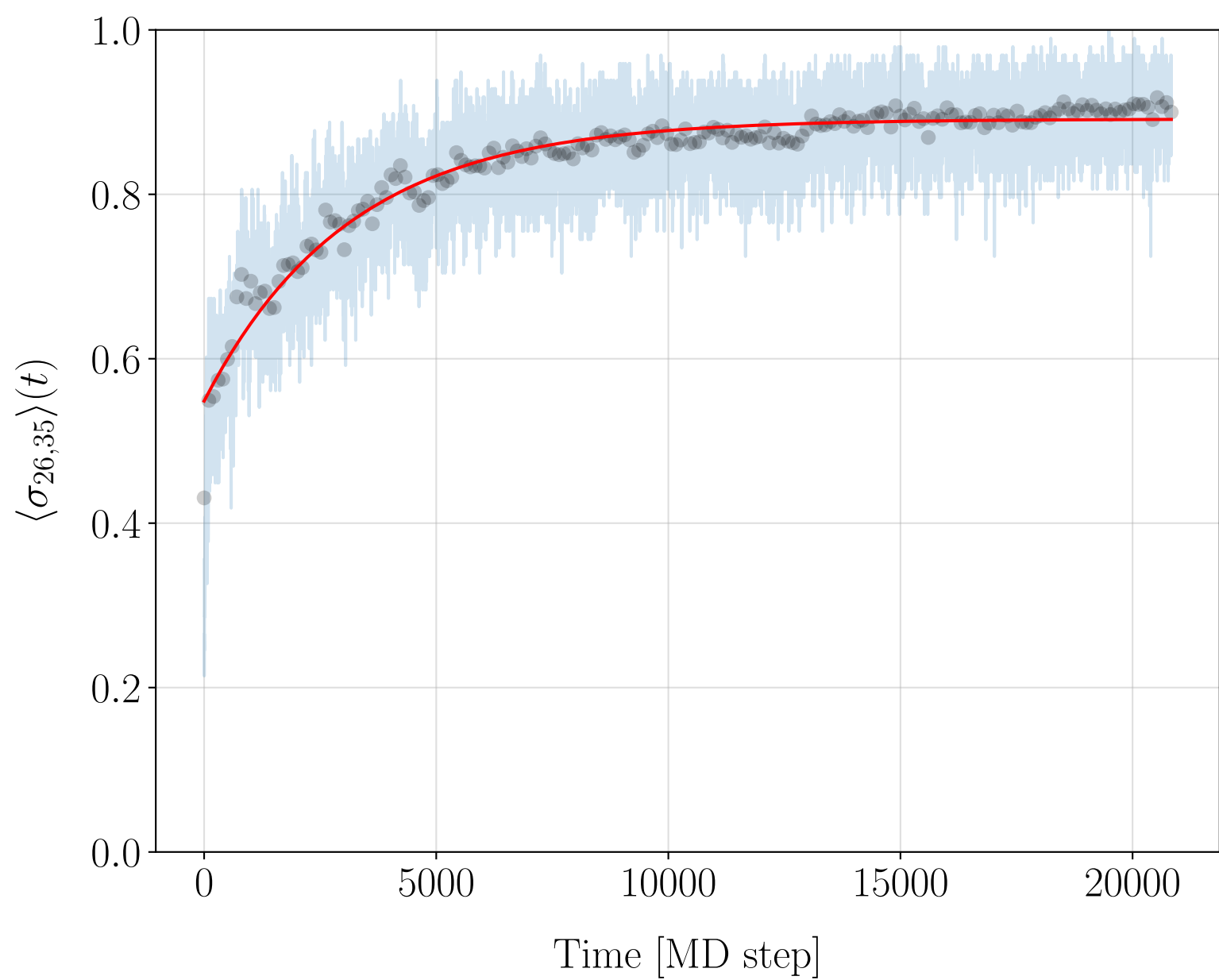

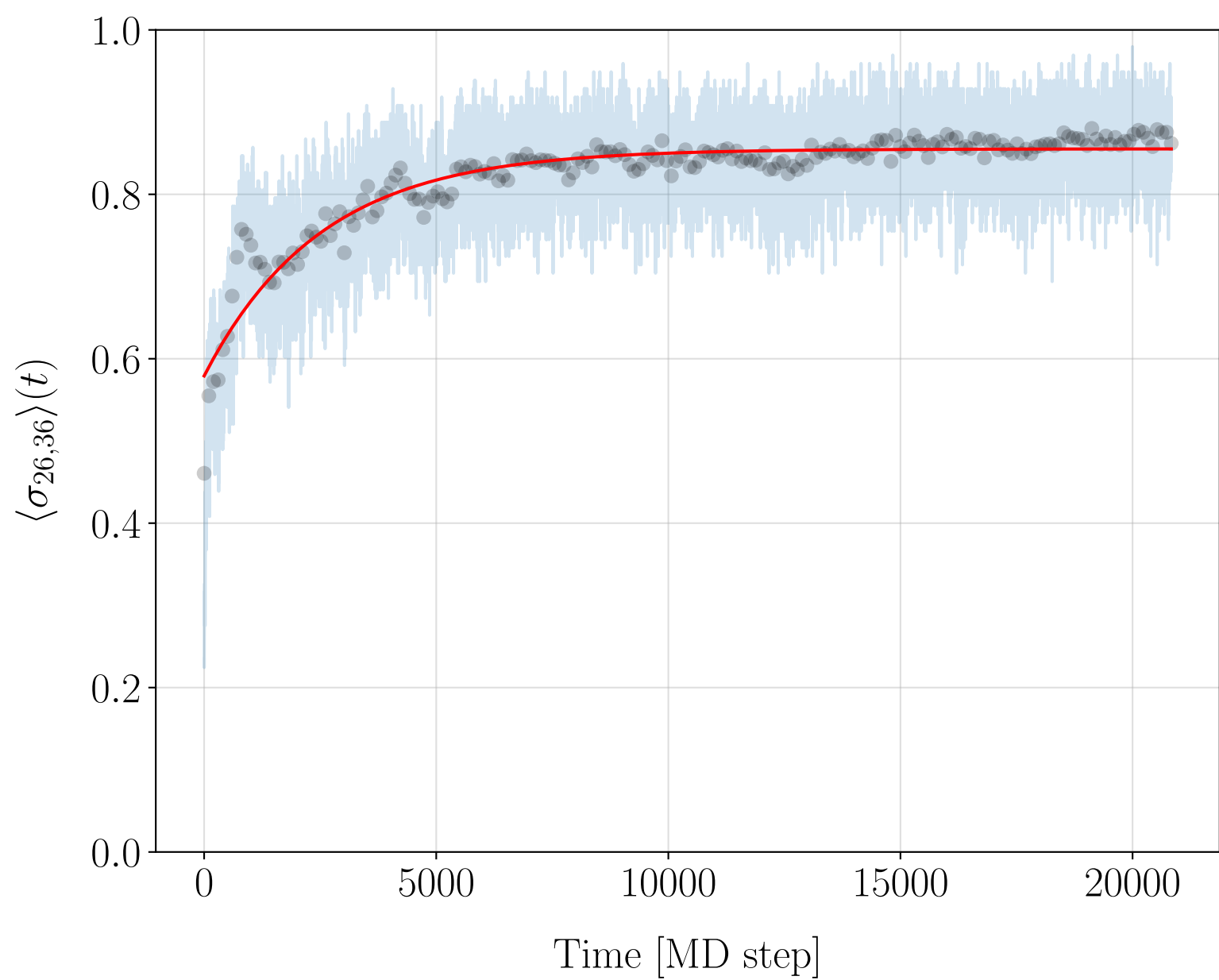

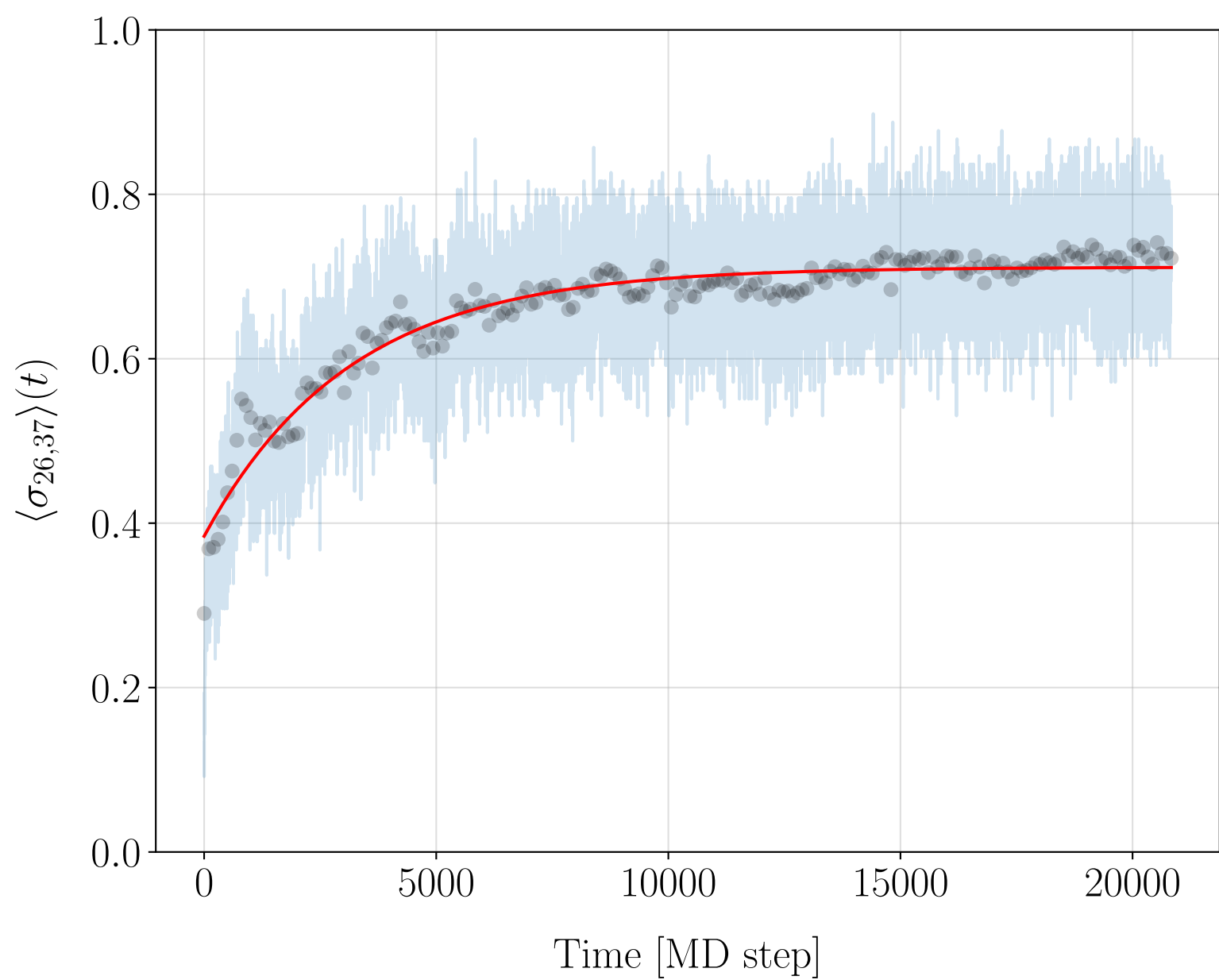

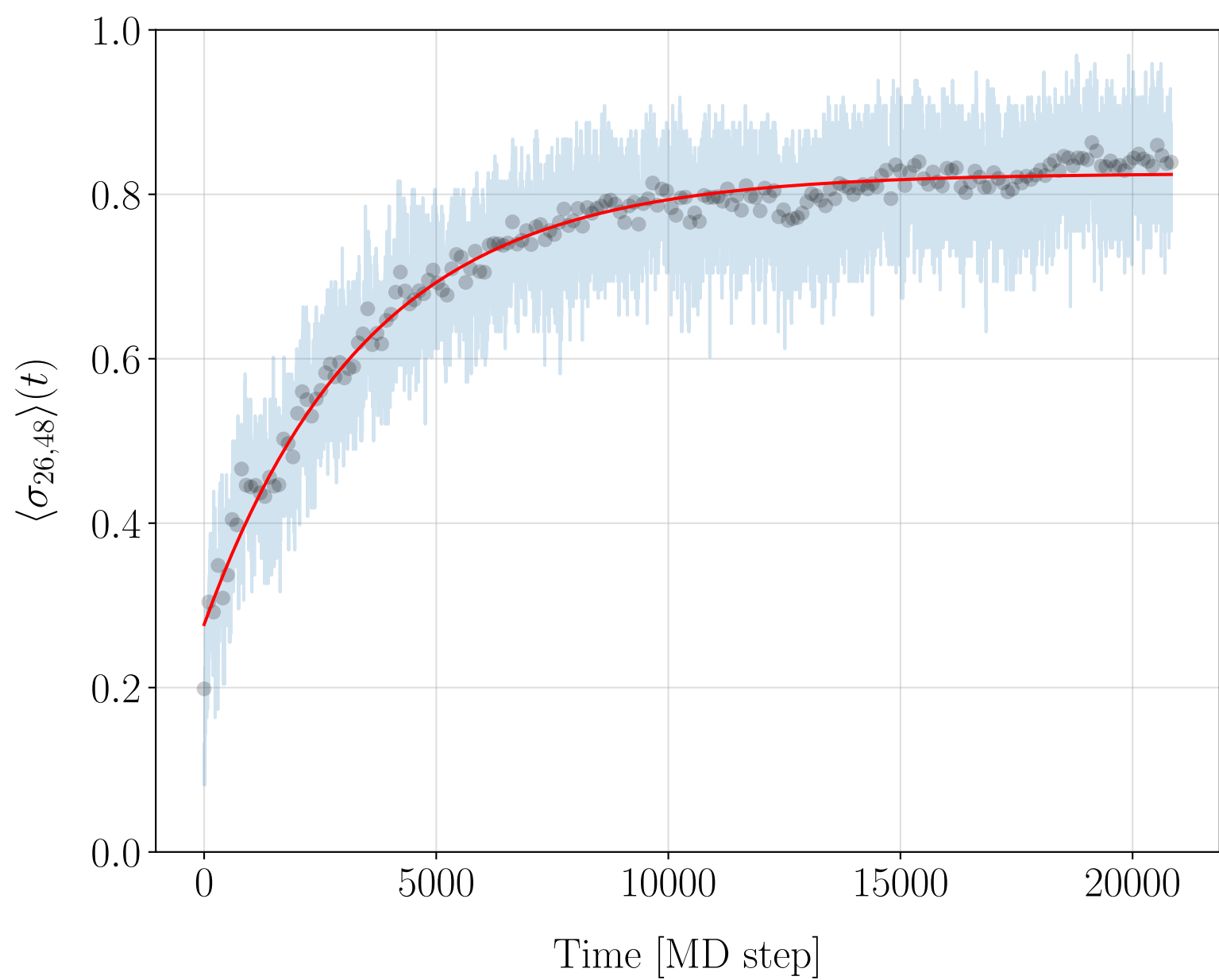

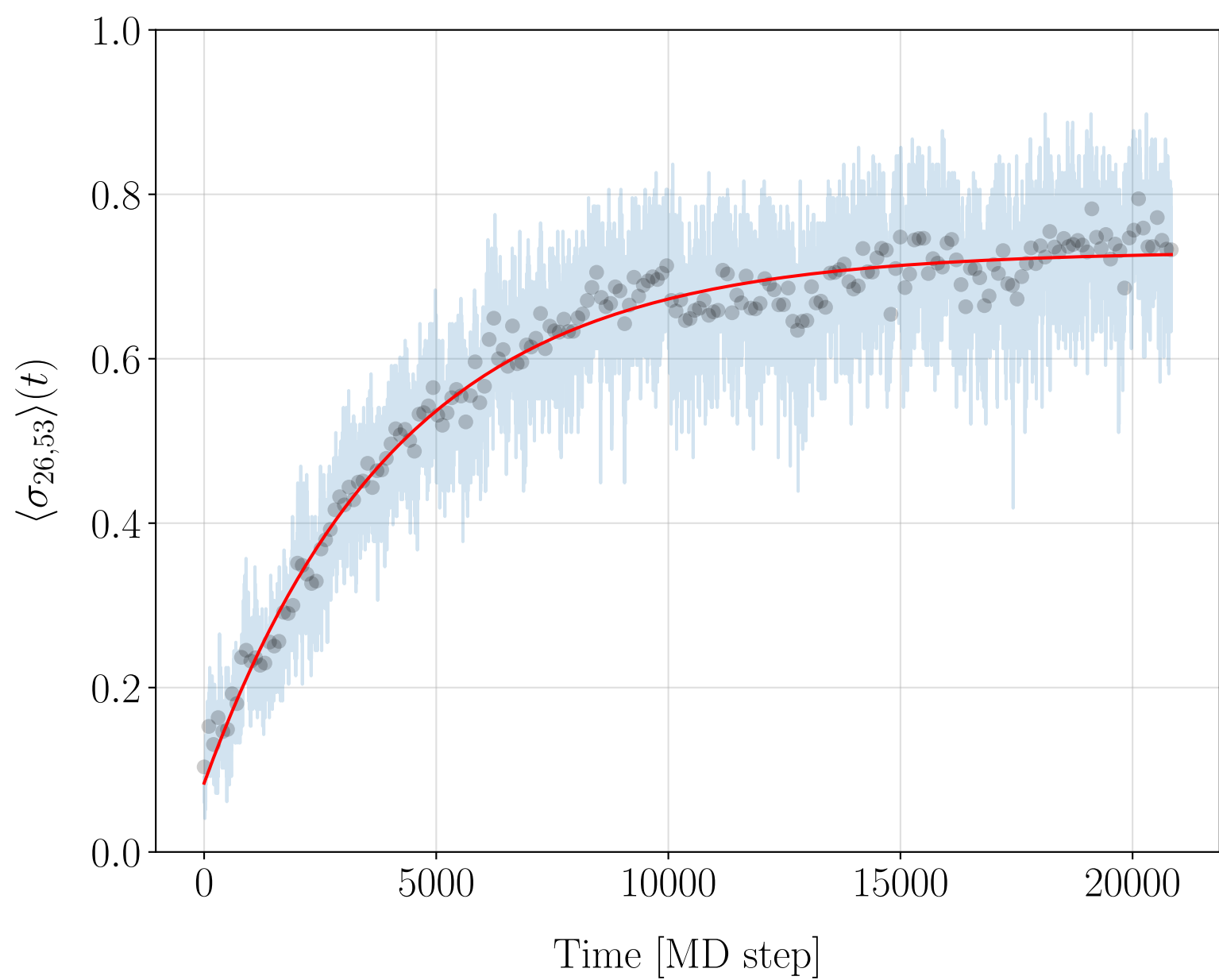

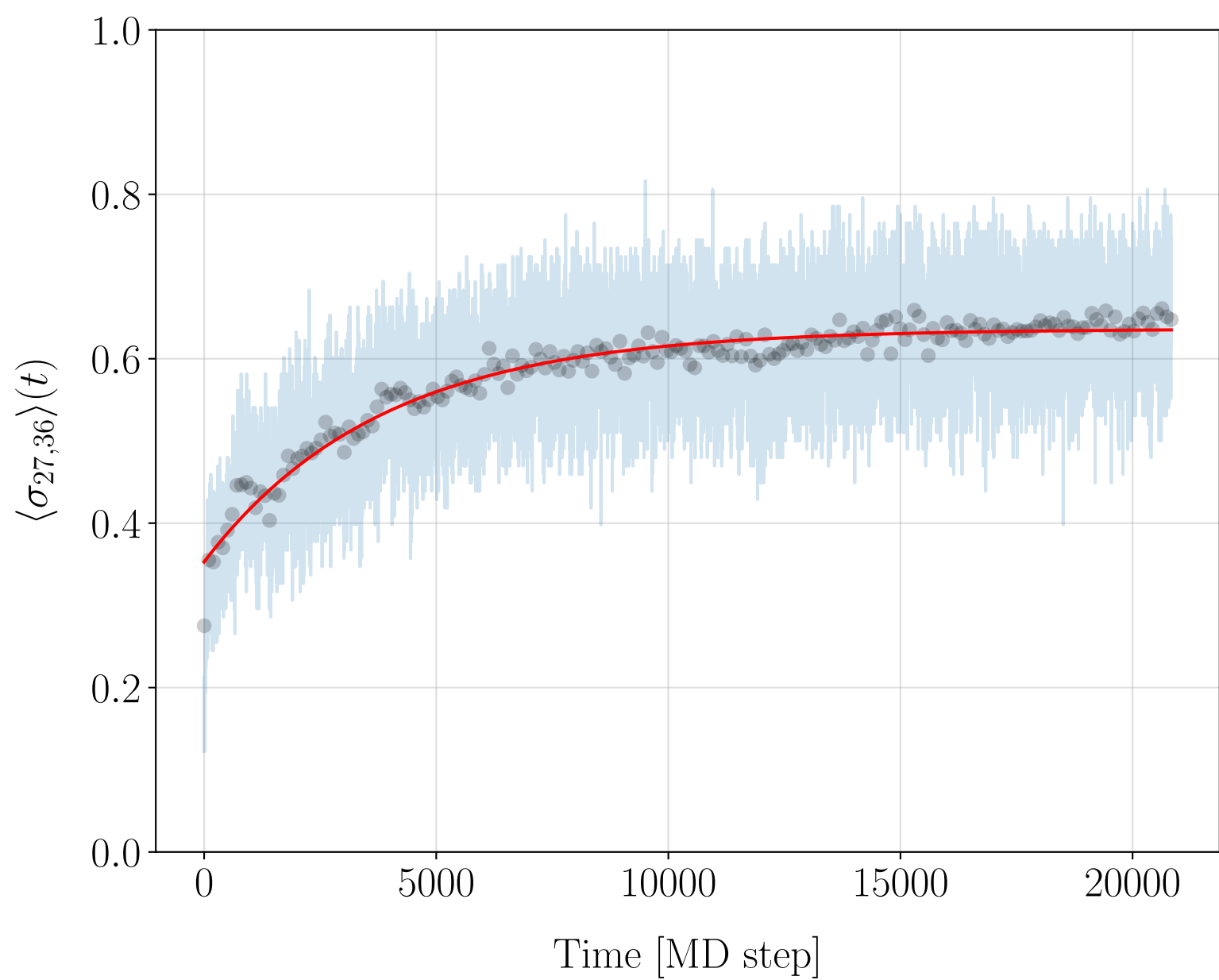

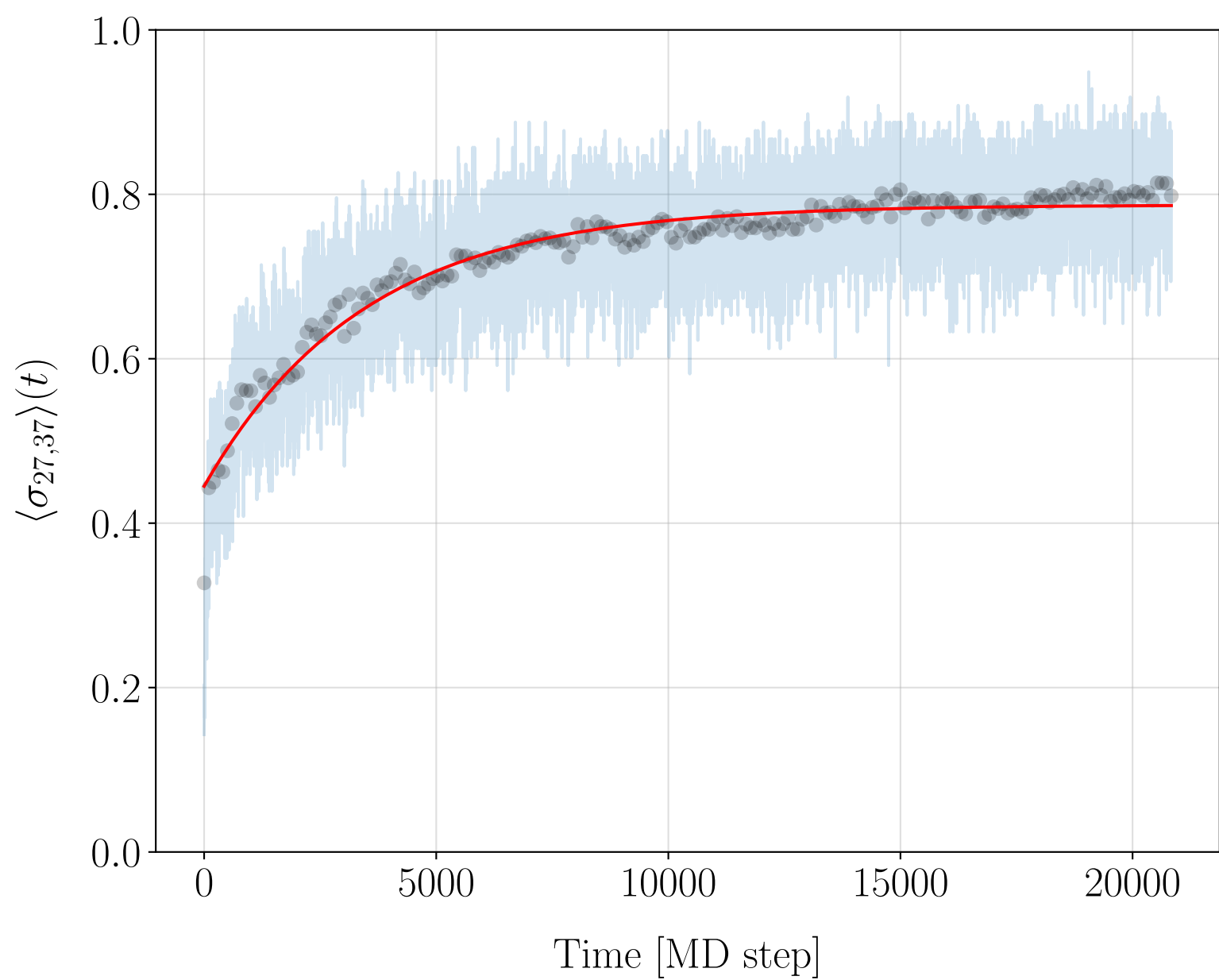

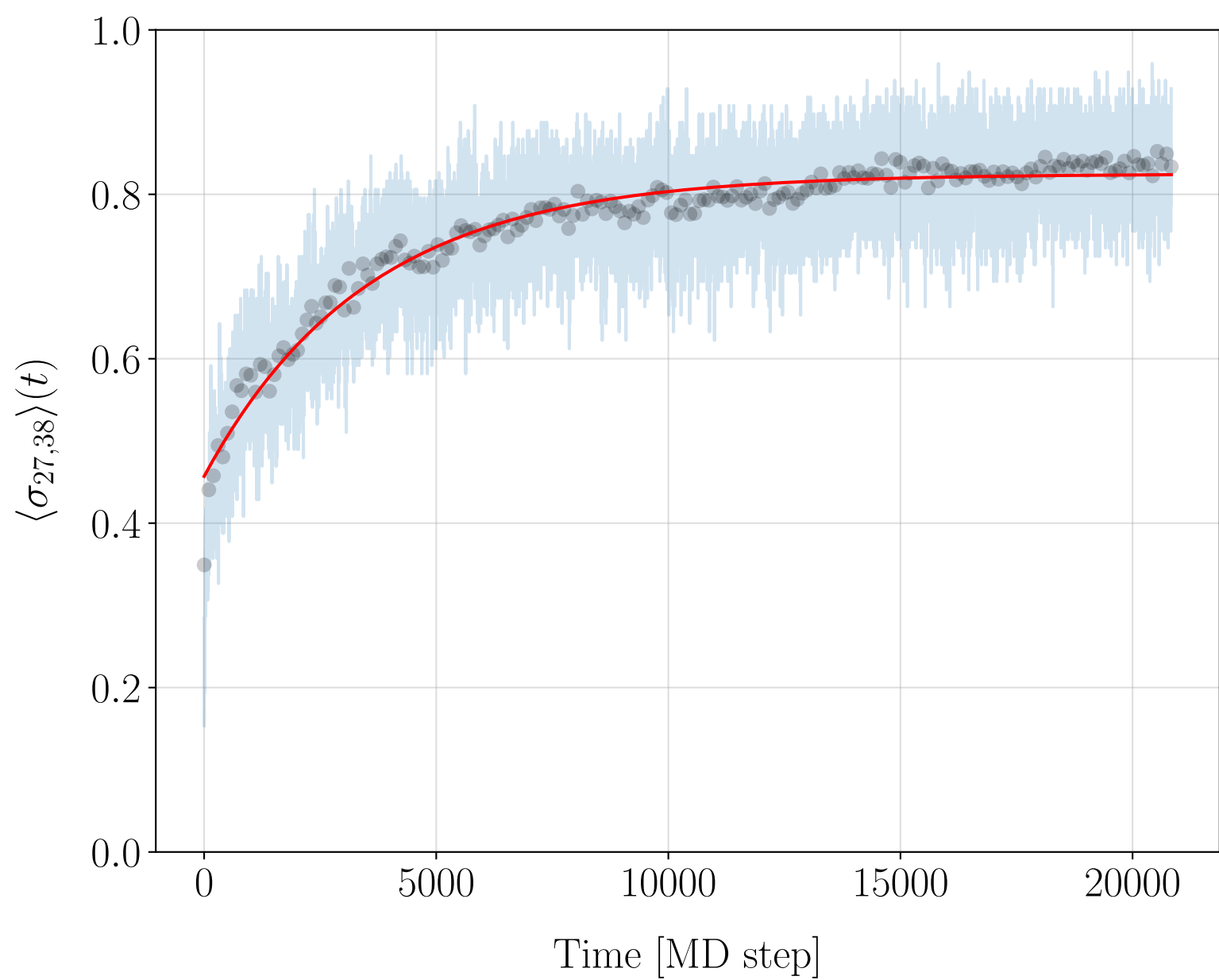

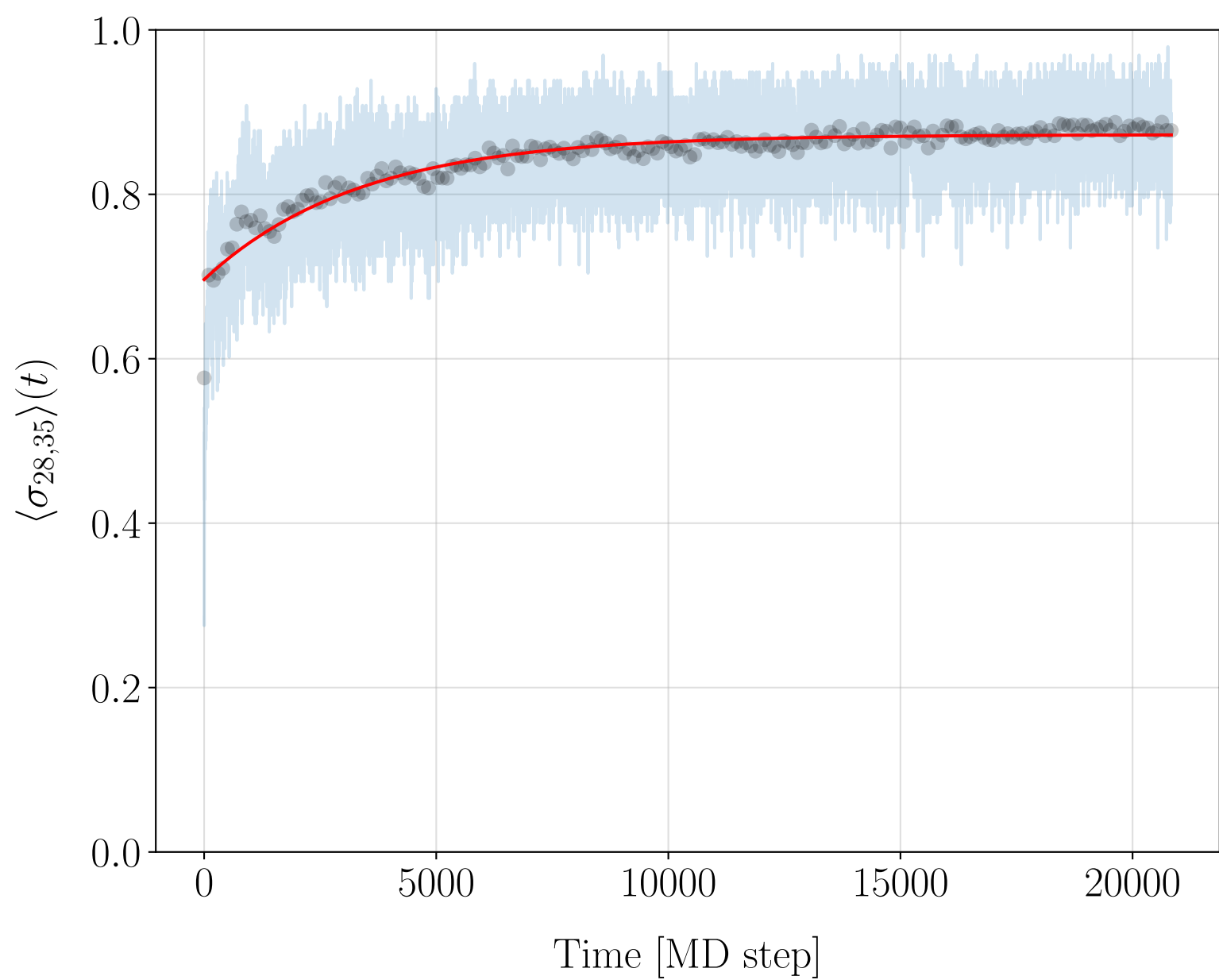

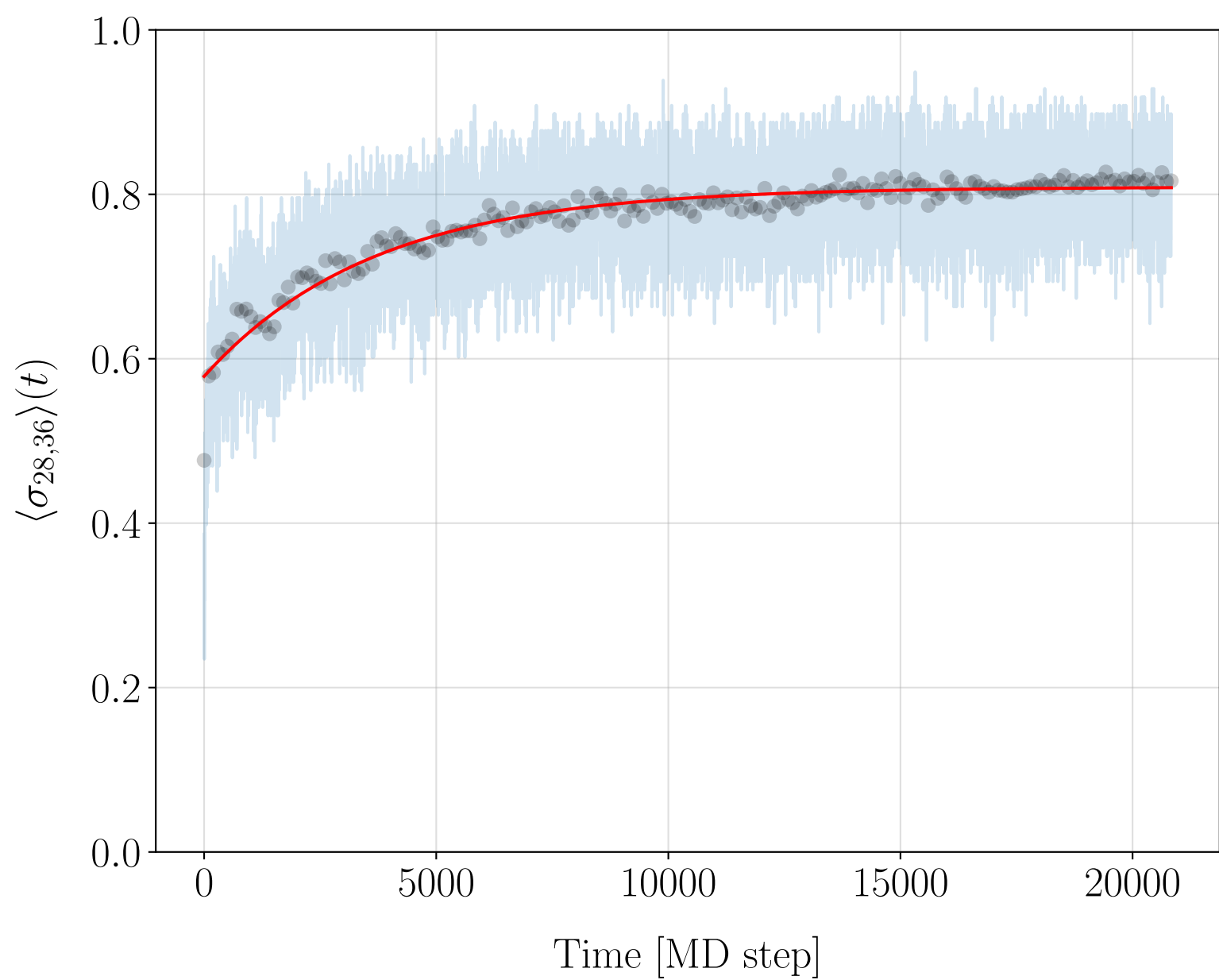

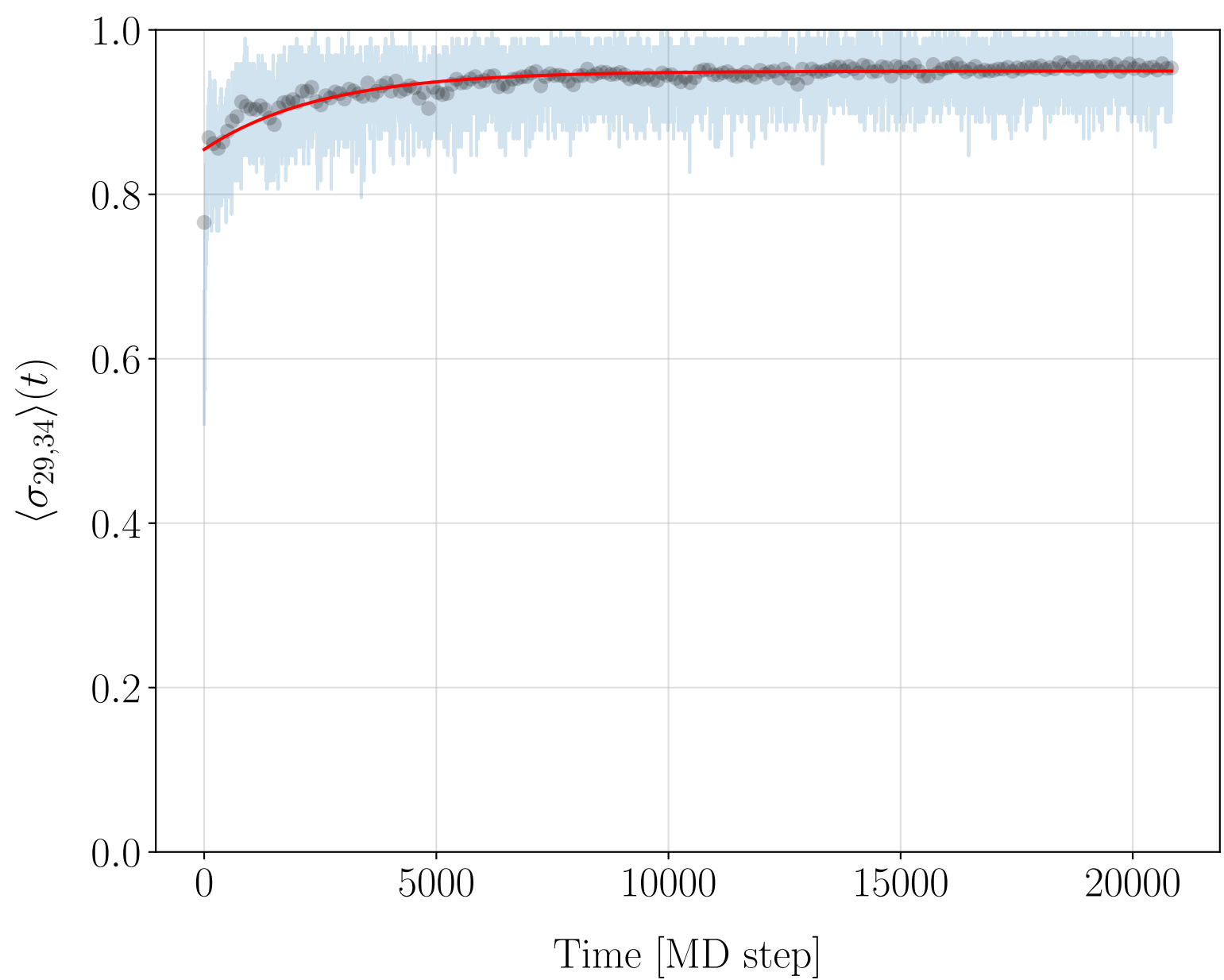

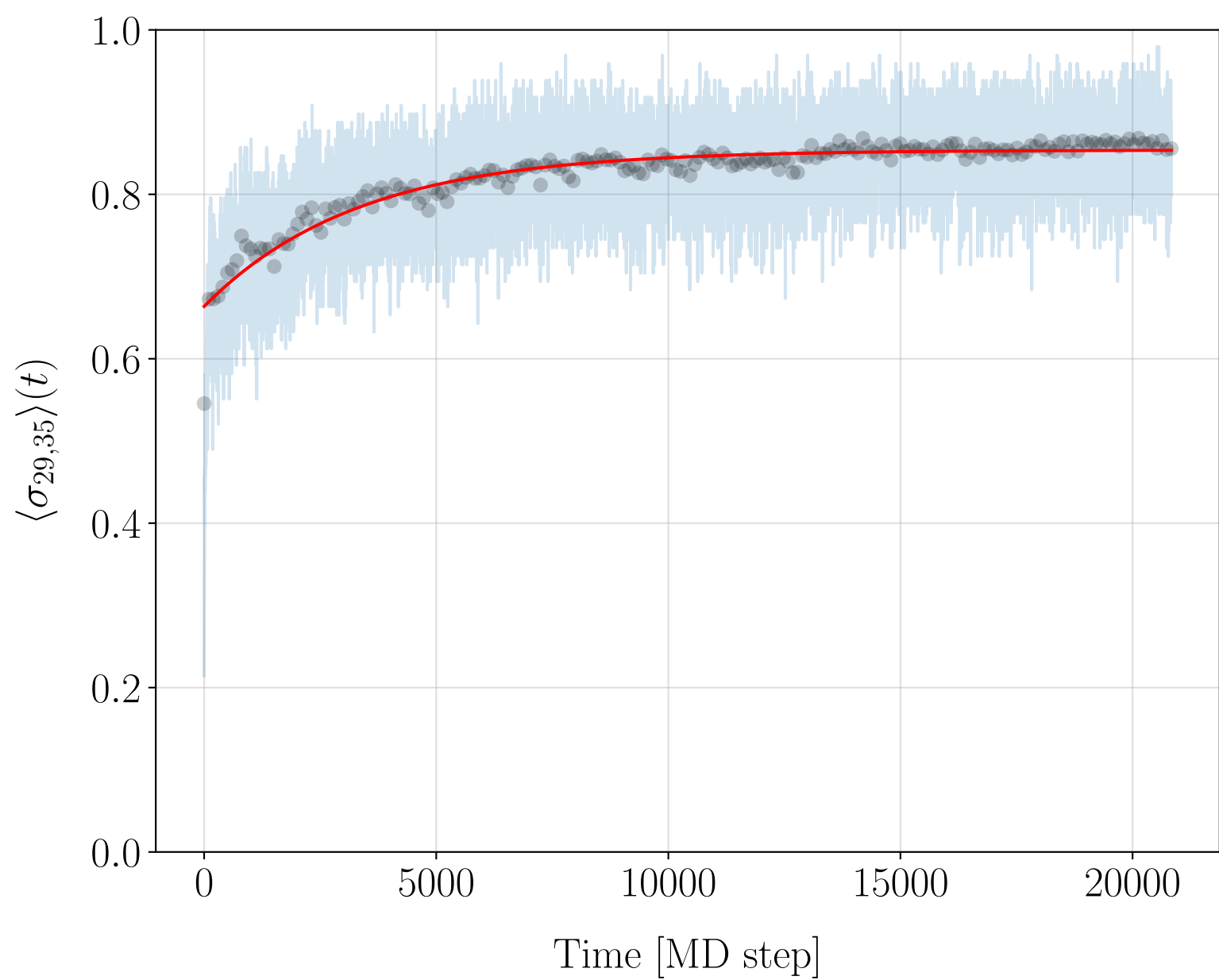

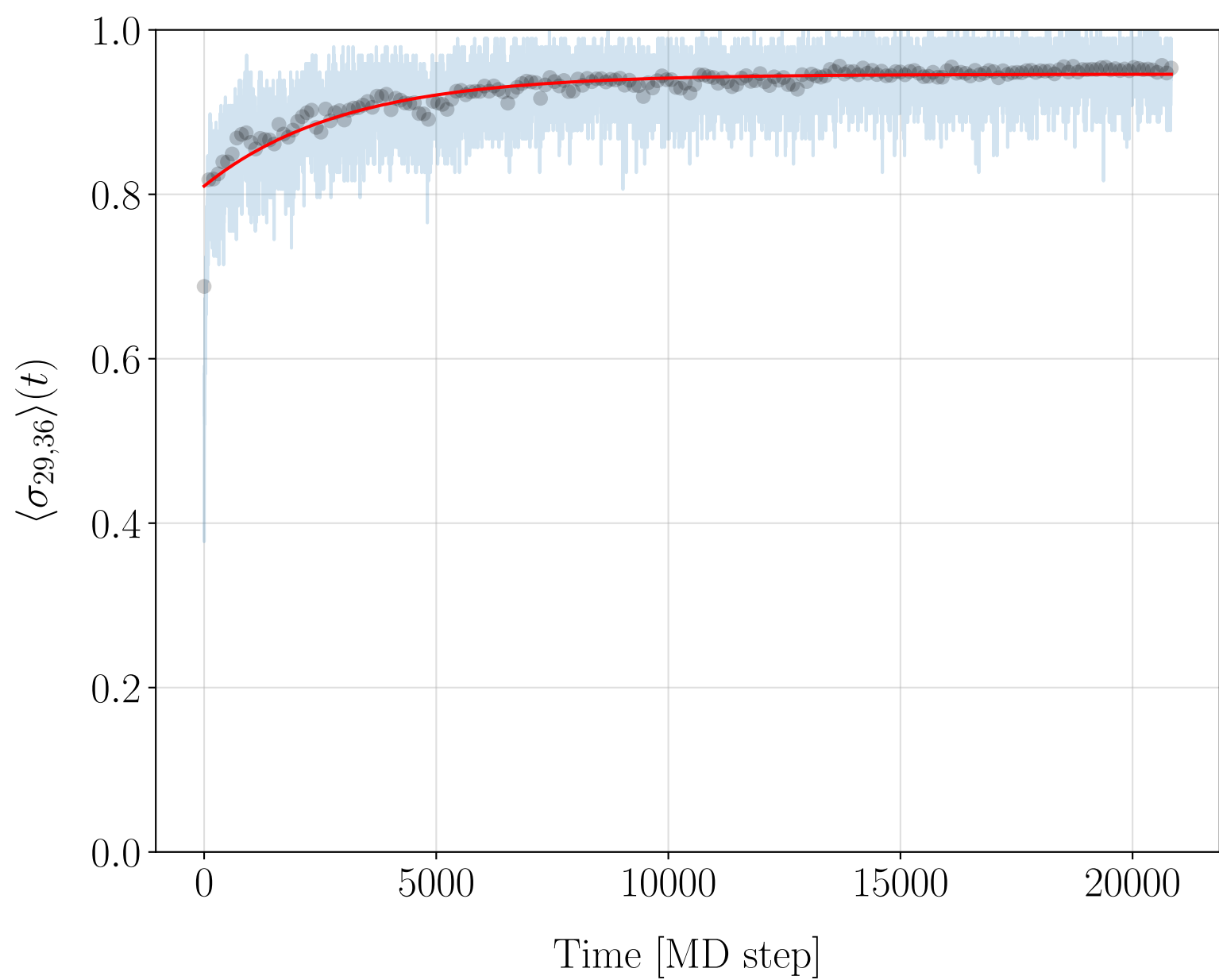

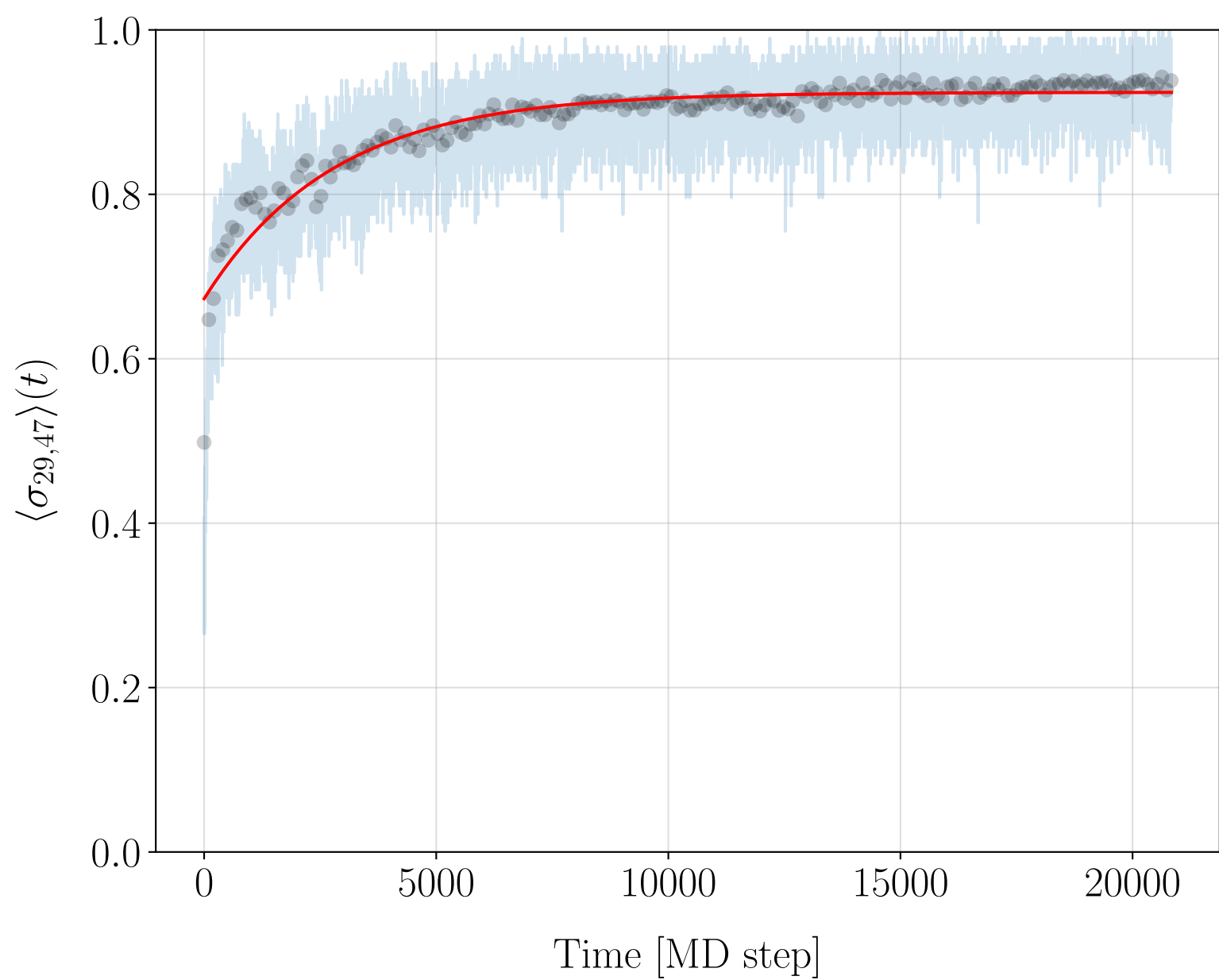

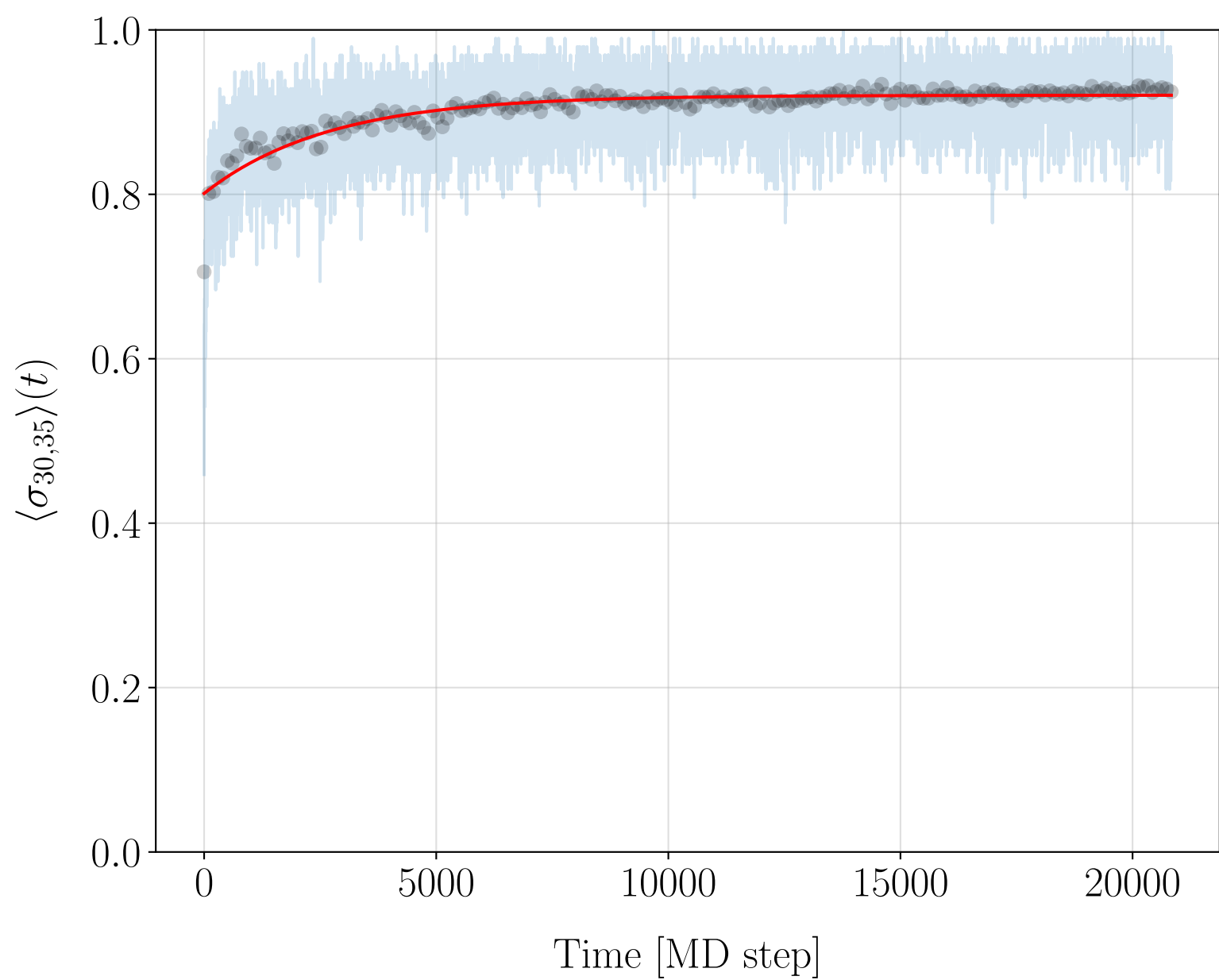

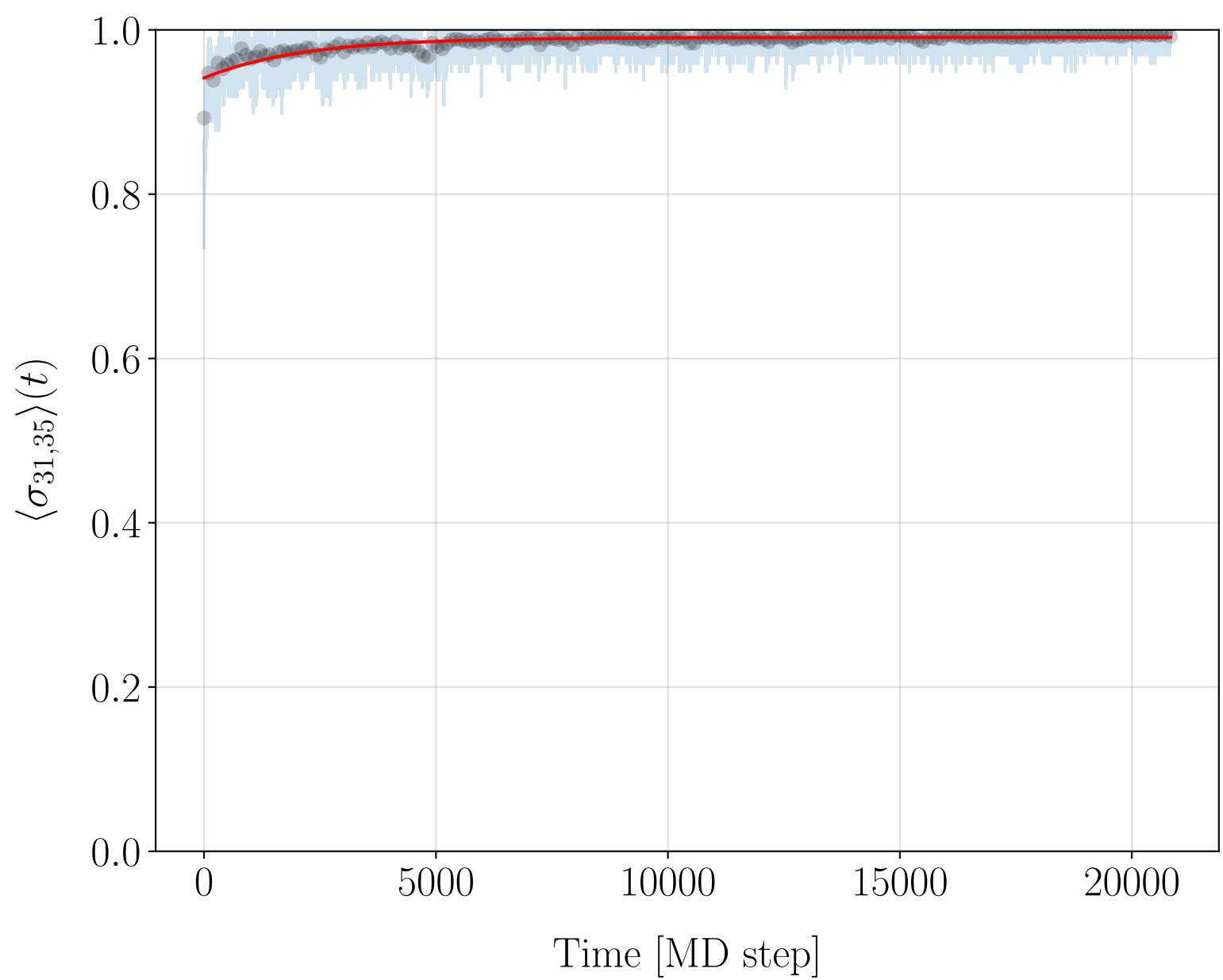

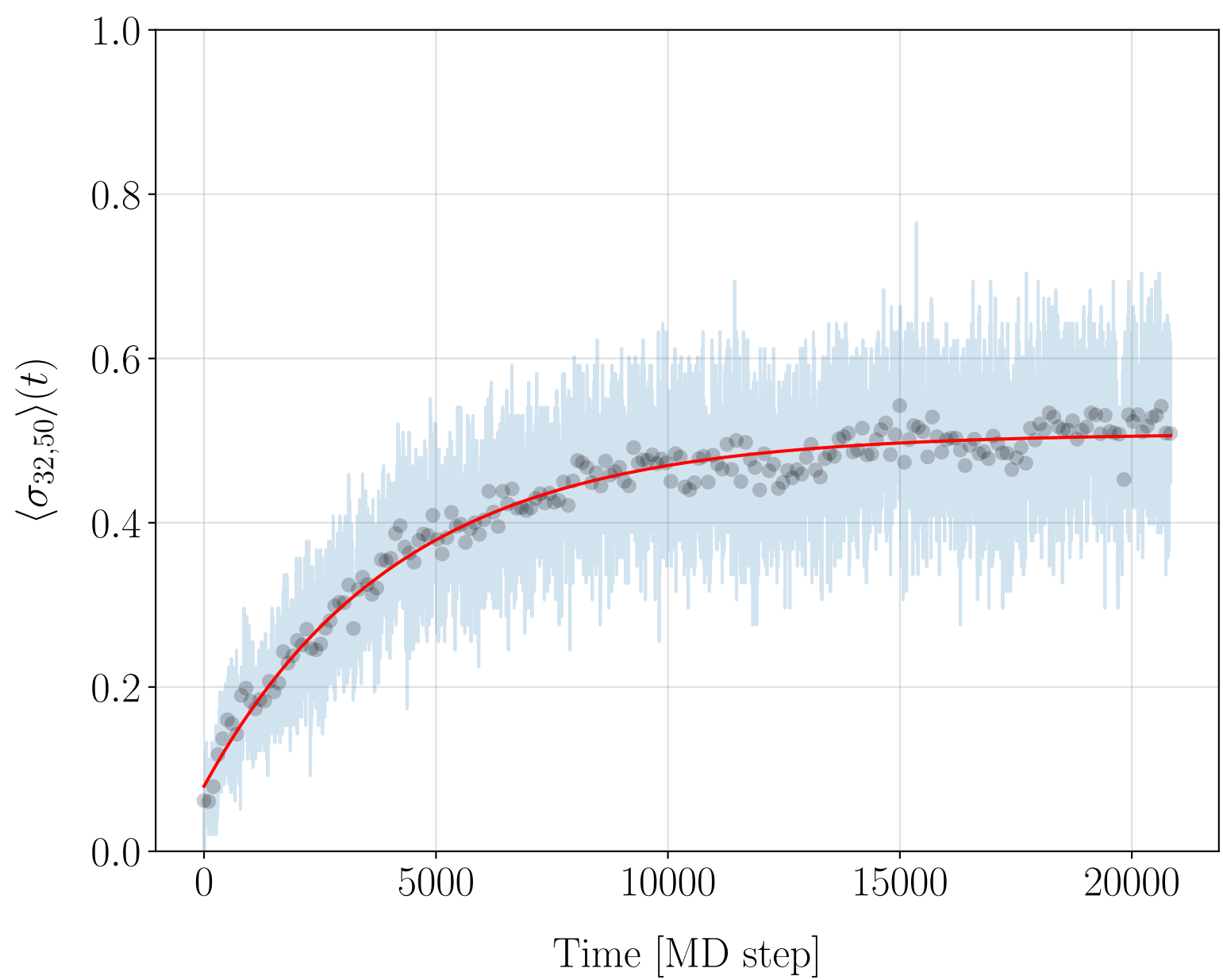

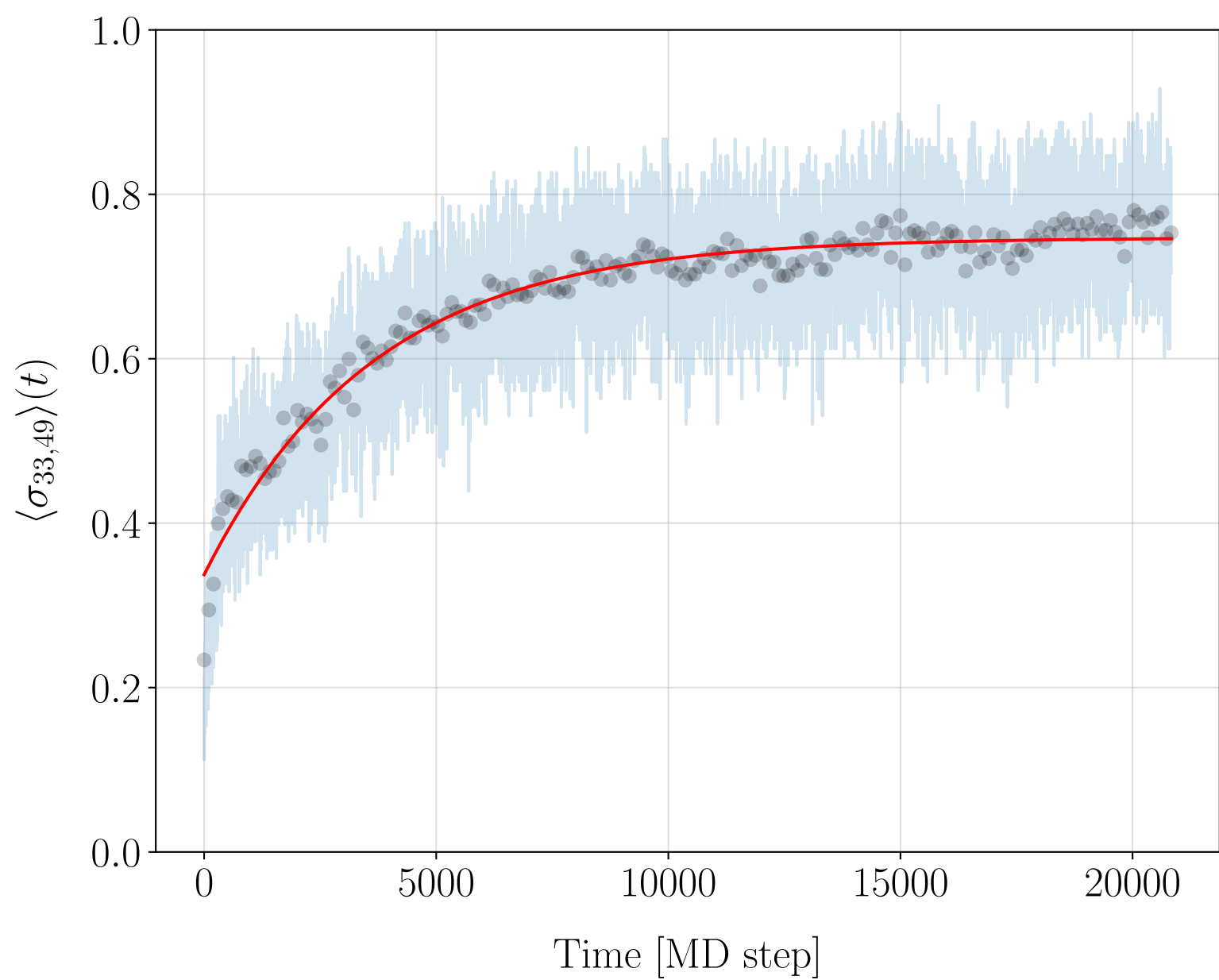

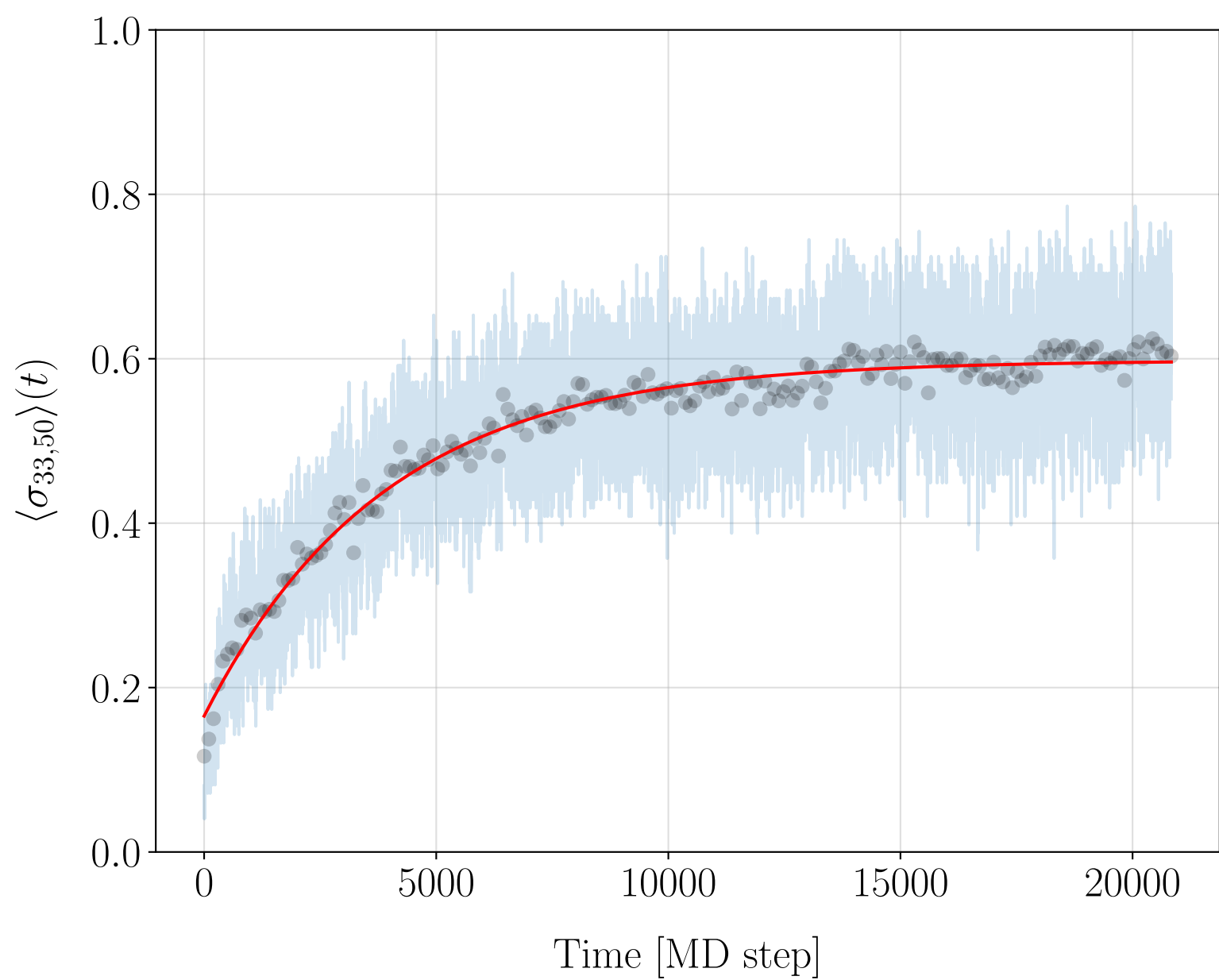

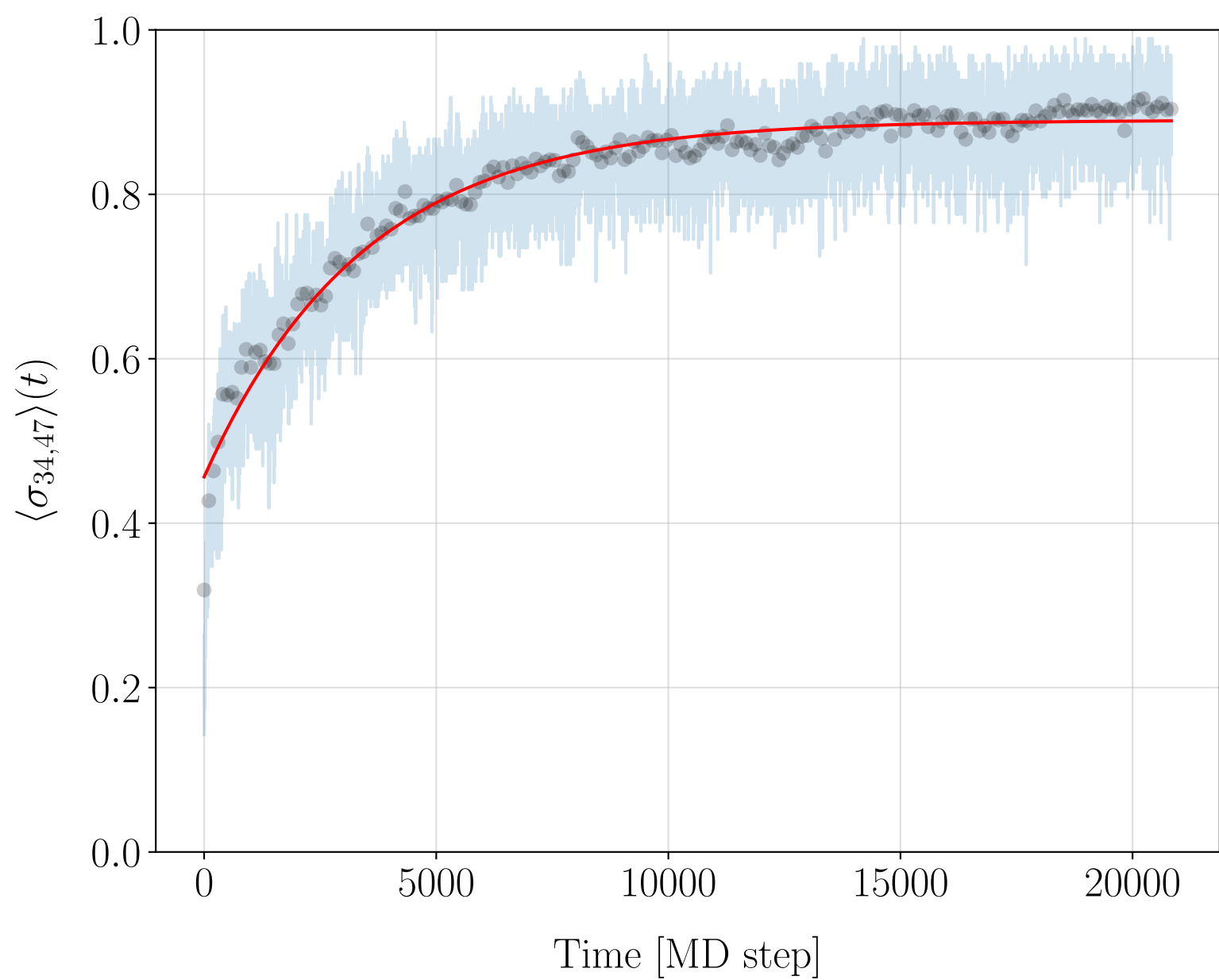

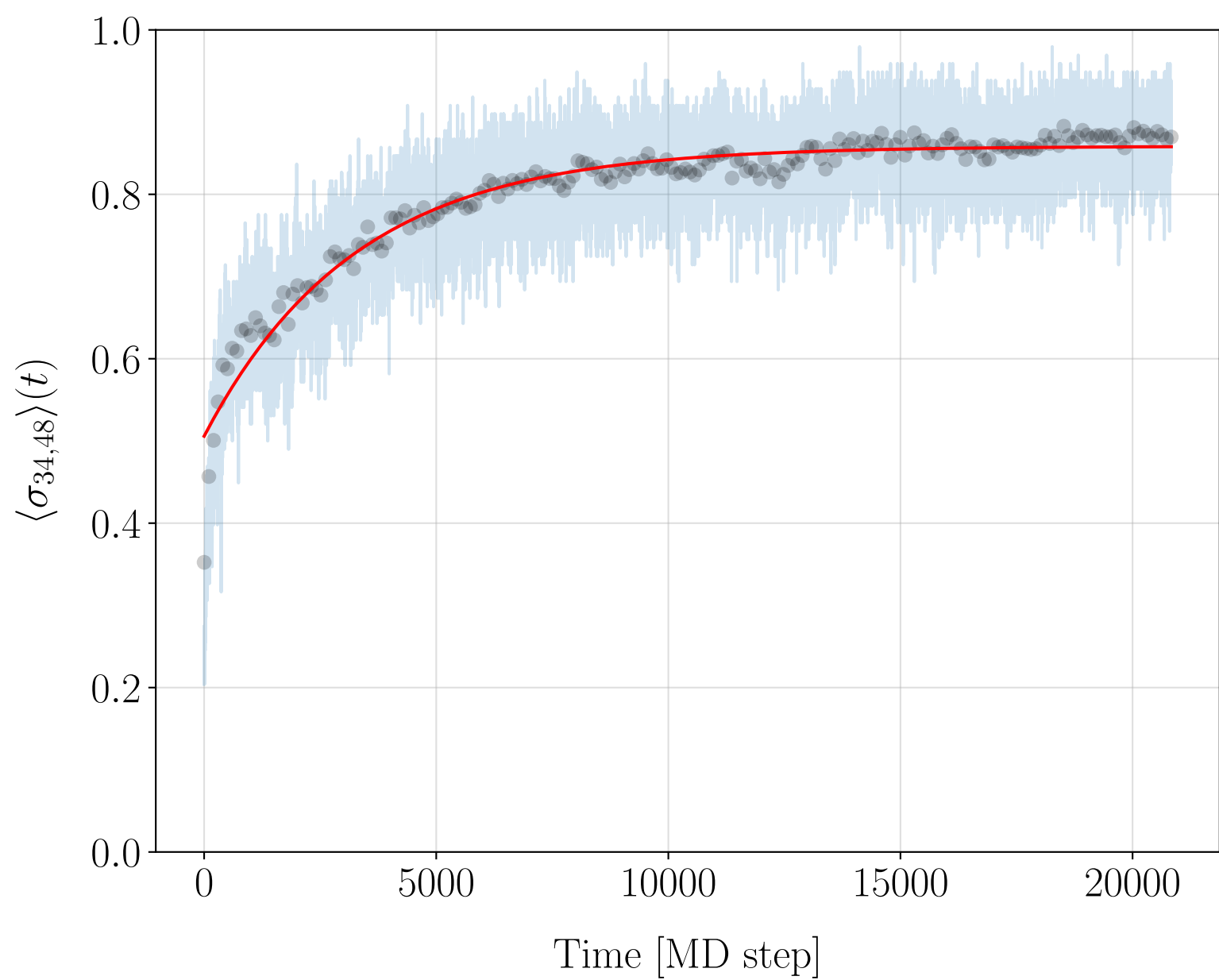

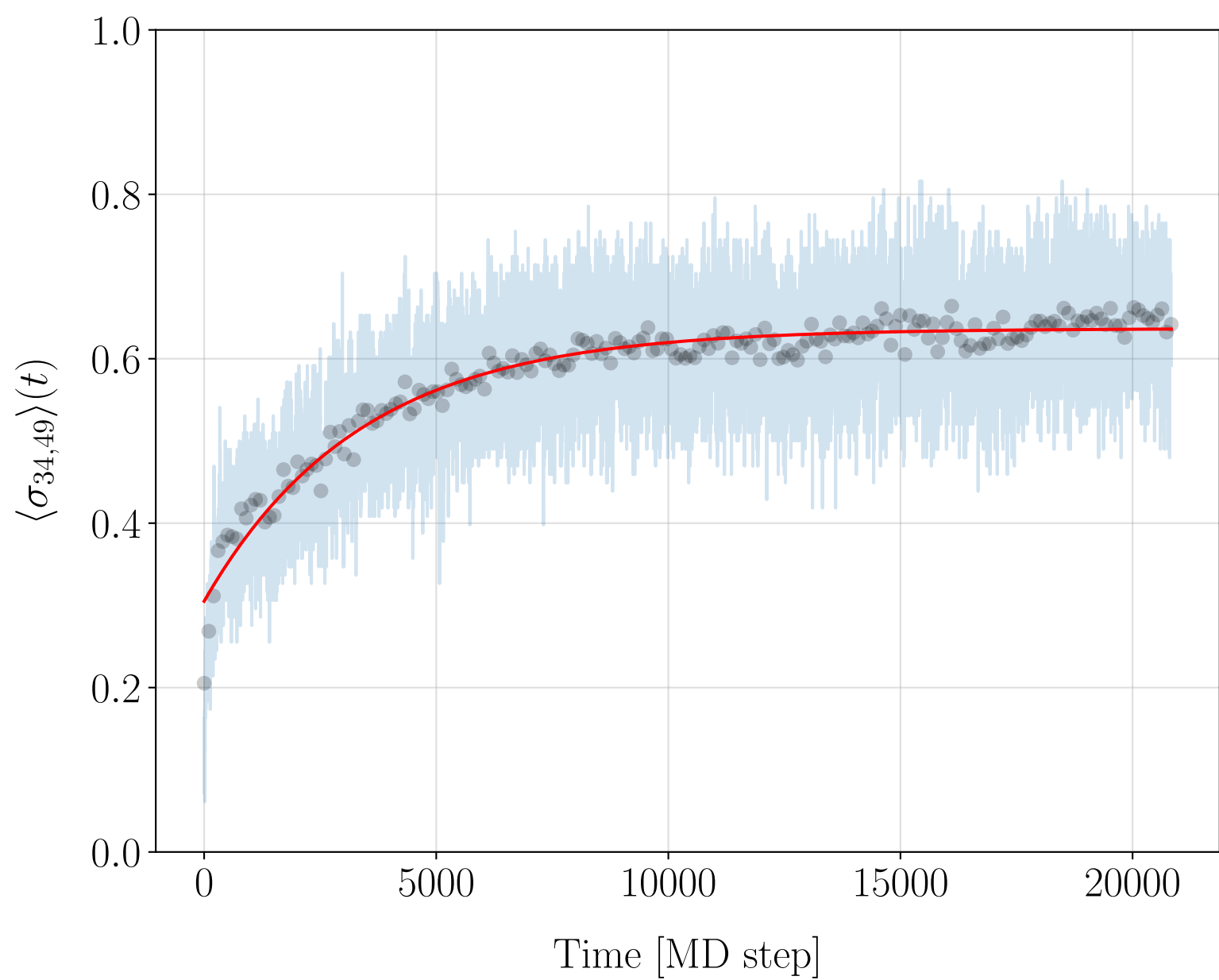

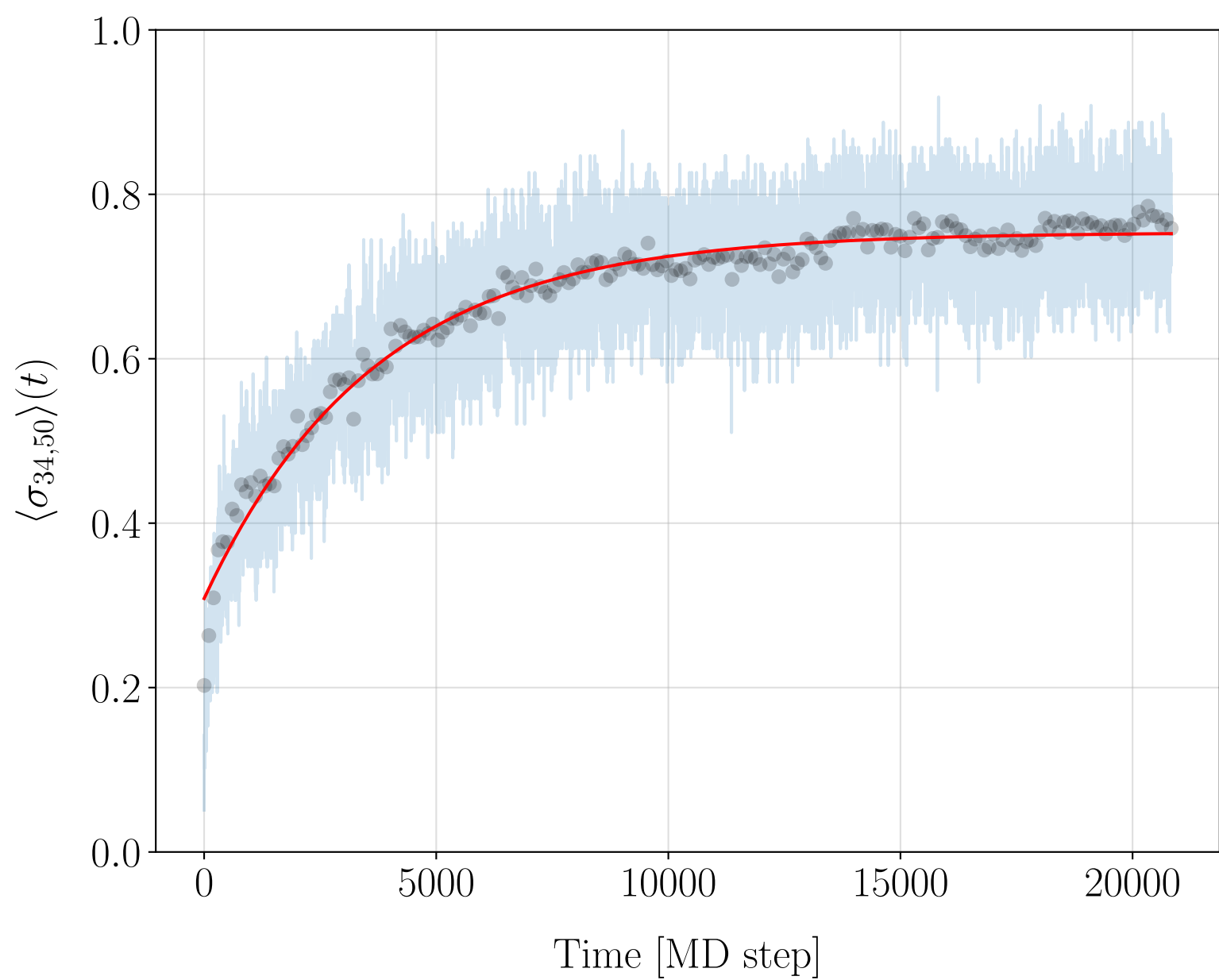

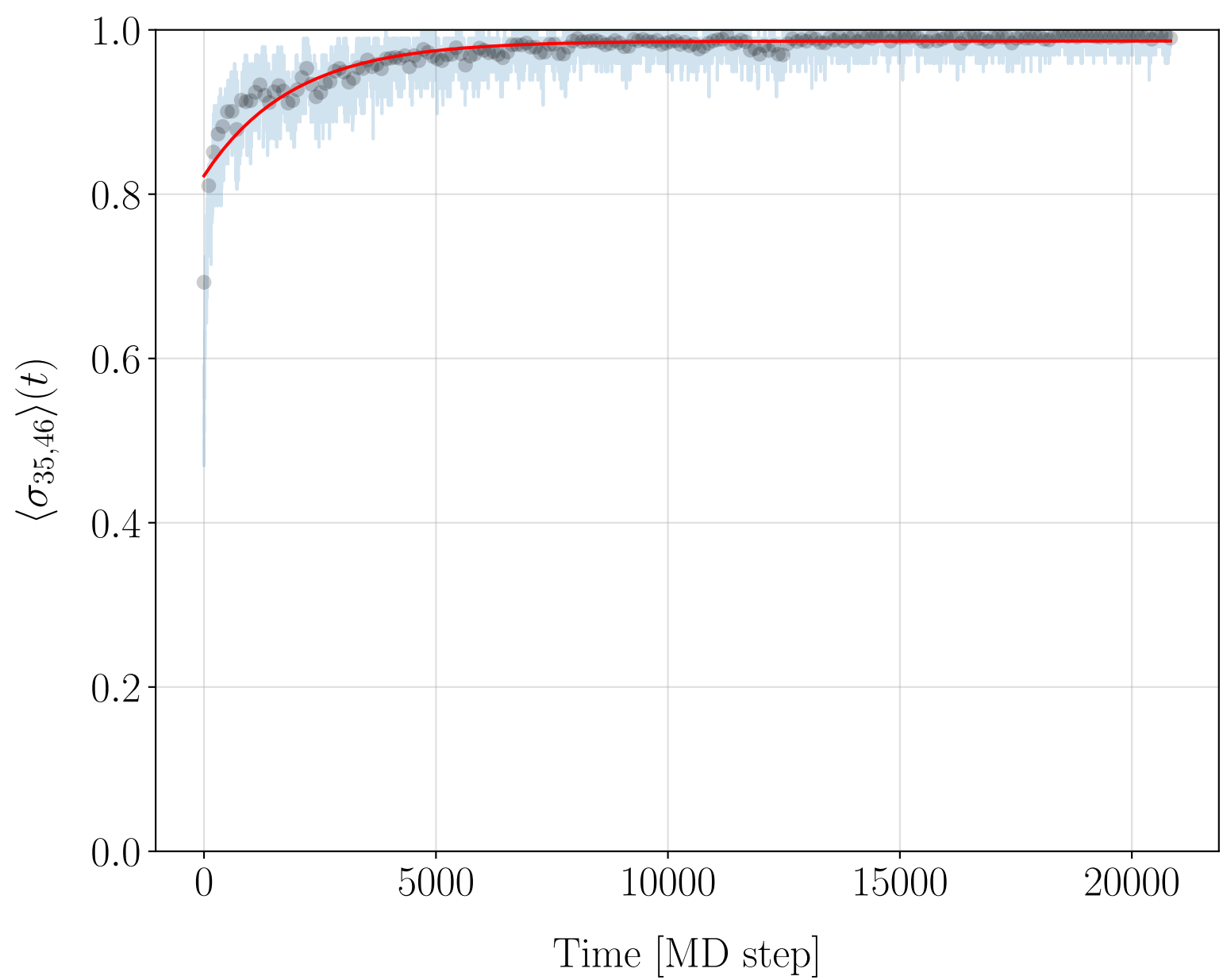

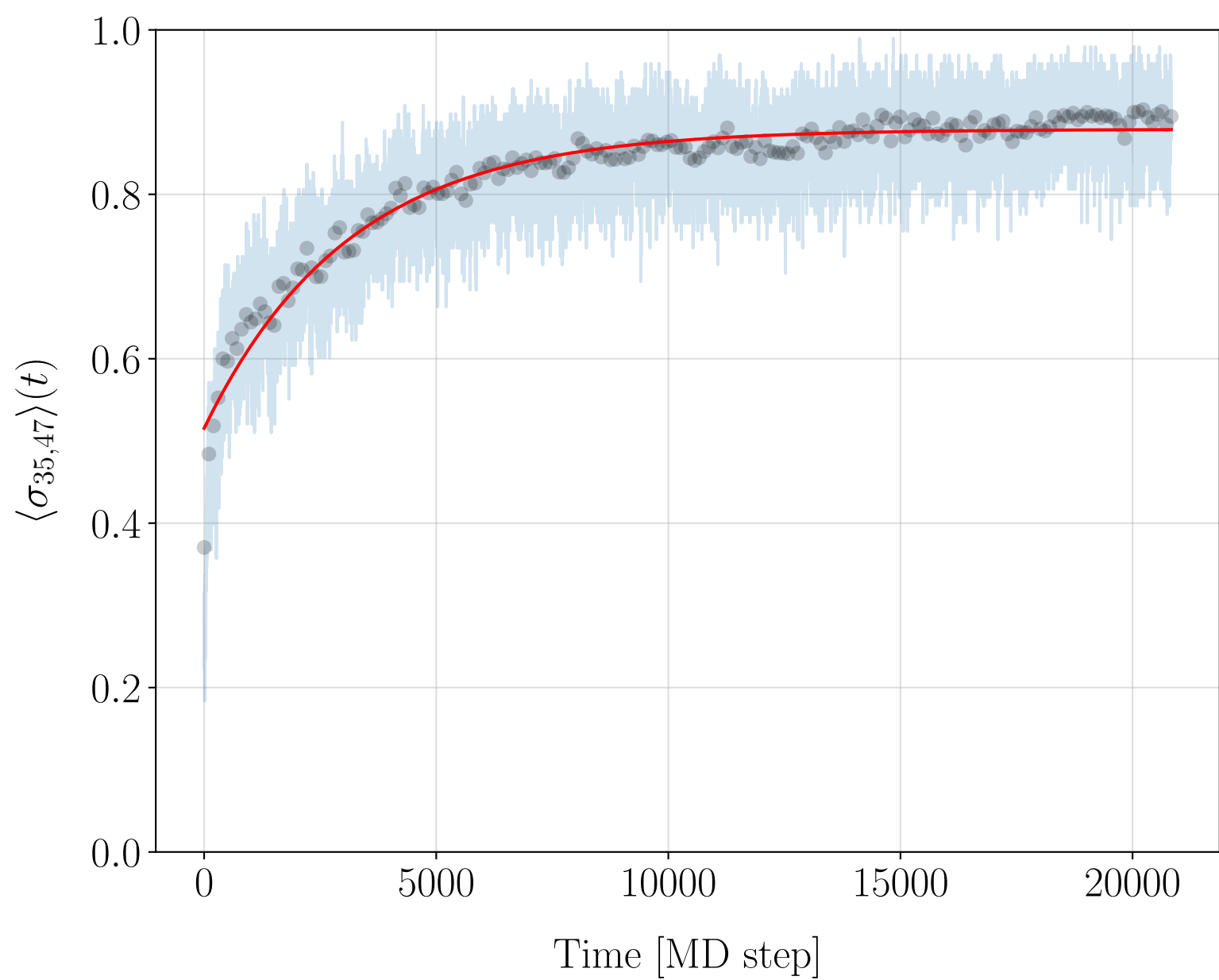

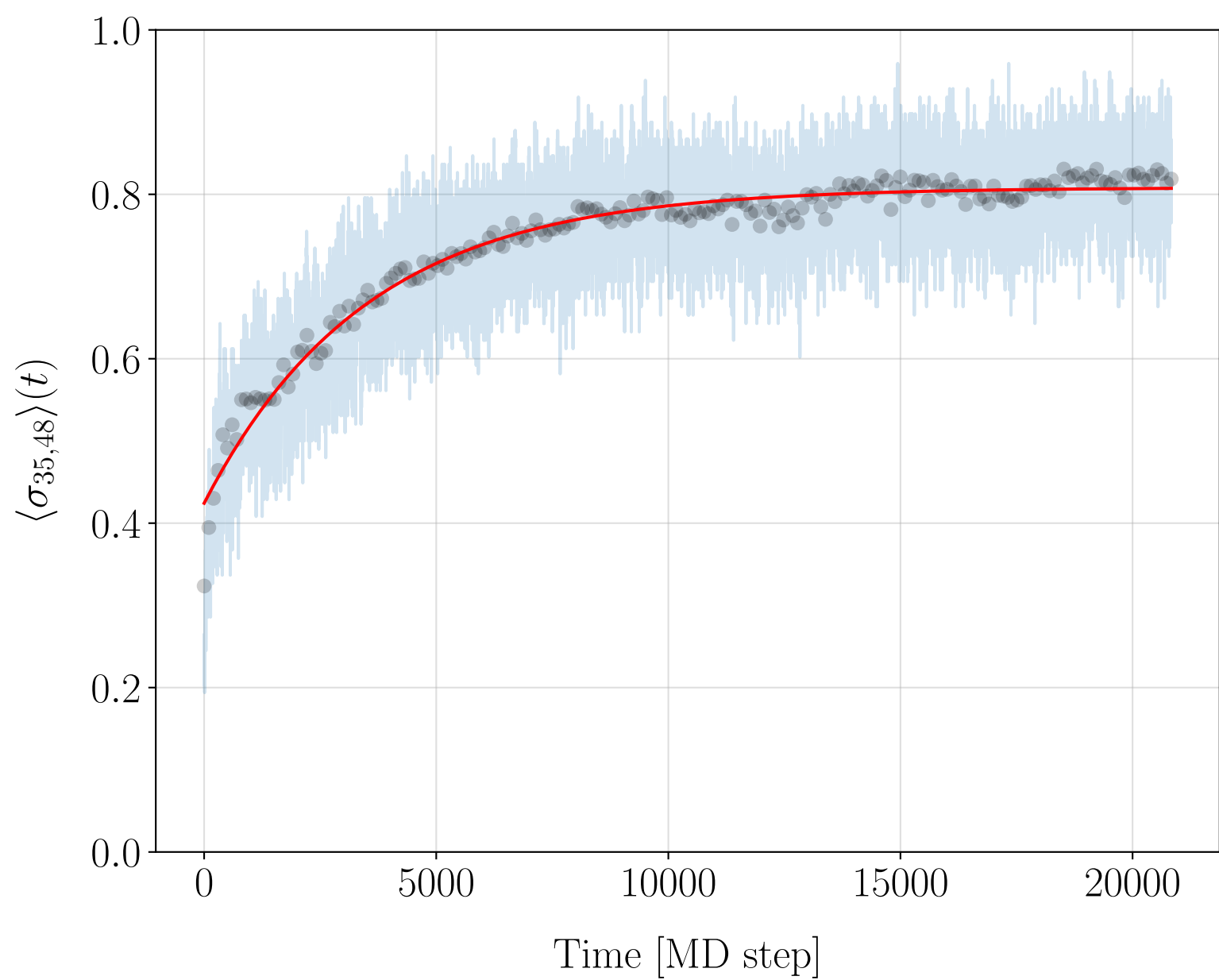

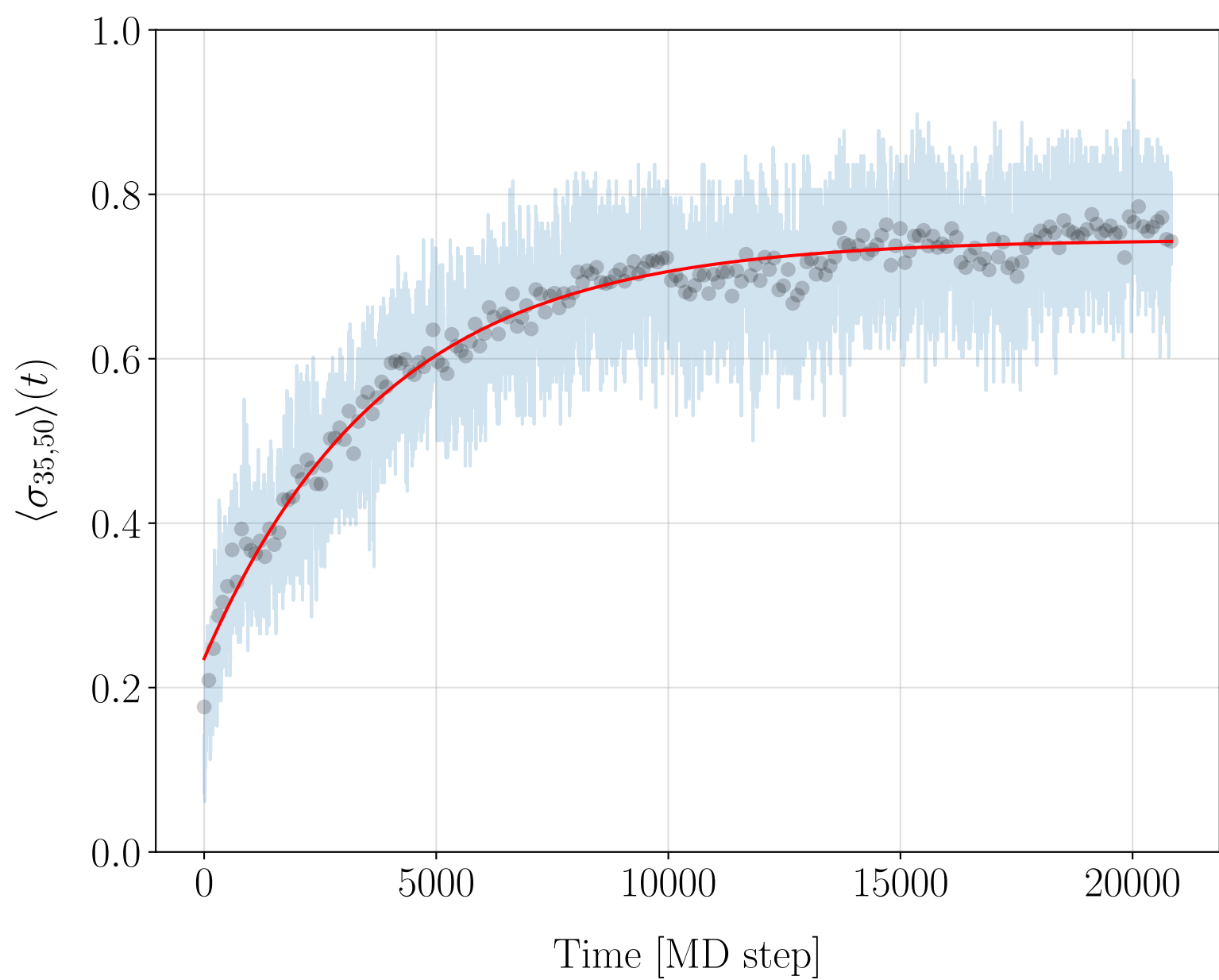

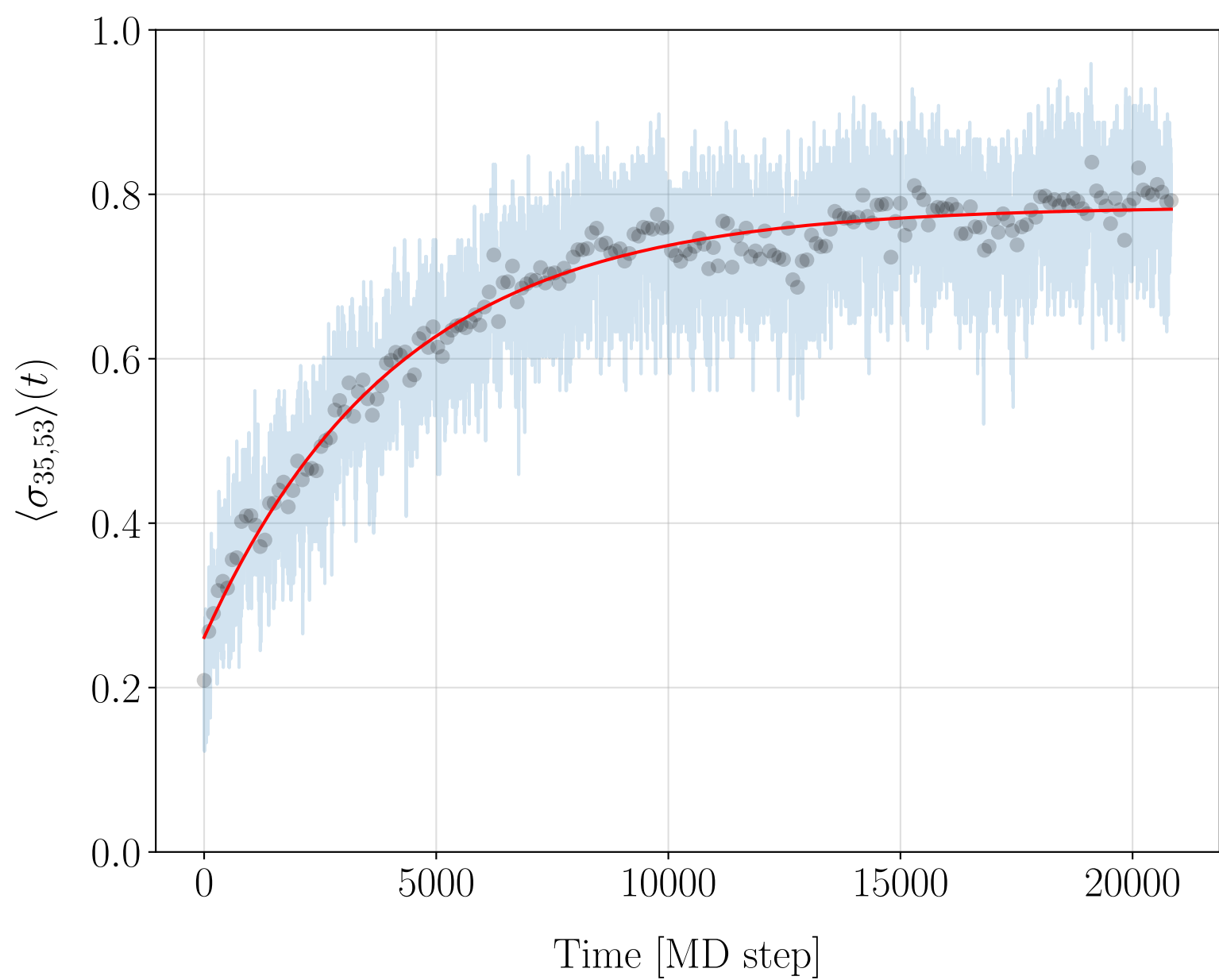

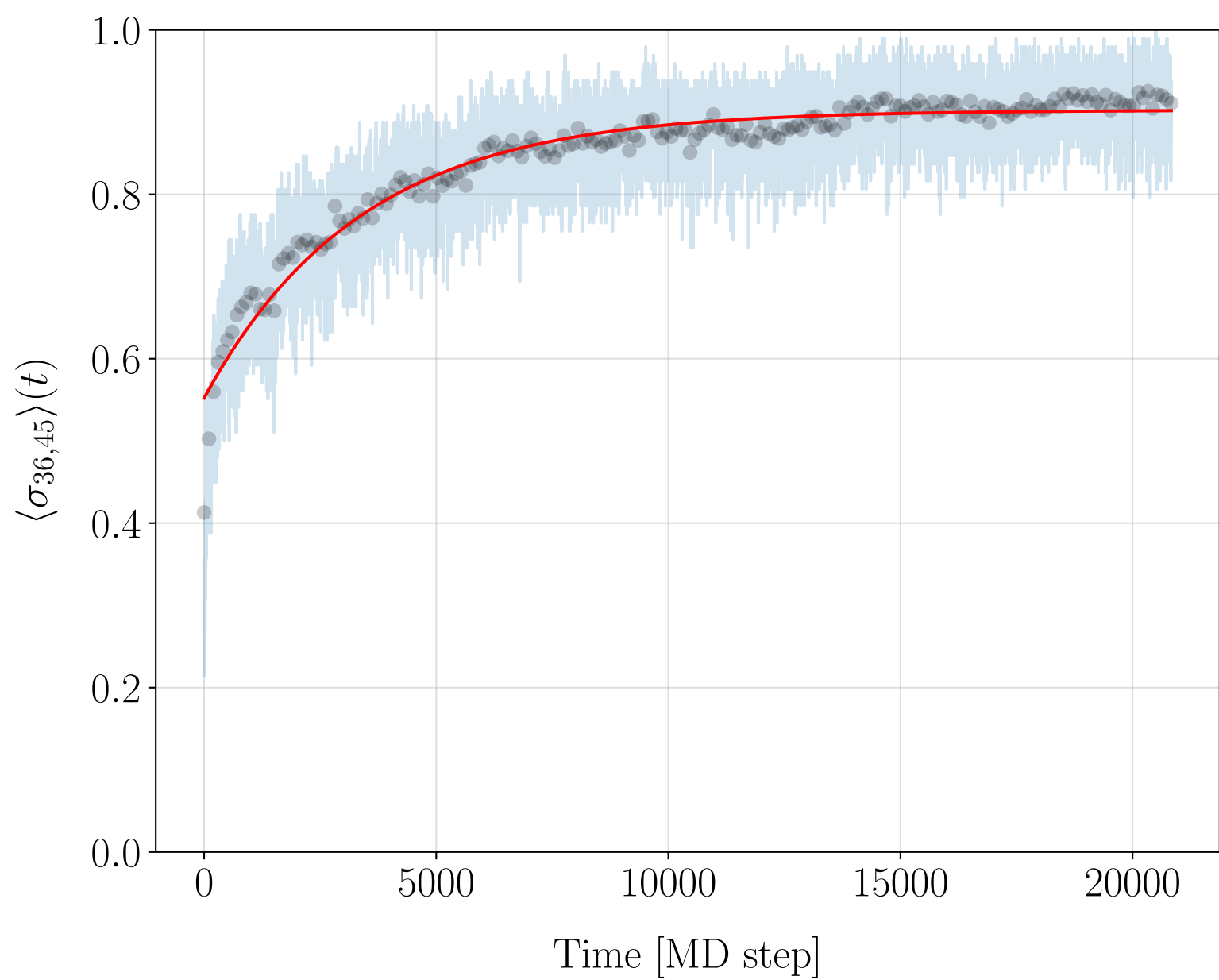

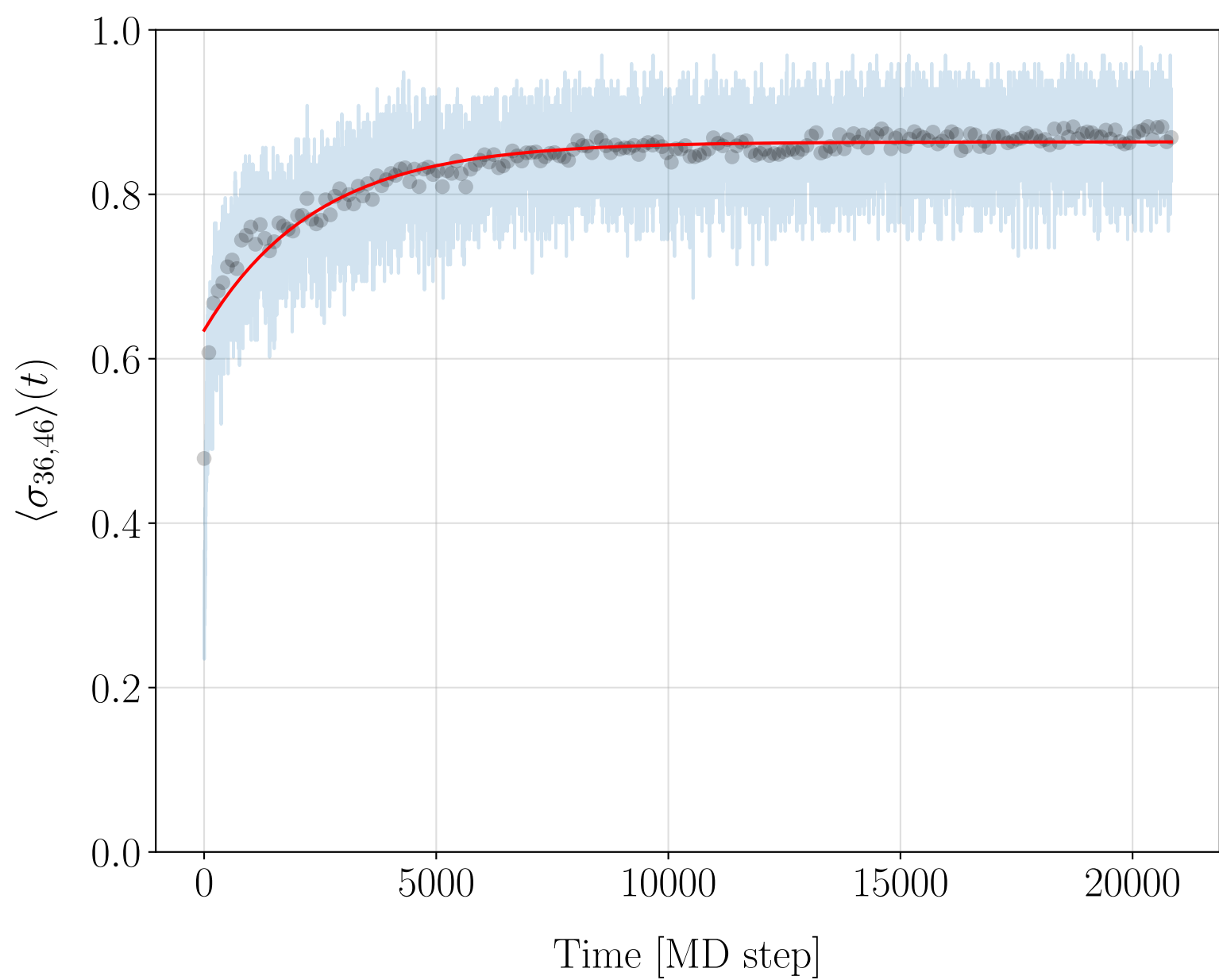

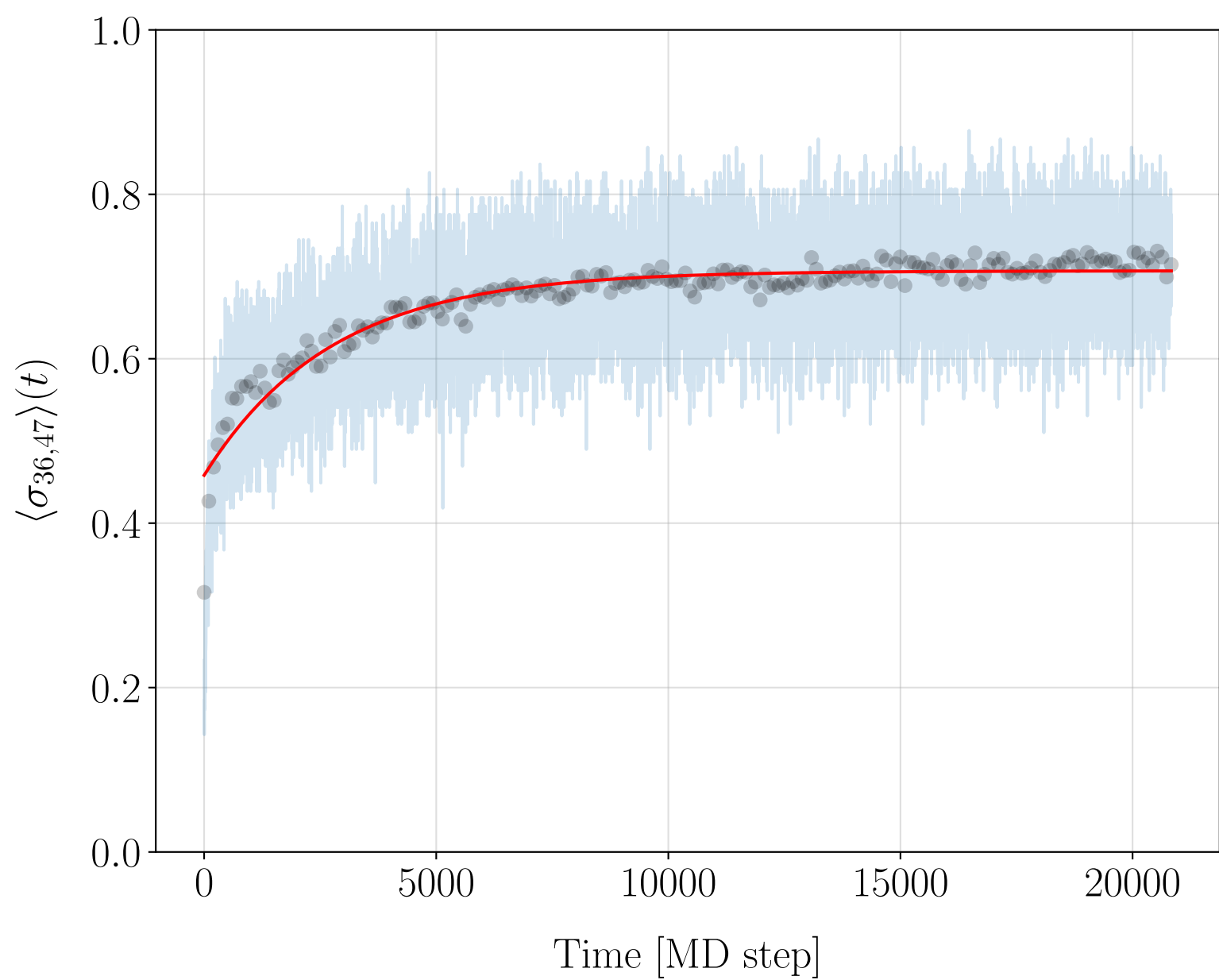

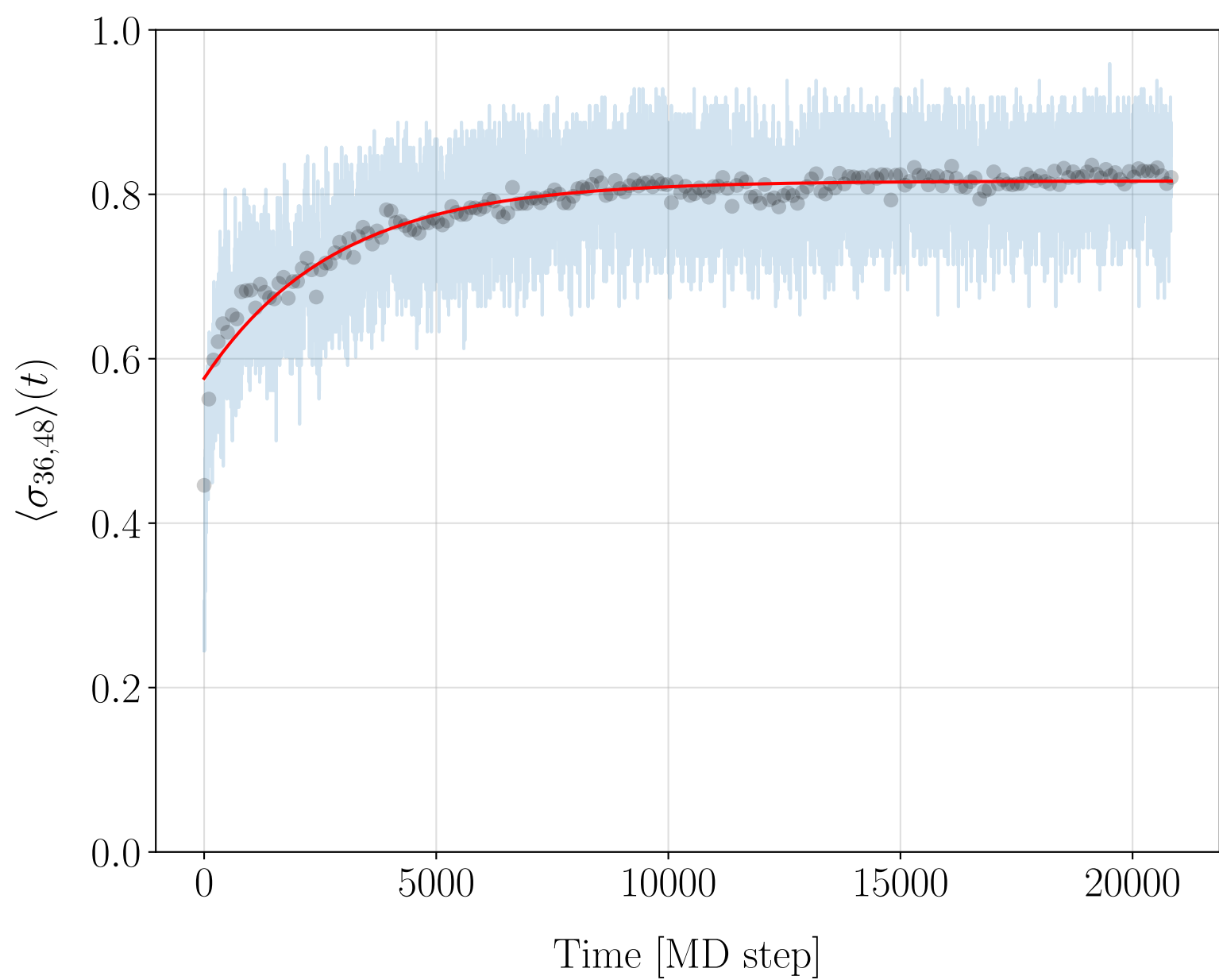

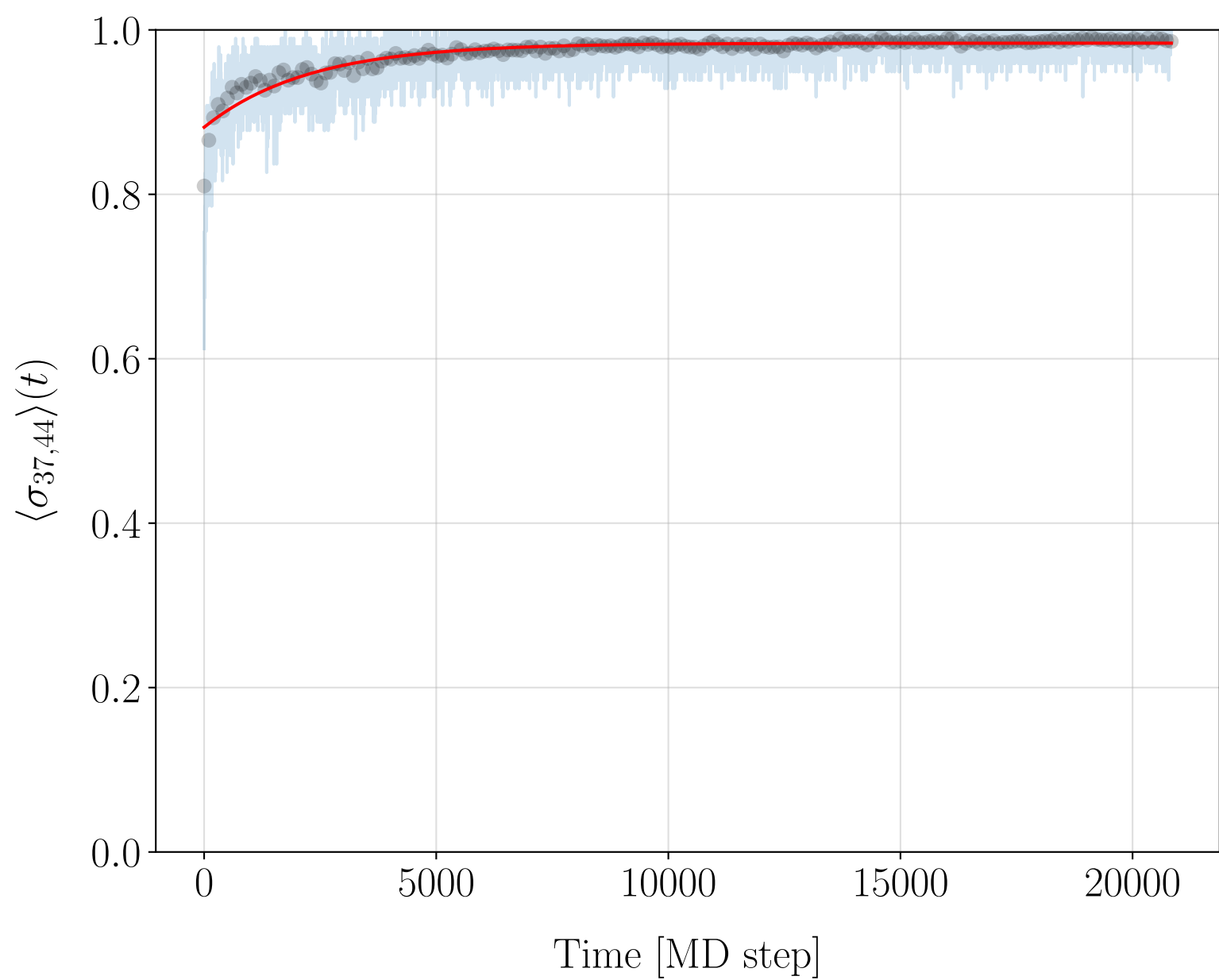

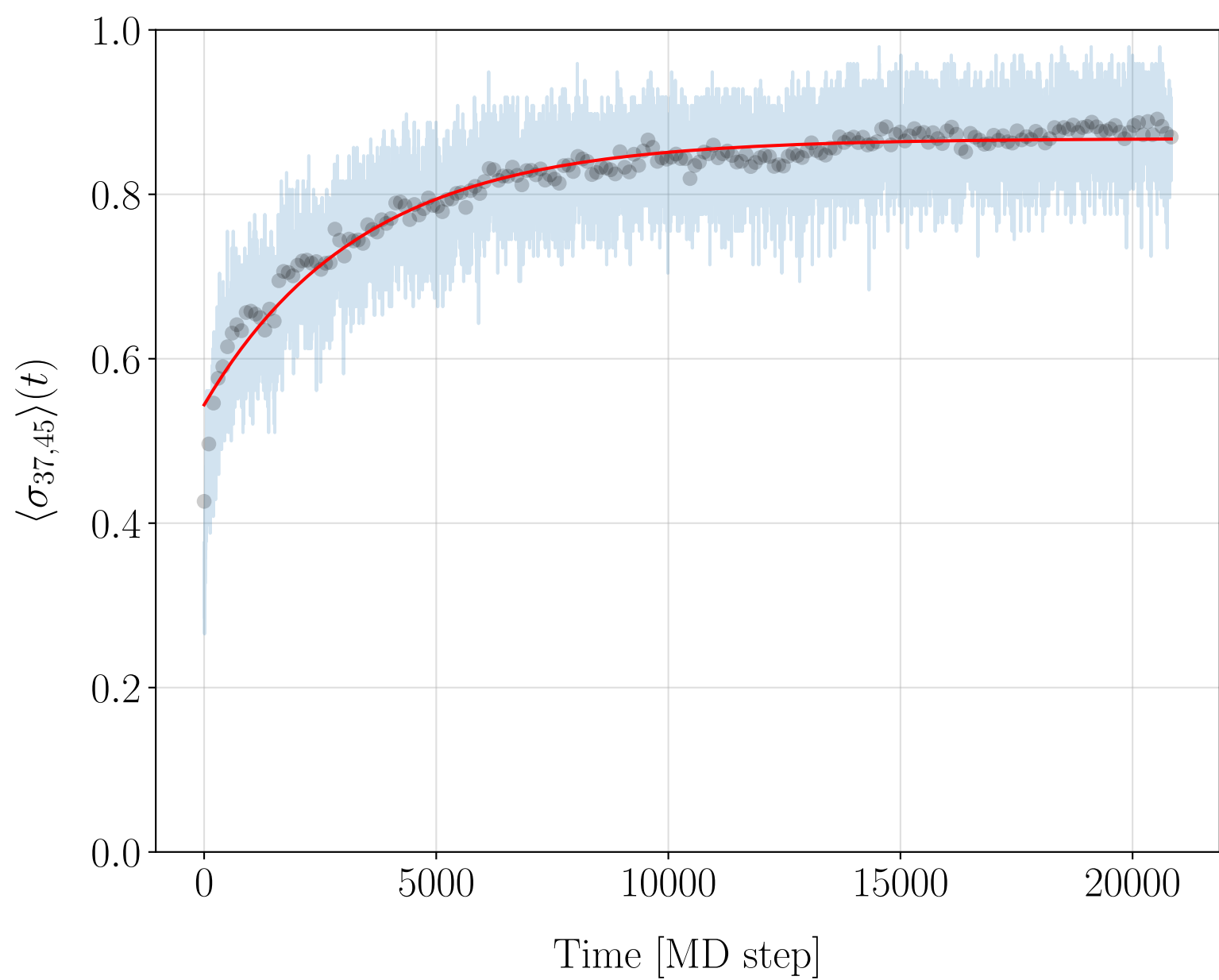

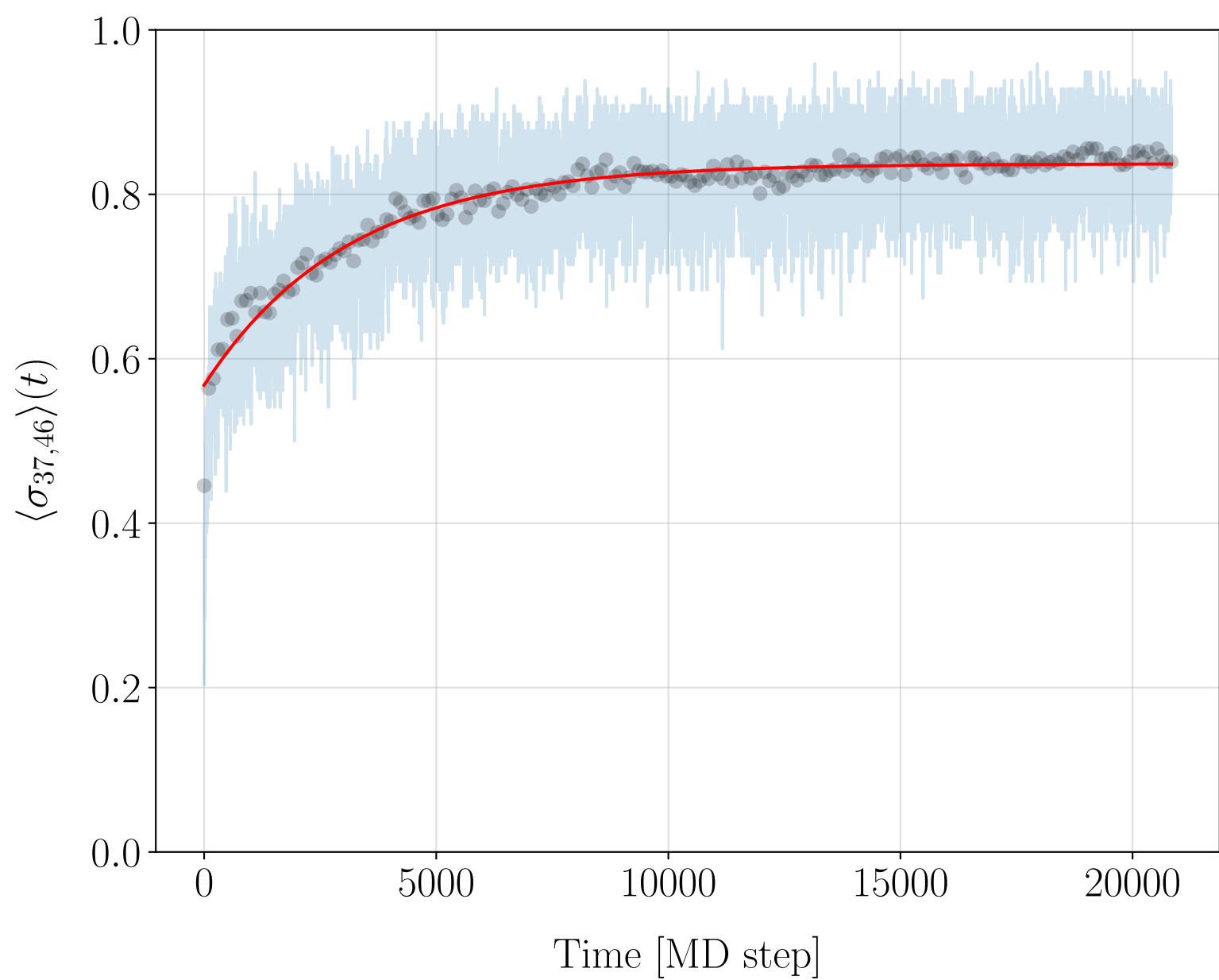

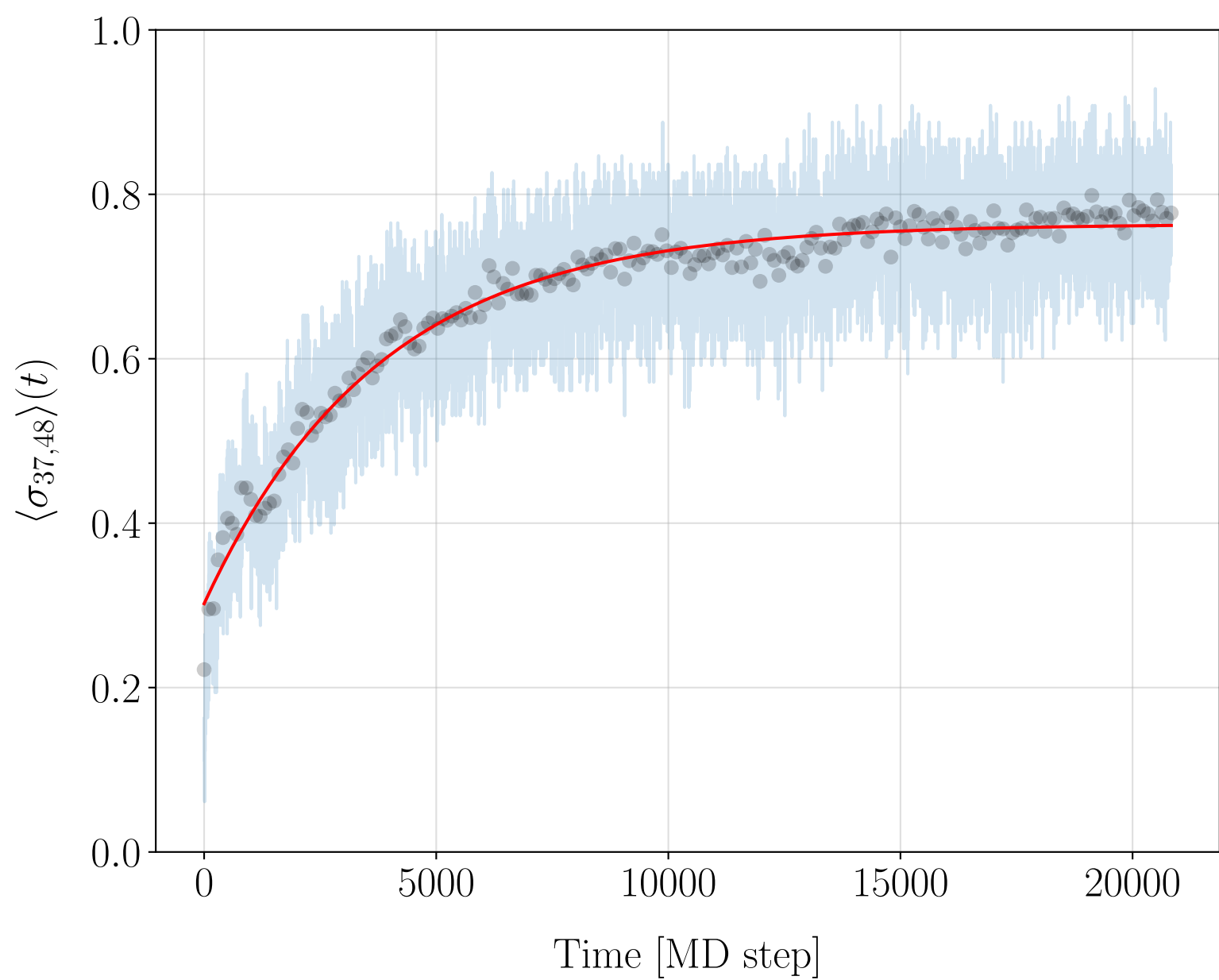

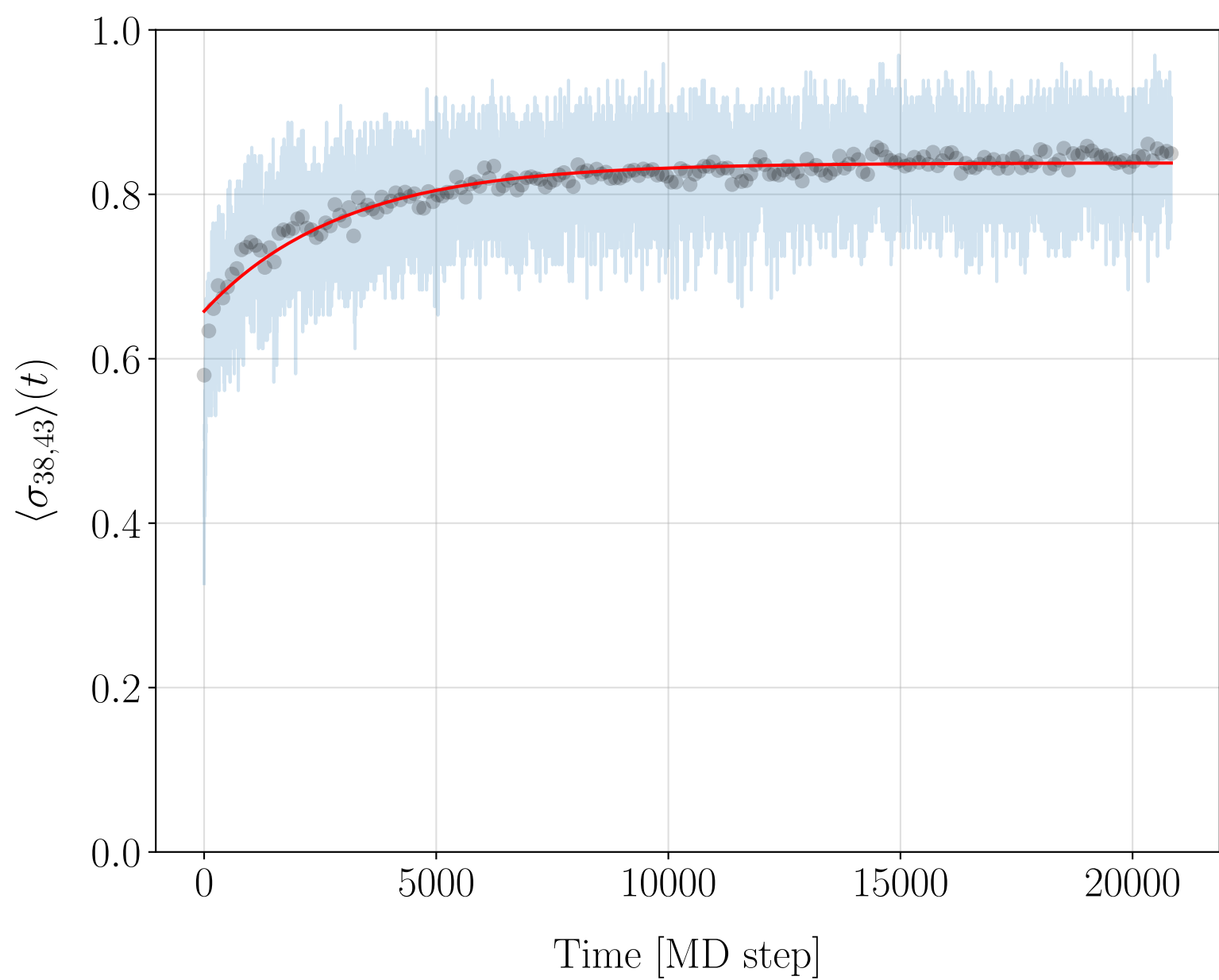

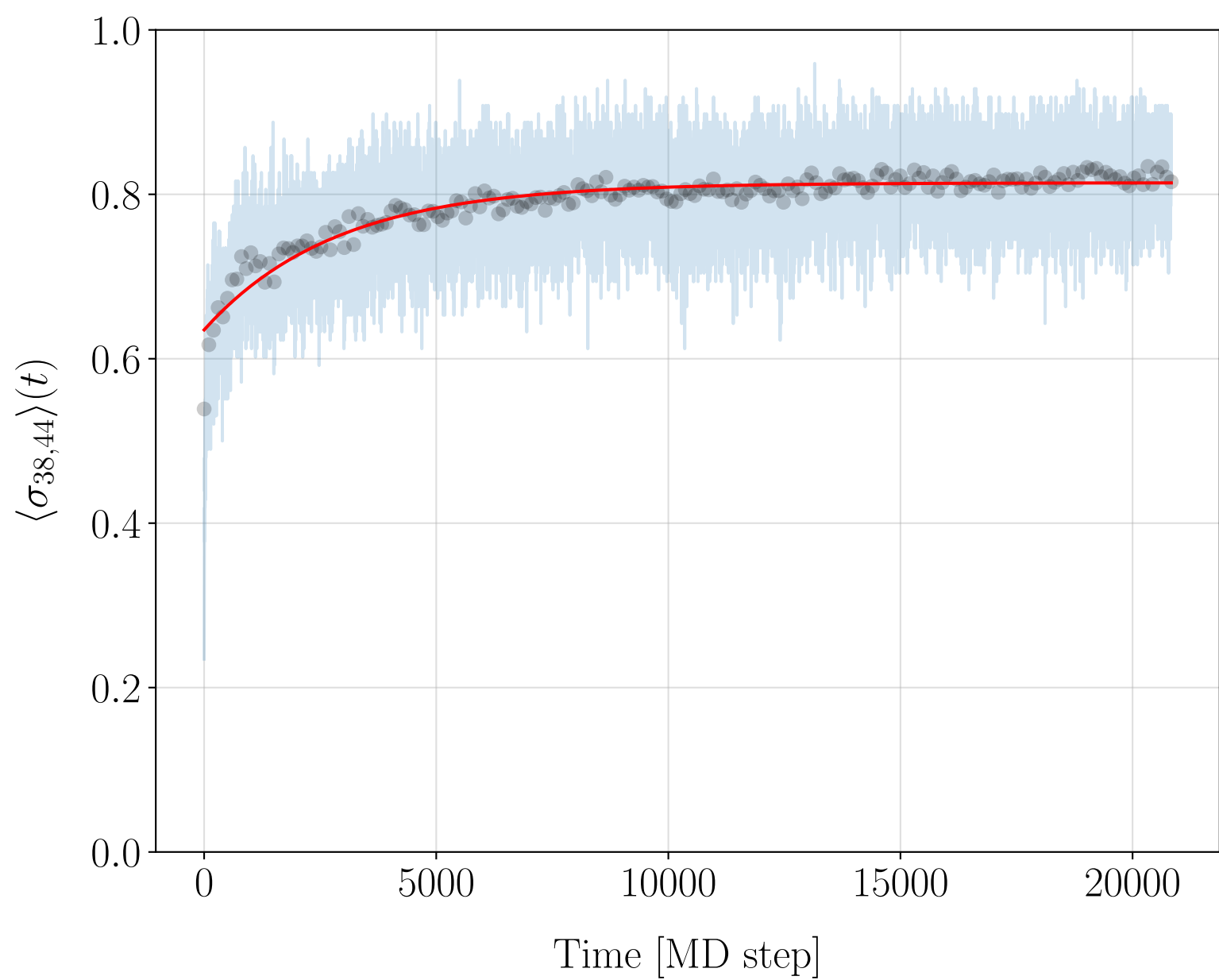

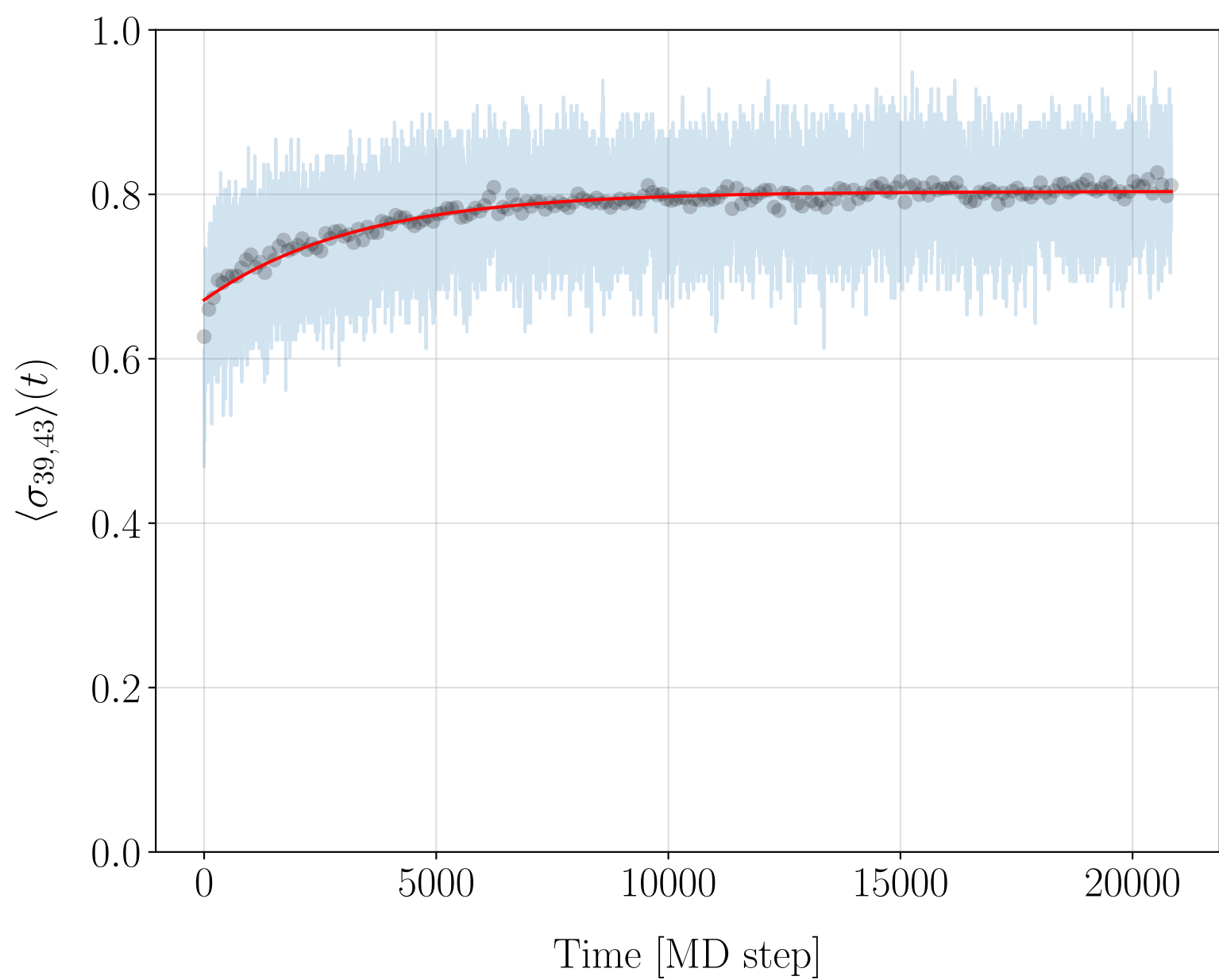

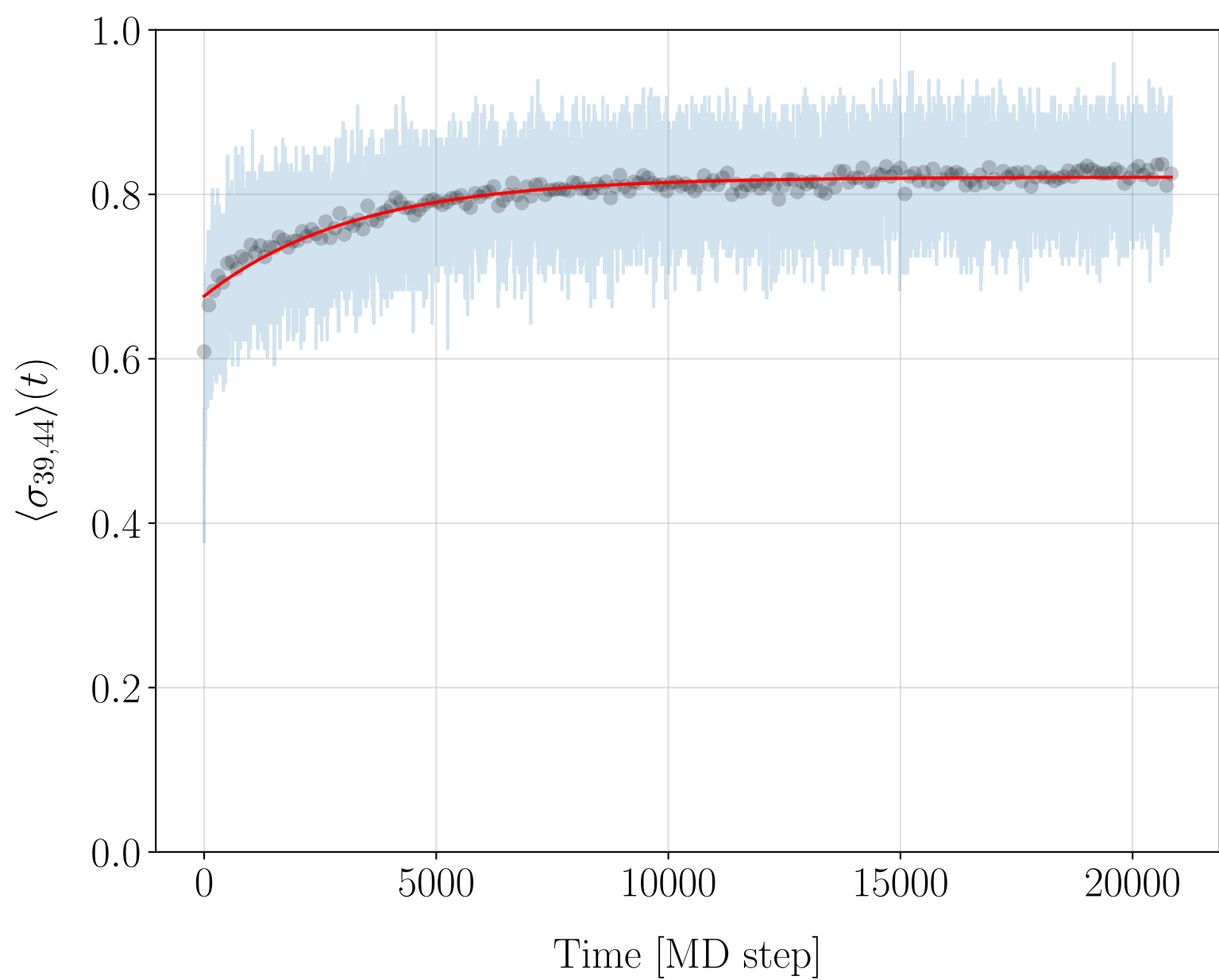

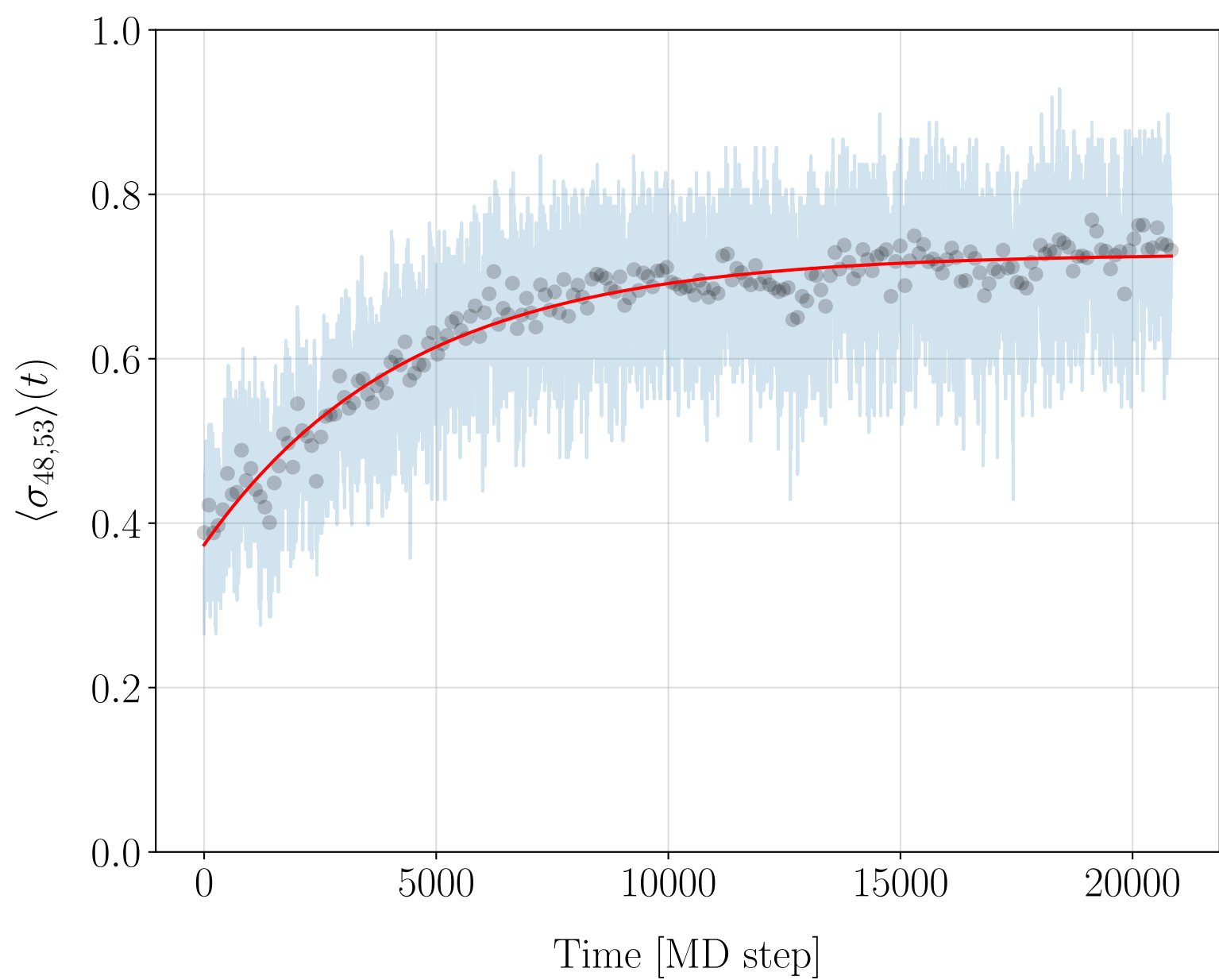

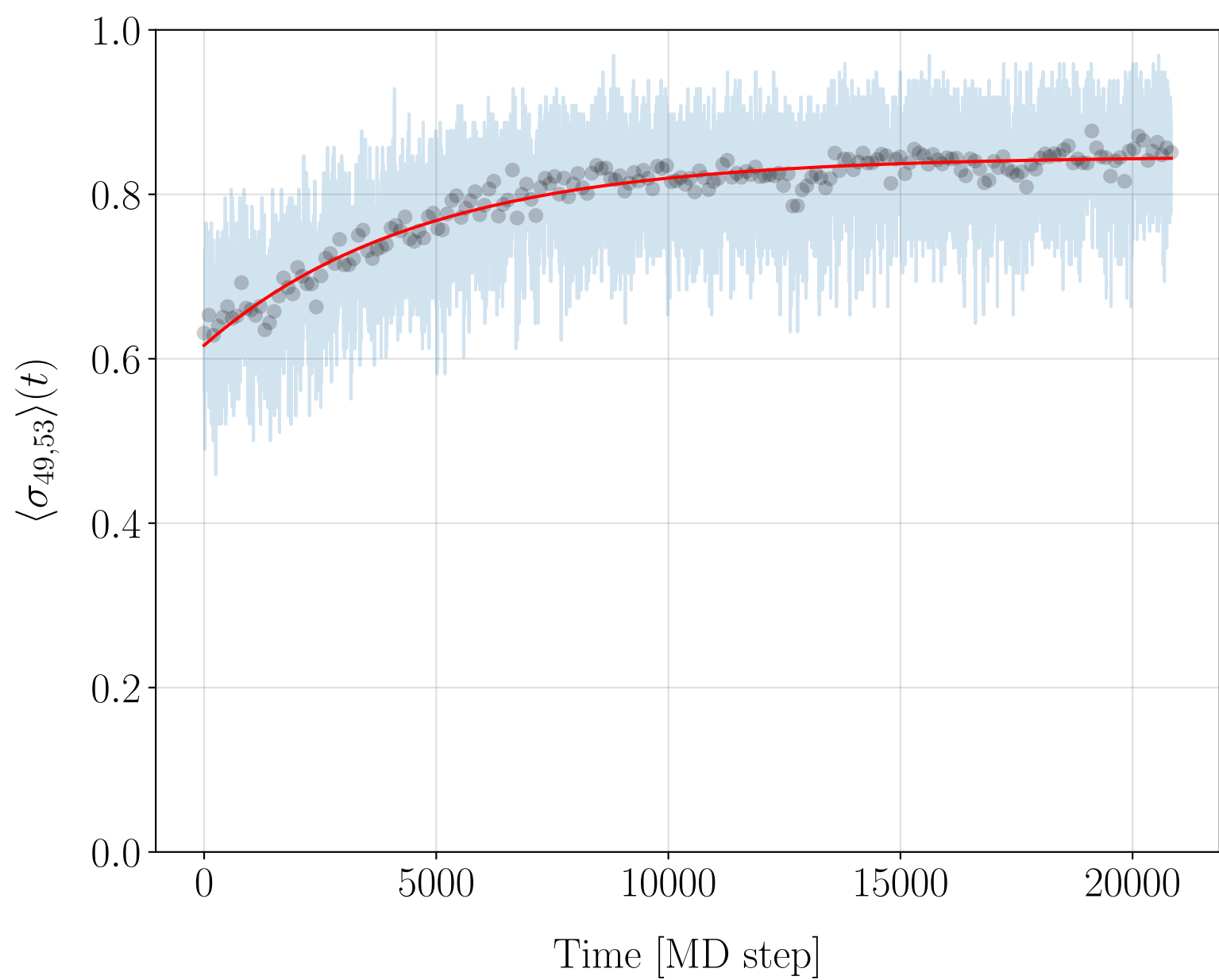

Supplement: S2 File — All 100 refolding trajectories at t = 0.9Tf are used to compute contact formation probabilities. All plots in this file are drawn as explained in the caption of Fig 7. (PDF) [file pcbi.1011107.s003.pdf]
