# Supplementary material for: Folding kinetics of an entangled protein
Source: PLoS Comput Biol. 2023 Nov 13;19(11):e1011107. doi: 10.1371/journal.pcbi.1011107 (PMC10681328; doi:10.1371/journal.pcbi.1011107)
Supplement: Attachment — Submitted filename: response_letter.pdf [file pcbi.1011107.s007.pdf]

Response letter for "Folding kinetics of an entangled protein"

Leonardo Salicari, Marco Baiesi, Enzo Orlandini, Antonio Trovato

We are writing in response to the valuable feedback provided by the reviewers for our submitted manuscript titled "Folding kinetics of an entangled protein". For clarity, we have organized our responses according to the individual reviewers' feedback, using their respective comments as headers. We warn both reviewers that the numbering of references in the track-changes version is not the correct one (e.g. the one found in the revised version). In order to address a comment by the second reviewer, we moved the place in the text where some references are first quoted. Because of this, such references are present twice in the track-changes reference list, one as removed from the position they had in the original manuscript, and one as added to the position they have in the revised manuscript.

Response to the first reviewer

We are grateful for the time and effort the reviewer has dedicated to evaluate our manuscript and for her/his fruitful suggestions. In this response, we have addressed each of the reviewer's comments comprehensively, outlining the revisions we have made to the manuscript. We believe our manuscript greatly improved, as a consequence of the revision process.

1. **Use of the term long-lived to describe the intermediate IT on the folding pathway of RD1. The authors may want to consider whether this is the most appropriate term (longer-lived may be better). This is a protein which both computationally and experimentally folds very fast. None of the intermediate states are very long-lived with respect to folding intermediates observed for many other proteins. Everything is relative, but this term may mislead some readers.**

   We agree that the term long-lived may mislead some readers and we changed it to longer-lived as suggested by the reviewer.

2. **It is stated that the intermediate IE speeds up folding but no evidence is given to support this statement. The whole debate on whether intermediates speed up folding by reducing the conformational search or whether they slow down folding (by acting as kinetic traps), or whether they are neutral in terms of folding speed but still obligate species that are populated on folding pathways has been debated for more than 40 years. I would simply remove this sentence or rephrase it. Or, if the authors feel that they really have evidence to make the statement, then they should indicate what this is.**

   We agree that our original phrasing was not appropriate and we removed the sentence. The speed up was meant to be relative to the threading pathway, not to the folding in the absence of any intermediate. Thanks to a comment by the other reviewer, we realized that, in the original manuscript, the way we were computing the folding time was not consistent with the way folding pathways were classified, due to a slightly different definition of the folded state in the two procedures. In the revised version, the definition of the folded state

is now the same (all definitions used to classify states/pathways are now provided in the Material and Methods section "Ensemble definition and pathway classification"); Table 1 (the average folding times) and Fig. S6 (folding time distributions) were revised accordingly. Those changes are indeed quite minor with respect to the original version.

3. **The paragraph linking folding pathway to function is completely speculative and unnecessary and could easily be removed.**

   We agree that the paragraph is speculative. Although we think that some speculations might be useful when discussing results, we removed the paragraph as suggested, with the additional aim of shortening the discussion section.

4. **The definitions of t^* and k aren't very clear. Further explicit description should be included, i.e., confusion over whether t^* was the time to first make the contact or the lifetime of the contact.**

   We are especially thankful to the reviewer for this observation since it led us to reconsider this whole part of the analysis. The main message is maintained, but the procedure for computing what we now term "contact folding rate" is simplified.

   As a general remark, we note that we had used the phrase "contact formation rate" for k for simplicity. Still, we were referring to the rate of the stochastic process by which the contact formation probability increases from its initial value, p_U, in the unfolded state to its final value, p_F, in the folded state. In general, $p_U > 0$ and $p_F < 1$ because contacts form and break in both states. We acknowledge the above phrasing is likely misleading and modified it to "contact folding rate". We also changed the terminology "contact/loop form" to "contact/loop fold" in the passages referring to the overall folding process. Moreover, the t^* in the logistic curve was meant to represent a "lag time" which has to elapse before "contact folding" kinetics (with characteristic time $1/k$) could take place.

   The real question is whether a lag time t^* is needed at all. We realized it is not since the increase in time of contact formation probability can be better fit by the simple exponential kinetics $A - B\exp(-kt)$, where $A = p_F$ is the saturation value of the contact formation probability in the folded state and $B = p_F - p_U$ is the gain of the former quantity in going from the initial unfolded state to the final folded state. With hindsight, the lag time t^* obtained in the previous analysis from the logistic fits correlates to p_U (the higher p_U, the smaller t^*). Moreover, k is this simple exponential kinetics' characteristic "contact folding rate".

   We revised all parts of the manuscript dealing with this analysis (Abstract, Results, Discussion, Materials and Methods). We added two supplementary files showing the exponential fits to the contact formation probability curves for all native contacts (one for SH3 and one for RD1). The revised Fig. 7 (Fig. 8 in the original version) now shows two examples of exponential fits for a trap-avoiding "red" contact and a first-entangling "magenta" one. The revised Fig. 8 (Fig. 9 in the original version) shows the new analysis results. The main message is maintained: all trap-avoiding contacts (red) fold at a significantly faster characteristic rate with respect to all entangled contacts (dark circles).

   p_U turns out to be the relevant parameter that rationalizes the emergence of the first-entangling "magenta" contacts. The revised Fig. 8 shows that these first-entangling contacts display a larger p_U value than almost all other entangled contacts (dark circles in the lower-left corner of Fig. 8), which generally have a very low p_U. The p_U values and the associated standard deviations, which turn out to be smaller than the marker symbols in the plot, are evaluated by sampling 1500 conformations from the equilibrium unfolded state at $T > T_f$,

with the same procedure used to select the initial conditions for the refolding trajectories.

The picture emerging from the revised study is obvious: any "trap-avoiding" contact does fold faster than any entangled contact along the fast folding pathway. On the other hand, provided trap-avoiding contacts are correctly folded, fluctuations already present in the unfolded state allow "early-entangling" contacts to generate the native entanglement.

5. **The authors discuss the diffuse nature of the folding transition state for RD1. They may be interested and want to reference a study by Zhang & Jackson on the 5-2 knotted UCH-L1 which provided experimental evidence for the diffusive nature of the transition state on both folding pathways.**

   We agree that the broad energy barrier which constitutes the transition state for RD1 at $T = T_f$, as shown is Fig. 3, is reminiscent of the experimental results reported by Zhang & Jackson (now inserted as Ref. [67]) for the knotted protein UCH-L1. We now mention this connection in the "The entangled RD1 protein exhibits a two-state behaviour at the folding transition" subsection.

6. **The authors relate the height of the energy barrier between denatured and native states as representing the cooperativity of folding, and therefore state that the folding of RD1 is more cooperative. This might be something which it is accepted in the computational field, but for experimentalists, the height of the barrier merely determines the rate at which the protein folds and doesn't inform on cooperativity (which is associated with other partially folded states that might be populated on the folding pathway). Further clarification of how the term cooperativity is being used is required.**

   We connect cooperativity to the height of the free energy barrier when we discuss the equilibrium free energy profiles at the folding transition temperature in Fig. 2 and Fig. 3. In this context, the free energy profile determines the sampling of the different states according to the Bolzmann distribution. Therefore, the higher the barrier, the less populated the partially folded states, and the more cooperative the folding. To make this connection explicit, we added two brief comments after mentioning Fig. S1 (SH3) and Fig. S5 (RD1) in the main text.

7. **The term "unnecessarily hard" is used to describe the folding of knotted and entangled proteins in the Introduction. Although several decades ago when they were first identified it was assumed that the folding of knotted/entangled proteins would be very challenging, there is now substantial evidence that this is not always the case – experimental results have shown that for shallow knotted proteins the knot may only slow the folding rate by 3-10 fold, and considerably less than single mutations which do not affect the topology of the protein. In addition, experimental and computational studies have also established that in a cellular environment chaperones can accelerate the folding of knotted proteins by at least one, if not two, orders of magnitude. General statements on how knotted/entangled proteins fold are frequently misleading as they do not illustrate the diversity of behaviours that has now been found for topologically complex systems.**

   We agree that quite diverse folding behaviours were actually observed for different knotted proteins. We revised the phrasing in the Introduction section pointed out by the reviewer (we were indeed referring to the earliest attitude in the field), adding a brief comment on how the folding of knotted proteins turned out to be "easier" than originally thought.

8. **In general, the discussion is far too focussed on comparison of the results with those from other computational studies apart from the reference to the experiments on RD1. There is almost no discussion of any other experimental studies looking at the folding of knotted/entangled systems. This is a short-coming of the paper. For example, there is a reference to a computational study demonstrating that knotted topology forms in the early stages of folding. First, this is just a single computational study and other computational studies have addressed this question. Second, there is experimental evidence for some knotted proteins that the knot forms relatively late on the folding pathway, and isn't present for example in intermediate states.**

   Again, we agree that quite diverse folding behaviours were observed for different knotted proteins. Also, we acknowledge that we were not discussing other experimental studies on knotted proteins. We added a sentence in the Introduction section, highlighting how experimental evidence is available both for cases in which the knotted topology is already present in the denatured state and for cases in which the knots forms relatively late along the folding pathway, as mentioned by the reviewer. Overall we inserted 4 different references to experimental studies of the folding of knotted proteins in the Introduction section [17,29-31]. We also added other references to computational studies addressing similar issues [46,72,74], and the related one of the relevance of non-native interactions [75], in both the Introduction and the Discussion section, while making the corresponding statements less general. We also added a sentence in the Introduction section to mention a recent computational study [50], which highlights how different metastable states were found to be populated, within a similar scenario of folding heterogeneity, also in the case of a multiply connected multidomain protein with interwoven chain topology.

9. **It would be most informative if some of the results could be related back to the sequence/structure of RD1, e.g., three contacts key for the fast folding track - it would be very interesting to know what these residues were in the protein. I assume that all native contacts are weighted the same by using a Go model. In reality, these three residues might form stronger contacts than average and may mean that a greater proportion of molecules fold via the fast track. One might expect these three residues to be more highly conserved than others – a quick sequence alignment study would demonstrate whether this is the case or not. (a note of caution about this though - for fast folding small non-entangled proteins there is only evidence that residues are conserved for overall stability not for folding rate, i.e, the residues involved in forming a critical folding nucleus are no more conserved than for those conserved for stability.**

   We agree that more information on the residue/interactions that we find to be crucial in folding kinetics should be given. All native contacts, including those labeled "trap-avoiding" and "first-entangling" are now listed in table S1 on the Supplementary material. We had in fact already reported about the fact that the N-terminal S4-A7 residues and several residues in the V26-E35 stretch are also highly conserved across different isoforms (now Ref. [77]), although they do not participate into the ice-binding surface, consistently with the crucial role their interactions play in the folding mechanisms highlighted by our simulations. We now specify that such contacts are involved in "trap-avoiding" interactions and that the residue I37, which is involved in 2 of the 3 "first-entangling" contacts without being part of the ice-binding surface, is found to be conserved as well [77]. We also added a cautionary statement on the likely role of overall stability in residue conservation, as rightly pointed out by the reviewer, along with a corresponding reference [82]. The weights of the energy terms

are chosen to be uniform across all residues; this information was given in the Materials and Methods section and we now added it in the Go-like Energy Function subsection as well.

Response to the second reviewer

We are grateful for the time and effort the reviewer has dedicated to evaluate our manuscript and for her/his fruitful suggestions. In this response, we have addressed each of the reviewer's comments comprehensively, outlining the revisions we have made to the manuscript. We believe our manuscript greatly improved, as a consequence of the revision process.

1. **Figure 1: Please add the contact maps for the native structures of RD1 and SH3 for direct comparison to Fig 7. Clearly define what are considered "entanglement contacts."**

   The contact maps for SH3 and RD1 are already shown in Figure 2 and 7, respectively. Although the color code is not simply b/w, since it refers to the contact formation probability in the transition state (SHR) / intermediate state (RD1) ensemble, we think it would be redundant to add essentially the same plot in another figure. Since we are not dealing with pairs of closed curves, any choice of a strict threshold on $|G'|$ to define entangled contacts would be somewhat arbitrary. This is one reason why we chose a smooth activation function $h(|G'|)$ (see Fig. 1 and Eq. (10)), with threshold g_0, as the weight to be used to compute the entanglement reaction coordinate $\langle G' \rangle$. In keeping with this choice, $h(G')$ could be interpreted as the probability to be entangled for a contact with Gaussian entanglement G', so that contacts with $|G'| > g_0 = 0.5$ are likely, yet not necessarily, entangled. We briefly extended a sentence in the "Topological properties of the SH3 domain and the RD1 protein" subsection (Results) to emphasize this point. In practice, for RD1 there are 30 native contacts with $|G'| > 0.75$ (with NO contacts having $0.5 < |G'| < 0.75$, see Fig. 1c), that we classify as entangled. This practical definition is the one referred to in Figure 7 caption and in the result section "Entangled contacts form at the later stages along the fast folding channel of the RD1 protein". Similarly, the only native contact in SH3 with $|G'| = 0.63 > g_0$ is not considered entangled, since its Gaussian entanglement is just slightly above threshold, with no other native contacts above g_0 in SH3. However, the choice $g_0 = 0.5$ is crucial for a useful definition of the reaction coordinate (see point below). For clarity, we added a supplementary table that provides a list of the RD1 contact map. This table also specifies which contacts are classified into entangled, first-entangling, and trap-avoiding.

2. **A previous paper used the maximum Gaussian entanglement to describe conformations. Why should one switch to the average? What are the pros and cons? Also, since there is a distribution of G', can the average represent the distribution? For example, what if there are both positive and negative G' in the distribution, which leads to an average of close to 0, but this is different from having a distribution centered around 0?**

   One reason for defining the entanglement reaction coordinate as a weighted average is the need to be not overly sensitive to changes in the state of a single contact, which could be caused by small scale fluctuations. Single contact formation/breaking events, for example, might lead to abrupt changes in the maximum Gaussian entanglement indicator, which are not related to large scale rearrangements of the whole chain. Moreover, due to the use of a smooth activation function, the weighted average indicator increases if the number of likely entangled contacts with $|G'| > g_0$ increases, as it happens with RD1 in comparison to the SH3 domain (see point above). We inserted two sentences along the above lines in the "Gaussian entanglement" subsection (Materials and Methods). Ultimately, however, our

choice for the entanglement reaction coordinate is supported by the independent analysis of the contact formation probability curves, in which "trap-avoiding" and "first-entangling" contacts identified using the reaction coordinate have a direct interpretation in the p_U vs k plot (Figure 8 in the revised version). The weigthed average or the maximum indicator obviously do not convey all the information present in the G' distribution. Additional/different reaction coordinates would be needed (e.g. the second moment of the distribution) in case the average can not describe the folding kinetics in enough details. We agree this would be the case, in the event of different entangled motifs coexisting in the same configuration with opposite chiralities, as mentioned by the reviewer. However, this is highly unlikely to happen for protein chains as long as $60 \div 65$ residues as the one studied here. We extended a sentence in the Discussion section highlighting the need of additional reaction coordinate in the case of multiple windings/motifs.

3. **It's mentioned two minor adjustments were made regarding how the Go-like model was constructed (as compared to reference 9.) Why were these adjustments necessary or better?**

   In the last couple of decades, many different flavors of structure-based, coarse-grained models were used in the field. The choice as a benchmark of the model by Clementi et al. (Ref. [9]), indeed one of the most popular ones, is in itself arbitrary. For example, in the context of knotted proteins, Wallin and coworkers (now added in the revised version as Ref. [75]) implemented a model identical to our except for the exponent in the attractive Lennard-Jones potential; similarly, in describing the folding of the SH3 domain, Liu et al (now added in the revised version as Ref. [66]) implement a model with a finer coarse-graining, where each residue is represented by two interaction centers. The two differences introduced in our work with respect to [9] are as follows. Firstly, the native attractive interaction is modeled with a 12/6 Lennard-Jones potential instead of the 12/10 implemented in [9]. Fig. 2 shows how the SH3 transition state ensemble (TSE) structure is practically not sensitive to this change. We use the 12/6 to gain computational efficiency, thanks both to the ease in evaluating the 12/6 term and to the optimized LAMMPS implementation of the potential. Secondly, for the identification of native contacts we chose to follow the definition already used in our previous works [37-41]. In this way we could compare to the Gaussian entanglement computations already performed in those works for RD1. However, this contact map definition was used in many other works, e.g. in [75]. Both these aspects were already mentioned in the "Model benchmark and validation" (Results) and in the "Structure-based energy function" (Materials and Methods) subsections, respectively. We extended briefly both points along the above lines, adding references [66] and [75].

4. **I saw only one folding event and one unfolding event in Fig S5. How can one determine the populations of the folded and unfolded states faithfully?**

   We agree that, on average, there are few transitions for each equilibrium trajectory for RD1. However, the estimates of equilibrium populations, shown in the free energy landscape in Figure 3, are the result of combining all 100 equilibrium trajectories through the WHAM procedure, which outputs the density of states with respect to the fraction of native contacts Q. WHAM offers a practical tool to test the relative error of the population density (following the main text notation):

$$\frac{\delta W(Q)}{W(Q)} = \left[\sum_{i=1}^{R} N_i(R)\right]^{-\frac{1}{2}}$$

   We checked that these errors are below $2 - 3\%$ in the equilibrium unfolded and folded ensembles.

5. **Equilibrium simulations were performed at Tf, and refolding simulations at 0.9*Tf. Why 0.9? What happens for refolding at 0.8*Tf, 0.7*Tf, etc.?**

   In equilibrium simulations, we perform both MD at different temperatures around T_f to estimate it (through specific heat profiles for both proteins, shown in S2 Fig.) and MD at $T = T_f$ to compute free energy profiles, exploiting the WHAM procedure for both analysis. This aspect is pointed out by the first reviewer as one of the positive qualities of the work. The temperature of refolding simulations was chosen to mimic physiological/room temperature conditions. If we assume that the folding temperature is $\simeq 333°$ K (60° C), then 90% of this temperature is $\simeq 300°$K (27° C). A cautionary note is warranted as the relationship between temperatures coarse-grained implicit water model and real temperatures is not trivial. Therefore, the rationale is to use the same ratio of the refolding temperature over T_f to compare the behaviour of RD1 and SHR in similar refolding conditions. On the other hand, for too low refolding temperatures, set below the glass transition, one expects much higher kinetic barriers in the free energy landscape, with several metastable local minima. We observe that other computational studies employ refolding temperatures for similar structure-based models even closer to the folding temperature. Examples can be found in references [57] and [75]. We added related brief comments along the above lines in "The two-state behaviour of the non-entangled SH3 domain does not depend on the entanglement indicator and does not change in the refolding kinetics" and "The entangled RD1 protein exhibits a longer-lived non-entangled intermediate which acts as a kinetic trap when refolding below the folding transition temperature" sections.

6. **In the introduction, it seems quite some papers are from the same group. The authors should make it clear for clarity and transparency.**

   We guess the reviewer is referring to papers from our group. We grouped them the first time are cited (now [37-41]), as suggested by the reviewer, mentioning explicitely that the references are to papers by some of us. In order to group those references together we had to mention explicitly that the Gaussian entanglement was originally introduced to describe entangled dimers. We also added a reference to the most recent paper by some of us (Ref. [41]), where entangled loops are found to be abundant in transmembrane proteins as well.

7. **What is the smallest entangled peptide? Is this peptide amenable to all-atom MD?**

   To answer this, we refer to the table compiled by some us in [39] (link to data repo: `https://researchdata.cab.unipd.it/123/`, file: "G1_Lk_prot.txt"). The smallest peptide with maximum Gaussian entanglement satisfying $|G'| > 1$ is the Human defensin beta 4A (PDB reference code: 1fd3, chain A) with length of 41 residues and $G' = -1.06$. Instead, RD1 is the smallest protein with $|G'| > 1$ and without disulfide bonds. Both are amenable for all-atom MD, nonetheless we chose to focus on coarse-grained models for two reasons. Firstly, as a follow-up project, longer than 100 residues polypeptide chains will be considered, therefore posing a challenge to the computational resources needed to perform MD on such large systems. Secondly, the computational efficiency of a coarse-grained model allow us to perform numerous equilibrium and refolding simulations to acquire appropriate statistics.

8. **How did the authors determine which pathways were observed (Table 1)? Was it by manual visual inspection, change point detection, or?**

We especially thank the reviewer for pointing out this aspect, which was not present in the original manuscript. We have taken steps to clarify it by explaining the procedure in the Material and Methods section "Ensemble definition and pathway classification". To summarize it, ensembles are approximately defined through rhomboidal regions (in the main text we specify the Q and $\langle G' \rangle$ regions, visualizing those of IE_- and IT in Figure S7). Then, trajectories are classified considering a rolling average (temporal window of 555 MD Steps) of the reaction coordinates and, subsequently, testing the passage of the trajectories through the ensembles. "Fast folding" trajectories refold from U to F without passing through IT. On the other hand, if the trajectories include IT states, they are classified using the last states before F, IT for "threading" and U for "backtracking". Thanks to this comment, we realized that, in the original manuscript, the way we were computing the folding time was not consistent with the way pathways were classified, due to a slightly different definition of the folded state. In the revised version, the definition of the folded state is now consistent (being the one described above); Table 1 (the average folding times) and Fig. S6 (folding time distributions) were revised accordingly. Those changes are indeed quite minor with respect to the original version.

9. **Overall, I found the movies the most helpful for understanding the paper. I wish the authors could find a way to show the conformations and steps on paper. I suggest the authors include representative structures above different segments of Fig 4. I suggest the authors combine Fig 5 and 6 (putting the structures next to each basin). Also, the entangled part (colored red and blue) of the IE- conformation should be aligned to that of the F. IE+ and IE- should be visualized so that one can see they are mirror images. A label of "threading" can be added next to the IT F arrow; a label of "direct" can be added next to the U IE- arrow.**

   We implemented the reviewer's suggestions in Figure 4, highlighting the different states using the letter associated with these. The 3D "ball and stick" representations (Figure 6 in the previous version of the article) are now incorporated within Figure 5, as suggested. The IE_- 3D structure image has been rotated to better align with the F state and compare with IE_+. Pathways labels were added too in Figure 5.

10. **Fig 9: Very difficult to tell which circles are magenta and which are red.**

    We agree with the reviewer on this point. Therefore, in revised Fig. 8 (Fig. 9 in the original manuscript), we increased the thickness of the circles highlighting first-entangled and trap-avoiding contacts to help the viewer distinguish them. Moreover, a comment by the other reviewer led us to reconsider this whole part of the analysis. As a general remark, we note that we had used for simplicity the phrase "contact formation rate" for k in the original version, but we were in fact referring to the rate of the stochastic process by which the contact formation probability increases from its initial value, p_U, in the unfolded state, to its final value, p_F, in the folded state. In general, $p_U > 0$ and $p_F < 1$, so that contacts are expected to repeatedly form and break in both states. We acknowledge the above phrasing is likely misleading and we modified it to "contact folding rate". We also modified the phrasing "contact/loop form" to "contact/loop fold" whenever the overall folding process is referred to. Moreover, the t^* in the logistic curve was meant to represent a "lag time" which has to elapse before "contact folding" kinetics (with characteristic time $1/k$) could take place. The real question is whether a lag time is needed at all. In fact, we realized it is not, since the increase in time of contact formation probability can be better fit by the simple exponential kinetics $A - B\exp(-kt)$, where $A = p_F$ is the saturation value of the contact formation probability in the folded state and $B = p_F - p_U$ is the gain of the former quantity in going from the initial unfolded state to the final folded state. k is then the characteristic rate of this simple exponential kinetics, the "contact folding rate". We entirely revised all

parts of the manuscript dealing with this analysis (Abstract, Results, Discussion, Materials Methods) and added two supplementary files showing the exponential fits to the contact formation probability curves for all native contacts (one file for SH3, one file for RD1). The revised Fig. 7 (Fig. 8 in the original version) shows now two examples of exponential fits for a trap-avoiding "red" contact and a first-entangling "magenta" one. The revised Fig. 8 (Fig. 9 in the original version) shows the results of the new analysis. The main message is maintained, that is all trap-avoiding contacts fold at a significantly faster characteristic rate with respect to all entangled contacts. p_U turns out instead to be the relevant parameter which rationalizes the emergence of the first-entangling "magenta" contacts. The revised Fig. 8 shows that the latter display a larger p_U value than almost all other entangled contacts, which, in general, have a very low p_U. The p_U values, and the associated standard deviations which turn out to be smaller than the marker symbols in the plot, are evaluated by sampling 1500 conformations from the equilibrium unfolded state at $T > T_f$, with exactly the same procedure used to select the initial conditions for the refolding trajectories. With hindsight, the lag time t^* obtained in the previous analysis from the logistic fits was related to p_U (the higher p_U, the smaller t^*). The picture emerging from the revised analysis is remarkably clear; any "trap-avoding" contact does fold faster then any entangled contact, along the fast folding pathway. On the other hand, provided trap-avoiding contacts are correctly folded, fluctuations already present in the unfolded state allow "early-entangling" contacts to generate the native entanglement.

11. **I also found it lacking to combine all 100 refolding trajectories into Fig 5. In the SI, please include the plots for the 100 trajectories separately, grouped according to the four types of folding pathways shown in Table 1.**

    We agree that providing examples of the refolding trajectories in the $Q, \langle G' \rangle$ phase space is useful to enhance the understanding of Figure 5. Therefore, we added Figure S8 to the SI that collects four examples for each folding pathway. Since we have 100 refolding simulations, we find it clearer to include just a few examples. Nevertheless, we have updated the data repository (URL: `https://researchdata.cab.unipd.it/984/`) to include all equilibrium and refolding Q and $\langle G' \rangle$ time series for both proteins, including a file for refolding RD1 trajectories specifying the classification as performed by our code. The latter is as well available in the repo.

12. **What are the standard deviations of the data points reported in Fig 9?**

    We agree that the info about standard deviations is important. In the previous version of the analysis, standard deviations were smaller than the marker size in the plot. Therefore, they were not drawn in the plot. In the updated analysis (see Fig. 8 in the revised manuscript), we now show standard deviations for both observables when these are larger with respect to the marker size. We specified this point in the caption of revised Figure 8, as well as how the errors are determined in the revised "Exponential fit to contact formation curves" section.
