# Supplementary material for: Folding kinetics of an entangled protein
Source: PLoS Comput Biol. 2023 Nov 13;19(11):e1011107. doi: 10.1371/journal.pcbi.1011107 (PMC10681328; doi:10.1371/journal.pcbi.1011107)
Supplement: Attachment — Submitted filename: review_v1.docx [file pcbi.1011107.s006.docx]

This manuscript reports Go-model simulations of the SH3 domain and an entangled protein, RD1 (Fig 1a). Gaussian entanglement was used to characterize the conformations of the protein, in addition to residue contacts. The two different folding pathways (threading and direct) reported are interesting. However, I found the manuscript rather difficult to follow, and I also have several concerns regarding the simulation protocol and analyses.

1. Figure 1: Please add the contact maps for the native structures of RD1 and SH3 for direct comparison to Fig 7. Clearly define what are considered “entanglement contacts.”
2. A previous paper used the maximum Gaussian entanglement to describe conformations. Why should one switch to the average? What are the pros and cons? Also, since there is a distribution of G’, can the average represent the distribution? For example, what if there are both positive and negative G’ in the distribution, which leads to an average of close to 0, but this is different from having a distribution centered around 0?
3. It’s mentioned two minor adjustments were made regarding how the Go-like model was constructed (as compared to reference 9.) Why were these adjustments necessary or better?
4. I saw only one folding event and one unfolding event in Fig S5. How can one determine the populations of the folded and unfolded states faithfully?
5. Equilibrium simulations were performed at Tf, and refolding simulations at 0.9*Tf. Why 0.9? What happens for refolding at 0.8*Tf, 0.7*Tf, etc.?
6. In the introduction, it seems quite some papers are from the same group. The authors should make it clear for clarity and transparency.
7. What is the smallest entangled peptide? Is this peptide amenable to all-atom MD?
8. How did the authors determine which pathways were observed (Table 1)? Was it by manual visual inspection, change point detection, or?
9. Overall, I found the movies the most helpful for understanding the paper. I wish the authors could find a way to show the conformations and steps on paper. I suggest the authors include representative structures above different segments of Fig 4. I suggest the authors combine Fig 5 and 6 (putting the structures next to each basin). Also, the entangled part (colored red and blue) of the IE- conformation should be aligned to that of the F. IE+ and IE- should be visualized so that one can see they are mirror images. A label of “threading” can be added next to the IT🡪F arrow; a label of “direct” can be added next to the U⬄IE- arrow.
10. Fig 9: Very difficult to tell which circles are magenta and which are red.
11. I also found it lacking to combine all 100 refolding trajectories into Fig 5. In the SI, please include the plots for the 100 trajectories separately, grouped according to the four types of folding pathways shown in Table 1.
12. What are the standard deviations of the data points reported in Fig 9?
